# Genome-wide analysis of long intergenic non-coding RNAs in chickpea and their potential role in flower development

**DOI:** 10.1038/srep33297

**Published:** 2016-09-15

**Authors:** Niraj Khemka, Vikash Kumar Singh, Rohini Garg, Mukesh Jain

**Affiliations:** 1 School of Computational and Integrative Sciences, Jawaharlal Nehru University, New Delhi-110067, India; 2 National Institute of Plant Genome Research, Aruna Asaf Ali Marg, New Delhi-110067, India

## Abstract

Non-coding RNAs constitute a major portion of the transcriptome in most of eukaryotes. Long non-coding transcripts originating from the DNA segment present between the protein coding genes are termed as long intergenic non-coding RNAs (lincRNAs). Several evidences suggest the role of lincRNAs in regulation of various biological processes. In this study, we identified a total of 2248 lincRNAs in chickpea using RNA-seq data from eight successive stages of flower development and three vegetative tissues via an optimized pipeline. Different characteristic features of lincRNAs were studied and compared with those of predicted mRNAs in chickpea. Further, we utilized a method using network propagation algorithm to reveal the putative function of lincRNAs in plants. In total, at least 79% of the identified chickpea lincRNAs were assigned with a putative function. A comprehensive expression profiling revealed differential expression patterns and tissue specificity of lincRNAs in different stages of flower development in chickpea. In addition, potential lincRNAs-miRNA interactions were explored for the predicted lincRNAs in chickpea. These findings will pave the way for understanding the role of lincRNAs in the regulatory mechanism underlying flower development in chickpea and other legumes.

Recent study of transcriptional landscape in human revealed that most of the genome sequence can transcribe into RNAs and have some biological role^1^. The identification and characterization of all non-coding transcripts along with coding transcripts is indispensable for the comprehensive understanding of biological processes and their mechanisms. Based on length, non-coding transcripts can be classified into small and long non-coding RNAs. Long intergenic/intervening non-coding (linc) RNAs represent a class of long non-coding transcripts transcribed from the intergenic regions of protein coding genes^2^,^3^ with minimum length of 200 bp and lack coding potential. Thousands of lincRNAs have been identified in animals and plants^2^,^4^,^5^,^6^,^7^. A few studies have revealed involvement of lincRNAs in gene regulation by wide variety of mechanisms at transcriptional and post-transcriptional level, such as formation of chromatin modifying complexes, attachment with promoter binding complex and acting as miRNA sponges to protect mRNAs from degradation^8^,^9^,^10^. Several evidences suggest that lincRNA participates in different biological processes, such as cell cycle regulation, embryonic development, cell pluripotency and aging in animals^2^,^8^,^11^.

In contrast to extensive investigation of lincRNAs in animals, only a few such studies have been performed in plants. Recently, lncRNAs have been predicted in different biological contexts in quite a few plant species^6^,^7^,^12^,^13^,^14^. A few studies have provided evidence for the role of lncRNAs in stress response, male sterility and phosphate homeostasis in plants^13^,^15^,^16^. For example, lncRNAs, *COOLAIR* and *COLDAIR*, were found to repress the expression of *FLOWERING LOCUS C (FLC)* gene through an epigenetic silencing mechanism, thereby regulating flowering in Arabidopsis^17^. *Mt4* lncRNA in *Medicago truncatula* was induced by phosphate starvation and acted as decoy of miRNA-399 to allow the accumulation of *PHO2* mRNA, which is important for phosphate uptake^16^. *LDMAR (Long-Day Specific Male fertility Associated RNA)* was found to be important for normal pollen development in rice under long day conditions^13^,^18^. Due to recent demonstration of their important biological roles in various aspects of development, lncRNAs have become the focus of plant biology research.

Chickpea (*Cicer arietinum*) is second most widely grown legume and is an important source of dietary proteins and fibers. High nutritional and economical value makes it extremely important for food security, especially in developing countries. The availability of draft genome and transcriptome sequences of chickpea provides an opportunity to perform advanced genomic research^19^,^20^,^21^. Further, transcriptome analysis have been performed to understand the molecular basis of some agronomic traits, such as flower development and abiotic stress responses^13^,^22^,^23^. Recently, small non-coding RNA component (miRNAs) in various tissues/organs have also been characterized in chickpea^24^,^25^. However, the discovery and annotation of lncRNAs still remains obscure in chickpea as of now.

In this study, we identified lincRNAs from 11 different tissue samples using RNA-seq data in chickpea. Different properties of lincRNAs and their comparative analysis with coding RNAs were carried out. The differential expression patterns and tissue specificity of chickpea lincRNAs was established. The functional annotation of identified lincRNAs was performed using network propagation algorithm. Furthermore, the interactions of identified lincRNAs and chickpea miRNAs were analyzed to reveal the potential regulatory aspects of lincRNAs. Our study provides the first systematic study of lincRNAs in chickpea and lay the foundation for further studies to elucidate their precise functions.

## Results and Discussion

### Genome-wide discovery of lincRNAs in chickpea

**Figure 1:**
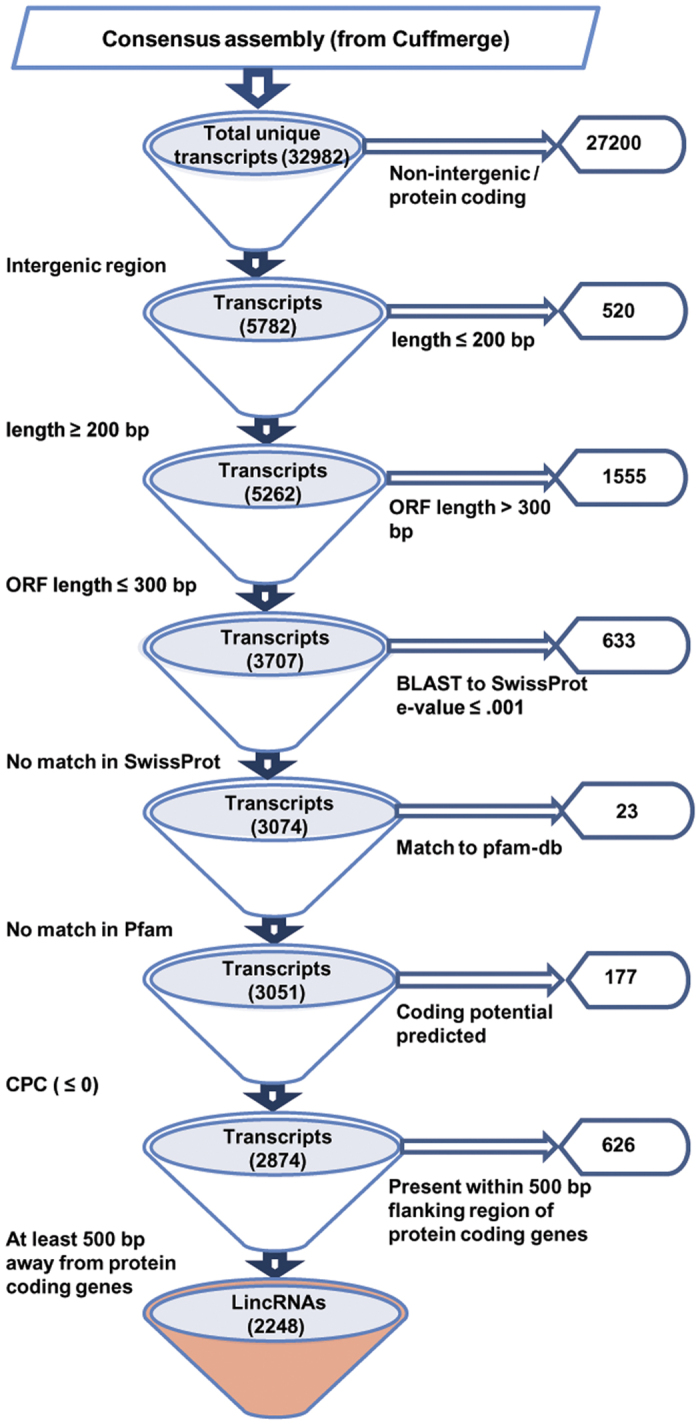
Strategy used for identification of lincRNAs in chickpea. All the steps followed for the identification of lincRNAs in chickpea are shown in the form of a flowchart. Numbers represent number of transcripts.

High-throughput sequencing technologies are the powerful tools to detect novel transcripts quantitatively. These transcripts contain both protein coding and non-coding portion. LincRNAs represent a class of non-coding transcripts with minimum length of 200 nt originating from intergenic region. For comprehensive identification of lincRNAs in chickpea, RNA-seq data (>350 million reads) obtained from 11 different tissues samples^26^. The samples including three vegetative tissues [germinating seedling (GS), young leaves (YL) and shoot apical meristem (SAM)] and eight stages of flower development [Flower bud (FB1-FB4) and Flower (FL1-FL4)], were utilized (Supplementary Table S1). The RNA-seq reads for each sample were processed via TopHat2 and Cufflinks programs using chickpea (Kabuli CDC frontier) genome as reference. The transcriptome assembly obtained after Cuffmerge was used to retain a set of unique and non-overlapping transcripts. This resulted in a total of 32984 transcripts with an average length of 1435 bp. From the total transcripts, putative intergenic transcripts (5782) were extracted and subjected to an optimized pipeline (Fig. 1) to identify lincRNAs. The pipeline included different filtering criteria for lincRNAs identification. The transcripts with length ≤200 bp and putative open reading frame (ORF) length of >300 bp were removed as transcripts having ORF length >300 bp might encode for a functional protein. This resulted in a total of 3707 chickpea transcripts, which were subjected to BLAST search in SwissProt and hmmscan in Pfam. At least, 633 and 23 transcripts shared sequence similarity with annotated proteins and protein domains, respectively, available in these databases and hence discarded. In the next step, coding potential of the remaining 3051 transcripts was predicted using Coding Potential Calculator (CPC). A total of 177 transcripts showing CPC score >0 and additional 626 transcripts present within 500 bp flanking regions of the predicted coding genes were removed. Finally, a total of 2248 transcripts were retained as putative chickpea lincRNAs and designated as Ca_linc_0001 to Ca_linc_2248 (Supplementary Data 1).

### Characteristic features of chickpea lincRNAs

**Figure 2:**
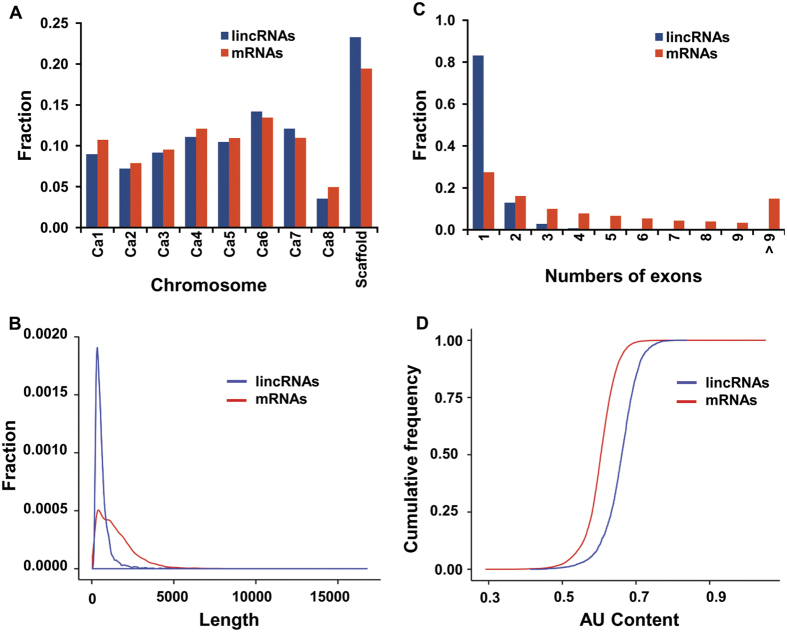
Features of chickpea lincRNAs and comparative analysis with mRNAs. (**A**) Chromosome-wise distribution of lincRNAs and mRNAs. (**B**) Length distribution of lincRNAs and mRNAs transcripts. (**C**) Distribution of numbers of exons in lincRNA and mRNA transcripts. (**D**) Cumulative frequency of AU in lincRNA and mRNA transcripts.

We investigated different features of identified lincRNAs (2248) and compared them with those of mRNAs in chickpea. Both, lincRNAs and mRNAs showed similar distribution pattern across different chromosomes on the chickpea genome (Fig. 2A). The length of lincRNAs varied from 201 bp to 5663 bp with majority (>88%) of them having length ≤1000 bp (Fig. 2B). The mean length of lincRNAs was 614 bp, which was quite less as compared to mean length of mRNAs (1495 bp). The lesser mean length of predicted chickpea lincRNAs was similar to that reported earlier in other plants, such as rice (800 bp), cucumber (322 bp) and *Ganoderma* (609 bp)^6^,^7^,^27^. However, the mean exon length of lincRNAs (395 bp) was substantially higher as compared to mRNAs (244 bp) in chickpea. The presence of more number of exons might have resulted in lesser exon length in mRNAs. Similar observation has been reported in rice with mean exon length of lincRNAs and mRNAs being 322 and 159 bp, respectively^6^. The majority (81%) of lincRNAs in chickpea were single exonic (Fig. 2C), consistent with earlier reports in maize (81%) and cucumber (89%)^7^,^14^. The average exon count of lincRNA was 1.22 as compared to 4.97 for mRNAs in chickpea. The chickpea lincRNAs transcripts were AU rich as compared to mRNAs (Fig. 2D) similar to the predicted lincRNAs in Arabidopsis, cucumber, rice and maize^5^,^6^,^7^. Various characteristic features, including genomic location, length, number of exons and GC content of all the chickpea lincRNAs have been given in Supplementary Table S2.

### Differential expression and tissue specificity of chickpea lincRNAs

To investigate the putative function of chickpea lincRNAs, their expression patterns were explored in all the 11 tissues of chickpea (GS, SAM, YL, FB1- FB4 and FL1- FL4) using RNA-seq data. For estimation of expression level, number of fragments per kilobase of transcripts per million mapped reads (FPKM) for each lincRNA was determined using Cufflinks (Supplementary Table S2). The number of lincRNAs expressed in each tissue varied from 1267 in young leaves (YL) to 1779 in flower bud (FB4) (Supplementary Fig. S1). The number of expressed lincRNAs was higher in tissues representing actively dividing cells (SAM and GS) and stages of flower development. Earlier report of lincRNAs in different plant species also showed that reproductive tissues contain higher number of lincRNAs as compared to other tissues^5^,^6^,^7^,^14^,^28^. Based on the expression level, lincRNAs were divided into five classes, very low (<2 FPKM) to very high (>20 FPKM) (Supplementary Fig. S1). The expression profile showed that a large number of lincRNAs were expressed at very low level, while smaller number of lincRNAs expressed at higher expression level (>5 FPKM). The expression pattern of chickpea lincRNAs showed similarity with that of lincRNAs identified in different plants, such as Arabidopsis, cucumber, maize and rice^6^,^7^,^14^.

**Figure 3:**
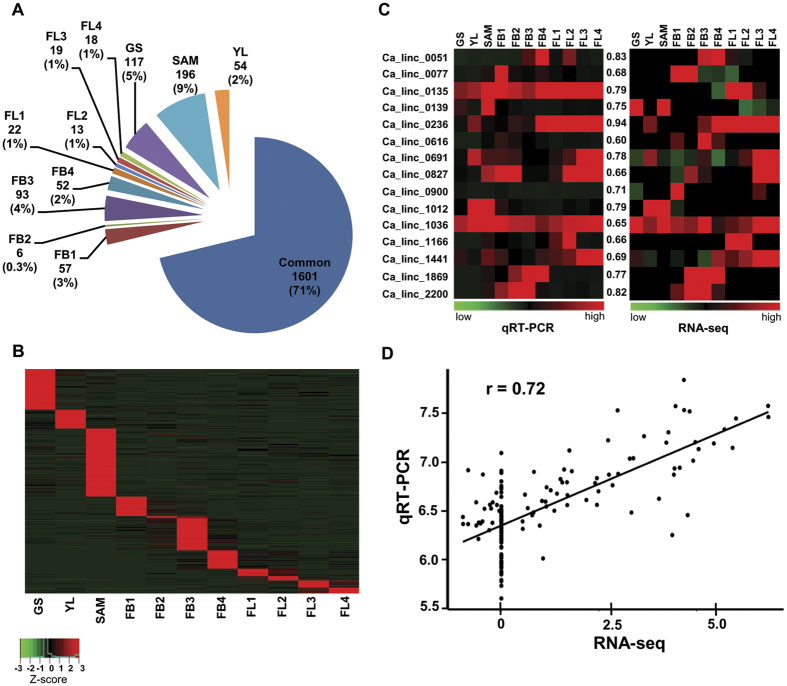
Tissue-specific expression of lincRNAs. (**A**) Number and percentage of lincRNAs showing specific expression in different tissues. (**B**) Heatmap of lincRNAs showing specific expression pattern in different tissues. Color scale representing Z-score is given at the bottom. (**C**) Heatmaps showing expression patterns in qRT-PCR and RNA-seq experiments for 15 lincRNAs. The correlation between the expression pattern obtained by qRT-PCR and RNA-seq for each lincRNA is given in the middle. (**D**) Overall correlation between qRT-PCR and RNA-seq data. Each dot represents one data point (normalized expression level of a lincRNA in a particular tissue obtained via RNA-seq and qRT-PCR).

To study differential expression pattern and tissue specificity of lincRNAs, we calculated the tissue specificity index (TSI) for all the chickpea lincRNAs. The TSI differentiates between housekeeping genes and tissue-specific genes. The TSI value of 0 represents housekeeping genes due to similar expression in all tissues, whereas TSI value of 1 represents strictly tissue-specific expression. Chickpea lincRNAs having TSI score ≥0.9 were considered as tissue-specific. Applying this stringent criterion, at least 641 lincRNAs (29%) were identified as tissue-specific (Fig. 3A). A heatmap depicting expression pattern of tissue-specific lincRNAs has been shown (Fig. 3B). The largest number of tissue-specific lincRNAs were detected in SAM (196) followed by GS (117). This may be because, these tissues can act as primordial cells and various tissues originate from these tissues, which suggests putative role of lincRNAs in tissue specification^29^,^30^. At least 13% of total lincRNAs showed tissue-specific expression during various stages of flower development (Fig. 3A). A comparative analysis revealed that a larger fraction (0.2–9%) of lncRNAs exhibited stage-specific expression (present study), as compared to the protein coding mRNAs^26^ (0.04–1.5%). Different reports suggest that lincRNAs expressed in a specific tissue can perform specialized functions. For example, functional analysis of lincRNA, *XLOC_057324*, expressed specifically in panicle and pistils of rice showed decreased fertility and panicle development in mutant rice^6^. Likewise, lncRNA Zm401, which is specifically expressed in anther, was found to be associated with male sterility in maize^18^. The higher expression of *IPS1* lncRNA in Arabidopsis shoot was found to be important for phosphate homeostasis^16^. A large portion of tissue-specific lincRNAs suggested their role in regulation of important characteristic processes in a particular tissue/developmental stage in chickpea.

To gain more insights into the role of chickpea lncRNAs in flower development and other biological processes, we identified soybean orthologs of chickpea mRNAs co-expressed with tissue/stage (FB, FL and SAM) specific lincRNAs. The top 10 co-expressed mRNAs were extracted from the mRNA-lincRNA co-expression matrix for each set of lincRNAs specific to FB, FL and SAM in chickpea. These mRNAs were searched for their orthologs (BLASTX, E-value < e^−10^) against the differentially expressed transcripts (2951) reported in the time series RNA-seq data of SAM in soybean^31^. A total of 292, 184 and 117 chickpea mRNAs co-expressed with lincRNAs specifically expressed in FB, FL and SAM, respectively, shared significant similarity with differentially expressed transcripts in soybean (Supplementary Table S3). Many of these orthologs represented well known proteins (FT, AGL6, AP1, SVP and TCP1) associated with various aspects of flower development. These results strengthen the idea of the crucial role of lincRNAs in flower developmental process in chickpea.

### Validation of differential expression patterns

We randomly selected 15 chickpea lincRNAs for validation of their expression patterns using quantitative real time polymerase chain reaction (qRT-PCR). We observed similar expression pattern in qRT-PCR analysis as that of RNA-seq data for the selected lincRNAs (Fig. 3C). Overall, the results of qRT-PCR analysis were in good agreement (r = 0.72) with RNA-seq (Fig. 3D). For example, based on RNA-seq data analysis, Ca_linc_0051 and Ca_linc_0139 showed specific expression in FB4 and SAM, respectively. Similar expression patterns were confirmed via qRT-PCR analysis as well (Fig. 3C,D).

### Functional annotation of lincRNAs

Several studies have demonstrated that lincRNAs are involved in various biological processes^3^. Therefore, it is important to annotate the putative function of lincRNAs. Function prediction of lincRNAs using sequence homology based approach is trivial due to non-conservative nature of lincRNAs across different species. Recently, a few methods have been proposed for functional annotation of lincRNAs^32^,^33^. To infer the putative function of lincRNAs in chickpea, we adopted a recently proposed method for annotation of lncRNAs using network propagation algorithm^33^. This annotation method uses transcriptome and protein-protein interaction (PPI) data for the prediction of putative function of lincRNAs and showed improved accuracy compared to other available methods. We used this network propagation based method to annotate the putative function of predicted lincRNAs in chickpea. We integrated RNA-seq based expression and PPI data to form the bicolor network. On this bicolor network, global propagation algorithm was applied to infer the putative function (gene ontology) of lincRNAs.

**Figure 4:**
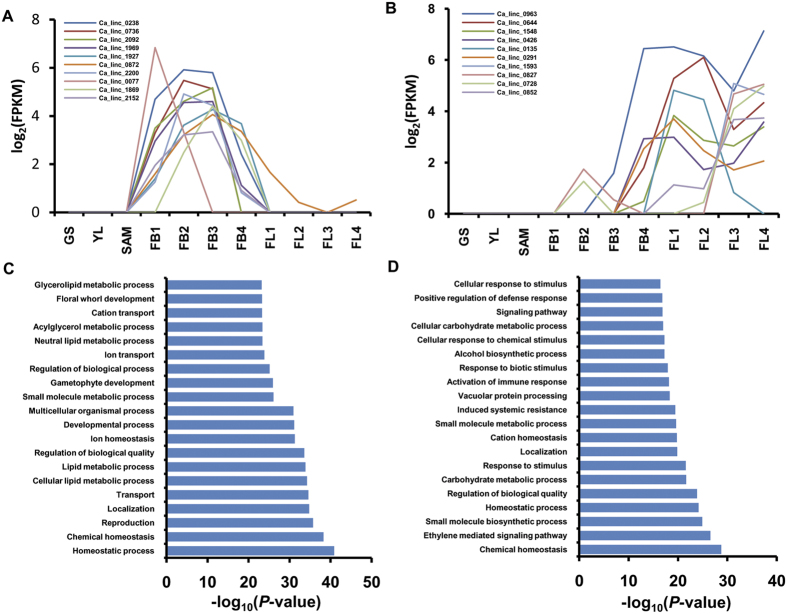
Flower bud and flower specific expression patterns of lincRNAs and GO enrichment analysis. (**A**,**B**) Expression pattern of 10 highly expressed lincRNAs in flower bud (**A**) and flower (**B**) stages of chickpea. Normalized expression (FPKM) of lincRNAs in different tissues has been presented in the line graph. Expression pattern of all the highly expressed lincRNAs in flower bud and flower stages of chickpea is given in Supplementary Fig S3. (**C**,**D**) Gene ontology enrichment for lincRNAs expressed only in flower bud (**C**) and flower (**D**) tissues. Top GO terms showing very high enrichment (P-value < e^−10^) are shown.

Using the network propagation based annotation method, putative function could be assigned to at least 1790 (79%) of chickpea lincRNAs (Supplementary Table S4). In general, the functional annotation revealed the predominance of GO terms related to different biological processes, such as, photorespiration, regulation of cell cycle, cell morphogenesis, RNA splicing, mRNA processing and histone modification along with a variety of metabolic processes among chickpea lincRNAs (Supplementary Fig. S2). Further, we explored the functional annotation of lincRNAs specifically expressed in flower bud (FB), flower (FL) and shoot apical meristem (SAM) in detail. The enrichment analyses showed that majority of lincRNAs are involved in biological processes in a stage-specific manner. Flower bud specific lincRNAs were found to be enriched in GO terms associated with homeostasis processes, developmental processes and floral whorl development with p-value < e^−10^ (Fig. 4A,C, Supplementary Fig. S3). The lincRNAs expressed specifically during flower development stages were enriched in GO terms, such as chemical homeostasis, different signaling pathways, developmental process, response to external stimuli and localization with p-value < e^−10^ (Fig. 4B,D, Supplementary Fig. S3). The SAM-specific lincRNAs were found enriched in GO terms associated with anatomical structure development, secondary metabolite process, regulation of timing of transition from vegetative to reproductive phase, regulation of timing of meristematic phase transition along with gibberellins biosynthetic process and auxin influx with p-value < e^−9^ (Supplementary Fig. S4). Previous report of coding genes showed that GO terms associated with the reproductive structure and floral whorl development were highly enriched at flower bud (FB1-FB4) stages, whereas GO terms associated with different signaling pathways along with reproduction and post-embryonic development were enriched at flower development stages (FL1-FL4)^26^. Likewise, reports of SAM specific protein-coding genes in chickpea and soybean also showed that GO terms associated with anatomical structure development, cell differentiation, cell proliferation, floral meristem determinacy, reproductive development process along with GO terms associated with gibberellins and auxin hormone were highly enriched^31^,^34^. The correlation of functional enrichment between co-expressed coding transcripts and lincRNAs indicates accuracy of our functional annotation method. These results also revealed that lincRNAs are involved in several biological processes occurring at different stages of flower development in chickpea. A few lncRNAs have been characterized for their biological functions in plants. The regulatory role of lncRNAs, such as *COOLAIR, LDMAR* and *IPS1* in flowering timing during cold stress, fertility and phosphate homeostasis, respectively, have been validated in different plants^13^,^16^,^17^. The enrichment of GO terms associated with the specific processes with different tissues/stages of development can provide significant enhancement in knowledge and functioning of lincRNAs in plants.

### LincRNAs as potential regulator of miRNAs

**Figure 5:**
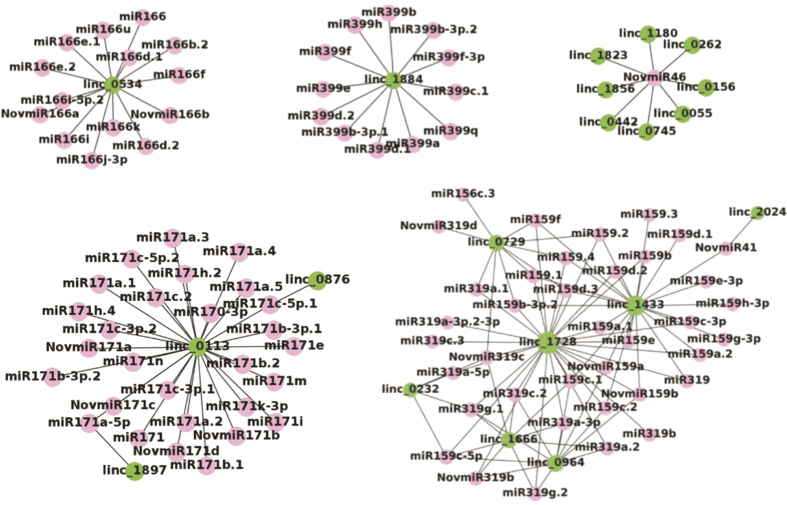
LincRNA - miRNA interaction network. The network includes lincRNAs and miRNAs. Red circle nodes represent miRNAs and green circle nodes represent lincRNAs. Examples of one lincRNA to many miRNAs, many lincRNAs to one miRNA, and many lincRNAs to many miRNAs interactions are shown. The complete network of chickpea lincRNAs and miRNAs has been given in Supplementary Fig. S5.

**Figure 6:**
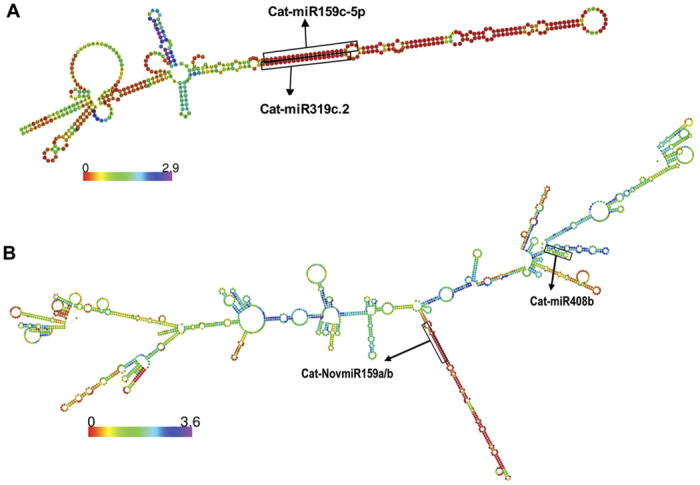
LincRNA – miRNA binding site prediction. Representative examples showing the secondary structure of lincRNAs with binding sites of miRNAs are given. Color coding shows the positional entropy of sequence (**A**) Secondary structure of Ca_linc_1666 with binding sites of Cat-miR159c-5p and Cat-miR319c.2. (**B**) Secondary structure of Ca_linc_0964 with binding sites of Cat-miR159a/b and Cat-miR408b.

Recent studies have shown that lincRNAs can act as targets or decoy of miRNAs to mimic mRNAs and therefore regulate gene expression^35^,^36^,^37^,^38^. The lincRNAs interact with miRNAs to regulate biological processes, such as plant growth, differentiation and reproduction^36^. In this study, predicted lincRNAs of chickpea were used to identify the potential targets of miRNAs using psRNATarget^24^,^39^. A total of 144 lincRNAs were identified as putative targets of 236 chickpea miRNAs (Supplementary Table S5). Multiple interaction patterns, such as one-to-one, one-to-many and many-with-many, were recognized. This resulted in a total of 349 lincRNA-miRNA pairs (Fig. 5, Supplementary Fig. S6). Majority of miRNAs involved in interaction with lincRNAs were from miR-156, miR-159, miR-166, miR-167, miR-170, miR-171, miR-172 and miR-319 families. Several reports have suggested regulatory roles of these miRNAs families in flower development in plants^40^,^41^,^42^. Many of the chickpea lincRNAs were found to be associated with GO terms as that of known function of their interacting miRNAs. For example, miR-159 and miR-319 are highly conserved miRNA families that have important role in plant growth, morphogenesis and reproduction^43^. Members of these families, miR-159c and miR-319c, were found to target Ca_linc_1666 (Fig. 6A). The lincRNA, Ca_linc_1666, was associated with GO terms, such as specification of petal number (GO:0048834) and male meiosis cytokinesis (GO:0007112). Similarly, Ca_linc_0964 harbor binding sites of miR-159-a/b and miR-408b (Fig. 6B). Earlier studies revealed that miR-408 family miRNAs are associated with stress response in chickpea^44^. The GO terms associated with lincRNA, Ca_linc_0964, included reproduction related GO terms (seed development (GO:0048316), DNA replication associated terms (GO:0006260, GO:0006261 and GO:0006275)) and stress related GO terms (response to UV-B (GO:0010224) and defense response (GO:0009814)). These examples suggested that function of lincRNAs can also be assigned by knowing their interacting miRNAs and their function.

Some of the miRNAs that target lincRNAs were found to be conserved in soybean. The orthologs of at least 12 SAM related soybean miRNAs reported earlier^42^ were found to target 44 lincRNAs in chickpea (Supplementary Table S5). For example, miR319a and miR159a targeted SAM-specific chickpea lincRNA, Ca_linc_1728.

Earlier reports also showed role of lincRNA-miRNA interactions in different biological functions. In Arabidopsis, it has been demonstrated that miR-399 targets *IPS1* lincRNA instead of *PSO4* mRNA to increase phosphate uptake during phosphate starvation^16^. Likewise, miR-160 and miR-164 were found to cleave lncRNAs associated with reproductive processes in rice^6^. Genome-wide analysis of degradome data in maize identified 166 lncRNAs as targets of 165 miRNAs, while 34 lncRNAs acted as decoy of 33 miRNAs^35^. The interaction of miRNAs with their target lincRNAs suggests that the lincRNAs can disrupt miRNA-mRNA interactions to regulate gene expression and biological processes. Overall, our analysis provides evidence for interaction of lncRNAs and miRNAs in chickpea and suggests their putative roles in various aspects of flower development.

## Conclusions

In this study, a total of 2248 lincRNAs were identified in chickpea using RNA-seq data following stringent criteria. At least 1790 lincRNAs (79%) could be assigned with a putative function. The expression profiling revealed tissue-specific expression of a large number of lincRNAs in distinct tissues. Several chickpea lincRNAs were identified as targets of miRNAs and implicated them in the regulatory network of various developmental and reproductive processes. This study will provide an extensive resource for lincRNAs in chickpea and allow further study to gain insights into regulatory aspects of flower development in chickpea and other legumes. Further experiments focused on individual lincRNA are needed to elucidate their exact function.

## Methods

### Transcriptome data processing and lincRNA identification pipeline

RNA-seq data (fastq files) of 11 tissues samples, were downloaded from the Gene Expression Omnibus (GEO) database under the accession number GSE42679^26^. These samples included three vegetative tissues, germinating seedling (GS), young leaves (YL) and shoot apical meristem (SAM), and eight successive stages of flower tissues from closed flower bud (FB1) to drooped flower (FL4) as described^26^. The RNA-seq data consisted of >350 million high-quality reads (Supplementary Table S1). The reads from each sample were filtered to remove adapters and low-quality reads using NGSQC Tool Kit^45^ followed by mapping on the chickpea (Kabuli CDC frontier) genome^19^ using splice read aligner TopHat2^29^. The transcriptome of each tissue was assembled using Cufflinks and merged together using Cuffmerge to generate the consensus transcriptome assembly.

The pipeline employed for the identification of lincRNAs from RNA-seq data has been described in Fig. 1. Briefly, from total transcripts, intergenic transcripts were extracted using class code “u”. The transcripts having length shorter than 200 bp and overlapping with known mRNAs were discarded from the intergenic transcripts. The ORF length of filtered transcripts was calculated using OrfPredictor webserver^46^ (http://proteomics.ysu.edu/tools/OrfPredictor.html). The transcripts having ORF length >300 bp were discarded. The SwissProt^47^ (BLASTX, E-value ≤ e^0.001^) and Pfam^48^ databases (hmmscan, E-value ≤ e^0.001^) were used to filter transcripts with matched protein and protein domain family, respectively. The remaining transcripts were used for coding potential calculation using Coding Potential Calculator (CPC), (http://cpc.cbi.pku.edu.cn)^49^. The transcripts having CPC score of >0 was considered as coding transcript and hence discarded. Transcripts present within the flanking region (500 bp) of coding genes were also removed from the remaining intergenic transcripts, as there may be chances of extension of transcription start site (TSS) for protein coding genes in improved annotation. Different in-house perl scripts were used for analysis.

### Tissue specificity index

Tissue specificity of the putative lincRNAs was evaluated according to the tissue-specific index (TSI) as described previously^7^,^50^. The TSI was calculated based on the expression value of each lincRNAs in different tissues. The value of TSI of lincRNAs varies from 0 for housekeeping genes to 1 for strict tissue-specific genes. The lincRNAs showing a TSI ≥0.9 were considered as tissue-specific lincRNAs.

### qRT-PCR analysis

To validate the expression profile of predicted lincRNAs, total RNA was isolated from germinating seedling (GS), shoot apical meristem (SAM), young leaf (YL), flower buds (FB1-FB4; where FB1, FB2, FB3, and FB4 were flower buds of different sizes), and flowers (FL1–FL4; where FL1, young flower having closed petals; FL2, flower with partially opened petals; FL3, mature flower with faded petals; FL4, drooped flower with senescing petals), as described^26^. Total RNA samples were checked for quality and quantity as described previously^26^. Gene-specific primers were designed using Primer Express (Version 3.0) software, and real-time PCR analysis was performed using 7500 Fast real time PCR system (Applied Bio-systems) using at least two biological replicates and three technical replicates (for each biological replicate) of each sample as described^51^. The expression of *Elongation Factor-1 Alpha* (*EF1α*) was used as internal control gene for normalization^51^. The primer sequences used for the analysis are provided in Supplementary Table S6. The correlation between expression profile of lincRNAs measured by RNA-seq and qRT-PCR were analyzed using R statistical package.

### Functional annotation of lincRNAs

Functional annotation of lincRNAs was performed using network propagation based annotation method^33^. In this method, gene co-expression and protein-protein interaction (PPI) data were used. The gene co-expression data and PPI data were integrated for bi-color network formation. Network propagation algorithm was applied on the bi-color network to obtain the annotation. Expression data (FPKM) of coding RNAs and lincRNAs were obtained from the chickpea RNA-seq data using Cufflinks. PPI data and GO annotations (biological process) for chickpea proteins were obtained for their best orthologs in Arabidopsis using String^52^ and TAIR^53^ databases, respectively. The Spearman correlation coefficient was calculated based on FPKM values for all pair of lincRNA/mRNA. Top 0.5% percentile of the absolute value of correlation coefficient was used to make the co-expression bicolor network. GO terms for all annotated mRNAs were transferred to the bi-colored network. The lincRNA nodes connected with mRNAs were annotated with their first degree neighbor annotation. Further, the PPI score between chickpea proteins were normalized for further processing as described^33^. To generate a weighted matrix, transcriptome based correlation matrix was combined with the PPI matrix and scores were normalized using min–max linear normalization algorithm^54^. The global propagation algorithm^54^ was applied to the bi-colored network using weighted matrix for inferring the putative function of lincRNAs as described^33^. This process ranked associations between mRNA/lincRNA and all GO terms. All the lincRNAs were ranked according to the association scores with given functional category. The lincRNAs ranked in top 100 for a GO term were annotated with the same GO term for functional annotation (Supplementary Table S4).

### GO enrichment analysis

Gene ontology enrichment analysis of lincRNAs was done using Bingo plugin of Cytoscape (version 3.2)^55^. The GO terms with p-value < e^−10^ were considered significant. The lincRNAs which expressed only in FB and FL tissues with FPKM ≥3 and no expression in other tissues (FPKM ≤ 0.1) were considered for the GO enrichment. The lincRNAs expressed specifically in SAM (TSI ≥ 0.9) was considered for GO enrichment.

### Identification of lincRNAs as target of miRNAs

All the identified lincRNAs were analyzed for miRNA target sites using psRNATarget webserver^39^. The chickpea miRNAs sequences were taken from Jain *et al*.^24^. The miRNAs and lincRNAs were submitted to the psRNATarget and interaction were calculated using default parameters (maximum expectation = 3, allowed maximum energy to unpair the target site (UPE) = 25). The interaction network of lincRNAs and miRNAs in chickpea based on the miRNAs binding sites on lincRNAs was drawn using Gephi^56^ and R statistical package^57^.

## Additional Information

**How to cite this article**: Khemka, N. *et al*. Genome-wide analysis of long intergenic non-coding RNAs in chickpea and their potential role in flower development. *Sci. Rep.* **6**, 33297; doi: 10.1038/srep33297 (2016).

## Supplementary Material

Supplementary Information

Supplementary Table S2

Supplementary Table S3

Supplementary Table S4

Supplementary Table S5

Supplementary Data S1
