# Supplementary material for: Genome-wide analysis of long intergenic non-coding RNAs in chickpea and their potential role in flower development
Source: Sci Rep. 2016 Sep 15;6:33297. doi: 10.1038/srep33297 (PMC5024101; doi:10.1038/srep33297)
Supplement: Supplementary Information [file srep33297-s1.pdf]

Supplementary Information

Genome-wide analysis of long intergenic non-coding RNAs in chickpea and their potential role in flower development

Niraj Khemka[1], Vikash Kumar Singh[2], Rohini Garg[2], Mukesh Jain[1,2]*

[1]School of Computational and Integrative Sciences, Jawaharlal Nehru University, New Delhi-110067, India

[2]National Institute of Plant Genome Research, Aruna Asaf Ali Marg, New Delhi-110067, India

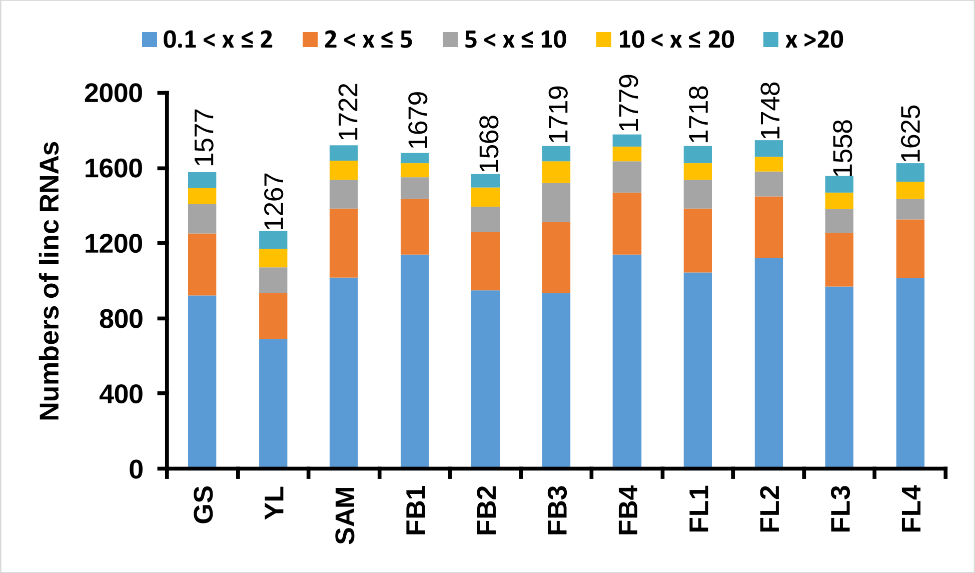

Supplementary Figure S1. Distribution of lincRNAs in different classes based on their expression level. Total number of expressed lincRNAs in each tissue has been indicated.

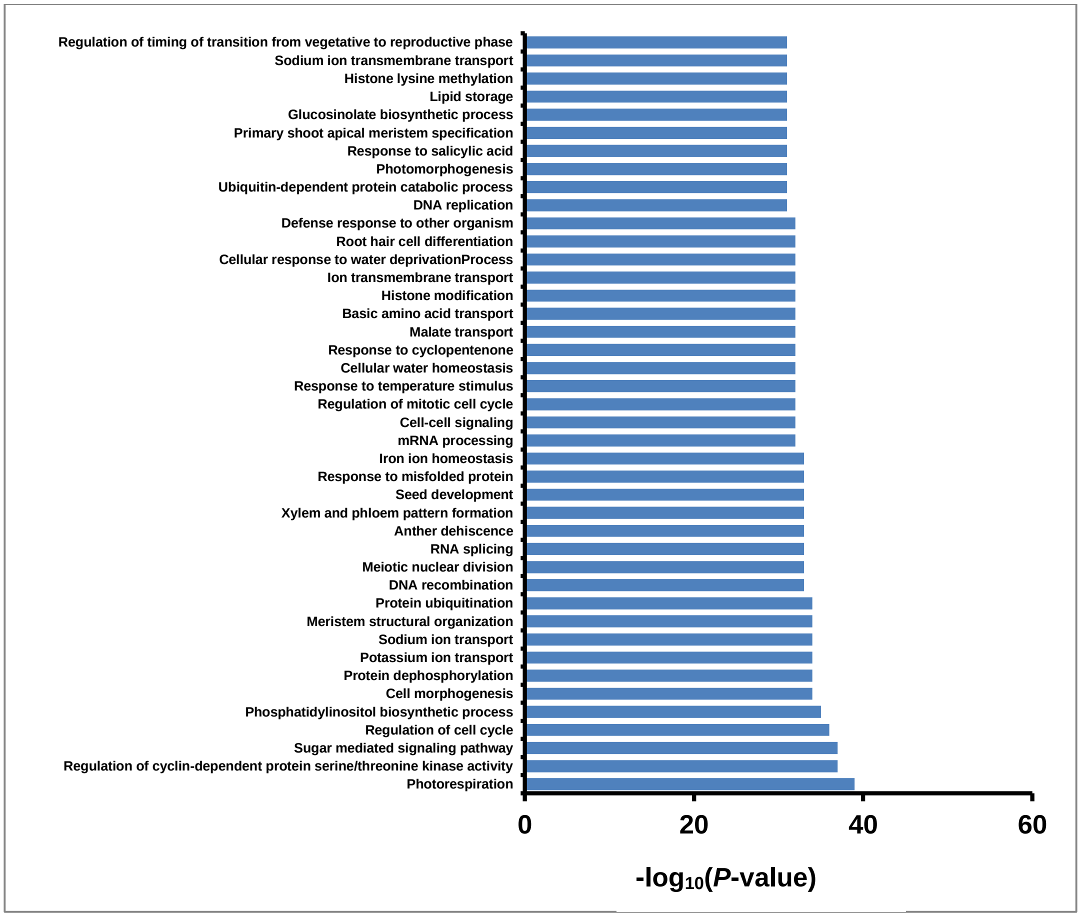

Supplementary Figure S2. Highly frequent GO terms in overall lincRNAs annotation.

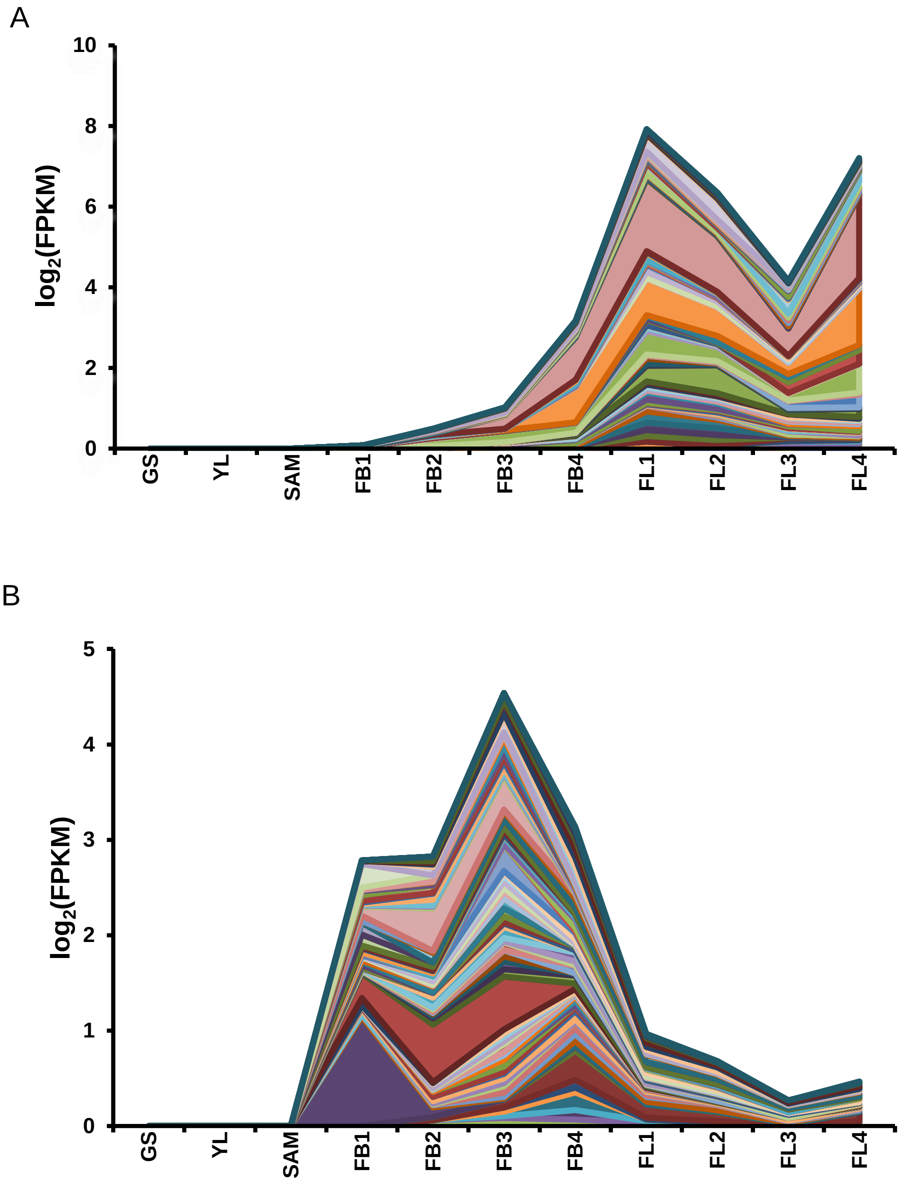

Supplementary Figure S3. Expression patterns of lincRNAs expressed specifically in flower bud (A) and flower (B) tissues of chickpea.

A

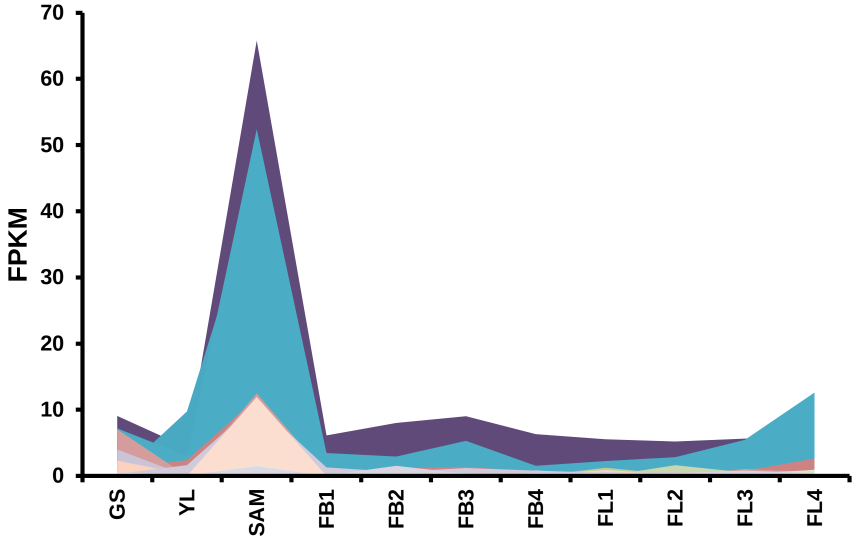

B

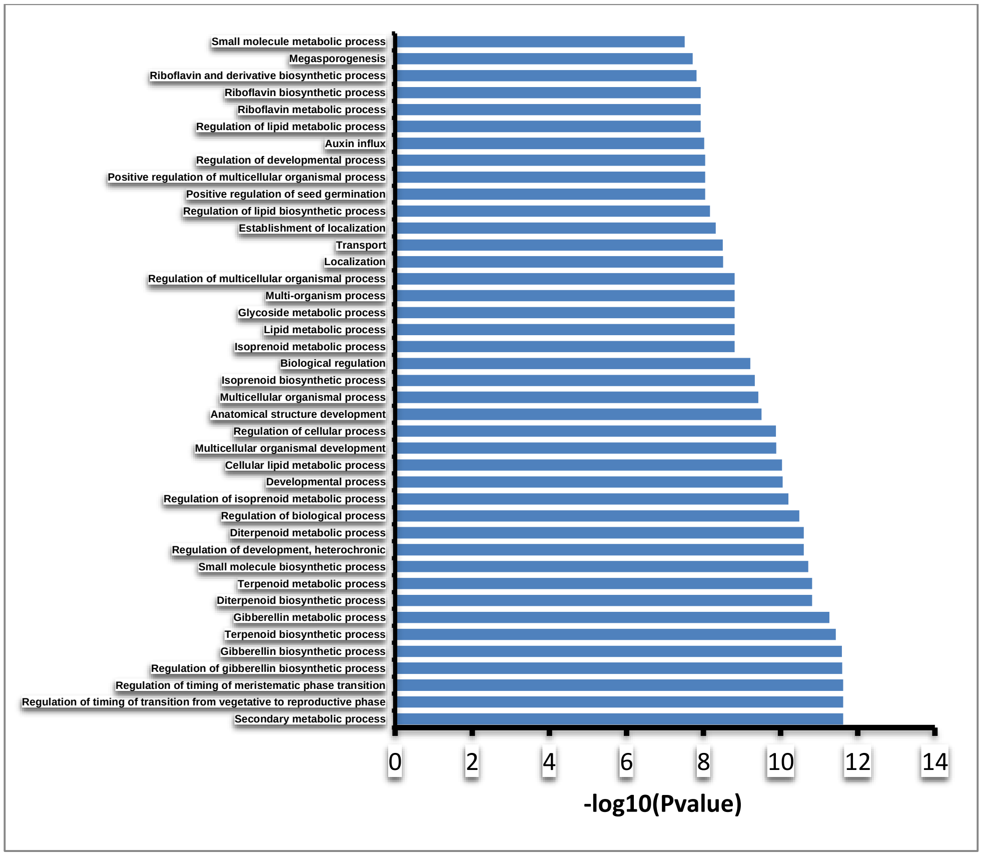

Supplementary Figure S4. Shoot apical meristem (SAM) specific lincRNAs expression pattern (A) and gene ontology enrichment analysis (B). Top GO terms showing very high enrichment are shown.

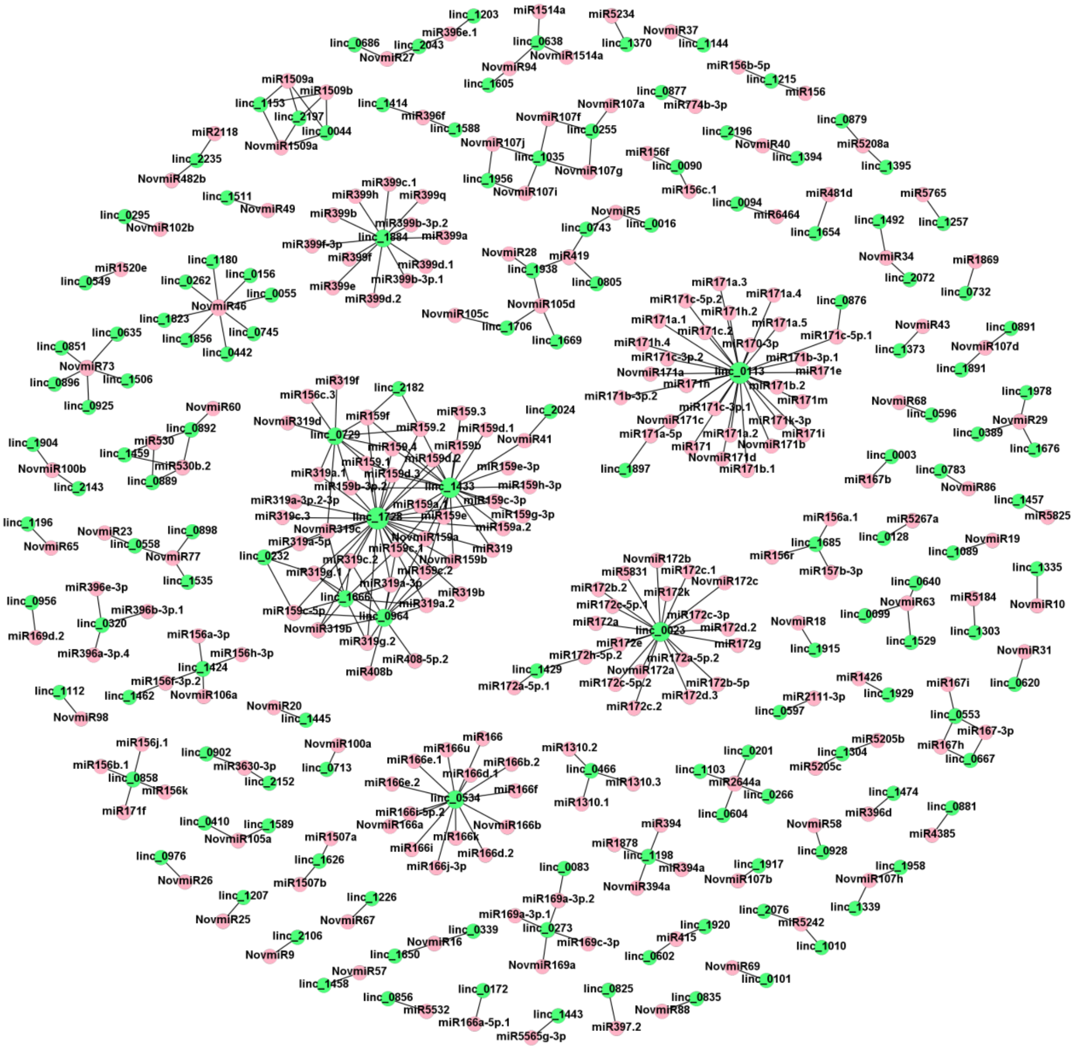

Supplementary Figure S5. LincRNA - miRNA interaction network in chickpea. Red circle nodes represent miRNAs and green circle nodes represent lincRNAs.

Supplementary Table S1. Summary of tissue samples and sequence data used with mapping statistics.

Tissue	Total reads	HQ reads	Total mapped	% mapped read
Germinating seedling (GS)	27889925	25954346	25321953	97.56
Young leaf (YL)	32762959	30339998	28892367	95.23
Shoot apical meristem (SAM)	36003749	34508868	33868015	98.14
Flower bud 0.4 cm (FB1)	41850211	36409370	34543919	94.88
Flower bud 0.6 cm (FB2)	17827737	16064057	15518412	96.6
Flower bud 0.8 cm (FB3)	41365615	39663080	38226953	96.38
Flower bud 0.8 cm open (FB4)	35211613	32605824	31846686	97.67
Flower unopened 1cm (FL1)	29091495	27624457	27029338	97.85
Flower opened 1cm (FL2)	33634177	32109896	31495523	98.09
Flower mature (FL3)	26396381	22418080	21244187	94.76
Flower drooped (FL4)	30245721	26865870	26406217	98.29

Supplementary Table S2. Genomic features and expression profiles of all 2248 lincRNAs identified in chickpea.

(Provided as separate MS Excel file)

Supplementary Table S3. Tissue (FB, FL and SAM) specific chickpea lincRNAs and their co-expressed chickpea transcripts (expression in 11 tissues) with their orthologs in soybean.

(Provided as separate MS Excel file)

Supplementary Table S4. Functional annotation (GO assignment) of chickpea lincRNAs.

(Provided as separate MS Excel file)

Supplementary Table S5. List of miRNAs targeting the predicted chickpea lincRNAs.

(Provided as separate MS Excel file)

Supplementary Table S6. Primers used in qRT-PCR validation of lincRNAs

LincRNA ID	Forward primer	Reverse primer
Ca_linc_0051	CAAAAGATGGACAATGGCGAAT	CACTCACGTGCCACATTGAAT
Ca_linc_0077	CACACTAAGAAAGACCAAAAATACAAAGA	ACAAGCAATTAATGTGGCAGCTAA
Ca_linc_0135	GAATGACACAATTCCATAATCACTAAAAC	CCAGTGAGAATTTCCTTGCCTTT
Ca_linc_0139	TTCAAGGGAGGAATCAATGTTATG	AAGAGCTCAAGATCCCAATGATG
Ca_linc_0236	TCCTTCTTTGGTTCCATTTTCC	GCTTCCCACTTCTTGAGTTCTTG
Ca_linc_0616	TGGGATTAGAGGCACACATG	ACTCCTTTTCATTTTATCTTTTTTGT
Ca_linc_0691	TTTTGGTAAACAACCTTCCACATTT	TTGAAGCCTAAGAAAGACAATATTGG
Ca_linc_0827	ATATTCTCACCCCCAACCACAT	TGGCCATTGTGTAATTTTGAGAGT
Ca_linc_0900	TGGAGAGGTGTAGGGTTGTTGA	CTTGCCACCCCTTACACTTTCT
Ca_linc_1012	TTGGATCCATTTGAAGTTAAGAGTT	CGAACCCACAAGGATCTCTGT
Ca_linc_1036	GGGAAAAATGGTCAGCAACAA	TTATTTCATGGCACATTTCACTCTTT
Ca_linc_1166	GATCATATACGTTAGCCCTGATTTCAG	AGTGACATTGGTTGAAGGTGAAAC
Ca_linc_1441	GGTCAAGATCAAGAGACAAGTCCAA	CCTGGTTGATCGGTTTGTTTTC
Ca_linc_1869	GAGAAGGTTCGGGATTCATCAT	GGTTGCCTTGCAAAATTAATACAA
Ca_linc_2200	GGCCCATGATCATTTAAGCAA	CCCCCTGACTTGATGTTTCTG

Supplementary Data S1. Sequences of predicted lincRNAs.

(Provided as separate text file)
