# Supplementary material for: Genome-wide analysis of long intergenic non-coding RNAs in chickpea and their potential role in flower development
Source: Sci Rep. 2016 Sep 15;6:33297. doi: 10.1038/srep33297 (PMC5024101; doi:10.1038/srep33297)
Supplement: Supplementary Data S1 [file srep33297-s6.doc]

Supplementary Dataset S1 : Sequences of predicted lincRNAs

>Ca_linc_0001

CTAAAATAACTCGCATGATCAAATATATCATCCGCTCTCCAAAAACAAATATTTGAACTATATCTCACAA

CAATCTTATACAAGAATATAAGAAAAAAAATAAAAGAATTAAATACAAAGTTAAAATATTTATAACTTGT

ACAGATTATAATAAAAAACTAGAGTCATATATATAAATATTGAGTTTCATCTTTCTTTCAATTCTAAGTA

ATATGAAACTATATATATAATAATTTGAACGGTGATTTTTTATCACATAAAACACTTGAGGTTTTTTCTA

CTTCATGGCTCAACTTTGATTCAATGGTTTACATCCAATTTTATTTTTCTAACGTTGTGTACTATGAACT

CAAGATATTTAAACAATGTCACTCGTTATTATGAAATATTCTTTTGCGATTCTACTGGACAAGTGCATCT

TGTGTTAGTGGAAGCTGAATCACATTTTCAACACACTCAATCAAATCTCTCAAGTTTCCTATCTTATGGA

AAAGAAATGATGAAGAGGTTTCATTGGAAGAACTTAATAAGGTGTCTAAATGTTGATTACATTTAAGAAT

AAGTGGATGTGGTCTAGAGGGCAAACTATTTGAGCGACCATGGAAACTGGATTTTGTGTTCAAAGAAGAA

GATGTTGCCATTTTGATTCCTTAAGAAAAGAGAAAGAGATCACTTAAGGCTTGATGTTGAAT

>Ca_linc_0002

ATCGAGTCTATGCTTTTCCTAGAAATCCTCCTCTTCCTGTTCTATTGATCAAAAATATCCAGCAGCAAGA

TAGCGCCGGTTTAGAATTCGCGAGAGATTTCAGTACGCTAAGTGAAACGGTCGATTTGCCGATTTTCTTT

TTCTTTTTTTTATTTTATATGGGGCGTGCAGCACTTTTCGATGTCACGCACAAGCATCAGGCTACTAAGA

CTTTTGTATGTACGAGCGGTGTTGAACCCTCTAACACCATGGCACCGATGTTCATGACGCCGTGAAACCT

TCCCGACGGCTGGCAAATATGGAACGCGCTGAACGATGGCGTCCTAATGGCGTCACTGTCGTTGTCACTG

CTGGAGGAGAGAAGCTGACCAGGAACTCCGGCGTGACGCGGAAGAGGAATGGAATTCCTGGTAGGATTCT

TGCCGCTCGAGGAGCAGAGCCCTCTGGAGGTCGTCCAAAGCCCGAATCCGGTCT

>Ca_linc_0003

CTCTTAGGCTTGTGAGATGATGAAATCATGTGCTGCATACAAGCCTAATTCGGATAGTACATTTCACTTT

ATGCTGCTGGGATATGTAGCTGAACACTCATGAAGGAAAATCCATATTGGATGGCTTTATAGAAAATAAT

CCAGACCCAATATTTTATAGTAGATGCCTTCTCATGCCATCTACTAATGTGATGCAAGGAGTTAACTAAT

TCCAATTTTCAAGGCACGTTTTTTACGGCTTAGCACATATACCACGCTTTTGCTTTTGATGGTCTTTGCT

ACTTAGTTTAGGCATCTAAAATGTTGTAATTTTAGATTCCTATGTTGCATGAAACATAGGAATTAGTTTT

CTTGATCAGTTATGGGTGGTAGCTTATTTACCTAAGCACCAAATGTAAAAGTGCACAGAGCTGGACAAAC

CACTCCAAATAGTTTTCGATAGGTATCAGCAGGCTCCTATGCCCTCTCAAAATGATGCTTCACAGATTTG

CAGCCATCGCCTTTTTTATTGGAGTTCTCACAAAGTATAGCTACTGCTATTGTATCTTTTTTATATTAAT

TTTAAATTTGTAAATCTGTTGAAGCCTTATCTTTTTGCTATTGATTTTGAATTTGTAATTTGTAAATCTG

TTTTAGCCTTGTCTTGTCTGTATATAGCTCTATATTCCTGTAACAAGATTTTGTTATCAGCTTCAGCTTT

ATCC

>Ca_linc_0004

AGAAAGACAAGTAATTAGAATGACAATTGTTTTATGTGTGTTTTTTGGGTGTGATAAAAAATAGTTGTTT

TATTTAGTTGAAGGTTCTGTATCATGAATCAAGTTTTGAAATAATTTTAACATTTTCTTTCTTATGTCAG

GATTATACTGCTCATTTTTCTTGGACCAGTGCTATTGTGACTGGATTTGCTGATGATTTCTGCCATAATC

CTTTTATGCTGAGCTATCTTTGTATTTTAGAGTATTCTTGTAATTCAAACAGTTTGAGAATACTATCTTG

TACACCTGTAAACATTTAATATTACAGTGCTATATATTCAATTTGATCAACTTCAATGGTGTCTGATGAT

TTGCATTGAACTGAGGCTTTCATTATATTACTTTTGCTTTTGTTTTAATGAACTTTATATTACTTTTGTG

TTTCCACCTCTAAATATATTCTTGAATTCACATGCAACCTTGTAGATTTATATTATTTATTCGTGGATAT

TCTTCCTAAGCAGTGAAGAATGGAGACTTTAGCTCAACTGGAAGCATTGTGTGAGAGGCTTTATAATTCC

CA

>Ca_linc_0005

GAAAGAGAGGGTACTGATCACTTCAAGCAAGATAGAATCTTAGGGCAGTTGAAGCCTCTGGGCTTAGCAC

TACTATGACCGAAGAAGACGATTGACTAAAGATAAATCGCAGGATAAACCGTGGGGAGATTAGTGAAGCT

AACGAGCTCAAACAATTCCCAAGCACAACAATAGCAATAGCTGCAGCTTGAAAAGCGAAGGTAGTTCTTG

GGACCTTCACATTCACAAGAGATCAATGGATAAGGTAGTTGTTATGGAGTCATGAAAGAGCCGATTGTCT

GATGTGCTCGTCAGGTAGGAAGGAGATTGCGAAGAGGTCTTCCAAGGGCGATATGTTAGTCAAATTGGAC

CTTGAAGCTTTTTCATTAGCTAAGCTGATTCAATTGAGTAAAAGAGTAAGCAAATCAACATTTGCCCCCG

GATCGTGTGCTTAGTCTTGTTAGTGACATTCAATTCCTTCTACCCTCACTAATCTCTAAGTACTCTCCAT

GGATCTCAACACTGCCCCTTAACCA

>Ca_linc_0006

GGCTGCCAGTGCATTGTATCTAACCACACTGTGGCAAATCGATACAGGTTGCTAAACAGCTTACTATACC

ATACAAAGGCATATCCGGCTGTTATAGAATCCCCTGCTATTGAATATGAGTAACTATCCAATTCCATAAT

AGAAAGAGATGGGACTAGTTGGTTTTATTCCTTAACTTAAATCAAGGATCTTAGATCTTGCCGCTGTGAT

CTTTTCTCTTGTTTCAGAAGCTGGGATTGGGCCGGCTTTCCTTTCTTAACCAGGCTCAAGGGAAGTTAGA

AGTCAAAGAAGCCTTGGTGCCTAGCGAAAGCGAGTTCTTCTTTCTTTTCTTGCTCGAAGGGTAAGCGGAT

CAGAGTCAATTCGATCTTTTCGGCACTTTCGAATGATTGTGTTTAACACCCTAACGATTCTGTGAAAGGG

GATTCATATTACTGTTGAGCTGACTAAACTCAGTTGTCGGTTAGCGGGTGCAGGTCAAACGAGGAAAGAA

AGAAGAGTTCCGGTACAACCAGACGAAATATCACTGATCATCCAGGCACAGAAACTCAACCAACTGTAGA

TGCAATCTTAATGCATTCTGGGATGAAGGAATAACCGATCTTCCCCACCTCGACTCTCACTCAACCGGTG

GAACAGCTCCCCGAAACAGCGAGAGAAGCCGGAAAAGCTGAACTCGGGGAGCCCTCAGAACCTCAACCGG

TTTCGTCAAAAGACAAAGGAAAAGCACCTATCCGGGAGGAAGAGGAAGCCGAAGATACAGAAAGAACCGT

CTCCAGCGCACCAATGGGAAACGGGGCCCCCGAAAGGATTCTGATCATAGTCGTTCAGCGCGTCAAGTTG

ACACTATAGCTACAGGCAAGTGTTTAAGGTCGTGATTTCTCATTCAATGGCCTCACAATTTTAATTCCCA

TGTACAATACGACATCGGGGATTTCACCCGCAGGCTATACTGTCAAGCAAGGAAGGCGACCTCAAAGACT

GGCGGATTACCCTCGAAACAAAGGATTTTTATATTCCTTCTGCTTTGGACAAGATAAGTGGACAGATGAG

TTGAATAATACAAGGACAGATAAGCTAGGCGAGGTTCCCCTTTGATATCTCCCAACCTAAAGGTTTATTC

AGAGGTGGGTCTTTCAACCGCCTACTCTTTCGATGAAGG

>Ca_linc_0007

ACTAGCCTAGCTTGAATATTTCTTTATAAAAGCAAAGATTTTCTACCTCCTGTAAGCTGGTTACGCTTAA

CGCCCACTTACTTAAAGACTCGTTGGTTCACGACTCTATAAATGAAAATAAGAATTGGAGTCGTTACCTA

CAGAAGCAGTTGCCCTTCTCCGGATTTACCCGGTGAGGGTGGCGCTTGTGAGTCAAAGTTGAGACTTTCA

ATGTATGTTTGGCATCCTGGGAGAGTAGTCCTAGGTACTGAAGTGACTCCCGGGTAGAGAAATAAAGTGC

TGGCAGAAAGGAACTCTCAAAGCATGGGGTTTCGCCGGGCGAAGCTCCTATCCCCCAAGGCTGTTGTCGG

CATTTCCGTTCTTTGCATGTTAGCACTTTTACCTGACCCTTCGTCACTACTTTCTGATCATGTGGATGCC

GCTCTTAGAGAATCCGCGAAAGGACACTGTGAGGGGGATGGGTCCTAGCTACTTCTTTCATACCAAGAAA

GATAAGAAGGGATGCTTGCTATCATTCCAGTGCCACTGTCCAATCTGTGAGTCCATATTATGGAATATAA

TTCCCAAGCAAAGGATTAGAAGGTAAGGTGCCAAGTTAAAGGCAAAGCTTAGTCGGATTTTTCCCCTACC

CCTAGGTTAACTACCTTTAGCTTTCGATAAGACAGTTCAAGCAGAAGACTAACTTGAGGATGTCACGTGG

AAGCTTTCCTAAATCCATTTGACCGGAGCCGGGATAAAAACTACTTATATATGTAAGGATATACCACCAA

ACCTGTGAGCCTTCCCAAATTCAACAGATTCCACTATAGCAAACTCTTCAGATCAGGAATCGCCTTTCTT

TTCTATTTTCTTTCACGCCCTGAGCCTGAGCTAACTCATTTATCTTTCGGTCTAGTCCAGTTCGGCGTAG

CTCCAAGCGAAAACATAAAAGAATTCTCTGAGTAGATCCATGTACTGTCAAATCTCTTTCATTTGAGAAG

CTTGCACTTAGGAATACAGGGCGAGGATTGTCAACAGTCCCGAGATCCATTTACTTGACCTCGTCCTCTT

TAGGGGGCGCAGGCTCTACATCCTATTCTTGAACTGACTCTCGTCTCTTTGCTCTTTGTTTTACGTTTAT

AGACAATTGGTTGGAAGAGATCTGAAAGAAAGATCTCCAGAACTTTCGGCGTCAGACAAAGTTTTCGGCG

CAGGGAGCGGAGTTGGCAGGTAGCCCAAGCCTATGTTTTTTGAGATCGACGTACTGGCCGGCTAGCCAAG

CTTCCCTTTCTTCTTCACTTAGATGCGGC

>Ca_linc_0008

TGTAATGGTTTTGTTTGGATGAAAGGTTCTCGCAAGGGCTTTAGCCTCTTGACAGCCAGGAGCCACAATG

GTCTCATTCCAATCTCCCATCTTCCGGGAAGCAAGAGCCTGTGAGACCGGCTCCGCTCAACGTCTAATCA

CCGTGTCCGCGGATCTGGGATTGGTCCCTGCTACGTCATATAGCCTACAGGTTTCCAATAGCTTGTTGAA

CTTCAGGAAAGCAGCGGAAAATAGCTAATGTTTAAGCCATTGAAAAGAGTGCCGAGTCATCAGTCCCACA

GCCTAGTCTTTCAAAGAGAGCAGGGGATTTGCCCACTACTAGAACGAGGACAGGCCTATCGGATGAAGTG

AGAGGGCATTCCATGTCCCCTGAAAAGAGGACATTCACAGCCTATTCTTTGTGCATGGGTGTGGATATCA

ACGACTATTGATTCAATTGTTCGAGGAGATAACAACAGATCAGAGGAAAGGGCTTACGACGAATGCCCTG

TTTCGGAGGGCTGGTATTTGATTAGCGGAGGACTAGTCGTCAGAGCTAGTAATGAGGATGGCATGAAGAT

CGG

>Ca_linc_0009

AAAGAAGGTCCTCTATTTTTTTAAGTAACTGCATATCCACATGCATAACAATCTATTATACTATTAGGAG

TTTTCTAAGGATCATTGCATATTATCTGAAGAAAAAAACCCTCCACTTGAATACTTTTCAATTAATTATC

ATTCTCAATGGATTTGTTTTCTTTTCTTGATGGGAATGTTTATAATGTAGTCTCACACTGAAAGTCCCAG

GTTTATAATGCATGTTCTATCTTTCTTGGTACTTACTTCAATTTCCCTAGCTTAATTTACTTTATTTCAA

CCCTTCATGCAGGAAAGGAATGAGAAGATAGAGCAATTAGAGAATGACTTTAACCTTAGTCAGAAAGTAT

TCCCAGAGTTTTTGGCTGACATTTCTTCCTATTTGAATGGGAACTTCATTGTAATTTATTTATTCCCATT

TATTTTGCAGCAAGTGGACAGATTTCGTGAG

>Ca_linc_0010

ATCATGATGTTGTTCATAGTAAATTTAATCTGTTCTACATTTGATATCTTATCCATGAACAACAGAAGGC

ATTAGAAGGCAAGCAATATTTCACAATTAAAAATCTCAAACAAAATTCATTGGACAGCTATCAACACACA

CATTATTCCGAATAAACAAGAAAGGATATATGTCACATTGAGTTTCCTTTGCTTCTATTCTCAATAATAA

ACACCGACACCAGAAGGTGTGATTATTTTTCTCAACGATGACCTTGTAAAGGTTTTTAGCTCAGAAACAA

AATCATCATCAACCAAAC

>Ca_linc_0011

TATTTTGCCTATTTAAAAATGGTAAGCATCGACATTACCTGGTGGAAGGCTTGAACTCGCATGAAAGTTG

TAAATGAAGAACTCTGGTTTTGAAAGCTGACGGAAACACCTTCAAAGCAAGCTAAAAACATTTGAAAGCA

AATACAAGCCAGATTTATTGAAATATAAAGAAAGGGGGGGATTTCATGACTTGTGCGTTTCACAAATCGC

AAGTTACGCAGTCGTGGAGATTCGATATAAAGACCAAGAATTCGATAGATAATTTGAGATCTAGGGCAAG

TAGAGCTTCGATTTGGGTGGGATCTAAGGGGTTCGTAGAAAAAATAATGGGTAGAGAAAGATGTAGATCG

TCTCCATTTCCACTAGAAATCAACATCTCCTTTTACTTCTATCTTCATCCGACACATGCCAATTAGTTAT

TTCAACGGTTCAGATTCAGAAAGGCAGAGAATATCGTTGAGTACACTAAAATGCAAAAAAAACTATTTTT

TCTTTTTTATTATTGTGGACGTTTG

>Ca_linc_0012

CGCATTTCAAAACAGAAATTGTAGCCGCCATGTGTTTGCAAAAACACAAACAGAGAGAAAATAAAAACAA

AGAAAGAAAGAAAAATAAAAAAAAAGGAGGAAGAGATGGAAAGCTTACAAAGATGAGGTCGTGAGAAGGA

GAAGAGACTAAGGAGGAGGGTGCGGCCGCTCCAAAAGGCATGGTTGTCGTTCCGGCGGCGACAATGACAA

TACGAAGTTACAACAGCTACAAATGTATAAGTGAGCAAGGCTTGTGCATCAATATCAATCAGCATCAGCA

GAGCAAGGCTTGGCAGTAAGCAAGGGCAGCAATAGAGAACAAAACAGAGGCAAAACAAGCTGGAGCAGTT

GACTTAATTTTTATTGTAATTTGATTTGGCTTTATTTTTAAAATTGTAATTTGATTTGATTTTAAAACTG

TAATTTGATTTCACATTGTAAATTGATGAGACATGATGAACATACTTATTGTAATTTGATTTGG

>Ca_linc_0013

TAGTTCGTCGAGGGCACCAAATATCTTAGAACCGGCCCTGGATGGAGAGTGCTGTTGTAGGATTATCTTG

TATCAGTAATTAGGGTACTCTACACGGAGTTACCATTTTCATTTCTTCTAGTAGAGAGATTCTATCCAAC

CATCTTATTGTAACCTCTGATGTAATCAGTATCTTAATTCTCCTATTTGTCATGGTTCCTATTATATTTG

TATGCTGAATGTTGTTTACAGTTTCAAATACAAAGTACCGGGGACTTTAAGCCATTAATTTTGCACCTTT

TTCTTATTTCTCTCTCAAATGGAAACTAAATCCTCCACAAACTTACTAGATCAATTAGTGGTGAGTGCTT

G

>Ca_linc_0014

GAGAACTTGTGCAAAAGTAATAGTTTTTTGTTGCAAAGAAAGTAAAGGGGAATTGTAACATTGTGCTCTT

CTGATGTTAGACAAAACCAAGTCACATTCTCACTAACTTGAGATGTGCAGGAGAGATGCTTACCAGAACT

GAAATGCAACATTTTCTGTTACTTTTGGATTTTGTTGTAAAAGTTTAACATATATAGAATTGACACTGAA

GAATATGTGTGATGATGGATCTGTTCTATTAAACTTAAATATGTTTTATGAACTTCAATTATTTGTACAT

GTGTCACTTGGTAATAATTTGATATCATTAAAAGCAAACTAAGTTGTGTTACTATTAACACACTTTGGAG

GTAAGGTATGTAAATGGCTGTTTCTCACAAAAGATATGGTGTATATTGTCAAATCTTT

>Ca_linc_0015

GAAAATAAGTTGAAATGAATAAGCAATTAGTCTAAAAATCATGTAGTTTTCATGATAACAAGTTATACAA

ATTTGGATTACTCTTCTTGTCCTGTATATGATAACCAAATAAGGATTGCATTAATTGTCTAATCTGAATT

ATTGATAGTGTAGCATAGTTGTCGGTCAGGAGAATCCATAAAATTCTGGTGTGATTCTTGAGAATATCAA

AGTTATGTTGGATTCATCCCTCTCCATTACAAGGCATCCTTAACTAAGAAGGAAACTCAAAATAGATAGA

TAGAGTAGACTAACATTACAAACTAGGGCTGCATTTAGATTGACTTTTTTTTTTTAATGGAATTTAGATT

GACTTATGAACAACTAATTTGGACTTATCTGTGCATAAGCACTTGTTGGGTTGTTTGGAAAATTGCTAAT

GGATGTAACTTATGACATGTTTAAGCTATTTTCAATAAACTCTCGAGGATAACTTATCCAAACAGCTTAT

AGCGCTTATAAGATAAACTTGTAATTAGCCTGTTTCTGTCAGCAGGCCCTTGTTTTCATTTCTTATTTTG

TTAAGAATTTGGACTTGTGCATTTGGCTACACCCTGAAATTTGACACTTAAATGCTGAAGACTTTTATGG

AAACCAATTCAAATTCACAAAATGAATTTGGATTTGCAAAATTTGTTGTTTAATTCGATGGCTTTTGGGC

TGGGTAGTTCAGACCAGCTGCTTTATTTATTAGTGGACCTGCTCAATTTAAGCATGCATATTATATATAT

CTTAGACTTCTTCTGACTGATAAAAAGCAGTTTGGAATCAAATAGGAATGTGCAACTTTAACAGACTTCT

TATAGTTCATTCTATTTAGGTAATTATGATTTTACATCTTTAAAAATGTAGATAATTGTAAAGTTACTTT

TGTCCTTCGCTGAGCTTTGGTTGTGTTCTGTTCTCAATCATCATTTTCAAAAGTTTATTGTGCAGACTGA

TATGTATGTTCTTGGTATTTCTTAAAACTTGTGGGCATGGCTGTTCATTTATATTGTTCTTGAAGTAGAT

GAGATCATACATTAGTTCTATGGTTTCTTTGTGATTCTAACATCTTATGTATTCACATCCATTACCTTAC

AGAGAAAGTCATGTTGGCAGCAGCAGATGTTTTGGGAGGCTGACTAGAATTGTTTGTTCGCCTGCATTCA

AGGATTCTATTCAGATTCTCTAATCATGCAGCTTTTATAAAAATGAGTATTGTAATTTGGAGTTAGAATT

GTTAACTATTTTAGGAATATAAAATGTCAAGGCTTTCTATTAGAACATTTGAAAGGTACGATTTGTGTTG

GTGTATTTTATTTCATACTTTACAATTTCGATTTCTATAGGTTTTTCCGAATAAAGAAATAAAAATGCAG

ATAGGTTAATATTTTAAATGTTCAAATGCTTGAACATAGAAGTGCATGCTTGGATGAGTTATAGAATGAC

TCGAAAGAAAAAGGTGGACGCTTTCCCAACACAATAATCTACTAGAAGGAAGACTTAGGGAGTACAAATT

TGAATAAAATACTCTACAGGAAAATGTTTCCCTTAGTGTTTTTTTAACTTGTGAGCAAACATTGGGGTGG

ATGCATAATCACCAAAAGAAAAACTGCAAAATGCATTTTGACCCAAAAGTTGTGCGACGAATTCTCCTCC

TAGGTTACATTTTTATTAAGCCACAGCAAGAACATATTTGCATTGCTTTGAGTAACCATCGACACAGTTG

TATTTCATTATATCCAAATTCCAAAGTAAATTTCTCAACAATTTGTCTTTTGATTATTTCACTCATTCCT

TT

>Ca_linc_0016

GGTTAGGTTTGGGAGGCCCGCACATGTGGGTTCTGCAAAGTAAGCAGCATCAACACATGAAGAGCCCATC

ACCCCTCCATAAATTCTTGCACCAATATACGGATGCGGCGACCGGCTCTCACCGCTCGCTTTGTTTTCCT

TACTCATTAGTTTAAGGCAACGATAATCGTAGCAATAACCTTCTTATTTATACACTACATGTTGGCTTCT

TGAAAACAATATAACCGATGTGGGACTTTAACTACATTATTACTAGCATTATAATAGCTCCATCTTCTCC

TTCTTCTTCGTCACCACCAAACGTCGGAGTCATGCTCGTTCCTCTGTTCATGTCGAACTGTGCGACTCAT

GTAGATTCATTTTCAAAACCTTTATCTGGTTCTGATGAACCCCAGCAGGGTGGTTGGCTTCGGAATTTTG

CAGTTTTTTTTCCATCCAAATTTTATCATTCTGTTTTCACTCTAATTTATTACAGGCCGTTCCAGATCCA

CCATCTGTAACCGTCAAGTTCACCGGTACAACTCTTCATAACTTCATTTCCATTTTTGATTTTGTTAATC

GGTTCATTTGAATAGATCAGTTTCCGTTGTTGGATTTTGATAGCTGGAA

>Ca_linc_0017

ATAGCTTCAAAAAATAGCCTTGTTTCTTTATGGATCATCATATATGCATTATTAGTAGTATATTATGGTG

CTAGTCATTAATTAATTACATTACAACTATGATTATCTTAATTAAGGAGTTTTATTATTATTTTTAAATA

TTTGTTTCACAGTTTTAACTTTCACGTCCAATAAAATGTGGGAACCCACGTGAAGAAAATATATCAAAGA

CTGGAATTGGAAATGAATCTATAGTACCCCCCAACACTTATGGGGAGTGATGGAGACATGTCCCATGAAA

GATTTCTCTTATTTATCTATATAGATAATTTGATACTCTCAAAAACTCTTGTATTATATTCAATATGATT

TATCAATTTCCAAACAGTTTTATTTATCTAATTCTCATTTTGTTGCCTTAGATTTGAAAGAAAATATTCA

CAATGGTCCCAATGTTGTTATTAAATGTAGCATTGCTGGCACTGCAGACACGCAAATAGTACTCTTTATT

TTTCAACTGATCTTCATTGTTTGTATTTGAGTCAATAAATTAGTGAGACAAAACCTTACTCACACACATT

GGCTAGAGATGTAGCTTACATTCAGCTTCTTCATTGTTAGAAATAATGATATAAATAAAGCTTATAAAGC

T

>Ca_linc_0018

TATGAACCAAAATTGAGTTGAGACAATTACTATGAAATTCCTCTTTAAGATCAGCCTCATCATCGAAAGA

TAGAGACCATTAACCTCTTAAGCACTGGCAATCATCCTCCCCGATTTCAACTCCTGAACTTGACAATTAA

CTAGATTAAAAATAGCCAAACAATTAAGATCTTGTGTTAATTTGCTTATGGACAACAAGTTACATCACAA

ATTTGTAACATGGAAGACATTGAGAAGAGTTAGAGTTTTAGAATTAAGGATAAAACCAACTCCAGCTATG

GTTGAAAAAGTACCATCAGCTATCTTTACCTTTTTATGACCTACACAAGTACTATAAGTGGAAAACAATT

TTGAGCAATCTGTCATATGATCAGTGGCTCTAGAATGGATGATCCATGGAGTTTTTAAATTTGGTTTGGA

ACTCAAAAGAGCATAAGAGAAATAGATACATTTTTGAGCAAGAGAGGATGAAGAAGTAACTGACATAGAG

GTAAAGAGCTTATACAAATGCTCTAGTTGTTCCTTAGAGAAAAGAGATGCCTCAGAGTTGACTTTATTCT

CCACATTTTCTCCATTTGCAACTTGGAAGGCCCTACCTTCTCCATTTGGTTTCTTC

>Ca_linc_0019

CTCATGCATAACAATTAATCTCTTATTAATATTACAACACATAGCAATATATTTATTAAGTGTTTGAAAA

TAATAAAACAAAATATTTACATTCGAATCATTAACATAGCAATATATTTTGTTGTTGTACATTAACGGGT

CTTTAAAATAAATACTATATATAATTTTTTTTGGGTACAACTATTTTTATATAATTTAACAAGTCAAAAT

ATAGATTCTCATATCTAACTCACATTTCGTTAACTTGTCGTTATTGAAAAAATCAAAAGCAAAATCAACA

CTAATCAGAGTCCCAAGTCAAAGCTGCATAATTTAGCAGAAAAATATTCGTTGATGCTCCAATTAAATAA

TTAAATTAAATGCTCTCTCATTCCTTTGATTAGAGTTCTTAATATATCCATCAGTTAGTTAGTAGTTAGT

TCGTTATTCATTCAATTGTATTCTACCTTTTTTGCTGTTTTAATAGAGGTATTTTTTTAGAAAATCATTG

TGCAGTCTTTTCTTCTTGTTCTGAACTTTTTATCAACAATTAAGGAAAAATCTCTGACAAATGGTAGTAA

AATTAATGGATCAAAATAAAATAAAAG

>Ca_linc_0020

CTTTTTTTTAGTTTAATTTATTTTTCATAAAAATATCAACAAGTTTAATCTCTACTAATTTTTTAATTTA

GGTTTTAGTCATTTATTTAGTAGATTTGAACTTTAATAAAAAATTGATCCTTTTTGTTTTATTTGACTCA

ACCTTGGAATTAAAAAGTATTGAGTGAGAAAGAAGAATGTTTCGAACATGGCGCAAAGCAATTTCTCCAC

TCTCCATGATTCGATCTCCAATTGCTTTTCACCAAACCTACGCGAAGGTTGCAGCAGCACCAATCTTGGA

AGAAAAACTCCAACCACAACCGGTATGCAATTTATCCATTATTATTACTTCAATTAAACTACACTCACAT

ACCAATTTAATTTCATATGTAAATTTTTAGGTTAACCTAGAGA

>Ca_linc_0021

CTATATTATAATAGACTATAATATATAGTTTATTATAATAGACTCATAATTTAACAGCTTCAACAAGGTA

GTAATTGGAAGTAGCAGATTAATTATTTAAGCCATGTGTTGCTGCTGCTGTGATGAGGATTGTAAATTCA

GGCCTCTTGGGTTTCTCTTGGGCCTGCCTTTTGCTTTCTTGTCTCTCATCATCTCTCTTATAGGTGTTAT

TGTTTGGATCGTGGGGTTGATTTTGACATGCATATGTCCATGTTGCCTTTGTGTGACCCTCATAGTTGAA

TTTGCTTTGGTGCTCATCAAGGCTCCAATTGTTGTTATGGAGTGGTTCATCTCCAAGATTCCATGTTAAT

TAGAGATATTTGTATTTGTAAGACTTTGTATTCACGGTGCTAATTTTAGTACTATTCTTTGGTTGATTAC

CTTTTAATTAACTTCATTCGTGCATTAGTTTTTAGTCTATAAAGAAAATTCATGCATTAGTTTAAATGTT

TAATAAAGTCCAAACGAAAATAGCAGGTGTATGGAACTACTCTATGTGTCCTATAGTAATAATTCAGTTC

TTACTTCTTTCTTGTTTTACCCTCACCCTATTCTAAAATAAGTGTTGTTGACCCCAGTTTGCTTCTCAGA

GAATAGAATAATTTGTTTCTCAAAAGGAATAGAATCATGATAGAATATCGAATTGGATAAGCATGTGATA

AAGT

>Ca_linc_0022

CCTTGTGCGAACTAGCTTTAGATTTCGTTAGTAACCAAAGCGAAGCTGAGAAGGGAAAGCGATCGACAAA

ATAAAGTAGAACGCATTCAGCTGCAAATGAAAAAAAAAAAAAGCTGATTATAAAGAGGAAGTATACAGTG

ATTTTGAAGAAAGAAAGAAAGAAGAAGAGTTTGAAAAAAACAAACCCTAACTAAGAGTGCATCGTTGTGT

GTGAAGAAGACGACGACGACACGCAAATTATGGTTGTGTTGTGTTGTGTTCTTCCTTTACTTTGC

>Ca_linc_0023

TATTATTTTTTAATAATAAATATAAATTTCCTTTTCATTTATGGAAAAAGGGTGGTTAACTAACCATGAA

TCAGGTTGGTTTAGGTTTTGAAAAGCATGTCCTTTTTCTTATTTAATTGAAAAAAAAGATATTGGGGTGT

TAGCTTTTGTTTTGTTGTTGAGACATGAATTGAGTTCATATGAGTAGATTCCTTTGAAAGGTCCTCGTGT

GTTAAACTTGTTGTAACAAATGGAAAGATGCCACGTAAGCATATCCTCGTGGCCTTGTCACTACATAAAA

TCGTATATATGAACCCTTATTAGATGAGATTGGAAATGAATGAATGAATAATTCAATACATATTCACAGT

CGTTGTTTGCAGAAGTAGCATCATCAAGATTCACATGTGAATGTGGCTAAGTGGAAATATATATATATAT

ATATATTTCTGCTAATCTATTTTATTTGAAGTGAGAATCTTGATGATGCTGCATCAGCCATAAATGACTA

TATAAATAGTACCAGAAACATTCACACTTTTTCAGACAATCCTAGGTTTATCTATTTGGGGCTTCACTGC

CTGTTTATGTTAGCTTTCTTTCTTTTCTTTATGTCCTCACTTTACAGATCTATATATCAATCAATATTCT

TTTTTTTATATATATATATATATATCCCCTTATCCAATCATAAACTATAGAGATATATGTTTTGTATTTG

GATCACTCTACTACCGTTCTGTTTTTATGGTGTGTTTGCTTGAAGATAGATGAAGGAGAGATAATATTTT

TAATTTTTTTTTATTTTGATTCAATTTTTAAGAGGGGATGAAGTCAAAATTTCTCCTATAGCTCGTTTTG

CTCTCCCTCGATTTTGGAGGAATTTGAAGGGAAGAGTTTAATATAACTAAAAAAATCATTGAAATTTTTT

TCATCTACTCTTTTGAACCAAACTTGTGTTAACTTCTCCTTTCTCCCCTCTATAGAACCAACCATGACTC

TTAAATAGAGGAATTAGTAGATAGATATTCATAATCGAAAATTATTTTTGGACACTCATTCACTCAATGT

ATACCTTGACAAAGAACTTAATAGAGAGAGTGATATGATAGTAAAACTAATATGTTAGTGATGTGATAGT

AAGAGAAAATGCAATGTTGAGAAATTTGAGATATTCAAGTATCATTATAGATACAAAATAGCATGATAGA

GCTCTTGCCAAAAGGATATATATAAGTCTATACTTAAGACTTCTTTGGATTAAATTTTTTAAGTTTCTCT

ATTCACATAAATATTTGTGGCTGTATTTACAACAATTTTTAAAACCCTCTCAGAAAATCAATTTGTGAAT

GCTAAAATCCACAAAAATTGAGGAAAACACCATCAAATTTGACAAGTTTATTTTGTAGTTAGAGATGATT

GATAGATGATATCGAGAAAAAGAGAAAATTAAGAGGGAGGCAAATATGTTAAGAAGATTTATTTGATAAA

GTTGTATCAATTATTTTTGTTTTGATTACAATTTCTTTGATGCACCTCTCTAAATCAAGGATCGTAATGG

AGTAATTGATACGTCTATAAATCACTAATGTGTAACTACTGTGAATCATGATCTGATAAGGATTATAATT

TATTATCGTGATTATTTGGTACATTGAACATAATTGAGCCATTAAATGTTTTAATTATTCCATCATGACC

CTTGACTTTTTTTATTAAATAAATAAATAGAAAATTAAACTGGAGAAAAATAAAGATAACAATTATATTT

GGTATTTGGGAACTATAGAACAATTATATTTTCTCTAATTTAGTTATATGTCACGAGGCTGGTTGAGAAA

ATTAATGTGCTTTCTTGGATATGATTCTTGGGTAAGTAAAGGACATTGTCTATAGTACAAATGATGTGCA

TATATAATATATAATATATGATTGTAATTTTAGATAGTATGTATATATACCTTAACAAGCATATAGTCTC

TAATATTGTCCTATATATACAAAGTGTATTTGGTTAAATTAATTACTCCTACACATTTTTTAATTTTTAT

TAATATTATTTGCTTTTTCAAATCTCTACATTCAGCTGTTTTATGGTGCCTAATTGTATTGGATATATAT

AATTGTTAATTACATTGAGATGCAAATTCTAAAAGCCATTGGTTTGGGAAAGGGGGTGGAATTTGGGTTC

CAATGACCACTAGTCCTTTTTTTTTTTGAACTCGTGACCACTAGATCTTGTTACTAGATACATTTAATAT

TAATTATTTGTAGTTAGAGTTAATTTGAAGTATGGACATTGTTCCAAGTGGTATATGCATATGAGGATGA

ATGAAAATTGAAGCTTGGTTGGGAATTGAATAATGCAAAATTCACGGAGTACATTGTGTCAGTTTCGTTC

ATATTTTTGTCACAAGGCTTCTTTGGCTTTAGCATGCACTGAATTCATCACTCACTCACTAGTCAAGCAA

ACTATAAGGAAGTAGTACTCTTGCAACATTGCTAAGTCATCTCAAATGCAGAGATATTTCAACTCATCAA

TTATTCAATTTTTATTTCAACTGTCTCAATTGTATTTTTTATTTGGGTAAACTATCATTTTAGTTTAAAA

AAATATAGGACGCTGTGTCATTTTAGTAGTTGACTCTGTAAAAAAATCTAAATTAATTATCAA

>Ca_linc_0024

CTATTTTTGGAATGGGATTATACTCTTATTTCTGCATAGAGGAGAACAAAAAGAAACAATTATTAAGTGA

TCTTCCTTTGCCTTCTCAGGTCAAAGACAAAGATAGCTCACCCCATTTGGTTGGAAAAAACATGGGTAAT

CAACAAGAGGAAAATCATGAGCATAAAATATTAAGCAAGGATTCTATTGTTTAAAATATGTCTTTTATTG

CATACTTTGGGGGTTCTATGGAACTTGCAATGAGTGTAAATCAAGTTTTGTTAAATTTATGTAACTTTAA

GTCAGTGCATTATATCTTTACCAAACTTAATGGATATACTTAAGTGATCAAAATACAAGAAATATAGCTT

CCATGAAATCATAATCATTTTCAGGCAATATATACCTTTAAGTAAGCACATTATTTTAGAAAGTGTTACA

GACATTCTTGTTTATAATGTTCACTCCTCAATCCTCA

>Ca_linc_0025

GCACACTTTCAGGTAGTTTCTTAATTTTCTTCTGAATTTCTACTTAAATTTTGCATTTTGTTTTCTAAAC

ATTTGCTTTGGTTTCAATGGATTGCTACTGACTTTATTTTGATTTTATAATTATTATTCAATATTTGAAG

CCTGGGTCACCTTGGATCACTTGTTCTATTTGATAACCCTATGAAGCAATTAGAGCCTAGTAGCACCATT

TCATGTCTATACCTCCATTGAAGTCTAATGATATTAAGGAAATAAGGAATTTACACAATAAAATCATCTA

ATAATTCTGTTAAATAGTTAAACAAGACCTTTTGAGAACAAAATAATTCTTTTTATCTTCTGTTTTTTCA

GTCATGGATATGGATGCAACAAGCAATGCACCCTGAATTGAATTACAATAGCAGTTTAAGGAAAGAATGG

TCCAAATAATGGTTGAAGACAGATAATACCAATAAAAAGCATGTCAATAAAGAAATGGTTTAAGGAAAGA

AAGATGAAGAGGAAAGAAGAACAGAG

>Ca_linc_0026

AAATTGCACACGACACATAATCGATAATTGCAAAAGTAACCATAGAAAAAATCAAAGTAGGTAGATTTAT

AATCCAACCAATTATAAACACATCCTCTCTAACATAAGCCAATAAATATTTGAAACCTCCTTACTCCCTC

CCTATAATAAACTTGTAAAGATAGATTCTATTTTATTTATAATTATTGTTTATGCTTTAAAGCACAACAA

AACATAAAATGAAAGAGCTTGCAACAAGGATTTCAACACCACCTCGTGATCATCCACATATGATAGATAG

ATTTCTACTCTTCCAACAAAATAATATTATTCTAAACCTTGATTTGCTCCTATGAGTAGACCAAGATATT

CAAAAAGAGTAGTTTCAATGTTACAATTGAGGACCCTCACAACCTCTTCAAACAAAGACTGTTTCACATT

GATCACCACAATAAGATTTTGTTGAAGTTAACTTTTGAAGTCCAAAGATTACCTCAAATAGTTGAAGAAC

AAATTTAATCATTCTAAAATTCGTCAATAAATACAAATTTGAATTTTTTAGATTACCTTATGAAGGACCA

ACTTCAATATCTTAACAAGTGTATTAACACCGTTTGTTAGCATTTACCATGTTTTACTTTTGTCTGGTGC

TTAATTCAGTTTCTTTTTTATAAAAGATGTAAAATTGAAATTAAATACACATTTTCATAAAAGATATTTC

TTTTTAATTAATTGAGTTAATCTTAAACTTGAATTATCTTCTAATTTTACTAGTTGGAATCTAATTAATC

TTGGAAATATGTTTTATGCCTCGATTGTGAATTTGTAATATTCTATCGCGAAATAATCTTTATAGTTACG

TGCGAAAACAACTGATTGATAAAAAAAAGTCTTTAATTTAACTTTATTTATTATATTAAACCACCGAATT

TAATCAAATATTCAATAATTTGAGCTCAATAGTTTAAGATAGTTCACATTGTTTATACTCCTAAGTCCTA

ATAAATAAATTGATCAAATCGATCAGAACTAGGGATATCCTTATAATTCGTCGTTGTAAACATGATTTGG

ACACACTTGTGTTAAATAACAAAAAAAATATTAGAGTTAATTTTTGGTGCATTGAACAAATAGCCATAAG

AGAATGTGGTACTGGTACAAATACCAAGGCTTCGCCGTCATAAAAAAAAAATTACTTATTACGTGCTTTT

ATAAAATTAAATTAATGAATGTTACAAGTGCCTTTGTAAAATAAAATAAAAAATATGCAAAAATAAAAGA

AGATATACAGTGATTTATTAAATTGAGTCTTATTCGAATCTTGACTATATTAGTCGAATTTTATTTATCT

TTGGATCAATTACTCTCAAAATTATTATTGCCTACTTCCTCTAAAAATTAACAGAACAAAACCCAAAAAT

GTTCTCTCATAATACTTGTCATGAGAAGTTGATTATTTGAAAGGTGGTTGGTTGTCGGCCATTTTGATAA

ACAATGTCAGAG

>Ca_linc_0027

GTAATTTTTATTTTATTTTATATTTTATATTTAACGTGTACGGAAATCAAACTATTAACAAATAATCTCG

AATGTATTAAAATGTATGAATGTGTTGGTGATTGCAAAATTGCATTGTAGATAAAGTTCAATGCAACAAT

CAACACAATCGTGTCCATTGTCCCAACCTGCTTTTCAATTTGCATGCACATGCACATTCACATTCACATT

CACGGGTCTAGATTCTAAATCGCTCTCACATTATTCTTGTCACGAGGTACAATATAAATGTTCTTTCTAC

ACAAGTCAGTATTACATTCACACATCCTAAATGAGAGAAATTCCAAAAATTCTAAGGTGATTTACGGTAG

GTTTAGATAAAAAAATCATTTGAAATTTTAATGATAGGCAAATTATTTGATTGGCG

>Ca_linc_0028

GCAAAACCTAACTTAAATCTAAACACTACTTGATCTTATCTGAGCAGAGAGTAGCCGCCGCCACGGTTTT

CACCTTTCCGAGTCTATCATTTTCTATCTCACTCTCGTCGGATACGCTGACGGTGGAGCCTCCGTTTACG

GCGGACTCTGCCCTCGTACCCGCACTGACCGTGAGATGGAGATTGTATCTATCGGGTTTAAAGGTTTGAC

TGGTTTGCCGTCGGTAACCTGCGATGAAGACTGCCCGCGGTCAGAGCCCCTTTCCTATTGGTTGGGGCTG

TGAAGGCCACGCGGGCTGCCCAAATATCATCAACTTTTGCTTTTGTTTTTTTTTTCTTTTATTAAATGAA

TGTAAAGATTTTCGCATGTGCTTATTAATTTTGAACTTTATTTTACATTTGTTTTATTTGTTTTTGCTAT

TTGGCAATTTATTTTGTGTTGCTGAATTCAACTAATTCATTTGGACTTGTTAGGCCCTCTTTTTAAGGTT

CATATGCTGCTTGGTGTGCAGCAGTACTTTGAGAGTGATCCTTGGAACTTAGGGCTGAGTTCTCGAGTGT

TTTTAGGTTCCCAAATTCATTTTCATGTTTTCGTTAATTTGTTATTCCTTGTACATATAAATATAATAAA

TTTTCACGTCAATTGAATATACTATTTATCTGTTATGTATGATCCAATTGCTATGTTCCAGTGGATATTC

TACAAGTTAGTCAACTCACGAAATGCAAGAATGCAACTTGTATCATTTTGATTGACTTCAATATATTTTC

AACTTACCCTATGATTGATTATGTGGTTCCTTACATAGTGATGCATGTTTCGTTTTATTTCGTCGGCAAT

GGTACTGTACCAAGTAGCAAC

>Ca_linc_0029

TTTATCTTCAACCTCTTATTTTCCTTATTTTCCTTCATTTTTCAAATCAGTTAAACCAAAAGCTTTTAGG

ACCATTTTTAAACATGCATGTCTCATCTGTTCATAACCAGATTCAACCAAGGAAACAACCAGGGATAATA

ACCCAAAAATGAAGAACTTGAGTTTCAGGAAAAAAGGGAGCTCAACAATCAATCTAATTAGAACGTATTC

TCCCTTATCACAAACTCAGCTGGAGGAACATGAGACATGAAAATTAGCATCAGAAGATCAGTTCCAAACT

CCTGGACTTAGAATTTGATTTTTTGTTTTGTTTTATGATGTAATCAGACAATCGTAGTTTGTTTTCAAGA

TGTAATATTTTATGAATCTACTAAACATGTTGTAAACTATATTTCTGAATGTAGTTTTCAGTTATGATGG

TG

>Ca_linc_0030

CTCACACTTTCAGTTATCTTCTTCCTCCATCTCCTCACCTCACCTCACATAGCAGCGTGAGTGCCACTGT

CTACCTTCACTCTCCACTCTAACCCTTCCTCCATTCTTCTTCTAAGAGAATATCAATATGCATTGTTTCT

GTTTGGCAGAAATGATAATAGCTACAATGGCTTTATTCTCAAACAAATGAATCAATCATTTTTCACTTTT

TCAACAGTTTCC

>Ca_linc_0031

CTCATTATTTGTTATTATAGTTACTTTTAGGTACAAGTAAGGTATTACTTATTCTCATCTAATATCAACA

CATCATGTTAATTTAAAAAAAAACTCACATATTCACCTATTCATCCAAAAAAAATATTCACTTATTCACC

CAAACATCAATTCACCCAACAATACATTTTCCCTTTCTTCCCCATTTCCATCTTAGAATTTTTTTTTTCT

ATTTCTTCTCAAATTTGCTCAAAAGGTCGAGGAATTATTAAAGTTCTTCTAAAAAAATTCCTATTTCATC

TTAGAAATTATTGTCCCAATTTCTTCATTGATTCTTACTTTTCCTTTGAAGAAGTTTTTCTGGATTTCTT

CATCATTCTTTAACCTTGAAGAAGTTTTTCCCGTTTTGTTAAAAAAATTCAATTCTCTCTTCAATTGATT

CCTTCAATTTCCTCTCCAATCGTTTTCCTTTAGTTTCCTCTTCAATATGTATTCATCTTCAATCTTTTTC

CTTCAATCAGATTTCTCTTACGATTTAGTGATTTACGTTTTACGATTTTTGATTCACGACTAAGCAGTTC

AAGGATTCATGAGCGATGGAGATTTCAATTCGAGGTTAAATTCACGTTAAGAGGGAGGGGGTGGTGAAGA

CATAATAACCAAGGATACTAGTGAAGCACTTCAAAGTCTAAGAGGACCATGATAAGGGCACATGCCAAAA

AATCTAAGGATGTCTTAAACCAAATAGTGACTGTTATCAAGGAAACAAGACATAACTTAGAAGAAATGGA

GGCAAAATTGGTCAACTACATAAATCAACATGGAGAGGACTTGGGCACATGAGATGCTGCCTATTTGCTT

TGTTTTATTTGGTTTAAGTTGTAATA

>Ca_linc_0032

CTTGAATGCGCAACTCATAGTAATAAATAAAAAACTAAGCTAAACCTAAAATAAATGGAGAGGAAAAGTC

CAACTAAGAAAAAAAGGAAAAAAAAAAACAAAGAGAATGTTAGAGAGAAAGAGTACAAACGTGAGAGAGA

GAGAGTGATGACAGAGGACTTCGTGTACAGTGGTGGCCTGCACCAGAGTGGTTCATAGAGGGGGGTCGTG

GTGGTCGGTTAACAAAGGTGCGGACGCTCGATCGAAGATGGAAGTTGTGGTGGCATAAGGTCTGGAAACA

GAAGCCGCGATCCTATCCCGACGACAGTAAAGTTCCTCCCTCTTCTTCTTTTTCTATAGTTTCTTTGATT

TTTGTTGAATATTCTGATTTTAAGAGTTTTTCTGATGAGATACAAAAAACGGATTTATTTTTATGTTTTA

TTTTTCTAAGTTTTTGTTTTCATGGTGGAATGTATTTGTTGATGTATTTAAAGAAATAAAACCTATTGAG

TTTCTAATTTTTTGTTCAGAAAAATCCCCTTCTTATGTTCTTTTTTGTTTTCTTTGTAACTCCCTTTTTC

TTCTCATTTAATTAATTTTTTTGTCTCAATTTATAGACTAAAGTGGTGAATCTTCGTGTTCATTAGATAA

AATTATGTTATTGATTTTTTTATTGTTTTGCAGCAAAAACAAGCAACCTCAAATAATTAAGGAAATAAAT

GACTTATCTTTTTTATTTTGATTTAGTGTTCAAATAGTGAAGATATAACCTTATTAGGATTAAAAAAAAA

AACATATTAAAATGGGAAATGGAGTTGCTTGCAGACAAAAATGGAGGAAGGTGTAAGGAAAGTGCGGAAG

GAGGAAAGGGCACTGGTGTACTTCTATTTGTTGTTTTTTTTTTCCGCTAAATATAATGTAATTGAAATCC

ATATAAATACATACTCAAAATTTGGATTATTTATAAAACGAAA

>Ca_linc_0033

AGGAGATCGAGGAGGAGGGTGAGGCTGCACCGAAAAGCCTGGTTGCCGTTTCGGTAGCGACGACAACAAC

ATCATCACAGGTAGAGTCACCTATTCAATGTGAGGCAAAATTAATTCATGATGCAACCAATATTGGGTCA

AGTTACAAAAATGTAAAAATTTGTGAATTGCAAGATAAATCTCAACATGCGATAGAGATTTTAAGCTCAA

AATAAAATATGCAGGAAATGTTATGCACAACTTGTACAATTTTGTACTATCTAGAACAACCAATATGAAT

TTTGAAATTGATACTTGTCTTTTTCTCCTTTTCTGTTTAACTATGTAGGAGATTGATATTTTCATCATAG

TGTTGATTGGAACATCAGGTAATCTTGATAATACACTTAAATATGTAGTAGATTGTTCAGATGATGCACT

TGGAATGATGATCGATGAGAAAACATT

>Ca_linc_0034

GAACACAAAATGATTGGTTTGTTGTTGTGCATGCTAAAGTTAGAGATGTGTATGATTTGGGTGATGAGTT

GTCCAATGATGTAGATCAAGTGAATGAACAAGTCTTGCAAGATATAAATGACAACGACTTGATTAGACCA

GAAGTTGATGATAATCATGATATCATGGAAGTTACAACATCCATGGAGAGCATTCTCCAAAATGATGAGG

GTGGTGATGATACAGAGGATGAGAGTGAGGGAGACTTCTTCTAATATATAGAGCAAATTATTTATCAATC

AATTTATTTGACTTCTAATTTATTGAAGTTACAAAGAAAGTACCATGGGGAAGCGCATAAGGAAGTTGAA

TACTACTCAAAATTGTGGACAATCACAAGAGGAAGGGCACTCAATTGATGGCTATAATGAACTCAACCAA

AATAACCATTC

>Ca_linc_0035

CTCAAAGAGTCTTCCTCCTAATTGGTAGAGGCAATATAACAGAAATTGCAGACTTAGAATTACAAGATCT

AAAAGAACCAGTTCTCATAAACAACAAAAAAGAGTTAACAATTTTCTTAACCTTAACAGCAACCGTTTTA

ATCTTAGGCTTTTTGGGTTGTACAAAATTATCCAAAGATGATAATTTGTAAGTTGTGAGAAAGATTTGTC

TTTGTTTTGCTCTTCTTTGTCTGTAGCTAATAGACTTTAGCATGTTGTTGTCTTTCCCTTGTAAACTAAG

AAGTTCATAGTTAAGAAGCTCTTCTTTGATTGTATTTTTTTTCTCTCAATTTTTAGCACATACATAAGGA

TTCTGAGAAGATTATGGCCCCTTATTTTAGGATCTAAATAGGATTCATTTAGCAGATGTATTATCATAGG

AAATCTCAATAACAATGTCATGAAAATAAATTGAATCTCAACAACAAAAGAGTAGAACTCAAAAATAGAA

TGAAGAAGAATACCTCGACAAAATAAAACAGAGATGGTGGCGAGCCGTGACGGTGGCGAACACAAGAAGG

CCGAGTCGTGACCGTTACGGTGGTCGGAGAATGGAGGAGAAATTGGACTGAACGGATGAGAGATTAAGTT

CTGAATTCGTCATAGATCACTCTCACTCTGAAAATGGCAAGTGTCGGCGCTGGGATGTGTGACGGTGGCG

GTGACGACGACGATGATGGTGGCGAGCTTTGACCGATGGCTACGGTGAGAAGAAGTCTCGCAATTGTTTT

TTGTTTTTTTTATTTATTTAATTTCTTTCTTTTTATTTTCTAATTTATTGTTTTATTGGGTGACTGCATT

AAATTGCCACATAAATAAGAGCATCTCCAACGGTGAACTCAATATGGACTAGAGTCTACTGTGCCACAGC

AATTCACTCATTTTCTACTCGAATAGTGAACTCAACATAATTCAAGTTCTAGTAGAACCCAAAATTGTGA

AGATGATAGAAACTGTACAATCACTCATTGACAAATCTGAAGACGCTGCATCGATAATTACTCTATCAAA

TATATTCAGCGATGATCTTGCTATGATCATTAATTATTGCAAGAAATTATTATACTACGAACAAGAAGAG

ACCAATGATGCAAAAGACAAGTTTGATGTTGAATTTGAGAATCAACTGAGCGAAGATGACGAAATTGAGC

GTAGATGCAAATTACCTAGGCATAACTAGCCTTCTTGAATCAAATGAAGAAGAGGATCGTCGACTTGAAA

ATACTCTTGATGAAGAAGAAGTCGAACAGAGTCCAAAATGCTATTGAATGTTTCAAAATATTTTGTTTAG

TTGAGTAGCCTATGAATTAGAATTAAGAATTCTTCAATTTGTACCAAAAATTAGAAATTTAATATTTTAT

ATCGTCTCTGTATTTCTTGTTTTTCTTCTCTACTTTTCA

>Ca_linc_0036

CAAAAGGATAGAAACAAAACCCTTCTTCCACCAACTTGCAGCCACCGAGAAACAAAACCCTTCCCTTCAG

CGCGCATTCAATCAACGGTATTGCCGCCACCGAGCTCTCCGTTTGCCGTTGACCACCACTTCAAATCACA

GCTCATTGAAATCCCTAACATCTCATTTCAATGGTAAAAGCTTGAAAATCCCTAACTCTGTTTGTTTAAT

TCCTAAATCTGTTGTGAGGTGTGATATTGATTTTTTTTTTTTTTTGCTTGGAAAATGATATTGATATTGA

GGTGGGGGTACCATTTTTTGTGTGCTTAGATTCTGATTTTTGTTTGCTTGGATAGTGATACTGGTATTGA

GGTGGGGGTACCATTTTTTGTGTGCTTGGATTCTGATTTTTGTTTGCTTGGATAGTGATACTGATATTGA

GGTGGGGGTACCATTTTTTTTGTGCTTGGATTCTGATTTTTGTTTGCTTGGATACTGATATTAATTCCTA

ACTCCTAAGTCTGTTGTGAGGTGTTATGCTGACATTGAGGTGTTCTTATATATTAATACATGTTTATATT

GGTTGATACAAGTTTATATTTTTTTGGGAATTGATTGGAATTGGTTTAAATGTGTTCTTATTTGTTGCAA

AAGGAATGTTATCAGTGTGATTTAAATGCTTTTTACTCTTGCTAATTGGGTTTATTGCTCTTCTTCCATT

CTTTAATTGAACAGTATTTCGCTAGGTTTAACATTAAGAAGAACAAAAATGATTGAAATTAAATTATTAC

ATGTAATGTGGGTGACTAGTTAACCATGAAGGAATAAAGGGTAGGTTTAATTTCTACTGTAGAAACCATG

AAGGAATAAATGGTAGGTTTGTATAGCATTTTTTCCAGCACAATGATCACATAGATATGCAAGAAACAGA

AGCCTGTGTAAAAGTGAAATACCTTAAATGACATAAACATTTGCATATTTTCAGTGCCTGTCCTTGTGGA

ATTTTAGTTAAAAGCTTAAGATAATGTTAGTGGAACACATTAAGGTTTTCTTGTGGGAGTAGTTTTTGAC

TATTTTTGAAGGTGGTTTTGTTTGTTAAATGCATTTGTACTAAGGTTAGAATATCTTTTATACAGTTGTA

CTTAGTGGAGATATTAACATTCATTTAAATTAAAATTGCATTTAAATAAGAAAACTGCACAAAATCAATT

TTACCAATTTTATTTGTTTGTATTTTATCAAT

>Ca_linc_0037

CATATATGTGTAAAGAGGTATAAAAATTATTTAAAGAATTTTTAGTTCCTCCTCTTATTAGTCTTATCCC

CACACACCAATCTACAACTGAATAAGCCAACACTTTATTCTCACAATGCTATGACTTGTAGTGGAATAAT

ATCCCATTCTTTAATGCCCCTTTAATGTCCCTTTCAAATGAGATCTCCTTGCATCATCCATTAATCTCTT

TGTTAAGCCTTCTTTAACGCCTTTTATTGTCTTGTTGGTAATTGTAACTTTTTGAAGCCTTTGAAAGTTT

TGTAAGAACCATTTCATTAAGAGGGGATCATAAAGAAATTAGTAGAAGGGTTAACACATCAACTAGGATT

AATATTAGTACTCATGGCCAATTTTTTTTCTTGTAACCATCAGCAAAATGGCTTGCCTTTGTCCTATATA

TACATATATGCCACTCATGAATAAGAACGGAATGAATTTTTTTAATTTAACACCTTGTATATTTTGGTTT

GAGAGTGTGCCTTTCTTTGGTTTTTTATTTAACCAATTCTTTGATCATATCATGAAAACAAATTTTCGTG

TGGTGATCATTCACTAGGCTTATCAATTTTTGTTAGAATTTGAGACTTTTCTAACACACATTTAAGTTAT

ATTATGTTTATTTGTTTTCTGTTGTAGTATTCATGGTTAGGTGAAAGACTCATATATTTTAGAGTGTTGG

ATGTGTGGTAAGTGTTGGAACCAAGTTGGTTTTTCTCTTGTGATTTTGAAGTTAACAAATGTATTTTAGG

AGAACATTTTGTTTGGACTAATGACTTCATTAAGTGTGCAGCAATTAGAACAAGAAAATAAGCGGAAGGT

ACCAGAGAATGCCAACAAAGGAAGCTACCAAAGAAAGTGTTCAAGCATGAGATTTGAGATGTTCACCAAA

AATATTTTATGATCTTTTGTTTTATCTTGAGTGTTATTTTGTTGTTCAAGTTACCTCTACATGAAGTATG

GTGACCTATACTTGTTGGAGTGGTTTA

>Ca_linc_0038

TTCTTTAAACTACACAGGAAATGCCCTAGTTGCTCCACAAGCCCTACTGTCAATTGAAAAGGTTGTCCCA

AACATAAGGCAGCTTATTCTGAACGGCAGGGATGTCAGTATGTTATGCAATGGTCAACTTAATCACACAC

CTATCTACACAGTAAAAGCTCTTCGCCTGCGATGTTTTCACGATGAGTCCGACAAATTCCATCAGGTTTC

CTCCATAGATTTATTAATCTTGAAAATCTCATGGTGACTTGCAGTTCCTTCACAGAGATTGTCTCAGGTG

GCAGCTTTGACACTGGTCATTCTGAAACAACAATGAAGTTGAGAAGTCTAGTACTTGTTGAATTACATAA

CCTCGAGTTTATATGTGTAGCCCATTCTGAGATGCAGTTCTTTGTTCAAAATCTTAGCGTATTTAAATGT

CCCAGACTGAGGAATATAGTTTCATCCTTGGTGAGGTTTGAGAATTGAGAGCATTTGAGGGTGGCCTATT

GTGTTGGATTGGAGAATATTATGTCATCATCAACTGCTACTAATTTGCCAAAACTTCGGGAATTATTTAT

AGATAATTGTGAGAAGAT

>Ca_linc_0039

TTTGCAACTCACATGGCGAGGGGAACGCCATTTTCGCAAGGGAGGGGAAAGTCATTTTCAACAAACATTT

AAAGGCATGGTGCACACTATGTTTACTAACCACGAGGGTCGTGTAAGAGTTAATACATCTTATGACCACG

AGGCTCGTGTTAGACCAACCACTTCTATGCGGCTCATTGACGATGCAACAATCATCCAACGTCTTTGAAG

CATCCATACCATCCAGATGATATGGATCAACCTCTACAACCAACTGTAGAGGAGCCCTAACAAAAACACG

AGGGTTTAATAGTTTATGGATTCCTTGATTATCCTTCTGACACAATCATTAGATTATTTGGTGACCATGT

AACTTCTCCACTTTGGGATAGAGTGACAATTTTGTAGTTATATTAATTTTTCATAGTTGTTGTCATAATG

TCATTTATCCTATATTTTTTTAATTTATTTAAGGATCGTGGGAAGTTGGGGGTATTTAGTAATGAAAAGA

TGTCATTTTGCAGAGGTGTAACAATTGGTGGAAAGTTCGTGACTATT

>Ca_linc_0040

CAAAGTTGATTTTCTTTCTTCAACTGCAACAACGCCTTCATTCCCATTTTGATCTTGAAATTATTTTTCC

CTATTTCTTCTCTTCTCAGTTTGTTTTCCTATTTCATCTCAGAAATTGTCAAAGTTGATTTTCATTTTTC

CGTTGAAGAAGCTTTTCCCAATTTCTTCATCATTTTTTGGATTCCGTCTTCAATCAATTCCTTCAATTTC

CTCTTGAATCGATTTCCTCTTTAATATGTATTCATCTTCAATCGGATTTCTCTTACGATTCACCGATTTA

TGATTTACTATTCAGACGCAGCAATTCAGATTCAACAATTTAGGGATTCACGATTGCAAATTGGATATGT

TGAGCAAACTCTATTACCTGCATTAGGCCAAGCTGCAGTGTACACTGAAGAACGTCCGTCCAACAACTTC

CCCACTGCAGCTGGACTAGCTTCATCAACTACTGGTTTTGCTTATCTTGATCAAGGGAATGAACAAGTCT

TGCAAGATATAAATGACAATGACTTGATT

>Ca_linc_0041

GTTTCATTTCCTGAAAATATCTATCTTTTTTTTCTACAACACTGTTTTGATGAGGGTGTTCTATGAGAGG

AAAAGTTATGTAAAGTTCCATTATATTCACAAAAAATTTCAAGGAGCTTATTTTTAAATTCATTCTCATG

ATTACTATGAATTGTTGTAATAAAAACCTTTTTTATTTTTCACATTTTTGCAGAAGGTAGTGAAAACCTC

ACGTGACTCATCATTTGTGTTTAAGGAACTTTACCCATGTCCATATGGAATATTCATTAACAATAACAAG

TCCATAATTTTTTTACCACTAATAGAGGTATTCTATACATGGTCAAATAAATCAAAATGTAAAAGTTCAA

GCGGTCTAGAAGTGGAAACAACATTTTTTGAGGAAAAAATAAGATATTGTTTGTTTCCCTTTCTAACATG

CCTCACAAAGAGCATATGATTCATATTTTAGATTTGGCAAGCCTCTGACTTGATCAAGTCTTTTCAACTT

TGAGATTAACCTCAAATTGGCATGTCCAAAACTTTTGTGTCAAATCAGTTGTTATTCATTCACAAACATA

ATACATTTTACTTCTTGTTTTTCCAAATCTGATAAGTTTATTTTGTAAATATTGTTTTTCCTTTGACCAG

TAAATAGTATAGATCCATCTATTTGAGTTATTGCCTTACATGAATTTTGATTAAAGACAACACCACTACC

ATTGTCGCTTAATTGATTTGTTTTCAATAGGTTATTCAATAATCCTTATACAAGTAAAACACTTTTAATA

GAGGGAAGTGAACCATTACCTACTGTGATATGCCCAATGATCCTACTCTTCTAATTACCTTCAAATCCTA

CAAATTCTTCGTTCTTGAGGGTCAGGGAACATAGACTTTTCTCTCGTCACGTGTCATCAGCATCCATTGT

TCAAATACCGCGACTGGTGTTTTAACATTGTTGCATAGGATATCTGTAAGATGTAATATTTTATTCTTAG

GTACCCAAATTTGGTTGGGTCCTCTTTTGTTAGTTATTAAAGGTGTCTTGTCTTTCCATGTTTTTGCATA

GGACAAATTCTTTTATTGTGACATTTCCTTAAGCAATATCAAGTAAGTTAACTTTAGCAGTTTTCTTTGT

TTCTTCAAAACCAATACCACATTTATTGTTTCTGCTAACACCATAGATCATGGAGACTTGTTTACTTCTA

TCTATGTCATTTTCAAGAAATTATTAAAAGATTTTTTTCATACTTAACAGAATTACAGACTTTATCCTTT

AAGAGTTTTGAAACATTTTCTTTCAAAGTTGAATTTTCTCCTTTTTGGTTGTCAAAATCATTTTCAAGAA

ATTTGTATTTATTTGTTAAGATGACATGAGCTTTTCTAAGAGTTTTTAGATTTGAAGAACAATATCATTC

TTGTTTAGAAACTTATTTAACATAACGACAAGTTCATTACGAGATAGGTCGTATAGTACCTCAATTTCAT

AGTGTGTATCTATCATCAGAGCCATGTTTGCATGCTCTTCATTTTCATCAGATGATTCATTAGAGTTATC

TTAAGTAGCCATAATACCTTTTTTCTATGCCTTGGAATATTTTCTTAGGGAACTTCCCTTCTTTTCTTAT

CTGATTTTTCACCTTTTTCTCTATTATTTTGGGATCGTTTGCAATCTCTTTTTGGAAATACAACTCTTTT

GTTCCACATGCATTGAATTTTCTTTAAGATGAAATACAACTCATCATCATATGCATCTTCACTTTGATCG

TCTGATTTGTTTTCCTATAAGCCTTCTTTGAGATGAAGGAGGTCTATTAACAATAAGATGTTTATAATGT

TGTACTCTTAGAAAATATATCTCAGCACTTAGACAAAACAAAAAATTAATAGCAAGAGTGTAGAGACGAT

TGTATTGTGCTTATTGTGGTAGGTTGTTGTTGTATTCTTCATTGAGAGTTGCAGATCCTTTTATAGAGAT

CTTTAGGGTTTCTGGCTCCTGTCCTGATTGTAAGAAATCAGTTGTATCATAAATTTGTGTATCAAATCTT

CAGAGATGTATTCACCATTAAAGCATGTCTAAAATTAATCCTTGTAATTGATTATGTGCTTCTTGATACA

GAGACAGAAAAAGCGGACAATATCATGATCATAGTATGTCACATAGTCCGTCAAACAAATTACGAATTCA

GAGTGTTGAGCAGATGATGCACAAATTTGTCTTCTTCAGATTGTTCACTTTTTATAGCGTTGACTTTGTT

CAGGGATTTGACTTTTAGAGCATTTACTTCTATCAAAGCATTGACTGTCTAAAGTATTGACATTTTATCT

GAGTGTTGACTTTTAAATAGAGTTAGATGATCAGTTGTTTGACGTGATTATTAGAGACAAACAATCATTG

GCTTGTATGAGGAAGAATAATCATATGTTTGTTGCAGTCATCAGAGTCAAAAAAATCAGAGCATTGCTTG

TACTAGCATGATCACCAGAGGTGTGTTTCCAAGTGCGTTCATCAGACTCAAAATACGGATTTCAGAGCGA

AGTCATGGTGTTCCCTTAACTTACATCAAAACATCAAAGGATGCAGCGTAATGTCATATAAAATAATCTC

ACCTGAGAACTACACACTTAAACAAATAGTTAGTGCTTGAAATTGTTCCCACAAAATGATTTGTTTTCAT

CAAAACAAATAAAAGTCATAATTCTTCAAATTAGCTTAATTCTAACAATATTATCCAAACTAAACCGGTG

AATTATAGGTTTGTTTGAATACCTTCAATGTGTTTGATACTTATAAAAATGTATAACTTATTTTAGGAAA

GAGATTTTTTTTTGCATATTATAGTTTTTTGTATTCACCAAATTTTGATCGATGAGATTAAG

>Ca_linc_0042

ACTTAATTGTAGTTTTATTTTTGCAAAAACGAAAAATACATTATTTATATTGACATGAAACAAGCTCGCT

TGCATGAGGAACGCAAAGTTAAAACAAAGAAGGATTATGCATTGCTCTGACAAGCAAACAACCATTAGAT

CACCTTACCCCAAATAAAAAGCAGATACAAAAATAACTCTTAAATCTCATAAGATACAGCAAACTCCTAA

AGGTAAACACCGTAGCGGCATTTTGTGTGATCAAAATATGCAAAATACAGAGTTGAACAACCATTCCAAA

ACCCAGCAAAACTCCAGAACAGTTACAGCCTATTCCACCATTAAACACCTCATCCTCCGCTGCTCGGCCG

CTCCAGCATAACGCCAGCCCACACCAAACTTGATTACACCGCCCAACAGCGCTTGAAATGCAGATATGTG

GCTCTGTTCCTACAACCCGCACTGCCAAAACACCAACAAATTCATCTAAGATAGAAATGTGCACATTGCA

GTCGCATAAAACAGCCTCCAGACTGATGATACTGCCAACAATTTAAGCCACACCAACACAGCAGCAAAAA

ATTTATAATCAGCCTGCTAATCAATGGGGTGGCAATTCACTTGTGTTAGGAATAAAATCTTATTGAACTA

CTATAGTTAATAACTTACATGGATGTTGTTTTCATTAATTTCAACTATTGAAGAGTTTTCATTTCTTACA

AGCTAAAAACTGGACAATCTTTTGTATTTTATGACTTATGTGCTCAAAGTTTATACTTTTTTTCTATATG

GAGGATGCCTTCTCGCTTTAATTATATTTCTCTCTATTTTCTTCTTCG

>Ca_linc_0043

CTCACATTTCTTTCTCCTTCTCTATTCTCTTTTTCATCATCTTTTCTTTCTTTCCTATGTTTCTTACATG

AGAATGAGAATCATAAATTCATGAATGGATCGGTAGATTGTGGTTGATGGGAATGTTGTTTGGCTCGAGG

TAACTATAACTCAATTTCTTCGTCTTTGAACTAAACATAGTTTCAAAGGTTTGATTTTGAATGATATATA

TCTATTTATCTATTTATCTCGGACCAGGCTTCATTCCCGTCAACTTGATCTTCTTTTATCTCTTTAAATC

TAACGGTTCTTACAACTGCTGCACCATATTCTGGTAAGATATTCTAGTTTGTGTTGTGTGTGGATTCATT

TAATTTAGAGATTGCATGAATATTTGTCAAATAGTATTCATATGTAGGGATGCAAAGAGATGAGGAAGAT

GCCTCTGACTTTGTTGTTGGTTGGTTGGTTGCTGCACCTTTGCCACTGCTTTTCTTAATTTGTCTTCTTT

CTTGCGGCTTCATATATTTATCTCTTTCAACATGTGATTTTAACTGTTTAAAGGAGGCTCAGAAATCTAG

ATTATTCATAATTATTTTTCTCCGGTTATGATTAGTGAGTGATTGTTATTAACAGTATTATAGCTTTTTT

CTGATTGAAAAGTTAAAGCAGTAAATACTCTGTATCTTATTAAAATATAATGAGTCAACAAAATAAATAT

ATTTATCTAAATTTTATATTATATTTGATTAATTAAACTTTTTCTTATATTAAAGGCGGCATAAGAGAGG

AGTGTATTAAGAAACACATGAAGCTTTAAAATAAATTCTGCATTTTGTCAGTAGATGTTTCAGAATTATG

TAGCATCTTTAGGGTTTCAGATTCCTAGCTTGCCTTTTCTTGCTATCAAATTTCATGCTATCAAAATACT

TTGCCTTGCTTTTTCAGGTAACATATATATTTATATATTTTTGTTTTTTGTTTTGACAGAAATTAGTTAT

TTCTTTTGATCTTAGGAACAAGGTGGCGGCTGCAAATTGCAGTTCTTTTTTGCTTTCTTCTTGTCTCTTT

TTCTTTATTTGTCTCAGAGATTGATATTGATCACTCTGAGTAAAGTGTAGTATATGGATCTAATCTCTAA

AACTATAGCACATTTTATATTTTTCTATCAATTCAATTTTTATTTATACTAGAGTATTCACCACTATATA

TTTTGTCTTTGTTTGGTTGCAATGGCATTACAATTCTAAGTCCTAAAACATGACCAAAACAAACGTACTA

TTAG

>Ca_linc_0044

TCCTCACTCATCTAAACTCTAATCGATCTATTTATAGTCTTGCCTCCTCTCATACTTCTACACCTCACCT

TCTTTCTAATTAGGGTTTCAAAATCTAACCTCTTTTTCTTGCCGCCGCACCCTTTTCCATTTGTAGCAAA

CTACTTTCACCTTCATTCTTCAGATCCAATGTAGCCAATCGTTGACCTAACCTCTGTTTTGCAGCTCAAT

TACGAGGTAAATAACAATACCAGATCAGACTTTATCATCCGTTATGGTTTCGTGTTCACATAATTTTAAT

CTTTCGTGATAGTTAGTAATAATTATGGTTGATTTTTTCAATCAATTATATTTTATGCTTTTGAATTGGA

GTCCAAATTGCATGCTTTAGCAGCTATACTGTTTGTATCTTCATGCATATTAAGTTCTTGAAGAAAATAT

CGATTCTCCATACATTTTTTATCCCAACGAAAAACAGATATATGGTTATATGGATTTATGTTGTTAATTG

AACTTCTATGTTTTATTTTGTTTCTATAGACTGATATTTTTTTATATTATTGACTTGTCTCTAACATGTG

TTTGATGGTTTCAGTGTGCAGTGGTTGAAGATCTGAAGATGACGTGTGTGTTGTCTATGATTTGTTTGAC

CGAATTCTTCATATTTGTTACGGCTGTGATTTCTTTGATTAAATATCATACAAAGTCTATGTCTTAACGG

TGACATCGATTAATATAATTCTTAATTGAGTTAAATCGGTGACATCTGTATATAGGCCGAACTTACAAAG

TCTATGTCTTAACGGTGACATCGATTAATATAATTCTTAATTGAGTTAAATCGGTGACATCTGTATATAG

ACCGAACTTACAAAGTCTATGTCTTAACAATAGAGAAAAAGTGATATCTGAATCACGTTCAACAAAAGTT

AATAGAATGCAAGAACAAAAAAAGCAAAAAAAGTTATGTATCCAATATAATTTGTAATTTTTTTTTTTGA

ATAATGTTATTTTTCATATTGCAGTTTTAATGAGTTTCTGAATTAGCAACTCGTATCAATTAACATTTAT

TGTATTTATTCTGCCCTAAATAGAGGAATGA

>Ca_linc_0045

TTTTAAATCTCAAAATTTAATGTGCCCATTTCCTTTTTGTTGTGTGGTTGTTGGCAAAATTAAATGTCAC

CATATTTAAGACTCTGTATTTTTGTCTCTGTAATTGGTCCCTCCCCTCTCTCACACATTAATTTTAAAGA

ATCTCACAGGACAACATAGTTACTCCAAAACAAAACATGCCACCTTCTTTCTTCTTCATCCTTGTTTTTT

TTCTTCATAGGGTCAAGTTAAACCTTTCTTTGTTCTTCTTTTTGTGTATAAATTCTGGTGATGGATATTG

AAGTTACTTATGGTAGTAACAAGCTAGTAATATTTTCTTGTCACATAGATATATTAATATTTTATTATAT

GTTGTTATTTTGTTTATTCATTAGTGCAGGAACAGGTGACATTCTAAGAAGTTTTCGTATTCCATTTCTT

CATCTTAGATAAAAAAATGTAGTGCAAGAAATTTGATATCACCGTCTTAATTGGGTATGAGATATTGGTA

CAGTTCAATCAAAAGGACGTGTTTTATGAATCTAAAGCTTTCTTATTTGATTGAGCCGTGCCAATATCAC

ATATAACTATCTATATTTCTAGAAATTGGCAGCGCATTAAGGAGACAAAGGTTATAAATTTTTCATTGCT

TTTTCATTTTCTTATGTGATGGTTATTGTTTTGAGTACAAGTAATTGGGGATTTTTTATTTGCAGATGAA

TCAGGATCTTCATATGAGGTGTATTAAAGCATGTAACAATCGAACATGGAGTGATGATCAATTCCTTGAA

GTGGGATTCAAATTGAATTTGAGAGGGAGCTTTCAAGGTACATACATGCATTAATATTTGTGGAAAATAT

TTTCATCTAAAATTACACTGATTTGGTGGTTTAAAACATTGAATTTAATTTATGTACACAATCACAATAA

AGAATATTATTGTTGTCTATTAATCATAATTGTCATCTTACAGAAACAAATATTTGACTTCAATTATAAT

TTTTTTAAAGTTATATATAAATGATT

>Ca_linc_0046

TCTTCTCCCCACCCCTACATACTCCCAAAAAGAGCATAAGAAAATCCAAAAAGGACCACCTGTAAAACTT

AGGCAACTTTGGCTTTATTTAATGAAACTAATTATTTTTACAAAAAAAGGGGTCTTGGCTGCCGTCTTTA

CCAATTGAACCCTTTTTCCATTTCTCTTGTCTCTCCCCTCACTTCTATCTTCTTCAATAGCATCTTAAAT

ATCCCCTAAAGAGGTGTATTACCTCCAAAAGCAAGAAGACTCATTAGAAGCTCTTTCAAGTTACTATCAT

ATATTGTTGAAAAGTCAATTTTTTTATAAAGAAAATGAAGCAGCATAGCATATATAACCTGCAGGTCCAT

GTTTGATTGGAATTGGGGTGGGAAGACAATGCATTGATGGAGTTCATGGGGGCCTGGCTAGTGAAGGGAA

ATTGAGTAGTGATGAAAGAGTGGTTCAAGTACTGCAAATATTAATGACAAAGTGAGTGAATTATGTAGTT

GTCAAATGCTACTAGACACTAGTACTACCATATAATTGTTACAATTAGTAGATAGTTATTATTAATGATT

AGTTGAATGTTGGTTATATTAG

>Ca_linc_0047

ACTCATAGTTTTACCTATTTAAAATCATAAACCAGACACATAGGTAAATAGTATATAGTATGGCGTCAGT

TGCTGATGCTGCTTCGAAAATGAAGAATGAATTGAGGAGTCATGAAGTCGCCATAGCCGAACTCAACGCC

CTTCCTTCTTCCAGAACTGTCTATCAGAGAAATGGAAACTTGTTTTTTCGCACATCTATCCAAACGGCAA

CAAGCATGGAGCAGAAACAACTTGATTCAGCCAAAGCCAAGCTAAAGAATCTGAATTCTTCGTCGTAGTC

ATATGCTAGAACTTTGGTGGTTCAGTAGAAATGCTGCATTTTCCTGTGAGATGAGGGCGATAAGAACATG

TTATATGTTATCACTTGGAGACTTCATTTTCTAAAGAGCTGTTACGTCCTAGATACTCTAAATTACATAA

AATGCATTCACTCCAATGATACTGTTCTCGGTTGCAAAATAAACGTCTCATGAATACATCAAAATACTTA

ACCAACCTTTATGAATTTTGTTCTTCATGATGGTTTATTTTAATTTGTAGTTTGTTTTTTGATTGCTTGC

TTGACATTTTATCATTGTTGTGTAATTTATGAAACATATGTAGCTTTGTCCATTTCAATAGAAAATTGTT

CACCCCGCCCCAATACATACATACAATAATTATATAAATGTCATTTTATATTTTATTAAATAGTTTAGTA

TATAATT

>Ca_linc_0048

ATGAACCCTACATTTTGAATTTGAATTTGAATCCATCACCGGTTCATATTGAATCTGAAATTGAATCCAT

ATTGGTGAGCCCTTCTCTCACACTTTGTCAAGGATGAACCCTACATTTTGAATTTGAATTTGAATCCATC

ACCGGTTCATATTGAATCTGAAATTGAATCCATATTGGTGAGCCCTTCTCTCACACTTTGTCAAGGATGA

ACCCTACATTTTGAATTTGAATTTGAATCCATCACCGGTTCATATTGAATCTGAAATTGAATCCATATTG

GTGAGCCCTTCTCTCACACTTTGTCACCGATTCTCATCCCTCTCAATGCCATGAGTATCGACGCCAAACC

CGTTCCCATATCGAATCTGTGAGTATCAAACTCGTCCCCCTCTCGAACCTGTGACCCTCTCGAACCTGTC

ACCGTCACTCTCAGAGTCGTCGTTCAATGTCGTCGTCTCGCTCTCATCTGTGAATGACTTCAAGGAAAGG

CAAATGGTTTAAACTTGGATAGTAGTCGCATGTGTCAGTTTTGCAGTGGTTTAAACTTCAAGTAGGAAAG

GCAAATGGTCTAGAGCTTTATCTATAAATAGTAGTCGCCTGTTTCAGTTTTACAGCGGATTATCTTGGAT

ATTCATTTTGCATAAGTTGAAAAGCAAATGGTTTCAATTATATGTTGTTTTGGAGATATCAACCTCAATG

CAAATATGACTTTTGTTGTTGAAGAGTTCAAGAATTGCATACTCCAATAAAAGATGGTGGTCCTTTCTGG

CAGCTTTAAAAGAACTCGCCTTTTGTGGCTAGACTAGATAATATCTACAAAAGTTCAAAGTTTTGTTTCT

CCCTGTTAAAAATTCTGAGGTAATTATGATTTTTTTGTAGCTTTCTAT

>Ca_linc_0049

AGCACATCCATAATCGTTAATCCATTCATCAAAGCAGTCATCCGTCTCACACTCTCTATTTCGTCTCTCA

AATACTCAAACTCAACATCTTGGAGTCTCGTCTTGGTCACGGTCAACATCGCCGGCGACACCATCTCCGC

CGTCGCCATCTCCATCGTCTCCGTTTAGTCTACAAACATGCAACAACAGTACTGATAATATATAGAGGTT

GTTCATATCTAATTTCTCTGTTGAAACTTTAGAGAAGAAGAAAAAATATTCATGCTGCTGAAATTACTAG

TAAATTCTCATGATGCAGCAATATTGTCTTCCTTTGGTTAATGTCGAATTTTAATTAAGTTGATCACGAT

GTAATTTATACTTAAGGATGTAATTTATACTTAAGGATGTAATTTGTATTGGTTGTGTTGTCATGTAACT

GTGATTTATGTTGCTGATGAATTTGTGATTTAGTATTAGCCTTATACTTTATGTGGCTAATTGCGAATGC

TATGTGTTTGAGAACAATGATACAACAAAACATGATGTATGATTGGCAGGTTGGGCCAGTTTTTATAATG

AGTCATAGTTGTATTTAGAAAAATTGAAGATATTATACAAGTGTGATGTATGATTGACAGGTTGGGCCAT

AGTTGTATTTAGAAAAATTAAAAATATAATACAAGATGTATGATTAGCAGATTGGGC

>Ca_linc_0050

CTCGCTTTGTTCAATTGCTTGCCATGAATTTCTCTCAGGCTGATGATCATATCGGCTGTCTGGCTAAGCT

ATATTTATAGCAATTCTCTCCGACTTCACCTGCATCCCTGAGTTGCTTTTCCATCCTTTTTATTATTTTG

CAGAGCAAAACATTGTTTTCTTGTAGCAAGCTAAAGCTTAGTAAGCTTTAGCTTGAGGGGGAGTGTTAGA

ATATTAGAAAATATCTTATAATATCTTTTATATTATATTAGAGTATCTTGAGTTATTTTCTATGACCAAT

TTATAATCATAGAGAATCTTAGAATATCTCTTATTATTATTATGATTTATTCCTAATTTAGGATTTAGTC

TATGTTTCTCTATAAATAGAGATTAGTATTGAGTCTATTGTAATAAAATTAGCATTAGGCTATTATCAAT

AATATTCAAACCCTTATTATTTTCTCTCTTCATTTTCTCTAAAATCCCACAACTTCAACAGTTCGAACAA

GTGGAAGCAATATAAAACATTTAAGATATAAGTTAGTGTGTGAGTTTGGTATGCAATATGATAAGAGTGG

AGTTTTAGAAGATTCACACAAAGAAGTTATCATGGTTCAATTCAACCAATGTGGGCTAATCTAGTCTTCA

AACTTACGATTAGTCACTGTGACTTTCAGCTATGTATTTTCTTAGCACACATGGCTTACAAATTGTATGC

TCTTAGCCTCAACATGACTACACTCTCATTCCAACTAACTTTCTTAGAAATCTCTAGGAAAAGCAAGTAA

GGAAATGTATAATTTGTTTGATATCTAGTTATTGTTTGCTAAGATAAACTTACGATAGAAAATGAGTTTC

AACACACTCCAGAAGGATGATATCTTGTATGGTTTGATTAGATAATTGTGGATAAGATAAAGAGAATAAT

GTTGAGTTTGAGTAACTAAGGTATGTGTTTGCAATTGATCCTCATTGAGTTGGTCATACAGAGAGGGGGA

TCTCACAGGACTATCCTCTTGTTCTATGCAAAATGAGAAGATTCTTGTTAAGCTCACGACCTTGGTATTC

GGGACTGGTGTGCTGTCACATTTCTTTCTTTTTCCCTTCGTGTAGTCTTGAGCAACGCTTTTGTCCTTTT

ATGCTAGTTCTCATTTTTTCATATGAGAGGATGAATCTAATAAATGGTCATCCTCTAATCTCAAAGGTTT

ATCTTATAGATGAAAGGTTTCTACTTATTTCAAAATACTTGATGTAATATTTGTTTGGTCCATGAGGATA

ATGTTGAGATGCTTAACGAGGATGGTGATCAAAATCTACAAGAGGATGATCATTAATATTGATGCATTAT

GCTTCTCAAGTGTCAAGTTTACAGAAGTCGATTCACAACTTGTTTTATACTAAATCCACAGAAATACACA

TGTTCAAGAACACAGAAGATCAGATTGTTTTGTCATTAGAATCAAGTGGTTCGATATCTTCAAAACATCA

TAAATGTTTTTGGCTTAGCATATGGAAGTTGTTATAAGGCATGGTTTTAAGTCTCTTCTTTCCTAGTCAA

TGATAGGAAATGTCTACTATTCTTTGGAGACCATGGAAGAGTACAAATGCCAAAATTTAGGAGAATATAT

CCAAATAGCCGTAAGTTATTGTCAAGTGTGGCTTGGTTCAGATGGAAGATTGCAAGTTGGCATATAGATA

AAAGGGTTTTCTAACTTCCAAGAATTAAACTTGAAAGTTGTCACATGGTGATGCAACCAGATACTCTGAT

CGTTTCAAATGTAATATTGATATGTTGTAATTTGCGCGCCAGACTTGGTTGCTTTGGTATTGATATTTGA

TCAATATTGCTTTGTTATTGTTTGATCATTTTGAATATCTCCATTTTGCTCGATAAATGAGATATAGACT

TTTGAATTGGTC

>Ca_linc_0051

AAAAAATATACTTTCGAATAGATTTTATCTAGAAGTTAAAGGACATGTACGAGATCAGAAAAGAAGCTCT

CATTTTATTAAATGCTGCAATCCCTTATATATAATTTCATAGTCCATACTATCTACCATATAAATTCACT

ATTCAAGGATTATATCAATATGCATGGTTTGCAAAGTTGTCCGGATTTCTACAACAGGCATTCCTTGACA

TTCAAATCAATGATCTAGCACCAACAGGCATTCAAATCAATGATCTAGCACCAAAAGATGGACAATGGCG

AATTTGGACCACCGTTATAGGGATTAATTCAATGTGGCACGTGAGTGATTCAAAGTCCTACAACTTGTTG

CTTGTTGATAGGAACAAACAGTAACATGGCCCGCACGTTAAAACTTATAATTCAATTGATGAAAAAGGAA

CATGATTTTGGTTTATGGAATCAATAAAGCAAATGGTCACTCTTCATCTCACTTGATCAATTCACTTCAA

TTACATGTCAGAAGACGACACCATGCGGATATACATGCATTTGCATTGGCTTTATTCATTCATTTGTATC

ATTCTTTTTTCCTCTTGCTTACTTTGTGTGGGTCCCAACCAACATTTATATACCTAATACCATACGGTGT

CTTCTGGATTTAATGCTTGTTATTTTCCCCCTATTGCATTCTAAAGTGATTGAGTTTGAG

>Ca_linc_0052

ATTTTGAACAAAACAACAAAGAATGAGAAAAATACCCTTTTCCTCTCTTGTTGCTAGAATTGGCTTGAAG

ACTAGGGTTGATGGGAAGCAAACTGATGGTAGATCTTCCTTTCTTCTTTGCGGCTAAAGAGGAAGGGGTA

TGTTCATTGTATAAATTAATTTCTGAATTTTCTGCTGCTTTTGGCAACTATATCCAGCAAGCAAACTGAT

ACAACTAAAGGGAAGGGTTGTATTCATCATTAAATTCTGGATTTTACCCTGGTTTGGCAACCAGATCAAG

CAAATACAATCAAGGAAACTAGGGCTAGTCTTTTTTGATTGAGTATTCAACATGATAAAATTAATACCAT

TATTAGATTGAAATATACTTTTTATTTGAATTGTGGACAAAATTGAGGTATTACAATTCTTGATTAAATT

CTTTCAATGGTGCTAGAAACAATCATCTCTAAGTTTCTCTGCTATGACACAAATCTCCAAACTTTCAAAA

AAC

>Ca_linc_0053

CGCCTACAAAACCCCAAATTTTAGGTTAGAGCCAAAATACTTAAATTACTTATTTTATCATTGGAATGCC

TAAATCCTCTTAGTTCTGCAACATCAACGCCACGAATAGAAACAACAACTACCACGACGACGGCAACAAC

CACTTGCACGATGGCAGCGCTATTCCTTGGCTACAGCAACCGTTGTGACGACATCGCCTCCTCTCTCACA

ATCTATTTCACACCACCCCATCATTTCTCTATCGGTACGTTTTTTCGTTTTAGGGTTTCTGTTTTTCATC

AATCGGTAACAATCAATGAATCGATTATGAAATAAACACAAATTAACGAGTAAACACAAACTATCAGGAA

TTGAATCTGGCATTGGTATTGCGTTTACCCTTATTTGTTCATGCATTGGTACAGGTTTTGAGCTAAAGGG

AAACTCAACTAAAAGGTTTCGAGTTCTTTTGTTTATCATTTCATTTAAATCTATCGTTACTGAATTGTTT

ACTGTGAATACCTATCTTTCAACTTGTATCTCAATTGGTGATGTTGTTAGTTGTGCTTGGATTTTTTTCT

TTGGTTTGTGATTGTGCTAGAAATTAGTGAAGATGTATTTTTCCCTATGAAAACAAAAAAAAACTTCTCG

AAGCTAAAACATAGTATTATAATGCTAGAATTGGATTTCTAGATGATAAGGAACTAATGAACTAAATGTT

AATCTGTTGAAAATTGTTTTTGTTAGTTTTAAGTTGCCAAATAGAATTAACTTGTTCTGATGTAGTAAGT

AATGTTGCATTTGATGAGAAAAGGGCCTAAACGAGTTGGAGCCGCATATTAATAAAATAATTATGGAGCA

TTAGTGCTTGGCACATTTTTCTCCATTCTGTTGCTCATGGATGTCTTAAATATTGTTTGAGAATCCTATA

GCTTGCATTTGTGGAAGAGATGTATCCTTTATGTTTTTAGATCTGACCTGTTTGATTGTAAGAAAAAGTC

TCGGTTAATTGTTTACTGCAAATTTGGAGTATGGAGACCAGTAAATCTTTTTTGTTTAGTTACAATGCAA

ATTAGGATAGTATACTGCTGTAAATAGTATTTGTTGTTGCCGGTTATTATTTTGCAAATTGTGTGTACAC

ATGGTAGGGAAGTTTATCTGGTTTTTCTCTTCTTTCCTTTTATTTAATCTACTACTTACTAGAACCCTTT

GTTCACGAAATGCATTTGTACTAAACTATTTACGTAAAGAAGCAACTTTTGAAGACAATTTCCTCTACAT

TTATTTTCCACCCACAACACTTAATAGTTATCTCTCTATTTGGCTTAAGTTGTGTTGGATTGAAGACATG

ATAATAATTTATGGCTAACAATTTCTCATAATCATCATGATACCACTTAGATAAGTTACATTATGGCTGA

TGATTTCTATTTGGCTTAAGTTGTGTTGGATTGAAGACATGATAATAATTTATGGCTAACAATTTCTCAT

AATCATCATGATACCACTTAGATAAGTTATATTACGACTAATGATTTTTCTTTATAATTGTGCATGGTTA

TTATGTTGTACGATGTTATGTTGAAATAAGAGTTATTCTAGTTGCAATTGAGGTGCTGAACTACCATCCA

TTGGTTGAGGTGAAGATTTAAAATTTATTTGCCGTTGAAGAGAGAGTTTTGGCAATTAAATGGATATTTA

ATTTTGTTGTTGTTGAGATTGTTATATAAAACTTTGTAAGCCTATTATTTTGTCAGTAAGACACTTCTTG

TATTATTAGTAAATACTCTAAAGTTAATATGATTTAGCTATTTTGATATGCTTGTAAGAGTTTTTGTTAA

TTTATAATATATATGATGAG

>Ca_linc_0054

GCATCACTCTGCCAAAACGATGAATCCTAGATTGCGATTCCAACTATAGCTCGTGATTGGATCAAGTTCA

TCCACCTCATTCCCTCTTCTTGACCATTGTTGATAATTCATTTGTTTATTAACCAGGTTGGAGAATTCGA

AAAAGCATGAAGAGTTCAAGCTTTTGTTCACAATTCCTTCTGACTTCCTCCTCTTCCGCCCCACATATGA

CGTGCCCGTTGTCGCGCTTGCCTCTGCTGCACAGGTAACCTAGCTACTTACCAAGTGAAAAACTATTCAA

GAATAGTTGAGGTCATGGGTTTGTTTGGTTCTTATCCATTATCTTGATATAGAAAGTTGTAGATTATGAA

AATTTTTTATCAACTCATTGATTG

>Ca_linc_0055

CTAAGTTTCAAAGTAGCCATCATTTCAAAACACCTTAGCCTCATCTTTTTTTTTAATACAAACCAGCATA

ACAATTTAGCACCAGCATTATTTCAATAGAAACCAACATCATCTCAAAATAGAAGTAAACCCATTCAAAG

CTCAATAAATCCTAAGCAAGTAAACCTATTTTAAAATAAAACATAAACAAGTATAACAACAAGCATGACA

GGGACACATATCAAAAAGGAGAAGTGGCGGCGACGGCGGCGGTGAACGGTTGATAGCAGCAGTTACCGGT

GAAAATGACGTTACAAAGCAAAGGCTGGTGCCTCAACCATGACACTGTATCAGGAACAGAAAGGCAACAG

TAAGGATAACAACAAAACAGAGCCAAAGCAGACTGGCTTAATTTGGTTTAAATTTGATTGTAATTGTAAA

TTAAAATTGTAATTTGAGACATAATGAAATTATTTATGGTAATTATTTTTATTTTCT

>Ca_linc_0056

GTCAAGTAGTGGTTTCATGGGGTTTCTTTGAAACAATAATATTAATAATTCAGTGTATCCAGCTAATAAA

TATATACCTGGTATTACATTTTGGAAAAGCTGTATATTGGCAATCAGATTCCTTCAGGAAACACTATTGG

ACACTAAATGTAGGTACATATCTCAGTTAGGATTCGTACTATGATTCGCCATGTTGTTGGAGTTAGTTAG

ACTCGACTCCGTTGTACTTTAGAATGCTATTTTACATCAGAACATACAACATCACTGTAACAGATTGGCT

CCCTCAACATCCCCCCTGAACTTGTTGGTTGGAGGCTTGGAGCATTATTCTAGAGAATAAAGCTGACTTG

ACATACCAGTATAGTATCTGCTTGCAAATTCCTTTACCTTTCTTTTCATCCTTTCATGTTTCCAGTCTAG

TCTGGACAAAAAATGTGGCATCTAGTTTAATATCTCTCTAAAAAAAAAAAAAGCTTACAAATAGTC

>Ca_linc_0057

TGGGGTATCGCCCTAGCTTTTCTTGGCATAATATCCATGAAGCTCTTTGAGTTCTTAATAAGGGTTGCTT

TTAGAGAGTTGTTACGGATCACTCAATTAGTATTTGGGGGGATATTTGGTTGCTGCTTCAAAATGGATAC

AAGGAAAACGTTTTGAATTGAAGGAGGCAGTTCAAAAGGCCTTGTGAGTCATTATAGATTTCAAATTGCT

ACTGCACTCCCGTCCCAGAGCTATCACTCCACACCAGGAAGTTTTTCCCAACAATTGGTCCCCTCTCCCA

GTCAACAACTATAAGATCAATGTCGACTCTACTGGCCTAATACATAATACTTGGGGCATTGGAGCAATCG

TCACAGATATTGAAGAGGTTGTACTAGCGGTTGCTACTTAGAAATTTGATGCTCTAATAGACTCATATAT

AGAGAAAGATTTGGGGATGCATCATTTTCTTCAATTTGCTATTGATTTGGGATTTTGTCGCATTAACGTG

GAAATAGACTCTTTTAATGTCATTTGAGCTATTCACGAACTTACAAC

>Ca_linc_0058

CTTAAAAGCATAAGCTATTTCCAATTGGATGTCAAATGATTATGTTAACCTTGAAATTTCATGAACGGAC

TTTGTTGAAAGATCATTTGAATTCTTTCCGAGGAATTATGAATCAACTCATTATGAATTATTTCCAAAGA

TATTACTTTTGATGATGAAATTCAAAGGTTAGTGCATCTTGGTTCCCTGTTTGACTCATTGTAAATTTTT

AGAATATCATTGTCTAATTTTGATCAGAATGGTGTGTTTTTAGTTAAACTTTTTAAAAGTAGTATTTTGA

ACGAGGAAATGAGAAGAAAGTTTCAGGATTTTCCTTTAAAGTTAGAGGTTTTTGTTTCTGAATCAAAGGA

GATAAGTAAGAATGGATATACAGGTAATAAAGACCAAAACATAAGTAATTCTAGGTATAGGTACAAAGTT

GTTGAGTGCTATCACTATGATAAAAAAAAATGGCATATTTAGAAGAATTGTTGGCTTTTGAAAAAGGAGA

ATGAAGACAAAGGTATACAAAAGAAGAAAGAAGAAGATTCTGATGAAGATTGAGCTAATGTGACATCTGA

TGAATTTCTTCTTATTGAAGAACATGATATTATTTTCATGAGATTGAACCCGTTGAAAGAGTTGAGTTAT

GTCATAAGAGATTGCGTCACATGAGTGAGAAAGACATGATCGTGCTAACTAAATGGGATATGTTGTCTGA

AATTACAAGTTGAAGACTAGAAAATTTTAATTTTGGTGCATTCTCCTTTATTAAGCCCACATGGAGCATT

CCTATTATATTTGGAAAATCAAAACCATTCGAAGATGAAGAAATGATGAATGTCCGACAACGTTGTGCAA

ACTTGATTCTTGAACTAATAGAAAATGCTTGAAGCACTTGTATCATTTTAGTATCTTTAGACTGTTAATA

GAAGATAATGTTCTTTTTTAACTAGTGAATATTATGTAGCTCTAAATTTGATAATTTAGATTTTGTAAAA

AAATTATGACTCATTGTCATTGATTTTTA

>Ca_linc_0059

CCACAATTTTCCTCATTTCCTTTGAATAGAAAGCAAACACTCCCTCCCTCTTTTTAGAAGCTTCTAATAG

CCTTTCCCTTCTATCTGTCATCCACTGTAGTTTTTTTTCTTGTGTTGGTGAGTGAGTTTTGAAGAAAAAA

GTGGATTTTACACCAAGCTCAAGTGAGATTTTGAGAAAGGCTAAATGCCTCCGGTTTTTGTGGATCAAAT

TATGTAGAGACTACGACTTGGTGGAAGCATAGTGGTGTGAAAACTTGGATATGGGCCAAATACTTTCGGC

TTGGTGTCACCGGTGGGTCTCTAGTTCCCTGACGTCCTAGTTTAGAGTTGTAATGATTAGCTTAGGGGTA

GTTAAAGTGTTAGTTTTGAAACCTTTATATTTGTATTTAATTCGTTGTAAGTTTACTTTTAAAAATATAT

GTTTTATTTTGAAAGCTATCATTTACCTTTTAAGTAGGTCGTCGAACTTATTTGCAAAGACTTTTGAAAA

AAGTTTCATTGGAATAGGAAATTTTTTTACAAGTATTT

>Ca_linc_0060

AAAAAATGAATGAGTTGATTTAGGGTGATGTGACATACTAGAACTTGAATTATGTTGAGTTCACTATTCG

AGTAGAAAATGAGTGAATTGCTGTGGCACAGTAGACTCTAGTCCATATTGAGTTCACCGTTGGAGATGCT

CTTATTTATGTGGCAATTTAATGCAGTCACCCAATAAAACAATAAATTAGAAAATAAAAAGAAAGAAATT

AAATAAATAAAAAAAACAAAAAACAATTGCGAGACTTCTTCTCACCGTAGCCATCGGTCAAAGCTCGCCA

CCATCATCGTCGTCGTCACCGCCACCGTCACACATCCCAGCGCCGACACTTGCCATTTTCAGAGTGAGAG

TGATCTATGACGAATTCAGAACTTAATCTCTCATCCGTTCAGTCCAATTTCTCCTCCATTCTCCGACCAC

CGTAACGGTCACGACTCGGCCTTCTTGTGTTCGCCACCGTCACGGCTCGCCACCATCTCTGTTTTATTTT

GTCGAGGTAAGAGGATGGAGGCAGAGCTGGTGTTACCTAACTATCTCAGTTTTAAGAGAATTCAAA

>Ca_linc_0061

AATTTCCTCTCTACATTTTAAGTATCGTCTTTTTTATTTCTCTTCCATTCTTCCGCTCGTTCTTCTCCTC

TTGTTACTGTGTTACTGTGAACCACCTCCCTCATCCTCCCAAATCTTCAACCTCACATTTCCTTTCTTTT

TTTTTTTCAAATCAGTAAACCCAAAAGCTTATAAAGGTCTTCAAACTCATTTTTAAACTTTGCATGTCTC

AATTTGTAACTAATGCTAATGAGCTTCAACAACGAATAACCCAAAATTGAAGAACTTGGGTCAAGGTGAC

AATTCAAGCGAAAGGGGAGCTCAGCAACCAAGTTAATCAGAACATCATCTTCTTCCTTGTCACAAACACA

GCTGGATGAACATGAGACATGGATATTAGCATCAGAAGATCAGTTTCAAACTCCTGGACTTAGAAGTTGA

TCTTTTGTTTTATGATGTAATCAGACAATCGCAGTTTGCTTTCAAGATGTAATATTTTATGAATCTATTA

AACATGTTGTAAACTATATTTCTGAATGTAGTTTTCAGTTATGATGGTG

>Ca_linc_0062

ACCATTCTTCCCTATCGAGTATCAACCACCAATACTCCACCTGCGATTCTTGCTTCCTCTATTTTTGTTT

TGGACAAGGGAAAAAAGTAGAGCTTCTGGTGGCTAAGATTTGGAACCTTCATCCCAAGTTAATTTTGTCA

AATGGAGGATAGAAGGTGGATCCGTGATCCATCGAAACAAAGTGAAGAATATATTCGAGGGGTTAACAAA

TTTCTAGAATTTGCTTTTCAAAATTCAGAAGTCAATGGAAAAATATGGTGTCCTTGTAGAAAATGCGCCA

ACTGTAAGTTACGTTGTCGCTCCAGTGTTTATGAACACTTAATTGATTCGCGGTATGGATTTCTTAGAGG

TTATACGCGGTGGGTATTTCATGGTGAGAGACTTGCAACTTCAAGCAGTTCCAAGGGTTAAAATATGGAT

TGGATTCAACGCTGTCAAAGAAAATTACGCATTTGGGCATCAGTGACCGTCCAATTAAGATTGGACGGTC

TAAAAAAATACCGATTTTGATGTTTAGTTTATAATTTAAAATCTGGACCGTCTGATCTTAATCTAACAGT

CACCGATGTCTGAATGTGTGATTGTGAAATTCCTCTGTTTGCATGAACTCGAATCCTGAAATACATTGTC

TATTAAGATTCTGTTATGTATGTAATATGATGTATTAAGATTCTGTTAGTTTTTGTTCCATAGGTTGAAA

TATGACGTTGATTCAAATGTAACATATTTATTTAGATGCTAAATGAGTTTATGTTACTATATTTATCAGT

TTTTTGGATATAGTTTATTTGTTCTGTTGGGTTCAAA

>Ca_linc_0063

AATTAACAAATTAATATTAATACAAAATAATAATTACATTACACTCAAATAGTTGGACACCATATAATCA

ACTAAGCTTATACAGCATAGGTGAAATGCAATCTCTCAATCTAGTACATAATAAGTGCAGCTTGATTTAG

AACTTCTGCTACAGATAACACTGCTCTGCTTAATACTCTAATCTGTTATCTGTTCTCTTAATGAATCGAT

CTTGCAATGAACCATAATGCATAGATGGAATATGATCCCTTTGATCATTGACATTCCACTAGAACACTTT

GTTTGGCTTTATTCTTGATACCCCCATTTAAGCATAGCTTTCTCTCAAGGCTGACCTACACTGAATTTTT

TGCCTATCTTACATATTCGTGGCTTTTAATGCCCATACACATGCCTCTTTTTCTTTTTCTCACCCCTTTT

ATTTTCTCTTTACATGTTCCCTTTCTCAAAATATCAACCATTTTTATGAGTGGATACCTAAAACCATTGA

AGGGGTAACAAAGATTAGATAGGAATAATAGCCCAACACCACAGGATTATAAATCTG

>Ca_linc_0064

CACATGTATCTGAAGAGGTAGATGAGATGTATCTTTCTTATCTCGATGTGGTTTATGTTGATCGTCATGC

TTAGAGTGTCATCCCACTTGTTTGTCACAGGTTTATTCCTTCCAAATAATTTAAATTTGTTGTCACGACT

ATGTTGAAACAGAAGTATCAAAAACCTTATAAAACTTGGGGTGAAGTAAGAAAGCAAGAAAATATAATTG

ACTGAAGGAGCACAGTAGACAACATTACTGATTCTTCATACTATGATGCAATACATGCTGAAATTGACAG

TATAAATTAAAGATGGCGAGAAATATTGGAACATGAGGAGTCGATGCAAAGACTTATTGAGACTATTATG

AAGAGGGAGGAAGACCAAATGCAAGAGTTTGAGAATCGATTAAGGAGAAGATGCTACCATCTCACCATTA

TAATTATGAGCGGCAGATGCAACCACTACGACCTCCTCATTTGTCCACCTCATACCAATATCCTTCCTAG

CTTCAACCGGGAGGATATTTTTCGACCATATATGTCATCTGTAATAATTGACCTAATTTGAATTAGCCAT

ATCTGTCGACATCTTTTCATGAGCAATCATCGTAAATTGCTCCAATTCAGCCATCTATGTTGATATCTAG

ACAATTTATTCTTCCTTCGGTTGGTCCCAATTCCCGAATCTTGTTGATTTGAAAAATTTATCATTT

>Ca_linc_0065

GTATTCAACTTGTTTAATTTATCTCACACTACCTTTTTCTTCATCTTCTTTCACATAGTTACCTATTACA

AGCCATTCACTTTACGTGTACATGAAATTCAGCACCTTGTTTTGCATGTTTGCTTCTCTACTCTTCACTG

TTCTGTTCTAGTCACACCTCAAAATCAATCAAATCCTTCATTTTCAACACTAGGAATGGCCATTCTTCAA

TTCCATTGGTCACTTACACACACATGCACTTCTATTGTTTATTCTTTTCAAAGTTGCAACTATATGATAG

TCTCTTCCATTATTTGCTTGATCATTCCTTTTATATGGCCATCATGTGAGAATTGGTTCAAAAATTTGAA

CCAATGTTTTGAAAATCGACATGGACCGGATGGTTCAACTGGTTTAAAATAGTTGAACCATAGTCTAGTG

TGCCCGGTTCAACGTCCAATTTGTTTTTTAAAACATTAAAATGAGATTTTGAGAAATATATTTGAAAGGT

TTTTGCTATTTAAGGCCAATCCATGGAATGAGGTTGTTGAAGTAACTACTAGGGTTTGAATGAAGAGGAA

GGATTAAATAAATCAAGTACTTGAACAGAACAATGAAGAGAAAAGGAGCACACAAGCAAAACATGGTGTT

TATTTCTAATTTTTAGTATTTAATGTGAATGGCTGGTAATAGTTGAATATTAAAGAAGAAAATTTTGTGA

TCAAAGTGAAGTTATCTCAGCAAAGTTTCTCCGATGACTAACTATAGTTAGCAGTCAAAAGGTTCAACTT

GTGAATGCAGGTGCAAAGGAACATGTGAATATACACACCATTAACAAAATGAAAGGAAAAAGCCTCTGAA

GTCAATAGTGTGAGATATGAAAGTTGATGTCCAACTATGCTTAGAGTAATATGGAAATTATATATATAAT

TTCAATTTGAAACAAAAGTTCTTGAAGATTCAAAAAATATTCTATCAATTATGCAGTATTGTTTTGCCAA

GAGTGTTTAGAATATGTGTAGATACATTATTGTAATTGGGCCAATTTTGAAAGATCAACCAAATTCCTTT

TATTAAATGTTACTTGATACTAAATAGTGCTTTCTGGATCCCTATTTTTTCATTCTCCAAGGTTTTAGCC

TCATTTCTTGCATTGCAGAAAGGAATTTAG

>Ca_linc_0066

CAAATGCACGCCGCTGAAAAAAAAAATAAAATAAAAGAAAACTTGCAGACTTCATCTCTTTTCTTTGGCC

ACCGTCTTTGTAACCAAACACCCGACACAATTCAATTCTTTTATCTTCCATTGTATACGGATGAATGACC

AATACCTTCCTTCTCTTTTCATTATCTTCATCAAAATCACTCTCGTTTATATGTCACATGAAGAAGAAAT

CAAACTCTGACCTATTCCTTCTGGCATCAACATTTCCGCAAAAGCTTTTTTGGCCGCCATAGTGGCGCAC

TAAAACATCGACAAGCAATTTCGCGTTCTCCACGTCGATTTGTCCTCTATCTCTTCATTTTTAAGTTTCG

CCAACATTCACGAAAACCCGACGGCGGTGGCGTTACGTGTTGTTGTTATCCATAAGCATCGTTTCAAGTC

ATTCGTGTCGTCATTTCAGGTTATCAGTTGGTGATCCACGCTCTGATAGGTGATACTGAGTGGGAGTAAT

TTGTCATATTTTATTGAGTTATGAATTAATTTAGATTTTTATAAATTCGATTGATCAATTGTCTTATTTT

TATCTTTTATTCAGATGTAGAAATTAGTTGTCTATAAGTTTAGCACGCGTTTTCATGTCAGAAATTTTTA

TTTAATAAACCTCAACTATATTCGATATTAAAG

>Ca_linc_0067

GTATGTGTCAATGTTTAATTGGTTTGACAATTTCATAAGAAATTTTTTTACCGTTGAATATCTCGTGATT

CTTCACTGAGAGGATGCAACATTTTTCACTATGGCGATTCTTCATTGAGCGTCTTGATCAACGTCTCTCG

TATCTCTCGCGATTCTTCACTTCGCGCTTTTCCAATCAATTTCAGAATCATTTACGATACTGTAATAATT

GATATGAGTGGTGTGATCATCAACAAATTGAGATTGCAAAGTCTTAATCAACTTTCTGCCAATAGATTCT

AGCATATGAAGAACAGGAGCAGGAAGAAAACCGACCCATGATTTTCTGTCACTCTACAATAATTCAACTG

TTGAACAAGATCCAAGGCCCTCTGCTCAAGGTTTTTCAATGTCATCTAAAGAATTACATTTGTGCAGCCC

AATAAGGACTTGAATTTGCAGTTTAATTTGCTTGATCGTTAGTGGATTACTGCAGGTCCCTACTTCTCAT

GGCAGTTATGCGAGAAATTTCTACAACCTTGCACTCCCACTCAAAACCTCACACTTCTCTTTTTTAATGT

CTCTTCCTTTGTAGAAAGACTTCCCAGATTTTTGTTAAAGGTTTCCATTTTGACTGGTACTACCGCTCGT

CTGTATAAGAAGGATGAACGACTTCAACACATTCTATATGCAAGTTGTGTTTGAAGTATTGAAGCATCTT

TATAGACTGTTTAATGCCATATTAAAGGTTTCCTCAAGTGGTTCCCCAATTATAGATTGGTAAGGCAGGG

CAGTGGCTCTTAATGTTGGGAGCAAGGCAGAGCAGTGCTGAAATTGTGTGACACTTCATGTTGTAACCAT

AATTTATTCTCAGTTAGAAAATAGTTATACCTATATTTGACGTAATTGATCTCAAATTTCTCCCTAACTA

TGTTACATTATTTTTATAAAATAG

>Ca_linc_0068

ATGGTAACTGCCATTAATTATCATTGAGGATAGTTTTGCAAGAACCGTCGTAAGCTGTGTGCCCGCAAAA

TTGAAAATTTCTTACAATGAAACAAATGAGTTTTCGAGCAGAAAGACATACATGTGGTTATGGAGATGCA

AATGACAATGAAGAAGTTAGAGATCTATCAAAGTGAGATGAGGCATGCATGCATAGATCTCAAGCTCTTG

ATGGTTCCATGATTGACAATCAATGATGAAAGAAAGACATCTGACACACTCCCCTTTCTGAAAGATATAA

ATTAATATTGATGTAGCCTCAAATGACTCTCATTGATGAGACTTTGATTGCGCAATTAGAAACCCTCAAA

CGACTCTCATCTTTGATCATGTAAGAACTAAAATGGTTCAAGTACAATACTTGTTATTGATGTTTTGATG

ATAACAAAAAAACAACAATTATTTGCTAATTGATTGATTTAAGTGTGCAGTTGCATATGGCCAAACACAA

AATATCTAATATTAACTATCTTCTAATAG

>Ca_linc_0069

CTTCACGCCTCCTTCTTCTTCCTTCTTCCTTCTTCTTTCTTTTTCTTTCTTTTGCTTCAAGAAAAGAAAC

CAAAGTGTTGGTGGGTGAAAAAGAAGAATGTTTTTCCCTGTTGCACCTCGCCTTCTCCTTTTCTTCTTCT

TTATCGGTTTCAAAGAAAAAGTGTTGGTGGTTTTCAAAACCACACACACACGAACATCTATTTATCTTCT

AGATCTAAGCTTCCATTCGAAAAGTAACAGAAGGGAAAGTTGCTCCTCTCTGCACTACGGTGCTTGGGGG

CTAAGTTTTGAAGTGACGACGCGAGCGAGATTTTTCACCGTACTTGAGCGCGGTAGCGGAAACGGATGAA

AAAACGGTAACTAAGTACCCTGGCGACGGGAAGGCCGTTAGTCATACCTGACCAAAGTTGTGTCAGGAGG

ATCTTGCAAGGCGCGTGGAGATCACTCAGGTTTTATAGTTTTTGTAGCATGATCAGATTAGATGGTTGTA

TAGGGGCTAGATGTCTTTCTTTTGTGGTTGTATTTATTTCAAATTGGATGATTTTACATATATCTTATAT

TGTCATCTTAACTTTTATTTTGATGGGTCATGTTCCCCTTTTTTATGTGACATGGAGAAAGTCGAGATTC

TTGGCGAGAATACCGCAGTGGTATGAGCTATGTCGGATATGTCTCATAGCCCTGGTGTATTTTGCTGTTT

AAATAGTCTGAATTTTTGTTTCAAAAACTATTTCCGCTACTTGTAAATATTGATGACTTTTGTCGTTG

>Ca_linc_0070

ATGATCATCCAAAATATATTATGGCAATTTCATATGAATAAAAAATTCATATTGCAAACCATATTTTAGG

TCAAGAATTCATCTCTTAATTAAATACCTGGTCATCGAAACATATTATATCAGATTCATTTCATATGAAT

AAAAAATTCATATTGAAGGCCACATATAAGTCAAGAATTTATCTCATAGTTAAATTTGAGATCATCCAAG

CATAGCGACTTTTCAATTTTTATTAACCTTCTCAATCAAGAATAATCAAGAATCGTCAAGATTATACATT

CTTGCAAACAAAAACCTACCCCAAAGCATTTGTGTCAAACACATTTTTGGAGACAGAGAATGTTTGCTCA

ATAGAAGAAGGGAATCAATAGACCAGAAACCAAAGGATAATGGGTTGAAAATGAAATAGAGAGATTCATA

TCTCTCTTTCAAATGAAGCAAATCATGGACAGCAGAAACTTGAATCCAAATAACAAAGTCGGTGACTTCG

TTGCCATATTTTTACGGCACCAATTCAAATCAGAATTCTAGGGTTCGAATGACCAAGATTCATAGAGATC

GGGTGTTCTTGGTTGAATTTCAAGAACAGGAACGGGAACGGAAAGCTTTATATTTTATGTATCATCATCT

CTTTCATTCTTGTGACTATGCGATCAACTTTTTTGTTTTGGCACCAATTCAAATCAAGATTTCTAGGGTT

TGAATTTCAAAAACATCATTACCATTACCGTCAATGTCGCAACGAATAAGAAGAAAAAAAATTGGCTCAA

AACATCGACACCAAACACGAAGATGATACCGATCGAATCGATTAATGCAGAGGATTGAAGATTGATAGGG

AGAAAAAGAGATATCGAATTTAAGCGATAAGGGTTGAGAGCACACACCTCTGCTTTTCTTTTTCTGGTTT

CGGGGGAATAATTGAATTCAACACTGAAGATTCTATTTTTTCTGATTTC

>Ca_linc_0071

CTTTACAATTCACTTTACCAAACAAAATAATTAATATGAGATATTATTAAGCATGTGGTATGGCAAGGAT

CTGTTTCATTGCAGGCAAAAAATCGTTGAAACAAGTGAACAGCGTCAGTTTAAGGCTAGATTTTGAAGAA

ATCGGTTTAATAGTAGACTAGTAGATAGCTACAAATATAAATTTTCACTAGCTTAAGTCTCA

>Ca_linc_0072

CCAAATTCTTTTTTTTTTCGGTTCTTCTTCTTCGCGTTGTTGTGATTTGATATAAGCTTAAAAAAGTGAT

GGTTGATGGTGCATTTTAACCTACTCTGAAGTTTCTCCTTTGGCATGGTCATTCGTGATATGAACAACAA

TTATTGCCGCAATTACTATAGCAGTTTAGGTGTTGAAAATGTTCTTTTTGTTCAAGTTTGGACTCTTGTA

CATAATGTTAGGGGTGTTTCAAAATATAGACTTAAAAAAGTTCTATTTGAAAGTGACTCTAAACTTCTGA

TTAACCAGGTCAGATGTATGTTCTCTACTTTACCGGATCTCCTATCTCTTTTGAAAGATATTCACTCTCT

G

>Ca_linc_0073

CTAATTCGGTTTGTACGTGACACACACACTTTCATTGCCATTCTGTTGCTTAATTGTCTCTTAGTGTGAG

CTAGCTGTTTTGGTGTACAAATTGAAGGTTTTTCGTGTTCATTAGGCACTCCCATTTACTTTTCCACTTC

ATTCATTTCCTTGCTATTATGTAAAAGGTAAGAACATGACAAAATAAAGAACTACTAGTCTACTACAATC

AATGATTTTTGTTACTATTTTTTCTTGTTCTACAAGTAGTGCCAACTACAGATTAAATTTGTTTTCGAAT

CAAGTAAGTACCTATGTATAGGCCCAATTTCAG

>Ca_linc_0074

CTTTTGTGTTTCCCTTCTAATTGATGGTGGTTGTTGTCTCCTCACTAAGTCTTTGTGACTTACCCCTTTA

TGTTGCTTATTATTTTTCAGATTCGCAAGCAGCTGAGTAGGAATCTATTGTTTGATTCAGGTCTCAACAA

ACTTCTTTTTTTTTGGTAGGCCTCTTTGATTGAGGACCATTTTGTAATATTTGTTGAGTCTATGTAATTG

CAGCTATTTTGGAGTCTAGAAAGTCTATTTAGTTTTTTTGAGAAGTGTAGTAAGCAATTTGATTATGTTA

TTTGAATTATGACTGGATTGAAAGCTGAACAGGGATTTTA

>Ca_linc_0075

CTATACACGTCTCTTTATTTTTTTTCACAAACAATCATGATCATAGTCCATAACATCTTAATTGATCGAT

TAGAAACAAATCGTGTAATTCATTTTGGATTATTCTTTTGAAGGAATTTTGTATGTTAATGTAACTTTCA

TACACTCATTTAACACTATTTTCTCTCAATATCTCTTATTCTATTACATCATTCCCTCTATTTCTATCTC

TCATTTGTATATATTAATGTGTAATTTCAATTTTGTATGTGGATCGAGAAAACATCTTATGGACCAGATT

ACATTCAGTGAACCTAATAACATCTTAAGCATTCATGCTTGAGGGTTGTGGAGGCCTTATGTTTGGGGCT

ATCGACTGGGTGAATATTTACATCACTGGTCAGGCCCACTAGCATTCCATCCATTTAAACAGAGACGTTA

GTCCTAAAAAATTCGCAACAATAGTATGGTTTTTTTTTTTTTGACAAACTGAAAG

>Ca_linc_0076

ATTCAAAAGAAATGAAACATCTAAAAACGGTCACAACAAGATGCAACATATTTCTTTTAACAACATAATA

ATCACGATTTTTTTAAAAGGTATATATTTCAAAATTAACTAATGCAAACTCTCAAAATAGTATTTTATTT

TAATTATTTTTACAAAACATGGTGATAAAAAAAAATTCTATACACCATAGAGTGTGTATTACTTCGTCGT

TCTTTTAGAGAAATATAAATTTTGAACTGAATAGGAAGGGGGCCGCGCGAACGCTGGCCCCCACTGCAAC

AAATTATGATGTAGGCTGTTCTACCTGAGAACACCTTAAGCGGACACCCCTCCAAAGTGCAATGGAGGTC

ACCAACTTAGGACATGGACCTTATTGATCATCCATAGACCCCAT

>Ca_linc_0077

AAAATGTCATCATGTTTTTTAGAACTATCCACCTGGTTGGTTTTGTTTACTCATCTACTATATATTTGTA

TTTAGCTTAATTAGATTATTGGGTTGATTGAATTTCACCTTTTTAAGCAATTTATGCATACATATATATT

GATATACGGCAAATGGCAATCAAGGGGAAATATGACAAGCATTCAAATGAGCATATTCAAGGTCAAATTA

AAAAGTGATAAGCTAATTGCTAATTACTCAAATATGAGTTAGAAATGAATGTTGATACCAAATTCATTAA

ACTTATAAGAAACCTGAAGGTATATATAGTTAAACATTACCAAATAAATAGTTGTTAAACATTATTCAGC

AATTATTGGATAATATGCATTAGCAATTTAACATATTACTGAAAAAAACGTTAGCACATTTGATGTTACA

ACAACATGTACTAATATAACTCTAGAAATGTTTCATCATGATAAGAAATAACTAACAAGAAGTGCTATAA

ATACACAAACACATTATTCTCGCTCAACACACTATCTGATGAATTGATATTCAGATATGTGTTGAAGTGT

TCTACTAAATTATATAGATTCTATATAAATTTATTATAAACCATTGAAATTCAACAAATTAAAAAGTGTA

AATTGATTAATCATAAGGGGCCATAGCTATATTCATAGCCATGTTTTCATTATTTGTGTCTCTAGGAAGG

GCACCTCCCTGCAGCTTGGGTGAAACATTAGCACCTGAAGGTGGAGAGAACCTACTAACTCCATAGAAAA

TGTTTGGAATAGAACTTGGAGGCTTTGGAGGGACCCTACTAGCTTCACACAAAAATGTCACACTAAGAAA

GACCAAAAATACAAAGATTAATGTAATACATCTTGATTTGCCCATTTTCTTAGCTGCCACATTAATTGCT

TGTGAATGTGCGATGTTTAAATATTAAAGGGTTGGGTTAAGGACTTGGAAATGAC

>Ca_linc_0078

GGAAAACCCAATCCCCGTTGCAGACAAGATGAAGGTCTAAATTCTACACCCGCTATAAAATGGGAGTAGA

TGTTGGTTTTCTCCAATTAATCAAAGATTAAGGAGGCAAAATTCGTGTCCGACCCACCGTGTTGCCATGC

CTATAACGGTCAACATCATTTGGTAAAAGGATTGTATTATCTGTCACAAGACTTTAGAGTAGGAAGAATT

TGCATCCTCGACAGTTGAATTTTTACTAAAAACAACTTGACAAAAACATAGAGTTTTGGGTGATCGGAAT

ACCATATGTGCTTGGAGACTTGAGAAGGAGGAATTTCTAGAACATTTAATTTTTTGTTGTTTAATTTTTG

GCTGTGTAGTCATATATATTCCAACTCTTGGGAGTTTAAAGTGGTTTACATAATGCTTTTGCTCATTCTC

AG

>Ca_linc_0079

GTGTTCTTCTATCTTACCCCTAGTAGCATGCTAACTAACAATGATGTGGTTTATGTTGAGTCCTAATGTT

TATGTTGTCATGTCAACTTAGGTAGTTAATTGATGTTCATTTTGGATCTTACTCCTCATGGTTTGAGGAT

AATAAAGCAACCAATTTCTGATTACTTGGCTGATTTGGAAAATTACTCCTCTTGGTTTAAGGTTAATAAG

GCAGCCAATTTCTTTGCCTGAGACTTTAAGACCAAATTGTCAATTGTTGCTGTTGTTTTAGTGTGGAAAG

TGAATGACATATATATGTTAATGTCTTTGAAATTGTATTTCAGCACTATTTGTTTGAAGCGCTCTGATTT

TCAGCCTTAAGACAATTATGGCTTCTTGTTTCTGGTTGTAGTGGCAACTTCATATCTATTATCAGTTGGT

GGATCATCTATTGTGAGTCATCCTCCTTCGCATCCTCGTTACGATGATGGTTTCGACGATCTGTCGTTGG

ACGCCCCTTGATCTCATAGTTATGCTTACTTTTTGTAGTCA

>Ca_linc_0080

CTATAACTAAATTTCTTTATAATTTTTTTCCACTTATATTATCAAGATCGGAGGGAATAATATCATAACT

TGCACTTCATCTTTTAATTAAAAAAAAAAACGCTTACACTTCATCTCGTCTAATATCTTATTAGCACGTA

TCTGACTATAGCTTCAACCTATATAAAAATTTGTTGTCTTAAAAATCGTAGAGAAACAAAAACAGGTCAG

ATCATATAATCATGATATAAAAACAGGGTTCTGATGTATTGCAATCATGTTCTTAAGACTACTTAATTTA

TGAATAAAAAAAGATTATCCAATTTCTATCAAAATTAAAGCCAGCCTGTTATTACTAATTTATTTCCCCT

AAATTTCTTTTTTGGAACTTATGTGTATCTTACATGATGGGTTTTCACACAATCAAATGGATTAAGTTTT

TTGTTTTACAGAAAATCACTATAATTAGTGTTTTTATATCCGCCTTTGAAAAAATAACACAGCGATTTAG

CTAGAAATATAAAGATTTACATGCAGAAGATCCATATTTCAATTCCTAAATCCTAATATCAAC

>Ca_linc_0081

TAAAGAGTTAGATATGCACCAAGATAAAATTAAACATTTAAAACGGAGTGTTAAATTTGTGAAAATGACT

AAATTGACAAATATTCTTTAATATAAGAAAATAAGATCTTATTTCACAAATTTGAGATATTATGACCCTT

TACATTTTCAAAGGCCAAGATGCTAAATTAACTTTTTTAGAATTTTCCAACTCTTCCTTCTCTTGAGTTT

GATGAATGTTGTATTTTACACCCAAAAATATGAATATCTCAATACATACTTATAATCAAATTATGATTTA

TGGTGAGAAATTGTTCCTTCTCGGGAAAACATTGTAAAATGCAAATTGACTTGTACGGTTTGTTAGAATT

TTGCACAAAAATCGATTCAATGATGAAAAGCTATTTTGGTTGTGATGTAACTAGCATGTTGCAGTGTGGT

CCATATTCTTGTGGATTGTGTTGTGTAACAAGTTGCCGTCCAAAAAAGGTCTTCTTGTTCAAGTAGTCTA

TTTTAGACTCATGAAAATGTTAATGATACATGATAATAATACACCTACAACTAATAATAATAATAATAAT

TTGTCACATCCACGAAATATTTAATAACAATATTTAATTTTAAAACTGTTTAATATGATGTGACAGATTA

AATAGTTATAATCAGACAATAGTGTAAAATAACTTTATATTAGTAGTGCACTATTTTTAAACTCTAATAA

TAAACATGTTTAAAATTCGGTGGGTACACTC

>Ca_linc_0082

TTTGAATCTTGCAAATCCAATAACACTCTAAACCCTGTCTCAATTAAATCGTTAAGTATAATGTTTTATT

CTTTTCACGGCTACATTTTGGTAACGCCATATAAGAAAAACAGAGTTGCATAGTAAATTATTGCATCCCT

AACATGAATTTTAAGAAACTAAAAAATGTTGCATTCAATGGACACACAATAGGTGGGAGATCCACCTTAG

TTCCAAAACTCTTTGTTTTTACTAGAAGCTTCAAAAAGTGTGTGAAAGATTTTTTACTCTATCATTGTTT

TCAGATTTTTATAGGGTTTAAATTCGTACAACTACTATTCATAAGTATAAAATCCCGTAGTTTATTTGTT

AATTGATAATTTATTTTAAAGAAAATATCTCTACCATTTAAGATACATGTAGATATCTATTTTCAAATGA

CATTTATGAATGTCTAAAATTTTGTATGTATGATCCATCATTAATATACTATGACTATGATCCACCAATT

GAATTAATTAAAATCACATATTGTCAAAAAGTTATAGAAAGGTTCAATCAATTTGATACCTGACATCATA

TCTTTGTATAAATATACATGGTTTTGATTGGAAAGTCGTCATCACAACCAAATACTATTGAAACATTATT

GTAATTCCTAGTTTCCATTTCATTTAATTAATGAGCTCAAATCAACTTAAACACACGTAATATAATAATA

ATAAAAGAAAAT

>Ca_linc_0083

GGAGAATTCTCCATAATACACAAATCAAAAGAGTTTAAAGTCAAACAAAAATAAACAAGTCAATTAGTAG

TTTGTTTTCCTTTCTGTCAGATATAGTTGATCACAACTTGTCTATCCATATTATGTAGTTCACGAGTGCA

ACACATAAGAGTTGGGATATTTTAATTGATTTTGCAGTAACCAGTCAACATGTCGACTAGAATCGTTCTC

CACTTGCAGGTGTGTCCTTGTCGACGAGTCACTTCCATATCCACATACATACCCCACATCTCAACATGTA

GAGCGTCACAAACCCCAATTTTCCGTATATAACTGATCATATACTTCCCAATGTTGTCACGAAAAAGGCC

TATAGATCCTGAAAGATTGATCAAGCTTTTGTGTGGATCGTCGTTGTTGAGTTTGATCCATCCTTCATGC

GACCTCTAACAGCTAATGAAAACAGTGTCCAATTGTTGAGGCCTACCAATAAAGT

>Ca_linc_0084

CTTGGGTTTGGACCAACCCTTCCATTATTCTAGAAGTCTAGGGAGGAAAAGTGAAAGTAAATAAAAATAA

AATAAAATTTTACATAAATCAAGTTTGAGTGAATTGAACGCAATTAACATTATCTAAAAATACAAATCAA

CCCTATCCACCTTATATCATCTAATGAACAAATTTTGAAAACAAGACAAAATTCTTTTCACCAAAAAACT

TTTAGTTACCAACAAACACAGTCAGCTTTAGTCTTTTAAGCATGTTGAACCCACAAGGCATTGTGGTCCC

ATTACATACTCCCATACCACTTTCCAAAAAGGCCCATGAAGCTAATAACTGATCAAGGCACTAGTCCTCC

TCACCTACCAATTTATGTCCCCTTCATAAGGTGAAGAATTAGAGAAACAAGAAATGGGATTGAACAATAT

ATTTTCAACTTACAAATGATTGTAATAAGTAGACATTGAGAAT

>Ca_linc_0085

GGTTTAGGTTAGGGTTTACGGCTGACAGTTAACGCTTAGATCTGAATCAGCAGGCATATAATTTTCTTTT

TTAAATTCTTTTAATTTTCCAAAATTCTCTTTAGCAACGCTGTTGTTGTATGATTTTGAGTTTAGTTATT

GGTATTATTCATGTTATTTTGATGAGGCTATATGTACCAAGAGAAGGGTGGTAATTAAAAAAACCAGTAC

CCTTAATCTTGAATGTGCCATTGCGGCTGTAGCCAAATTAAATCGAAGGAGCTTACTGGCCGGTTATTGT

GTTTCTGTCGATAAATCTGCTTACCGAGCTTCTCACATTTTAGTTCATATATACTCTTTTTCTTTTAATT

TTTTTATTGGTTTGATCCTTTTGATTTTTATTATGAGAAGGTTAGAAGAAGGAAATAAGTTTTGTATTTG

GCTTGTTTCGAAGATCTTCTCTTATATTGAACTAACTTTTGTTTTCAAAAGGGTCACTTTCCAGGCAATT

TGCTTTGATTTTTCAGCAAATTGCGTCTAACGGTGCCTACACTTTCTCACATATTTTCATCTGGACATTG

TTTGTGTTTGTGTTTTAAAATTTTCATTTATTTTGATTTTGTTTGTGTTTTTCTTTGACAATTTATTTGT

CTTGTTTTTTAAATTCTGTAGTTGATTTAGTAAATATTACATTATTGTTATATTGATCCAAAACTTAATA

AATTTTGTTTTAC

>Ca_linc_0086

NNTTCTCATCAAAATTTCGGATTTCGCTGTCTACAAAGGTTTCTTCGCGAACCCTGATTCACCAACAAAA

TTAACTTTTACAAATATTCCACTGCAATTTATATCTCAACATCAAGGTTTTCATGTCATTACAATTGTAA

TATATATGTAATTGAGGATGCATTATCTACAACCAGTTTATGCAATTCAATATCAAGAATTGTGACACAA

CCACAATTTTAAAACTTTGTATAGTAGTATTATGTGTTGTATAATATCAATATTATCTCTATAGGTAAAT

CATGTATTCATGCTGTTAGACATCGATGAATATTGAATTGAGATTGAACTATTTACATAACATGTGGATT

TTGACCTTTTTTTTTTTTTAGATTTTAAATACTGAACTTAGAATCTGGACTATTTGATTTTGATTCAATT

GTCGCGTGAATGCAG

>Ca_linc_0087

AAAAATAGAAATAAAATCATAAAAAGCATGTATGTATATGATGGAAAACATGTCTACTCTTGTTGGTATT

GAAGATGGAAAATTTATCAAATGTGAGATTGAGCACCTGCATGGCTCCCTTAATGCCAAATAAAAAGATT

GTCAATTTTTCTGACAATTTTTTGAGTTGGCATTCAGGGAGCCAGGCAAACAAATCATATATAGTCATTG

AAATTTTGCAATCCTCTACGAGAAACTTGAGCTTAACCAGAATCTAGAATCTGCAACATAAAAAACAATA

TTGAAGTGAATCTTAAAATCCAAAAAGTTTCTAGATAAGATAAACAAACACATATGACATATATATGGCA

AAACTAACCAAAATATTGACAAAATTATCAAGGACTATATACATAGTAATATAACACAATAACAAAGAGT

TAATATATGTGTTTGTGTGATGAGATGACAAATGAG

>Ca_linc_0088

CTTGTAGCTACCCACTTCAAATGCACTAAAATTTGCACTAAATAAAGTGGTCCTCTTTCACTTCATGCTA

GCTCACAAGATCCAATGTAGATAGATCCCTCTCTGCTCTTTTTCCCCTAAATAAATAGTAGGATGCTATT

TTATGCCATTTATTATATCTTAAAGCACGGGTGGGTTTTTACTTTTTCTATGTGAATTTTCCTTTTCCCC

ACTTAAATAGTACTAGAGACTCAACCATTCAAAACAATGCCTTTATTGTATATCAAATATAATCATAGTC

ACCTAAATGTCATCAAATTAGGTGGTCATTAAAATAAATTTT

>Ca_linc_0089

TTTTTAATAAATTTAATTTTGATTTTGCAAATATTCCTATCTCTATATGTGGTTCACAACTTTGACCACT

TTTTGAATCACAACACAAGATCTTCCAAATTGGTTGGCACATGAATGGAACCTTAATTTGAAAAAGGAAA

ATAAAAGATAAAAAAAGTAAAACAATAGTAAACAATTGTTGATGATCAGTGTCAATTGATGTTTCTCCAA

ACTTCAAAGCAAAATTTACATTATTTCTAGAAAAAAGTGTTTTTGGAAACTATGAGGAAAATTTGAATAT

TTTATTAGCCAATTGAAAAAAATTAATAATATGAGTAGTGTTCTTTCCACGTTAATGATGAAATGGACAT

TCAAAATAAAGTACATCAATAATCTAGACACACTTTGACATTTAGTGGCATGTCTATGTATATGTTTTCA

ATTCCACATGTAACAAGCAACTGCAACCAAACTTTGTCTTTGCAATTAGGTTTTGGAGCTGTCACTATAA

ACGACAAGATTACTTTAGCTACCAAAGACATCTCACACCCATATTCACATTTCAAGCCCACACTTTGATG

CATTTATAGAAATTTTCTATGGTAATTTAATAATTGTACTTATCTTTAGATTTCAATGAGGACAATGTAG

GCTTGCATTGGAGTAATCAACACAATAACGTATATATGTAGCATCCATGAGATAGCACAATAATAATAAT

AATAATAAAATCTTGTGTTTTTTTTTAAGGGTCATTATCTATAGTGTAGTACTATGTATTTAGATGTGTA

CTTTCTCTTAAGCACATGAACTTTT

>Ca_linc_0090

TTTCCTTTAATAATCTATATTTGAGCCTCTCAATTATATGTGAATGGTATGTTTATGATACCAAGTGGAT

CTATGTTAATATTTTGATGATGGAAGAAAGCAATAAAAGAGGAGATGAGAATCCATGTATGTCTACATTT

ATTTATGTTCTCTCTTTTTTGTTGGCCACTTATTTTCTTTATTTTCTTTATGTTACTTTAATCTTTATAC

CACACACCTTTTCATTCATTCATAGTGCGTGCTTGTGAGGGAATTTAAGAATGGGAAATGTTAGATGGCT

ACGGACAAAGTGGGTTAGTGTTCCTTTTTATTCAACCTATACACTATAGTACTTGATATTGAAAATAATT

GATATTTGCAATAGATCACAATAAAAGCAATCAATTTTTGCAGGCAGAAGATGATGTGTAGGTTCCATGC

ATGTTAGTTGAAACTTGAAAGTTTGCTAAATGATGTCAATCAATTCATATATAGGACCCCAAGAGGTGTT

CCTTGTTTCTTTCCGTGACAATGACTATTGCATATAGATGCCATTCATTTCTCCATAAACTATTTTCTTA

TTAT

>Ca_linc_0091

TTTTATGATTCTTGTCAATCCTATACACAATTATTACAGAGATTCCTCTTCCTTTGTCAAGAAAGAATTG

GAGTAATAACACGGTAAAACTAAGGGAATCCCTTCTTTTTTTGCATATAATAAGTGATAAGTTGTTGTAA

TAAGCAAGGAGAAAGAATAATAGCATAACAAAATAATGGGAAGTGTAAACATGTACACATGAACAATGAT

CAGGAAGGTCCTGCTACTAGTTTTATCATTTACTCAGGTCCCATTTGGATAGATAACTTAATTAAAAGCT

TAAAACATAAGCACTT

>Ca_linc_0092

GCAGAGTTCAACCTCTAAGAAAATTTCAGCATAAATTACCCTGAAAAAATATTTACAAAATCTTTCAATA

GAAATATGAGAACTTTAACATATATAAAGCAATCATGGAGTATATATAAGAGCAATATAGCAAAGTGCAC

TAATTGCTATAAGATTGCTTACACAGATTGGTAACTTATTGTGAATATAGGATACAATAATATTATTGCC

AAAGGATCAGAGGGCTGCCCTTCTAAGTTAAGATCTTGACCTAATTTGTAGGTCTAACATGTAGCACCAT

TTTGCCTTTTATTTTTCTTCTTAATTAGCTTCCATTTTTGATGCAAGTGCTGTGGATGTTGAGATCTGAA

CTTGAGATTCATTTTGAGATATGTGGCTGAAAGAAATAAATTAAAGGTTAATTGATGCATATATAAATGC

AACACTTTGGTGATGAGAGCAAAAAGATGAGAATAATAGTGGTGCTCAAG

>Ca_linc_0093

AAAGAAATTCCATTCCAAGGGAAGAATAGGCAAAGAAAAATCAGAACGAACGGCACAGCATGAGCACCAG

GTGGTTGTTGGACATGTCAATCTTCCGGTGGCCGGAGTTAGATCTGTCGTTGCCGTGGTCAATATTCAGG

TGGCCGGCGTTGGACTTTTCGTATTGGAGCAGCGGCCGTAACTTGCAAGAGACTCTCTTGCAATGGAATT

TTTGGTTGGTCGATGACGTGTTGTGGACTCTCGTCAGGTGTATCGAGTCTTTCGCTTTGCTCACCATGCT

CTGTTATTTCTTTCTCTGTTGTGGTTGCTCTCTTTGATTTTTTATTTACATTAAATTTTTCTCTAGATAG

TTCTTTTTTTGATAGAATTTGTGTCTATAGAAGTTGTGATATATTTCATTACATTAAATTTTTGTCTATA

GAAGTTGTGATAGAATTATATATTTTTTAGATGTGCTGTGAATATTTGTTATCTTGAAAGTGGAGATTAC

AACTATTGTTGTGAGTTTTGAATTATTGATATGACACTCTTTATGATTAAGAAAATAGACAAAATTTAAT

ATAATCATATTTATTGGTCTCGTGTTGATTTTAGATACTGATATGTGTTAGATCCAAGTAGGTCTTTAAT

CAAATAA

>Ca_linc_0094

CAGCACACCCTAGAGTCACTTTTAACACTATTCTTGCCTAGACTACTATATCATCTGGTTTGTGTAATAA

CCCCACAACACAAGGGGAATTTTCAAGATCACTTCCAAATCCAAAGAAGTAAAAAGAGACCTTCTAAGTT

GCTTCATTCTAAGCACCAACACTAGTATCAATCAACTAATTAATCGTGTGATATTGAGAAGGAAAAGGTA

GGGAGGTGGAATTGCACCCGATAATTGACTCTATTACTCAACCAAGAATCATATCAAATATTCACATATT

AGCTATCACCAATCTTTCAACGGTGTCATGCTTTTAACACTATACGAGAAGACCAAATACTACACAAAAT

ATAGTTAGAATTATTCAATGGTCACATCCAAAAATTACTGAGTATAATATGTTGTTTTGAAGTCTAAAAA

AACTCTAGTACCTGAGTTGATAACAACCTCCAACCTTATTTGCCCCGACATTGATAACTTAGAGTGATCA

CAAAAGCCGTGCCATATCATTTTCTGCACATGATTAAATTAATAGTCAATTTTAATTGACCATTATAAAT

TAAAGTGTCG

>Ca_linc_0095

AAAGTAATTCATTTAAATAACTCTAAATCTAACTAAATCAAACAATTTAAAAAAAAAACAAAGAAAAAAC

ATGATAAAGATGTTGTTGCCCAAGCTATAGTATATGGTGTCATAGGTAAATGGTATGATCAGATTTTGAA

TGCTATGAATCCAACTTCTTTTGGTATCTATATTTCACCTTTGACTTATGGCATGATGAAATGTAGATAT

AGCATAAAAACAACATGTTGCTTCAATTGTATATGGACTAGCTGCATCACAATTACAAAGAGACAGTGAA

GGAGTTTGTTAGAAGATACTTTGGTTATAGAGAATATTTTGTAGGAATTGTTCAATGTGTAGTTGTTGGG

TTCACATTGCTATTTGCTCTCTCATCTTTGCTATTTCTGTAAAAG

>Ca_linc_0096

GCTATTACAAGAGATATCAATGATTTCACCATTGCAAATTAATCTAAACTTCTTATTTTACATATGAGAT

CTTAATCTATGGAGCTCTACAACAATTGCATGGTATACGCATCAACTGCGGAAACCGTTAATTTAAAGAA

ATAAATTTTATTTTACTATCTAACGGTCTAAGCAGCTTTTGCATTATGCAATTAGAGATTCAAGTCCGAG

AACTTAACCTATTTTAAGCTTTTTTATGGTATATTAATTAATTTTTGTGTTGTGTTGGAACATGGTTTTG

TATTACTAAAAAAATTTAAATTAACGAGAGAAATTTAGAGAAGAAGAACATGAGATATCTCTCTAGTTAT

ACCTATCACTATATAAATTTTGTGAGAGGGAATCAGAGAGAAAATTTCATTTTTTCTTATCAATATTTCT

ATCGATAATTTTATTATTTTTATTATATCTATTTACCTGTTCCTTTTTTTTCTACTGACTAGTGACTACC

AATTATGTTTATGAGACCGTCTTTGTGGGTTGAATAACATTAACCCTAAAGTTATACCTTTTGATATAAA

TCTGAGGTTTTTTCAAGGTCTCTTTTCGTAGCTATCACTATCATAGTTTTTTAGAAGTTATTTGATCCGT

GTGAGTTCTTCTTTGTCTTTCTTTTTGTTGGAGAAGGTTATTAATTCTTTGTATTTAGTCAGTGGCTTTT

TCAATGTATTTTATGTAATATGCCATAATCGTATAGTAGAAATAAAATTTGCTTAATGCATTCATCTAC

>Ca_linc_0097

GGAGATTTCAGATTTGTGTATGTAAATTTCAAGTAGTGCTTACCGCTAGTCTAAAGAAAAAAAAAAATAG

GCTTCATGAATAATTATAAATTGCCTGGCTTGAGCCTGTGTGCCGTAAGAAGGCGAAGAAGATACAAAAT

TAAATATAAATCATGATGGAGTCATCATCATCTTCACCTTAATTCACCACCTTGTTTTCTCCCATCTCTT

GAATTGGATCCACCATAGTAGGTGGATAGTTTTGGCAAGAAAAGGATTAAGCCTTGAGATATATAGTAAG

TACATATATATTATAGGAAGAAAAAGGGGTGATCAATTTATGTAGGTGCATATAATATGAAGTATATAAT

AATAATGATGATGGTATAATGCATCACCCATTCATTTGTAACTCTTGTTCTTTGACCCAACCCTTCCAAC

ATAGCCAAACAAGTAAT

>Ca_linc_0098

CTGAAGTTTACTTTGGTGACCATTTTCATCTTTGCAATGGCTCTCTCTCTCACTTTACCATCATGTGATG

CTGCACGTGTTCCATTAGTAGGTAATTAATTAAGCATTGAAAACGTTAAATTAATAATGACCTCATTATA

TATGGTTTTGTATGATAATTAAGGAACTCGTAGATTTTGTTGTGCAGTTAATAATAGGCCTATATGTCCT

GCTTGCTTGTGTTGCACTCCACCACCCCCTGGCTCTTGCTGCACAAAGTGTTGTGCTTCGGTACCAGTTT

CATAATAGGGAAATCAAATGTCCATTTACATATAACATCACATATAGTTATTAATGATATGTTTGTAATT

TTATATGTTTAACAAATCATTATCATGATGTTATCATATATTGTATGTT

>Ca_linc_0099

GAAGTATATATTTAAAAATTAGTATATTTAACACAACTTTTACCAATGTTGTGATCATTACTCGGATATT

ATTGATAAATGTAAAAAGTTCTAAAGTGATAATGGATCAATTGAGGAAATGTTGTAATAAGAGAATGTTT

ATTGTGTTCTCGATTTATAGACTCAACACCTTTAAATTTGTCTTTGTCTATGAGTTTTAAATTTGAAAAA

AAAAACAAATTTACACCTAATTAAAGAGAAATTTCTCCATCCAATTTGAGACAAGTTTGAATAAATACTG

CAAATGCGGGTTGTATTGTGATTCCTACTCAAGTCAATATATGATTGAGTTGTATAATTAATTGTACTTT

GATCAAAAGTGGGGTGTGACAAACGAGAAATGTATCACGCACCCACCCACTTTAACTTCCCACCCCCATT

TTTATAAAAATATAATTTTATCTTTTTAACTAAACAAAAT

>Ca_linc_0100

GGCCCTAATTTGGTTTCCATATGGCCTCGCAAGCATCCCATCATAGCTTGTTCAGAGCTATGGATGAATA

CCACGGCTTATCTGTTATCACTACAGAAACAGTGTGGATTCTGTCTGTGTGCTATTTGAATTGCAAACTC

CTTTGAACAATCCAGTTGTTTTTGGTAACTACAAAAGTGAGGTAGCCCACAGTCACCATCCAGTTTTTCA

TTCTTCGACAAGACACATGGAGCTTAACTTATAATTTGTTTGTGAGAAACTCCTTAGCAAGTCTCTTATT

GTGTCTCATGTTACAGCTCTTGCTCAAGTGGCG

>Ca_linc_0101

CTGGTTTTCAAAAGTATTATTTATAAAATGGTCAATCCAACAGATTTTTCTGTTTTTAAATTTTTGAAAA

TTAAAAAACAGCTTTTATAAAGAGAATCAAACAGCCTTCCATAGCTTCCATCCAGTTTCTAGATTTTTAG

AAGCTTCTAGAAAATGGGTAGTTCTCTACACTATACATTGAAGACAAATGGACAGTATATTCAAGAAGTG

TAAACTATAATAGTTATAGGATTAGGAGAGTGAATCACTACAATTTTAAATGAAACTACATTTTAGTGTT

TAAACAAAAAAAACATTGACACCTTAATTTTGTCGAAAACTCAGTATTCATTCAAACATGGAGATAGTAC

ACTATCACACTTCAATGATCAATTAGAGATATATAATTAGCAAAAAAACAAGAGGCAGTGATCTTACCCT

TTGATGTTACAAATTCGGAAGCACAACATCATAATTAACCAACATGGCATTCACCTGCATATACATCTGC

ACCTTGCCTGGGGTCCCTCATAAATTGTAATATAATTTTTTTTTATATATAAAACAATTTTGTATGTTAC

AG

>Ca_linc_0102

CATTAGACCGTTTCATTCATTGCCATTAGGATGTGTGCCATAATCCTTGCGAGGAATATAATGATGATCA

TTATCATCAGTAACATGATTATTTGGGTCAATGAGTCCATGATCTCCTTTTGTGTCTTGCTCCTTCTTCT

GGTTTTCGGACATAATTTGGGACACTAAATTTCTTTTATGGTTTGCCATACATGAGCTTGCTAGAACCAA

CAAAAGTGCTATTGCAAGAAGCCTCATTTTGGACAACAATGTTACAACCTTACCCCTTCTTGCCTCTAGT

TAATTTAGTATAGTAG

>Ca_linc_0103

CGGAAAGATATACATATATACTTATAAAAAAAAAATAAAAAATCACTATCCTCATTTCAATATTAATAAA

TAAAAAAACTGTATGTTACTTCTAATGAATTTTCGACAATAATATAAACTTCTTGTAGTCCTACGTCATG

GTGGAGGATCCGAGAGAATTAAACTTTATGATGGATCAATTGTATTGATCTCTATAATTTTCTTCAATGT

TTCTTCATGTTACTACAACTAACCACTAACTTTTAATTTAATTTATTTTCTTTCATAAAAAATAAATTAG

CCGATCAGATGTAATCTTACATCCACGTTTAGAATATAAATTAACAAAAATAAGAAACATAAACGGCAGA

AAGGTACAAACATGGATGGATG

>Ca_linc_0104

GGAAGTCTTGGATCAATAATAACAAAGTTCTTCTCGGTACATTCGAGTGTGATAGCAAGATCAAGAACCT

TCCTCTTTTTCCTCTTTCGTGTAATTTTGTCATCAAAACTATCAGACATCTCATCTTATCGAATATCCAT

GATATCACCATGAATGTATCTTCATCTTGTTGGCGCTATAGATGAAAGCTCACTGAGGAAAAAAAAGGAA

AAAAATACTACAAAATCGAGGACAAATTAACCATAAACCTAATGGTTTTCAATCCCCAAACTCATGAAAT

GAAAAGAAAAAATAGATTGCCATTCAATCATAAATCTAGGGCTTTCTCAAGAGCAAATTGGGAAAAAAAT

TGAAAAGTTAAAAGGGAAGAAGAGAAAATACCAAAAAATGAACAAAATCAAAGCAAACTTGGATTTTTCT

G

>Ca_linc_0105

TTCAATTTCAACTATTATCACTGCATTGAAAATCAATATGCAATTTAACGACTCAATTGACTCATCCCAA

GTACTGCTAATGAAGTACAGAGAAGGCAAACAAAGGAGCAAATCTACGCAAATGGTCCCACCGAAAAGAG

GGCAGATTCAATTAAGGATAATTAAGATGATCGCCACCGCGCTTTTGTGTTCAACCTGTAGGAAGGAACA

ACGCCACAACGGTGATGCAGTTCCTTTTGAGTCTTTGAGTTCCACTTCAATTGAAAAAGGATATAGCTCA

TAAGCTTAACACTCGTTTCTTCTATGGACTCCGAAATTTTGCA

>Ca_linc_0106

CTTACACACTAAATATTAAATAGATAGATATTTTAAATTCGAATTCTCAAGTCAACAAACTAAATGTCAT

CCAATTACAATTTGAATATATTTACAAGATTAACTTTCAGCTCTGAATTTTTTCTGTCGATAACTTTTTC

CTTTTGTAGCTTACAGTTGAGAAAGGGTTACATCCTGGCATCATTGGTCAGCAAAAAATAGCAGTCAAAG

CAGCATAGAATGCATGC

>Ca_linc_0107

NAACGCTTCGGTGTTTCTCTCTTTCTCTCTCTGCAAACGTGATATCCTCTTATTGCATCACAGATGTACG

GCAACGGAAAAGTTCTTCTGTGGAACCGTGAAACGGAACGCGTCTGGGGGTGTACCACAAAGTCTAACCC

GCGAACGATAAATGCATCAAATTTTGGGGAAAATAACTCACGCTACCTTTTGGAGCGTGTAATGCACGTA

CGAATAAGATGTTGTTTGGCTTTGGTGTTGTTGGTAACGCGGCACTTGAAGGGAGAGAGTCTATTGTGGC

GCGGAATTTCGTTTGCGAAAATTTGAAATGTTTTCATAGGCGGATGTTTGGC

>Ca_linc_0108

GTCTTGATGTAAATGATTTGTTAATGAAATCAATCTTGATTACTTGAAAAATGACCATATGATCTTGCAT

GAATATGCATACTTCAATGTATCACGAATTAACTTGTTTTTGACTTTGTCTAAATAGATACACTTAAAAA

TACAAGTTAATAATTAATAAAATCAAAATCATAATTAACAAATATGATTAAATGTTTGATTATCTTCAAA

ATATTGATTAGAGATTTTGCCTCAACACTAATAATTTGACCCTATTAGAAACCAATCTCCCTATCATTTT

AGAAAACACCAAGTGAGAGATGAAGGAATGGATGTGAAGCGACTATTTTCTAGAAACCATAATGAAGAGA

GAGACCATAACACACGTGAACATATTAAATGTAACATAGACACAACTATCTTTGAAACCCCCTCATGTTT

TGGTGTGGAGATGTATTTGAGAGGAGGAGATGGAGAATTTATTCGAGCAAAAACTTCATGATTTCCAAGA

CTTCCTCCACATGTTAAACGTAACGTAGACACAACTATCTTTGAAAACCATAGATGTTTTGGCGTGGAGA

TGTATTTGAGAGGAGGAGATGGATAATTTATTAGAGCAAAAACTTCATGGTTTCAAGGACATCTTCCACC

GCATGAAGTTGAAGCTTGAGGCTTAAAAGTTGCTATAGACTGGCTTGTTGAGATAGGTATTATTAAG

>Ca_linc_0109

AAAATGAGTGTTAGAATGAAGGTTAGAAAGAGTGGATTATTAACACTCTTTTTAGAATAAGAGCTCCTCC

AAGACATTTCTAAGCATGGTGATGTTTGTGGGCAGAATTAGAAGCATAGGCTTCCACTATATGATGCACC

TTCGCAATCAAAGAAGGCAATGACGTGGCTTCACTCTCAAAATTAAAATTTGTTTGGAGTACACCAAAGA

GTCGACATGATAATGATAATGGGAACAATGATACCAAACTGAAGGACAACATCATATGCACCCGTTGCCA

ACATGTCATCATTTCAGTTTCATTCATTGGTGTATATTCTTTAGTTGTTCAATAGAGTTCACTGCTTCTT

GGTTTAATTCAGTTTAAAATTTGTTAGAAGAGCAAGAAATTAGTGACAAGCATAACCTTCCTTAAGTTAT

CTTAATAAAATTGTGGGCAGCCCGACAAAATTTGCTACGTAAGTAAATTTAAAAATGAAATACACATAAT

TTATGATTACTACTTACACATTGATTTATGCAGTTAATTTTTATTAGATGATAGGTAAATTATTCATAAT

TTTTAAAGACAACTTAAGCATCACCTTTCTATAATGTACTGTTTTAAATTTAAATTTAAGATGCATATTT

TTAAATTCTTATTAGTATCACCGCATGCAC

>Ca_linc_0110

ATTGTTAGTGGCTGCCTAGGCAACTGGACAAGATTAAATTTTGGAAGGATCGTTGGCTTAGATTCCTTAT

GGATGAAAGTGTTCATATCCTTGAGAACAAACATCACTTGCTTCAAGATACTGTGAAGGACTTCATCGGA

AATAACAATTGGTATATTCTTCATTCTATTCATTTGAATTATCTTGATATCGTTGATGTTTTGCAGATAC

TTCTGTCTTTAAGTTCAAGATGAAGAATAAGCTAGTTTGGAAGCACACCAATAATGGAGAGCTTTGTTTT

TTAGATGCTTATATCTTTCAAAAACCTTCATTTGATGAGGTTTATTGTGTTAAACTGATCAAGAGCTGCT

TTAGTTTGTCTTGTATTCTTTTTTCCTTCACCAATGTTTTATCCCTTTTGATTTTTATTGATAAAGTTTT

TAACGAG

>Ca_linc_0111

GGTTCATCCCCACTAACATTTTAAATAAGTTTGTGTCGGATAAATGTGAGGTTTTTTAGATTAGAATTTC

AATGTACCACTACAACCTCAGGTGTCGTTCTTTTATATCGGATTCCTTTAAGGTTTTTGAAAGGATTATG

TTATAATCGTCAGAAATGGGAGATATTTGATTATCATATCACCATATGATGTGGGATGTGTGTCCTTTTA

AATGTGGAATGGTGATTGTCCTCTGGTGGTCATAGTCACATTTGAATTGTCATCAAATGGCTCAAGATGC

GTAAACATGCGAGCTATCGTGGTTATCATCATCATATTATCTTTTTGTCCATTTGATAGCTATGATTAAG

GAACTAGGTGTGCTACCATATACATTGACTTCATATCTCATCATTAAACGTAATATGTCCTAATTTCGAG

AAGCTTCTTTGTTGGTCGGATCATGTCGATCTGAGGTGGTCTGATTTCTTTAATGGGTTTGGATTATAAT

GTCCTTAGTCTGACCCATAACAAGTATAAGTAACTCGTTTTTTGTAGTTGGATGTAAATAACCTTTTATT

TGGGGGTGGCGAATGGAGTTTTTAAAGTTGTGGTTGTTATTAAATTATTATAATTTGATTAAGTGCCAAA

TTTATCTAGTTTTCAATATATA

>Ca_linc_0112

CTCATATTTAACAACTATCAATTATAGTTGTATTTTTGTTTCAATTATTTAAATTGTTCAGAGTGTTACC

TATTGTAGTATAGTATGAATGACAATATGGAATGGTTAAAAGAAAAAGTTCTTATCATGATAACTTATAT

CAACATTCACACTACTAAAAATTAAGGTTTTACTTGCAGATTTTCCTGCGGCTTTAAAACAGGAACTTCA

GATTGATCATCAAATCTTCAGAAAGATCAAAGTGCCAAAACCAGATTGATAATCAATCTCCGTTTTCTGC

AGAATTCCAGCAGTGATCCTCCTACTTCTGCAAAGGTTTTTAATCATTCTTCAAACTATTTTGGCCTGAT

TCAAAGCTCCAGATTCAAAGACATTTAAACTAGAAGACAAAAAGTTTAAACAAGGCTGTAACAAACTCAG

TTAAAACAAATTCAAATCATTCAAAAGCTGAATTATTTTAATCTCATATATTGGTTTTGGTCAAAACTGA

CAGAGCACTACCAACAACTCTTTTTGACCAAGATTAAATGCTTC

>Ca_linc_0113

CAAATGTCGATATTATTACATTGAATAGTGTGAATTTAAAACCTCACATATGTGTTTTGATAATAATTAT

AACATTTTATTTATAATAAAAAATTTACTTGAGTATCTTAAAATAAACTATGTACTTATAGAACATGTGA

AATTGATCCATACATACTAAGAGAAAACAATGAAAAATAGTGTGTGAGAGAAACAAAGAGACAAACAATG

CTTAGGAAGTGCTGGTACATAATGCATGCTCTATTCTGCATCAAGCTTTCTAACCTTTGACTTGAAAGCT

TTTTAACGAGACTTTAGGAGCAATGACACGTGATATTGGCACGGCTCAATAAAAAAGAAAAACTTGGACA

TTTTAAGCATTGCTTTGTCATTGAACCACACCAATATCTCGTTCAAACTCATACCTCTATAGCTCCTCTC

TGAATTACCCTTTACCTTCTTTTGTAATACTTGCTTAACATGATCACCATCACATAATAAATTAAATTGG

CATGAAAAATAAAAAGAACACCCCCATTATTATTGAAACAACATAATAACATGAGAAAAAATTAAATAAC

CCCTTGATCTATAATCTTTATCATGCCTAGAAGAGAGTAATGAGGAAGAGATACATGCAAGAAGAAATAT

GAAATGGGGTTGATTTGCAAAAGTACTAAAGAGTTACAAAACA

>Ca_linc_0114

CTTAATATTTCCACAAAAACTGAAGTGAATTCAATAACCGAACATCATAAGTCCAAATCCAAATATCCCA

CCTCCCTAGATTTTGGACAAATTGGTCAAAGAAAAGTTTCCAATTCACTGAACACTCCTCCCACACAAAC

ATAGCCACCTCCAACTCCAACCAATTTCAAAAACACTGGTATCTTTATTCAAGAAACCTAATAAAAACGA

CGAAATAAATCCAACAATGGTGAAATATCAATCCATAAATCGCCCCAAAAATGTGCTCCCACACTGGAAC

CAAGGTAATATTTCACAATATAGCTGGCGGGTCACATACCACCTCACTCCTTGAGAATGCTTGGGAGGAC

ACTATGGAGTGATTTGGAGATCATATACTATTTGGACGTGTGGGTAGTGGTTGAGCCGCCTAAAGAGAAG

TGAATTCATTTTGAGGAGTTTGAAGTAAATTCATCAGCAAGTCTTCATATTGTATTGTCTCCAAAAGATA

ATTCTTAATTGCTATCAATGCTGATCCATCAAGTCTT

>Ca_linc_0115

CAAACATCCGTATTTTTCTTCATTCTCAATGTTTTCTAAAAATTTCATCATGTCCCAAAATATTTAAAGC

TATATATGGTGATGTTTCTGACTCCATGCAGAACTCTTCTACTTCTTCACCTTAAAAAATTCTACTAACA

AATTTGAGGGTGCTTTTTTTCCTATATAGAGTCACATACCCTTCGTACTTCGACGTCCAAGATCAATTAC

AAATATGGCCTTCCAATATATCACTCGCAATTCGTCACAATCGAGGGCAGGCCAATCTAAATTATGATAT

GATCATGCTATCGACCCAAGATATCCCTCCAATATGGAACTCTTCTTCGACTTCCTTAATTATTAATTAC

TTCGAGAGGAGAAGCTCCATCATCTTATGCAATCTCCATTTTTTAGTACATATTTTTTAGGAGGTACTAA

AATTTTATAGCAATATATATAGAGTATGGTTTAAATATTTAAATAAAATTCTATGCCAAAGTTAATGACC

TTGTCAATAATTCATAGCACAACACATTCTTGCTGCATTGGTGCAACCCAAGTATTTTACTTGGACTATT

ATGTTGTAGTGGTTCAAGAACTCTATATAAATCATGAAGGGTCACTTTTGGGATTTTCTTTTCCTTCTTC

TCTATCCATCCCTACTTTCAAATTTTGATCAGCTAGCCCACTTTTGTTTATGCAACTTTGTATCATGCTT

TATTTTCGATATAGTTTTTATTTAGGTTTGGTCCTCGTTCTCTACAGCATGATGTTCACTTACTTAAAAA

TGCAAGGAAGAACAACATGCGTGTGGTAAGATTCCTATTGCCACTCATCTAGACAACACTGTCACTTGTA

AGGATATAAATTAAGTTGATTTAGTGGTTAGTTAGGAAGTGAAGTGATAGGAAGTAGATAATTTTATCTC

TCTTTCAATAAAAT

>Ca_linc_0116

CTCAAAAAACAAATTGGAGACAAAATTATGAAAATAATGTGTTGGGTTTTAAATTACGGTTGTTTCACAA

TTTTTTATATTGTAACTTCAATTGTAGTCACAAAATTGTGATATAGAAATTGCAAGACATAAAATAGGTT

GATGTCACAATTTAAATTGTGGTCGAAGCCTTGTATGAAAATCCTAATAATGACTGAATTTGAAGTATTT

GGATTTGTGAAA

>Ca_linc_0117

CGCAATCTGATGTTGGAATTTAGAACTGCTCCGTCTTTGTGAAAATTGAGAACTTCAAGTATGTGAATGT

GAATGATTATGGGAATTGACAACACTTGAAATTCTTAGCTTAATTTCTGGATATTGGCCTTCCTTTCGTA

GGCGTTTAGAAGCAATTAATAAGGGTCAAAAAGAGTTGTTGGTAGTGCTCTATTACTTTTGACCAAAACC

AGTATATGAGAATAAAAATAATCCAGCCTTTGAATAAGTTAAAACTGATTTGACTGAGTCTGTTACAGCC

TTGCTTAATCAATTTTGTCTTCTAGTTTAAAGGCCTTTAAAGCTTCTCACTTAATCTTTGATGCTACAGC

TTCGATATGG

>Ca_linc_0118

CTCTTAATTCCAACCTGTGGGAAAAGGGATGGATCTCCCAAAATTTGTACATTGATTTGGAAGCTTTAAC

ACGGAAAACTTCTAACTAATTTCAAAAGAAATCCTCGAGGTATGTACAATATTGATGTTTGCATCGGATG

CTCCCAAACTTTTGTAAACCATTATGCATGTTCTTAGATATTGTGATATAGCACATCATCTTTGAGATAA

CTTTATTATTATGGAGGATGAACAATGGAGTTCCTTTATTAGCCTCGGGGTTTCTCAATGGGTTGACTTT

AATCTTACGTTAACTGATGTTGGTTCAATCAGAGGCCATTGGCCTCTGGTGTTTGGAATTATGGTAAACA

TGTTGTGGGTGAATAGGAACCAATTAGTTTTCTAATGAACACCACTTGATCCTAAGCAATTTTTATAAAC

TTTTAGTAAA

>Ca_linc_0119

CTAGTTTATTGGCTAATTATATAATGACCCTAACACACTTTATTAATTAATTAATTACATATGTGACCCA

TAAATCTTACAATCTCCCACTGGTCACACATGTATCCTTAGGAGTGTGTTAGACTTTATGATGTCAAAAA

TGTCATTACAATTACTTTGAGCATAATCCAAATTGTCCCGTCCATTAATCATATTAGCACATAGAACCAA

AGAGGCTTTCGTCATAATAAGCATAACTAAACCCATCAATGATCACCCGTACTGACACAACTAAATGACA

TAGACCCATCATGAAAAGTGTAGCATGAAAATTACATGAAGTTGGTCAATGCATGTTAATTTTCAACTGG

TCCTACTTTATCGAATGAGATCATACCATAACTT

>Ca_linc_0120

CCCAACTTCTCCTATTTGAATGACAACAAGACAAATAAGTCACTCAACCTCCTTTCTTTATATTTGCAGA

CTGTTAGTTGTTTCAAAAGAGTCCTTAAAATCACAAATTACAAATTGAAATAACTATGCTTCCTTCTTCA

ACCTAACAACATTTACATATATTCTTAAATTGAACCATTGATAGATAGATGAGTTTAGGGGCAGAGATAA

TGATTGGCTTTGTAGGCTTGTGGAGTGTGATTATGAGGCCAATAATGTTCCAATATGCTGTAGAGATTGG

AGAAGTGATGACATGCACCATCCATCGGATTATAAGGGAATGGCATTTTTTTGTTCTCTCAAACTATATT

TCATAGTTCATCATTAATTTCTCATCTTATTGGGAAACATCTCTTCTTCTACAAATTATTGTATGTCTTC

ATTAACCTTGGACAATTCTTGTTTATTTTATTTTATTTTTCTTTTAATTCCAAAAGTATATACCTACAAG

TTGAAAACTCTAGCCATTTTTTGTGGTGGAGATGTAACTAAATAACATTTGCTTATGAATTATGACTTAT

GTTTCCATTTGAATGTTATTTGGATGTTTTGAATAATTTACTAGCTAGTTGTTGAGCTTGAATTTCGATA

TTTGTTTTATTACCCTTGTTGTACATAAGTTCATATTTCCCTTCCGTGGAAAATTAGAAACAGAGGACAA

AATTGTTTGTTATATTAATGCATTTCCATGTTTCTTATATTCTTTGTAAAGGGAATTATTGTGTTGATAA

CTTA

>Ca_linc_0121

TAGAACATTTTAATGTTGATCAAGCAATAAAAAGCATTAAACACAGAGATGATAAAAATAATTCAATAAA

CTCATTCATATAACTAGAAATCAAATCATAAAAATAAGGGTTTCATCTTGTTACACTCATCCCTAACAAA

TAGGGTTTAGTTACTCATGACAGAAATAAAAGAGATAGAGATTAGAGAAGAATTACAAGAAGGATTCATG

AATGATTCTTGATAAAACTGCTCAAATGATGTTAGAAACGGCTGTCTTTGAGTTTCTATGCTAGGGCACA

AGTCTCTCAACTTCCCAATAGTGAAAAAGATCCCTAAAACAGTAAAAATCTGTGTTTTTATGGTTGCTGG

TGCGGGTCGCGCTCCACAGGCGCGGAAAACGCGCTCCAGACGCGTTTAGGCAGTAGCAGATGCTGGAAAA

AGCGCTTCAGACGCGCTCCAAGCGCTCATTATCTGATGTCCGGTGCATATTGCATGTTTCTTCTCTTTCG

AGTATGAATTTGGTTCCGGTGTCTTCATGAAAGTTGTAGCTATGAATCCTAGCTTTCATCTGCACTTGGT

TTGACTCCAATTGGACATCTACAACTCCAGATATGGCTGAAATACTCTACATATGTCATGTTGATTTCTC

ACCAAAATTCAGCACTGCACTAAAACAAAACGCAATGTAAAATTACGTAAAATTCCTACTTAATCAACGA

AATAAACAACACAAAACATTTTATCAACTCAAAGAACAAAAATCAACAAAATATATC

>Ca_linc_0122

GGGAAAAACATTTGACAGTTGTGTTGATCCACTGCGATAGTACCAACTGTGTTGATCCTTGCCCCTTTTT

CGTGTTTCAGTTTTGTTGGTAAAAACACTAATCACTGGTCTCACGCGTCTGTTATTACCCGTAAAAAACC

TAAGTCAAAACTGAGCACACTTTTTATGGTCAAACAAAACATGGCACACGTTCTGTGGTCAAAACCTGAA

TATCGGAGCACACACTTAATGGATCAGACCTAATAGATTGCACTTTTCTCTCTGATGT

>Ca_linc_0123

CTGGATATGATATAAGTAAATAAAACAAATTAGCTTATAATGAAGTGTAATTTGTGTGTGCAAATAATTA

AAAATACGAGATAGAAAGATCACTCACAAAAAGTATACTGGTTCACCCAACTCGGGCTAGTCAATTCCTC

ACAACCGTGATATTTTCACTATGTGCTTCATAACTAGAATGTTATATCTCACACATTTTCTCTTGATCAT

ACATTGATCAATTAAAATCTTCACATTTCAACCTAGAAAGAATTTTTACAATCACCTTTCAACTTTGAAA

TGATTTTTATAAACACACTCAATCTAACATAAAAAATACACTTGAATTACAAATGATGTATATGATAAAA

GTGTTTGAGATCTTGAAACTTTTCAACACTTATTTGAGATAAATAAAGACAAACTCAATAAATGATGATC

CACAAATATAGTGAAGATAGTTGGAGTTTGCTTGAAGAGTTTTTGACTTGTTATTACTTTCAGCTTTGAC

TTGTTATTATTTGATCAGTCTTGATACTCTTTAAATTTTGATTTTGACTATGGAAAATGATCTGTTTTAA

TTTTGTCCTTAAAAGAGAATAACCAAAATGTTGTTCGTAAAACCAAATGTTCTTGGTTTTGTGTTATATG

GAAAATGTGGATGAATGATTTAGTAGTTGTCTAGTGTGAGTCCTTTATCTCAAAAGCATGATGTACTTTC

TATAAAAAGGTGCAACATCGTTGTCCTTGCTCATAGCTCATCAATCTAAAGATTGATCTTGATGTTGTTT

GCTTCTTTGGGCATTTAACTAGACTTTTTGGGCTTGAGTTTGTTTTGAATATTCTTGACACGTTTATCTT

AAGGTCTGGACTATGGAAAATAATTCATATTACTTCTGCCTATAAATGAAATAAGTAGAATAATCTTCGT

TTTTTTAGACTTTTGTTATTAATACACCAGAATGACATTCATTTTCCAAACTTTTGTTTTTAATAAACTA

TAATGATTTTCGTTTTTCCATAAAGATAATTTTGATAAACCATAATGATCTTGTTATAAACTTCCAATTT

AGTGAAGGCATGATTTGCTTTGGTTTATACCATGGTGTAATTATTGATTGATATATGATATATTCTTTTT

GAGATTTTTTATTATTGAGTAGATACTTATGCATTTTGATGATGTTGATGACTTCTTGCTTGTTCACTTA

TATAATTCATGTTTCTTAAATAAAACTCAAGTGCAA

>Ca_linc_0124

TTTTTTTGTACATTTATCATTTCTTATAATATTTGTCATGTCTCATGGTTGCCTCTGTTATAACCACGTT

GGTTTGAAGAACTACGATCATTACCTTTTTTGATGATAATAGCATAATTTTGTGGGAACAATTTAAATCA

CCAATGATTTGTTGTAATGTGTAGTTTTGAAAACAAGTTATTTTGTATGAGTTTATGATGCATCCTCTTA

TGTTTGATCTAAGTTAAAGGAACACCAAGGCTCCACTTTTGATTTCTGTATTCTAAATATGGGGAATAGG

CTCTAAAACGCGCCTCTGATGATTATGTTATTGCAAGCAATGCTCAAATTAGTTTTGCTCTGATGACTAC

AACAAGTAATGCTCGGATTAGTTTGGCTCTGATGACTTCAATAAGCATATGAATATTTTACCTCTAATAA

GTTTGACAAGCAAGTGATTGTATGTCTCTGATAATCACAACAATCATATGATATTTGTGTCTTTGTTTAT

CGCAACTATTTGATTTTTTGACTCTAATAATAGTCAATGCTTTGAAGAAACCCTAAATATCTCTATAAAA

GAACGTGTAACTCTCATTGAAAAGTACAATCAACAACATACCACAATAAACACTATAAAGTCGTATTTGC

ACTCTTGCTCTTTACCTTTTGTCTTGTTTAAGTGCTGAGAAATATTTTTCTGAGTGCAACATTGTGAACA

CCCTCTTAAGAGAGTCATTTAGAAAACCCTTAAATGCGTTCTTTTGTAAAGTAGCCCAATAGTGGTGGCA

TACCTCAACGAGTTGATTGTACTAAGCAAGAAGGTTGAGAAGACTATAACCCATTTAATTTGATTAGTAG

GTGTTCAATGGAAGTGTAATTCAGTGTGTGAATTAGTGGGTTAAATACTACAGGTTGAAGGGACTAGACG

AAGTTACCGTGTTGATGGAGAACTAAGATAAACTAGGTTATGGCCCGCGCGCTGCGCAGATATCAATTAA

TTAATTTTATAAGTTTTATAAGTTTTATAAGTAATATTTTATGTATTATAAATATAGTTATCTTATAATA

TTACTTTATTGATTTGTAATAATTGAATATGAT

>Ca_linc_0125

CCGGGTCCTTTGATATTCCTAGCAAGTGCATCAACGTGAGAACGACTCAAAGAAAAACCATATTCTTAAT

AATAGACATGGCTGATGAATCCAAGAGTTTTGTTGTTGGTGTGCCAAATAGCAAAAATATTAATAATGGT

GAAACACAAAAAAATCATCCAAGAATGCCACCTACTTCATTTCCAATTCCTTTACCCGGAAGGAATCGTT

ATAGTAGCTACTACAATTACAATTGGGGTTGGAGACAAAGGTTCAATCAAATAGGCCACAATAAAGGCAA

CTCTAATGGGGAAAATGAGAAATCAACTTTGAAGACTTAAAAAGCTCACTCAAGGGAAGGGTAACACTAT

AGTCATGAATAATTTATGTTGCCTTGAATATATGTAGTTTACTATGTATATTGTATATATATTTTAAGCA

CTTTTATTTAGACGAGTACAAATTTGAGGCAACATATGGTGGAAAATAGACATGTGAAAAATATGTCATG

TGTTATTAGAAGAATACTTTTCTTAAAATAGATAAATAAATAAGACGTACTTGTGTTTTTTTTTCTTTCT

TTTTTGGTAGAATATGTACTTGTATTTTAAGAGCTTGATAACTTGAGAATGAGAACTTTTATTTATTGTT

GTTATGTACTAGAAATTTTCTAGTGGTCGTTAATTTGGTTTTGCACCAATATCCCTCTATGTTGCTTGAA

TGATGCTG

>Ca_linc_0126

CTATGTTGTTTTTTTTTTCCTAATATAAAATTGGTCATAAAAAATCAATGCTCCCAAATGGATAGAATTT

GTGGGTCCATTTTATTTATGATCCAGTTGAGTTTGTTGAGGCTCATTTCCATTGTCATCTCTACAATCTA

ACGCCTACCTCCTTTCTCTTTCATTCTCTCTATTTCATTGTAGAAAGATAGATGTGGCTTACACATCATG

CACGAAAGTAGCACTCAAAATGTAATAAATAGGATAGGATATTTACATATTGATGGCCACCATGCCACAT

GAATATAATAATTCATTTTTT

>Ca_linc_0127

AAAAAATTTAACTTACATTTTGCCTAGATTTTTTTTTGTCTGAGAGCTGATGACATCTATTAGTTAGGAG

TCATAAACTAATTCTTCGAGGAATTAATGCAGTAACATAAAATTTGATCGAAAAGATAGTAAAATGTGTA

CATAAGTGTCTTACTTATAGGGCTCAATTGCACAAACTCTATTTGAGTAACTAATTCCTAGCTACCTAAT

TGCTAAATACTAACCAAATTGGCAAAGTATAAAAACCTTATTACAATATATATATAAATATATAGAATTA

TACATTTTGGACATTTCCATACCTATGATTCATTCTGGAGAATCTTGATCTCCCAATGAAACTTTACTTA

GTCTACTTGGCAACAGTTTCAAATTGTTATTACTTGCAATTTTTCTTTTCCCATTTTTCCTTAGATGAGA

TCTTAATATAGCTCTCCTTGCTAGCTCTTTCCCCATATTGGATCTATTTGTGGTAGAATTTTCTTCTGAA

GAGAATCCTTGGTTGTTGTTACTAGAAGATGAGGCTTTCTCTGAAGCCATGATTTTGGTTTTGTTACATA

TATAGTATGTAGAAACAATTAGATGTTGAAGAAAAAAGGTGTAAGGTGTCAGGCTTTTAATAAGGTTGTT

TTTAGAGACATATTTCCA

>Ca_linc_0128

ATTTTAATTTTGAAATAAATATAGATGAATATATTTAAGATCAATTGTGAAACCCGATAGAGTAAATTAA

GAACACAACCCAATAAAGGAACTTTAACTTAAAATTAGTAAGTGTTCTTAAATATTGGTAATTATAGGCC

CCACAAAAAGTAACTTTAACTAAAGCATAAACATAGTTTTTAACTTTCCTACATAAAGAGGTAACTTCCA

CTCATCCACTTAAGACTTCACATTAATACAAGTGAGTACATATTTTTGGTACTCAAACTACATAATACTT

TATTAAACATACACCCTTCTTAGCTACAGAAGACCTCGATTTAAATAAACTCGCATGTCTTCGAGTTTTT

TGTACATATATAAACCATAATTAACATCAAACATAGACCAAGTTAAATTTGAAGGCAAAGTTCTTAAAAT

TTAATGAATCTACCCCAACGATTTTGGGGTAATTTTCCCTTAGGGTAACTTACAGCCGATATCCCAACAA

AGTGAATAGTTGCATCCTTGGTAGTCCCAATAATAAGGTCTTCTTTATTGCTTCTTCTTGTTGGACCATA

GAATTTGTCTATTAGGTCAAACCCTTCTAACCCAATCTTCCTTAGGTCAGCTGAAGGAGGTGCCATTGAT

TCTAATTAAAATGGGGGGGTGTATATAGCAAATGCTTCTATTCTATCAAATGTGTTTATATTGAGTGATG

ATTGATGTTGTGC

>Ca_linc_0129

TGTTGTTTTTGAAATTCGAAAACCTTTGAAAACAATTTATGCCACCGAAAATATTACTGGGATGAGTCGC

GGACTAGGTCGCTCTCTTTAAGACGTTTCGAGGCACTGCCCAAAATTGTGCAAGGCAGACTATCAACCAC

GAAGTCCCTAGGATAAAACAGCCCAGATTCACTGTATCGGAGTTCTACTGCACTGTAGAAAACCGTTCTA

GAATTACACCCTCTGTATGCCAGTCGATAGACTGGCACTCGAATATAGCACGGAGGCTACTCAAATATAG

CACGGAGGCTACTCAAGAAAAGAAGAAGAAGAAGAAGAAGAATATAAAGGGGAGAAGTGAGAAAAGCCT

>Ca_linc_0130

TAGCAAACAAAAGTTTAATTCATTAAAAATATCAACAAAAAACAATGATAGATAGACCTAACCTATTACG

ATTATACAATTATAAAAGGCGAGAAGGGACACCTTCCCCCAAACAAAGTAGTAATGCTTAAGATCTAAGG

AAATAATATTGTTAATACAACAATCGTCTTCTCTTAAGATATGAAAAATAATAAAGTGTACATTTTTAGA

TTTCTTAATACAATTTCTCCACCTATTTCTTAGCCTCCAAGGAACAATCTCTTCATTTTTTAAAATAAGA

GTCACTAATTTAGAGTCTGTTTCGAACCAAATAGATATTCAACCTTTTGAAAAAAAAAACTATCTCAATT

GCATGCATAGCTCCAATGAGTTATGAATGAAAAACAGTTGAGATGTTTTATGTTTTGAGCAAATCATCCC

ACAACGACATGTTTTGAGCAAAT

>Ca_linc_0131

CTCTTATCAACATTCTAGTATGACACAACCTAGCCCCAAATTGTTGCATTATACAAAATTCAGAAATAAA

AACCTAAAATCGCCTCATCGTAATTATAACATAACATTATACAATAATGATACTGCAAGGTCATTTCGCA

TAAGCATGAGTGAGCAATAGCTGTTGCATCTACGTTGGGGTGTAGTGAAAATAATAAGTGAAATCCCAAC

ATAGAAGTAAAGAGGGATAGGTAGATAAATACCACACGAGAATATTCATTTTGAAACTTCGAATGTACGC

ATTATTTCTGGTAAATATACGGAAATGGTGGAAACCATTATTAATAGAATTGAAACTTTTATGTGCCTCA

AGAAACTATGTTGGTAGTTAAAATGCCTAGACAAACTGAGGAAGTTATAGCAATACGTAATGGAAGAAGA

AAATTGCGACAGGTTAAAGAGACTATAGTCAATCAATAAAGCAAAAAATGTCCAATACTACTAGTGCATA

TATACCAATATGGAGGGATGGAAGAACAACACAAGATAAAGTTTTGGGAAGATCTGGAAAGTATATTTCA

AGAACATCCTTTACATGAAAAGAAAATTCCTAGGAGGGCATCTAAATGGTCATGTCAGTCGGGCATGTAG

AGGGAAATGAGCCAGTATGGTAAGGCTCTAAAAACAAGCAATTGGCTTATTTACATTCAAGAAATCAAAG

ACATTCATTCACAAAGGGGCCACATGTTCACAATTAATAGGGAAATCAAGATGGTCTTCTGCTCTGTTCT

AAAGTTATAGTCAATGTTCATTGTCTTCTCCTGATTTGTTTCCTACGGAGAGAGGGGAAAAAACACAAGC

TACGAAAATATGTATGATTGAGTTAACTGATTGATGTACATTTTTTCCCATTGTTCTTTCTTTGGTTGCT

TGCAAACACTGATTACAACCAGACATTGATTCTTGTAAAAACAAATTTAAAATTCTGGATTTCTGTTCCT

TGAATATTTAATATCCAATTGTTCTCTTTTCCATTTCTCTTCCAATTCTTTGAAAATCTTGTCAACTTTT

CCTTTGTAGAGAAGTGGCATAAGACTTGATATGGTAAGGGAAGATAGACCAGGCTAGAACAAAAAGAATA

CAATGCTA

>Ca_linc_0132

CCCACAGTAGATGAGGACGGGGAGAAAAACTCAACAAGGTGAAGAACAGGACGGGACAGTTTGGGGGTTG

GGGCTAGTGTCCACCAACGGTCAACTTTGAACCTTTATCTTATAGTCTTGGTTGAAGACAAGCTTACTGA

AACTGATACACGCTTCTTGCAAATATTATAGTCTGTTCAAAACTCGAGAAATAATAAATTTGAAGTGAGA

CTGAAATTCTGCTTATATGGTAACAAAAGAAGGTATATGCCTATATGGCAGGTGTATTTGACGAGACACA

ACCCTAGCTTAGAGGAAAAATTTGGAGAACTTATCATACAAGTCTCACATTTTAAATACCTAGGCTAAGT

TTATGAAATGATGGAGAGATAATAGAAGAAAATGTCATCACAAATCCAAAAAAGGTGGAGGAATATTTCT

AGTGTGATTTATGACAGGATGTCTTTAGCTCAGAAAAAAATTTCTACACCTACAAAAACTAAATGGATGA

AACTGTGGAATGTTTCAAGAGTTATCTTAGTGTTGAATGTACCTCTCTAGCTCAAAGAAATTTTTTTACT

ACAACAATAATACATGTAATGTTGTATGGAACCAACTTTCG

>Ca_linc_0133

CACAGGTACATGGTTCGAATAACACGGAAGGCAACAACTCACATGGGGTCAGAAGATCATGAGTAACACT

GAAGGAAGCTGTTACAAAAAGAGGCGCAGGAATACAATCATTATCATTAGTAAAAACAATTGGGGTAGCC

ATGTGTAGAGAAAACTCTTAAATGCACCTGTAAAATTTAGTAACAAACAATTTATGAGAATACTATGATC

CATGTGGCTAACCGGCCTAGTGAGGAAAAGCTTAACCATCGTCATTGTTAATACCCGTAACTCAATTTTG

CATATTTGTCCTACCTAAACAGTCCACACATTGGTCAAGAATCGAGATTAAATTCACAAACAAACCATCG

CTATTTCAAAGCTCAACCATTGAAGTACCAAGTTTTCAATGCTTTCTATTAACTAAACTATAAATGTTGA

GAGTCTCACCTTGGATTAGATATGGCCTAAAAAAGTATTTATAAGTGGAGGCATCATTCACCTTGT

>Ca_linc_0134

GGTTACGCTCGAGATGTCTACTCCCGGACTTAAGGGGGATGTCGAGAGTCCCATTCCCACATTGGATAAA

ATATGGAGGCATCTCTGACCTTATCAATGGGTTTTGTAGAATATTAAAATTGAGAAACTTCAATGCTATG

AAACTGCATAAACAGCTATCCAAAATGGCAGTGTGTAACAACTAACCCCTAAAATTGTTTAACAGGATGG

CGCATTGGAGAAGGCCAATAAAGATTGATAATTTAAATTTAGTTATATAGTTCATACATGCATAAATGTT

CGAAAGTAAAAA

>Ca_linc_0135

AAGTAGGACTCTAAAATCTAAATGACAAAGCTCTCTGAATGACACAATTCCATAATCACTAAAACTATTG

ACAAAGCCAAACAGAAACTATAAAGGCAAGGAAATTCTCACTGGTAGTGAAGTCACAATCCAACTTAGTC

TAGGAAATGATACTCTATGATAGTCACAGCAACAGAACATTTACACGAGAAATTTTGTCAGAAATGATTA

TTGTATAATGTCATACCACAATAACATTTAACAGAGATAACA

>Ca_linc_0136

AAAATGTCTCTTAGCTCACACCAAGAAGCAGAAAGTGGAATGCAGCCAAGGGTGATTTGCCGGCACTTAT

ACAGTAATTAATCGACCGTATACATATACGTACATTGATGCATAGTGTCGACAAGTTTCTCTTGGCTATA

TTTCGCTTTCTACTTCTCTTGTGTGATGCTCTTTGCGGACTCTGTTGTAACTGTCTTAGATAGTTGTAAT

TAGGATAGTTAGTTTAATTTGCTATGTACTTTTTCTGTTAATTTTGCCTTTCATCTTCATCCAAATCAGT

TTAGATGGAATGAGAAGAAGTATTATTCAGAATATGCTTAATGGTTCTCACATCAATAGTATATACCATT

TTGTGGTTAATTTAGTCTGATATATAGTAAGGTGAAGCAAGCAAGCTATAGTTTGGTTGTTATCCTTATG

GTTGATCTGTTATGGCATCTTCT

>Ca_linc_0137

TCTAATACTTTTGTCTTATGGGGAAACTAGTGGCCAATGATACTTATAATTAGGCCAATCTTCAATTTAG

ACAGGTACACATATGACTAAGAGGTCTCCTGAAGCAATAAGAGCAATTGGTATGCCGCAAGTAGCGCCGT

ACAGAAGCACCGTGTAAGATAACCACATGTCTTATGGTAGTGGACCGAATTCAAAGACTAGTTGAAGGCT

ATTAGACTAAACTGACATTATGGTTATTGTCCACTTGTGTTGAATAATACAGCAAAATACCTTCCAAGAC

TTGATGTACTAATTTCATCCTTAGTGAGTTCAAATTTACCACGACCAATTGATGGTTATGTTGGTGAGCA

GACATGTGTTACTAATCCACCCTAATGGTATAATGGTC

>Ca_linc_0138

CTCATTTGAGCTTCAACATAATAAGATAACAAACATAAATGGAGCAAGAACCATTTTACAAACTGAATGA

AATTCAAATAACAGAAATGAAAGCATATAAATAAAGAATAAGAATCTCTACAATTTATGGCTCTTCTTAT

TTTGAGAAGTAAATGGGTTTAGCGTTCAAAAAGATTGGTGAAATACAATTAAAGAAAACATGGAAAATTG

AAGACTTGAA

>Ca_linc_0139

CACATTAAAAACCACACAACTCAATTTAGTAGTATTAGGCTATTAGCTATTATATACATCTACCAAACAT

TAATATGCAACAACCCATACTCATTGAACTAGCCAATCACTTGCTTCTTGTAAACACTTTCCATACATTT

CTACAAGTAATGAGAATAAAACAAAATTAATGAAAGCTTTCATATTATAGCTTCCCAAGGTCTATTTCCA

TTGTTGGTACAAACTTCAAATTTTGACAACATATCAATCCTTTTATGGAAAACCATCATGGATTCATTCA

AGGGAGGAATCAATGTTATGAACTGATCGAGAGAAAGCTAATACATCATTGGGATCTTGAGCTCTTTCAC

CACTTGAAATTGTGTTCTCACGAATGATTATTTTGGCCATTGTGTCACTCTCTACTTCCCTTTTACACTT

ATCATCGTTGCTAATTTGGTTACTCTCTCTTTTCTTCTCTTGTTCTTTTTCTACTTGCTCTTTCCTTTCT

TTATGTGCCTCCATAAACCTTGACTCTTGTTGTTTGATTAATTACAC

>Ca_linc_0140

CAAGGTCTTTCATCATATTTATAGAACTGCCATCCAGCTGTTCTTGGACTGGCAGAGGCCATCCTCGCGC

AGGAAGATCAGCTTCCTTGAAACTTGATCTTGAGATGTCCTTTTCTATTCTCTTTCTTCAAAGAGTGTAA

GGTTGTTTCTTTGACTATGTGATCTACAACCTTTTCAGAAACTTTAAGGACTAGGTTGATTTAAAATTTT

GAGAGTGATGTTGACAAGAGAGAGAGTCACCCTCTTACATAGGACTGCCATCCTCCAATCCGAGGATCAG

ATCTGCTTTTAACATGTGAAGTCTTCCATTTTTGGCTTGTTTGTTTTTGCCTTTCTGTAACTTGACTTCT

TCATGTATTCATTCGTGCAAGCACAATAAATTGTCTGATTTTATCCTTTGCACATGTTTGATGGGTTGCT

TTCAACTTTACTGAACCATCCTCCAGCCTAGCACTGTCCTCCAGCCTAACTTCTTTATGTCCAGTGTTTT

ATTTATTCATTCTTTGATACTTTTGTGTATTTCTTTAATGAATAAAAATATTTGTTCTGGTGAGGACATG

TTTTGTTCAGATTTGAATCTTCATCCTCTTCACGAGGCCATCCTCAGGAATTTTAATCCTTCAGATTTGT

ATCTGATTTTTCTCCTTTTTGCTTTAATGATTAATAACCTGTTTTCTTAAATAAATTCACTCAAAAGCAT

AGATTAGTTATTAACCACAATCAT

>Ca_linc_0141

CTCCTCCAGTATAAGACACAGTGTTTGCCTGCACGATGTTTTCAGACAAACTTGCGGATAGTAATTTGTC

CTACTACTCTAGATGGCCTTCGAGCAATATGTACATATCCATAATGAAGATTAGCCTAATAATAAACATT

ATTCGCCATCGGTTTCCTCATAGGAGTTGGTTGAGGTAAAAATACTAGGATATAGATTTATAGGGATAAA

AGGTACTTGTTCTATAGGTTTTCCTGATCCTATCAAGGTCCTACCATTGTGGTAATCAATTGACTTTGCA

TTGTAATGTATGTCTGAGTTTAGTACCCTTGAGCTCTATAAGGGGGGAAGTGACATAATTGTCATCATTA

TTGACTTGTTTGGACTCTAACTCTCTGGCGTGTTGAACCTCGATGTGAACCATGAGTTTAACAATGTCAA

CAATTTGTTGAGATACATGATACTTGATGTCATATATCCTCATATCAATACATTGTTTAATAAATGACGT

GGACATTACCACAAGCTATTCAAGTGGATCAACTATATCATTTCCAACATAACTAAGAGGAGTCACAATG

GTTGTCATCATTCCCAGCATTGGTGGTGGATCATTAGGCCTTAGGGGGCCTAATCTCTCTAATGTCAAAG

CTGGTTGTAGGTCCATGTTGTTGAGACTCTTGTAATTGTGACATGGAAAACTGAACTTTTCAACAATTTT

TGTCATAAGGTCCCATCGGGTATGTTAAATTTGTTGTAGAAATTGAAGCTTAGCTAGGGAATCAACTAAT

AGATTATTGAAAGTTCATTAAGATATCGACCAATAACTTTCAACTATAATTCCCTTTTTTCTTCAAACAA

TTATGCCAAGAAAGAAACTTTATCCCTCGACGATAGG

>Ca_linc_0142

GGCTGTCATAAAGGGAGAATGTTGCATAGAAACAAAATATTATTTTTGGATCTGGCACAAAATCCATATG

AGGATAATCTAAGTCTGACATTGTGTGGATCTAACGCAAAATCAGGATGCATTTGATCAAAGTATTTCCA

TGTCTGACCGTTAGATAAATGTCGCAACACACTTGAATTTCTTCTATTATCATAATATCACGTGATCTTT

CTTGTAGTTTGCATTGATGCAAACATTCTTTGTGATATTG

>Ca_linc_0143

ATGAATCATTTGAATATGACCGTATTGACGCAATACCTTGTTTGACAGATGAGCATACATCTTCGGACCC

CAACGTATCCACCCTTGAAATAAAAAAACCTCTTCAAACGGCCGCTTCACTCGATGCTCCTCATATGGTG

TCCAACAGACCTCATCAACATCAATCTCGTCAAGAGTTTTCCTAAATGGAAGAACCAAACCCTTATCCCT

TTTTGGCTTCTATCTAAGTGCTCTAGGGTAGTCTTCAACATAACTAGGAGAAATGCTCCACACAATGTAG

GAAAATGCTCCACACAATGTAGGAAAATGTAGGAAAATGCTCAAAAACTCACAATGTAGGAAAATGCTCA

ACACTCACATATGCACATATATAATAACATATCATTAGTTTAG

>Ca_linc_0144

GTTATTACCTACACACACCAGCCACAAGTTGATGAACCTACACACACACCAGCCATAAGTTGATGAACCT

TCACCTAACGTAGTGGGTTCAGCTTTACCAAAACCAAATGTGAGTCATATTGTTGAACAAACATGGATTG

CTAATCCACTTTACCAATATAATACACCAATGGTTCCAAGCACTCTTGTAAGAAACTTTGTGGAACTTAA

CCAAGAGAAGCTATATGGGGAATCCTCCACCTTAGGTACAATTTCAACAAGATTTCTAGAACTCCTGAAG

CAACACATGATGCTAGCCAATTGTAGCTGAGGCTAACAATTGTTTGATCTCGACCTCATAGTCGATACTA

TCGATAGGTACTTTGGGATTCAACTGAAACCCATAGATAGACCTATGTATAAGAAGTCCTACCTTGAGTG

GGTGGACATATTGGT

>Ca_linc_0145

CTTGTTTTTATGCAAAACTAAGACTAAGCCATTTCATTAGCAATCATAAAATCAATCCAACTGACGCGTT

GATTACTAAAGGTTGTGTCCTCTCGATTGCATATGCCATTTGAGTGCTATAGATGTGTTGATTCATGGCT

TCCTATGAAACATTGGATGAAATTCGACTCTTGTTTGGAGGGACTTTTGAATCTTTCATAGTTTTATGGA

TCTTTCTTCCTCCTCTTTATACTTCATGTGTTGCTGTAAGTGGTCTTTGTGTGGAATTCTTTGTATTTGT

GACTCGTTTTCCTCATGGTTTTCAGCTTCTTCGTATTTTCATTTGTGCAACTTGGAGTGTTATTTTTTCC

AACTTGAGATGTTTAGCTATTTCTTTGCTTTGTTTCCACAATAGACATGTGAAAATGTGTGTGTGTATTT

TCGCTTGAGTGAGAACCATTTATGGTTTCATGTTCTTGTACGGTGGCACCTAAGAT

>Ca_linc_0146

GTATTTATATATTAAGATATTATCATGAAGGCGTACATTTTGAATTCGAGAGAACAGATCCGAGAGATAA

GAGGGATGAGTAGTAAACTATTCTCATTTTCATTTAGAGTTTAGAGCACTAGGTTAACCGTATAATAGGA

TAACAATTAAATAGCTAGACGGTCGAGGGCAGAGACCTCTCGTTGAGCTTAACTAAATTATTCAATCCAT

CAAAGTCTATTCATAAATAATAACATAACACATATTCATCAACTAATTAATGTTTTATAATAGTAGAC

>Ca_linc_0147

CAACATTATATTATAGTTATAATTATTATTAAAACCCAAAACCCAATACAAGAAGAATTATATATAACTG

TGTTTTTACTTTACCCTTTGTTGTTGTTGTTTCTATATAATGAATGAATCTATCTATTTTTTTTATCAGT

GTCTTCATTCCTTCCATTCTTCTATTGTAAATAGTAGGTAGGTTCTGAGCTTGATATTGGTTGTTTTTGT

TGCAGTGTTGGGTTGGGTGGGTGGTGTAAGAATCAGATAGGACAAAAGGGAAGGTGATTTGGTCGGATTG

ATTTAAGAGAGACAGAGATAGTATTAATAAAAATGGTTGAAC

>Ca_linc_0148

GACAAATAAGTCAACATAATTTACAAGCAGCGGAATAGTTTTTGAAATACGAATCCAAAGTATTTACACC

TCAAAAGTGGTACATGGAACCCATCGAAAATAAAATGTCAAGCTGACAATATTGGTGTAGGTACAATATT

CCAAATTAAAATGAACTCATCCCAAAAGAAAGACTTCTAGTCCCTATGCAACCACCTAATCTGATCATGC

TACAAAACTACACCACCCTGGTGATCTCCACGCGCCCTGCAGGATCCTCCTGACACAGCTTTGGTCAAGT

ATGACTAACTACTTTCCCATCTCCAGGGTACTAACCGGTAGGATGATCCTGGTTCTTATCTGAGGGCAAA

GCCCAGATTTCCACAATAGTTGTAAAGGTCACCAACCGAAATTAACAAACATCACATAACAAAATTCTTA

TATTTTCAAATGCTCAAAAGAACCTTTCGTCTTAGCATACTCCTCGAAAGGGTTTTCATATGTCCAAAAA

TGCATAAACAATGTTGAAAAATGAAGTTTTGACAAACTGCATTGTCGTAGCCGAATACAATTGAGAAAAG

ACAGATTTTCAATTCTAAAATGGCTCTGAATCAATCAACACTTCAAACATTCATGTTATCAACTATGAGC

ACATAATCAACACCAAATTAATCTCCACAAATTCACACCTCAATCACACATCGAATATTCATTAAATCAA

TTATGAACACATAAATACATCAAAACTTGACCAACCTTACTTCACACCTCAAAGTAATTACGACAAATCA

CAACAACAACCGATTATGTATATACAATCATCAAAGTATAACAAACATGACTCGCGTGCCAAGCACCCTA

ATGCAATGCGTATATGCCAAAATGCATCATTTCAGAATTCCAAACCAAAACCCTCATCGAGGGGCGTAAA

TCATAATTGTACAGCCTCTCACAGACCAATACTAATTACCGAGGTGCAATCTCTCACGGATCAGCACGAT

TTACTAGCGTGCAGTCTCTCACAGACCAGCACGACATACAAGAGTGCAGTCGCTCACAGACCAG

>Ca_linc_0149

AAAACTCCACATTTTTACACATAAACCACCAAATTTCATCAATTCATTTTCTACAAAATCCACAAATAAA

TCACATCTCAAAAATTCATAATAATAATTATAAACATATAAATCACACCAAACTTGAATCACCACAAACC

ACACCTCAAATTTAAATTCCACTAAATTACACATAATCACATTAAATTAATTTTTCACTAAATTACACCT

CAAATACATCAAATTTCTTTTCCACAAAATCACAATTAATGTCCTAAATGCAAACTAAAAGTTTGGAAGG

AGCCCTTACCTTAACGATAGCTCTAACACGCGATTACGGTACCGTAATTAATTCCGGTAAAATTTAAGTT

CGTGATGTCGCGTCGAAAACTAGCTCACTAGCACCGTAGCGTGGTAATGAGCAACTTTCTCTTCTGTCAC

TTCTCGATACGAAGCTTAGATCTAGGAGAAAAGTGAAGGTTTTTGTGTGTTGTGGGTTTTGAAAATAAAG

AAAATTTAGAAAACAGAGAAGGAGGAAGAAGATGGAAGGCACACGGGAAAG

>Ca_linc_0150

CACATTATCAAACAGCAAGGAAAGAAAAAGAAAATAACAGAGTTCAGTTACACTTTACAATCACTATTTT

AATGAAATGCATATATGGTCTCTACTTTTAAACTAAAACAATATTATGTTTTACAACTTTGTTGCTGTCC

TTGGTACATATTTTGATGAAAAAGTTGTGAAATTTTTTGGTGTACTCACACCATTTACTTGAATTTTTTT

GTTTGCAAGATTTACCATTTCGGATCATCTACCATTTTTTGCTTAACTTTTGAGGATAATAATCTTCAAA

GTAATTAAAAGCAAGCTCAATAAAACTAACACATTTTTCATTGTCGCTACCATAATCTAATAACACTCGT

TTGTGTAATTCCTCTAAAACAAACATAGGAATCCCTTAGTGTGATTATGTAAATCCATGAAGTAGTAAAC

ATGTATTCATTTTTCTGTTCAGATTCATTAGATTTTCTCAAAAATAGCTCCATGATGAAAACTGAATCTA

AAA

>Ca_linc_0151

ATTAGATACGGTTGTATTGAAGGTATTGCGTTAAGTTGGAAATATGGATCTTGATATAAATGGAATTGGG

CATTGAGATGATATAAGATTGTTTTGATCGTATTGAGATTGAGAATATGATGTTATGATTGAGTAAATAT

ATGTTCACTAATATGCATATAGTCTACATTGTGTTGTGATAAGTCTACTACCATGTGTATGGACGCACGA

GGTTTATTGTTGAGCTGGTTACTTGAGAGATTATGATAATATTACTAGCATGCATAAGGCTACCATGAGA

CTATCCTAGTCTGTTAGGCTAGACGACATTTAAGGTTAGATCATCCTGGTATGTTGAGGTCGTGGTTGAC

TTATAATATCTAGATTGCATGTGATATTAGCTGGTGCTTGGTCTAA

>Ca_linc_0152

TTGAAATCAAGAATAAGTAAAAGGATAAGTTAAAAGATAAGAAAAGAAACACAAACCAAATAATCTTAGT

GCCCTCAAGTGCATTCAATGGGCATCTGATGCCAGATGCCCTCAAGTGAGTTCAATGGGCATCTGATGCC

CTTAAGGCCATTGTCTTCCCATGGACCTAAAAAAGAGTTGTACTACTATGGTTCTCTCCCCTTCCAAGAA

GATCAATATGAAATTGGCCGAGATGTTTGTGTTCAGGATCACCACCAACAGAACAATTCAAAAGAGTTCA

ACCTATTTAGTCGATTTCAAGCATAAGTTGATGAGTCCCTAGTTCATTTATGGAACATTTTAATGGAAAA

GCTTCTCAAATGAAGAACCTAAATAATGTCTTGGCCCTTCATGCCTTAGTGGCAGGCTTGGATCTTGAAC

CTTTTTCTTATTCGTTGGCACGAAGGTCTTCTAAATCACTTGATGATCTCCCAACAACAAAAAAAGACCG

AAGTAGGATGATCTGATCAAGGAAAAGACTGGAGAAAGGAGTACATCACCTACTTCAGACCACTAACTAA

AAGGCCAAAATGATATTCCATAATTGTGAAGGAGTGAAGATCTAGATCGAGAAGCTGATACGAAAAAGAC

ATCTACAAAAATATGTGG

>Ca_linc_0153

GTTAAATTGTATTCAATAATACATGTCATATAGATTTTCGAATATCAATAAGAATTTTTGGGATATACAC

TACAAAAATAAAGAAATAAATTGCTTCAGGTGTGCAAGCCATCAATTCTTTATTTTTGTAGTATACAACC

AACAATTCTGGTAGATCGATATACACTACGAATTACACAACTTCAAAACATACAAAATCTTTGAAAATTG

AACATGTTAAAGCAAATTAAAAAATGATAGTAAATGTTGTTGCAGTCAATTAAGTTGCCCGCACCAAAAT

AAACAGATCGCGGGCTAATTCAATGAAACTACAACTGAATCAATTTAGCAAACGAAGAAATTGCCTCAAA

ACATTCGTGTCAAACACGGTTTAGGGACAGAGTGCGTTTGCTTAAATAAAAAACAGTTATTATTGATAAA

TAATATAGATGTTGCAGTACTAAAGGGTCTGAAATATCATAAAATATCAAATTCAGACGAGTGAGCCTGC

AAATAAATTGATATTCGCAAGTTTGAGTAAATAATTGACTGAACCATTGATTCAAAAAGGTTCAATCAAG

AAGC

>Ca_linc_0154

ATTAATCAAATGTTATGGTCATTACTAATCAATACAAGAATATTTCATTTTCAAAACTATAATAACAAAC

ACAACGAGGAATTCAAATCCATAAATCATGTGTCCAGGGAATCCTTATTATGCCTATAAATAAAGTTGTA

TTATAATATCTCATAATAAAGGCAACACAAAAAGAATCAGAATACAACCCTACAATATTTTCAAAATTCT

TAACGAACATTTAAGAAACAACTAATTAAATTGTGTTTCGTTGTGAGCGTGAGTATCACATATCAGATGT

GGCTATAAAAGCATAACCACAAGAGAAAATGTCGACGAAATCATATAACTCAATTTTGGATGTTAAATTG

TATTCAATAATACATGTCATGTAGATTTTCGAATATCATTAAGAATTTTTGGGATATACACTACAAAAAT

AAAGAAATAAATTGCTTCAGGTGTGCAAGCCATCAATTCTTTATTTTTGTAGTATACAACCAACAATTCT

AGCGGATATATGGGAGGAAATTATTCAGGGGTAGATCGATATACACTACGAATTACACAACTTCAAAACA

TACAAAATCTTTGAAAATTGAACATGTTAAAGCAAATTGAAAAATGATAGTAAATGTTGTTGCAGTCAAT

TAAGTTACCCGCACCAAAATAAACAGATCGCGGACAAATTCAATGAAACTACAACTGAATCAATTTAGCA

AACGAAGAAATTGCCTCAAAACATTCGTGTCAAACACGGTTTAGGGACAGAGTGCGTTTGCATAAATAAA

AAACAGTTATTATCGAAAAATAATATAGATGTTGCAGTACTAAAGGGTCTGAAATATCATAAAATATCAA

ATTCAGACGAGTGAGCCTGCAAATAAATTGATATTCGCAAGTATGAGTAAATAATTGACTGAACCATGGA

TTAAAAAAGGTTCAATCAAGAAGCTGACTTGCGTTTTATAAATCTACTTGAACTATAATATAGTACAATT

TTAATAAAATAAATCAAAGAAAAACAGAACTCAAACCCTAAACATAAGAAAGCAATATTAAGTGTATATG

ATTCAAAT

>Ca_linc_0155

GCTTCATGTTCATTCAACCCTTGTTGTATAAGATCGTCGATCTGCTGTAGGATAGGTGGCGGCTGTGAAC

TAGAAGTATCTGCACTGGTGGGCGGTACACAATAATGTGGTACCTAGGGTACACCATCTGGTTGCACCAA

CAGTGGATGAGACACCTGATAGAACCACTCCAAATACCCATCTTCATACTCAGATGAATATGTAGCTAGA

CGAAGCTTGTGAGATATCTCTGTCACTAGCGTCACATATCTCAACCATTCCGCATCTATATCAAATAATC

TTGAAGGATCTGGAAGTGGCGGTGATGAAATATATTTGATATATCCAAATTGTCTCAAACACATCTCTAA

CAGATATTTCACTAAAATGCTGCATAAACGGAGATGG

>Ca_linc_0156

TCTCCATATAATATCTGCATGTGTCAATGCATCAATGATACGTCTGTACTCATGAACCTTGTGCTTGTCT

TGTTTGTAGCACCATTTATTCATTCGGGCAAAACATTCCACGTTAGCACCCTGGTCACATCTATTACAAA

TATTTGAAAAATATTCATAAATCCAACACTATTGTAAATAAAAATAAATATATTAATGTTAAATAAATAA

CAATAATTAATAAATTAATATATAAATAAATGACAGATTAATA

>Ca_linc_0157

AAGGCACGAATAACTGTTTAATCGCTTGACAGAAATATTAAAAATCCAGTGAATAACGAAACGAAATCAA

AATGAACAACCTTAAAATCATCATAAAAATCAAACAAGTAACAATACAAATTTTTAGAACCAAGAGGCAG

CTTTATCTCATTGATAGCCCTTTCCTCCTAAGGGTGGCCTTTCCCTTAGGGGCGGCTCCATCTAACGGAT

GACCCTCTCCTCTCAATTCCTGCAATGCATCATTACGTATCAATTAGGGAGCTTACATCTCGTTGATAGC

CATTTCCTCCTACGAACAATATTTCTCATAGGGGCGGC

>Ca_linc_0158

GACAATTTTAATTATGAATCAGAGACACTCAAAATGAAAGACACAAAAACTATAGGCAACTAGAATTAGA

GGAAATGTGAACAAACAACTAATATCATAAATTTTACATTATTTATATAAAAATAAAATTGACATGAAAT

ATATCTGCGGAATTACGTACGAATATAACTAATGTATATTTTGCTCCGGAATTTGATGACCATTCCTGCG

GAAAGCTATCGGCAGGAAAATCCTTAAGAAAAAGATGATGATTGCTGCGAATATCCAGTCGCACATAAGT

CAGCGGCAAATGAAACGTTTACCTGCCATGTTGCCTGCGGAATACGATACCGCAGGAAACATCTCTGGGT

AAATGAGATCCGCAGGAAAATCCTATTGTGCTACCTGCGGAATTACTTCCGCAGGAACATCCGCATGTAA

ATCACTAATTTCTAGTAGTGATTGAAACTTTTGAGTAAATAGTTGTAATTGAATTTATATATGTTTTTTT

TAAGGCACCAATTGAAGTTTGTTGATTTTTACTAAAATGGAAAATTGAGTTATTAAGTAGCTGGAGCTAC

TTACTATGATGAGAGATTCAGAGTTAAAAAAGAAATATAAAGATGAAATGGTGAATAAAACGTAGGAAAA

ATTGAGGCATAAATGTAGGATTGTTTATATTGATTCTAGAGGAAGTAGACCCAAATGAAGATAAGAGGCA

ATGTCACAAAAAGCAGCGGGTTTTACGGTAGGAAAGGACAGGGGTGGCAAAAAGTTGTTTGTTTGGAGTA

GGATGAGATTCGGAATTGAACGGAGGGTAGAGATAAGATGAATTAGATCCATTCCAATTCTAAATGGTAC

AATAGAATTGTTGAGTGGATTGGAGTAGTAACACACACTTACTGTTGGATTGAGTTGTTGTTTTTGTTTG

TGTGATGCAAACTTAACCACAAAGTCTTCTTCTACAGACACAAAAGGGGTAAGTGGTCAGAACTCAAAAA

AAGCTCATAAACGCTATCTGCACAACCTTCAAAGAGAGAGAGAATGAAATTATTATTATTGTAGAGAGAG

AAAGAGAAAGAGTGCCACACATAAGTGGGCATGGCGGCGTTTGCAGTTCTGTAAAACCGTGCTCTTTTAT

TAATGCCTCTTCGTTGTCTAGAAACAAACACGCACACCATTTCATCTTTATATCTTTGTTAAGCAGTTAA

AATCCGCTGAATAAAGTTTCTTCGGATTGGATGGAGCATGGGCCTTAAAACTTGCGTGTTCCTCTTTATT

GTTATGAGCAGAGCAATTATTATCAGTGATAAAACTTTTTCCTCGTGGTGAAAACACAGTGTCT

>Ca_linc_0159

GGAAAACGCATTTGCTTTTTACTAACTTAAAAAGTATATTTGCTTGCACATGGCATATATGAATTGTCAA

GTGATTGTCAATCCACTTTCTCCTCTTTATTGTGTTCATATCTTATTTGGTGAAAGTAACTTTTCAATAA

AAAATAACCAAAAAAGGAAAATCACAACAAGCAAACAAAGATCAAGGCAATGGGTAACAACAAACGACAA

TAGATAGGTACTAAAAGATCTAAGAGGCCACAAAGACAAAATGGCTAGAAAAGTTCCAATGGTAAGTTGT

GTTGGAAAGCTCAATAGGCTAGAGAGAAGAAACAAATGCGTTTGAAATAAATTGGAGGATAAAGAAAAGG

AT

>Ca_linc_0160

AACCAAGTCTAAATCACTCAACAAAATCCATGCATGCCTCTAACGCCACTTTCAGTTCACCTCCTTTCCT

CTTAACGGACCTATCAAAGGTGATGAAATCACCTTAACCAACTCTAATAAAAGCTCCTATAAGCTATCTG

GGAAAGGAACGCCAACAACCAGACAGTTATGTGTGTATTTATTTTGGTTTGGCACTGCAGGGAAGAGGAC

ATCAAAGTGGTTCAACC

>Ca_linc_0161

TTTCTTTCTATCAAATAAAATTTCATCTATTTGTTCTCGTGATTTTAACAACTTGCAATGCTTCTTTAAT

GTTAAGTAATTAATCAAAAGAGTAGAGACCTTGCAATGATCTACGAAATGAAGGTTGGCAATCCCTTTGT

TATATGATGACGATGAAAACGTGATAAAATGCAGTGCACTGTGCTGAGAAATCATCAAGATTCGATCAGA

TCCAGGGGACGGTCAGCGTGATCTGGTTGTTTTAACACGGGCGAAGAAGACGCCCTCACCTGCTTTTCCC

ACTTACGCATCTAGCGCCTGAGAAAACAAATGAAAGGCCTAATCGCGTACACCGCATGAAC

>Ca_linc_0162

GAAAAAGGTGATTTCGTTTTTCTTCAAAGTTGGATTTCGTTTCCTTCAGCGTTGAGAAAGGTTGGTGAGA

TTTGGGGCTTTTCAGAAGTTTTAGGCTTTGGAGAAATTGAGAAACGGAGACAACACGAGACTGAGAAATG

AAGACATCAAAACGGAAAATGAAACACGATAGTGATTTGAGTTTTCTTTAAAGGGAGGCGACAGTTGAAA

AAAATCGCCCCAAGACCTGTCGCATTGTTGTAGTGAGGATGGTTATTTTATAACTGTCGCAGGCTAAGTG

TCATAATAATGAAAACTTCTTGTAGTGGTTATTAATGTTTATAGGGACGAAGCAGTTAATCCGCTTGAGC

AACAAATGGTTTTGATCACATCTTTCAATAGACAACAAGTTGACATGTGGAAAGATAACAACACGTATAT

CATCTCAC

>Ca_linc_0163

AAACGGTGAGTAGTGTAAATAAAATAATCACCCTTTTTGCAAGTTGAAGGAGCCTTGTCTCTGGTGGATG

GTTTGGAGGAAGGATGTAGAAGGTTGATGGGCACAACAGTTTGAGCCAAGTTGTCACCAGTGCTTAAAAC

CCTAAGATGGGCATGGCATGAAAGGAAGCCACATCAAGGAAGGTAATGGTCATCATACACCAAAGAAAAA

AAAATATTTAGCATCTACTGACTAGTAGCCAAGGAGACCAAAAAGGTGCTCCATGTCAAAGGTCACATCT

ATCTTTGAAAGCTTGATCATGTGGTAAATGCCCTATATTTTATAGTTCTTCTCTTTAGCCGAACTCAC

>Ca_linc_0164

ACACTTCTCAACGGAAATGCTCCTTGATAGATGGAAAAGGAAGACAAAGAAAACTCGTCGTCGGTGCTGA

AGTAGAAAAATGTTGTGTCAGACCAACTCGTCGACGACGATGAAGGCTAAAAGTTGCGTTTGATTCACGA

TTCACATTTTCACTGAAGGATGAAGGTTAAAGGTGGAAGGATGAAAACGTTTTCACGGGTAAGATGCGCG

TGTTTAATTTGTGGCTGGGTTTGATGAAGGTTGAAGGATGAAAAATAGTTTTGAATTCGCG

>Ca_linc_0165

CATCTCACCCATAACCTCTCAAAACAATTTGATAAAAAATATCCACTCTCGTTGCAACTCAATTGATAGA

TACCATTTTCGTCAACCTGGGATATTTTTAACAAAACTGTCTGAAATTAAACTCTTCGTAACATAAATAA

ATTATTCTTTGACAAAATATTCACCGTACTTAATCTAATTTATTTATCGACGAATAATTTTAATCGGGCA

TCAAAAGATATTTTTGGGTATTAAACTCACAA

>Ca_linc_0166

TACAGATTTTTCGGAGTTTTGTGTTGTTACTTTGTGAATTTTGGTGAGAAATCAACATGACCTGTGTAGA

GTATTTCAGCCATATATGGAGTTGTAGACGTCCAATTGGAGTCCTACTAAGTGCAAATGAAAGCTAAGAT

CCATAGATACAACTTTCATGAAAACACCAGAACCCAATTCAGACTCGAAAGAGGAGCAAAATGCAGAAAA

CGTCAAACAAAAGAAGTTGTGCGCTTGAAGCGCAACAGCTGCGCCTGAGGCGCATTTTCACCCTTTTAAC

ACTGTCCAGGCGCGCCCGGAGCGCAAAAACCTCTCTTGGGGCGCGTAGTGCGCTGCTGAATGTATAAAAA

CATGTGTTTCTCACGTTTTAGGGATCTTTTTTTTTTTTTTGACTATTGGGAAGTTGGGAGACTTGTGCCC

TAGCATAGAAACTCAAAGACGGCCGTTTCTAACACCATCAG

>Ca_linc_0167

GCGAGACTTCCAAAAATTTGTTTTGTGGTCATCACCCAGTTTCAAGGTAACATTATCTTGAGTTAACTAA

AGATGAGCTCAGGTACCTGCCAAATGGAGGAGGATATGTGGTACTGAATTTTGATATTTATTTCTAAGAT

CAATTGGACCATTGAGTACTTGAAGAAATGAAATTCCACAAGAACTTCAACAAACTTCCCTTATTGCAAA

TCTAGACCATGTCTTCAATGGGTTGGTAGACCTTTCTCCAAGCTATTGTCACCATCTTTTTCGAAGAGAT

ACGCTCACACCAAATAAAATTTTAAATTTAAGTATATAAAGATTTTATAAGAGCATCATGTTAGGAATAC

AAATGAAAACTGTATAAAAGCATACCATGGATAATAATGTTCGC

>Ca_linc_0168

AAAGAGACCCGTTAAATTGTACTTTCAAGGGGAGTAATCGATCGCCATTAAAATTTCATATGATTTTTAA

ACATAGATCATTAGTAAACAATTTATAAAAGTGGTCCATGTCGAATTTACCCACTCTTGTTATCTTCCAT

GTTACATGAGTATTCCCTAAACATAATAACTAGCTGTGCGTATACAAAACACTAGGGTCTTCTAATGAAG

ACATAATTTCTTAACCTCACGCATCTAACTAACATGATAGAGTTTATGCAGCGGGAGCATAGTCATAACC

ACCATCATCGTCATCGTCACCTTCCCTATATGCATCTAGAGCACAGCAAAAACCTTCAATGCATGCTGTA

GATGCATAACCATACAAATCATGATGGTCACCTTCACCGCATGCATGATTCTTGGACCTTGAAGCAATTA

CAATAGAAATGAATGTCATATTGTCTTAAAGATCTAAGTAAATGGAATTAGCGAAGAGATGTTAAAGCTA

GTTGTGTGCG

>Ca_linc_0169

CAGAAGAGTCACAATATTTTCATTAAATCTGTAAAGTGAAGATTTACAAAGAACATAAAAAAACTACATA

AAACAAAAAAAAAAATCCTAAAGGTTTGGACTAGTTAAGTGAGCAAGGATAAGAACAAAGATGTTTTAAA

TGGACTTTTGATTCTTGGTCATTCATTCCATAAATTCCTCAGTCATAGCTTCAAACCTTTCAAAGTCCTC

GTTCTTCACAAATTGAGAAGGGTCAAAGGACAATGTATAGAGAATAGTAGCTAGGCCTTCAGAGGAAGAC

ACATCAGAGCCTAAAGCAACAACAACAACAGTTGGGACTTCCAAGAAAGGAATGTCCACCCGAGGAATGA

CCACAGGTAACTAGAAACGATGCCAAGGGATCAACCATTGGGATTTCCTGGGATTAGAGCCAGAGGGAGA

CACTGCCACCCTTGAAGTTCCTTTTGATGTAGTAC

>Ca_linc_0170

CCCAAATCTCTGAAATACTTTTTGAAACAGCACACCACTTGTTCGACACAACTCCTCTCTCAAACAAAAT

GCCACCATCGTCACTCTCGGACCCACTTCAGCCCAAACACCCAACGACCGACGTGTCTCAAACTAGATAA

AATGGAAGAAAAGGTTGATACCAAGACACCCTTTCAAATAGTATGCTTTGAGATGAAGACTCTTTCTTGT

TCTTCCAAGCATGGTTTCCTTGGTTTTGGGACTTTGAGGATGCGATTTCTGGAAACTGAGACTCTAGAAT

GTTGAAATTTCAATGAATTGGACCTGAACATGTTTGATTATAAAAAGATTATGTTGTTGGGAGTAAACAG

AGTGTCTTTGGGAGTTCAAACATTTTAATTGAACTAGTGTGGACTTGTGGAAGGAACATGGTTTGAAAGA

GATTCTAGAGGCTATTGATGTATAGATTTCAGTCCTTCACTACCTCATCAGACTAATGTTTTGGTTTATG

GTTGTGTTGGTGTTATCCTTGGAGGC

>Ca_linc_0171

AAATAACATTGCTTAAAGTTGTATAAAAAATGAATAGTCATCTATTCTTAATAAAGCTAACATAATGTAC

CTACAAAACAATCATCTTTATAGCAATCTATACTTAATCAACTATATTAGTCATTTATTCTATCATTTAA

AACTTCCATCCTGTGATCAAATGCTTACTGTCTGTCCTGAGAATTGCTACTTGGAGGTAACCAACTATTA

TTGCAAGTTCCCTGCAACAGAAATTATAACCCCCATGAGAAATCACTATTTTCACTTGACATATATAATA

AAAAATAAACCAGTATAATCACATATACCTGACTTAAATAATTATACGACCTCATTCCTTGTAACAAGTC

ACTAAATGGAATTGATGGAGCATTTAAACCCCAACTGGAGGGAACTTGTTGTTATAAGAGGATATTTTTG

AGTCAAAGCAACCGAATATAATATATACAACCTCTTTATATACTTATATCAATGAAACTTGAAACAAACA

TAACCTTAAATGCATAATAAATAATCAATGGAACGTTGAATAGAATTGTATACTGTAAGTAACTTACTGT

AATAGGGGTGCAATAG

>Ca_linc_0172

CCAGTTCTCAAAGTGTTTGAAGCTAGAGGGTGTGAGTGGTCAACCATGCACAACATTAGTTTACAAGTAC

CTCTTCTTGAGAGGTTTTCCTTAACAATATGGAACCATCATTCAAATGAATCATATAAAGATTCCATCAA

GGTTAATAGTCTGCAACTAACCGATTTCTATTATGAGGGTGATCTCGAACAAGACATCATTCTAAATGAT

TTGTCATCGATTCATAATGCTTCTGTTGTGCTTGTTATTGATGAAGACAAAAAGGACAGAATAAAAAAAG

TTGGGTTTCAAACACATATGCTTCTTAGGCAAATCTGTGAAGTGGAACGACTGAAATTATTG

>Ca_linc_0173

GTAAAACCTAAACGAGTTAGAGGTCCTACATGATGTTAGATGTATTGGACATGGAAGCCGATGATTTCAT

AATTGTTAATTTGGCCAACTATCATCGATCTATTAGTGAGGAAGCAACAACTATTACTCGTTTTATAGAT

AGTGTGGCTAGGCATTAATATGCTTCCATCAATTACAAATCCTGGAAAAGGCTAAGAAAGATGAGATGCT

AGAAAAATAATAGAGGTATAGTACAAAAAGTTTCTTACATATTATGAGTAGTACATTAAATGGTTTATAA

TTTCGACTTATAATTGGTATGAATTGCATAAGGTATCCATTGTAGGGGTACAAAAAGTATCCGAAAACTA

ATTGATGAAAAGGTTTATTTTTTCAGTATTTTTTATGAGGTGAATTTCATGTATAACCTATTTGTTTGGT

TTTAGACGTTCAATATATGTATCATAATGTAGTAGTCACACTCATAACTTTTAGGCTGTCTTTG

>Ca_linc_0174

GTTATGCTAGCTGTCTTTTTCTATGTTCCTCTTATTCGATTAAAGGAAGTATCCAACAATATTTTCCCTT

TAAATTTTATGCTCTTGTGATTGTTTTTTTTTTAAGTTTTGTATAATTATATGAATATATGTAATTTTCC

AAAATTTGTTCGTCTCAGATCTAACATTTTGAATCGGATTTAATGTTTGGATTTATGATTTATGAAATCG

AAGAAATGTAAGGATATTATTATATATTAG

>Ca_linc_0175

CAGAATTAGACGTAAGGAGGTACTCTTGAGGTTTTAAAATATACTAACCCTGTGTTTCACTCATCAAAAT

TTATTGTATTCTTTAGATTTCTAGCAATTCATAACATCACAAATTTGAATGCATCATACACATTATATAT

AAAAATGCAGGATTCAAAAGGTAGTTTCACAAATATTTATTTCATGCTAAAATTTATGATTTCCCGTACC

CTCAAATTTTATGAAACTCCATAAATCATTTTAACTCTTTAATATTCATTAGAATCAACACCAATAAACA

CCATAAAAAAATAAAAAAATTTAAAGAACTCATTGCTTTATACAAAAGTTTTCTCTTCGATTCTAGTTGA

TTTGTGGTAGCTATCTACTTCCCAGTTTTCTACCTCTTCGGTTTTCGCACCTTGCTCCAACTTTTTTGAA

GTTTTTCCTTGTTGTTGCTTATCTTCTCCTCAATACGATTTGATCGATTATTGATGGTTGAGGGATGAGA

TCGTGCATGCAATCGGTCAAAGCAAGTCTGATGAGGTTTAGTAAATTAAAATTTTTGGAGATTTTGAAAA

AGAAAAAGGAATAGAGGATTTGGGGAAGATGATGAACAATACGATTTTGGGGTTTTCTCCCCGTTCTTGG

GATCGAGAAGGAAGAAATAAG

>Ca_linc_0176

AAGTAAAGCATTACAAATGAAACACAAACACAAAACATTCAGAGAAAATGAACCAAAAAAAAAAAAAAAA

CAGTTTCTCCCTCAATCTTGAAATTGGTAAAAATCAAAGTTTAAGTTAGTTGCCAAGACATCAAAGAAAT

TACCGCCTCACCCCAAAGTTCCACCTTGGTTAGGCTGTGTGGGGGTTTGAAGACCGTTGATGAGGATAAG

GTTGGTAGGGTTGAGGATACATTTGAGGCAAGGGAGGCATGACATAGTTGTGCTAATAGAAATCAAGCAC

ACTAATGTGCTATGTGTTGGTACTTCAAAACCTTTAAGCATATGTCATCCTTTCATGTTGGTCTGTCAAC

CAAGCTGATGGTACATTTATGAATAAGTATTTGTCGTGCATATAACATAACATTATCATTCGAAATCGAT

GTTCATCAGTTCGCTCATCCTGCATCTCAAATGGCGAGTAATGGTGAGGTGCGTGCGGTTGAGGAGGCTA

GGATTATTGCTCTTGTTGTTGAGTCTGCAGCGGTTGAGAGGGGCCGTCAGCAGGAACCTTGTCAAAACCT

CTTGTGCTAACTATCAAACCATCTCATGGTCATAGATTTTCGAGGAGTAAATTTACCATCATTATGGTTA

CTCTCAACCCCTTGTGACAAACAAACATATGGATAATTGAAGCATGCCTCATAACCGCT

>Ca_linc_0177

TGATTCAAATATTTGTGAGTAAACGAAACTGAAATTTTGTGAAAGAAAATGGTGCCAACTTCACTTCAAT

TGTCCGGTTAGTTTTTTTTGTTTGAACGGAATAGAAAAGAAAACGAAAAATCGTTGAAAACATTTCCCAC

GTAGTTGCAAATGCGAGGACAACAATGGCAGAAGAGAGCAACGAGAAGGGAATCCAAATTCGAACCCTAA

CTGGTGAATCCATCACTCTTCACATCACTCCCACTTCAACCGTCCAACACCTCAAGCTTCTACTCAATCA

CTCTTTNN

>Ca_linc_0178

TGTATATATACGTATAATAGAGGAGTACAATGTGTTTGAAGATCCATAGGATACCGCGTGATAATCATGA

GACCAAGTATTCAAAATACATTGATATATCAAAACACTAAATAGGAATTATTATGACAATACACCCTAGC

TATCAACAAGACAATTGTCCTCATCATGCAACACTAGCGTCTCCATTTCTAGTTGTATGCGCACTCAACA

AATCATAACAACATATGCGTCCAAACGATCCATATTTCTCCAAAAACACAAATATACTATCGGATCTAAT

TAGGGATGAAAGAAATGCAAATTATAGATGAAATTATCAAGGTTTAAGGAATGATAAGAGATA

>Ca_linc_0179

CATAAACCTACAATTGGGAGAAGACTTTCCTTATAAACTACAATGCAACAATAATCTCGACTTCCTTTAA

CACGAAGCTCATCAATTGTATGGGTACTCATATCATACCACACTTGTTCATCATCATTTTTCTCAAGAAT

ATATCACCTTTCTTTCCTGCTCCAATAGGATGTTCAACGGAAGGTAAATGCTTGACAATGAAGATATTAT

CCCACGACTCCTTCACACCAAGTTCACCCAATATTCTTATGTGAAACGTAGTTGTTTTATCATATATTAT

GAAAGCAATGGATCCATTTAACAATACCAAGTGTAAAATATCTAAACGATCAACCTTGTCTGAGGGAAAA

GGTGTTGTAAGAAACACCTCATTGCACAAGTCAAATGACACCAAACATGGCCCAACATTACTAAGAATAC

CGTAGTCATTTATACTTCACCAATGACAAACTCCATCCATGTATAGTTCATCATACGTCCTACGGTAAGG

CATGTCAACATCAAGTTTCCTCCAAGAATTACTTCTTATACTATATATCTCCCACTTCGGGGCGAAA

>Ca_linc_0180

TAATCATTTTATTCATCATCCTCCAATGATTAATCACAAGTTAATCAACCTCTGAAGAAATTCCCAGATC

GTAGTCGAAGCTATCAACATGATATGCTTGATTCATACTTTATGCACATGAGTGAATATCCCGAGTTTCT

CTCGTGTCTCCACCTCTCAACAACTGTAAATTCTACAATTGGTCCCGTTCAATGCTTGTTGCCCTTCGAT

TCAAGAAAAAATCTAGATTTGTTCTTGGAATCCCAAATTTCCCTTTTGGTAAGATCGACTCTCTATTTAT

TGGGATCGTTGTAATAGCATGGTCATGTTATGGGTGTGAGTTTGTCATGGCTCTTCACCCAGTGGTGGTC

GTGATTTTGGTGGTCGCGGTCGTGGCTACAAGATTACTCATTACAACAAACTTGGTCATATTGTAGATGT

GTGTTTCAAGAAATACGATTATGCTCCTGACTATCCAAAGCCAAACTCTATTTTTTCCAACAATTGCTCC

TCTACCATTCAAGATCATGGTGAGGATAACCTTTCCATAAGATAATTGTGATGTTGATGATGGCTCTCAC

AAGTTCACATTGAATGAATGAAAACAATGACTTTT

>Ca_linc_0181

CTAACACCACACAATTTACAGGAAAAATAGATTTTCCCACTTTCACCAAAACATCTTCCACTATCCCACG

AGAGTAAGGTATGAATCTATTAGCTAACTGTAAGGAGATGTTTGTAGGTTGTGGTTCCAGCAAGCCAAGT

TTTCTAAACACACACAAAGGCATCAAATTTATACTTGCTCCTAAATTACACAACACCTTTTCAAAGAGAG

TTACCTATTGTACAAGGGAGAGTGAAACTACTTGGATCCTTAAGCATAGCAAATTGCTTATCATGTTGCT

CATTTTTATGTCTTTTAGGAAAAGGAACACTTACCTTTACTTGGTCATCTTCCTCATTTCCTTGTGATCT

TTGGAAGCATTTCCTTCAGCCGGAGTTTCTTTTCCTTTCTCGAGTGTACACCCATCTTGCTTATGTTCTT

GATTTATCTCCTTACCACTCCTAAGAGTGATAGCCTTGCAATGCTTTCTTTTCTCTTTTAAAGATATAAG

AGGATCAAATTATAATTAACTAAAAGATTAAAAATAAAATAAGGTTAAGTTAAGTTGCAGCCAGTAGTGA

CAGTGAAGTCCACACAATTCAGTTTCAACAACAATGCTGTTGTCTCTCTCAGTTTCAGTTTCAAAGTTTT

TAACCCTAACAAGGTATGCTTTCTCTCTTTCTTCCATTCCCAATTTTCTTCCAATTTCTTCTTCAAACAC

TTTCCTTCTTTCATTCTCTTCTCATTCTCGTTTCAATTCCAACGAATTTGATGTTAATCATGCTGTTTCC

TCATTCCATCGCATGCTCTCTATGAATCCTACCCCATCCATTGTTCAATTCAAC

>Ca_linc_0182

AATGGATTCTGATTTTGAAACTTAGTAGTGATTGAAACTTATAGCCAATTGAGAACTGAACTTTGAAAGA

TGTTTGAAAATCTCAAAGAATTTCGAATATGATTGTAAATGAAGTATTGCTTGCTTTCTTGAGTTCTGAC

TTTTCCTTCATCTTGAAGTCCCTTTTATAGGCTTCAAGAGAGCTTATGACAACTCATTTTTTCGTTGGGA

GTTATCCAGCTGTCATATGGCAGTGAGTGGATAAGATCAATCATAAAGCAAGAATGTTTTTGGATTTGGG

CAGTCTTGCTTTTTGAAAATCAAGAACGATCTTCGTTTTTGCTTTGTGGCAACTAAGTTTTGACTTTAAC

TTCATATATGTGTGAAGTGTTATTCATGCTTTACAACCCATGTACAGACTGTTAAAAATTGCACGATTGT

TCTGTCCAAACTTGTCAATTTTTATGCTTCTTGTTTGCTTTAACTTTGGAGAATTATTTTGTTTTAATGA

CGTATGAATTATAGTCTTTGTCTTGCCTCTTGGAGTGATCTTCATACTGAAATCTATCCTTGTATATTTT

GGAGAACCTACTAAAACGAATCTGCAAGTTCTGAATCTAATCTGATTACTTTTTGAAAT

>Ca_linc_0183

ATGTGGGTCCAATCTGTTGGGGGGAAGAATAAAGGGAGGATCTATGGTGTTGGAGATAGATCCTCGCTTT

ATAGACCAGGTGTTGCAAGTCTAGTTCCAGATTCTCGTCCATCTAGATGTTGCGCCAATTTCTTGTCTCA

ACCATCTAACGAGATAGCTGCACAGATAGTAGCATTTGAGGAGCGAGCAAAAGTGGCAGAAAACGAGGCA

CGAGAGGCACAAGAGGAGCTAAGGAAAGTAGAGCAACGTCGACAAGAGGAAGTTCAACAAACAGAGCAAC

ACACTACAGAGCTATAGATGCAGCTAGCAACATTGGCAAAACGTGTTGCTTCCATACAGGCCGAGTCATC

TCGTCGTCGTCGTCATCCAGATTATGACGAGGATGAGTCTAGCGATGATGACGACGAGGAGTAATACTGG

TTTTAGTGTTAGTTTCGTTTAGTAGTTCTGACTACATATTTTGTTTAGACTTGTAGGAATTATCTATATA

TATTCCTACGATAATGTCGTTATGTGTATTTTGAAACTTATTGCAGTAACAATGTGCTTATTTTGAGACT

TATGGAAGTCAGGATGTGTATTTTGAAAACACTATTTACATAATATTTCACTTTGTTTTGACAATGATGT

TTTAATATGCTTACA

>Ca_linc_0184

GTTTTAACTCACATTAAATTTAAACATCAAAAGGTAAAAAAAGGGTAAAATACTAAGGAGTTCATTACAA

CAAAAATCAATGTAGAAATGAAAGATACAAAGAGGAAAATACGAGCTACAATACTGTAGGCAACAAGAAT

AAATAGTATAAGCAGTAGTACAACCTCCTTTAAGCTGTAAATCTTCAAGAATTAATGTCAATGGTTCCAA

AATGAATTGAAGATAATGATGAATTGTAGTGAGATACAATGGAATCTGAGAATTATTGTTGGTGGAGGCA

ATGGAAATTAG

>Ca_linc_0185

TTCTTCTCGGTCTACATTCTTGAACACCATTTTTCCACCAATGCTAAATTAAAGGTCCTCAAATCCCTAA

TTCTTCATTTTCTCTTGCTATACAAACCTTTTCCCATTTCACCCAATTAACTCCCCTCATTTCTTCGCTC

ATTCCCTAAAGAAATTGTTTAAAAAGATATTCAGTTTCAATATTATACCCTCTCAACAAAACAATGCGAC

CACCTATCATAAATTTTTGCTTCTCCATGACGACTTATTGTTTCTCACTGTATTGATCACTGACTTCCAT

GTCTTCTGTAGCCATGGTTTTGCACCTATTGTAAACCCAAATACACAAATGGAATTCCCCCAATTTAACA

ATTCAAAGACCTTGTTGCCTCTGAGAGCCACTCTCGTGAAACATTTTCTTATCACTCGACTTATGTGAAT

ATGACATTCAGACTTGACACCAATTGAAATTCGTCTGTTTGAGTGAAATTCTGAGATGTCACTCTTTAAA

TTAACTCGATTATTGATAGGGAAGATGCACACACTTAGCAGACATAAAAGGTAAGATTAATTGAGACTGA

GTATCAAAGTGAATTGAATATAGTGTATGTATTCACTTAGATGTGATTTTACGGAAATATTGAACACTTA

ACACATTCAATGATACTATGTCTATAAAAATATTAGCTTACTAAGTACTAACTATCACATAACTTTGAAT

ACTAAAAAGATACAACATCTAATTCTTCTCTCAG

>Ca_linc_0186

NNAGTAGGTGTTGTGAGAGTGAGTGATTGAAGGTTGACTTGGGCTGCTGTCTCTCACTCTCTCTCTAACT

TTGCTGTGAAATGAAAAATGGAGAGAGAAACACATAAGAGCGGAAGGCGACACCTTCAATACAATGCAAT

CAAAATACACACAATTGTAGTTTTGTTTTTATTTTTTATATACCAAAAAATACCAAATTAGGATTTCATT

TTTTTATTTTTAAGAGATTG

>Ca_linc_0187

GAGAATATAATTTGGGTTTCAAGAGTTAATGATGATGATGATTTTAGAGGTTCAACATGTGAAGGAGGAA

CACAAGCTGAAGTGCATCAAGAGAAAGACAAGATTTGTGAGAAGGGAAATGAAGTAATTGACAACATGGG

CAGTATTGAAATGAATGATGGTGATTGTTTGGAGAATTCTAATTTTGATTTTGGGTTGTTAGAGAGTGTA

CTTAACTCTGAATTTATATCTCATGATCTGGATTATATGGATGAACTTGCATGGAATTTTTAGACCATAC

TCTAATTCTCACTATCCATCATTTATGTATATTATCAATAACTATCCCCCCAATTAATAAGATGTGCATA

TTTTACTTTTACCTACATTTTGAAATTAACAAACAGAGGATATATA

>Ca_linc_0188

CTGGAAGATGCCACATCATGTCTCAATGTGGCTCCTATTATTCAGCTTTGTATTGTGGTGTTGTGGATTC

TTCCTAGCCTAGCCGGCAAGTTGCAAGGTCTGTCAATTTGGAAAGCAAAGTATGAAGCCATTTCCAAAGA

CAACATGGAGAGCAACAAACAAAGATAACACTCTCTCTAACAACTAGAGCCCTATTTGATTACACTTAGT

GCACTTAGCATCAGGTCATCTCCAATATCTAAATGGTGGATGGCCAAAGCGGATAAGATTTTTTTCGATT

TTCATTTTCTTTTGTC

>Ca_linc_0189

TTAGCTTATGTATTGTATGGTTTTTAATTTCTCAACTAGCCTGATTCCTAGACTGATTTGTGTAAGATAT

TGTTTATGTTTTGTTCATCTATTTTGTTTATCTTCTCCCATTATTTTTGTTTTTTCCATTACAGAAAAGG

TTTACATGGCCCCTAACTTTGAACGACATGGTGCGACGCAATTTTAAAAAACGGAGTGCAGCTAAGATGT

CGCAACTAATGCAAGTTGCGCGTAAAGATTTGGAAAATAAGCCTACTTGGATGGGAGAGAGAATGTCGCA

ACTAGGATGTTGGTAGTGATCATATTGAGTAGTATTTGTTTCTATTTTGTTAAATTGGTTGTATCATGTC

CTTGATTGTACTTAGTTTTTTTTTTTTTTTTTTAATCATGTTTTAATTAATACTTAGTTATGATTTGTGA

ATATGATATAAATTTTGTTTAGAATTATCACAATATGATTTATG

>Ca_linc_0190

ACTTTTGGTTTTCTCTGAGGTCTGCATCGACCTTTATCTGTTCATGTATCTTTTCCCATATGGTCACTGT

AACTAGGATACATTGGTAAGATTTGGCATTCAAATATGGTTGTCGCGAATTTGCACTGCCACTAAATAAC

CATCAACATCAGAGCATTAACGTTTGTGTCATACCTCAGTGTTGAAGTGGGTAATATTGTATGTTGTCCA

AGGGTACTTGCCTTTTGGTTTTCGCAATGTTGGAAATTGGATTGAGACAGCAAGATTCACAGTGGATACA

TAATAATAGGTCTAATATCTGGTTTTAATAGAATAGACTATTAATAGTTGACAGTTTCATTTCTCATGCA

TGTCTGGATTGATCAATTTAGATGTAATCTTTTGTTGGGGGAACAACCTTAATAGTTACGGACTAATGCT

CATTGGGTGGCATTTTATTATAATTTGGGATCCCTACAAAATCAATTTGTATAGTGTAGATTGTCCCACT

TATATAAACACATTTTCAAACTATAT

>Ca_linc_0191

TAGTACACGACTAACGTAGTTTAAAAAAAGATATAGAGTGAATGTTGTTAAACCAAATACAATGGTAGAC

CATCCACAATAAATCTTTTATTTCCTACTCTTCGCCCATATTTTTCATAATATGTAACATTTTTATGAGA

AATAAAGAACACGACAATTCCATAAATATTTTCTAAGCTGCAAATAATGATTTAATCACTATGAAAGTAC

TTGTGGAAGTTATCACAGCAATTACATTCTAATGTTAAAGTTTCTTTTGAATACGAGCAATATTCAAATC

ATCCAAGACCTAAAACTTAAATCTTCGAAACTTAAGAAACAGATAAACTTAACTTAAGTACCCAGATTAA

CAAATGAACAAGCACATTAGGTTAGGGTGTGAGAAGGTGGAACAGGGACTAACACCCTCACAATCCACTT

TCGTTTTGTCACAAATTTAAGGGAAAGTGAGGGAATTATATTCGGTGGATGCATTTACTCTTGGCAACCA

TCCGATCATGATTGAATGGTCGAGATCGTTTAAGGTTAATGTGAGAAATCAGGGTCAGTTTGGGTGCCTC

AACGAATCCATAAACATGAATGCAATAAAGAATAATTAAATGTAGGGCAAATTAAAAATTTTAAAATTTA

AACATGAACAGAAAAAACATGTAAAAATTAAGTCTGCCCCAAAAGTAAATTAATATTGGCCGGGTGAAAT

AGAGATCTCAAAACATAGTTTACTGAGAACCCATTGGATCGGCCGAAAACACAGTTTGAAAATCAATATG

AAATCAAAATAAATAAATAAATCTACGATAGTGACATTGAAAATTTAACGAAAGCTATGTATAAATCCTT

TCAAGGCTTCTGCAAATTTGAAAATGAATTGCAGAAATGAGAGAAAAGGAATGCAGATTTGATTTATGCC

CGCAACGGAACTCGGACGCAAATGTTTGGC

>Ca_linc_0192

TTATTAACCGACAGCGATAACACAGATAAAAGAATTGGAAAATCTTAACCTCCCATTTATGTAAGGGAAT

CATTACACAAGTGCTCTCATGCTGATAGGTATCTTATATGTACTTGTAGTTGTAGGCTCAATTGTCCAAG

GCCCAGTTGAAGTGATCCAAAAGGCAAATTAGAGAAAGGGGGAGAGTTAGTTATAAGGGGTAGGATTGAG

AGGGGATTTGTCATTCTGCATATTTTCTATTAGAACCTTTAAGGTTGGGCATAGAAAGAATTATCTCTTC

TATGAGGAGCATTTGCTCTAGGATTACAG

>Ca_linc_0193

CTTAAATTCATTTGGAGTCTACATTCACTCAAGGAGTGCCTTTCAATTGATACTAGTAGGTTGTATCATT

GATTTTTTCACTATGGAATCTTTAGAAATTATGAACGTCATCCAATCCAAACTCAATCAACAAAATGTTG

CATTCAAAGATATGCTCCAATCATCTTTGGAGTTGCATTTTGATTCCATTCACATTACACTGCACCAACA

CCTTTCATCAGTTGATTCATGTGTTTGTTAACCTATGTCCAGAGCATACATCGTCCAAATTGGGTTCAGT

CGGATAAGCATCACGCACCTTGTTTCAGATTGGAGCTTTTTTTATTTCCCCATAACACCAGAAGCTTTGC

GCCTCCAAATCACCTCATTTCATCTGGAAAATCATGTTGTTGTGTGGTTTGCCTTCCTCGAAATCTATGC

AAAACAAACTTCTTCTGTCGTATGACAACATGTCACGACCCAACTAAACCTTTTAACCCAAACACATCAA

CAACAACAATCCAACCTTATCCACAAACCTTTTAACAACCTTTACACAAACTGCCAAATCTACTACCCA

>Ca_linc_0194

CTTAAAAACACTTGAAATATCCATTTAAAAAATATTCACATGGATATACATATGACACGAATATTATTTT

TATCTAATGAGACATATAAGAGACAATTAATATTTGTACTCGTGGATACCTATCCATGGTATTCTTTCAT

GCTCTAACTCCATAGTGAAATAAAGAACAGCTACACAAATAACAATTATTGGGGATAAGGAGCGATAACA

AACACTCCAAAAATTGGATGAAAAACTCCAATAAACATTACATAAAAATAATTCAAAACTTAAAGACATC

AAGAAATACAACACATTGTCAAAAAAAAAAATCCAAGTGTTATTAATTCTCTCCTCTCCTTATTTATTTG

ATTCATTCATCCGATGATTTGAAAGGTAGCCTTCAATAAAGCATGTCTCTTTTATTATTCTCTCAACAAA

AAATTACTAAAAAATATGCAATACTTTTTTGAACAACTAGTAGTTGTTCAACAAAACAGAACTCTCCTTG

AAACAGAGGAGTTAGAGTCACATGTTGTTGTTGACGATGAAAAACATTTGTCACCACTAAAATCATTCTC

TTCTTCAACAATAGTATCCAACATTTTCTTCAATGGAATTCGTGTCACCGACACAAACCTTGGTGGAGCA

ACCTCTGTTGTGATTAATCTTGCAATTTTTTGTAACTTGTTGGCCATTTCATGAAACTCTAGTGTTGTTG

AGGTAAACAAGTGATGAAAACGTGAAGAAATACGAGACATAAAGAAAAAAGAAGAGTAGTTGTGTGTATG

CTTGTTTTTTGAGTGAGAGAAGAGATAAATATATAGGTGCTGATGAAGTTGAATTAATGTTTATCGAAAA

CAATAGGCTTAAATATTCTATCAGTATAGTTAAAAATACAAGAAATTTATTTTAGTTTCTA

>Ca_linc_0195

AGTGTTGAGCCCAACACCCTGGCAAGCCATCAAAACCAAGGTTAAATCAAAACAGCCTGGCAAACAAGTT

GACATAAAGCAAAGATATTTGAAGATATGGTATGAAAGGAACCGTAGCTGAGCAAGAAAAATCCCATTTT

AAAGCTTTGTTAACATAACAAAAAAAATGATTTCATCCAAAAAAAACAAACATTCACACAACTAAAACAA

AACTCCATCTTCCTAGTTTTAAATTTTAACTTCCAATCTAAGCAGCAAATATGAAGATAATAAGAAGATA

CATGCCTTTTCAGATACTGATGAAGACAGAGACGGTGGTTCGCGGTGGAAGTTGAAACGGCGGTGATGCT

TTCGGTTTCTTCTTCTCCTCTTTCTTTTTATGTGTTTTTTATCAGTATTAATCTCCGATTTCTCCCCCTC

AAAAAAAATGTCTCTTCTTTGTAAAAAAACAGATGTATTTATAGAGTTTTTTTTATCTGATTAGTTGTTT

TCTTG

>Ca_linc_0196

CTGCCATGATAGTATGAGGTAGACCATGTCAGTAGATGATGTCTTTCCTCACGAATTTCTTGACTTTTTC

AGTCGGGATGACTTTTAATGGCTTGACCTCTATCCTTGTAGAAAAATGATCAATATAAACAACTATAAAC

TTAAGTTGACCTAGTCCTCACAGGAATGGACCTAGTATGTCAAGTTAGAATGCGTGAAAAGGTCAATAGG

ATACTACTTGATGAAGCACTTCTAGAGGCTGATGAGCTAAATTTATGAATTTTTGGCATTTATCACATGT

TTGAACAAGTTCCATGTAATATTTCTAAATGGTACACGAATAGTAATACGTTTTGAG

>Ca_linc_0197

CACCCTTTTATCCTTCTTGTATCTCATTCGAGATCTGGTTGTTTCATCTCTTGGAATTATTCCTTAATTT

TAGTCTTCTATATTTTTTTCGATGCGATGGCGTATGTTTATATTTCTTGACCCAACCCATATACAAGTCC

TTCCATAATCCAACCCATCTAAGAACAAACCAAAAAACCAAATCAAACAATGCATGCATCAAGTATGACA

TTTGTTTGCAATGAAGCATAATCATCAAACATAAAAGCATGAGATATGCATGCCACTTTTTCTAATCCTA

ACCTCGACAATGACAAATATTATCTCACATTCTCACTCACTTGTAAATCTATTCATGCATGGAAAATAGG

TAAGGAACCCACCTCTAGGTTTTTAGAAGATGAAGAATGAAGGGTGGTTGGTTGTGGATCTAACTCTCCC

ATTGTAGTCATAGTAGAATTTATCTTCCTTATTGGTTGAGCAAATAAGCTCTTCTTCTTCTCAATCTTTC

TATCATATCCCTTTATTGTTGTAAGATTCTCTAAGATATATTTTGTATTTTGATAAATGTCAAATTTATA

TTTATCTAGTTAGA

>Ca_linc_0198

TTTTCTATCATCATCCTAACAGTTAAAAGTAGTAGGAAACTATGAATTACGTATAAACATGATTGGAATG

AGCTTATACCAAACTAAAAGGCATTATTGAAATGGTAATTTATTTTCCACTTAAGCATTTACTTACAAAT

GCAATTAATGTGCATTTCCAAGCTTATAATAAAAAAGCTTGTCAATGCTAAAAGGCCAAATTAAAAAACA

CAAAGCATACACAAATTATTGTGCACATATGCTTAATTAGAATATTGATAAGAAACATAAAAGGGAAACA

AATTAACATTAAATAACCAAAGAAAGTAATTTGCAATGGAGTTACATGTCAATCTTTCTTTTCTTCAACC

TTTTCTTCTTTTTTTTCTTCAACCTTTTCTTCTTTCTTAGCTTCTTTCACTTCCTTGACTGCCTTCACCT

TAGCCTTCCCTGCCTTTGGCTTCATGAAACAAAAGCATGAGCAGCAAGGACACTGGAACAAGAACTTCAT

TTCCAATCAAGTGCTCAGATCTCAATTTCTGAACAGAGAATGAAAATGTTCAGCTTTTCTGTCCTCTTTT

TATCTCCTTTCTTGCTTAATAATTTATATATTTAATTCTCTTTTGTCTTTTACTTTTTCCTCTATTTTTT

CTTAGTATACAATCAATTATGTATTTAAAT

>Ca_linc_0199

GTTTAGATTTTTATTTTTAAAAAACAAATCAAACTAACTTATTACACCCTAACAAAGCTTAGGACCAACA

CCAAATTAGATGTTACCTTAATCAAATAAAAACCTTATAATGTCTCTGATCTATCCCTTATTTACAAACA

GCACCATTTCTTTCCTTGGTACATTGTCCTTGCTAAATAATTCTCCTACACTACACATCAAAGAGAACCT

AAAAAGAGAGGGACCATTTAAACATGGTACCACAACAAATGCACACTTCTCAGTTCCCCTTGCATTTGAA

TTGGGCCAGTTAGGCTTTGTTTTGACTTCATGCATAACAAATATTATCA

>Ca_linc_0200

CTCATATATTTAATGCATTAAGATTTTCGGTTGAGATGTGGTGTCAAGATCTCTTATGAATCTTGAATGT

TTAGCACTCCCTCAAACTTCCCAACAAGTGGTATCAAAGTCATGATTCAGCTTGGTGGGAGATCATAGTG

CGAATCGGTTTTAGTGTTGTAGTGGAGGGAGATAGTTGGAATTCAGGTGTGTGAGTTAGTCTCACATCGA

TTAGAAATAGACGAAATGATGGATATATAAGAG

>Ca_linc_0201

ATAAAATGATTTTTTACTTTTTATTATTATATTTTTTATATATTCAAAAATTTAAATGATATTTATTGAG

GAGATAATATTTATTTGAATGTTTTTAAGTATTTATTAAATATTATTGCCAAAGATTCATAAAATAATGT

GGTCTAGCACTTGTTAAAATAAACCCTTTTTCAGGTTGATGATGATGAGAAGCGTGCGTATTTGATCAGA

AGGGTGCAGAAGAATTAATGAATAATTAAATTGTAGAGATCAAAGGGTTTTCATAGTGATTCAAAGGGGT

ATTATCTTATCTGGTTAGGCGCGGATCTCGATAGAAAATTTAATCCTACGGCTTGAATTGTGCGGCTAGG

TCGACCGAATGAAGAGAGAGGGTCGTCGTGTATTCGTTTCTTTTGATGATGACTCACTCATTCATTCTGT

TTTCTATTCATGCGTTGCGTGTTCTTTTGATAAAAAAAAAGAATAAAATGATTAAATGGAGTATTAATTA

ATTAGTGTTAATTGTAATGCAGGCGGCACCGACGAAGAAAACGCTGCGTAGAAAGAGAGACCAAATCAGA

AGCGGGCGTGTTTGGAGGTGGGCCCCAAACGAGCATTCCGTGTTGACTATGTGCCTGTCGGAGTTCATGT

TGTCTTCACGTGATGTTTCAGCGCCCCCATCTTTCTTCTGTCTACACCTTTTCCCACTCCTCTTTCTTTC

CCACTTCCAAGTGGAGTACGTATAACATTTTTATTATTACAATAATACAATTATTTTCAAAGTAAACAAT

GCGTCCTCCCTGAATGCTTTAGAATTGATGCGTATAAAACATATATTTAATCTGAAGTTAATTAAGTTGG

AG

>Ca_linc_0202

GGAATATATGAACAATTATTAGCAATTAAAACAAAAGGAAAGGAAACATTAATAGTGTCATATAGAAGTT

GTGATAAGATCGTTATCATTTTTGCATTTGTTTTTAAACCTCAAGAGAATGTGAGATGCATTTAGTGCAG

GAACCCACCTTGTTCTCGAAACAAAAAAAGCATTGCTACTAACATAACATAAGCTCGGCTACTCAAGGAA

GCTTTCCTCTTTAAGGAAGCCTTAATTCCTCTTTTCTAGGGAGATATGCCAAGTAAGCTTTTAAATCAGG

ATAAACTGCATTAAGGATTCATCTCCTTAAAAAGGAGTCTGCAATCAAAATCAGGTATAGGAACAAAGAA

GCCTTGGGCGTCATTGACTTTGAACAAAGAAGGCACATTTCCTTTTCAGTATTTAGTTGTCATCGATCCT

CTTCAGACACCACTTTATTATCCACTGACACCTACAATCCTACTTGTTTGACAATCCCCATTGGACAAGT

CAGTTTAGCTATCAACCATAACATATCATAGGAAGCTCAATCAGTGCATCCTTGGTCAGTGTAGCATCAC

CTTCAACCTCTGAATGTATTCTCGTCCATACTCCAACAAAACCACATAAAAGCAGTCCATCTTCTAAATG

ATAAGAAGAACACAAGAGACTTGTGCAGCCATAATTCAACCCCGTCAACCTCACTCTTCCTTCTCACTTC

ATCTACTATGCACAATGCACCTTAAGCCCTTTTCCCCATTCAAGCCCAATGACAATTTTCCGTCAAATCC

AACAAACAGCCTGTGAAGCCTCACTGCCTCAGAAGTTAGAACCTCCACATAAAAAAACGGGTGCTACTGA

TTTTTCCACTAAGGAGGATTTCTAACTTCCAAGTCAATAAAGCCCTAAAGACTGCCTTAACCCTGTAGAT

GTGAAGTACAGGTTCTCAAGTTATCCATTATCAAGTTTTTCCCTTTGAGTACTAATCTTGTTC

>Ca_linc_0203

CCCGGCCAAAAACAGCAAGCTAACCAGCACAACAATCAGCCAAAACAATGAGAAAACAAACATGAAATTG

AGAACAAAGTTCAGCAAAAAACCAGGAAAAACTGATGCTGTCCGAGTTCAATTTCTCCCACCTCAGACAT

CTGATCGACGATCCAAATGGTCAGAATGAAGTACCACGAGTCACGAACCTATCCGTGAACGAAGGAGAAA

TCGCGAATCTAGTGTAGCTGCTTTGTTTTATGCGAAAATAGGGTTTGCTCTTCCTCTCTCACTTTTCTCT

TTACCTTTCTCTCTGAATTCAGCTCTTACACGCCCACTAATCTGACCTCTCTCCACTTGGTTAAGTGAGA

CACATGGGCTTGCACATATTAGGCCTTTCTCCACATAGGAAGGGAGCCCAAAA

>Ca_linc_0204

AATAAAGATCAACCATGATACTTAGCATTTAGCTTTTGGAGGAGTGCCTAATGTAGACAAGAAGGAACTA

TTAAAACACTTTTAAAGACAAAGTCCCCACTGACAAGAAAGTGTGATAGAAAAGATGAAGAATAGCAAAG

GCATAATCATTTTACCCAAGATGGGGGGAGTTGGGTTTGTGACCACAAACTCCAAAAGTCCATTAATGGG

ATGAGTAAGCCTAGGTGGACCTTGTCACATGGAAATTATGTTAGTGGGGAAAGGTGATTGTTACACTTGC

ACACACACAACTATTTGTCTAAAGGCAAAGAAAAAAAAT

>Ca_linc_0205

CACACCACATCCTCATACTTTTACCCACACACCACATCAATCTTAAAGATTCACGTTAGGGGAACCATTT

AATTTTAACAGCCAACAACCACATCAAGAAGACAACCGTCGACTATCATTTCCATCAAACAAAGAGGAAT

TGTTGTATAACACGTTCAACATTCGTCATAATACACCTGAGTCCGCTACTCAGTTACTGAATAATACGTG

CCAACAAAACTCTCAAATTCCCAGCTTTGGGAATGCATATACTCTGTTGAATCAAATATCCAACTGGACT

TAAGGTGAAAAAGAAGAAGAAAAAGAAGAACAAACTCGTGATATACCAAGGCGTCGTCAAAATCCTGTAC

GTGTGAGAAAAC

>Ca_linc_0206

CTGGATTTTAAACTGATTTGGCAACTACATCAAGCAATCTGGTACAAATTTAAGGGGTGGTTGTTGATCT

GGATTTTATGGCAACTACATCAAGCAATCTGGTACAAATTTAAGGGGTGGTTGTTGATCTGGATTTTACA

GGTGTGGGTTGTTATTCTGGATTTTACACTGGTGTGGGATATGGAGACGCAACCTCACATGGAGACGCAC

TGTACACGTCAAAGCTAGCACACGGCAAACCAGAGAAATACAGCAAAGCAGGCAAACCAGA

>Ca_linc_0207

AGGAAAATCCTCAATTTTATCGTCTGATCTCCTCCTCCCCTTATTCAATTTTTGTTTCAATATATAGACC

AAAACGAGGAAGGGTTGTATCCATTGTATAAATTGATTTCTGGATTTTAAACTGATTTGGCAACTACATC

AAGCAATCTGGTACAAATTTAAGGGGTGGTTGTTGATCTGTATTTTATGGCAACTACATCAAGCAATCTG

GTACAAATTTAAGGGGTGGTGGTTGATCTGGATTTTACAGGTGTGGGTTGTTATTCTGGATTTTACACTG

GTGTGGGATATGGAGACGCAACCTCACATGGAGACGCATTGTACACGTCAAAGCTAGCACACGGCAAACC

AGAGAAATACAGCAAAGCAGGCAAACCAGAGAAATACGGAAGAAAC

>Ca_linc_0208

TCAACTTCGATCAAGTGCAGATTCAATCAATGACTTTGTCGTATTTAATATTACAACTCCTGAGGTATGT

GTGTTCCAGACACTTTGATTTGGTCATATTTGGTGGTTTTGTCACAATTATAGGCATTATGTTGTTTTAG

GCTATTAACTTGGGTGTTGCGAGTTTGTTCGCAGAATTATCTTTTTTAGCTTTAACAACATTGAATTGTA

TTATTC

>Ca_linc_0209

NNAAAATTTCATAGTTCATTGCCTATCCCTAAGGGATTGGATAGAGTAAGCATTTTGTAGAAGAAAACTG

GAATGGATTGCTGAGATCAAAACTATAGAAGGAATGGCTAGCAGCCTAGCAACACTCAGAACAAGATTTG

CAGTACCATATGAAAGATAGATGAGTCATGATGAAAAAGCCTTGTGATTAATTATTAGAGCCAAGGTTGC

CAACTTTCTTTACCACATTTCATATCATAAATATATTTATACACTAAGCTATTAAAAGTGAAAAAACCTT

GGTTTCTATGAGTTTCTCACATTGCTCATATGGTCCTTTGAGGTTTTTAGAATTGAGCATGAAGCTGGTT

GACATTGATATGTGGCCTATTGTATCATTGTTCTTATATTAGGATTTAGAAAATGCTATTTTCAATGGTT

ACTAAGGATTACTTTTGGTCCTTTGTACATACATGCCTAGTAATAAAAGAATTGTCATTTGGCTTCAAAT

TTGGTCATCAGCATTCATTTTAGAGTTATGTTCTAAATTCTAATCCTTTGGATTTGAAGAGTTCTTTATA

TTTAGAGAAACTTTGAACTTCTGTATAATATAATTTTCTTATTTCATTTTTCCTTTATTTAGTTATTCTG

TTGTTGGTGGATTCAAAGTCAGAAAATGA

>Ca_linc_0210

AGAAACCCTAAAAATAAAAAATCCCAAAAGAAGCCAAAAATAGAAACCCTAATAATAAAAAATCCCAAAC

GGAGAAGCAAAAATAGAAACCCTAAATCAATAACGAAAAACCCTAATGATATGGAAAAGTAAACTTACAA

TGACGAAGATACAGAAACGGCAGAAGATCGGCATAATCGAAGAAGATCGGCAAAGACGAAGATACAGAAA

CGGCGGAAGATCGGCAAAGACGAAGATACAGAAACGGCGGGCAGAAGATCGGCAGAATCAAAGAAGATAG

GCAAACGAAGAAAACGGCAGAAGAAGAGAAGAAGATGGAA

>Ca_linc_0211

CCAACTGCTGTCACCTCATCAATGGGCTTAGGTACTTGAGCATTAGCATTTTGCACCTTGATAACCACAA

GCCTCACCATATTATTATTATTTAAAATTTGATTATGTATAGTGGGTCCCAACTTATACATATCAACATA

TGCCACAATATGAGTATGCCTATCAATATATAATTCACAATGTTCTAGGATATTCCTCTTCATCTAGTAT

AGGTGGCACACCAAAACAACTACCTTTTTTTGCTTGCATGTAAAGTGGTATTAGGAGATTATTATTTTGT

TAAGAAAAATCCATAGATGATATATTTTGCATCGCTTGTTCTCTAATCACCTTAGTAAGCTTTGCTTTTA

TGGTCTCTATTTGGTTGCTCATAATAGGCGAAGTAGAGGATGGACGAGAATTCGGTCCAAAGTATTGTTG

AATCACCACACCTCCGCCAATTGCCTTAACACAACCAAAGTGTTTAGGTCGCCCAATGGCTTCCATCAAG

ATATCTTGACGTCTTCTGGATGGAAGTTTTTTTTTTG

>Ca_linc_0212

CGAGCTCTCTTCCACCTTTCATGGCGTGATGGTGGAGATGGAGGTGGAGCTTCTGAATCTCCAACTGATT

TTTGCCTTTGTTTTAATTTCTCTTAAATCATCCACTTGTCTAGCGATGCATATCCCCCACGAGACAATAT

GTGGAGGTACTTTTTAAAAGACTGAGTCATTTGTGCCTTCATTCTTTTCTCCTATTGTAAACAACAAGCA

AATTAAAGATATTACTAAAATATTAAATAGAGCAACCAAATCATATATACAACTAACTTGA

>Ca_linc_0213

GGATTATGTGTTATGCTAATAACAGATTTATTATCACAATAGAGTTTCATTGGTTCATCCCACTTTATCT

TCAGATTCTCCAAAATGATCTTCAACCATAGTAACTCGCATATCCAAGAATCCTTCCACGAACTTCATCA

AGGTCCTTGTTTAGTCCTAATAAGAATTTGTAAATCCTATCTTTTTCCACCAGTTCCTTATACTTCTTCT

GATCTTATGTACAGCACCATGTAACCTCTTCATATATGCAGTTGCTGCCAGTATCTGTTAAGTCTGTTAA

AATACTCGGTAACAGAGGATTCTCCCTGTTGAAGGTCATGAAGAATGCTTT

>Ca_linc_0214

AGAACATGCCTTTTTGTCAAAATACTTGAGAATATTATCACGTGTATATGAATCTGAGTGCATTTTAGGT

TTCTATATATTTCATATCACAATCATGTTTCATTGGAAACTGACAATGGTTTGGTTATGACTTTCTTCTT

TATTTGTTAATTAATGTTTTTTATCCACACCCACGGTAGTTGCAAGCATCATTGCTTTCACTGTGGTAGT

ACGTTGCTATTGTTTATTATTTTGAATTCATATTGATGATTCCACACTATACTAGTATATGATGATCTGA

TGGAACTGTACTTGTATTAGTTACACTAAAACCAGATGAGTAACAAAGTCGTATGTACTTTTGTTGT

>Ca_linc_0215

AAAGTGGGAACAAGAATAAAAAGAATAACAGGCAAGGCATATATTCAAACAGGGCATAAAGTCATAGAAG

ATAACAACATAGAAGTCGAGGCATATATTAATAGCATAAAAAGAAGAAAAAAAACATTGATCTAATTCTC

CACATCATCATCATCATTCTCGTCTCCCTCTTCCACTTCCTCATTGTGCCTCGTGGGGATGTCCATGATG

ATATCTATGTCCTTCATCAACTACTCCAAGTTGTCTACTTTGTTGCTTGGCCCATGTAGTGATCATTTAG

TGTTGGTGGTGATGGTTGCAGTGTGGTTGTTTAAAGTCGCAGTGTGACAATCAGTGGTTTCGTGTTATTT

CTTATTTCAGAAAGGATTGTTCGGAGCACTTCCATCATCTCAAAGTAGTTGTTGAGTGAAGGAATAGGGG

GAAGGTCTGAAAACTGAAAAGAAGTCTGCTGTAGGGGTGATGGTTCTGCCTTAGGAATACCTTCGACTAC

AATATGCTTCCTTCTTTACAGCTTGTGAAAGCATTGGCCATTCATATAGATCAACTTATTTTGGTTCAGA

GTCCTTCCATTGAACCATGATGAAAACTTAGATTTGGGATACCTCATCTTAACAGATAAACCGACACCAT

TATATACCGATGACCGAGTGACTAGATTTCTGAAAGATAGTGTTTGATGGTCTTCATGTCATACAATGGG

CCACATGATGAAGAATGCCTAGTTCAAAGTCAACCCGTATAGACTTACAGATTGTATAATGCAGTAACCG

GTGCTTGATTTTCAAAATGCAAGCAGTGATATCTTTGAGTCTCAAATTAGCAAGATCAATCAACAGAGAT

TTTGTAGTTTTGTGGGCAGCATAGTCCTCAACACTTACATAAAGACCTTTCGAGAGCTGCCCCAAAATCT

GGCTGAAGTCAGTGACAGAGACAAAGATAGGTTTGTGACAAACTTCAGAGGACAAAATGCCATCAGTGGA

TCGAAGGTTGCAGTAGAAACTTCAAGTTAATTTTGAATAAATGTCATCTGCTATAGGAAGTTGGTGATTC

CAATATTATCCATTAGAGGTAGGAAACTAAAATTTTCACTTCTAAAAATGTCCAAGTCCAAAAATTTGGG

TTCTTGGATGTCCCTTTCTCTGTAGAATTTTTCGAAAGTTTCCAATAAAAGAGTGTTGGAAAAGAATTTG

GATTGAGCAATAGTCAGTGGTTACTCATTGTTCTGTACTCTTTGTTTTGGGAGCCATTTGAGATGAGAGA

TGAAGATTGATAATAGTGGAGTATAAGGATGATGATTGTGAAGAAAGAAGGGAA

>Ca_linc_0216

TTTGAGTCTACCTCTGATCATGATGTTTCTGATTTTGCCTTTGATCATACTTCCTCTGAGTCTGCCTCTG

GTCATGTTTCCTCTAACTCTTCATCTGATCATGTTTCCTTAGACTCTGCCTATTGTCATGTTTCCTCTAA

CTATGCCTATGGTCATGTTTCCTCAGATTATGCTACCTCTTGTTAGAGTTCGTGTATGGTCATGCTACCT

CTGATCTAAGCTTCATTTGACTAGTTTTTCTATGGTCGTGTTTGCCTATGGTCCAAGCTTCCTGATTCGA

ACGTAAAATTTAAAGTGCCAATTTTCAATTAAATCAATTATTATCTAAGGTTTTGTGGTTAGAATTGATT

GATGATTAAGAT

>Ca_linc_0217

ATGTTGCATGAGTTACTTTTGCACCAGTTTTAAGCCTTTGGTCCCGGTAGTCTTTTGCAGCAATGAGGGC

AGACCCAGTGTAGTCCCTTGCTATTACTCTCCATCATGTCTTACCATTTAGGAAGCTGCCAACATTTACG

TGGATCTTTGATTGCCCACTGATGGAGGAGACTCCATCATGTCTTACTATTTAGGAAGCAGCCAACATCT

ACTTTGTGTTCTTGGGAGTTGTATTAACTCTCTCTTAAACTTGCAACAAATAGTG

>Ca_linc_0218

GTTGTTTCTTTGAAGATTTGAAATTTGAACTAATGAGTGTTTTTTGTGATCTGGGTATTGCTTCGTTTTT

TTGGATCTGTTGTGATTTATAATTTAGAAGACGGTGTATTAGTAAAAATGTATTTTGATTGATCCACGTG

ATTTTTTCTGATGAAACAAGTGAAATAATTAGATCTGTATTTGATTTGTTATTTTGAATCTAGGGTTGTT

TTCTGTGTGAA

>Ca_linc_0219

AAAAGATAGAAAAAAAAAAGAAACATCTACAAAATGTAGAACTTTGTCTGTATCACACCACCACACTTCT

ATTCTCTAGTCTTCAATAATCCTAATAATACAACCCATAATATTTGAGATAGATGTAAAGAAACTAAAGA

ATAACAATTAGAAGAAAGGGAAAAGGAAAAATTTGTAACCACCACCCACTGTATCAACCAACGTGGAATC

AGCTAGAGCCACCACCATCTTTGACCAACCGTGTACACCTTCGTCAAACCTAGCCATCGCCGATCACCTC

AAGCTCCGCTCTTGTTGTCGTGCAACAGTCATTGCCTTCACCAAGTTACCGCCCACGACCTCCATGGCTA

CACCCCAATTGGTTTCTTTCCTCATTTTATCTTCTTTTACTTCACTTTCTTTAAAAGCCA

>Ca_linc_0220

GTTAATAGTGTAAACTTTCTCATGCAATTGTCAATTGTGAGTGCAAAAGAGGCACAACAACACTGCCACA

AAATGAATCCTTTCACAACTTGGCCCAAGTTCTTTTCATATATGTTCTCTCTCCTACCAACCAAAGTAAC

TTTAATAAGTGTTTGGACAATGGAGAGGAACATCCCGGGCCTACACCACAGATAAGACACAATTTTCACT

GGCCAACAAATGCAAAAATAAAAAAGATACTATATGCAAGGAAATTTGCAAAATAATAATTAAAAAGAAC

CGAAAAGGAAAAAGTATGGAGCTTCTGAGTAATGGTAAAGTAAGGTGTGTTGTGTATTCTCACGTATTCG

TTGGTAAAGACCAAGCAACTGATAAAAATAAAGGTTTTTTCAAAAATAAAAATATTTGATAAAAAAAGGT

TCATTCCATTACCAACGGTTGGATGCAATAGCATGATAAAAAAAAGAAGGTTCATTCCATGTTTGTGTTA

AC

>Ca_linc_0221

GTCCTTGATCATGGAATCATTAGAGAAGACTTTTGATGATATATATACAATATTATCATATTATATTACA

TCTATTTAGCACATGACATGAGAAACATTAACCCCATGTCACTAATATATAATATGAAAAAGAAATTAAG

CTAATGTTAGAACATAACTTTTTTTTTTAAATGCAAGAACAGAACTTTAGATTACAAAGATTGCATATTT

TGCTATGATGTATAAGAGTATGAATAATTAATATTGAAGAAATGACTAGTGTCCAGCACCCGGGCTAGGA

TTAGAAGATACAAGAATTCTTTCATTGTTGGCAAGATGAGAAACAAGCTTTCCTCTTCCATGAATATTGA

AATTATTAATAACCTTTCCTAAAGTTAGGGTACCCTTTAAAAAGGTCATCTTTTGTGTCTCCATTTTGAG

AATCTTTCGGGCATCTATAGATGAATACATTACGAAGAAAAGTAGAGTGATGAGTAAAATGGAATTCAAG

CGAGCCATGTGTATAGTGAACACTAAAAGAAAAGGCAAAGGTAGGTACTAATGTGTTTGTGTATTTGCTA

TATGTGTTGAAAAGAAGAGTGGCTTATGTGCTATTTATAGGATTTGAAGTTTCATGTTACTTTTCTCATG

TGTTTTTGTT

>Ca_linc_0222

ATAGTGCTGACAACATAAACAAATGCATGTGTGTCACTTACCACTTTTTATGAGTACAGAAGCAAGTAAC

AAACTTTCCTATCTTTGGGTTAACAACTCAACACCTTTCAAATGATGCTCCACATTTCAAAACTTCTATG

GTAAGGAAAAGAATATTGTTTTCACTTTGATCATGGATAAGAGGCAACAATCTAGAGAGACACACGATCA

TGCACTCAGGTGATCTCAATCGTTCGGGTATAATAATTTTTAGATTTAATATGTGTACATTGAAATTGTG

TTTATTTAACTCTTTATATCTCAAACTATAGTTTTAGGATATGTTTGTGTTGGCATTGATTCTTGTTATA

TAAGTGATAATGTTATGAGATTTCATTTGACATGTTTGGTTTTGATGCATATTAACTATTTTAGTAGTAT

AATTCATAGAGAGATTGAATTTGGTTATTATATGAAAATTTTAATTTGATGAATATATAATATCAAGAGG

ATTATTTGGTCGTCAATATAACTTCTTGTTGGTTGTGGTATTTGTTCCTCTTCCTTTTTTCATATTGGTG

TTAAGATTTGGTATATTTTTCTACTTTCTTTGAATATTTACAATTGTACTTGGCACATTAATATGACTTT

ATATTATGCTTCTTTCTATTTGGAAAATTTTAATATGGTTGTAAGATTTTTGAATTTGGATGACTTTTTC

TAGAACACACGTCTGTTCAAAATTTCAAACATAATTTTGATCTATTTTGGGGAATATAGAACTAGTAATA

ATTGAACATTTCCCAAAAGTACTAATTCAATTTGTACAAAAAGTCAACAATCTGACAAAGTTTCATTCAA

TGTACACTACTTTATTCTTCATACATTTCAAAAACTTTCATTTGGAACCATCAATTTCACATTTTGAGAG

AACATAGCAATCAACTTAGACTTATTAGCCAATCAGTATTCTGTGCTGCTCTATTGGATATTTTGAATTT

TCTGATTTTGCTTTTGGCTTTTTATGTATCAATCACTGTTTGAATTTCATGTTTCAACACTGTGTTTGTT

TCTTGAGCTTTGAATGATCATAAAACATAGAGATAGACATTAACATATAGTTAAAATTAATAATTTGAAA

TGATTTTGAAAATAACTGCATGTGTCGTTGTCAAACACAGACACTTGTTAGACATCAGATACAACTTTCA

TATGAAATGTCAATGTCGATGCTATGTAACATTATAGAATCCAAATTTGTTTTGTAATATTTTGTATTGA

TGAGTTTCCTTAGCCATACACAGATCCTTAAAGATCTCCTTTTGTAACTCAAATGGAAAATTTTCCAATT

TAACAAATTATATATCATGATAGGAATAGTTGATGTTGTATTCTGAGACTCAAAAAAAAGGGGACAACTA

TGATACTTAGTGAACAATCAACAAAGAGAAAGGTCATTCCTTAATCATTTGGTTCGATGATCCATCACTA

GTCAATGTGTATCAGTAAATATATGTGACAGATGTTAACTCGTTACGTAGATCTTAATTATCATAAGAAA

TTAATTAGTCTCTACAATTATATGCATAAATACTATTACAATTCTTGATTTAAATTATTGATAAAAGAGA

AATAGATAAAAATAAATTGAATAATATAGATAATAGATAGAGTTGTTTACATTAAATAATATATAGACTT

ATGTAGTCAATTATATAAATATATAATTAGTGTTAAATAACTAACTAGATATAGCCAACAAATTAATTTA

TCTAAGTAACCAATATTGGATCAAAATTTCTGCTATCTTCTTAATAATGTGTATTGTGAATGCCAAATTG

GATCTAGCTAGTACTTGAATAGGGCATTTGTTTAAATTAGTTTTATTGATTTCATAATCAATAATCTATA

CTAAGCTGAAAATGTGATTGGCCTAGGAAAATCATAATCTACACTAGGGAAAGGTGGAAGGTAACTTTTT

TTTACCATGCCACTATTTACCACGGTTTTCAAGTTTAGTAGTTCTATTCACATTACTGATTATGAAGACT

GGAAAACAGTTGTGACTTTTTCATGAAAGGTCATTGCAAAACTGAAGCTTGATATGAAGAGAACTAGACA

TATCAAGGGATGGATAGCTAGCACATTTTCAAACTATAAATATGCACATACCATGCCATCCTTCTCCATA

TTTTTTAATTTGTACTATATTATTGGTGTTGATATTATGCCAAATTCTATAGCCATTTGTGAGAGCCAAA

ATCAAGTGAAATAATAAGTCAATTCAAAACAAATTTGTGAAGTTTTTTATTTTACGAAGTATTGGGATAA

AG

>Ca_linc_0223

CTACTTTACGCTTTGTTTGGTGGAAACAATTTTGGAGAAATTGAGAAGTTTATTCTCTTTGTGAAAAGAG

AAAAAGCCGAAAAGTGAGAAAACCTAACACTCCACCATTTCTTCAAGTTTCGTTGATTTTCCCGCCAAAA

AACTCGCATAAGTTTTTGAGTTGTTGAAGCCAAAAATCAGACAATAGTCCCTTGAAGGTCATCTATGGAG

CGAAACACTACTAGTGTTGTTAGTGAAAATGAAGTGAATTTTGATGGTTTAATGGTTTAATGGGTACATT

AATGAAGTGGAGTTGCAAGTAAGACGCATGAGTAATTTTGGGATGGGTCAGAGAAGAGGCAGCAGATATG

CGCTTTAGTGTTGCTGTGTTATATACTCAGAGTGAAATAAGTGATGATATCTCTAAGAATGAGTGTAAGT

CTGTTCAATTGATGACTCAGTTAGTACAAACTGAAAAATGTTGAGATATAATTCACATGAGTCCGCAAGC

GTTTCTGCAGCTGTGCGAAATTAAGATCATCGGGTAGAATGAAGGATTCCACAAGGGGTACAGTTGAAGG

GCAAGTCGCCGAATTCCTATTCATTGTAGGGCATGATGCGAAAATTATCATTCACGGGAGACATTTAATG

GACGCTTTGACAATGTTTTACGATCCATCATATCATTGGAAAATGAGTTCCTTGTCCAACCTTCTGATGT

AAATTTGCCTCCAAATATTCTTGGGGATAAAAGATTCTATCCTTACTTGAAGGTAATAATCTATTTAATT

TAGCTGCTCATTGCTTTTCATTCATGCAGTCCATGATTGAGTTTTTATAGTGACATAGTAATGCATTTAT

AAAAAAGGATTGCGTTGGAGTCATCGGCGTAATGCATAGTCGAGTGAAGG

>Ca_linc_0224

AGTGTTAAATTTTGAGTGAGATCAATGATGAAATGGAAAATTGAGGAAGAGAACTGAAACAGGGACGGTG

TTGCAATGGCGCGGACACCGTGAGGATTCAACGGCCAGAAAGTTAAATCACGAAAAGTGTAATAAAAAGA

GTAGAGAAAGTTGGGGAAGAAAAGTTAAGAGCCGGAAAACAATGAAAAAGAGAAAGTGCTTTTTTGGAGA

AGTCGTTATCTGAGGGGCACTTTATTAATAAAGAAAATGGTTTA

>Ca_linc_0225

CAACGAGAAATTACAAAAATGTTCAGTTTGAAGATGACATGAAACACTTATAATTAATATTATTGTACAT

TTCACAATACAAACAACTTCCTTAGGATTCTATATAAACATTGTATACACATAGCTTCTCTTATACCATC

ACTATCCTCCTTTGCTCTTATCTTATCTACCTTTTATTTTATTTTATTTTTTCTTCAACATGTTCAACAA

ATTACCCCTGCTTTGCCTCATATTATTTATGGTTTTCTCATGTGTTGCATCTTCTTCTATCCAAACTTCA

AGAAAACTTTTGTCAAAGATTGAAAAGTCCTCAATGCAAACTACACATGTTCAGGAAAGTGCTATTGGAT

TAAGTAATGAAGAAAAATTCACAGTAAAGGGAAGGGTAAATTTGGAAATAGAAGATTACGAAGATCCAGG

AGCAAACAACCACCATGACCCTAATGCACCAGGAAGAGTTTAATTGATGCATAAAATTTTGAAAATTATT

CTCCATACATCTCATATGGGACATACCTATATATTCTTGGTTTTATGATATGCTTTCATCCATTAATTTT

TATGGATGAAATTTGTTTGGCTGTAAATTGTAGGATATTATGGTAATTAAAGATAAAGGTTTTTTTATCT

TTCTATTATTTATATGAGGATGATTATTAGATATTTG

>Ca_linc_0226

TGACTCTGGCCCATTCGATGTCATGCAGCTCTACACGATGACATTTACCGTATGAAACCTTTTCTTTACC

CAGTAACGCCTCGACGAGACGATGCAGAAAGCGCAGAACTGTGAATATTCTCTTCTAACCTTCTTCTGTA

AATTTGCTTTGTTTTTTTCAATTTTATAAAACTCTTTGTTTATTTGTTGTTTTTTCTTCAATTTTTGCAT

AATAGAAAATTGATCCATATGCATTTTCCTTATGCTGCAAATTTGAGGATTTGACCTAGAAAAAATGGAA

ATAATTATTGGCGAAATAGAAATAATGTTTTTCCATATTCACTTCTGGTTGTTGATACTGTAAACAATGA

TGGTTTGATTTCAACAATTTGATGATGTAGAGTAACTTTGTAGCTGGGTGATGGTACATGCAACATTGTT

GTCAAATAGCACGGTGGTATTCCCCTGTAGTAGTGTAGTGGAATTTGAACCAAATGCTATTGTTATGTGA

AACACTACTGTACATAGTGTTGTAAAATAAGGGCTATAGTGGTTTTATAGCGTTGTAGAGTAAACAAACC

ACTATTTTATGCTATTAGCTATCGACAACACTGACATGCAGTTATGAAGGAGTAGTCTAGATATCTACTT

GTAAATAACTGCCCTTATTCGACATCTTTGGAAAATTAGATATATCAACTAAATGGTATTGTACAGACAA

GTAACTCTTTTGGAAAATTAGATATAACAACTAAATGGTATTGTACATGGACTCTGTTTGGCATAAGCTA

TTTCTATAACAAAAGATAAAATAACGTTAAAACTGTTTTTGTTCAAGTTATAAACTGTTTTTATAAACTA

TCCTAGAGAGTCTATGAAATTAGCTGAAAAAAACTTATAAACATGTGATAAGTTGATTCCACAAACTCTC

AAAAGTTTCACAAGTGCTTATGCAATTATGTCAGTAGATAAATTCAAATAGGTCAAATCAAACTGGTTGA

TGTTGTATCAAATGGGATTTTGTTGTTTTGGTTTACACATTAATTGTCAGATCTTCTGTAGTACCATGAA

TTGGTGAAAAATATTACTGCTATAGAAATTTAGGGTGGACCGTTCTTCCTAGTCTTTATTTGATATAAGG

CTGATCATGTTCCAATCCCAATGTTATGCTTGTGACTGATCTGTCGAACCATAATTTACTAAAAACGTTA

AAACCTACAATTTTTATACTACTCACATTCTCATTAGTTTTTGCTGTCTGCCACATTGAAGATTTATTAA

GAATCTTATAGTTCGTTTACTTTGTAAAAATATTTTTTGTTTTCATTTCATAGAATTTGTTTTAATTTTA

CCGGTTGATAAGAAAGTAAGAGATTTTATGAAGTGAACAATTGTCTATATTTTTTAAAACAATGAAAATA

TCTAAAAAGTATTTTCATTATTCTCGGAAATGTATCTTCATTAACTAGTATTTCATTTTCTCTCCAGGAC

AATGTTTTACTTAAATTGTTTTTCATCAAGATAAAATGAACACACATTTTAGAAAATGGAAATATAAAAT

ATTTTCACAAAGACACAAACTATAAGGGATCTTAGTGTTTCTCAATTATGAAAAGGATATTAACTTTGGT

TTTTCTGTTCCCACTATTTCCAGCATCGTACAATACATTCAAAGGATGTTGAAGCAACACTTCCTTGAGT

TTGCATCACATATTCTATTATTATTATAGCTGTCAAAATATGTTTCTGTTTAATATCCCTCAAGTATATG

GATTCAGTTATATTTTAATTCTTGACAATAGCTTGTATATGACTTTGATTGATATCCATTATAGGAGTGA

ATTTGTGTTGGCTGACATGCTAGTTTTCCATTTTGATTTTAAGAACACCAAAATATTTTGAAAATGTTCA

TTATATTTTGTTGATAAAAGTAATGCCTTGCTTTTGGTAAATTGGTAAGTTGCATATCCTGTCTCATGCA

TGTGAGTCTAGCATGCTCTTTATCGTACTAGTATGAACTAACTTATTTTGTTTTGTTAACAATACTCTTA

TTATTTGTTCTGTTTTCATGGGCAAACCATATTTGCTTTTCACCAAAACATGCACACCCGAATCACAAAA

ACAGAAGAAATAAGAAGAGAAAGACTGTAAAGACAAATTCCTAATTTTCTATAAAATACTGTTTTACATG

ATGAGACAGTACAGGTGTTCAGAATTCTGATAAGAATATATAATACTCAACATCTTTACTTTTGTTGGTT

AAGCAATAAGACTTGTCTGTAGGGTCCAGAGATTGAAGATACGAAAGTTCGTTAGATTCGAATACTTCTG

AAACATGACATTACAAAAGAATTTCACTATAATCTCGCCGAAACTGAATACTAGTGCAAATGAACATTTT

CTGTTTGAGGAGCTGGGATCATCATCTATACTGTCATTTTAGATGATGCTGGTTCATTGAATTGCTTTCC

AAAATTTATGTAGTAGCCTGACTGTACAAAATACATGCCCGATGTCTCTCTATCACAAAACTCTTAAAAC

ATAGATTGAAACTAGAGAAAAAACCAACCAACTGTTGTGAAGAACTTTTGAGAATGCAGGTACAACTCAC

TATGTTTCTAACCTGCTCTGCATTTCCAAGCATGTCAACTTCTGTTTCTCACACTTCTGGATGTCAGCGA

CTTAGCTTAGTTACGATTTCTCTCCAAGTCAAGCGAACAGTATATGTTAAGTCCTGTTGTGAATAGGATC

TGATCCTCTTCGCACTCTTCTTTTGCTCGAATAATTTGGATCAAGCCTGTTGTTAAAATTTGCTGAACTC

TTCTTTGTGGGATCAGACACATTGTTGACATTTGCTGTATTCTTCTTTGTGTTCAACATTTCCTTTGTAG

CTTTCAGAATATTAAGAATCCAACAAAGAGAAGAAGAATTATTCTAGAAGGATGGAACACCATATTCACT

TCTCTCTAAAACAAGAATAAAAGGTTACTCCATATACAAATTCAGGTCTTAAATGGGGTTAGTATGGTAA

ATAATCATTTCTTTGTGATTAAGAAAGAGCTTATTAAACATTAGTTGCTTTTAATTGGAATGAGCAGACA

GGGAAAAAATGGGCCAGCCAGAAGCAGGAGATTGCAACAGTGCTAGCAGCTCATATAAAGAATAAATGTG

GGAATAGAACAGTCCCCAAAAGAAACAAACTATTGCTTTATGCTCTTTACTAACTTCTAAGATCTCCCCT

TGTTGGCTTTAGAATTCCAAACTCTCTTCCCAAGTTTGGAACTGTCCAAAGCATAAATTCCCCACTTTAT

CACAACTGGGTGACATATTTTTCTTAAGATGAAGGGTGACTGGTCATAGTCCAAGTGAAACAAACAACCA

ACGCATGAACAATTTATGTTCTCTCTCCTATGTTTTTTGATTTTTATACTTTTTTATCATCCAATTAAAG

TGACAGTTTATGTGACTCGTTGCTTTCCATTTTTATTTTTTCTTCCTAACGTTTGAAACTTCCTTTTGAG

TGTCACCTTGTTGCAAACCATATGCCCTTAAAGAATATGCTTCTTTATTTTCCCTCTGATTAGTTGTTAA

TTTACCATTGTATCGTAACATGTGACATTCTAATGTGGAATGCATTGTTACTAGATTGTTTGTTACATAA

CCTTGCTTTGATTGGCACAAGCACATATGACAATGGGGCGGGGCGGGTATGTACGAGTAGTGTTATCGCT

TGTCCCTTATTCGTACAAATATTTGTTATGCAAG

>Ca_linc_0227

TGACACCTTTATTTCTTATTTTTCTCTCTCATGGATACCCTTAACTAACAAAAACTTCCATTTCATTTCA

CCAACCAACCGATTGCACCCTTTTTTCCACCAACAAATCCAACCTTATCTCAAAACATTTAAAAAACATC

AACACCGTACCGTTACTGTTCATCGTCTCTCTCGTCCATCAATTTTTTTCTTTTTTCCAAAATTCTCTAA

AGTCTTCTACACATGTCTAACCCATTGCATGTCCCTATCTTCATCTCAAGATCCATTAGCTGACCACACT

AACGCTAAAAGAAGATAACAAGCAACATTTGGGAGATCTATAAATTCGAACCAGAGTATAAAGGGAAGAA

GAGCTAAGAGCTGAGAAAGCAAGCAAATCCCAAAACCAATTCGAAGTATAGGAATCCCTTCTTGGCAAAT

CGTGAGTTCTTTTACATTAATGATTGACATACCTTGTATTTGGTGTTACAACATGATTATTAAAAAATTG

ACCTTGTTAATGAAATCAAATTCATATTATTAATAATAATAATTCTTGAGCATTAGAGTTCGAAAAAACT

TAGAATTTTTAAAAAATTACATTTTGAAGGAGTTAAAGTTGACTTATTTTGTAAAATAAAAATAAATTTT

GTTTAAATTACTCTTGATTATTTTAAAGAAATAAATTGAGTTGAATTTTTATTGATTTAGAAAAAAAAAA

TAATTCTACACAATTGATTTTATTTAATTTTTAAAATAGCTGAAATTAACACCATAGTTTACTATGTGTT

GTTTTTAAGAAAATAAAAAAGAGTTGTTGTTATGTTGGTTTAGAGGGGTTAGTGGGAATATTGAATCGTG

AGGTGTTGGAATATTTGTGTAAAATAACTGATTTATTATAATATTATGAACTCATTAGAGTTTAACTTGA

CGTTAGAGTTTTATATTTTAAGAACTTATTAATGATTTATAATGGTACTAGTAAAAAAAAGTCAAATAAT

AAAATGTTTACTGGTAAGGAAAAGGAAAACTAAAGAAAGAAAAAACAAAAGTTTTGAAAAGTTATCTAAC

TTTTGAATTCTATAATCTATGTATTTTATGAGTAGAATGTTTTTTTTTAGTAGAATTGTATTTTTAATTA

TAAAATTGCATTTTTTTTATTAGGGACTCAATTTTTAAGATTAGAACATGCCTCAAAAATCTGGTGTGTT

ATGCTTTTAGACCAATTTTGTGGCTTGATAGAAACTGAGTGTGTATCTATAAACTTGTTGAGTAGCTAGT

GTGAAAGGAGTATGTTATTTTGTAAATTTATGAATTAGTGTAATTCATTATCGTAACCAAGTATGTCTTA

TGCTTGTTCATGTGTACTACTTTAATGTATATAGATATACACTTTTGCTTTTGTAAGTTTTGAGGAAATA

AGAAAATAAATTAGAACTATTTTAGAAGTGTTAACTTATGGGAAAAT

>Ca_linc_0228

TCTCTATCAAAATAATTAAAATCATTAGCTTAGAGAAAATAAAAAAGCGCCATGAGGAAGAGTATTCGAA

TAGTACCAATTACTATACCGGAGGAAGAAGAACTGCAAGAGCCAGTACCACCACTGGCTATTGAACAACC

TCCACCGCAGCAACAATCATTAATCAACGGCTTAGATAAATTCTTAGTGATCATAATCGTAACTCCAATT

GTCGTTATGTTGGTATTTGGTATTATCTATGGTATACTGCATTATATGCGACAACATCGACACTGACTTA

AACAAGTTATGGCAGCAAACCCATTATTACCAGAATATTCATTGAGGCCATTAATGTTATGTAATTTTTT

TATTGTTTTTTTTTGGGGACAAGTTAGTAATTAGTTACCTTTTCTCTCAACTATTTCAGCGATTCTTGTT

TGTCACTCTCCAATGAATTATTAGTCATGTGAGTCATTGTTGGGAGCATTATTACTATCATTG

>Ca_linc_0229

TTCCGAAGCTTGTGCTCAGGATCCTATGGTGGGGTCTCATCTATCTCATCCGAGATTGACATAAACAGTT

TCTTCCCTCTGGGATTGAGGTGTAAGTTGCAACAAACCAACCCCAGTTGGTTGTGTTTTCACTTTACTTT

TTCTGGATTTCTAAACATGTTTATTCTGATGAGTCAGATCTATTCAGTGCTAAAAGACAAATAAATGGTA

GGGATTTTGTAGCTTTTGTTTGATTTTAAAATTTGGGTTTGTAATTTTGTAGTTGAAAACTTTTCGAAAA

GTTGCTGATTTTTTATACTTTTCTTTCTTGTCCTTTTACACGCTGTGCTCTGTCCTTGGATTCTACTTGG

AAGAATTCTTTTTCTATTTTAAAATAAAAAAATAAAAACAGATAACTGGTACTTCCTTCTTCTTTTTTGT

TTATTTTCTTAGGCTCTTCGAGGGTACTGTTATGTATAACTACGGTATAATTCATATTATAACAATGGAT

AACACATGAATTGCACAGGTACCATACTAGTTCTTAAACAAAACAAACTTGATTGGTATGTGTCGATGTT

TTGTGGATCCATGTTTGAAGCTTAAGATAGAATATGATCCTTATTTTTACTGATTCTAGTGCAAATTTAA

ACATTGTGCTAAACTTTCTATATGGTACTTTCTTATGTTCTTTGGTTTACTTTATAATTGGGATGAAGAA

TCGAATTGGAATCTTTTCAATAGCATTTAGCAGCTCAAGTTTCCTTGCCTGTAAGCAAATTAGGAATAAT

TACAAAGGTTTCTTAATTTATTAAAAAGGTTCTTG

>Ca_linc_0230

AGGCATTAGAATACATTCACCCTAATAAACAGATAAACAGTATCGATGCTTCCTCATCTTTTTCTTAAGA

CTAAAGCTTATCAACACTATGATAATGGTAGGACCGTCCAATGGTGGAGTCATATGAAATGACTTTCTTG

AATCCAAGGCCCGCTCATCATCTTTATATCTTGTAGTTTTCATTAACACATCAACATTGAGCAGCTGGAG

GAAAACAGAATATGGGTTGAGTACTAGAGGATTGGAG

>Ca_linc_0231

AAAAAACAAAAGCCTCCAACTGAAGTTTAATTCCATGAAGCTCTCTCAAACTAATGGAGAAAACATCATT

TCCTAGAAGTTTTTCAGTTTCATGTTGGGGTTGTCTCAAATTGAAGCTTCCTTGGACAAAAAGAAAAAGC

ACTTACAACAAATCAATTGGTGGTTTTGGGTATGACCCTTTAAGCTATGCTCAAAACTTTGATGAGGGTT

TTATGGAGGATGATGAAGAATCTTCATGTCGTAGATTCTCAGCAAGATATGCAGCACCTTCATGTTCAAT

TAAATCACTCAAGTAAAAATTAAGAGAAAGAAAAGAAAGAAAATGACTCAACCAAGAGAATGATGGCAA

>Ca_linc_0232

ATTATTTCTTTTTATAACTCTCATTGTCTTTCTTCAGTTGAGCTCATGTATCAATAAAAACATACTAGAA

TTAATTTAGGGTTTTCTGGCAACATCTACTTTTGAGCTTTAGGTCTAAGCTTATAAGGAAAAAGAAATGT

ACACACATATATGCATGGTGGATTGATCCAACAAAGATGAATAATAATGGAAGGAAAAAAGAGTGAAGGA

GCTTCCTACAGCCCAACCATAGATATAAGAGCAGAATTAATTAGTACTAAGTGAGTGATATAATAAAAAA

ATGATATTCCATTTAGAATGAAATAATAGCAATGTGGTTGGACTGAAGGGAGCTCCTTCTTTCTGTTGCC

TCCATTTGTGTAAGATTGACTCAAAAGACTTTATGCATGTCTCAGCTTAATATGTATATATATGCTTTAA

TTGAGCATATAAGAAGGTGAAGAGTGAAGAGTGAAGACAGAGTAAAGTACAGTGAAAACTACTGTAGCTG

ATGTTACATATCGTCTTGTAAGTATTAACTCTTTTACTATTCATGCACAACATTCAGGAAACATATATCT

GAACAGTTTCATATCAATTTAATTTCCTCATCACCCACTTTGTACTTTTTTCAAACTACGAAATATTCAA

CATCAATAATTAAATAAATCAATATTCTTTTCTTATATAGACTTCACTTGCATGTCAATAAACAGTTACC

AATAATTTATTTGGTTAAAACAAACAACAATCAAAGGTATAGAGAAATTTAAAATGTGATCTTGTTTAAA

AATATTTTAATAAAAATTACAACCAAAATTAAACAAAAGAAAATTAAATCTTCAACTTGAAATTCAAATT

GAATGTACAAAAGATTATTTA

>Ca_linc_0233

GGAATAAGGAAAATTTTGTCAAGAATTTGTTGTGTAACCGCAAGCTCATTTTTGAAATATGATTGTCTGA

TAAGAACTTGAAAGAAGGATCACAAACATATACGATTACAAGAACTTTTGTCCCCGCCAACATTTTCCTC

GATACAACAAGTAGTATCCACATCAAACAGGAACCAAAGTCACCTCAACCGTCAACGTCCAACTCAAAGG

ACAAAGAGGATATCAAACAAAAAGGTAAAATACATGAAGAAGTGGACTATAGAGATGAGGATAATGATAA

AGACAACAATGACAACCAGTTATCCAAAGGAAAAATCAAGGTAGAACAAAACAATAAAAATATTCAAATA

TCATCTTCAGATGAAGATACAATCAAGACAAAAATTACAAAAAAAAAGAAGATTATGGTCGAGAATATTA

CATCATCCGACGAAGAGCCCATCCTTCTACGTACCTTAAGGGGAACAAAAAAGAAGAAACCAAAAGAAAA

ACAATCTGGAAGTGTAAGGAAATTGATAAAACAAAAAAAAACCAAAATAAGTGAAAAAAATTAAAATCGA

TGAATG

>Ca_linc_0234

TTTTATTTTTTTTGTTACAAGGAAGTAGTAAAAAATAAAGAAATACAACTAGAGTTTTTGTTTCTCTTAT

ATTTTGTTATGAATTTGCAAAAGTCATCAAAATTCCCTAACTTGGAAAAGAAAAAGTTTATCTTTGCTTA

CCTTTGTTTTCATTTCTTTTGGAAAATAGTGCTTTTGCCAGATTTGTGTGCTTATTTATTAGATCTTCAA

TTCCTTTCCTTTCTATGACTCTATTGAAGACAGTGACACCTCTCTCTTCACTCATTTTTTCTTCTTTCTC

CTTTCATTTCCTTTGCTTTCTTCTTTTATCATCTTGTTTATTTAATTTGCACTATGTTTATTATTTGATT

TAATTAACTTACTCATTTTCTCTTTTTAGTTCATTTCTATTTATTTATACCCTTTTTGTCTTTACTATTG

TGGTTCTCATTCTTTCAGTAGTATCATCCTTTTGTTGAAGCTTAATTTGGATTCATGATGGGATCTTTCA

CTTTATGAGAATATGGTGGAGTCACCCCACATGCGATCAAAGAGTGAGGAATCCATGTGATGTTGAGTGT

GTAATTTATGAAGCTAATAGAATGAAAATAGTAGTGGAGGAGTATATGTTAGGAATTGAGATTGAATGAA

GCAAGCATATTGATTGTGGTGTGTTGTGTTGTGTTGTGCATGCAATGTGTCGGTTACTTTATTCCAAAAC

AGCGAATAAAAACAGACTTTGGGAAGATAAATAAATATTGTTGCACGCTTTTCTTTTATTTTCTTCGCTT

CTCATTCCAATTTACAACACTGTCATTTTCATCAGGTACCCATTTTTCACCAACGTGTAATATTATCAAC

AAACCATACTTATAAACATTGGGTGAGACGATTCTACGGTTTGAAGCATAGTTTCTTTTTCTGGAGATAA

TTGAAAGGTTGAGTAAACATGATGATATGATGGCCGATGGAAACAAACATGAAGTGGCTGAATTACGCAA

TTGGAACAGGAAACTCTGCCTTTTCGTGTAGCAATAAGCTATAGAGGTTGTAACTTTGAGCTATAAAGTT

CTACACTTTTTGTTTAACATTCGTAACCTTTATCATTCCCTCCATATATATTTCCTGCACCATACAGCTG

CTACTTTCTTATTCTCTTTCAATCAAAC

>Ca_linc_0235

TTCCCTTTCATTTTTTCATCACATAGTTCGTTCTTCTTCATCCTTCTGCAAACCCACGGATCTAAACATC

GTTCGTTCTGCTTCATCCTTCTGCAAACCCACGAATCCAAACATCGTTCGTTCTTCTTCATCCTTCTGCA

AACCCACAAACCCACGAATCCAAACCCGCGAATCCAAACCTGCGAATCCAAATCCATTTTTCATCCTTCA

ACATTCGTTTTTCATCAAACCCAACCACATTTAACCTTCATCCTTCAGTGAAAACGTTTTCATCCTTCCA

CATTTAACCTTCATTCTTCAGTGAAAACGTGAATCGTGATTCGCAGCTTTTAGCCTTCATCGTCGGCGAG

TTGGTCCGGCGCAGCTTTCAGTCAATTTCTTTCACTATCGAGAAGTGTTTGTGGCTTTCTCCGTTTGATA

CATTTTTCAGACACAATTTTGATTCCTGACAAATTGACGCACGCTTGAGAGTTGCACAAATTCAAAGGAC

TATGCATATATATATATATATATACTTATTCTTATATGATGATAAAGTTCATTGGCTTTGGAATGCGTCA

AAATTAAAGCTGATCTGACTCCTCCTACCTCATGCCAATGCTCAAACAACTAAATTCAGATGAATTCGCA

TGCTTCCCATCAATCAAA

>Ca_linc_0236

CTTTTTACCAAGAATATTGTATCCTTCAATATTTGTTTCGTCAATTTATTGACAACAAATAAAGTTTGAT

AACATATCTATAAACATGGATAATAACAAAAATCAAGCATGGTCTTCATCTTCTTCATCAACTGTGAAAT

TCGATCAACTTTTCGGTCCCAAGGACTCTGCTTCATCTTCTTCCTTCTTTGGTTCCATTTTCCCACCACC

ACCATCTGTTGAAGGGAGAGGATCAAGAACTCAAGAAGTGGGAAGCAAAAATTTTGGAGCACAAGAATTC

GTTTTAG

>Ca_linc_0237

CACATATTCTACGAACAAGATGAACACATCAAGGAACTCTACTGCTCTCAAATTGGCATTCTTATTTGCT

TTCTTCATCATCACATCAGATATGTGCATGAAGTCAGCAGCACAGTCACCTGTAGTATCATGGCCCTGCG

TATCATCAAATCAGTGCAAAATTGGCAATTTTAGTTGCCAATGTATTAAAAATCAATGTGTTTGTGGAAG

ACCCCCTGTTAATGATAACAATTCTATTCCAAAACGCTCAATTGCAAATTGAGTAATGAGATATATAGTG

CGTCAAGCA

>Ca_linc_0238

CTTAAACCTTGACACAATGGTTAATATGGAGATGTGCGACCATTGTCGCAAGATTGTTCAAACCACAATT

CATACAAATGATGGCATGTATTTCAGTTGTTGCACTTCTTGTGGGAAGCTTCTAAGTGACTCAACAACTC

CTCCAAGTTTGAGGACAAATAGAAGTAATGATAGAATAATGAAGATAAAGACCACTCTTGTGAAGAAATC

AGAAACAAAATAATAATATGATATAACTTCTAATAGTGTTGATGTTGTGAAGAACTACGGAAGACACTTT

TTGATAACTATTGGAGAATCACATTATTTGGAATAATAGTGGTTGTTATTGTTCCTAAGAAAACTAATAT

CATTTGCAATAAGCATTTTAACTTTAATATATTGTTCCTCTAACTGTGTTTGGTGTAATATTTCCTTAAC

TGTTCTTTTATGTTTTCATACAAGTTTGAC

>Ca_linc_0239

GCACAACATAACAAAATCTTTAATTATATCATTGAACATACACAACATTTCGTCCCACGAGTTAGCTACT

GCAAATGGAAGTTCCATAAACTTTTTGTCTGATTCACAAACAAAAATTTTGGTGGAGATTTTTCGAAAAG

TGATTGCAAATGTTTAAAGAAAATCTTAATTGACTCCGTAACCAACAAGAAGAAAATAAATTCTCTCCGC

AGGATAATTATCAATACGTATAATGGTATCGTTGTTTCCGATCAATTGATATTTCCACTTGAGTAATGTT

ATTCTTTTCTTTGTAAATCCTCACAACCTTATAGTGTTTATGTTCGTTGTCACAACAGAAATCAAATCCA

ACAGAAATAAAATCATAGAAGAGATGTCACTTTAAGCATTTTTCATTTGTCACCTCCATATACTCTGATG

TGATTTAGTTGCACAACATAACGGAATTTTTAATTATATTTTTGAACACGCACAACATTCCGTCGCACTT

GTTAGCTATGGCAAATCGAAGTTCCATAAATTTTTTGTCCGATCCACAGACAAAATTACGGGTGGGACTT

GACAGGAAGAAATTGCAGTGAAGAAAGTTTGAATTCAGCCCGTTGAAAAAGTTTTCAAGCATGTAATAGG

TTCTTGTTTCAATCTCACCTTCCTCATTCATAATAATGAGACCCTGTTTGAAATGGAACTTCCTAAAACT

AATATCCAGAAGTTCCCCTTCCATTGAAAGATGCCGAAAAGACTATCGAAAAGAAGTTTGGCCCATATCT

CATGGGTGCATTCCCATGGTAGACGGTCAAACAATGCAACATCAGCAGCAGCAGCATCAGCAACATCATC

ATCAGTAGCATCAACAACAGTAGCAACATAAAGAACATCAGCAAAAACATCAACAACAAAGGATTCAAGC

GGTTGATTTTTATTTATTTTTTCCATCTGAAATATAAAAGAAGTGTTATTGAGATGAGTCTTGATTATAG

GAAAAAAAAATGCAAACAATATACCTTTCAAAAATACTTTGGAGAAGCACACAAGAGAGACGTTCTTGAC

ACTTTCTTTTTTTCACAAATATTTGCTTACTCACTCAAATGTTTGCTGCATACCGCTCTAGAGTTTACTT

ACTCACTCTATAATTATAAGAAGAAAGAAAAAAAAAAGTAATCTTTAGTTTATTAAAGATGATACTCACT

ACAAGAAAACATTAATATACCTATGGAAATTTATCCACGGATTTGAATCCGTAAGTAAATTAT

>Ca_linc_0240

TTCGGCAAGATTGGCGTGATACTTATCAATACTTACAATGTTATCATTGTTTCCGATATCTATCCATCCT

GCACCTTATACTGTTGTCACAGCAAAAATCAAATCACACAACCATGAAATCATAGGATAGATGTGGCTAA

GTATTATTATTTGTAATATGTGACTTCCATATACTCTGATACGATAGGATTGCAGAAATCTTTAACTATC

A

>Ca_linc_0241

CATTGATGTATCAATACCTTAATTTAAAAAAAAAATACCATATCACATACTCGTACCAGTACCTGATACT

AGTACCGTACCTATGCGTCATAGATCTTATGCTCATACAATTTTCATGCCACTGTATTGATGTTACCATA

TATTTCCTCTGATTATGATATCATTGTGCAAACATATCGGTCTATGAACAGGAGTTTGAAGTAGAGGTTG

TGTCGTTGGGTTGGGCTCTATGCTCACTAAGTTTGATTCTGATTTGCAACGGAAGATTAGGTGCACATGC

TTGAAGTGTGGTAATGAGTAACGGTACCTGGTCGCATGTGCAAGAGCGAAGTGATTGTTATATTTTTTTT

TTAGTTTTCCTACTTTTTGGTCGCATGTGCAAAAGTGAAGTGGTAATTATATTTTTTTACCTTTCTTACT

TTCATTTCTACGGGTCAAGTGGAAATCATATTCTTTGGTTGCTCGTTGCTCATTCAGGTTAGGACTAAAA

CTAAAACCTTTGGATTCTCACTCATTTTAAACATGTTTTATGTTTTGTGTCTACCTAATCCAACATGCCT

TAAATTATTTAGTACTTGGGTTACACATTATATTAAAGGTTTTGTCTATGGGTTTGAAGTCGGACACATA

AGATTTTGTCTCTGAGAGTTAAAC

>Ca_linc_0242

GATCAATTATGAATAAGGTCCTTGTAAATCTTTTCAGATTTTAATAGTAGGAATTATTATTATGCAGTGC

AATATTATGCTGGCTGTACAAGAGAATATCTCTAAAAGAAAGGGCAAATGAAGTTCAATAATATAGTAGT

AATAACTAATAAAAGTAATACATCTAAAGAAGCTTTTTAGCAGTACATGTACATCAGGTAATCAATAGAT

TAAGTTTGAGACTTAAGCTAGTGAAAATTTATATTTGTAGCTATATACTAGTCTACTATTAAACCGAGTT

CTTCAAAATCTAGCCTTAAACTGACGCGGTTCACTTGTTTCAACGATTTTTTTGCCTGCAATGAAACAGA

TCCTTGCCATACCACATGCTTAATAATATCTCATATTAATTATTTTGTTTGGTAAAGTGAATTGTAAAGT

TAGTAACCCTACACATGTTTTGAATCTTCAGCATTAAAGTCAAAGTCTAATTTCTTCCATTGTCTTTGTA

GAAATCATAGAAAGCTATGTCTCCACATGGTTATTACGGCCTGTTTTTTCACTTCCCGAGTTGATGCCCA

ATGTAACATTCATTGATGTATAATGTCCTGTGATTGAAGCTAATATATGTCCCTGGCATAGAGAAGGGGT

CAGTGAACTGGTGGGAAGTAAGAAATTGAGGGGTCACATGGGATATCATGTAAATCACAGGGGAGGAGCA

ATCTTATCAGCAGCTTCTCGAGAAACAGAAGCATGCTATAGATTTAATGTCACATAATTAAGGCGTGTTT

TGGCTCAGAACCTTTAATTTTTTTAGTCATTTTAGACTACGCTGATTTTATGGGTGTTCCTTATGAGTAA

GAATTTTATTTGTTAAACAATATTTGATTGGGTTTGTAGAAAAATGTGACCAAATGATAACCACTTGGGT

TTCGCAGCTGGAGCTTGAGCAGAAAGCCAAAACTCTGCATGAAGATATAATAAATCATGTATGTAGCTTA

TAAATATTATTCTGTTGTGTAGTAGAATCTAAAAACTCCCAAATTTCAATCTCAAAACTCGAAAAGGGTT

TTCCGGCAATGCTTCCTTCTGGCAAACCGACCTTCCATCTTTGAAGCACCGTCCTTCCTTCTTTCTCAAA

CGTTTAGCTCTCTCGTTCGTTTCTATTCCTCCTTACCAAGTTTTTTCTCTTATTTATTCTTCTTTTATGT

TGTTTCAATGACTATTTTGATTGTTATTTTGAACAATTTAGTTTAGTTTTGATTGTTTTCTAGGTGTGTT

GTTTCGTGAAATTACTATTCTTC

>Ca_linc_0243

AATGTATTTTTGTTTCTGAGCGAAGCAAACATTATCATTACTGAGGGAGAGTCCCAACTCACAGATCGTG

AAAGCGGCTTTTTACTATCTTCTTCGTCGGTGGGGTGTGTTTGTACTCTTTTTACAGATCCTTAAAAGCA

AATGCTGTTGCAGTGCTGTTGGATTGGAGGAAATTCAACCCTCGCTGCTGGCATTACCATTACCATTACC

ATTACGCTTCGCTTTGCCTGCTCCAAATCTTTTTATGCTTAACAACGCATCAATCACACTTTGCTTTGCT

TTGCTTACGAATTCTGTATCAAGTATTACTTTACTTGCTCCTATATAAAGCAATCATTCTCTTCTCCAAC

TGCTCTTTCCCGTACCTTAAAC

>Ca_linc_0244

TTCGGCATCAACAAACTTGTCACTTTTAAGTTCCTCATGATAGAGTTCATATTTGATGGACATAAAAGAA

TAATGTCATCCTGAATCAAAGCCTCATCTCATATTGGATTGTTCGATTCCTTACCAATAGTCCATCTACC

CCCTTCATTCAACAAAGCTCGTGTGGGCCATAAGATTCTCCAAGAATAAGAAGGATTATGTCCAATCTGG

GCATTCAAAAAAGACAATAATTTAGCCTTAAAAAACTTTTTCACTAAAGCATCCGGTTGGATAATGAATT

TTCGTTCTTGTTTACCAAGCATTTCTATACTGAAAGCATGCAGGTTCTTGTGCTTAAACTCTTACAAACA

TGAAATTATCATCGTTAAAAAGCAAGTGAGAGATTTTCAGGGGCACCTCAAATGATCTTGACTCCATGCA

ATTGACCTTGTTGATATGCATCTTTTATTAAACTAGAAAGACCTGCTGCACATAAATAAAATATATAAAT

AAGGGGATAAATGATCCCTTTGACGAATACCACGAGATGGTTGAATTGGCCTGACATCATCAGTGTTCAC

CATAATAGAGTACTTCACCAACGTCACACATATCATGACTTAGAAGGTGGAGTACCTCTCAATCAAATTT

ATCACTCCCTCTCTCTTTTTATAAGTTCTCTTCATCGAAGATAATCATATTTGAAGTCTGTCGGATATTA

AATATTGGCATCTTCAAGAACACATCCCTAAGCAAAAAAAGAAGGAAGAAAATCAAGAAGAAAATGAAGC

AGAACATGATGGCACTTCTTATCGGGTTAATAGGTGCATCATTTACCTTACTTGCTTACACACAAACCTT

CATACAACCAAGTCAATGCATAGCAATTGGTCTCTTAGTTCTCATGTTGGGATTGCTTGTAGGAGAAGGT

TTTATATCTTTTTAATTCATCCTTGTTTTTCTTTTTCTCCTTTAATCTTCCCTTTCCTTTGTATCCCTGA

ATTTTACATGTTTATTTGTAATAAGTCTTTAATTGGCTATTTTAAATTTTTGCAAGTATTTTTTTTCTCT

TCCATATGTATTATGAGTATAAATTGAATGGATGCAAATTTAGTTCGTG

>Ca_linc_0245

CCAGATTTACTAGGTAAATAAATTAAAAAAACTAATATAATAATAGGCTTAGAAAATTGAAAAAAGATTC

TAGGTCTTCTACTACTACTACTAAAGAGTGAGAGACGCTGCCTTTTTCATGAAATGGATTTAGAAAAAAA

TGAAAGAAATAGAAAGGAAATGAAGAAGAGAGAGAGAGTACATGACCGCCCATAGGTGGTGGCTAACGAT

GCTTTGAGCGTTTCCCTGTGACGGGTCTCAATAAATGTTTGGTGGCGAAGGGCGATGAAGGCAGTGGTGC

GCAACTGACTAGGAGTCCCACAATAGACATTCGCATTGAAGGCGTCGAAGGTGGGGGTCGCCGAATTCTT

CGAGGCACGGTCGATTGGAGATTGAGGTTTCAAAAGAAGTTCCCTTTTCCTCTCTTCATCTTCTATGGAC

TAATTTCTTCCCCTCACAAGTACTTTATTTCTATTTACAAGTTTTGCTTTGATGAGATAGTAGAAAGTTG

TTTGATGTTGCAATGGTTGTTATGGTGTCTAGAGGAGTGTGGTAAGGAAGAAGTGATCACATGAGTATCA

TTTTTGTCATCCTCACTCCAAGATCATTTGTGGGTAATCCCTTTTCATCATGGAAGATTAAGGTGTCAAC

TTCTCCAGCTCAAATTGGTTTTTTAAATACTGAAGAATTTATTCATTAGGAATAGAAACATTGGACATGT

TTTGTTGTTTTATAAGTGTACTCTGAGTCGTTGAAGGACATATACTATTCCAGGTTGTTGCTATACTACT

CAGAGGATTAACAACATTCTTATCTTTATGCTTCATGTTCTAGTGCCACGTCACATGTACTATTCATCAG

ATGATCAAAATTCTTTCTTTGTAATTTAATCTCCAGTCGAATAATAATTCTTTGTTAACTCTAACATTGA

CTTGTTGACTTAGAGGCATTGACTTATTCAAACAATAGCTTTATTACCGGATTCTTTAAATAGACGTTCC

AGAGCGATGACGACATTATAACCAAAGATAGCAGTGAAGACCTTCAAGGTCTAGGAGGACCATTGAAAAG

GGCACAAGCCAAGAAAGCTAAGGAAGCCTTAAAGCAAATGGTGACTACTATCATGGAAACAATACCAAAC

TTAGAAGAAACTGAGTCTGGTCAACTACATAAGTTCACTTGGATAGGACTTGGGAGCATGGGATGCTGCC

TATTTAAGTTGTTTTATTTAGTTTAAATTGTAATAAAAGGTGTGGGTCAAAATTGATTTTAATTAGAC

>Ca_linc_0246

GTGGGTATCATTAAAAGAAAGTAACTAATATATATTGTATTCTAAATAGAAAGAGTAGTTACAAATATTT

TTGGGAAATGAATAAATTGCGATATATATTTTTGGTTGAAAATCGTCAAGTGAAGCACTTGTTCTTGGGT

TGGGGAATTAGGGGTTTAGGTTAATTTGATTATTTATTTAGTGAGCAGAATTGAGAAATTGAAGGATGCA

TCTTTGGCCGACAAAGAATCTGAGAGATTCTTTCAAAACTTCATATATAAAAAAGCTGGATTGGAACTAC

CGGAGAATGGAAAAGGATTCTCAATCATCTGAATCATCAACACAACACAAATTGCTTGAAGAAGAAGACG

CTGATACTCCTCAAAGTGTTTCTTCCATATTCCATCATGATCTTTTATTGCTTCTTTCTTGCTGTTTCTG

CTGCGCCGCTTGTGCTGAGGAGAAATAAGGAACAAATTGCTGGCTTGACTTCTATTGGGTCATCAACCAC

ATTGTGACAAAACTTGTTCCAAGTATAGGGACCAAATTATGTACATTTTTATTACTTATTTGGTATCCCT

ATAATAATGTATAGATTTTTACATTATTATACAATTATTGTACTATGTGATTTGTTTTTTACATTATTAT

ACTATTATACAAGTGTGATTTGTAAAGGGTATAATGTAAACAGATTAAATATACAAGAATCATAATCTGT

ACATTGGTTCTCATTGTTTCTATATTTTAGTGACTGTCC

>Ca_linc_0247

CCAAAATATATGAGATTAATGTATTTTAGCGGAATGAATGCACAATCAAATCAAGGAAATCTGATTCCCT

CATTCCACATAATAGTATCTAGGATATTTGATTTGTATGTCACAGCTGAATGGCTTAAGTACCAAACCTA

ACTACACTATGTTAGTATGAAATTAATCCTTTCACTTTCATTGCATGACGGGGTGATGTATCACCACTAC

GTCAATTCTTTATCCATTTGATTTAATTATCACAATTTTACTTCACTTGTTAATTTAACACACAAAGTTT

CCTTAACAAGGTACAACGTTTTGTAAAACCACAATCTTTGTAATGCGATGTTTGTTTTCAATGTGTTGTG

TTTTAGCACTTGAATGCCACATGCTTGTGAAAATGCCTGAACGAGCATATGAATTTGTTGCAAAATAAAT

TCTCTAGAGTGACTTTATAGTGCATCATGGGTATTAAGAGAGATTTTTTTTTTCTCTCTTAATGTAACGG

CAAGTAGCCATTTGTACCTTGCAGGATCCAAGTTCGGGGACATGGTGTTCAAGTGATCGATTGTGAAGAA

AAACTAGCAAGCAAGTGTTATAAATAATTAATACTTGGCACTGAGGTTTTAAATTGCAGTAATGTGATGA

TTCTTTATATTGCGGAGAATCGGGATCGGAGGCAACTGATGCAAACTCAATTGCAGCCAAAGACTTTTTG

TGAAATATGTCTTATATATGCTTAAACTTTACAGCCAAAGACTTTTAGATTTTGGACTCATTTGATAACA

TGAGTTAAAGAATCTGGCTTGTCATGCCCAATTCATTTGAATGTCATCATATGTATAGGATCTTTGTTTA

GAGTTAGAAGAAGGGCAAAGCTTGCAAAAAGAGTTTTGGCTAGAAGGATAATGTACTAAGGGCTTGTTTG

GATTAACTTATTTGAGTTTATACACTATCATAACTACATAAGTACTTGTGTGATTGTTTGAGACAATTTA

TGAAAACAGCTTATGACATCTTTGTAAGCTGTTTTCAGCTTATTTATGTAATCTTTCAAATATAGTTTAC

GAAA

>Ca_linc_0248

CATAGTAGAAAACAAAACCAATTTTAAGCTTTACTAACACAATAAAATGATCTCAACCAAAAACAACGTC

CACACAAACAAAAACAAAATTCCATCTTTCAAATAAACCAACTCTAGTTTCAAATTTTAGCTTCCAATCT

AATAATATAATGTTCGAAAAGCAGCAAACACGAAGATAATGAGAAGATACATGCCTTTTCAGATTCCAGT

GAAGACAGAGACGGTGGTGTGCGGTGGAAGTTGGGACGTTACAAAGCAAAGGCTAGTGCATCAACCATGA

CACTGCATCAGAAACAGAAAGGCAGCAACAACAACAAAACAGAGCCAAAGCAGGCTGGCTTAATTTGGTT

TGGTTTAAA

>Ca_linc_0249

TGACTTCCTAAATAACAATATATTCCACTGACTATAAGTTCCAAACTCCAGCAAAGAGAAAAATTGCAAT

AAGTTCAACATTTGCCTTTATATTTGATTTGATAATAATTTACATTAAATGTTGACAAAAAAAATAAAGA

AAAAGGAAAATTCAAGTTCAAATGCGTCAACATAGAAGTAAAGAAAATATCATTCCAAAAAGAGGGCAAA

AAAACTATTAGGCAGGAATTATAATCATCCCAAATTATGTCAACACTCTAGAACCAAGTTCTGGATCCAA

ATCAGGTTTACTTGTTGGCAATAAGTCATTCTTCCAATCTGACCAACTGGAATGAGCCACGTGGCACCAC

TTGTTAGCCATTATATTAATTCAACCTATTTTTTGCTGATATAGATTTTCTTCTTGAAATGATTTCTGCT

GCCCAATTTTTACCTATTTCAGTTTGAAACTTCCCTGAATACCAAAAAGAATATTATGTG

>Ca_linc_0250

GAGACTCCCATAGTAAAAAACAAGCCACAACACATCTCAAACAACAACTACATGTGAGTTTTTTCTACAA

CCGTGAGTGCCGTCAAGGCCTCCAACCACCATATTTGAAATCACCTCGACTCCTTTATCTACGAGGAGAA

CCACAACCAAAACAACCATTCGGGAACAAATCGAAAATTCAAACCATCGCCTTCAAACCTCGATTTTGGT

CACTATGGTGTTGATCCAAAATAACGAAGACCAGTTTTGTGATCTATGCGTCAAGAGCTTCGTCCTACAA

TATTTGTTTTTCAATTAAAGCAAAATCTATGAAAACTTATTGTCGTCACCGTTACGGTAGGACGTGACAA

GCCTAACGATTTTGGGGAAGTACAATTGAATGAGGTTGATCGTGGCGGTCTGCTGTTGAGCCTTAAGGCA

AGATTGTTAGACCTTGGAGGTATTCGGATGGAGAATTCCTAGTTGACTTGGAGGTAGCACTCCAAATGTC

GGGAGCTACCATGCAATACACGTTTACCTCGTATGTCGTGTATATGTGCCTCGGGTTGAGTTGAGGGGAT

CTATGAGGACATAACTAATT

>Ca_linc_0251

CTAAAGTATGTGGAAGACTTACACTTGAAGGAGGAGTTTGTTGTCGATATCTCACTTGTTGATGTAAACA

TTAGAGTGTTTGCAACCATCTTACCCTTTTCCAATTGTTTCCATCATCGTCTTTAATCATCTACATGGTC

TCCATTGAAATATAATATAGTGCTCAAGCATGAAAGTGACCATTCGCGAACTAAAATCCATTGGCAAAGC

ATTATTTTCCATTTTTCTTCTTTAACTCTATCATGTTTCCAGGTAACCTACAAAGCTCATCTCAACTTCT

TTTTAGTGTTTCAGCATTTAATTGTTTCTGTGTCTTTTCTGTTGTCCTTCAAAATACAACACATATATTA

GAACGCGGATGATTAAAGTGTTCACAATAGCATGGACAACTTATACTGTGTTTGGTTTAGAGAACAAAGT

TAAAGGAAAGAAAGTGATAAGAAAATAAAATATGAGGAAATAAAAAGAATAAAAAAAAG

>Ca_linc_0252

AGTGTTTCGATTCTCTGTTGGTTTCGATTCTGTCCCTATCAACCCCGTTCAGAGAAAAGCCATCATCATC

ATCATCTGGTTCGGTCGCTCGTTTCGAAATTTCTGGTTTCAGAAGTTAACGTTAGGAGAAGAAGGATAAT

GGTGGTAGTATTAGGGATATTGGATTTCGTCGTTGCTGGCGTTTCTCTCATCATTGGTTTGTTCCTTTTT

GGTTTCATTACTTCCCTTCTATGTGCTGCCGCCTTCTTCAATAACGTCAAGGACGTCTCCTAATTCAACT

ATTATTTCACTTCTCTCTTTCCAACTATTAGTTGTACTTGCATTTGGGTCTTCTTGGGATGCCTTGGAGT

TTTCTGTATGAAATGTATCAAAAACCTACCTTCGAGCTTATGCCGGAATGAGTACAGATGGTGTTTCAGT

TTCAGAATCTTTCTGAAGTTTGGAAACATCTTATTCTCATAATTGCTTATATTGGAATGGATTATCTTTC

TTCACCATCCTGCTAAAGCTAGTACTGCTTAAGGTTCATGCTTGCCACAATACTATTCTTCTCTGGACCA

TTTTGTTTTACACTCTTCTATATATTATGTGAATGAGATGACATCCATTATTAATTGAAAGATATATCAC

AGTTTTGGATGTTTCTTTCATCTTACATGCTTCACCATTCTGTGAATATTTAATTAATTGCCTAAATCCG

AGTTCGGGGATGCTCTGAGTCCTTGCCTTATAGTTTAGCCATTCCTTTCTCCTGAAAGGCTGAAACCTAA

AACAAAAATCAGTATAGATAACAGAACACAAGGCTTTTGAGCACCTATATCAAAAT

>Ca_linc_0253

TAGATGTCATGGTGGAGTCTCACTGCTATGAGAACAAAACAAAGCTGAAGCAAATTTCTCACTACTCTTG

GGAAGAAATATTCATGCTGCTGAAATTACCAGTAAATCCTCGTGGTGCAACAATATTGCCTATAATGTTG

AATTTTAATTAATTTAATATCGGTTGAGTTGTCATGCGACTGTGACTTATGTTGCTGATGAATTTGTGAT

TTAATATTAGCCTTAGACTTTATGTGGCTAATTGCAAATGCTATGTGCTTGAGAACAATGATACAACAAA

ACTTTGATTGATTATTATACAAAATTGTGATTTGGAAAA

>Ca_linc_0254

CGATACGAGCCTTTTTGGATTAAAAAGCCCAGCCCAACACGTACTTGCTACATGTTAGGGTTTCTCTTCT

ACACTATAAATTGTTGCTACCTTACCATTCTCTGTTTCAGTGGCCATAAACTAGGGTTCCGTTCCGACAA

TTTGAAGTACCGTTCACGATGAGAGCCAAGTGGAAGAAGAAGCGTATGAGGAGATTGAAGAGGAAGCGCC

GAAAGATGAGACAGAGATCTAAGTAGTGCGGTTTTTATTTATCGTAGTTTCATCTTGTTCTTCAATTTGG

ATGTTATTGTTGTTCAATGGGATCCTCCCAATAACAGTCTTTACTAATTTTCATGTTTTGATACATTATT

AATGGTTTTACAGTTTATTTTTATTTTGTTAAATTTGGTTTAAAAAATCATTTTTATTTCTGCTCCCGAA

CTCTAGATATTATATATTTGGATTTTTATTATCGACTTGAAAATGCGTTGGCCAGTTTTTGGTGATTAGG

GTTTCGCGTTTTATTTACCAATGTTTTCATATGAATCCATTTAATTTTAACCATTATTAGATTATGGTTG

CTTTATATCTAAT

>Ca_linc_0255

CTACATTATTATCACTATTCTGCTTCTTCTTCTTTCCAATAAGTCTAAATATCCTTGTTTAGTGTTTTCT

TCTTCTAGACGACACACTGCGAGATTTTCCAATTTTTTGACCCTGTTTTTCTTTCGTATTATGAATCTAT

GAACTTCTCTGAAATTGGATTCTTTTTTTATTGGCAAAATTTACTAATTAATTAGTTAGATTATGTTAGT

TTTTGATACCGAACCCATACTTAAAACTCATTCAATGTTATTTAATTCATCAATCGAGTTACTTTTATTG

GACTCTCTCAATCTTGTTTGCAGTCTATTGAAAAGTGATATCATTAATCTACTCCCTCCCGACTCAATTA

TAAACAAAAAGTGCTTATATGTTTGGATTTTATAGAAATTAACCTTGGTACGGACATGCGGTTAGTTCAA

AAAATGAGTATATATCAAATAATATATCGATGAATCTATTTCAAAAAATGTATTGACAAACCAATATTGG

TGTAAATTTGTAGAAAATAATGATGATAATGGGATTGAGATTAGCCAGCAAATACCATTTGGTGATAATG

TTTGTAATAGCAATTGATAAGGATTAATCTGACATGTATAAGCAAAAATAATATTTAAAAG

>Ca_linc_0256

AATATATTGGTTAAAATGCAGGAAGTGTTAAAGTGTTCAATAACCATGCTCCATTAGATGAATTGCTGCA

TGAACATGCAGTAGTAGAGTAGACTACAACAAGTATGGTTAAGTTTGCCTTAGAACTCATTTTAAAGATT

GTGTCGTTAGCATTGCTTCTTTTAATATATCATTGTTGCACTGGCTATGTTGTTTTGTTTATGTCTACCA

TAAATTGTGCTGAACTTTGTTATAAGTTATAGAGACAAAAATCAAGAAGAAAAGCAAGTGGAATACAACC

ATTTCTAAATTCATGAGACTTATTTTGTCTTTTGTTTGTGTTTATGTGTATCCACTCTCCTCAACATACA

TAGTCCAACCTAATACACATCTCAAACTAAGGAAGTCATATATGATACTAAGACTCATGCACAATCATTG

CTATTGCTAAATTTTATGAAGGGG

>Ca_linc_0257

GATGATCCACAAATATAGTGAAAAAACTTAGTTTTTCTTGAGAGAAGAGCTTTGCAACTTGAGCTTAATG

ATCAAGTGTAGATTAGTTGTTTTGGAAACTATTGCATTTGTCTTCTGATTGATCTCCTTTTTATAGAGGA

TGAGAGGTTTGAATATTCATTTAAAACTTAATATAGTTGTTGGCCATTAATGTCAAGTGTCAATCTTGCT

TTCTTCAAATTTTCTCTTGTTTGTAGTAATTGTCTAGAACGTTCTACCTTGATTTGAAGGTAACATGAGT

GATAGTGAACGTGAGTTGTCAAAGGATCATTGGGACCATATAAAATTAGAAGCATGCCCTAGTGATGCAG

GTAACATGAGTGATAGTGAATGTGAGTTGTCAAAGGATTATTTGGACCGTGTAAAATTAAAAGCATGCCC

TATGGATGCAATAATAGTGACATTTGGAGTGTATTTGTGTACTTTTGTCCTTGTTTCCAATTCATCAATC

TGGAAGTGTGTTTATGCACATGGACCTTCAATCTTTGTCTCTTTTTAATGCACATTATGTTTGGATAGCT

CGTGTGCGAATTT

>Ca_linc_0258

GTCAAATATAACACATGGAAAAGCAGGATAATGAACTAAAGTATTATATTGACACATGTGTAAAGTGCAA

ACCCAAGTAGTAAATAGTTTAACTCTCAGAGACAAAATCTTATGTGCCCGACTTCAAACCCATAGACAAA

ACCTTTAATATAATGTGTAACCCAAGTACTAAATAATTTAAGGCATGTTGGATTAGGTAGACACAAAACA

TAAAACATGTTTAAAATGAGTGAAAATCCAAAGGTTTTAGTTTTTAGT

>Ca_linc_0259

TCGAAATAAAATAAATAAAAACAGAATACCCACAAATATGGTACGAGTCCCAGTACCGGTTAAGCACCCA

GTATGGGTACTTCACCATTTTAGAAGTACTTCATGCTTTATAGCATAACATGCATAGGGAATGTTTCAGC

CATTATTTCGTATGTAAAATGTAAACAGTATCCCAATCATCTTCCACATCATTAATTAGAACAAAATATA

AAATGACAATAGGTATTTACCTCTACAGACAATACCTGGACATATGCTTTCCCCATTCTTTCAACAAATA

TTGAATAAAAGTCACAAACCAAAGGTAGGTGACAGTTCACAATTTTATGGTCCGAGACTATGTAGATCAG

ATCAAGAAATATTGGGTAGCTGTTTTTACCAATTTTAAGGCATACAGATTTGGTGACAATTTGGTTTGAT

GAAGCCGAAATGCATATATTAGTCTACGCATAACTCATAAGTCAACGTGATAACATGC

>Ca_linc_0260

TCAGAGTAGAGTCCGCCATTAGATTTGTTTCACGATGAATATCTCTTACAACAAAATGAGTCAAATAGTG

CAAAGTATCAGAAATAAGTGAAACCAAATTGAAACTCACATGAGTTAAGTACAAACATTGATTAATAAAC

ATCCCGTAGCTAACTTCCACAAGAAGCAGCTAAGCCTCTCATGCCCCTTCCACCTTCAAACAAGACTAAA

GTGTATCCTTTAATACCATCATAAGTTGATATAGTAGAGAAGTTCCCATTCAGGTTCCCTAGCCCAGATC

AATAAATTCATGCATGCACCTCTTATCTAGGGAGCATAGTCAAAGCAAATCTTCCTGTAAAAGACAAGTT

TACCAAAAAAAACATCTTTTCCACCTTATCAAGAATGAG

>Ca_linc_0261

CTTTTTGTTAGTATCTAAAGTGATATATTTCTTTTTTTGAAAAACAAATGTTTACAAACACTAATCTAAA

CACAAGATCTCTAATGATTCAATAATAGTTACAATGTCTCGTACCATAGTAAGAAATTAGTTTCTACCAT

AACATGCTTTAGAACCCATAAGAACCAATCTACATATTCATAATTTCTTTGTATAGAAAAACGGTCTTAT

TCCATGTTTAACACAATGATGATCTTCACTACCAATGATAACTCTTTAATGTGAACCAATTCAAAATTCC

AATAAATAATCAAAGTGTAAAATTCTAATGCATCAAAAGACGATGAATATTAAAAGGTCTTAGGGTTTAT

TATTAGAAGAATGGATTGATGGTTTCTAGAGGTAATTGAAATTGTGAAAATTAATTCACCTTCATTAATT

AGTTTATTTTTGAAAGAAACCAAATTCTTCTTTATAAATAAAGAGTCTTGTGAAGTGTTATATCATCTCA

TCCAAGAACAAGTGTGTGAGAACACTTGAAAAAGTCTTTTGTATTGAGAATGTTATCATTTTTTGTAATT

ATTAAATTGAGAAAGTTTGTAGAGTGACACATAATTTGTGATTGAATTGCAAATTATTGTAATCATATTT

GAAATATTGAATATAATTATTGAAGAATTTTCGTAGACATAGACAAGAGACACAAAACTGTATAAATTAT

CATGTGTTTTTTGATCATTTATTTATGACATGTGTTTCTATATAAAATCTTAGGTTGTCCACCTCTTCCT

TTTCATAACTACTATCTCAACTTAGCACTTTATATATCTTAAAATTTGCAACATTTTCTATAATTTAAAT

GTATCAATCTATACTTTTTTTTTTCAAAGCAAAAGACAATTTTGTTAAACCAACCCTCTCCCACAAGGTG

TAGATAGAAGGTCAAGATACATCTTGAGTTGATTCCATAACAACACCCAAACAAGAAAAATAAACAAAAC

ACATCAATACATCCAAAGTCTTTTACCAACTAAAGTCTATTTTGAATTATTTGTCAATTAGACATACACA

CCTATATGCATACCATATCATATATTTTTAACATATTATTTAAATGTATTAATGTAAAC

>Ca_linc_0262

TCTTGATAAGTGTAAAGTAAATAAAAGCATAAATTAATATAATATTAAAAACTTAGTTAAAATGATAAAT

TGTTAAAAATTACAACATTGACATTAATTTAGGAGGAAACATTTGTATGAAGTAACTCAACAACTTTTAT

ATATTTATAAGAAAACTATATCCTTCTTATTACATGCATGTGCCTTCATGACTAAGAAGGACTATTTCTA

CTACTAATATTTTTCTCCCCTCTAACATATATATTAATTAAGACTTAGACTCGTGCGATGCACTGAAAGA

TAATTTCAAATTATTAATATTTTTGATTATAAAAG

>Ca_linc_0263

AAAATATGTTGACATAACCTCACCACAATGCACATTGAATGTTGTCATCAAAATCAAATGCCACTATTGC

AATTGTGTGTCATATAACAACTTGAGTTGGTCATCACTCATTGCAAAAACCAATACCAAATTTGCTTAGT

TGCTACCTCTAATCTCTTTACCCATTTCTCTTCATAAACCAAAATCTTCCATACGATGATCCTATTTATG

AACATTAATCTTTTCAATTTGGCTTGCTTTTTTATTACAATGACCAAAAAGCATTTTTGTCATGGTGAAT

GGCCATTGATTTTGGTGGAGTGTATGTATGTTTCTCTCTTATTCTCTCCCTTCCCCCTCTCCTAGTTTAT

TGCAAGCAAAATATTCAAGGGGAATAAACATCTAATTTGGTTACATACCACTTTGCTAATGTGTTATTAA

GTTATTGGAGTTCAAATATAGGAAATAGAAGAAAAGTGTTTGATTAATTAAGGG

>Ca_linc_0264

AGTTCTTCTCTTCTTTTTCAATAAGTTATATTTTTCTTCTTTAATCTACATTATTATATTTGAAGCTGCT

AGTTTGTGTATATATAGGCCCAACACAACACTGTGGCCACAGTAGATAATAGATCTTTAATTAAAAAAAA

AAAAAAAGATAATAGATCTAGAACTAGAACCACAAATTTGAAGCCTCAAGGATAGGGTTTTATCATTAAT

AACATCCATTGCTTGTGTGGTCCATCACATATGAACCATTGGTTACAAACGGATCATAGAGAAAATACAT

TCAATTGATTTTGAATATTTTCTTCTTGAGTCATATTCAAC

>Ca_linc_0265

ATTATGAAATATATAATTTTTTTGGACAATTATATTATAAATGGAATTAGTCCTCCTAAGTTGTAGGAAA

AGCATAGAATATAGAAATAAAGTTACTACAATAGGGAAATATTGTTACCTTATGCTTTGTCCCTTTCAGC

TGAAATAAAAAAGGTTGTTGCATTTTCACCTACGTGGCAAACCGGCATTGAATACACGACACACAAATTC

AAAGGGTACAAGACAACCTTATACCCTACATTAATAAACCTTGGAATTCACTACCACTAATTCTTTTCTT

CATATATCTCAATTGAATTCTCATAAGAATTAACGTGAATCTCATAGATAATATCTACACAGTAACGCAA

TGATATGGTCCATATTCAACAATGTATGTTAAGCACTAAAAAAAATTAATAATCCAAAATTTATAGTAAC

TATTTTCAAGG

>Ca_linc_0266

TTGACACCAACACAAACATAAACATGAGACACGATAGCAAAACTGACACACATAGACACCGATAATAATT

TGAGAAAATAGAAGTAATTGAATGTAATCACATCTGTCAGCGTCGTATTACGACGCTGACACGATATGTG

TTAGTGCTAACATGTGTCGGACATTAGGACATGTTTTCAATCTGAAGTGTCAGTCAAACAATAGGACATA

CCTTCAATCTGACGTGTTGATGTTACTGCTACATATTTTTATATGAATTACACTCTTCCAAATTGCTAGA

GGCGGAGGGAGAGTGGGGTACTTGGTAAA

>Ca_linc_0267

CAAAAATATAAATTAAGAGAGTTTAATACAAACAAGGGATGGAAAATCAAAATACTTATAATAGCATAGC

TCAAATTAAGAACATATAAAAAGATAACAAGTTTGCATCACTTTTCATGAATACAACAACATACATAAAC

CCTAGACATCTCTAGATTCATAAACTTTACTTGAAAAAAAAAAAACTTAACCAACGTGAAGAAAAAGATT

AGAAAAAAGAAAGATCCACTAAAGAGCTATAATAATAAAGTAGAGTGAAATTAACTTGCTTGATGTGGCA

AATTAATTATGAATCGGACCAAGATGCTTGATTCCCCATAAAAGATCTTGAAAGAGAGTTTGAGAATGAA

TGAAAGTACATAGTGTACCAATCTTCAGTCAGAAACTATAAGAAACAAGAAAGTGATGGTGATGGTGAGG

GAGGGAGGGGGGTGTAAATAATAATATTCTTGTTGTTTGTGTATATAGAAAAATAAAAGAGTAAGTTGAG

ATATTGACCTTGTTTTATTGTTTTGGTGATTGTGATGAGTTGTGGTAGTAATGATGGTGCTTTTAGGTTT

GTGGGGTGGTTGTGACCTAAAGGGAAATGATGGTGATAGAGAACATAATAGTGAGACATGATGATGATTT

TTTGGCGTTGAAACAGAGGCACCTAGCATAAGAGGTTCAAGGATTTTCTTTATTTATCTCTGACATTTTT

TGAGATAGAAATAGAGTGCATCCATTTATCAAACCTAACTTTG

>Ca_linc_0268

GTGACTATCGGAGGTCGGAGTGGTTGTGCTAGTCATAGTGATGGTCGACAATGGTCGACAATGGTCAACA

TGGTGATCAATGATAAGTGATGGTTGTAGGGAGTGTTCAACATAGATCGCAGTTGTGGCTAATGGGGCAA

GAGTGGTGGCAGGTGACTGATGATTTTCGAAGTGGTGGTTGATAGTGATCAAAATGGAGATCAATGGTCG

GTCAAAGAAGTGGCTAATGAGTGTTAGAGTGGTGGTCACCAAGTGCTAGAGTGATCGAAGGTGGTAATTA

GAGTAAACTAATGGTTAATGGTGATTGAAGTGGTGGTCA

>Ca_linc_0269

TTTAAATCCAACTTTTAGTATGTTTTTTTAACTAGTTTTGGAGTTGACCAATGACATTACTATGTGCTCA

ATTCATCAACTATCGTTACCATGAGGAGTAAAAACTAGTTACATGACAATTTTGACTATCAACTGTAACA

ATGGATTACATTAGTCTTAAAGTTCAATTTTTAGAGAACATAATAAGTTGCACATTAAATAAATACGTGA

CTATCTATAAATATGTGAATATCTAAATTA

>Ca_linc_0270

CTTTAATGCAAATGAATCTACTTGTAACTCAATAATTTTGTATTCATGTGTTCATTTTTTCTTTTTTGAC

ATTAACAAATATGTTGTCGTTAAATGTCATTATTAATGCCACTAAGCACGTAAATTCATTCAGATTGATA

CAATGCTTAAATTTTGTATTATATACAAAATCATATTTTAGAGAATAAAAAAATATGAAATAATTTAAAC

TACTCGAGATTACTCGAGGTTGGAGTATTTCAAGAGACAATGAAGAAATTCTTGCGGAATCAAAAATAAG

TCACAGTGAAGGCTAACAAAAATATTGTTAGTGCTACAAACTCTATTGATGAAGATCATGTAAGGACAAA

TCTTGACCTATTCATATATGCTAGACAAAATTTTATTACAAACATATTGTTAAAAAAAGTTAATTGTTGA

TTGAGAGATTGTACTTGTTGTCATGAAATCTGCTAATACAAATTTGGTAGAGTCGATTAGAAGTAGATAT

GAAAATCTACTAATTTTAATATTTCCTATGAAAATAAAAGATATCTATAAAAAAAATGTGATATAGCTGT

GGTCCTTGGTGTCACTAGTGACTACAAAAATTTGTCTTTTCATGTTTTTTCAGTCATAAGTTACATCATA

GACCACAACTATATCACGTTTTCTTTGATCGTCATTTTCAGCTTTCATATCTAATAGTAAAACCAGTAAG

TCTTTGTATCTATTGTCTATCAATTCTATAAAATTTTGTGTAGTCAGACTCAAGTGCAAGAGGGGTGTAT

TCTATTATCCTCTACAATCTTTCAATTAATAGTGTCTTACATTTTTTAACAACATTTTCATAACAAAATA

TTGGCTGACATGATTTTTTTAACAACATTTTCGTAACAAAATATTGGCCGACATCTATGACTAGGTGAAG

AGATGTAATGATCTTCCTCAATAATGCTCATAGTCCTTAACAATATTTTCGTTAGCCTTGATCATGACTT

TTGCTTAATTCCACATGTGACGAGCCTTTCTTCATTTGCTCTTGATATAGTTCAACCTCATTTGAAACAC

GTTCTTTCTTCACAAAAAAAATGTTCTTCCTTTACAACAAAAGTATGTATTGTGGCTTGCAAATCATCAA

CAAATAGGCAATTTAAGTAATTTCAAGCAGTTTAGGTTGTATCATACTATTTTATTTTCTCTAAAATGTG

GCCATCAAATGAGTTTTGGATGTAATATAAAACTTTATCATTTTTTAAAATCCAATCTGAATCATTATGG

TGAAATCTCCTACAATAACATTTTTCCAGTGATACACAATAAGCCAATAATCAATTTTCTGCATTTTGGA

GTATTTTTTCGTGACAAGAAGTTGAAGAAGGTTAGGTACTTATTTGGTGTGCAGTTATTTTCACCCCTTT

TGATGTGTTTTATACTTCTAAGTTTGTTTTCCTTTACTTCATGATTTAAGTTGGGCTATATTTATAAGTT

TTTCCATTTATCTGTAATATAACTACAAAATGTTTATTTTTTTCTACAAATTAACTTTTGTTCATTTTTC

TTACTGCACCTAATTTTGTACTGTGTTGTGCTATCTTTTTTAGCTACATTTACAAAATCTACACCACATA

GTTATAACCACTTCTTAATTTAATAATTTATCTACCAAGGAAAGAGTCAATGACATTCTCATTACTAATC

ATGACTTATGGCTCAAGGAAAAAAAAAAAAAAAAAAAGTATACCAGCATCAAGATAAGCCCCCTCGGAAA

GCTATGAAGCATGTATACTGACATGTACACTAGACACGGCACTGACACCAGTAATAATTTAAGAAAATGG

TTTAATTGAATGTAATCAAAGTGTCGGTGGTGTGTTGGTGTCTGACACCGACACGTGCTGGACATCGAGA

CACGCCTTATTTCAGAAGTGTCGGTGCAATAGAGCATATGACATAATCTGGCATCTATCCCATTTTATTG

AATAAAATAAACAAGTTCCAATCATGAATGTCATCTTCCAATCTACCCTTTAACCCCAAATTGCACTTTC

ATTCTTCCCAAACATATTGCACTCTATTGATGTTGAAAAAGCTCATGAAGATAGCAAGTGAGGATAAGTT

GCAAAATAGAAAAGCAAGTGAACTGATGCAACTACCACAAATGTAATGTTCAAAGAGGTTTTCAAAAACC

TCTTACAAGTAAATATTACTTTCACTTCCAAATAAACTTG

>Ca_linc_0271

CGTTGATTAGCGGCAAAGAGAAATTACTCCCTACTTATGATCACAAGTCTAACCACACTAAAACTACTAC

TAACCACTACTCTAACCACTGTCGGACACTACTTAGACAACCACTAACACCACTTTAACCACCGTCAACC

ACCATCAGACACCAATTTGACCCTGCTAAACATTTTTAATCATCAATCTAACTTTCGTCGACCATCACTT

TAACCATTGTAAACAACTATTTTAACTATCGTC

>Ca_linc_0272

TGAAAATCAAATAATGAAAATACCGTATCGTTTCTAGGATTATAGTTTGATTCATATCAACAATCTCCTT

CATGAATTGCAATATATAGTACCCACAGTCGATGTTGTTAGTTTGACGAGGGCACTATAAAATAAAACAT

ATAAGTCAATAAATATCTGTGCATTGATAGCAGTATAAGTTCAACATGCATTTTAGTGAAACAAAAAACT

AATAAATATAATACCTGAAAAACAAAAGAAGAATAGGGTTACAGCGCATAAATACGAACGGTTTGTAAGA

CAGTAGGATTCGCAGCAACAAAGATGGTTCGCGATGACAAGGAGAATGAATAGGAAACAAGCGTATGAAG

TTTACGTAATGAGGGTTAGTAACAAGACAATATTGTTCGTAGGGTTCACAATGAGAAAGAGAATGAAGAG

GAGGAGGAAACAAGCGTACAAAGTTTACGTAATGAAAAGAAAACTGAAGCAGTTTAGGGTTTAGGGATAG

TATTTGAATGAGAGTTGGTAGGGATAGTAACAAAGGTGGAGATGGTTTGCAATGGAGATGGTTGGCAATA

TGGTTCGTATGGATAGTAGGGTTCGCAATGAGAAGGACAATGAAGAGGAGGATGACTCTGAAGCGTAGTG

AAGTTTACGAAATGAAGAGGAAACTGAAGCGGTTTACTGTTATATATAAAACACTATTATAGCACTCATT

GAAAGAGCGATGTAAAAGACCTCCTTAGCTTAGTTTTTAAAAATCTGTTACAGCGCCCTTTGAAAGAGCG

TTGTAAAAGACTTTCTTAACTTAATTTTTAAAAGCCTATTACAACGCTCTTTGAAATTGCG

>Ca_linc_0273

CAATTTCAGTTGCCAACTTGTCATTACCACGTCTGGTAGCATAACCAATTTTTTATATTAAAGCTTATCA

GAATGCTTTCTCTGCTTCAAACCTCATTCGACTATTTTCTGCCAAGAAAGATCTTGAAATCCAATTGGCT

TCTCCTTCTCAACCAGTTATCACAGATACCCCTATGAGAATCATCCATGTGTGTGTTTTTTGTCAAATAT

AAAAGGGTTTTATTTGTTTCTCATTTTAATAGAGAATGTTGTCGTCCACATTTGGGGAAAAGTTCCTGCA

AATTTTATAGCTCTGTTATTATGACAGTATGGACTATTTGATTGGTTAAACTAATCATTGTTTTCAGCAA

GAATAATTTATATATTCTTGTAGATTATTGAGCCTATTTTCAGCTTGTGATAATACAAATGTCATCTAAT

GTCTTGCATGTGGGAATGAGGTCATATATACAGCCAAGGATGACTTGCCGGCGTGCTTCTTTTGGTAATT

TTAAGACCTACTAATCATTTGGATAACCAA

>Ca_linc_0274

CGTACACTTCATTAACGGAAAAAGATGATGACCAAGACGCTTACGCTGGAGCCATACTTGTGCGGTAGAA

GAATACGATTCTGATTGAGACTAAAGTGTAGAAGACAACACCTTCTGACCAGTTTGTGCATTAGACAAAT

AGTATAACGCTTCTTGTTCCTTAGCAACTTCAATTGCCTTCCATTTTGTAAGATCTTAAAAGGTACAATG

ATAATGCAAGAAGACTATTGTGCAATTAGAACATGATTTTAACCACAAAAACAAAATGAGGAGAAGTTTA

ACGGCAGTTAGCATGGCAAAAAATGGCTTTTAATGATGGTCAACATGGCGACATGTGGTTGTCACACGTT

GTTGGATACGCTGCGAGTGGTTTTCACGTACGAGAGCAAAGGCGTTCGTGGAGGAGTGTGGTTGGTTCGA

TGGTGGCTAAATTTTGTGTCCTGGCTCAGTTTTTTGTGGGGTTGAGATGGTATGGGTGGTGAAGCTCAAT

GAGTGATGGAAAGGG

>Ca_linc_0275

GGGGAGTTTTGTAGAAACGGAGAAAAATTTTAAGATGCCAAATTCCTGGATAGAGGTCGAAGCAACTCCT

TTACTTAAGTCACAAGTAAGGAAGACATTCGTTGAGATAAGATTAGAGGCTGCCTAAAGGAAGACATTCG

TGGAGCTCATTACAAAAATGTTGAATAATCCAACAAGAATTGACAATCATCGATGAAATAACATCCTTAG

TTTGTGATTCCCTAGATTTTTCACACAAAAAAAAAAAAAAAAAAAAATAGATCTCTCAGTCGCAACATCA

ATCAAGTGATGAAAAGCGAAGCTTAGCCAAGATCACAATAACACAACCCTTACTTAGGAGATTATAGGAA

GTACAATAATAGAAAAAAGATTTTGGTTCGTTTAGGCAACAAAGATTCTAAATAAAATGAAATGAAAAGA

GACAATAACACAACCCCTACTTAGGAGATTATATTCGGAAATGAAATGATTCTAAATAAGTTTGAGAGAA

CTTGAGTTTGTATGCAGCCACCTTTTATCAAGTGTCTGGTCAAGAGGAACACAAACATCGTCCAAAATTA

CGAGGTTTGGATACATATGTATTAAGAAATATGGAAGGAGCCCCTTACGGTCTTTGATAAAATCACTAAC

CTTAGCCTCTAAATCGTCATGAAGATTAGAAAAGATATCAAAAAAAAAAATAGATACCAAATTATTATAA

CTCATACGTTAGTCCAAAAATTTGCCTTTTTGGCCTCCCCAATTTGCCAAGCGAAATTATCCATTACAAA

AGAGAACATGCTCTTGATACTATCCCAAATAGAATAGGAAATATGGTACGCAATGGGAGAACTTTTACGA

AGAACTCTAGCTTGGAGAAAGATAGTCCATTGATTATGAGAAGTATAAAACTCCAACAAAGTTTCAACAT

AGTAGTATGAT

>Ca_linc_0276

CGGGAGGGAAAAGCGGTGGCGGAGGAGCGGTAGTAACAACAAGAGTTACACCGGCATGTTTGGGACTGTG

AAGTTTCCGTTACAAATGGATCTGAGTGATATTAAGATGAGGCAGGAAAAGAGGGAGGCAATGGCGGCGG

CGAAGTTACCGGCGGAGGGTGGTGTGAGAGAGAGTTGTTGGGAATTGGTAAGGCCGTTGCGGCGTCGTGG

ATCGCTTTTGAGGAGCTTGAAGTCTTCCTTTAGATGCATTTCTATGGCTATTGACCCCAAAAAAAGAAGC

ATTTATTTGGCTTAATTTTTTTATTTATTCCTTTTCTATATTAT

>Ca_linc_0277

CAGAAATTAAATTCATAAAATTACCTCTAACTATTGCTAAGATGGAGAACTTCAATTTCATTTGTTTTTT

TTTTTTTTTTGTTTTTTTTAAGTGTTCACTCTTTTATCATAAATGGTGTATACCAAAACGGTAGAAAAAG

CCTAAGAGATATACAATTATTTACCGATTTTTCTACCATAAAAATAAATTCATATCCATACTCACTGTCA

TAATCTATTTAAATTGTACATTTTTATATTTTTTTATTATAAAAAATGCATTCATATCCATTACTTACTA

CCATAATATCCATTCAAATCGTACACTTTTATATCTTTTTATCATAAAAAATGCATTCATATCTATTAGT

CACTGTCATAATATCCATTCAAATAGTAAGTTTCTATCTTTTTATCATGAAAAATGTA

>Ca_linc_0278

ATTTTAATGACTTCTGAACCACCACATTTAAATATGGTGGATTTTCAATTACAAACTTTTGGATCACTTA

AATATTATTTTAATAATAAAATTATAAAAATTGTCAGCAACATAATATATATGACTCGATAAATCAACTA

TTGAAGATTCAACGGTTCTATGCAATAGAAATATTTGTTTAAAGTTAAGGATATGTTCAATTTACAATTT

TTATAACAATTTACCAAACAACAAATCATTCAAAGAAAGATTAAAAGAAGAAATTAAACCTTCCAAAAGA

AGAGTAAAGAAATATGGGTTTCTATGAAACTGTGTGTGCATTCTCTATGTATAGTATATCCCTTTTAATT

TGGAATGCATTGTTGTAATTGCAAAATATACACACAAATATTTTTGGATGTTAATACATTATTTTAAACT

AAAGATACATAAATACTTACCAGGATTTTCTCCTTAAAAAATGCATTATATTTTTAAGAAGAATGTTTTC

ATGAAAAGTTAAGAAACTTGGATGTTGACGGAAGATCAGAAAGCACAAGACTGCTCAAACACAATTTTTT

TGTATTTGTTCAGATGTGCACTCTATCTTCTGTCAACAAACAAAGTCGTAAATATGATACGTCTTCAAAC

CCTTTTAACAAAAAAAAAAAACATTACAAGTATAGATCTTTCACTCATTTACCGTCTACTTAAATAAAAC

AAAACATTTATTAGGGTCATAAGCTTCATATATGATATGATAAATTAAAAAACAATATACATGATACCTT

AGAACAAGTTGTTGCCTTTAGAAATTTCAATCTCAAACAAGAGGAGGTAGAAAGAATATTGATAGAAGAA

TTTAAAAGGTATAACAAATGTGCTCTTGGGGGTGAGAAATAATAAAAGGGAAAAAACTTAGAAAGTGCTA

GAAGAGCTATGTCTATGGTTAATGTAGGAAACAAGTTGGAGAAGAATCAAGGTTTGGTGTTGAAAAAGTA

GTTGAGTTGAGAGCAATAGATTTTTTGAAAATTTGGTAAG

>Ca_linc_0279

AAGGGAAAACTCTTTCATGTATCTAAAGGCCAGACACCTCTTTGATCGCAAAAGCCGTCCCTACTTTACT

AACCTTTACTCAACCAAAATTTTCCGAGGAAACATAACATATCCCAAACATAACATATCCCATCCTAAAC

AATGCATGCCGTCTAAATGCCCTTCACGAGTCCTCAGGTCAGATAGGGATACCCACTTAGTTTTACGCAC

ATTAATAAGGATTTCCTTAATAATGATGTGTGAACCAAAATAAGTCCACCATGAGAATAGGTAAACTCGA

CGTTCAAATTGTAATATGGAGAAGCGTTATGAATCGCATACCGCGTCCAAGTCTCCCTAAAACATACTTC

AATTGCTAAATAACATTGGGATAAATGGGTGAGTGTTTTTAAGTAATTAAGAGAGATTTGTCCAAAGTGG

TAGGCCGAGAATGGAAGGAGTTAATTAAGAGTGTCTTTCGAACCTTCACATGGGTCATTGAAACATAATT

GTGAGGCTACATTATGTTACCA

>Ca_linc_0280

TAAAAGGCCAATATCCCGATGCAAGGCAAGAAATTCAAGTACTTAGAAATCCATAAATCAATCACATACA

TACTTGAAATTCTGCAAAACAGAGGCAATCATACATTCTGATTTCTGCGAAACAGCTGCTGGTTCATACT

CATCTATTAGTTTTCGCAATTATCATTAGATCCTTTGTAAAGAATCAATTCTCAAACAAGAGTTGATAAA

TCTCAGATTCTGTCAACACCTAGTCTTTATAGTTTGTTTAAGGTTACATTCTATCAAGGTTAGATAGGTG

CTGGTATTGCTTTCTTGGTGGAAAGCAATAGAGGTTGAAATTGATCAAGGTTGATCTAGACTAGGTGTTG

TAAGATCAAGAAAGCTCTTTGTTTTAAACACTTAGTGAAAAATCTCACAGTGTGAGGACTGGACGTAACC

CACGTTGGGTGAACTAGGATAAAATCTTTGTGTGGTATTCTTTATC

>Ca_linc_0281

TTTTGGTGTAGTGGAGGAAATGATGAGATTCCTTTTGCTTTCGGTGCCACGTTTGATTCCTTTTGGAATA

ACAATGTTGGCTTTTGTTAGATCAATTTGTAAAAATAGCGTTTCGCCCTTTTTGTTTTCAATATTTTTTG

TTGTCGTAAAGGATGAAAAGATTGCTTTGTAATGGACCATGTAAATAATAGCTACAGAAATAGATACTG

>Ca_linc_0282

ATCCAAACCAAATTGGTCCTTGACATAGACTAGATTCAACTGTACTTATTATTGAATCTTGAAAATTCAA

GAACCTTGCATCCCGCATGACACATATGATTGAAGTGTTTAATCCTTCCCTTGTTAGAGGTTTGATACCG

ACTTGAACAAGTCCTATATGTATGAAATTATAGTTCCTGTTCGTGTGTTTTTTGAGTGTTTTTGGAGAAA

GAAGGTTTATTGTTTCAAAGGGGTTTGAAAGAGCAGCATCACGTTC

>Ca_linc_0283

CTCACACTATGAGATTTTCCACTAAGTGTTTAAAACAAAAAGCTTTCTTAATCTTACAACACCTAGTCTA

GATCAACCTTGATCTATTTCAACCTCTATTGCTTTCCACTAGAAAGCAATACCAGCACCTATCTAACCTT

GATAGAATGTAATCTTAAACAAACTATAAAGTAACTCTTATAATTTGAGTTTTTACAATTTGATTGTTTT

GATAGAATCTGAGATTTCTCAACTCTTGTTTGAGAATTGATTCTTTACAAAGGATCTAATGATAATTGCG

CAAACTAATAGATGAGTATGAACCAGCAGCTGTTTCGCAGAAATCAAAATGTATGATTGCCTCTGTTTTG

CAGAATTTCAAGTATGTATGTGATTGATTTATGGATTTCTAAGTACTTGAATTTCTTGCCTTGAATTGCG

ATATTGGCCTTCTATTTATAGGCTGTAGAGGCACTGAATTAAGGTACAAAAGCTGTTGTAG

>Ca_linc_0284

CATGGGTATTTCAAATTGTTTACAAAAAAAACCTAGTTCTTGTCTAGTTCTTTTCATTTCCCATTTTAGA

TGTTTTTGGAGGTTTAAATCTTGACAGATTTTCAAACCTTCCTTTTGAGTAAAGCTAACCAATTCCCCAT

AAGTTAAATGGTTATATGGGATTTGATTTTGTTTCTAACCTTTTCTCCTAAAAGGGTTGGGAGTCCTGCA

AGGAATTTCTCCTTCTAGAAAGGTTGGTTTGAGTCTTCTCTTAGCATAACTCTGGTTAGGAAAGTATTTT

TGTAATAGTGGAACCCAGAAAGTTTTTTACATGTAAGTGTTGGATAGGAGTTCTGCATTTTTATCTTTTA

AGTGTGATGGATCACCTATGAAGTGCAAGCTAATTATGTGTATTAGTGTTGCAACTGCATCAGGTATTGG

GTTTCCTATTTCTTCGAGGATAGGAATTCCTTCCTCTGTGGTTTGAATTGCGTTCAAAATTTCTAGGTGT

TGAGAGGGTGTGAAATGGTAATCCCATCATCCTTTGAGTTGGCCTGAGAATCCTGCTATTAGTAGTTCAG

CTATGGCTTTGTTAGGTGTGTTGGCTTGGGTTTTGTAAGCATTTGATGCCATTGTCATTTGTTGGAGGAG

ACTTAGGATGTTGTATTCGGACATACCATATATGTTTCATTCATAGACAGTATTTGCATTGAATTTGGAT

TGAGAGAGGACATTAGGTCTATTTTCAATACCTAAGTCTGGTGCCGTGACCATGGCTAAAAGTTGACCCT

TCTGCCATTGAAGTTTGCACAGGTTTTGTGGTTCGTGTTCGGTTTCAGAAACTTGGTCTAAAGTCGTAAT

TGTTTTGTCAAAGGTATCTTTAGCTACCATTGGTGTGGAGGTAGAGGAATGTTCTATCCTACTGAGTTGT

TCATGTAGGGATTGGGTGAATTCTGATCTGCCTTCCCTAAACAGTTTTTGGCTTGTTTTTGAGATTTGA

>Ca_linc_0285

ATTGCATATAGTGAATGAACAATGCTTAAAACCAATAATGCCTAAACAACAACAAATCATACAATAAATT

ATTCGATAAAAACAAGAAAGCTTCATATGAACAATAACAATCAAAGAATCATAAACCCCAAATTTGTCTT

CAACTTCAGTTTCTTCATAAAAGAGTATATACCACAAAACCAATCTTATAAGATGTGCAACCATAGATCA

CAATTGGAAGATTCAACATGGATGTTAAGAGAGAAATTGAATACAAAGTTTCTCATTTAGCATTGCTGAA

GATACGAAAGAAGCTATTGGATTTTTCTCGAGAAAAAATGGAGAAAAAAATAGAAAGAAAGAAGAGGTCG

TGGGAGATTGAGATTTTCTTCAATAATAATTAAGATCATCTCTTCAAGGTGGGTATCATGGCTCTAATAT

TATGATGAGAAGCACGCAGAATTCAACAACAACAAAA

>Ca_linc_0286

CTCCTTTTCCAACCACAATCACCGCCTCACTTCAATCCATTTTGCTATTTGCACCCTCCTTTTCCAACCA

CAATCACCGCCTCACTTCACTACTACTACTACTACTACTACTCCTATTTCTTCTTCTTCTTCTCGATCGC

TGAGCAAAGGAACAATACATATAGCTACAACGATTCTTCAAAGGGAGTGAAATCCAAAATCAGAGAGAAA

GAGATAGCGACTTTGTTTTTATTTATTTATTCATTTATTTATTTATTTATCTTCTTTTTCTATAATTTTC

ATTTTGAGAAAAAATAGAAAGTGCTGAATCTGTTTCTGTTTTTGGGATCCTTCTTGGCTTTGGATTCAAC

GGACTTCTCAGATTTGCCAATTAGACTGAAATTAGGGTTTCTGTTTGTAGGTATTCTTCTTTCTCCTTCC

ATTATAGGATCTTGTCTTCTTTTTTATTTTTATTTTTTATGTGCGAATTATAAATATCTTTTGTACGAAT

CAGCGACGAAAAACACGTGGCAG

>Ca_linc_0287

ATTCTGTACTGGTTCCATCCATCTGGTTATGGTTCTGGTTTCTAATTCCCTCTAGTTCTGGTTCTGGTTA

TGGTTCTAGTTTTGGTTCTTGTTCAAGTTTCCTTTGGTTGTGGTTTTGGTTTTGGTTCTTGGTTCCTGGT

TCTAGTTTCAGGTTCCCTCGGATTCTAGTTCGTGATATTGGTTCCCTTTGGTTCAGGTTGTCTATAATTT

AGGTTCTCTTTATTTTTGATTCTTGGTTCCCTCTAGTTCTAGTTCTAGTTCTGGTTTTGGTTCCTGGTTG

TAATTCTGGTTCTGGTTTTTGATTTCCTCTAATTATGGTTCTAGTTCCCTTCCCTTTGATTCTAGTTTTG

GGATCCTCTAGTTTTTGGTTCCTTTTGGTTCTGGTTACTTCTAGTTCTGGTTCCCTCTATTCAGGTTCCC

TCTCAGAATTTTGGTTTTAGTTCATATTCCTCCTGGATTCTGGTTCCCTCTAGTCCTTATTTCCTCTCTT

TTTATTTTTTTTTATTTGGGAACATTAACACTCCCTTCCATCTTTTCAGCATCCTTGCTTTTCCCTTTTT

TGCAGCTACGATATTTTTCCATATATACACGCAGTTAAAGAAGTACATAATCTTTTTAGCCACCTTTTCA

GCCTCTTTTTAGCTTTTTCTTTTCTCCTAATACGGCTATAATTTTTACC

>Ca_linc_0288

GGGTCATCAACGTTTGATTTTTTCGGTAGTTGAACACATTTTTCAATTCTCATGAGTTCATCACACAAAT

ACATCCTTTCCACAAATTGGATTTTTTTCCATGATTTTGATTCTAGTTTGTGGTGCATTCTCCATTCTAT

AGATGTCCGAGGTATTTGACAACTGTCCTTCAAGTATACATAAACAAAATGATTTTAATTTACTCATCTA

AAGCATAAAATACACTAAGTTGGATATGGAGGTGGATGACCTCGTAATGGAAGAAAAATTTCAGAAACTC

CATCTTGAAATCTCGTCAATTCAATTACAACTATTGTATTGAGTTGCAACATGATGTCCCATTTTTAGAA

ACGACATCCACTTTTTTTTTAGGTGTTTTATAAAACTTTTCAGGATACATAACATCTTTGATAACATTTA

ATCGTTTCTCTAACGAATACAATTTGACATAATCTGACTTATGCTTATTTATCTTTCTAATGAGATCATG

TCAGAAAATTGCATGATTCTCCTCACTCTTACCAAGTTGTGCAACGACAATCTGAAATCCACAATTCCCA

TCACCAGCAACAATTTTGTTAATCTATGAATGCATAAAAAGAGACATATGATCAATAAACAAAATTTTTG

GTTTAGGGAATTGATTAGGTGAGAGATTTCCAATATGTGCCCTCATCTTTCTTGGACATGATAACTTCAA

TTTAGAAACTAGATTGTTCGGATGTAGTGAATCAACATGCTCCATAAAGACGACAAACATTTAGTTGACT

CATCTGTTGGTGTATGTCTAACCTTTTTTGGCGCACTCTTGGTTTTCACCTTATCACTAGGTGGATTTAA

CGACGTCGTTTTTAGGAAAACTATTTCACAAATTTGGTCCTCGATAAGTAGTTTCATACCGTAATCCATT

TTGTCAAATCGTTCTTGAAATACAAACGTGTGCCCAAATTTTAAATACTACCACATAAATTCACTAAATT

AACAATGATTTAATTTAATTAATTAAAACATACGTGAATCGCTTTCAACTCTGTTAAAATAGAAACATCT

GAAACTCCTTCTTCTTTTATGATAACATTATCATCAAAGCAATTCATCTTTCAATGAGTGTGTACCTCAT

CAAATTGAATAAATGCGTCATGCATTACTTTCTAAGCAATGATACAAACACATGGAAAATCGTAAGTTTT

TCTAATTACACAACTACATTTTCTTTTGTCCATATTGCATTCTTTTGCTCGTGCTTCTTTGCAATGAATA

AAACTTAATTCAACTCTTGAAATTGTAAACACCTAATTCAAATACAAAACTTGTTCTAACACACTTAAAC

TCCTTACAAACGAGGTTTGTATATAAGTGAATTTTGTTTGCAACATATTGTTCATCGCTTTCCAACCCTT

CACCAAATCACCTTTACTATCCAACAAATATATTTTAAACCTACCGTGAGTAAGTTCAACTCTTTTTATG

GTTGTGAT

>Ca_linc_0289

GCTTTTGACATATTTGAGGAATTTTGGAAATTTTTCATGTTTTTTGAACTCGCATTGACGCCAACATATG

CATCATGTCAAGGAGATTCCATTACGAAGTCCCAAGCCCCCATAATATTTTCGGTTTGACTTATTTTCCA

TTCGCATCTACATGTTCCGCGACTTCTACCTTCACATCTAACTTAGATCTAGCTCTTAACATTTTTGAAA

TATGATAACGACACAATAAAATAGTAGTAGTTGGAAAAATTGTTGCAACAATATTCATTAATACAATATC

TCTGTCAGTCACAATTACCTTGAAAATCTTGTCTTTAGACTTCAACAAATTACGACACATCTCTAGAAGT

CAATGGAAGTTATCCTCCTTTTCAAATATGGGAAATGCAAACCCAACTGAATATGTTACCTCTGTTGAAG

TCACACCAACGATCTCAA

>Ca_linc_0290

ATTTCAATAAACTTACATTTTTTTTCTTCTATATTTTGATCATTAACAAATGCTCCAAACACACAAGAGC

ATTTGTGTGATTCTTCCCTTTAAGGTGAGTTGTGGAATATTGTCAAACTGACCACTGAAATTTCCACTCA

ACATCAAAAGATTAATTAGGAAGTGTGTGTGCATCAGGTTATCCATTTTGACCATGTTCGGTTGATAAAA

TCAAAAGTGGTTTGAAAGTTACAATTGACAAAGTGTTTTACAGTATATGCTCTTGTATAATTAATTGGAC

GGTCGACAGAAAGAAACAGC

>Ca_linc_0291

TCTTATCAAATTTTACATAATCTAGCACGAGTGATTTAGCTCATAGTTAACAAGACATAATCCATACAAA

AGGAACAAACTTATACATCAATTACAACTTAAAAGAAGTGAAATATAAATAACCTAAATTGAACTACCAA

AAAGAACTAAGGATCTGTCAACTGCCAATTGCTCCAGCAGGTGATGCAACATGTTGCTTTGGTTTGTTAT

GGAAGAATGAGAATACATGGTAATGTATGAAAATAGAGGTTGATTAAGGAAAGTAGCAACCACATTCAGC

TATATGATAAATAATCTC

>Ca_linc_0292

TTCTGAATAGTGGAGTTATTATGACAAGGAGAATAAGTGTGAAATACAGTTATATCTCCAGTCATGTGAT

ACAGTTATATCTCTATGATCACTGATTTTGATTTTCATGTGCTAGTGTGTAAAGCATGCAAAAAATTACA

TAGTTTGTTGGAGCATTTTCTGTAGAGCTTCCATTTGTTATTTGATGAAGGGAGATAGATCATGTTTATC

CTTTGTTTCTTGAGAACACTTCTTGGACTTTTTGAAGTAGTTTGGTTCCAATCACCCTTCCACGCACTTC

ATCAAGATCTTTGTCAAGTCTCAAATTTTGTTTGTAGATTATATCATTTTCTACCAGCTCTTTGTACTCT

TTCTTGTCATCCGTATAATTTCATTGCTGCTAATATCGACTAAGGGTGTTGAAGTATTTTGGAGGTTGGG

TTATCTTTTCTTGGACTATTTGCAGTCTT

>Ca_linc_0293

CGTACACATATGTGAACTGAGTTCACGTATTGAGGCATTGCCTTTGATCAGAGGCAACACCTACGTGAAT

TGGGTTCACGTTCAAAGACATTGTCTTTGATCAGAGGCAATATCTACATGCACTTAGTTTGCATACAAAG

GCAACACCTATGTGAACTGAGTTCACATACAGAGGCATTGCCTCTAATAAGAGGTAACACCTACGTTAAC

TGAGTTCACATACACTGGCATTGCCTCTTATCAGAGACAATACCGACATGAACTAAGCCCACGTACAAAG

GTAATGATCAAAAACACTATCTATGTGAACTGAGTTCACGTACAAAGGCATTGCCTCTAATCAGAGGTAA

TACCTACGTGAACTCAATTCGTTACGTGTACG

>Ca_linc_0294

TATGTGCTTGGGAACTTGCTTTGCTGTTATGGTTGAGTTTTAAAAAATTGTTATTTACAGAATGTTAATC

TATCTACATACATAATGACTTCAATAGTTCGCTTGACCGTAACACCTCTCTTACACTAAGTTCCTGTAAA

ACCCAAACTCCACTACTACAAACAACCATGATCGTCCTTTTCTCATTTCCATAGCCATGAGAAACAACCA

CCTAATAACCCATAACTCATAACCCCCATTAACTAATCTTCAATCGCCAGAAGCATCCTCAAACAACCTA

GAACCATAGCCAACACCTCTTCGCTATGAATCCAATTCCTACCGCACTCTTGCAGTCACGAAAACTTTAA

CCTACCGTCGACCTAACTACATCTCGCACCATAAAGGCAATACCTTAGACACCAAAACTCAATGCATCAG

AATCGTTCTTCCTCACCTCACCAAAACACTTCTTCACCATAGAACCACTGCGTCTATCTTCTTATGCACC

ATAGTCTTCGTTGGTATTAGGATCACACCCACCTCCCAAACTTGTGCATACATTACCCACTGTTGTGTCT

ATTGTCATGTTGCCACCGTCCAAATCACTGACCAACCACAACCAAATAAGTCACGTCATTATTGATGTCT

TCCCTCCTTTTTCCCGTAAGCCAGCAACCATTGTTGATTGATTGTCGAAATTGGATTCATT

>Ca_linc_0295

GTTTAGTTTAATTTATTTTTATTACTATTTTATTTTATATGTTTTAAATTTTTGTTTTGTTGTCCATCTA

GCTTCTTCCAAACACTATCTAGAACAACATCAAACACTTATTATTTCGATTGTCCAACAAATGATTAAAG

ATTAAGTATTCGGAGAGATTTTTTATCGTCACAAAAAGAATTTATCTTTTAAAATCAATTTATTTGTCTT

AAAGAAACACTAAAAGAAATCTTTAATTTATCTAGTTTGATGAATTGATCATTAGGGTCTTGATAAAGTT

AGAGAAAATTTTGAAAGCACTCAAAAACATATTGGTAGCTTTGCAAAAAAAATGAGACTAGCATCTTTAG

GTAAATATATACTATAAAATTGAAAACAGTTCAAATAAAATATATATTATATAAAAAAGTTTATATGCAG

T

>Ca_linc_0296

CTGATGGTTTCTAACTCATCTCATATTCAGGATACAAATTTCAACTAGAGAGATAAGATAAGATAAGATA

AGATAAGATAAAGTTCTAGATTAACTTATCATATCTTGTTGGTTTATCAAACACTAGATTCAGATTGAAT

ATGATAATTTATTCATATTATGTCTCTTATCATGTCTATACCATAAGATTTTTGGTTAAGATTTGGTGTC

AATGTCTCTTAGGGATCTTGGATGTTTAGTCCATTGCTGCTGCCACACTTTCCCGAACTCTCTAACAAGT

GGTATTAAAGCCTTGATTCAGCTTTGTGGGGGAGCATGAGTGAGAGATTCTAGTGTGAATCAAGTCTAGT

GATGTGGGTGACTCACACTTGAGAGGAAGATTATTGGAGTTCAAGTGTGAATGGTTAGTCCCACATTGAC

TAGGAATGACAGAAATATTGGATATATAATAGAGGTGACCCATAT

>Ca_linc_0297

TGCTCTTAGTGCGCTCCCACAAACTCTTAAAGTTTTGGAGATCTTGAAAGTGCCAATTGTTGATTGATTT

ATCTTATGTTCTATTATGTTGATTTGTCGATTTTATTATGACGTTTTATGATTGTTTGAATATGTGTTTC

TTGAATGATTGTTTTATGATGTGTTTTTGAATGGTTGCTGCTATTTTAGATTTGAATGAAATATTATATT

TTAAATATGATTCTATAAAGATGTACCACACATTTGTTTACTTGGATGAGCTATGTGCTCCATATAGACG

AGTCTTATGTCCCACATGAGATAAGTTTTAAACTCCACAAAGATGAGCCTTGTACCATGCAATAGATGAG

TTGGTGCACCATATGATGAGATGACACTCACTTCGTAGAAGTAGTGCATCTTCCTATAATAATGGGACAC

TGTTGAATTGATTTATTTACTTTCTGCAGAATCAAATGTGTGTATTTGATTTATTTGATTATTATAGATG

TGAATTATTGTTGGCTTTAAGTGGATTGCTTTATATTATTTATTGAAAGTAAATGGTTATTATAGAAAAA

TTATTTGTATTATTTATATTT

>Ca_linc_0298

CCCAATTCAAACAACTCACAAAACCTTATTTGATTATGAATGCAAGAAATTTACTCCCACTTCATTTCAA

AACCCATTTTAAAACAAAACCTCTAACACTCATTATCTACACGGAAAAACAAATTTCAAATCAAAAGAGG

GAAAAAGGAACTCACTTTATGATCTAAAACCCTTCATAATCTTGAGCTAAGACTTACTCTTTATCTCTTC

AATCTACAACCTTGATTTTCCTTTTCTAGTCCTCTAATTCTAGGAAATGGGGGAAAGATGGGTTTTTGAT

ATGATATAAGGTTTGCATGTATGATTATGGTGAGAATGAGGTGAAAAACTTAGGAGGCAGAGAATGAAGG

GTGTAGTCGAAAGGGAGAGAAAAGGAAAGAGCAATAGTGGTGTGATATTAGTGTGATGAAGATGAAGGGA

ATGAAGAATTGAAAGGGTGATGATGGTAGTGAAGTTTCTTAACTTTTGGGTTTGTTTGAAGATGAG

>Ca_linc_0299

AGAGCATCTACTTTATAACAATTGCATTAATACCGCTCAAAATAATAAAATAAAACATTAAATACATAAA

TAAAGAAGTAAATTTAATGGTCCCGAATCTTTTTAACTCCACTACTTTCTTTGCCATACCAATGTATTCT

TCTCGGCACCAAAATTTGGTGGACCATAACCTCACTTTAATTTCAATTTATTTGGTGTATGTTTAACTTG

ACCGAATTAAAGTTCTGTAAAACCAAATATGAATTTATATTTATATAGGGCAACAAAACTAATAGGAGAT

TACAAAATTATTTTTTTTCTTCCTT

>Ca_linc_0300

CTTGGAAAAAAACTTTCTCACTTGATATTAAACTAATAATGAAAGAAAGTTAAGTGATATTAATGAATCA

AATATTTATACTTCATAATTTATCGATCACTTACTCTAATGCTATCAATATTCATCATGGCCAATAAAAT

ATTAATTAGTCTTTCTCTTAATCCATTGCCTTCAATATCGTGTAAGTAAGATAATAGTAACAAAAAGATT

TAAGACGTGGCAAATTTGTTGATTTATCTGCTCATTGAATTAAAGGAATCAAGTTAGGGTCAAAACCACA

CTCAAAACCAAAATTATTAAAAACGACATGTTGGTTAATCACGGTACGA

>Ca_linc_0301

AAAAATTTAAAATAATTTTATAAAAAAGGACAAAAACACATCTCAAGTGGTCATAAATTTAGGGACCCAA

CGTGATAATTAATCAACTAACTAAAGGTTTATCACACCTTATAATAATAAAACCACAAAGGTAAACTTAA

CCCATATGCGGAAAATATAGATAGTCACCTAAATAATAAATATAGGATCCACCAAATCTCGAAGGGAGAA

TTTAATTACATATAGTGTTAGTAAACAAAGTACAAAATATATCAATACTCAATCTCATTGAAGTAGTAAA

CAAATTAAAACCCAAAAATTAGTTGATAATTCACTTTCAAGTCTTCATAGAACAACTGGAGCATTGGCAT

GAAGTGATGTACGACCGTGCCAACCCAAAGAGAGTTG

>Ca_linc_0302

TTCCATCTAAAAAAAATAGAGTATAGTTTATTTTGAAAAGGCAACTAAGTAAATTCATAAAATGTCTAAT

GACATAAGAAGTGAATAGAAAGATGAGGTGGGAGAAGAATTCCCACATGGCACAAGAACTACCAAAGTAT

AAGACCAGTCACCAAAGGTTCACTATATATATATTGATTCAAAACATGATTGCTCCCCAGACAACAAATC

AAACTACAGTAATCAGCAATCATTCTTCCAATCAGCAATCAGCAATCACCAAAAAATAGCAGTGTGTTGG

TTGCTTCATATTAATTGAGTTTGAATAAATATGTTGAAATCAATGGTTGGATGGATATATATACATTCAT

TCATCACTCAACAATTGCAGTCAATTTTCTACATCAAGGAGCGTAAGCAGCAACTTATTTGACACGGCAA

GTCATGAAAAATACATTATCTCCCACGTTCAAGGGACCATAGCTATAACTTCAGGAAATAAATACCAATG

GTTAAACACCTCACAAAAACCATTATTTTAATTATAAAATCCTAATCGTACTTAGGGCCAAAATCCTTGA

AAACAACTTAGGGTTGAGGCCCTCACAACATATCTAAGGCTACCG

>Ca_linc_0303

CAAAATATGAATCCAAAATATTTACACATCAAAAAGTGGTACATGTAACCCACCGAAAATAACATGCCAA

GCTGACAATATTAGTAAATGTACAGTTTTCCAGTTTCAAAATAACTTAATCCAAAAAAAAAGACATAAGT

TCCCTCTATGTCATTCTAATCTGATCATTCTTCAAAAAACTATAGCTATACACCCTGAGTAATCTCCATG

CGCCCCGTGAGATCCTCCTAACATAGCTCAAGTCAAGCATTCCCATCTACATTCCCGTCCGTAGGGTACG

AACCGGTAGGATCGTCATGGCACTCATCTGAGGGCAAAGCCCAGATTTCCACAATAGTTGTAAAGGGTCA

CCGACCGAAATTAACAGTTAACACATAACATTTAAGTTTTTAAATGCACAAAATAACCTTTTAACTAAGC

ACGCACCTTAAAAGGATTTCCATATGCTAAAAGTTCATGTAACACTTGCCAAATAAAAATGAAATCAAAA

TAAGGTTCTC

>Ca_linc_0304

CAAATTCCAAACTCACTCAAATTCATCTTCTCCCCGTCACAAATCAAAATCACAAATGCAAAAATATAAT

CATAGAAACTATCAAAATCATAGATATGTCCATGCAAACATGAGATTGAAATGAGTGACTCACATTAGTG

GGTTGGGACGTGCACGGGAAGGGAGACAGCTAAGGAGAGTATTAACAAAGACAAAGGGAGAAAGAGGGAA

GAGATGATGACCGATGAGTTATCACGAGATTTGAGAAGATACAAACTTTAAGATTAGTTATATCAATAAG

GATATTTTGATCATTTTTTCTGCTAGTTTTTTGAAAAGTTCTT

>Ca_linc_0305

TTTAGTTACTCATGACAAAGATAGAAAAGATAGAGATTAGAGAAGAATTACAAGAAATATTCTTGAATGA

TTTTTGATAAAACTACTCCAATGGTGTTAGAAACGTCCGTCTTTGAGTTTCTATGTTAGGGTACAAGTCT

CTCAACTTCCCAATAGTAAAAAAGATCCATAGAAAGTGAAAAAAAATGCATTTTAATGAATGTTGTTGCA

TGCCACGCGCCCCAAGCGCAGCTTGGCGCTTCTGAAAATGTGCCTCAGGTGCGACTTTTGCGCTTCAAGC

ACCCATCTTCTTCTATTTGACGTTTTCTGCATTGTTCTCCTCGTTTGAGTATGAATTGAGTTCTGGTGTC

TTCATGAAAGTTGTAGTTATGGATCTGATCGTTCACTTGCACTTGGTTTGTCTTCAATTTGACATATACA

ACTCCAGATATGTCTGAAATACTCTATATATATCATGTTGATTTCTAGCCAAAATTCAG

>Ca_linc_0306

GTGAAAGTAAAATTTGTCAAAAAAGGATGGATACATAATACACTCATTCCATGGATCTACTACAGCTATT

GCATACAACAAGGTTTGTCTCTTACCAATTATAGAACCCAGACCCCCCCGGCAAATATGGTGGATCGAAA

TCCCCAAATCAAATACAATATTTACTATCCCCAAATCAAGATGCTTTTTAAGTAGGCCTCAACATCACTC

CAATTTTTCTTTAATGCGTGGGTTCCAGTCTAAATATATGAGTTGTTTAATTGGTGGTTTTGCCTTCAAT

TTTGTCAAATCATCTCACCATGCAAAGTTTAGGCGGGTTTCAGTCGAGGTGAGTTTGTGGGTGATGATGC

ATTGCGGTATACTTTGATTAGGTTGAGTTATTTTTTGTCAAATCAAATACTCAAACCAATCAATTTTATT

TGGATTCAAGTTGAACTCTCAACCCAGGAGGAGCAGGTTGGTTGGATTCACTAATAATAATTTTTTATTT

TTTTTATTTTAAAAAATATAATTAAATTTAAATATTTAAACATCTAAAAAATAATAAAGTTATATCGCAA

CATATTATTCAATACTTAAAATACAACTACAAACACAACAAAACCCTTAAATTAATTAAATTAGCAATAA

ATTCTTAAACATTGTCCAACAACTAAATTTTAACTATACAATATCTCATATTAATGGGTTGAGTTCATAT

AACCATGAACACCCCTAGTGATTCAGGTGGATTGGTTCAATAATGTACTTTAT

>Ca_linc_0307

ATACTTAAAATCTTAAGTGAAGCAGTCAAATTCGAATAACCATGAGTTTATTTTTTTCCATCTACACTCA

ACCTGTATATATCTACATCTATGAACATGTGTGACATGTGACTTGCTACAACCCGCTCGCCTCTCAAATT

GGTCCATTTGAATGGTTCAAGTTCAATTAGGTCAACTTGTTTTACTAACCCTACACTTTATATTTCCTCA

TTATTAGAGTTTGGTATATGAGGTGAGGTCCACAAATCGATTCATTTAACTTGCTATCGCCGTGCATTTT

GAACTACATTTTTTAGTTAATTTAAATTTTGGTCCACGTGGAATTGCACCGTGTACACCATAGTTTATGT

AGTTTAATTAAGTAGATTCAT

>Ca_linc_0308

AGAAAATTGACATATGTCATAGTTAAAAGACTCAACAAATTGTTTTGAAAACAAAGATGGAAAGATACAC

TTCATCAATGAAAAAGGAGGATGACCAATTCGCCTATGCTGGAGCCATATTTGGGAGGCAGAAGAATGAG

ACTCTGATTGAGACTGAAGTGGAGAAAACAACACCTTATGGCCAATTTGTGCATCAGAAAAATAGTGTAG

CCATTTTTGTTCCTTAACAATTCCAATCATCTTCTCTGTTGCAAAATCTTGAAAGGTACAATGAGAATGA

AAAATGATTGTTGCACAATTATTATCCTCTATAAACTTACGAATAAAGATAAGGTTATTCGAAAGAATGA

GAACATGAAGCACATCTTTCAATTTAAAAGATTGAAGGTCAATGTCTCCACAACCATCAACGCGAGTATG

AGAATCATATGCAACATTAATATAAGTAATGCCAGAGGAGAAGTACACTTCAAAATCGATTGAGAATCAA

ATGTCATTTGATGATTGGCACCAGAATCAAGAATCCAACTACTCCTATAGGCTCGATTTTTCGTTGTGAT

TCCGATGGGATGGGTGGTGAGGCTCAATGCACGATGGAAAGATAAA

>Ca_linc_0309

TAGAATAGAAATATTTTGTAAGGTGTTGATTGATTTAATTTTTTTAAATGTGTGGTATTTGATATTATTG

TGATATTGGGTGTCGAATGAATTTTATGTATATGTTTTATAATATGAGTTTGTGTGATGTGATAATAAAA

GATGCAATGCATAGTGATAATTTTATGTGATAATGATGATGTATGATTAATAATTGTGATGTTATAAATT

TGTGAGAAGTTTATGTGATGAAGTATGATTAATGATAGTGATGGTATAGCTTTGTGATGAGTTATGTGAT

GATGATGATGTGTGATTAATTATAGTCATATTATAAATTTGTGATCAAAATATTGAAGTAACCTCATAGT

TGATGAAATAATGGTGATTACAATTTGTGTTTGAGACGAATGTCTTAATGAAGTATCATATGCCAACGAG

GGGAGAAAATAATATTGAGAATTTGTGAAGTGGATATTATTTTGTCATCATATAGGTAGGGTCTGACAAG

TCTAGTGATTATGGGGAAGTACAATTGAATGGGCTTTGATCGTGGCAGTTTGATGTTGAGCCTTAAGGCA

AGATTGTTAGATCTTAGAGGTATTCGGG

>Ca_linc_0310

CATTTTTTACTGGCATCTTCAACAACTTGACATTAACAATGCTTTCTTACATTGAGACCTACATGATGTA

CATTAGATTACCATATGGAATTTGTAAGAATAGAACAACCCAAGTCTACAAGATAATTAAATCTTTGTAT

GGATAATGGCATGAGAAGTTGTCTCTACATTGTTGAAATATGGTTTTCAACAAGCAAAATCATATCATTC

CCTCTTCATTAAACATTATTCATAAACTTTACAACTTTTTATGTGAATGGTGTAATCCTATTTGAGAACA

GTTTGGATGAATTTCAACTGATCAAGGATAGATTGC

>Ca_linc_0311

GTGTACCATAACATTAATGGTGTCCAAGAAGATAAAATGCTGATTATAATGCAGCCACTCTAACTAGTAT

CCAATGAAATACACAACATAGCTCCATGATTGAAGTTTTCATAAGCTTTAGTAGTTCCAATTGTAAGTTC

CAAATCAACAACTCCCCAAATTTGGGGTTCATTTGACAAGTACTTATTATTATTACTAGACAAGTACTTA

TTTTGACAATTACTCAGCCGTCATTATTAGTTGACTTATAATAAGTCAGTTTTATATTTGTTATAGTTAA

TTGGGTTCGTTTAGAGATGGCAAGAGTCATTCACCCTATATATAATCATATTTGTAATTTTT

>Ca_linc_0312

AAAAGTTTTTCATTCATTTGAAATATAAGAATACTTTCACCATTCCTCATACTCAAGCATTCAAGATCAT

ATCATATGTTGAAAATTATTTCTTGATCAAATTAGGTGTGCAGTCGAGTTTTGAGTGTGAAACAACAACA

ATGAGATATTGGTTGTTGGGGGTGTCTATTGTCGATATGAATCATCAAGTAGACATCGGTGGATTGTGTG

TTGAAGAAATGAAAAAGATGATCATTTAGGGGTCTCTTAGAAAGATATTTCAAGGTGTCTATCTTGCAAA

GAGTGAAGTGGATCTTCATCTTGAAGATATGTGTGTTTATAATTTAGGAGTTGTTCCAAAATTATGTATA

GAATAGTGTTTAGAAAGAAGAGTTTGCATGCAAGATTTAATAACATCTACCGAAGGATCTTGTAAACACT

TGTAATCAATAATCAAATGAAGAAAACTTGCATTCGATTAAATGTAAGTTTTTACATTGTTCTTTGATTT

TTTATTTATTTGAAGGTTGGTGA

>Ca_linc_0313

TGACACTTCTCGACAAAAATGCTCCTTGATAGATGAAAGAGGAAGACAAAGAAAACTCGTCGTCGGTGCT

GAAGTAGAAAATTTTTGCGCCAGACCAACTCGTCGTCGGCGATGAAGGCTGAAAGTTGTGTTCATTCACG

TTTTCACTGAAGGATGAAGGTTAAAGGTTGAAAGATGAAAGCATTGAAGGGGTAAGACGCGTGTGTTTAA

TTTGTGACTGAGTTTGATGAAGAACGAAGGA

>Ca_linc_0314

ATTTTATTCAAACACAAATACAGTTTATACATCAAATTTTGTCAAAGATAAGTGAAACATTTACGATAGT

ACTTATGCACGAGACTTTCACTTAAGCAGAACATTATGAATTGTTTCTATTTACTCTCATTACACATAAG

ATTTTCTTTCAAAGTGAACAAATTTTGATTTTTATGCCCACAAAGATACATATACTCAACTAATTGAACC

TCACTTTGTGGGCTGAAGAGAAATGCTTTGATGCGGCAAAAATAGAAGCTTCAGGTCCACTAGTTGCAGC

ATCTTTGCTACGACATTTCCTAGTAGTTTTCACATCACCACAAAATAACAATGCGAAGAATCTTGGTATT

GATGAAAACCAGCTTTTGTTGTTGCCCTTATCACCCATTTTGCTCACTAATTATTATAACGTGTGCTCAA

TTTTATCAATT

>Ca_linc_0315

AACAAATTTTAACTTCTATAAAGTCTAAAGCTATTGACTCGATTACATTATCCTCTCGCAACATTTCTCT

CGCATCTTGAAATCGTTCCGTAAATGAGAAATTGTTCCTAAAATTAATGTTTACTCCCGAAATCGTTCTC

TGAAATCACATTTAAAATCGCATCGCGCACGTTCTTCTCTCCCTTTCAGTTTGATTGCTCTCGTTTTCAC

AGCTACTAGATTTTTTTTTTAGATTCAAAGTGGCACAACATCAATAAGATTAGAAGCTGCTTCTTGTTCT

TTTGAAGATTATATATATTAATGTGTTATTGCTAAGATTACTTTGATCTTGAAGTGGCTTATTGCTTGAA

TACCCCTTGTATAATTGTGCTTGTTAGACATTTGTTAGTGGATTTTTTTTTTATGCAATTGGAGA

>Ca_linc_0316

CTTAACTAATAAGCATTTTAGGTCAGATTGAGATTACAAAATGACATGAAAATAAAATGGAATAAAAAGA

AGTTCATTACAACCAATTTACAAGAAACAATGATGAATGAAAGGAAAACAATCCAATTAAATATCAGTTG

GAACCACGACTGGTTCATCTTAAAATTGTAACTTCTTTCCTCGACTTCCAAGTTTCAAGAACTAGCTCTA

ATGACTCCAAAAGGTGGAGGTTGGTGATGAAAATGATGGAACATAGTAGGAGATAATGAATGATGAAAGT

TACGACTAGAGAATGATGGTGGCTCCTAAAATTAGGGCAAAAAT

>Ca_linc_0317

CAATGATATTCAAGAAATGCAATAAAAAGAATGGTGGATTTGATAAGAAACTCACATGCAACCAGCTTGG

ATCTTACGGTAGAATTTCACCATTTGTAGGAAAAATTATGCTCTTGTCGCAGAAAAGATGATTGTGAGAA

GAAGAAAGGGATATATCAATTTTTCCTTTGATTTATTGACAGAAATCAAGAGCACAATTTTCAACCAACG

GTTGAAAATCTCCACAAAAAATGTGACCTAGAGGTCATGAGTTGCATAAGGCATTCAAAATTCTCCATAG

GAG

>Ca_linc_0318

GTGAAAGGATTTTTGCAATTTACTCAAGATTTGACAAAACAACCTTGAGTTACAAAGTGATGGATTTGAG

ACTATTTAGAATCTTGATATTTACTCAACTCTTGTTTGAGAAATATATTCTGTAAATAGACCTCAGTTGT

AAATGATTACAACACAAAGATTAAAACAAAACAGCAAACTATAAGAAAGATTTTGAGACACTTGAGTATG

ATCGAAAATATTGATCTTTTGCTTAGTGCTGATTGTTGTGTTTTGTTCTTCAACTTGCAGCTGCATTTAT

AGACTCCAAGAAGGCTTATGACAGCTCATGTGACTGTTGGAAAGGGACCAGCTGTCATAAAAGAGTTGTT

GGATAAATTCTGCCAAAAGCAAGATTGATGAGCTGCATCAGAAGATCGATCCAAGATTGATCTTCTGGTT

TGTGATGAACTAGCCTTGTACTTCTGTGATTTGTGTCAGTATGTCAGCCTTGAGTACATGCTTTTCTGTT

ATGTGTGCAGGCTTTAGAATAGTTCAAATCAGTAGTGTACTTTGCTACTTGTAGTCTTGTACTTGCATTT

GCTTTTCAAGAAGAATGATCTTTGTAATATGCCTTTATTGAAGCATGTATTTGATTCTTCAAATTCTGGA

ACAAATGTGACTTGGATTGATCTTTTGTTGATTCTATATGAGAGTATAGGATGAATATTGTTTCCAGGAT

TGATCTTTGTTTTCCAGAACTGCTTCAAGATCAATCTGCGTTTTCCAGAACTGCTTCAAGATCAATCTGC

GTTTTTCCAGAACTGCTTCAACATCAATCTGCGTTTTCCAGAACTGCTTTCTTTGATCTGATTTGTGATG

TTTGATTCCTATCTTGTTTTAACACACTCAAATGCACATATTAAATATCAAAACACTTAGAATCATAATT

AGAATTCTTAATTAACTTTTTGTTTGTTTTCATCAAAACATTAAATTGAGATTTTGTCTCAACAAAATTG

ACATTATTCACGTGTGCAAAGACGAATCTTCACTTTATAAAAATTTGATTATGTTTTCAATCATACTCAT

GCATGTTCATCATAGTTGAGGACATTCAGACACGAGGACGTTAAGAAGAGTATGTTTTGTGTAACACCCC

ATTTTTAACGGTATTTTTTTT

>Ca_linc_0319

TACAAATAGAAAATACACCGGGGCTATGAGACCTATCAAACATCGCTCATACCCCTGGGATATTCTAGGC

AGACACTTGCATGAAAGTACTGCTCGAAGAATGTTATCCCCATGGTCACGTCAAAAAATGGAACATGGAC

CCACCAAACAAAAGTCAACTCTCAATATACGTTATAGATACAATCATCCAAAAACAAAATAAGTACAACC

ACCAAAAGAAAGACATTAAGTCTCTATACAACAAAAATTAATCTGATCATTCACTAGCAACTACAATAAC

AAGGGTGACCTCCACACATTCCGCAAGTTACTTCTGACACCGTATCTCTCCTCGAAGTAACTTACTCCTC

CTTATCAGCCATAGAAGGATCAGGTCCTCAGAAGCTAGGTCCATTCACGAGATATACTACCCCAACTCTC

CTACTTGTTCTCATTACCCTCCCCGTAAAAGTAGCCCTGCTTGAGATGGGGGTCGTATACCCAAACATTC

GACGAATGTCGGTTAGGGAGGTCTAACGACGTTTCCATCGTCAAGGTAAGTCGATGGAACGAAGTCGAAT

ATCATATGAGGGCAAAGCCCAATTTCCACAATAATGTAAAGTGTCACCA

>Ca_linc_0320

GTCTAATCAATTTTTTTTAACATTGATATGCATTCACAAAATACCAGTACATGAATACATCAATTGTTAT

GTCATCTTTTTAAGAACAATCAAGTCTTTCATGTATTCTACTAGTTTCAATCTTCTTGAAGATGACGTGA

GTCCGCTTGATCTCCCACAATCTTCTTGAGGATGACGTGAATTCGCTTGATCTTCCACATGTTCATCAAT

TTTCATAAGCAATAAGGATATTCACTCTTCATGGTTTACCAAAACATAGTAGTCTTGTAGAAACCACAAA

CCTATCAGGTATTCACATACCATTGTGCTTCTTTTTCAATAATGTTTTTGACACATTTTCTATACTCTGA

ACTGAGTTTGTGAAATTAAG

>Ca_linc_0321

GTAAAATAGAACTGACTAGAACTAACTCAAAATACAAAGGTGGTCTCGTGCGAATTTGTGTCACTAAAAG

GAATCAAATTGGCCCAAAGTAGTGTCACCTATGCCATTTAATGAAGAAAAATTTAAAGATACAATGAAAA

GTGTATTTAATCATCTGGAAGCTAGTGCACATATTACTAGAAGAAAAACCGATTAATAAACATTGATGGT

TACAAA

>Ca_linc_0322

TCCAAAATAGTTCCCTTGAATCATTCTCCACTCTTGCCAAACCCAAACTCTTGATTGTAGCACCGCCTCT

GACCACTACCAGAGATGAGTGTGAAAAAACACATGAATTAAAACAAAATTAAGCATTGTAAAAGTGTGGT

GGAGATTGATCGTGCAATCGGTTTCATCGACAATGAAATATGGATCAAGCTCATTGAGTTCTTGATCGAA

TTTGAAACTGACCAGATTGATCCACAAAATGGTGGTCACTTCATTCTTATTTGAGAAAACAGAGCCTTCG

ACGAAGTAAAGATTGACAGAGTCGAAGAAAGAATGGAGATCAATTGGTGATTAAAATCAAAATCAAAAGT

GTTTAGAATTTTCATCATTGACGTTGATAATGATATAACAATATACACATGACCACATTTGTTGCGTATT

TCATGAGAGATCACAACTATCAGACACGATTTTCATGTTCTTCTTGTTCTTTATTGGAGCTTCACAGTTC

ACAAATCATGTTGGTCTTGATAGGGATGATAGAAGTGTCACACTTATTGCAAACAAGAAATTCATATTGC

AGCCTAACCATCAATGAATTTCCAAATCTTGTTTTCTTTGTTACTCCATTGATTCAGATTCAGATTCAAA

TTCAAATTTAGAGACTGAAAGAAAGCAAACAATATCTCAAATTTGGATGAATACTCCTGTAATTTATGGT

TCGATCATATTTATTGGAAAAGTATGATTCTTAACTGACTACCGAATAAATTCATAACTG

>Ca_linc_0323

CGCATAATATTGTTTGTTGCAAGAACTTGGAGATACTTCGGGTAGGTTGAGCTGCGATCGACGAAAAGGA

TTCCGCTTGAATTACTTGTGTTATATTTTTCGAAGTCCACAATCCTATATACTAGTTGTCAGTTAGATTT

TTTAACTTCTTCTTACTTTGCTGATTTCTCAGTGAGAGTTAAAAGAAGAGATAGGCATTTTAAATAAGAA

TGACATGTGTATCTTTTATATGAAGTACATGACTTTTTAAATAAGGAACCATTTGATAAGGTATCCTTTG

AGTAAGTGAGATCATTGCAAGATTTTCAATGAATT

>Ca_linc_0324

CACACATCTCCTTCATAACATCATCATTAAGATTATATTGATACTCAGTTTAAAACTAACTAACGGACAA

AGCCAAATAGATAGCAGGACTCAGTAACAGATGTCCTGTCCGAGGACTGCTTCAATATAATCTTAATCTG

AAAGATTATTGCAAATCCGATTAAGAATTTTGGTGCTTAGTTTAATTTTTCAGAAAATATACCAAATACT

GGCTAGATCAATGCAAATCAGACTAACAATTACACTTGCAAAATACATTATGCCAAAATCAAACTAACTT

GTAATTTGTTACCAATGTACAGTTATTCAAATTAGTTCTATTCTATTTATAGAGAGAGGTTTCAAATGTG

ACTAACTAGTTGAAATTCTAGCAGTTCATTACACAACTTAAAGAGGGGTAAATTACAATTGCTAATAGCA

AAACCAGCAAAGCAATCAGAGAACTTTGAAGACCATTAATATTAGGAATCAACATAAGATTCAGATAATA

AAATTTCATCATTTTGCTCTGCTTTCTACAAAAACCAATGACACAAATATTACTTTTATTAGATTTCACT

TTATCTTACGGACAATAACTTAACAAAAACCAAATAAGCAGCATCAAGAAGAAATTTCTGAAGAAACAAA

GACAAAAAACATACAAAAAAATTTTAAACATCCATAACAGTGCTTACTGAACCAACATATTCTAAAAATC

GGTCAATAAACCTAATAAATTAATTCAAAACAAAACATGAAAACCTATCATTAACAAAAAGGGAGAAATA

AATAAAAGGCACATAATTAAAGAAACGATCTAATTAAAAAATAATAGCAAAGCAATCAGAGAACTTTGAA

GGCTTGTATGAAACAAGAGATCAACATCAGTTTCAGACAAAAAATAATTCTTCATCATTTTGCTTTGTTC

ATTGAAATGTAAAAACCATTTATCAGCAAAGAATATAGTACTATACGGCTAACTGACAGAAATAATTTAG

CAATTTAACATTAATGCAAAATGGGCGATGAAAAACTAGTTCTTCACCCAAAAGCATGGATTTAAATTGC

TGAGTAAATTTTGCACCTCTAAAACCTTCTCGTAATTTCCTTGCAACAAAACTTAAAGATGCAAGGGGCT

ACGTAGTGCATAACGTATGCAATGATTTTAGGAATCAAATCTAAGAAATGAAACAAGAAACAAAAACCAG

GAATACACACAAACAGTTGAGAGGCGGCTACATACTTGTGGGTTTCATTTGCAGCGCTAGCGTCTTAAAT

AAGAAAAATT

>Ca_linc_0325

GTTTTTTCTTTATTTCTCACTGTGAATTGCCTCTTCTTTCAGTTGCAAGATCTGAGATCTACGGATCGAT

TACTTGTCGACTTTGTTCTGGGGGCATAAATATAATTTCAAACCAAAATCTTTTGGGAACTCGCATTTTT

CCAGATTTACGTTTTTTGTTCTTCTGGATCCCATGTTTTGGTTACATTTACAATTAGTTTACCATTCCTG

TTTGTCTGATATATATTTATACCTATAAATAAGAA

>Ca_linc_0326

GAAGAAGTGGAATCTTTGTGTTTAGCCCAATCAACATCATTGTAAGAAGTGAGAGTCGATGATGCTGGCT

TTTGAAGAAGAAGACCATGAAATATAGCTCTTTTAACATGGTTGACTTTTGCCATAAAGAATAGATAAAA

ATGGGAGTATCATTCCAAAGAAAACCACATCATTGATAGACATGATGCTGAGAGGAGAAACCTAGAAAGA

AACAAGACATACAACAATGTGAGAGCATATGGCTAAAGCCGAAGATAAACAAAGACACTCGAAGATTGAG

ATAATTGGCTTTAGAGCCAAAAAGGATTTCTTGAGGACACTTGTGATTGTGTTGTGTGGAACAAAACTGT

GAAATAAAATAGTAAAAATAATTTCTACTCGTTCAACAATGCCAACGAGTTCAAAAATG

>Ca_linc_0327

CCTTTCTGAACTTTTTTTGTTTGTTTTCCGAAAGCAGGATTTGGCTCAGGATTGCCCATTGTTAATTCCA

GGGTTTCTCTGAATTTGAAAGTTTTCACTTAGTAAGTTTCCATACCAAGGCTCAATCTAATTAAGTCCGT

AGCGTCTACCAATTTCGCCATATCCCCCGTTTTTTCTTTGAGATTGAGATCTCATTAGGATGTTTTTTCA

TTTCTATTATGAGAATTATATGCAGGAATTATTAGATACCTAATCCCATATTGGGAAAATTTTGCTTCTA

TAA

>Ca_linc_0328

ATGTGGTTTAGTTTTTAAAAAGACCAACCATGCATAGCCATATATAGGTATGGTTTTGATCATACTCAAT

TGAATGTGAGAGTAAGTTCAACTTTAGTGCAAAATATGTAAAGGAAAAATAATACCTCAAGACCTCATCC

CTTATCTCAAATAATACTTTCCACTCATTATTCTTTTAAATTAAGGATAGGAGAAATTTTTATTAATCAA

AGTGATACTAATAATTATTTTACAATTATCCCAAGAAGATTTGTCCATTTTACAATTTTACTCTCTCAAT

TGGGTCCCAAATTGATGATGAGAGTAATCGTGTCAAATGAGTAGGACTATTAATCCATTAATTAATTGAA

ACGACAAATCAGAATTACCCAATTTTGGGGGTTAAATTTCAGATATGTTTAAACCACCTGATGATCCTAC

ATAATCAAGTGTCTAATGTAACTTATGAAATAAATCAAAAGGAACAACTACATCATTTAAAGTTTCCACA

ATATGAATCATGATGAAGTGGTGATGGTGAATAAAAATTATCTGGGTTTATATTTTTAAAACTTGTATCT

TATTTGATTATTTTTAGTATTTAAAATGTG

>Ca_linc_0329

CGACAAACAATTTTAACCTAAAATTGGGCCCCCCAATATAGAGATCCAACATAAGAGGGTTATATTGGAT

ATCTCATCCATTAAACTCATTGAGTCTCACAACTTGAAAAATATACAAGTGAATTTTTATCGTGAGATTT

CCAACACACCCTCCTCATGTTAAGATTCATCATCATAGAATCCAAATCAATCAATCACATTTGTGCATTC

TTAGTGGTTTTTCCAAATATCTTCTACCATGGTGTTTAAACTTTGTAACTTCCTCACAACCTTAGTATTA

ATACTAAATTAGTTTTTTTTCTAAGTACTGTGTTTTACATCACAAGACCATGTTAAATTTAGTAGGTCAA

AACAAATCTAATCTAATTTAATATATAAAAATATATTGGTAGGAATTCAAAAACCTAACATGTCAAAAAT

GCAAATGTAGTAGTGTTGAATAAAACCATTCAGAAAAACATGGGCTAGGACGCTCCCCTTTAAAAAGGGT

ACATTGATATTCCTCTTCCAGATAATCATCAGATTACAATAACAAAA

>Ca_linc_0330

CTAGAATTGAAGAATTAATAAAGACCAACAAAATCTCATAGCGTCTTTTTTATCCTCTTTATCGAGAGTG

ACTCAACCAATATTCCAGCATTATTATGATCAATGCAAGAAAATTCAACTCATCTAAAATCTGTCTAGCA

ACATCTATCGACTTGAAATGACTAGGAAATAATAAAAAAAAACATCCCCTTGAATACCCTCCACACCAAC

CATTTCTTCACCATTTACATCATTGCTTAGCACTACATCAATTCTTCAGCTTCTGGCCTACTACCATACC

ATGAAAATAAATTTGGAATTGGCATTGGCAATGTCTTCCTTCAACCAACCATACTATGTGTGATCGTTGC

CACTGAATTCGTACCTATGAATAGAGCTGTCAATTTGACAAGCTCCTTAATAATTG

>Ca_linc_0331

CAACAAACTAACAACTTCATAATTGTGATTACAGATCTCAATACAGTTTGTTGACAATCTAAGAGAAATG

ATAATGTGCTCAAGAACAATCCATGAATATCCAGATTTGTGAAGAGCAACATTCATAAAGATTGAAGACC

CTTTTGTTTTACGTCTTTTATTCTTTCTGTAATAAATCTTTGAACCAAAACGAACGTTGAAAATCCAGCT

AGAAACTGGTGGCTACTTTCTTGGGTGAGAGGTCTCAACAAGAAAGAGTCGTACTTTGATTTTGTGTTAG

GCTGACGATTGTCTACAAGGATCAAAGGGTTGATAGAAAGCCAGCTAGAAACTGGTGGCTACTTTCTTGG

GTGAAGATTGTTCAACAAGGAAGAGTCGCACTGTGATTCTGTGATAGGTTGACGAGTATCTACAAGGATC

AGAGGGTATTCAATAGGAAAGAGATCATTAAGATAGATAGGTTTCAGGGAGGAAACTGGACAATCTGTAA

TTGATTCTTTCTATTGAAGGAGAAGAAAATCTGAAATCCGATTGGATTTTCAGGACTGGACGTAGGTTGT

TGTTGACAACCGAACTAGGATAAATCTCTGTGTTTTCTTCTCTAACTCTCTCTCTTTAATTTTCTATATT

GTTAACTTTGTATGCCTCAAAATTTAAATTCCGCTGTGCACTTGATACAGATTAATTTGGTAACGAAAAC

TGATATATTTTCTGTTATTTCGAGGTCAAGATACCAACACATCTTGCAAAGATGGCTCTTTTGGGGAATG

ATGTGGTTTTGTGGCCGATTGATTTTCCGCATAGTCTTTGCAATATCATTCAAGAAGATGCCAAAGGAAC

GAAATATCTAAGACGGTGATACTTACTGATATCTAAGTCTGAAAGTTTTGTTTGTGGTTTCCATTTTGAT

GGGGTTGGTGGTGGAAAC

>Ca_linc_0332

GTGAATTATAATTTTATTATTTGCATTTTGTCCCTTGCTCGCTATGTAGGCCATAATTTGAGCTCTGGAT

ATCGAATTGGCGCATACGAGCAGGCGTTGGAAAGTTAAAAATCAGAGCTACAACTTTTATGTTGGAATAA

AAGCCCAATGTTGAACTTTATTGGACCTAAATTGCAGATTTCGTAATCTAGACTTTTGTAATA

>Ca_linc_0333

CGAAGGCAAAAGTCTCTCAAAAATATTAAGTGGTAATCACATATTTTGAAATTGCAAGTCATCTTCAGGT

ATATCACCATATGTCGACGGAGCCATAACTAGTTGAGGCTGATTTAAATATGGTTTTATCAAGTCAACTG

TTGGCATAGTTGGTCGGTATGGAATTTGATCCACTAGAGGTTGAGGAAGCTACTGTGAAAGAGACGACAC

ATATTAATTATTAAAAGATTGTTGTTGTTGGAACATTGGTTGTTGTTGTTGCTGCGAGAAGGCAGAGTTT

AGAGGCTGTTGTTTTTGCTACTGGAAGTTTAGAGGTTGTTGTTGCTGGGAATGTTAGGAGTATTATTGTC

GTTGTTGGAACATTGATGATTGTTGTTGCAAGTTCTAAGACCATTGATATTATTGTTGATGTTGAGGCAG

GAACTATTGGTGAAAAGCAGCAGGAAATTATCGAGATAAAAATTGTCGTTATTGTTGTTGAAACTTTTGT

TGTTAGATCACATTTAAATTTTCAAATGTTGATTGTATCGTCCTCTGTGTTTTTTCTTCTTACTTAATCG

TCCATTGTTCCTTTAATTGCTCAACATTGTCTGCAATTGATCGATTCATACTAAGATAAGAAGAAGATTA

GGTTTAAATATATTATGATGGGTCGTTTACAAATGTTGATGGTAAACTATTGGTCCCCAACACACATTCT

TTGAACTTGCCACTACTAATTATGTTCATAAAACTTGCAGTCTCATATTGTTTACGCTCCAATTGACTAG

CTCACTGAGCAGGAGGAAGAGTTGCTTGCTGATAATCTCATTCTTTCATGATTTGTTGAAGTTTTCCTTG

AATAAATAAATTAATT

>Ca_linc_0334

TTAGCGTTGGTCCCATAAAGAGCAGGATATGTGGTTTGAAGTTGAAGTTGAGGAAATGTTTCCACCACTA

TACAAGAGAGGCCTTGAAAGACCTAAGAAATTGAAGAGGAGAAAACTTCATAAGGATCCTAAAAGAAGAG

GTGTATCATATAAGTGTACAAAATGAGATAAAATATGACATAATGTAAGGAGTTGTAAGAACACAAAAAT

GAATCTGAATACATTGTTGCGTAAGATATTCAACTTAATGTTATGTGCATTTAATCTTCAAAAAAAAAAA

CAACCAAATGTTGTAGTCGAGCTTGCAACAACACAACCAACAGTTGTGCCTATAGATGGATCTTCTTCAA

TACCAGTAAAAGACCTTACCTTGATAATTAACTGTATTTAACCTTCCN

>Ca_linc_0335

AAGATAGACTCTTATACCAATTTAGAATTTTGACTTTAAGCTTAACTGAACTCCAACAGCTAGATTAACA

TGTGGTGGAAACATAGAGACACACAAAAAGCCATGTGGTGGAAACATGTTCATATGATACAATGTGATCT

AGTTGTGAATCCAAATAATCAGATGGGATGAATGTGAGAGAGATGCACCAGCAGGGAGTATGCTTCCTTT

GCAGGTGGGTACCGAGTAAGGAGGACAGCTGGACGTGGGAGGAGGGTGCTGTTGTTTAAAGTTGCTTCAC

AGGGAGTGTACCAAACATGTAGTAACAACCACATTCAAATGGAGGTAGCTGGGTTGAATGGCAGTGGTGG

CAGGGGTACATGTTGCTGGGAGAGGTTTTTGTTTTCTTTGATACAGCTGGGATAAGTTTTGGGAGAAAAG

ATAATGGGGT

>Ca_linc_0336

AGCCAATCGACCCCAACAACTCCATGTTTGTAAGTTACATTGGAGCTGTTGTTCGTCAAAATATCCCTAT

TACAATCGATGACTGGAGAAATAAGGCGTTGAAGGATGCCAAAGATATATTGTGGAATGATATACAGATA

AATTTTTTGATCCACTCTTTTGTAATTTATTATTTTTTCCATTGAAATACTTTACTCAAACTATATCTTT

ATTTCGTTTGCAGACTGCTTTTGTTCTTGATGAGGTGAGAAAGAATTACGTCTTGAGAGTTGCTGGAAAA

ATACTCAGGGTTTTAAATCAATCAACACCTTATAAATATTTCTATTCCACATTTTATTCTCACATTTTCC

ATATTTGCATATTAATTAATGTATCCCTCAAAAGGTTATTGTTGTACGTAGCGAGTCCTGGATGAATTCT

TGGGAAATACAGTTTTCCCGTTCATCTCACAATATATTATACTCATGAATTCAAACGCTCTTTCAATCCT

TACCTTATTTAGATGTTTTCTCAAACACAAAAATCAATGGAAGTTTTCAACTGCAACAGTAGACCGACAA

GGTACAACGAGCGAATCCGAATAAACAGCGAGTTCACAAAATCGTCGAGACGCGATTGAAAAATTA

>Ca_linc_0337

GGATTACACTCTTTGGATGTCTACTAAGAATATACTATATTAAAATCTTACTAGAAAAAAAAAACCAATA

AAAAACAATTTTAGTGAAGGAAGAATAGTACATTTTCCTTTTCTATGGGATCTGACTCATTATAAACTTT

ATTAGCAACACATGTATAACATAGCCTTATTAAAACAACAACCCGTGGATCACCAAGAAGTCCATCTCAA

ACACTCACATTTCAACAGTGAAACAACTTTCAAACCAACTTACAATAAATCTTCATCTTGATTGGTGGGT

CCAACAAGCACTACCATTTGTGGGTCAATCGACAACACCAATTCAATATTTCCATCATGCCAACATAACA

ATACGTATATTATTTCACCTTCCATTGTGATATTTCACTTTCCATTGTAATAAGTACATTAGGAAAATTA

CCGCAACAATTTATAGATCACAATTTCCTACTAATACCGAAAAGGTGGGATTGCCACGATAGATAAGTTC

ATAAATTCTGCAATCTATTGTTGAATAATGTTGTGGGAACTTCTAGAATCAATGAGTATAGTAACTTTTA

ATTTATGAATATGGCCCTGAAGTCGGAAGGTGTGTGGT

>Ca_linc_0338

TCATCGTCGCATCTCAAATCACCACCGTCACACCTCGCCACCGCATCTCAACCATAGTCATCATAACCTC

GTCGCCGTCGTAAGTGTTGCGTGCCACTCTATTTTATATCAGTCTGGTGTCTCGCAATTTTTCATTATTC

TCGTTTCATTATTCATTTCTGTGAACTACAGTGAGTTAGGATTTTGATTTTCTTCTGGTTTAGCCACCAC

CGTCACACCTCACGGCCATCGAATCTTAACCTCTTTACGTTGCATCTCAACTTCGTTGTCATCTTAACCT

CGTCGCCTTCGTA

>Ca_linc_0339

TTATATTATGCTTTTGTGGGACATAATATAAACTTTTCAACCCCACAATTGAAAACCAATTGCCACAAGC

CACACATTGTAAATAACTTATAACTAGCAAACAAATCAATAAACAAAAAGAGTCTTATTATTTATATAGG

ACACTTTCCCTTACCCTTTAATCTCATCTCTCTGTATAAGAAATTCTAAGTCTTCAAAACAAGAAGAAGG

AGAAGAAAAAGTTCTAAGGTAGAAAGATGATAATGGTGGTGATTATGACGGAGATGTTCGGCGAGTACAC

CGCCGTGTTGACAAGGGTGACAGAACGATTTCTTTCACGAAGTGGCATGAGTTTTAATGGTCTAAGAAGT

AGGAATCTTCGTTTTGCTTCCTCAAATTCTTCTTCTTTTTCTTTGGATTCATCTTCTTTTCTTGTTTACT

TTTAATCAATTTTGTTAGGAAAAGAAACAAGTTTTTTTTTTCTTTTCCTTTATGTTTGAGGAAATATATA

TTTGTTTATTGCATAATAAATTGATGAATGTTGAGATGAAGAGTATTTCTTTGGAGGATTAGGCGTTGTT

AGACTCTTGCATTTGATGTGTAATTATGACACATATTTTGTTATAGAAAATTATACTCCTTATAGTTTTG

ATTATTTTTCATTCATTTAATTTTCAAACTTTCCCTTACCTTGAATTATTCTTCAGCTGCAGAGGCTTGA

GGTATGCCTTTATTTAAAATGTGATATTGAGTATTATTGGAAAAATAAAGCATGAATCATG

>Ca_linc_0340

GCCGTTTACATCCTCTCTGCTCTTCTTGTTTAAACCTAATTGAAATTAGGGTTTTTCAGTTTTCTTCGAT

TTTGTTCTTAATTTCCGTTTAATTTTTCAATTCTTAAAGTTTGTAAACAGGCTTCACTATCTGGTCGATG

AGGATACTTCAATTCCCACTTGACTCGTTCCCTTGAAGCAATAGTAATATGTGTTGCTCTTTCAATTTTA

CTTCCACAACCCCTTTTTATGGTTTTGGGAGCACGCGAGAACAACTAAATTTCTAATTTCTCTTTGAAGT

TGACTTTTTTATTGATTGATTTTCTCTGTTTTTGATTCAATCGAGTAAAAAAAGGGAATTGCTAGTTTTA

ACTGTGATGAGTCTACAGACCTGTAATGATTTGCGGATATGATCGCATCATTATTGAACATCTAAGGGAC

TGAGTTTTGTTGTGATTCTCTCACTTTTTTTAAAATTTTGTTTAATTTATTTTGGCATGTTTTTGATATA

ATTTGGCTTCAAAATTTTGTGTTATTTCCGCTTCCACTTTTTAGTGTGGTTTGCAATTTTTAATTGAAGC

CTAGAATTGTGTTCCCCTCGCTATCCGATGCTCCAAATTGATGTTGCATTTGGACGTCGGGGAACAAAAT

TTCTCATCATTCAATGATATTTTGTTATCCTAATTTATTTATTATTTGCAGAATTCAGATCCATTTTTTT

GGTTAGAGTTTTAGTATTGTAGATAAGGATGAAGCAAAATATATAATCCAACACTTGATCTGATTTTTGT

GATGCTAAATATATCTATATGCTCTGAATCTACATCTTGTGCGTTTTTACTTGTTATTGTTTTCTTATAT

TGTAATCAGTTTTGTTGTTCTATTACTCTCCTTTACTTCTCACTGCATCATGTGATGAGTTTGGAAATAA

ATCTTAGGACACCTTCCTACACAACTTATGTTGTGGATGAGATGAATTATCCTTACTGATGATGTGTTAA

ATGTTTTAATTTTTGTGTGCAGTTTAGTTCTCTACATTTAAATGTTGAATTTTTGTTGTTTGCTTTATGT

CTGGCCTTCATAATCCCAGTTAAGATTGAATATTTTATTCAGTTCTTATACTGATTCATGGGAGGCCAAA

ATAAATTGTTGGAAGTTTCGCTGATAAATGCCTGAATCTTACCTTTATATCAAGGTGTTTACTAGTAACT

TGCCACACATTTCACTATTGTTCGTAGGTTTTATTCGAACTATGAAAGGGACTCTTGTTGTTGCATCAAA

TCATTTAAAAGATGTTATTAAGACCATCCCATGAATACAATGCAAGCATTTTTTTCTAACGAAACTTCTA

ATGTCACTTTTAATCATTTCAGTCTTTCATGTATGGATGTTAATGTTTCAGTTACTGCTTACTTCTCGAT

ATGTAACTATTGCATATGGTAATTATTTAAAGCCAATGATGATTTATTGTTTTAGCAAGTGCCGTCTGAA

AGCTTAACCGTTTATACGGTGGTGCCTGATGCACAATTTTCACAATACAACACTGAGGCTTTTGGTGCTG

TTATCATCTTTCCCATGTGACCATGCCAACTTAATATAATGATTTGAACGACTGTTAAAAAAATCATAAT

TTTAAGAACGAAAATTTAAGACAATATATCCTAATTACTAAACTATATGAAAGAACTTATAATACTTATA

ATATTTTAGACTGGAATTGAATGATAAAAATAGATCTAAT

>Ca_linc_0341

ACTTGACTCGTTCCCTTGAAGCAATAGTAATATGTGTTGCTCTTTCAATTTTACTTCCACAATCCCTTTT

TTATGGTTTTGGAATCACGCGAGAACAACTAAATTTCAAATTTCTCTTTGAAGTTTATTTTTTTATTGAT

GAATTTTCTCTGTTTTTGATTCAATTGAGTAAAAATGGGGAATCGCTTGTTTTAACTGTGATGAGTCTAC

AGACCTGTAATGATTTGCGGATATGATCGCATCATTATTGAACATCTAAG

>Ca_linc_0342

GTATTATTCACGCTTCCACTTTTTAGTGTGGTTTGCAATGTTCAATTGAAGCTTAGAATTGTGTTCCCCT

CGCTATCCGATGCTCCAAATTGATATTGCATTTGGACGTCGGGGAACAAAATTTCTCAAAATTCAATGAT

TTTTCGTTATCATAATTTATTTATTATTTGTAGAATTCAGATCCATTTTTTTTGTTTAGTGTTTTAGGAT

TGTAGATAAGGATGAAGCAAAATATATAATCCAACACTTGATCTGATTTTGTGATGCTAAATATATCTAT

AAGCTCTGAATCTACATCTTGTGCGTTTTTAC

>Ca_linc_0343

AAAAAAAGGTCCAATTTTCATTAAAACCTAAAACACAATGTCCATTATTCTTTGGGATCCCGTTAAACGC

AATCTTCATTGGCTCTTTAAATCCAACTGCCTAATATTTCATTGATAAGAAAAGTTCCAATTTTCATTGC

CACTTGAATTGAAAAGTCCAATAATAATTTGCACCTCAACCAAATCCCCACGTTCTATTGGTAACGTAAA

ATCCCCAACTTCATTGGCACCATATAAGCCTACATCCATTAACATCCAAATATCTAATTTGTAATGGCAC

CAAAAACCCAATTTGCACTAATACATTGGCACCCAATCTGCAGTAATACTCATGATAGAAGAAGTTCTCC

TTTTGATGCTTACTCTTCTTCTAAACATGAGCTTGAACAAAGTCAAAAACTTCATCTTAAAGCAACCCTT

GACGGAAGAGAGGCTCGTAAAAGAGATCTTAAATTTGTCCAAATTAATGAATTGACCAGAAGCATTTTGA

TGGATAATAGTTCTCAAGTTGTTTGTTTCAGTCTCATTAGCTGTACAAAAATAAGACTATCATCGACAAA

AAATAAGTGAGAGATCGTAAAAGCCCTTCTGGCCATCTGTATGTTGTGAATGTCCTCATTAGCACAAGT

>Ca_linc_0344

TTCTTCTTAAACCCTAATTGAAATAAAGTTTTCTCAATTTTCTTCGATTTTGTTCTTAATTTCCGTTTAA

TCTTTCAATTCTTAAAGTTTGTAAACAGGCTTCACTATCTGGTCGATGAGGATACTTCAATTCCCACTTG

ACTCGTTCCCTTGAAGCAATAGTAATATGTGTTGCTCTTTCAATTTTACTTCCACAACCCCCTTTTTATG

GTTTTGGGAGCACGCGAGAACAACTAAATTTCTAATTTCTCTTTGAAGTTTATTTTTTTATTGATTGATT

TTCTCTGTTTTTGATTCAATCGAGTAAAAAAAGGGAATCGCTAGTTTTAACTGTGATGAGTCTACAGACC

TGTAATGATTTGCGGATATGATCGCATCATTATTGAACATCTAAGGGACTGAGTTTTGTTGTGATTCTCT

CACTTTTTTTAAAATTTTGTTTAATTTATTTTGGCATGTTTTTGATATAATTTGGCTTCAAATTTGTGTT

ATTTCCGCTTCCACTTTTTAGTGTGGTTTGCAATTTTCAATTGAAGCCTAGAATTGTGTTCCCCTCGCTA

TCCGATGCTCCAAATTGATGTTGCATTTGGACGTCGGGGAACAAAATTTTTCATCATTCAATGATCTTTT

GTTACCCTAATTTATTTATTATTTGTAGAATTCAGATCCATTTTTTTTTGTTTAGAGTTTTAGTATCGTA

GATAAGGATGAAGCAAAATATATAATCCAACACTTGATCTGATTTTGTGATGCTAAATATATCTATATGC

TCTGAATCTACATC

>Ca_linc_0345

TCGTCCTAATCTTCTTATGCACTGATTGTCATTATTATCTGGGTCTTGCTAAGCAATGGAATCAACACCT

TACTCAGGTTCATAAGGCAATTAAGTAATGTATTCAACAACTTGCTTAAGTTCATAAGGCAACTGAGTAA

TAGAGTCAGCACCTTGCTCAAATTCATAAGGTTGTGAACTTAGGGTATGCTCCACTTCCAAATCACAACC

AAAATGAGCTTTATGCATCTCAATAACAATAGATTCACTTGTATTAACATGACTGCAATCCCAATTCCTT

AATTTTGAATTGCTTGGACTCTAAGGAATATGAGTT

>Ca_linc_0346

TCAGTTAACTTTACATAGCAAAAGCATGCAGTCAAAAAAGTAAAAGAACCTTGCACTACGATTATCCATA

GATATATATACATATATATCCATCTTGTTTAACTACTGTCCTTTGGTATATAGTATATGCTCAACGATTA

TCATATACTATCATTTTGCTTCATGCATAATAACTGATATGATTCTGGCTTTGTTTAACCTATTTAGGGA

AACAAAAATACGCCAATACCATTGAGGGAGACAAATAACAACCAAATACTTTTGTTCACTTTAATCCTGT

GCTGTCATGTTCACGAGCTTATGGATATGTAGCACGTCTATGTAGAAATCTCCGCCTAATGAATTTAAGC

ATTCTCTTGACCAAAATAATTGAACTTAGAGCAGTGTGACAAAAATGATTTTGTTTTATAAACGACAAAA

CAAAGGTAAATCATGCTCTCAATTCTCTGGATCCGTGATCGTTTGGACTTCTAGCTTGCCCCCGTGATTG

TTCTATTCCTCTTTTGTGGTGGATGTAATGAGGGGTTGTTTTCGAAATTGGATTCCCTACAGTTGGGGAA

GTCCACGCATTCCCTACTATTGATTTTAATTTTAATTATATAATTTAAATTACTGAGTTTAAATTTGAAC

TTGCATGAATGAGTGATTACCGAAGCAGAAGACCAGATATGTTGTTTTCGTATTAGTATAATTTTTTTAA

TTAGTATATCAGTTTAATATTTTGATATTGTTACATCGTGCAATAACTTTTTAAATAATATATTGAAAGT

ATTACAAAATATAAGAATGATTTTTGTGGTTGAGAAATGTCGCAGCTGTGAAACAGGTACAGAAAATGAA

AAGTAGTCAAGTTGTCGTTGACTTGGTGTACATTCATTGGATGAAGGATAGCGATGAGATATGGATTAAA

GTGCATGGGATGGAAAAATTGGAAAGGCTAGATACATGGGCAGTTGTTGATGGAAGTAGGGCTCCTACCA

TGTCTTAGCCACTCAAAGAGACGAGTGGGTATGACATTTACAGCATCGGCGGCAATGCATACTAGGATAA

TAACCCGCCACGTCAGCTAGCTGGCCAAAAACAATTCCATGCCAACTCGGATTGACATGTATGTGTCCCC

ATGTTTGTTGGCTGTCAAATAAGGCAATAACACAACAAAGGTACTCATGTCTACATTGCACTTCATTACC

TGCTTTCTATCACCCTTCAACTTTTTAAGACAAAGGGAGACATATAAGGCATTATTTTTAATTATTTAGA

AGACAAGGCAGGGAAAAGAATGTAATAGACCTATTTTTGATCTCTT

>Ca_linc_0347

TAAGTTTAATTTATAGTTAATTTTGATTTAAAAGATTGTTATTTTGAACTTTGGAATATTGTAACACATA

GTTGACTTTTTAATTTATCTAAATTATGATTGTTATTTTGCATAATCATGTTCATATATTTGATATTGTT

CTTGAATGGTGCATGGATAATCAAGGGCATACTATTCCTCCTTTTGGTAAAGGGAGATGTTAGTTAGAAA

ATCTCAAAATGTTTGATCTCAAATTTTTGTAATTCTTTTGGTAGTTTTTGAGACTTAGGGTTAACTAGGG

TTTGTTTTTTAATGTTTTTTTCTTTCGGTCTTTTGAAGTCCATTTACATATAATTGGTCTAATGAATGTT

TTGCAATTATATATGAG

>Ca_linc_0348

CAGTGATATTGTTAGTTACAAATGATTAACCTGAAAGTACATTACACAAGTAAATCATATAAAACTAAAC

ATTAATAACAAATATAATATAAAATTCCAATGCTAACGACAATAAATGTAGAGATGGATCAGATTATTAG

GCTTGGTTTCTCAGGAGAGAAAAAATCGCACAGTATGTTCAGCTTCGATCATCTGGGGAAATAATCATCA

CATCCATCTCTACAATACAACACAAATAATGATAGTGAAACTATCTATTTCAATTTTCACCAACAAACCA

ATCATGAATCAACGTAAAAATCACATATTAGCTTGAAAAGTCAATGCAAAAAAAATTGAATTGAAAATTG

AGATTTCTGACAACGCGTTTTTTGAAAAATAAATAAAGAAATAACATGAAAATTTTGAATCATTATCATT

ATAAACCCTAAACCATAACATAATTTCTAATTGAAACAATACAATAGAGAGATAAGCGCAACCATGCTTC

GTATATGAGACAATGAGTACCGCGAT

>Ca_linc_0349

GAATAAATGTAGAGGAAATTGTCTTCCAAAGTTGCTTCTTTACGTAAATAGTTTAGCGACAAATGCATCT

AGTGAACAAAGGGTTCTAGTAAGTAGTAGATTAAATAAAAGGAAAGAAGAGATTAGAAGGAAACTCATAT

GAGATATTTTTATTTTGAATATAATTGGAGGTTGAACTACATCCAAAACACATAAGAGAATGCAGAATTG

CTCCGATAAAATGTGAAAGAAATAAATAAGAGAACTTAGCAAAATAAGCACAAAATGACTGGTTACTTCT

ATAAAAAATAAATATAAAACTTGTCAAGTACCAGAAGAAAAACCAGATAAACTTGCCTACCATGTGTACA

CACAATTTGCAAAATAATAACCGGCAACAACAAATGCTATTTACAGCAGTATACTATCCTAATTTGCATT

GTAACTAA

>Ca_linc_0350

GCTGGACTAGGTGCAGGTGAACCGGGAGTTTCAGCCTCAGGAGGCGAACTAGGTGTTTCAGCTTCAGGAG

GTGAACCAGGTGAGTATGCTACAGGAGGTGGACTAGGTGTTTCAGGTGTCGGAGGAGGTGAACTGGGTGT

TTCAGGAGTTGGAGGAGGTGTACTTGGTGTACCCGTGCCGGGCCATGTGGGCCAACCAGGCCATCCAGGC

CACCAACAATCAGTACTTGCAGTGCCATTTTCTTTAACACCATTCACATCATTTTGCT

>Ca_linc_0351

ATTTGACCCCAATAAGAGTTAGTAAAATAAAAACTAGAAAAAAGCATTTACCAACTTTGCACTATCTAAC

ATGAAAATAACGTCATTGATCGCCATTCTTACCCACCTTGTTGCTACTACAACTCACCATTCTCATCACT

AATCACCATGGTAGTAACGCAATGGAATTTTGATTTTTCACAACTTCTTCCAAAAGGCCCAATCCACCTT

TGAAGAACAAGAGCTAGGGACCTTAGCAGACTCCCACTCCCTAATTAAATGAATTCCTGAATCACTAAAA

TTAATAATAGGAAATTGGATGATCTGCTCTGCTTTCAAACCAAGAAAAGAGCTACAAATAAGAGGTATGT

TCCAGCTTCCATCCTCTAGAAACAGATCCTTAACTACCTCCACCTCACAGTTTTCTATTATTGAATTAAA

ATAAAATAAAATAAAATT

>Ca_linc_0352

CACACTTTATCTTCCATCTCTGAAGTTTGAGAGAGTATAAACCTGTTATGATTTTTCTGCATGCAGCCAC

AACAATGCCAAATATGGTCTTACAATCTCAAGGAGTGTGTTACCCACATCATTACTAGATAGATTCCAAT

CCAAGTATGCAAATAGACCCAGAATAAATAATTTACTTCAATATTATTCTTTAATAGTTCTAGAGCAAAG

TTCTTTAGTATCTT

>Ca_linc_0353

CTTCATTTTGTAAGTAGCTTACCATGTGCCATCTTCCAAAGGGTAGCTTTGATTCTAGTTGGCCCTTTAT

AGATCCACACATTGGTAAATAAGGGAACATTTATAGCTTCATCTTGGGAATTAGAAATCAGAACTTCATA

TGATAATATGATGGAGAGTTGCTACCTCAAGAGCATATAGATGATCTATATTTTTTTATAAATTATGATT

AAGTTTATTTATTTTAATTTATTAAAATATTGATCATCTTCGTACTCTTGAGGTCCTTATCAATTGCCTA

TTAGTATTCGATATATAAAAGAGCATATCATTCAAGTCTAGACTAGGCGGTTACACAAGCTCAATGGCTT

CCCTCGTATAAGCAAGAATATGGCAAATAATTCCATCCAAACACTGACCCGATTAAGAATTGTAGCTCGC

TACCATAAAATTCAGTTTCAATCGGGGAATAATAGGACTGAGCTGTTCCACCAAGTTGTCATAACAAAGG

ATCAATTTGTCCTTCCAGAATCTTGTAGTGTTTCCTTGATTGCTCTCCAGGT

>Ca_linc_0354

CTCCTCTATCACTTCTGGTGTTTTTGGTAAAGGACACTTTAGATTTAAACATATTAAATACCTATCCAAA

ATGTAAGCAAAATTTCTCGAGAACTTATTTGATTATAACTACCTGGTCTAGAAAAGTCTTTTCTACCATC

ACAAGATCATTAGCAAAGTAGAAATGAGATAGTATACCACCTATACCTCGACCGAACTTCAATTATCTCC

ATTTTCCTTACTCAACATAAATAGGTAGGGATAGATGGGTCTCCTTGCCTTAGTCCTCTAGTAATATTAA

ATAAATTAGTTCTTGCACCATTTCAATTCATATTTATAGAGGTAGTAGAGATGCAGTTCTTGATGACATT

>Ca_linc_0355

ACTAGCAAAAACTATAGCTTCCGCAGCTAAGTCAATTTTGAGCTTTTATAATTTGTATCGGAGAACTGCA

TTTGAGGAAAATATGCTTTCTCAGCGTTGCGAATTGGAAGTCATGGTTGTAACTTGTAAATTATGATTTT

CCTCACAATTGATTAAGGCAATGTGCAAGGTTAGTTCCAAATTCAATTATTTTGTGTACATATGTTTATT

TATTTATTACTACAATTTAAAACGGGTTACATCTTACGTGCTTA

>Ca_linc_0356

ATAAAAATTGGTATTTAAATTAACTCCTTATTTTTATAAGACTTCATCTTCAACATTAAAAGGGTGCTTA

TAAGCTTAGTTAGCACTTAGAATCACTTGTCCTAAACAGTTTATTAAACTGAAAAGGAAAAACATAAGAA

CTGGATTTATGATATTTTGTCTTGCTGCCTGCCCCACTCGGCACATGATAATAATATAAGAACAAATAGA

TGTCAGCCGATGCATGGCTCCTCCTAGCAACAGGAATAAAGGAATTATGGGATTTGTTGGACTTTGTCAA

TGCCAAAGCCAAAGCCAAAGCTAAGATTGATTGTGTGATAGGGAGAGGTATTTG

>Ca_linc_0357

CAAAGATAACAAATCAAATCAAATAAGAGTATCACAATATCATGCAACAACATGCAAGTTATCAATATTT

TTCTAACAACATGCAATCAAAATTCCTAAGTACACCTATGAATAACAAGGTTTTCTATATACATTGAAGG

GACCCCTAAGAGATGATTATATTCTAGCTTGTAAACTTCATCCTTGAACCCTAGCTCAAATTCATCTCCT

TCCTCCTTCTCTAAACTCCATGGACACAAGAAAAATCAAGAGTTTTGTTTTGTGTTGGTCTTCATATATA

ATTATTGTTTGTGGGAAAGGTTAGAGTTCCCCTTCTTCTTCCTTCAACGGTCTCTCTTTTCCCCTCTATT

TTTTCTTCTTTCTTTCTACCTCTCGGTTCTGTCTCGCTCTCTCCGTATGTTTTTTTCTCTTCTTTTCTTT

TGATTAGTAACGAATGAGAATTAAAAGAGAAAAAAAAAAAAAAAGATGTAAAGGGCTGCATGTATTTTAA

TTGGAGGAGGGAAGTGTG

>Ca_linc_0358

TATCATCTACATGAGAAACAGGTTCATTTTCTTTTTCATCTGAGTTGCTGCATTTGGAAACAGTAGCTTT

CCGAATAGATGAAGACCTTTTCATATTTTTTTCTTGTTCAGTCAAATCTGAGGAGCACGACCCACTACTA

GAACTAATAAACAACTTGTTCTTAACTCCTTTAGTAGCAGAACAAGGAGAAGAACAAGACCCATATTTTT

TCCTTTGAAAATTTTCACCTTTGTTGCATAAAGCCTCCATCTTGAACAAAAAAGTTATGATTTTTGAACC

AAAATGCAAAAGGGGTAGTTGTAAAACACACTATAGTATACTATTCAATTTAATCTCTTTCAACATGTCC

CAGCAGTGTAGTAAGTTTTATTTATAGATGAGTTTGAAAATTAGAAGGAAGAAGAATCATGTGAAGTGAG

TTATATAGATGGGTGAATAAGAATATTGAATATTGAATATTGAAGAGACCGTTGAGGTTGCTACGGATAG

AACATGATGAAAAAAGAGGAGGTAATGAACAACATTGTGGCTTTTTTCAGAAAAGTTTTGTGATAGTTAG

TTCAAAT

>Ca_linc_0359

CGATATTTGTATGTTTAGAAAAGCTCTATGAGATATGTGTGCTATAATACATGTAAATTGTGTTTTTCTA

AAGTTAATTTGTACATAATTTTGCTCCTACAAGTTAAGAAATGTCCGTAAAAGAAAAAAAGTCATATTTT

TATTTATTTATAAATGTACTAAAAAAAAAGTGCACAAGCAAGCTTTGTAAGCAAGACTTTAAATTGTGTT

GCAATTGCAATCGTGATTACATTGAAATGTTTGATATTACATAAAAATAAAAA

>Ca_linc_0360

CTCCATTCCCTTGCAACATCATTGTTCTTGATCTCGGCCAATTTGAATCATGTCATTTGCAATCCATCAA

GACCTTTAACAGCCATCATGGTGCCATTTAATTATTAAATTTAAAGTGGCTATTAGAATTTCATGTCTTA

ATAAATAATGTTTACTATACCTAAGTGATGTTTTCAAGATTGTGATGTTTTGTCATCGTTTGCAAGGTTT

TGTTCCTACCTATTTTGGTAAGTGCACTCTTTTAGTTTTTCAATTATGGTGGTTTGTATTAGTTAAACCT

TTGTAGTCTCAATGGATTGAATGTTC

>Ca_linc_0361

CTTTGATGGTGTGAACCAGAGCTGTGGTTGAAGATTGAAGGACGGATTTTGGGAGTGACGGTTCGCGCGA

ATCAGATATTGATTTCGATGCAAAAAAGAAACACAACAAATATACTTAGATCAAAGAAGACACAAATCAA

TGAAAGACGGTTAAGTTCAAAGAGAGTGGAGCGTCGCAGATCATAGAGAAAAATTGTAGATCTGAGAGTG

AAGGACAAATAATATCGATCAAGAGTGATAACGTGATTCTGAAAGCAATAGATATCGACTTGTTAGGCGA

GAGCTATGGAGAATGAGATCTTGTTTTTGGAGAAAGTGTGTGTTTTGAGAGAATGAAAACCCTATTTACC

AGTTTTGAGAAAACGAGAGTGTGTTTGAGA

>Ca_linc_0362

TATTGTGACTGTTTAGCTCATCTGACAGAGATCAATCTTTATATCAAACCAACTATTAATCTCTTACTTA

ACGGATTGCAAACATGGTAGATAGATGATATAAATTCATCACATCATACATATATATTGTAACAGTTGGT

TATTTGAACACATGATTTCTGACACAATATACAATTGAGTGTAGACATCGGTCCTAAGGTGAAAAAAC

>Ca_linc_0363

CCGGCGATGAAGGCTAAAAGCTGCGATTCACAATTCACGTTTTCACTGAAGGATGAAGGTTAAATGTGGA

AGGATGAAAACATTTTCACTGAACGATGAAGGTTAAATGTGGTTGGGTTTGATGAAAAACGAATGTTGAA

GGATGAAAAATGAATTTGGATTCGCGGGTTTGGATTCGTGGGTTTGCAGAAAGATGAAGAAGAACGAACG

ATGTGATGAAAAAATGAAAGGGGTTTGCAGAAAGATGAAGAAGAACGAACTATGTGATGAAAAAATGAAA

GGGTTGGCACGCGTGAAAGGGTTGTAATAATAACAAATTTG

>Ca_linc_0364

CTGGCAGCAGCCATTCATACAGGCTGGCAAGCAAGCATGCTTTCCTAAAGCCTATCAACAACAAAAAAGC

AATCTTGGCATAAATTGGAAAATTTGAAGCTAAAGCATCCAATAGAAAATAAAACCGTTTTACGCTTTAC

TATCACAACTAAAATGATCTCAACCAAAATCAACATCCACACAAACAAAATAAAATTTCATCTTTTAAAT

TTAAATTTTAGCTTCCAATCTAAGCAGCAAACATGAAGAAAATGAGAAGATACATGCCTTTTCAGATACC

GATGAAGACAGAGACGGTGGTTCGCGGTGGAAGTTGAAACAACGGTGATGCTTTCTGTTTCTTCTTCTTC

TCTTTCTTTTTATGTGTTTTACATCAATATTAATCTCAGTTTTCTCCTCCTCTCTTTGAAATTTGATAAG

GTGTATTTATAGAGTTTTTTTATCTGTTTAGTTGCTTTCTTTGCCTGTCTTGGTCCTTCTCTATCAATTT

CTTGTTAGATGTTTTGATTTCTTTGCTCATCATCCGCATTTGTCTGCACCTTTGATGCCTTCATTATTTG

TTCTGATATGTTCATTGCTGAGCTTTTATTTGTCTTTTAGTTTTTTTTTTTTAACTCTAAAATTTGTCTC

TTTGTTCTGCACAATGTGTACCAAGACTGATTTAAAAAATTATGTTTTTATGTTTCTCAAATTAGTTAAA

AGAGGAACAGATGTACCAATAAGCATTGCTTGTGCATCAATGGGAGTCATCATCAACATAGCAGGGTTTG

GCAGTGGACAAAGTCAACATAGCATGGCTTGATCTGATGTAATTTATTTTTGCCTTTATTCTTAGTTTGT

ACTTTGATTTGGTTTTAAAACTCTAATTAGATTTG

>Ca_linc_0365

CTTTATATTAATTGCAATAATAATAATAATAATTTGTAGACAAGAAGTATAAGAGAGCAATGATGGAAAT

GAAGAGGGACTTGGAAAACCAGCCACAAATTCTATTTCAACGCTTTAATTTGATGAATAACATTGAGTTC

CTAAAAGCCGTTTACAAATGTGTTTCTGTAACTATTACTGGCAATCTCAAACCCCATATAATTAAACCAA

CATAGTATATAATAAGAGTAAATTAATTGCAATTAAAAATAAACTATAGTCTATAGAAATTATTATTAAT

TATAACAAGATGAAAATGAAAAAAAAAAATGCCTGGCTTTCCCATGTACGCCGCTAGTGATGAATTCTCC

ACCACAAATGAGATCGATCCTAGTCATCAAAATCTTACCTCGTACCAACAAAAAACATCATCAAACAATT

GAAAATGAGTCTTAGCAAAGTTCATGAATTTCTTCTTCCTCTTCTTGTTCTTGTCCATGACCATCTAGCT

ATAAGTTCTCAATGCTTTGCTTATAAATATAAATATATAAATATTTGGCTCTAGAGATCCAAATTGAGGA

GTAATAATTAATTAACACAAAAAGGGTATCACAATTTGTTTGTGTTTATAATAAGAAAGACATAGAGTGA

GAGACAAAGAGAAAGGGAAGAGGTATATAGAAGAAGGTTAATGTTTTGTTCCCAAGACAAGAAGATGAAG

AGTTAGAGGAATTCCCGTTGATGTTGTTTTCAAAGAAGGTTTTAATTTTCCTTGCTGTGGGCCATGAAAG

AAGGCATTTTTGTTTCACACGTTGGGGTGGTTCTCTTTGTCTTCCCCTTCA

>Ca_linc_0366

CAACCATTAAAATTCACATAAAACGCAATATTCAAAGACTAGCTGTTCAACCCTAGTGATTACGGATAAA

ATATGTAAATAAAAATCACATAATAAAAGGATGGTATAAACAATACTTCAACAAACAGTGACATAATTAG

AATTTCGAAATTGCTGGGAAGAGGAGTTCCACCATACAAGCACCTCCAAAGACATTTTTAAATCCTTTAA

AGTTATTTAACTCATTCATCATGGACACTAGCGATCTGCACGGATTTATTGAACTTAATCTTATTACCTA

TCTCTCTCTCCTCCTTGTAATTTGAATTCTCTTTCTTTTTATTTATTTATTTTTCCTTCCCCCAACATCC

TTTGTGTTACCCATAGAAAAATAAAGGGATATAGTGAAAGCTACTAAAGGAAACAATTATATAACACATA

ACCACATAATTTTTTTAATGACAAATTCCTTATAATAACTCCTTCAAGTTTAGAAGTAAAAAATAAATAC

ATTTATTGTAGAAGATTACTAAGAATTAAAGAAAAATATAAAAAGTAAAACTATAAAACTCAAGAGAGAA

GAATAATAATACATGATATTACACACTATACTTTCTTAAATTTATAACAAATAATCTTGACCTAGATTAT

TAAAGATCTTGGACTTGCAAGGTCAAGAAAACTTGAAAATTATTTATAAAGTTAAAAAAATGAAAACAAA

GACATAAGAAGAATAAGCTCAGGAGAGATATCGCCATGATGTTCACCACACTTCCCTTTGTTTTTGTATA

TTGTAACCATCATCTTCATCTTGAAAGGCCATAACAAAGAGCATTGTAGCCATTTCCTCTATGAAACAGT

ACATGGTGAGAAAAACCAGAGACTGGTTGAAGAAGTTGAGCTAAGGAAGGTTAAACTCAACTTGAATACA

TTTGCATGTATGAGATGAGATGTAATCCAATGTAATGAAATGGAAGTATATATAATGAAAACTCAAGAGT

GTATAAGAAACTCTTGTGACCCTTTATGATAGATGAAGAAAATCAGAGGGAAGGAAATAAAAATGCAAAA

AAAGACATGAAAGACAGAAGCAGTGAATATAAGTGATGATTACCTGCACATAAAATTAGGGTTGATGTGT

CGCTTTCTAGCTAGGGTGAGACAGAAAGGATAATGAGAGTTTGTTTTTCTTGGGATTCAAAAATGGAGTA

CCCTTTATTCTTGCTAGGACCCTCTGCATACCCTCTTAAATTTTTATGTCAGGTTCTTGGCCTACTCATG

CTGACATTCTTGCTACCAAACAGCTAATTTTTTTTTTTTCATTTTGAACTGTGAATTGTTTCTTTGGGTC

ATTAGTTATGTACTATCATGTTTTATTGCATTGTACCATATTCTTTTCATTAGCTACAATTTTGTGCATA

TATTCGCATCCTCTTTAATCATCATGTATATTATATCCTACCCCTATGTCCTACTATACAATTAGTACAA

TCTTACGGTTTTGAAAATACAATTAATCAATACATTGATACTTTATAGGTGAACTTGTCAGGCATGGTTA

TTTGTTGCTGTCATATTGGTAAACTGTACACTCAGTTTAATTTTAGACAAATTGAAAATGTTCACATTGT

TTTTATCATATAATATGTTAATTATTTTCACGTTCCACCTAATTTTTGGTTTCTTATGATAATATTTTGC

TTTTCTCGGTTTCACACTGATGATAATCTTTGTAGCCTCTATATGACACTTGTTGCGTATGGGATTTGGA

AACCTGAGG

>Ca_linc_0367

CTATATTTTTATATAATTATGTGATATTTTCAGTAACAAATTATTGTCATGTTATTACATAATGCATTAA

ATTGCTCATGCTATTTTACCACAAACAATGTAACCTCTAACACATCTTGTGCTTATGTTTTATTGGTTCT

GTTAATAATTTTGATTCCCCTAGCTGTTAAAATATTATTGTTACATAATAGGTTGATTATTTTTGGCTAA

GTTTAAGGTTTGAGGCTTGTTTGTATTAAGAGTAACTTTTTAGAATATAGCTATCTATGTACAGGTTGGA

ATCTCCATTGTACTCTCTTCATAAATACATGTATATATTTACATTAACTTGATTTTGTAAAATTGATTAA

GTTAAAATAAAATCTGAAGTTAAATGTAAAAATTAAACGGTGAT

>Ca_linc_0368

ACTGCAACATAATTTTTATCTATTATTATCCAATTTGGATAAGGAAACACTTTACGTGGTGAGTTGAACT

ACTGAAGCATGTTCATAGTCTTGGTTTTTGTACCTATATTGCGAACATGAATTCTTTGCTATCAGCAAAA

CCCGAGCATCGAGAGTTGTTGATCTAATGGAATAGTTTAGATTTAAATACAGTATTTAAAATTTTAAAAT

AAAAACTTAAATCTACCTTTTCTTTAACCATCCTATAAAGAATAAACGGTCCACGTTATACTGACTACGT

GACCTCAGGTTCATCTGACGGCAGCGAATTCGTGTTGCAAAATTACACCTCATGAATCATGAACATACTT

CATTTGTTTCTTCCCTCTTGGATTCTATCTTTGTAATATTGGAACCATATTCTTCTTTAATTTTATATTG

TTTGATAAAGATGCAAAAGCATATGATGTTTGCAAGCTTCATGTTTTTCTTTGGTCAAACG

>Ca_linc_0369

GGCAAATAAATTAAATGTATAAAATTAGCCACACAACTACACAAACTCTAATTCATATATTCTTTCATAT

TTTCAAAAATTCAAAATCCTATTTAACTACCTTCTGTACAAGACTCTTTAATGTCTACAAGTCTAACTAA

ACAATCACTATTTTCTAATAATTTAATATCACAAACAAATTCAACCTAAACTCTACTACCATATATATCA

TAAGTAGTTATGTATTTCTAGTATATAATAAACAAATAAATAACTAAAAGAAACCATATATTATATATGT

GACTATACTATCTGAACAATGGATTTTGCCACCAAAAAACCTGTTGAGAAATCAAACTATCTCTTGTTGT

GTAAAGGGTTGGATCCTGTAGGCACTTTTCGTTTTGCTGATTTGAATTCTCTACCGTTGCTTCTTACCGG

AGAATTAACAAATGGAATATGAAAATTTTGGTGATGATTTGAAGTAGAAGGACTTGAGAATTTGATCCTA

ATAGCATGAACTCTTGGTGTGAGGAAAAGAAGAGAAAGGAGGAGAAAGAAATGGAAGTGCTTCATGTTTC

AAAGTATAATAATAAGAAGAAAAATAAAAACAAATGGATGGAAATGTGAAATTTGGTGCAAAG

>Ca_linc_0370

CTTCATTCTTCATTCATCGATTAATTTATAATCAATTAGTCATAATCTCTTTTTCTTTTCTTAACCAACA

CGAGAGAGAGAGAGATGGATTTTGAACTTCGAAGAGCGAGCGAGAAGATAGCGAAAGAGCAAAAGGAGAG

AAAGGAAAAAGCGAGATTGAAACTGCAAAAGGAAAAGAAAGCGAAAGAAGATGCTCGAATACAAAGCGAA

GCAATCGAAGCTGCTCAAAGATCTCGTNN

>Ca_linc_0371

ACATGGAAATTATGGTATTTACAAATGACATAGATTCTATTGGATAAATTTTGTTGTTGTTATATAATGT

GGGGATATGGTTGATATTTTGAGACCTTTGGAAACCAGGGGAGACTTTTCTGAATTTTCGGTAACTCGGG

CTTGCCAGGTGAAATATGTGTTAGGTTAGTGTTAGCTTGTGTGCCGGTCATGACCCAAGAGAGGGTTGTG

AGTTGTGACACTACCGTCACTGTTTATGAGGCAATTAGTTTATGGTTCACTTCAAATGGCTTTAAGTAAT

GTTTTATGGAATTAGGCAATTAGTTTGTGTCCCGTCTACATCCTTGATTACATTTCAACTTTCTTGTCAT

GAAATTGTAGGCAACTTGTAGTTGTGGTACACTATTGTTTCATTCTCTTATATCTGGCTATTGGTTGGTA

ATTTCACTCATT

>Ca_linc_0372

NNACGGAAATAGGGGGAAGACGTTTAGAGGCGTTAAGGACCGTTTGGAGAGCGTCAGTGACGGAGATCAT

TGTTTGATATGATGATGGGGTCAGTTGAGTATTGAATGTACTATAGTGGTAGAGAATCGTGTCTAATGTG

ACAGATAGTGTGACACTTATGATTTATTAAATTATTATTATTTTTATATATGTATGAAGAAAAGTATTTG

GAGGAAAAGAAGGTGACCTTTGTCTCTTGAAGGTGGCAGGGGTTGGGAGATTTTAG

>Ca_linc_0373

CTTCTCTAAGAAACCAAAGTAACCTAATATTTACACTCCTACCCACCTCTATCACTAAATTTTTCACACA

CGGCAAAGGATCTCTCGTTGTCTCTAAGGACTACTCTCACCAAATTTATAGCCACACATTATGAGTAATA

TGATTATTCTCACTAGATTCTTGCTGCCTTTCATGAATTGTCATCCATGTAGGAAACATACCCAAAGCCA

TTAATCATTTTGATGAAAATATAAATTTCTGAAATAAATTCCTTTAACCACTTTCATCTAGAGCTTCAGC

CTTCATAGATAATTACAGGTTTCCTTAGCAAAAATTTAATCTAACACCACCATTACCTTATATCTACTCC

ACATTTCACACTAATTTTCCAAAAAACCAAAATCTTCCTGTCAATTAATAAT

>Ca_linc_0374

TTTTGGAATATAACACGAGTTTACTTTCTATACATATCATCCAATCAAATCTTATCATCTAAATATGTCC

TAAATCTTATTTCCGAAAATACCTACACAAATATCAAAATGATAATATCTAATAAATTATTTTACATTGT

CTAGTCAATAAGCTCGAGGCATGCTTTGATGGTAGTGGAAATAACCATAAAAAAGTTGCAATGATACCTA

GGACTTGTTTGATTGTATTTTCCTTTTTAGTTTTTAAAACTATTTTATAAATAAATTTTAGAAAACTGTT

TTTAAAAAGAAAATAAAAAAAAAATATATTTGACAACTCCAATTTTTAAAATAG

>Ca_linc_0375

AAAAAAAAAACAAGAAAATAGAAGTGAGAGTTAGAGGAACTAAACCTTAATTACTTAATAGAATATCATA

CATTATGCCATACAAAACAAACAAAACACAACTATAGTTTATATAGAAGCTTACACAGATTAATTACTTT

ATAAATACCCCATCTCTTATTCTACAAATGCAGCACAAGTGCTATAACTCTTCATTTCTCACCTCATCAG

TCTCCTCCAAATCAACTATGCGGCTGGCGCTATATCGATATCGCCATCATCATCGTCGCCATCCTTCTCG

CTTCGGCCGGAAAAGTGGTGGAATTTGGTTGCAGCCAAGTCATTTCCCATGAGGAACATAGGAAGACCAG

ATTCCATGGAACTTAAGACCACCATTTTGCAGCACAAAAATGTTCTAAAGAATATTAAAGTGTTTTGGTT

GTAATGTGAACTAAATATGTTGACTCAAGATGAGGTCATG

>Ca_linc_0376

CGAGTCCTGATAAATCGTTGAAAAATACAGTTTCTCGTTCATCTCACAATATATTATACTCATAAATTCT

TTCGATGTGAGGGCGAATAGGCGACATTTAAGGTATCCTTCCGCTCCTTGGTAATCGAGAATGGCTTTGA

CATTTTCAATGTGTTCGTTAGGGTTAGTGGTCTCGTCGTATAAGTACATGTAAGGGGGTTTCTTCAACGC

TCTTGGTATTATGGAGTTCATGATACACTATGTTAAGGGTCCTAGAGACTCGCAAGATTCGTGTGGTTCC

CGATGAGTCATGTAAGCCAGAGACAAGACGAAATGTGACCTTGATCTAGTGACTCCTCTTCTCGTGATGA

TGATGCTAGCGCATCTCCCTAGGTGGGGAGTCCTCATAGGACCTTATGCGGTATCCTCGATCTAATGACT

CTTATGGAGAACATCTCATGCATTCAAGAGGCGATGACCTTTAGCCTCTTCTTCTAGGCGAAGCATTGTG

ACGACGTCGTCTCACAGAACCTTCATTGAATTCCAGTGTCTCCTGGGATGTTACCACTGTGGGGATGATT

GGTTGTTGGTTCTTCATAGTGTTGAGACGCCGGTTCTATTTCAAGATCAATCAAGGTTGTTCTTGAATCA

AGGGAAGGAGCTTGTCGAGGGTTTCTCTTTATTCTGGACCATCATGAAGTTGACATCCTCGTTAGGCCCG

TGTGTAGGACGAGAAAAAGTGTGTACATTCTCTGATGTCAGAGGTACTAT

>Ca_linc_0377

CTTTATTTCCAATTTATAAATAGAAAAAAGAAAGAAACAATTCTTATGACATTTTTATGGAACATGGTAG

AACCTTATATCTCTAAATTCTAATACAGTTGGAGATGACGACATTGGAAAATTTGTTACTCATAATTCAT

TATTAATTTATGCTTTTCTAGTCCAAACAGAAACATTAAAACACACCAAACTAGCTAATTAAGCAAAATA

TGAAGTCTATAATATAAAAAGCAAAAATAAAATACATTAAATTTGTGAAAAATGAATTAAGTCATTAAAA

TAAATATGAGAAGAGAAAACTAATTAAACTAGAAATCTTGTCAGAAGTGGCAACCACAAAAGAGGTAGAA

AAAGCAAACGGTTGAGACAAGAACAACAGCCTCAGCAACAGAGAGTAACCTATAAACGACAGTATCCATA

AATGTCGTTTGAAGATTATCAAGTCGTTTCCACTTAAACGCAGTGAATCTCAAAGGCCAAATTTCCTTCT

TCACTTTCTTCATAGTTCCCATCAAACGCTTCCTTAAACTTGTCTTTCTAATCTCTTTGCTTTGCTCTTC

CATGGAATTTAATTTCTACACATGAAATTGTTCTATGAGATAACTACTCTTTTTATATATGAAAAATAAA

AT

>Ca_linc_0378

CAAGCATCTTTGGGTGCTTCTCTGAGTCCTCACCACAATCTAAAAGATACATATGCAATGGTGATGTTTG

TGTTCTCAGAAATCCAAAGCAAAACATTCACAAAAAAGCTTCCACAACCAATAATAAGCAAAAGCAAAGT

CTTCCAATTTAATTTTCCCGTTTTTCTATCAAATGGTCTCCAAGGGACCAATCACACTTCTGAATAGTAA

GCATCATAGTTCTTCTATAAACAGGTTGATTGGTTTTTTTTAATTTTTATAGACAAATGTTAGTTGTTAC

GGTAAATTGAGTTTAAATCTGATTTGATTCTCAAACTCTTTTGGATGTTTCAACTCAACAAATTGATATT

GTAATTTGC

>Ca_linc_0379

GAAGAGTACGATGAAGATGAAAAATTCTGCCCATAAATTGATGAAGCTGTAAATTCAATAGGTATAAATA

ACTCCACTAGAGAGATGGTGACACATTGTGCAAATAAATCCCCCGTTTGTTTTTAAATGAGAACTAATTA

AAAGCTTGAGACAACCTTGATATGAATGGTATAATTATAATTATTGGATAGGTTTTATTGCCTTGTTGTT

GATTAGTCCTTATGCAGAGAAAGTGAAGGACTCAATTGGTCCAAGCATTCACGATTGTTTGCTCAATCAA

CTAAGTACAAATTCAAGGACAGTTGAAAATGTTGAAGAAAGACATTAAGATTGAGAAGGTTTAAATAACT

GTTATCATTGAATTAGTTAGTTACATCAGTTTGGAACCTCC

>Ca_linc_0380

ATGATTTTGATGGTCTCCATGCGTACCTAAACTGATCACCCATATGCAAATTGAGTATGTCCAATAAACC

TACGTGAACTGAGTTTGTATAAGTAAACTAATTTCATGTACGTTTACGTTGACTAAATTCACAAACATCT

ACGTGAACTCAATTCATGTATGTCTCACATACTGAACTGAGATCACATATACATGTAGGTGATCTGAATT

CATGCAACCTACATTAACTCAATTCGCGTAAGTGAACATGAACTCTATTCACGTAATGTCTATAAATTTT

TAAACTCCATTGCATACACAAAC

>Ca_linc_0381

ACAATGTTTCTCGTTTCTCTGCTATGCTTGTTGCAATGCATTCTTGATTCCGAGCGGTAGATCTGAAATG

ATACTAAAATGTCCGTGCTATGACTGTGAGGAATAACTATATTGTTTTTGTCATGTTTGAATCTGTACAC

TGTACAGTACTCTTGCATTTCGTGTTTATCAATTATGCCAGGCCTATTTGGTTGCTTTCACAACCGTTTT

GAAATATCAGAAACTAGCAAGTTCTGTTCTACTTCTTAGTTTTCAAATTTGGTGTACTTCTCCGGCCTCA

TTTCTGAAGCATGTACATGTGTCTAGCATTCTATTTTTACTGGGAATTAGGAATCGTTTAGGGTTTTCTC

GATCTGGAAAATGTTTAGAAGATCTTCTAAAATGTATAGGTAGGGGAGTTGGAGTTTTTTTGCAGTGAAT

GTGCCCCTTTTGTGTTTAGATTTAGAAATAGTCATGCTTTTTAGAGCACCCTGGGAATTAAAAAGTGTAA

CTCAATTACGTGACAATTGAGGAACAAAGAGACATGTACATCATTGCGAGCACAAGGCTCTGACCATAAA

AAAGCTGCTACAGACGTACAGAATAATGCCTTCCCAAGGCTCCTAGATTAGGCATAATTCATATATTGGG

AGAAATAACATGTGGGGATGTGGTCCACAAATCTTGTTATGTGTTATAATCCAAATTAAAGTGAAG

>Ca_linc_0382

GTAATTGATTGTAATTTGTTCATAATACACAATTTTTTTTACTGATAGTGTATGAAAATTAAACTCTAGT

TAGTATTATTAATATTCATAATAAAAGAAATAAATATAAGAAGTGGAGTTGAATTGTAGATTCTTTTCAC

AAAACATTTAGACTTACCTGCATTTTTAGGCCTCTATTTTAATATATTCACCAAAATAGTCCCTTTTATT

TTAAAATTGAACTTTTTAGTCCCTATATTATCACATTTGTTGCAATTTTTGTCCTGAAGCATAACTATTA

CTCTAAAAATGGTGACTTTTGATCTAAAAATTAGTGACTTTTGCTATAAAATTTATTTTCTCTTCAAATT

TACAAATGAC

>Ca_linc_0383

CAAGAGCTCTATGGTTACAAAAAGGATTTATAAAAGGGGTTCTAATTACAGTATTAATGGTTACCCTGAA

TACTGTCTTAGAATTGAATTTTGTTAGTTGGTTAATCTTCCTTTCACTTCCTTTTACATGTTCATTTTTC

TTACCATTGCTTTTGGCGTGCTGAAAATGTTTTCCTATTGCTATTCTTGATCATGCTCATGCTATTCTTG

ATTTTGCTCAGAGCAAAATTGGAGTTGATGATGATCAGTGTAGTGTTGTGAACTTGGAGTTTAAGAATGA

AAGACTACTGAGACAAATTCATTAAAGGAATTACTGGTCAGTATTCTTTATAATCAACTCTAACTTTCTG

TATAACATTCTTTATATGAATGAGTTGTTACATAAATAATTAAATATATACCGTTACTTTTAGTAAAATG

GTCTTAATTTAAATCGGTCTTTATTATACTTTATTAGCATGTTATCACGTTGACTTATGAGTTATGCGTA

GACTAATATATGCATTTCGGCTTCATCAAACCAAATTGTCACCAAATCTGTCTGCCTTAAAATTGATAAA

AACAGCTACCCAATATTTCTTGATCTGATCTACATAGTCTCGGACCATAAAATTGTGAACTCCCACCTGC

CTTTGGTTTGTGACTTTTATTCAATATTTGTTGAAAGAATGGGG

>Ca_linc_0384

CTATAGCCCTAAGTTGTTTACATTATTACTATAGCCCTAAGCTGTTTATATAAGAAGAAAACAAAATTAC

AATAGTAATAATTATATAAGAAGAAAATCAGATTTGATTTCTTTTGATTCTTCATTAAGGATTTTGCACA

AGAATGTGATAAAATGATAGTCTGAAGGCGGTGGTAATAAAGTGCACACTGTTAGTGTTAACAAGCATGG

CTGTTTCCATGATTAATAGTAAAATCTTTGTAGAATCACAAACTGAAATTGCAGAAAGATGGAAGCAATT

ACACTAACAGGTTCGTTGCGTCTTCATATCATTGATTTTAGTCATCTAAAATTGAAAAGTATTTGAGTTG

TACTACCATTCCTTTTATTTTGTTTTATTTAAATCGGATAAATATCCTAATTGGTATTGAAATTGTAGGA

CTATATCAATCTAGGCCCGGACATTGATATTTCAAAATGATCCCTAAAATTGTAAATTTTTAATCAAAAC

TTTCCCTATATTGATATTTGCAGGAACTAGAATTACATAAATTGGTAATATCGGGTTGAATTTGAAACTA

AGTGAACAATGTCGGAGATGCATTTGGTAACAGATTCAAACACTTGATTAACATTTTAGAGTTCTATGGG

TCATTTTGAAATATGATAATGTGGAAGACTAGATTGTTACGACCCTACCATTTAAATTATCAACTTTGGG

TATTTACTCTATTTAAATCTTAAAAATTAATCTTTTTTTGCAGAGCTTCTTTCTAATTAAATGATTGAAG

TAGGAGCGGGAGGATCTCTGCCTTCTTTTTAATAATAATAGGAATGCAGCAAGTTTATATTAGTATGTCT

TGTGCTCATGTTCTAAAAAAATGAATCCTCTGAATGTGTGGGTGTAGGTGGAACTATCCCTATTTTCATG

ATTTTATTAACATAGATGCTTATGAGTGTTATAGATGCTTTTCTTATGTACAAAATATCCAGAGAATTCA

GGTGTATGTTTTTGCATAATTGGCTTCAGTTAAAATTTCTTCAGATGGAATAATGATTCAGAATGAATTG

ATGAATCTTGAGGGAGTGTAGTTTTAAATATTTTTGGTTTGGTTATTCCAAAAATTTGCTCACTGTACTT

TATA

>Ca_linc_0385

CAAAAACGCATTCTCTAATTTCTCTCCAAACCCAAATATTTTCCTTCATTTTTTAGCAACTTAGAATCCT

CCAATCAGAAAACCTCCAACTACAAAGTTGTTTCACTACTTGAGAGGATTGCAAAGAGTTGAGGATCACT

AGTTGAGGTACAATAAAATTGAGAGGTGTTGTTGGAATCATCAGATAGGTTATGCTACCACAGCGTAAGT

TGCTTGTCTAATAAAGCACTGTCGAGATTGTCACCCTCTACTATTATTGTGGATCGGGCTTCCTCCCCAG

GTTCTTATAGTTTTGAGTTGTGCGAGAATTGCAACGACGTCGAAGACATGACAGAGTGATGATTTTGGAT

TGACAATCAAAAGCAGTGAGACTTGATGCCACTAGTGGACCCTCAGT

>Ca_linc_0386

TGAAAATACAAGAGGGGATGGAGAGTATAAAAAAATAAAGAAAAATGAGTGGGGTGGCGAGCGCAAACCA

GAGGGAGAGAAGCAACAAGAAGGAAGAAACAAAAATGACAAAAATCAGATGAACGGAGGGAAGAAGAAAA

ATAATATGCGAGAAAAAAAAAAGGAATGAAAAAATAATTATTTTCGGATTGGTTATCGATACGAAGATTA

ACAAGAATAAAAAAAATAATCGTCTTTAGATTGGTTCTTGATATGTAGATCGACAACAATAAAAAATGAT

CATCTTCATATGGTTACCGATATGAAGATTGACAAGAATAAAAAAAATGATGGTCTTCAGATTGGTTATC

AATGCGTAGATCGGCAACAATAACAAATGATCGTCTTCAGATGGTTACTGATTCGTCAATGGACAGGAAT

GGAAAAAATGATTGTCTCTTGATTTATTAATGGTGCTTAGATAAACAAGAATGAGCAATTGATCATCTCC

AGATTGGTTAAAGATGCATAAATCAACAAGAATATAATTGATCGTTTTCAGATTAATTATTGATACATAG

ATCGACAAAGATTAAGATGCAGTAAGATAAAGATCTAGTTTATAATTTTAATAAAGAGGAAATATATGTA

TATCATAATTCTAATGCAAACGTTAAAACATATAATGAGCAATGTACAACACAAGGTTGTTGATTATAAG

TAAAGTATGACTGTAAGACGCATGATTATGAATCTAAGATGCCTGATTCATGTTGTTATGCATTATAGAT

AAGTTCTTATTCAATTATTTAATTATTCGATCCTCATAGTCTTATGCAATCCCAAAATTCAATGGATAAA

TGAAAATAAGAATTATTTTCCCATAATGACTCCTTG

>Ca_linc_0387

ATTCAGATTGCAACCTATACAATTATAAACCTTTCTTCTTCTTCCAATCTCCTCCAGATTACAACTTTCT

CTAACCCAAATTCCCGCCGCTCCGTCAACTTAGACCGCTCTGGCTGCCGCCTTGACGATCGCTCTCTCTC

ATTCACAGGTTCTCTTTCTTCTTATCGTTCTATCTTCTTTCCACCGTCACTAGCTATTTCCTCTATCACT

AGCTATTTCCTTCTTCAAGGGTAAGTTATTTTCCCTTATTATCGTGCTCAAATTTCATCATTATCTAAAA

AAGCCCCAACTTTTATTTAAAATCCTAACAATTTTACTTCAAATTCAAAATTATTCATATTCTTTAGATC

TGATTCTGTATTTTTCTTACATAATGTTGTAATTAATAGTGTTGTAGTGAATGTTATGCATTGAAGGGAT

CAAGTAGTGAGACATTTTTATACATCATTTAGTTGTGCTTCTGCTATCATCTGAGGCTAGTTGAGTGAAA

CATATGACTCCAAAACAAAGTATTAGACTCAAGCTTCCTTTGAAGAAAAATCACATGATTTTACCGCATA

AGATAACTCAAGCGGCTTAGCAGGTACAAGCAACAATTAATCAATTGCCACTTGATGCCAATGCCACTAT

ACCAACGTCACAATATGTGCGGCATCCCACCAGCAACATGTTAAGCCTCATACCCAACAAGTTGCAGTAT

TTTCACTCCCACCTAATAAGGTGCATCAATCTGAATTAATTATAGTGATGATGTCCACACCGAGTTTTGT

CTCAGAAGACAACATTTTCCCGACTGCTGATCATTCACTGTCGCAACCATTAACATTTGTTGCCTCACAA

TTTTATTAAAAATAATTTAAGTAATTAAAATATGAC

>Ca_linc_0388

ACCTTGTCAGTGTTGCCTTTTAGAAGAAAAAATTCCTTTTCCAGCCATGTAGCACATATAGAATGACAGG

ATCTTTGAATAACTTCAGAAAAAACTTTGAGTAGATCATTCATACCAACAGATGTGGAAACACTTGAAAT

ATTATTAACCTGGATAAAAATAATAAACAATTAATAACAGTAATAAAATAATTGTAACAAAATCTTACAC

CTAAAGTCTAGTAGTTAAACT

>Ca_linc_0389

CTTTCTTATAATAAATCTATAATTTCAAGAGTTCAATTTTTTAGAACTATGGGTTCAACACAAATTTTAC

TATTAGTATAAAAGCATCGTTCCACAAATATTTTATATAACCAAACAAACATGCCTTGATTACAATAAGC

CCACAAATTGGGAATTTGTCTTGATTTCCCAAAACCAAACTCCCGAGTTAGAGAGAAGCGGGGGAAGCCA

TCATGGTCTCCTCAGACATTACTTATGAGTTTATGAGTAGGGTTAAGCCCACAATTCTACAAGTTATACA

TTAATGTAATTGCTTCTCTCATTACTGTGATGATATATGTACACCTGTACACCTATTTAACTTATAAAGA

CCTTTTATGCTAAAGCATGAAGCAGAAGTAAGCTGGCAGTTCTGTTAGCATGGAAATTTCTGTACAGCCA

CGACCAGCAGGTAGATTCTCCCCAAAGGAGCCAAGTATATCATTCTACTGAAATGTGAAAAGACAGGAGC

CATTATTACCAGTATAAATTTACACAATTCACAAACTAATTTACTACGTTATACAAAGTAAGCATAAAAT

AAAATA

>Ca_linc_0390

CAAATACCATCTAGACTAAACAAGTTCACATAACTCAAAACGCTAAGTCTAAACTAAAAATAACTAAGGT

GACACCTTATAATTTCAAAATCAAAATATTCTTATAGTGGATGACGTCAAAATAATGCTCTCCAAAAGTA

GCAGGACCCAATCCCAAGTGTCCCAAGGATTCAAGTCATGAAACAAAAAGAAAAATCTACTAGTTGAGAG

TAGAAGAAACGAGTGATAAAAATATCTAGTATCAGAGCCACGTCATCCAAAGTAATAACCATCTCCCCAA

AAGGTAGGTGAAATGAGTCAGTCTTTTTCTGCTACATTTACACAAATACAATATCATATTGAGATCGAAC

TTTGTCCCTCTTCGATATAAAAGTAGTATTTCAAACTTTGTCATTAGGATAAATAAGAATTGCACAATGT

TTACCTCTACTTCTAACCAAATAGTCATTAGGAGGATTGTGAAGTGGATCCTCTGAACCAAAATGAAGTG

AGATAGAGAGTGTGTAAAGAGGGTTGGTGGGGAAGATCCACATCCGGATGATTGTGTAAAAAGTGAAGAA

AAATTGAGTGTGGATAATATATCATATTGCAATGAGGTTTAGATATATCCAATAGATGAAACTTAAAATA

ATGAGTAGGTCTATCAAAGAGTTGGCTACACATCTTTTTGTTACTTTTGATTTTTCATCACAAGTTTAGT

TTAAAATCTTTTGTTGACTTGGGTGTGTGATGATGGCATTTTCACCCTTGTTCAGATTGATTTTGATTTT

TTTTGCTCATTTGGCAAAGTTGGTATGGATCATTGAAGACTAGCATTAGTGAGGCATTCTGTGATATGAC

AAATTTGGAAATCAGGGAATGATGATATTTTCTCTACAAAGGTTTGTTGTGTTGACAAAGAGTGGATCAA

ATGCAACATTTCTTCTTGGAATGGATTTTGGCTAAGTCCTTAGGATATCATCGTGTACATTTTATGAATG

GGTATCGCGTCCCTCCTTATGTTTCCTCGTTTGGTTTATGTTGTAATGGATTGGAGTAGGTTTTGCCTTA

TTTTTGCTTTGGTTATCTCGTTTGGTTTTTATTGTAATAGGTTGGAACATTCACTGTACTCATTTTCAAT

ATATATATA

>Ca_linc_0391

TGATGATGAAGTGGGTTAGTACTTTACACACCATCTTACTCACCTCACAAACTCAGCTACCTACTTTCAA

AAACAAAACCTCAACTACCCTATCCTTCTTTGTTTCTTTGAATCTCGTTTCTTTGTTTTTCTTCTTTTCA

TCTTTATTATATATTAACTACAACCTCTTATCCACATATCCTTTCATTCTTCCACATACACAAACACCAT

AGTAACCACATATTTCTTCCACAAATTAATTTTTATTATATATCATTTTAATTACCCTTTTTTTTACAAC

AAAAATTACATTTTACCACAAGATTAATGGAGGAGTCGGGAGAAAATTGGCCTTCTGATTCTGATTTGGA

AATAAGTGATGACGTCATATTTGAAGATGGGGAATATGAGGATCATTATTGTAGTGAGAGTGGAAATCCC

ACCGATAGAAAGAGAAGAAATGAGAGGGAAGTCGCAGAGAAATTGCTTGCACTTTCACCTACCATTCCTG

GCTTGAAGAAGGTTTGTAGTAA

>Ca_linc_0392

AAAAAACGTAAATGGGCTGCGCTGCAAATAACTGTGGCCCAATACCTTCAGAAAACTGATTTGTAAAACA

AATCCTAAATTGAAACAAGGCATAGGCAAAAACCAGAGCGGCGGCTAGGCAATGAGGGTCGCAGATCAAA

GGTTTCAAAAGAGAAAAAACAGAGCCATGCGAACTTCATCATCAAGACCGTAAATAAGAGAAAAAACCGA

GCCATGCGAACTTCATCATCAAGACCGTAAATAAGAACGGCGTTGGAGTGAGGCCGCTGGATTAGGTCGT

TGAGGAAGAAGGGGAGTCAGACGAACGGAGTGACCAGGAAACACGGTCGGCATTTCGGCGACGACAACGG

CGTTCAAGATAATAGTCAAGCAGATAAAAGGATGTAGAATAGAAGATGAAGGTGAGGTTGAGTGTTAGCA

GGGTGGAATGGATTGGTGTATTGGGTACTGATGTGTTGCCATGCCAAATCAGAAACATTGTTTTTATTGT

AATTCAATTCTTTTATTTTATGTAAAATAAAGATGTTTAGATTTTTTATGATAATTGTAAAATATATTAT

TATTATTATTATTATCATTATAAATTTATTTTATTTTATAGGACAAAATTGAG

>Ca_linc_0393

AAAAAATATAATTCCATTTTGAGCTAAAAATAAATAAAACTGACGAAAAGATTCGAGGAGCACAGTCACA

GTTACAAACACTTTACCCATTCCTTGTCCTTTCTACAATCAAATTCTGAACCCTTCTCTTCTCTCTCTTC

TTACCTTACAATTCAGAATTCAGTACTGAATTTGTTGAACGAACGAATGAATCTTTTGGTAACACAGAGA

AACAGTGGCGGTTAAAATAACAACAACATTGCCACTTCCACGCTTTTTTTGTTTTTCTCCAAATCTTAAT

GCCAACAATGCAATTAAAGTAAACAACATTCTTTGAGATTTACGCAACATCATCATCATCATCATTATTA

TTATTCATTTCTGTCATGTTGCGGTCGTTAAAAGCTTCCCATAATTCTATTCTCAGATCGGACTCACGCT

GACTGAATCAACAGAAGCAGACCTAAAGGTCCTACAATAAAATTGTGCTTTCTGATACAATGAAGAATAT

GATTTCACCCATTCAAAGTACTGTGCCGTCTCCTACTTTTCTATGGAGGTTCAAGGTCTGTTAGTAAATC

ATGTACAACCTGCATAAGAATATTTGTTGTACTGTACGTTTTAATTGCAAGCTAGTTTTTTGTTACAACA

TATGTGCTATAGCGATG

>Ca_linc_0394

CTTTGTTTCAGTTTTCGGTTAACAAAAGTCCTAACCAGCGTTTTTGCTGCTTCTTCGTCTCCGGCGATAC

TCTTCTGTTTCGGTGGTTCCACCACCTTCTTATTCTACCAGCCACAATGTCAAACCATGCTCAGGCAATT

GGGCTTTTGCAAAATATATACTTGGTGGCCCAACATTGGTGACTGTACGTGTCACATTAACGACATTTAA

ATCAAGATTCGGTA

>Ca_linc_0395

CTCAAAGTATACTACCGTAAACAACTCGGAGTATTGAGTAGTTCGAAACTTTCTCCTTCATCCTCGCAAA

CCCTAGTGCCTCCATTTTTCGTTCCCGCCGTTCTCCTTCATCCTCCATTCACTTTCTCCTTCATCTCCAT

CATCTTTAAGGCCTCAATAAACACCATCTCCCATTCCATTAGCATCTCCATCTTCGCAACCTTCAATGTC

CCCAGTAAACGCCAGAACCCTAACCTACATCTGGTGAGTCCTAAGCACACTTTCAGTCCAAATAATGGTT

GAAGACAGATAATACCAATAAAAAGCATGTCAATAAAGAAATGGTTTAAGGAAAGAAAGATGAAGAGGAA

AGAAGAACAGAGATTGCACAGAGCAGAGGTGCATGCAGCAATGTCAATTGCAAGTGTTGCAGCAACACTT

TCTGCTATTGCTTCTGAAAATTCAAAAAAGGAGGAATCAATGAAGATAGAGATGCAACAATAGCTTCTGC

TTCTATTTTGGTTGCTGTCCAGTGTGCAATAGTAGCTGAAGCAATGGGAGCTAAGAAAGAAGATCTTACT

AGTACAATGTTATTAGTTCTGCCATGAGTGGTACAAGTGCAAGTGACATCCTAACTCTCACTGTTGCAGC

TGAACATGTGAGTGTACAAGTCAGGGATAATGAAGATCATAGTGTTATTGTGAGTATTAAGAACCTGCTC

AAACACTTCAAGAGATGAAGTTCCAGTTTTCCACTGATTTTATCTGTTTACTTTGCTGCTGACCTAGTGC

TACCACTGCATTACACAACATATAGAGATAGTTTTCCACTGATTATAATTATTTGGATTTTTATTATTGA

ATAAAAGAAATATTGTATTTGGCATTTTTTTTTGTATTTGTAATTGCTATGTTTGTTTATTTTATTATTT

TGAATGGTTTTTATATTATGGGATGTACTTTTATTGGTGTGAAGGCAAACTAGTAATGTGTACAAGATTG

TGAAGGCAAACTAGAATAAGATTGTAGTTTTATTTGTAATATTGAGTACTTAGA

>Ca_linc_0396

GGGAAAAGAAAAGAGTAAGGGGAAGAGAGAAGGTAAAACAGAAAGGAAAGGAAAACAGAACGGAAAGGGA

GAGAAAATGGTGAGATAGAAAGAAAAAAAGATTGAGAGAAAGAAGAAGAGAAGGAGAAAAGAAGAAGAGA

GAAAACAACCAAGACATTAACAACCAAGAAAAAGAGAAAGGAAAAGTGCATTTTGCTAGAAGTCTCTGTA

GAAGGAGTTGAGGGTACCGTCATTTTCTCCATTTTTGGAAATCAAGAGGTAAGAAAGGCTCAGACCACAT

GTTGAAGAAATGACGCGTGTCAGTTTACACTATAGTCTGCTT

>Ca_linc_0397

TTGAGGGAGAGAAAAAGGGGACTAACGCGACCTTGAATTTCGATGAATTTGTAGTCGTTGAATCTGAATC

GTTGAATCGGTGGTCTCTTTTTCTGAATTCAAGTTTCGCATTCAGGTTTCAAGATTCTTTATAGTTGTTT

GGCGTCAATGTTTGGAGGCAACTCTTCTCTGTTTGGTTTCCGACGGTGACATCCCATTCTTGAAATTCAA

CCAAGAACGTTCAACTCGGTAAATCTTGATTATTCAAACCCTAGATATCTAATTTGAATTGGTACCAAAA

AGAGACGGATTCTGAATTGATGCTAGGGCTTCATACTGGTTTGCAAACTTTTTTTACTGCTGTGCTTTGA

GTCTGAAATTGACTTTCCATTTTCCTCTGTTTTCAAAGTCCTTTTTTTCTTCTTCTTTTTATTTCCTGCA

ATTGGTTATTTGATAGCTTGCTTTATGATGTCTTATTAAATGTCGAACCTATTTGGAAAATTAAAAATTA

TGTTACATGTTCAATTCAACTAATCAAGGTAGTGTGGTTCATTTGCATGATCCCTTGATTAATGGTTAGA

ATATAGCTATTTTTACACACTTTACATTAGCAACAAATATCAAGTTGATTTGATGTGGATGTATTTTGCA

AGGGGTAGAATTCTTGGATGCAAGATCAAGTAGAACATCAAGTTGTTTTACTAATGAAAGCAAGCACCAA

TTTTTTTTTGTCCG

>Ca_linc_0398

GTAATTGCAGTCCCCGAAAACATAAAGGCAAACAAGCACATCAAAAGTTAAGACATTATACGTCAGTCGA

ATTCAAGCAACCCCACAGTTTAGTGCACCAAAAATTGAAGTCATGATTGAAACTTACATCCTTAGTTTTA

ACCCCATTTTCATGAAAGCGTCTTTATCTTCTCGAGCAATATATTGACATTAAGATCCACCTGATTGAAC

ACAATAGAGTTTCACGCCATCCAAATCGACCATACACAAAACATCTAAATAGTGTCAAGTTTAGAACAAA

CTTCCTTTCCCATGTGAGAAAGACCAAAAAATTGATAAGCACGAGCCTCCAGATTTTTATGAACAGCACA

ACTAATTCCGAACAACTTCAAAAGTCTCGTCCAAACTTTCTCAAAGCAAGGGCATAAGATAACAGATGTT

GAATATACTATTCTTCTCCAAAACCCGCCACATGTAAGGGAGATGGAGACATAACAATACCTCTTATAGC

AAGGTTGTCTTTTGTAGGTAGTCTTTGTTGAAAAACCTTCCACACAAAGACGGGACGGATGATGCATCTC

TTGGCAAGGGACTAGATATCAAATTATACACCTCTTTGACCATATAACCACCTCTTGTCCTCCGATTCAC

CACCTTTGTCTGTAATAATCTCCTGGAATAAAAAATACTATCCAATAATAACACGCACTCACCCATCATC

TCCTTCTTCCACTCAAAAAAGCTCACATAACTTTCAATTTAATTTTAACAATTGGAATTGTATTGATTTT

GATCCACGTAAATTCTACTAATTTGAATAAATAGTAATGATCTAGGAAATTTTGAGAGTTCCAATTTAAC

ATTTGAAAAAAATAAAAATATGTTTTAGTCTTTAAAAAAAATTGAGTCAAATTAGTTTTTATGTACCACA

TTTATAAAATTATATTTTTTTTTGCTGACTTGTCTTTTGACTCATCAACTTGATTATATCTCACACGTAA

TTTCTCTAAAAAGTGACAATTAGGTATTTGACAAGAATTAAAAATAGACAATTAAGTTTATGACAATAAT

TAAACAAACAAACTTAGAGTTTTTGTTAGAAACTTAATTAACTATTTTTAATTATTGTCAAATACCTAAT

TTTTTTTTATATATAATAAAAATTACATGTAAATAATTAAGTTGACCAATCATTTGACAAATAAAAAAAA

AAACTCAATTTTAGAACTGTCATCCAAATAAGAACTAATTTAACTAAATTTATATCAAAATGAGTCTTTT

ATTTTTAAATAAATAAATATTATATATTTTTGAGTCCACAAGAAGAAATCCGGTTATTTGCTACAATTAT

TTCACCACTGATTTGGTTAGTAGTTAATGAAGTGACATTTAACTAGCAACAGTATATTGTTTTTAACTAA

CTATCAATTATAAACACAGGTCTTTTAAAAAAGAAACACTTGCCCATTCAGAAACACCTATCGTCCTGGC

TCATTCGGGAACATTGCGCTCCCTCTCTGAATTTCCCTCTAAGAAGTTTCTCGCTCTCGCTCATAAGAAC

ACTCATAATAAATTCCTTGCTCTCTCAATCTCACTCACCCGCACTCAGAATCTCTATCTCGTTTATAAAA

AATCTGGTTCTAATAGGCTTGCTTAAAACTAAGGGAGGTTGTGTTGTGGATACTATTGAAATTTTCTAGG

AACTATTGTGACTAATTGTTATTGTCGTACTTTTTTCTTTCTCTAGTAATTATATACCTCATTTTAGTCA

TCTCCACTGGCATATTACTTACATTGCCGATAAGTTGAATTCCTAAAAAATAGCATCCAGACAAAATATT

GGAAATACATTTTATAATATCAAAGGTGAAGAACCTTTTGGACAACTTGTGACGTTGAAGCTTAATGAGG

AGGCTTTGGGACAACTAAAAGGTTCTAGGCTCCAAGTTTCAAACTGGTAGCATTTTTCTAAAGTTACAAC

ATTGTTTTGTATATTATATACACTGTAACCTGATTCAGAGGTTGACAATTTGATACTTGCTTAGATTATA

ATTTTGAAATTCCATTTCTGACACTATATACATGGCAACCTGATGCAGAGGTCCACTATTTGCCGTCATA

TCTAAAACTCGGACCTCTCTTCTAACAATCGTAGTGGTGGCTTCTTACCATGAATATCAACAAGAACAAT

CAAATAAGATCCCTTCACTTTTCTTTTATTGTTAATTTTCTTGTAGTCTTTTAGTTGTATATAAAAGTTT

ACAACATAGTGGTAGAAAGTTGCCAAGGTAGTAGTCAAGACATACAAATACAAGAAATAGGAGCTATTAT

TTATTTGTGTTATTTAATAGGAAGTTGTAATTAGTACATGTGATAGGTGCTAAGTATGATCCATTTGATA

CATTAAATGGCTTTAACAATTTTAAATTTATGATCAATTGGCGTGACTAATAGAATTATGTTTGTGAAGC

TCAAGTTAGTAGTTACACTTTTTTTCCTACTGACATATATGATGGATCACTTGATAATTTCATATATTTA

TTGCATGTTTTTTCATCTAAAATAGGCAGCCATATTTTTTTGACTTGTGCACTTACCTAATGCTCGTATA

GCGATTCACCTAGTTGTTTCATATCAGGCACATACAATAAATGTACCATCATAAAATTTTATAATGAACT

GGTGCACGCATCAATTTTTTTTCTTCCTTTAGGAACAATTCTTACCACCGGAAAATGTATTGAACAAGAA

CAGGAATCCACATTAAAGTTAGAGCCAAATATAGGGTTGAGCTTAAGGCATATCTTTGAGTCCTAAAGGA

TGTAGGACTGTCTATTCTTGAAAGTTATTCGAAGGTAATGGAGAGACTGATATCTTCTTAGTGGCTCATT

TCTTTATATTGTTAGATGTATTGGAGCCTATTTTTTTTTTTAGAGTTTTAGAGATACTTGTTTTGAACAA

GTCTTTGAAGATTGAGACAATGACGTATATGGAATGAAATTCTAACAAAGTTTATATTACTTTAAGGTGT

ACTAGTTTTAGTTATATGTTTAATTTGCACATTTTAGATTTGAG

>Ca_linc_0399

CTTTGATGAAAAATGATAGGTAGTCTCAAGGTTATATAACATTATAACAATACTGTGTCCTTCTGCATTT

TGAACTGAGTGTGTAATTCAATAAATTTTCAGAATATACAACACATAAATGAATTTTCATCACAAAGTGT

AGCTTCTATAATTGGTAGAAGCAAGATTTTCATCTCTGCCTTCCAACGTCGCCTTTCAAATGCCTCTTCT

CCAACTCGTCAATGGTCTTTTTGTGAAGCTTGACAACATGGTAAAATTGATAGAAAAGTGCAGCTTCCAG

G

>Ca_linc_0400

CCGTGTCCAAATTCGTTTATTATTGATCATCCATCTTGTCTTATCTCTTCTTCTAAAAATTATTTGTTAA

TTAAGTGGCTGCGTAAATTAACATCATTCATTCCCTCACTGGATTTTCTGTTGCACACCTTCACTCAAAT

TATCACTCTCAGGTACACAAACACTTTTTTCTTCTGAATCATCATAAATTTGTATCCATTTTGGAATTGC

CAAAATTTGATTCCGTGATACAGATATTTATATTCCTTTATCAACCATTAATCTAGTATTAAATATTTTC

ATTATATATGTGTCTAGATTTCTTTATTACTGTTGTGAATTTTGAGTTATGAATTGAAGATGGTGTGTTT

TAGGTATAAAAAATAAAAGAAGAAAAGGGAAAGTTAGAGAATGGGGTTCGTGGTGGTGATATCGTTGCCA

TTAATATTGTTAATTTTGATATTGGCTATTGCTTGCTACTTGTTGGGTAGGGCCAGGGGTCGCAAACAAT

CGTCTTCCCATCCTCAGCAGTTTGGCCCACCAGCCCCTCCACCACAAGCCCAACTTAGTTAACAACAGGC

CCAAAAATGTTACACATTGGTTTGTTTTTATAATATATTCAAGTAGTTTGCTCATTGCACATGTACTCAC

AAAATTAGCTTTAGTCCTCATCTACCTTTCACTTCTCTTTATCATAATCTTTTTTAATATACAAAAGGAG

TGTTTAAAGTCCATTTTTTTTGTTCTAAAACCAC

>Ca_linc_0401

CTTCCTATAAAATCAGAAACTGCTAGCAGCGTTCATCACTGCCTAACGCTTACAACAATATTTTCATCAA

AGTGTTTTTATTTTTTTATCTGTAGATTGTCCATCAAATTTTGCAATTGTTTACTTCCCGATTTTATTTA

TGAATTCCGAATTTTGTTTTTGATATCTTGAGATCGCGATGATGACTCCATAAAATTCAAGCTCAACAGA

CCGGAATGCAGGGCTTTCTCTCTATCGAGAAATGCTTTGCGTGTCGATAACCCCGCTGAACTGAGCGATC

TCATAGATTTTTTCAACCTTCATCTTCAATTTTTGTTGTTTCTCTCTTAATTTTTTTGTAAAGTATTTTG

TCTCTGATTTTCAATATTTTATTTTTTTGTTGGATTGTGTTTTATGGGAAAAGCGTGTTCACAATGAGGA

TACAAAGCTCCCTCTTCTGATTTGAATTGAGGAAAATTGCAGGCAGAATCGAACCTAAATAAATCTAGCC

ACCACTGAGTGAACATTTTATCCCTTCAAGTATTTCCACTTTCCATCCGAAGAATAATTTTTAATTTGTA

TGTTATTTCAATATTTATTTACTTTTTTATGTGGAGGTCAGTGATGTGTTTAATTTGCCTACATTGTCTG

ATTATCTTTTATAGATTTGATGTTTTCAAATAACCTGAGACCTCATTCGAAATTTATTTGTTTTATTTTC

CAAATATTATGTGTTTAATATGATTATTTTGTCTGATTCGTTTATATTCAAGTTATTATTATTCCCGTAA

GTTTGCTTAACTCTGTAGTTTGTTTATCAAGTTGGATTTCAGTTGTTTAGAGGACTTATACGTTTTGTTA

TTTCTCTTGATTCCAATTTTGTGTGATTTTATCTTAAAATTATAAGTTATACGGGGTTCAAGTGTGTTTA

AATGTATAAATTCTTGCAATCTTTCTTCGAAGATGATTATAAAAATGTTGTTTCTTCATTTATAAAGAAA

TGTAGTTTTTGTTCTCATTTTCCTAGAATTGTTGTAATTTGTTTTTTCAAGACCATGATATGTTGTACTG

ATGGAGCGATGTGCGTGGTGGCAGGTGTTTGGATGGTCACTGTTATCTGTTTGTAATAATGATAATATCA

TTTCCCCTGTTCCTTAATTCTATAATCTAAAATTATTACTATTAAATAGAATTTTATTTCAATATATTTA

AAAGTTTTTGTAAGATATCATAATAAAATTCAATTTCGTTTTTATATTATTTGTTCAACTATTTACCACA

ACGCCACTAATTTGCAGACATAATTAAATAACTTCTTAAAAGCTTGTTGACATTGTCCGGATTTTGAATA

AGAAATACAAGTCCTCAATATTGGTCCTATGACAATACTTAGCCTAAATTTTCAA

>Ca_linc_0402

TGGGTCGCTCTTGTTGGAGAAGCAGGGCACGTGCAAACATAACTTTCTATTAGTTTGTCTCTCATTTTGC

ACGTGCTCCACTTTTCCAACTTGATTTTCCCACCTTCTTCTTCTATCTCAATTTCAACCTCAACTGACTA

AGTATCATCAATAACTAATTAAGGGTCAATTGGACTTTATGCATTGCAAACTTCTTTGGATTACAAGGAA

AGCTTTCAAGTACAAGCACTCCTCTTAGGAAGACACAAGCTCCTATGCTTAATGCATTCCAAAATGTTGT

TACTATTATTTGACATATATGCATTATATAATAATATAC

>Ca_linc_0403

GCCAGCGGCTGCCGCTGCTCCAGCAGCTGAGGAGAAGAAGAAGGAAGAACCAGCAGAAGAGAGTGACGAT

GACATGGGCTTTAGCTTGTTTGATTAGGTCCTCTATTAATATAATTTTGTCATGCAATACATTTTTCTTT

TGCCGTCTCTTGTCTCTATTTGAGGATTCATATAAGATAATGTTACTTATCTTTTTTCAACGTTTGTACT

ATTCGGTGTCATACATTAGCTTGTTATGGATTTTGTTAATTGGTACTAATCCTAAAATTGAGCCTGTTTT

AAAATATTTTTTAGTTAAGATTAATACATCTAATTGCGTCTTTTGAATGATGATTTGGTTGATACAAGAG

TATGCACTTTGATC

>Ca_linc_0404

GATTGTTTTCAAATGTATGATTTTGAATTTGTAATGGTTGATTAAATTTTGGCTTTTGCACTCAACTGGA

ATAAGGCTGTGTAATAGATTAATGTTATCATTGGCCTAATTATATGGTGCAAAAAGAAATTTTGGGTACC

GCTTTGCAGATCTAACTTATCATGTTTTAACTGCCAGTATAACCCTTACATTAACTATGAACTTTGATTT

TTTATTCCCAATAATGATAACACATTGAAGTTAGATAGAGTATTTTAGTTGGTAACCATTTTTTATTTTT

CTTTATTATTTGCTAGAAAAAGAAAACCATAAGGAGTTTGCTTGCAAAATTTAATATTTTTTTATAAAGT

TTTCTTATTGACCTAGTACTGTGTTATTTATCTCAGTTTTGTTTTGTTTTTGGTTGATGCCAATGCAAAC

ATGATCATGTGGCCTAAGATATGTGGCTTTGTTCATGTAAAATTGGGACTGGTTTCTGTATTGCAGCTTG

AACTTTCTTTTCTTTGCTGCCTTTAGATCTGTTATCTCTGTAAAGCTTACTCTTGCAAATATGATAGTAG

GATTCTTTAGTTAAATGTGCAGGATTCTGCACATTGAAACTTTATAAAATACAAACCCATTTCTTAATTG

AAGTATGCATTCTGACTAAAAAGGTTGTTTTTATTTGGATGAACTCTACTAGAAGGAAAGTAAATTGTTT

TGTATTGTCCTCAATCTGTATTTTAGAATGTACGTCGAAATGAAACATTGGACCCATTTTGAGGAAATAA

GAGGAAATTATGGCCAAAAAAATTATAAACGAGTATTCCAAGACATTTCAACATAATATGGTACTCCTTA

TTTAAAACATGATACCAACAAAATGAAACATGGTACTCCTTAATTTTGCAATGAGTTGAAAAGCTGGGTT

ACAAACATCCTAGGAAATATGAAAAAAAAACAATTCATATTGGGTGTTGAGAGTGCCTGAAATAAGTGGA

AAATGTGGCAACATAGGATTTGCTGAGTGTCTAGCTAGCAATATGGACCTTTTAGGATGTATGAACCTTT

TAAATAGGGAATATCATGAGTTTAATAGGATGGACTTCACTCGGTGGACTCTTGCTATAAATATAGGGAA

TAATATACGAGGCCATATTTGTTTTCTTTTAATGTTAATAATAATTTGTTTGTTTACTGTTACAAATTTC

TTTTTGGTAGGAGAAATGGATTCGTGATAGAACATTGAAGGAATTGTATTCTGGCTTATTTTTGTTAGAA

GAAAGTAAAGTATGATCACTAATATGAGAAGCTTTGTTGATGGTAAATGGTTGTGGATTTGGCAATGGGA

AATTATGCTCAGTGAACCAGAACATGGGAAGATTGCTTCATTGGAAATGATTTTGAAAGATGACTCTCTT

ATGATGAACCTTCATCACTCATTATTTGTAGAGTCATTTTGAATCAATTATTTAGTTTATTTATTTTAAT

TTAATGTTCGTATTAGAGGTATTTTATGCAATTTTGTTTTGTTTAATTGGTTTAGTGTAGTGCTAAATTT

TGGTGAAAAAACATGTCTCATCTGCAGTATATTAATTATGTCTAGAGTTGTAGACATTGAAATTTAGTCA

AATTAGTTGCAAATGAAAATTAACACCCACAGCTACAACTTTCATGAAGATAGTGAAATTCAATTTGGAT

TGTAAAAAGGTGAAAAGTGCAAGTTAAATTAGAGGGAAATAAATTTTAGAAGACCAAAAGTGTTTATTTA

ACACCAAATGACATTGTGTCTAAGTGCATCGCATTGTGTCCAAAATCAGTACATTATGCTTATCCATGTG

TCACAATGCATTTGATGCCACAATTTTCTCTCTAGAGCATAACAAATTGCATACTAGCACATGTCCCAAA

GCTCTATTAAAGCTAAAACTCAAGTTTTGTAAGGATTTTGACCTAGAGGACATTGGGATCATATTGAG

>Ca_linc_0405

GCCGTGCCTATGCCTATGCCTATTCATCCCTGCACAAACTGATCAAAAGCAAATGCAAGCTGGCTACTAG

TTGCTTTTACGGGATTCATCTTTCAAGACAAATTTCAACTTCCAATATATTATTTGAAATTTTTAATAGT

AACCTCATATTTAGTAAAAGGCAGCAAGGCAAATGAAAGTGATATGGAGCAATGAAACTAAAGCTCCACC

AAATAAACTACAAAACAAATGAAACACTATATTAAAAGAGATGAAGAACAAAGACTAACTTGAGAGACAC

AGATTTGTATATAGTGGTGAAGAAGAAGATGGCGGGTGTAACAGTTGATGGCGGTTGACGGCGGTTACCG

GTGGAAGCTAGAGTGACGTTACAAAGCAAAGGTCAATGACAACTACATTTGAAACAGAAAGGCAGCAACA

AAATAGAGCCAAACACTGCATCAGAAACAGAGAGGCAGAGAGGCAACAACAGCAACAAATTAAAAATTAT

TGTAATTTAATTTAGTTTGGTTTAAATCTTATTTTAATTTTGCCTTTATTTTAGTTTGTAATTTGATTTG

GTTTTAAAACTCTAATTAGATAAAAATGTAAATTTATGATACATAATAAAATTATTTATT

>Ca_linc_0406

CTAGTTTCAACTTTTCTTTCCAATATAAAGAAATTCGCAGTTTTAGTAACACATGAAAAGAAAAGCAAAC

ATGAAGAAGGGAGAAAGTACCTGCTTTTCCGGATACCAGAGGCCATGGTGAGGATAGAGGCGGCAGTGCA

CGACGGTGTGCAGCTCGATTGAAACCTCAGAATGTTCAAAAACAAAGGCTAGTGTCTTAACCATGATACT

GCATCAGGAACAGAAAGGCAGCAATAAGGACAGCAACAAAACAGAGCCAAAGCAGACTGGCTTAAATTGA

TTTAAAATTGATTGTAATTGTAAATTGTAATTTGATTTGATTTTAGAATTGTAATTGTAAATTGATTTT

>Ca_linc_0407

TTTCTTCTTCCCTCAGCCCTCACCCCTCTGACGCCCCTCAGCCCTTGCCCCTCAGCCCTTGCCCCTCTGA

CGTCCTTTAAGTAATCTCTCTCTCGTCCCTCTCACGCTCCTTAAACTTTTGCCTCTCGTCCCTCTCACGC

TCCTTAAACTTTTGCCTCTCGTCCCTCTCCCGCCCCTCAAGCTTCTCTCTCATCCCTCCCTCATCTCCCT

GGCTTCTCTCTCGTCGCTCGCTCGTCCCTAAGGCTTCTCTCTCGTCCATCTCTCGACCCTCAATCTTCTC

TCTCGTCCATCTCTCGACCCTCAATCTTCTCTCTCGTCTACCTTCTCTTCACTCGTCAATTTTCTCATCT

CGTTATTCCTCCGCGATGGAGAAAGGTTGGCGACAATGATCCAGGAAAATTAGATTGTGCTATGTTGATT

ATAAACAATTGGTGGTAGAGAATATGTAGTCTCAAGAGATGATTTCAATTCTTGATGGTGGCTGAGAATA

TGCAGTCTCATGGATCCCCTCAACTTCATATGTTAATTTTGAAGGTGATTTGGAAGTAAGTATCTCTTTT

GAGAATGTTGGGGATGAATTTGCAAAAAATATTATTGTTATGCAAAATCAACATCAAATATAATTACGAG

AATGAAAGATAAATCGAAGAAGCGAGTTAAAAGTGTAGTCAATCAAAGTTCGTTTGTGAACTCGAATGAA

AAGAAGAAAAAAAATAAGGCTTTGTAAAGTGTGTTGTTGGCATATTTTGTGTTGGTTGAACATTTTATAT

AACGATGTAGTTGATGGCGTTTGAACCACACTTTTTGTCTATTTTTTGTTTTGTTTGTATGAACAAATAT

GTATGGTAAAAAACTAGTGTGCATTAGATGTTGATTTTAGTCTTCAAATTGAATGTGAG

>Ca_linc_0408

CTTTCACTACTATAACCCATCACACTAAACCAATTCCTAATACATAGAAGCACTTCTCACCTTAACAAAC

ACCATTCCCTTCATAAAACTAGAACTACTCTACCGTTGAAAGTTGCCAATTTGAAATCAGCTAGCAAAAC

ATCACAATGAATTGCTTCCTTGTTTGTTACAATGATTCCTTCAATAGCCACTTTCAAAGTAATCATCTTC

TTCTTCTTGTGCACCCATTTTGATGTTAGTATTGTTGTCTCTGTCCAACACAAATTTTATATCTGTGTTA

AGGCACATATTCGTCACACACGTAAATAATGTGCATAATATTGGTGTTGTTACAAATAGGATAAAAAAAT

TACTAAATTGATGGATGCGGTGTTGGCCGCTGCATCCCTTAATAAAAAAGAGTTTATGATACATCGTAAA

GAAGGATGTCTACATAGAGATAGTTATTATCGATACAAAAAAGTTGAATCGCATATCTCCGACAACTTGA

AGAATGTTCAAAATCATTTGACCACATACTCCACAGCTACATTTGGTACCTGCAGCAATCAAGGGTTGAG

GATTTATGGTTATAATCAAGATACATTGTACGCGTAAAATCAATGGTTCCATATATTTCATGTTTGAGTA

TGCAGTTACAATATTTCAAAACTGTTAAAAAGAGTTACCAAAACAAAGTAACACCATCAGCTCAAGGAGG

AAGAAATTATGTTACCAAACCATTTGAATTTTCAGTCCAATTTTGCAATATCTCATGATATTGACCTTTT

TCCCTTAGTTTAGTTGACAAAACATATCTCGAAAGACTCTGAAAGACATATTAAAAAATAATATTCAAGC

AATCTAATTTCATTACCTTCGTGGGAATCTATAGTTACCTTCAACAAGTGTACCATTCACTTTTAGGGTT

GAGAATCAAAACACTATTTAGGGAAATTATACAATCTAATATGATTACCTTCGTGGGAACCTATGGTTAC

CTTCAACAAATGTACCATTCACTTTTGGGGTTAGGAATCAAAACACTATTTGGGGAAATTATAGTAGGAG

TTAAAAACTTTTATATTTTCGGTATATTATAATTATTTATAGGCATGATTTTATGTATAGTTATGTTAAA

AGTTAAATAATTCTAATGATTTAACGGCTGTGATTGACTGACAATGTAAAGATTCGTTATATTAATGTAT

ACATGAATTGAATC

>Ca_linc_0409

ATTTTGATTCTAATTTTAATCATAGAGAGCTTAAAAAGCTTACCCTCAGAATATGTTATAGATTTTGAAC

CAAAATATAAGATTTCATTGATGCATGAGAGATGATTATACATAAGATTCAAGAACAACAAAAAATATAG

AAGTTCACACTACAAAGAGACTTCTGAATACATATAGAAACTTATATAAAATCTAAAATACTTCTATCTA

ATGAGATTCTTAACAGAGTTTAGTTCCCCGCTATGATCTCCAATTGTTGTTTGCACTTTATGATTTTACC

CTTAGTTTTGGTGTTGATGATGTCGAGTTTCCAAGTCACAAAACTCTATTCCAACTCCAACCCTTCCCCT

CCTATCATAATATTATTATTTCCACTTCTATTTTTTGCCCTAATTTTTAATCATTTGTTGGATTTACAAC

AAGAAGCTGCAACTCACTTAGGATTTTACTCTCACCACTGGAAGACATGAGGAGGAATTCAAGTTGGTCA

TTTTAATTAGCATTTTTCCTTCAATTTCCTTGCATTTTTCTAAAAAGTATCAATTTCTTTGCATTTTATT

AGTAAGTATAGAGAAGGTGGGTGTTTTTTCAAAACTAAGGTGCTAGTGTCATTTAAGATGATCTCAAATG

AGGTAAATGCAATTTTTTCAAAAAAATTTCTAAATACCCTAAAACAATTCAAATGTGATCGGGTCGGTTC

AGTTTATGTTTCTCAAAAATCATACTGACCCAATTAGATTCTGTATTTGGTTTGGGCCTGAAATTTGCTT

CAAATTATTTAGTCATTTTTATGTTTGGTTGGCCAGGATAGATTGGGCCTCTTTTCCATTGGGCCTTAGT

GTTTGTGGGTGGTCTGTATCTAGTCTAAGCACAGTGGTAATTGAGTAAGTTTGTTTTATTCCTATAATCC

TGTTTAATTTAAGTAAAAGATTGAATAATTATTCAAACAAATAATAGTAGCTCACAAATTGAAGAATTGC

AAATTGTATTGGCTATTGCTCGGTTCACACTTTGATTAAGTTGTTATTTGTGCTACAAAAAGAGATATAG

TA

>Ca_linc_0410

AATGCATTTAGTTTAAATTTTATTTATCAATACCTGCGCCGCAGCACCTTTATGGTGCACCATACCCTCT

CAATCGCCGGCGACGCGGTTTCTGTCCAAACGTAAACGTTTCAACACTTCCACTTTACATACACACCCTC

ATCTCCTCCGTCTACTAAATTCGACGCTCTCCGTTCGAAATCTCCTCCTATTTCATTACGTGGGTTTCGG

AACAAATGTTTGGTATAGCATTTTATGAAGGGTGAAAAAGTTACCAATTATTTGAGGAAAATTGAGTTAA

TAATGGAAGTTATTGGTTTACCCTAACTTTATTAATGTTCATTTTTCTAACGAAGAAATGAAGGTAGTTA

TAAATGTTATAAAAACTGTTGTTACAGAAAATGGAGACCGTTATTATGAAATAAAATCACTTGAGTAAAC

TGGTAATCCTTCTTGTCATTGAGACCCTTATTATGAACTGTACAAAATATATTTCTGATCTTTATTTTAG

GTTTGATAATATCTTT

>Ca_linc_0411

TTGAGAAGTGTCATTGGAATCGTCAGATAAGTTATGTTGGCGTAGCATAAGTTGCTTGTGTAATAAAGCA

TTGTCGAGGTTATTAGAGTTTTGAGTTGTGGTGGAATTGCAACGATGTCGTAGACGTGACATAGCGATGA

TTTTAGATTGGTAATCGGAAGCAGCTTGATGCCACTCGTGGATTCTCAGTACCCAATCACCCTTAGTTAG

ATTAATGATCATCTTATGATTTTATTTAAGAATTTTATTTATCCTCCCTTTCGTATTGCAATGTTTTTGT

CGCAATGAAGTTTATGTATTTGAAACATTTGTAATTTTTTTAGTCAACGGGAATTGTCTTGTAAGTAAGT

CTG

>Ca_linc_0412

GACAAATCGTAGCATCCAATCCTCTTCCTTTTCCCTTTTGGAAGCTCGCTTCTTCTTCGCAAGATTACAA

CCATAACTTGATAATCTTATCATTGGTCACCTGTGATTATATCAAAGCGTTAAAAGTGATGAGTTCATCT

TTGAGTATGATTTTGCCATTCGAAGAATTTTTTCACAATGACATTACTGGAGAATATTTTTCCTTGGGAG

GAACAACTAGTTGTAAAAAACCTACTTTGGAGCCGATTGACGGTCGAGATACGGTGATATGTTCAAAAGA

CATTGCTATCAAACTTTATGAAAAGAAGTCATTACGCCATAGACTTCAGAATCATGTGCATAAAGTCTGT

GTACCTTTGAATTCTAAATCTAAAAGTCAACAGGTAAATCTATAATATTTAACAAAACATTGGTGATATT

TTATACTTGATCATGGTAGCAACCTTGCTCATTTCGTGAGTAGAATTTCTTATTCTTTATCTTATATCAA

CATACAA

>Ca_linc_0413

CAAAACAATACAAAATTCAGAACTTAATTTTATCTAATAAACACAAAGATTTACCACTCTAGCCCATAAA

TTGAGACAAAATTAATTCCAAAAGGAAAAAGAGGAGAAGGAGTTTTATTTTCTTCAAACGAACCAAAATA

TAGAGAGAAAAACTTTTTTTATTTTGTCTCTGAAACAAAAAAGAGAAAAAACATTGTAAAAACACAAAGA

AGAATAAGAGATTCCGAAGTCATTTTAATAATACAACCAACAAAGAGAATTGCATACGATACCTCCATCG

AATTGCATATGATACCTCCACCGTTGGCTTCTCAAGGTTCACTGGTCTTCATTCCCGATTCTATTCGTTA

AACTGCTCAAGCAATAAATGCAGCATCACTCCATGACTTATTAGCAGAACACTGTTTTGAAGATGGTTGT

AGTTTTACACGAAAAGCTAGAGCGGTCCTCATTATGTCTTGAAGTTCTGATGGATATATCCTTATGTTAG

TATCATTGGCCTTTAACATGTTTCAGGTAAATGAATTATGTCATATTATGTTGTAACCGATAAATATGCA

AACTATGAAGTTACTTGGACTAAGCATTATATACATGCCTCAATCATATTCTTCCTCATTTGAAGTGTTG

AAATTCCAAGTAATGAAGTACAATCATTGTCCCACATAAGGAAGTTACAAAAGCAATTGTCGTAGAAAAC

TTCAATGATCATTATGTATTTGTTTTATGTTGTTTAGTAGTGATACCAATAAGTGTTTGATATAAGGACT

CTAATATTAATTTACAAATTCTGATAAATTAAGG

>Ca_linc_0414

AATTTTTCAAGACATCCTAGCCACGCATCAGCTTTATGTCCAAGCTGCTAGCTGTATTCCCAGCTAATTA

TGATTTCAAACCCATTCATTTCAAAAGCAAAACAAACCATCCTTAGCCTCATTTCAAAACAAATGTAAGC

ATATCCAACAAAGCAAACCTCATTCCAAAACAAAAAAAGAAAACTCAGTTTCAATAAATCTACCTAAACA

AAATTTATTTTTAATCCTAAAACAAACTTCATATAATGTATCCTTTCATTTTAATAGTGAAATATAAACA

AGGATAGAGAATAGAACAACAAAAATGAGAGTGATACACACCGAAAAGAAGAAGGCGGTAGCAATGCGCG

GTTGATGGTGGTTAGCGACGATTATCGGCGGAAGTGAAGTTAAAACAGGAACAGATGTATCAATAGGCAT

GGCTTGTGCATCAATGGCAGTCAGCATCAACATAGCAGAGTTTGACAGTGAACAAAGTCAACATAGCATG

GCTTGATCTGATTTAATTTTTATTGTAATTTATTTTTGGCTTTATTCTTAGTTTGTAATTTGATTTGGTT

TTAAAAACTCTAATTAGATAAAATTGTAAATTTATGATACATAATAAAAATATTTATTGTAAT

>Ca_linc_0415

CAACGCTCTAACCAACAACACTCTAACCATTGTATCTTCATCACCACTCCCAAAAGAAAATATCAAATCC

TCATTCACAACCTTCAACAATCACCCATTTTCATCTTCATCCAACCACCCCTTCAAAATCATTCAAGGGT

TGCGGTCATGGCTTTTCTGATGTCAAAACCTCTCACTCACAAGAAACCTTAAATTTTTTATCTCATCACT

CTCACTAACAACTCATGCTCTTACGGCCACTCAACATTCATCAATAAACCACCACCACCTCCATCGATTG

TGCTTCCAAACCACCACCACCACCTCCATCCTTTTTGTTTCGAAGCCACCACTACGACCTTCATTGTTTC

TGTTTCGAACCGGTTCTTCCTGCGCAAAGCCACCATTGTTTCTTCTCATCTGTAAATGGAGAATAGATTC

CCCAAACGCAGTAGCAATTAATCACAAGAGGAAGGAGGAGATGAAGAAGAAGACAATGATGGGTGAAAGA

TGTAGGAGGAATAAAAGAAGAAGAAGAAGAAAATGACGTGTGACTT

>Ca_linc_0416

CATTTGTTTTAAACCAAGAAAATTCAAAGAAAGTGATGATCCAAAACATGTGTCTGGGGAATAAAGAGTA

ATATTAGTTGGTCTATTGAATGAAAATGTCGTTACAACCATAGCCATAAAATGAAATATCCACATGTGTC

TAAATAATGGTATTTTCTATACTTCAAACTAGGGGAGAACTATTAGAATAGTCCCTTAAAAGTATATGAC

ACTGTTATTTTGATATCTGACTCAGTAAAATACTTACATTAGTCATCGAATTATCAAAATTACAGTTTCT

AATGTTACAACCATCATAAAATATTTTGACGGTAAATTTTATCTTTCAAAGACTTTTATGATAGAAAGTT

GTATCTTTTAGGGACTATTTTGATAATTCGAGGACTAATTTTGTTTTTTTGTCGAGTTAGGGACCAAAAT

GACAACAAGTTATACTTTCACTTTGAGACACTAAAATAGTGGTTTTTACTTCAAACTAGTCCTCCCATTC

CACATAATTTGTAACGGATTGAAGTAATTGAATTAGTTTGAAATTAATCGAAGTTCATAGGTAATGTGGA

CAATAAAATCATTGATCGAATTTGTAATTGATCTGATTTATCAATCTGAACTAAACGGAGAACGTCCCAC

CAAGACGTCGAACAAAACAACACTTCACTCAAATGATAAAATTGCAGAAAACGCCAAAGAAAAACCAAAT

CTATATGGAAACATTTTATTTAGATAAAGTATGAGAAAAACCATGCAAATGAGTAATTTTGAGCTAATGA

AATGACTCCGAA

>Ca_linc_0417

TAAAGCATTGCATATACTTTTCTTTCTTCAAGTTAAACTGACATCGGTGTTGGAGTTCCATATATCAAGT

ATCCCACTCAACCTTCAAAACAAGTTGATCGAATATACTAAATTGGAGTGAGATCAAAGAATGATCGTGG

ATCTCCAACTTGGGAAGAAAATAATCAAACGTTTTTCTTTTTAAGATTGTCATGTAAGAATGCATTATGT

AAGTATATCTGATGCAACTCCCATTGTTTCGCCACGGCAACGGCTAAGATTACAATTCTCATCTTTGTGA

CATGCGCAATGGTTTCATTGTAGTCAAGGTCTTTTATTTGGTTATTACCCAAAATAATTCTCAACTAATT

ATGATTTACTTTTTCTTCAAACTATGAAACTAAGGTAACAAAGTGATTGTTTATGG

>Ca_linc_0418

NTTCCCTGAGGGAACTTCAATTGAAATATCAAACTGTGCCACACAAGGTAGGCAATGCTATGAGGAATGC

TAGCCCCGATGGTGATCGATACGGATTTGGATTTGTTGCATTCATACCGTCAGCACATTGGTGATTGTTG

GATTGAGATCGAATGGTGCAGAAAGCAAGCAAATTTTGATTTGTTTTTATAACTTCAAAATACCTATATT

TAAATTTGGACCGTCCGGTCTTGATCCAAGAATCACCGTGCTGACTGCGTGAGTGCATGAGATTTCTGAC

TGCAGGCAACATAAATCGGATCGGGACATTCTGACTCTGTTACCCTCAAAGTCCCTAAGAAGCTGCAGAA

TTTATGGGTTTGTCAACACCTATTGACTATGAATATGTTTTTCTGCAGCTACAATCTTATTGGGGGTAGG

ATAATAGCTATGTGGTTTAACGAAAACTGCATCTCAATTCTTTATAATGAGATATAATGTACTTAACATT

GACTCATACCACAGATCAGTAAAGAATAACAAGTGCTATTATTATTATTATTATTATGACATTCCTCTAA

TATATAACAATTTAGTTAATGTATCTAAAATTGTATAAATCCATCTTTTCTCCCCCCTTCAAACCCTCCA

ATTGTATAAACTTTTGCAACTCTATACCATAATAGTTTTATTTATCA

>Ca_linc_0419

CTTCCCTCAAAGTTCCTCCAATTTTGGAGAGCGAAAATGTAGTCATTAGTAATTTTGTTTCTAAATCCTC

CAAAAAGTTGAATTTGAACATCTCGACTTTTTATTGGATCTTCCAAATCATGTTCAAATTTTGTGTGAGC

TCTACTTCAAAGTATCCAAAATATGGTGATTTGGTGTGAGCATTGTGAGAAGACAGGGGGCTTGAACATA

TTATTGCTGGAACTACTTTATATGTGGTGAAGCCTGATGATAATCCAGAAGATAGTATCGGTCATGAGCA

GGATTGGTGAGGGTTTTTTGTGTGCCAAGCAACAAATATGAAAATAAAAAACCTTGTTGGGGACACTGAA

AATGCTAGTTTTTTTGTGTGGTCAGAATGGTTCATACAATATTCTATTTTGTCCAAGAACCAACCATTGA

TTATGTCAAATTGACTTTCCTTTTTACTATAAACCTTGATGTGTTATGTTTTATGTTCCTCCTTTATCAT

ATTTTTCATGTCGGTACTCTTGAATTTGAGTATTAAGACCTTGTTAAGCAAAGGGAAAATTTTAGAAGAT

TCCTTTAATGTTTGTTCTTGTTTTCTGACAGTAGGTATTAATATTACGCTTTACTAAGTTGGTTGGTAGT

TTGCTAAGTTATTTAATCTGTTAGCAATTCTGTTATAATTTGTTAGCCAATAATTCAAACTGGAAATGGA

TGCACAAGTGCCTAATGCTTGCATGAATGGAGACTCG

>Ca_linc_0420

GCAAAATGGAGGTGTAATGGATGTGGTGCAGCCAGCCCAGGCCCAAGAAAATCAGTCGGGCTTTGTTAGC

CACGGGACAGGTGTGTTCCATCCTGCCCAAGTTCTACCCAGTGCCAGCTGCAGCAATATCAAAGAAAAGA

GTCCTAAACAAGAAGGTGGAGCCAATTCTCAAAAGAAAAAAAAGAAAATGGCTTCAAAAGAAAAAAATGA

TGGATCAGTCAAGGTGAAGCAGGAAGAGTGCTATGATAATTCGTTGCCTCCTGGCATAAATTTACCCATG

GAGTGGACTTACTAACCCAATGGAATGGAATAATAG

>Ca_linc_0421

TGGGTTGGATAAAAATGAAAGAAAGGCTGCAATGTACTACTAACCTAGTTACCCAATCCAGTGAGCCATA

TCCCCAGAGGCTCCCGCACCCCTTATGAGTTTAAAAGTCTCACATTGGGGAGTCTCACCTAACATGTGGA

ACATATAAGTCTAGTGAGTTATTCCACAAAATGGACTAATCTCAGTACTCTCTTTCGGCTTACGCTCATA

GATTGAGGTCACAGAGTTCGCTAACCGAGATGGGTTCTAACTTCTAACAAACTAGAAAATTGAGTGAAGT

GTCATGGAGTGAAGACTGAAG

>Ca_linc_0422

CACTCCTTTTACAATTTCATTGCTGAATGGGATTTTCTTTTTGTTTCTATAAAAAAGAATAGATGAATGT

ATGGATCAGAACAGCAAATTTATAATGCAAGAACACCGACGTCTAACTATTGCCTTATGATGTATCCATA

TGAAGTGAAATATTAAGGTCGAACTGAACTAACCAACGAAAATGATGTATCCAAAAGTTTTGATCAACCT

CATCTTGAACAACTCAACTTCCATAGGTTGCCATGTCGCTCTCAATCTCCTTGCACTTAATGATGTTGGA

TTTGATCTTTATGTAATCAAAATAATAGCCTCTAATTCTTTGTTGACCTGATTCCTCGAAGATAATATGT

TTAAATCGCAAAACAACTTCAAAATATCGGCACATAGTGGTGGTATTTGTAACTAAATGTTTAAACTCTT

CCAATAGTTGTGGATTCTTCAGCCAGGCCAAGATCTCTGCCACAAAAGAGCTAAATGTTTAAACTCGTGA

GGAATGCACATAAAGAATTG

>Ca_linc_0423

CTTTGTTACAATTGGTAACTCAGAGAAGGGGAATTAGGGATAGAATAGAAGAATGCTATTAAGAATCAAG

GTGTTTCTGAATTGAATGTATTATTTCGCAATGAAGAACATGTTACAAGCTTAGAGAAGAAAATTCCTGA

TTCCCTATGAATCAGTCCAACCAATTCTACTTCTTCCCTCACCCCCCTCTAACTGCTCCTAACAGATTGC

TATTTATAGTAATCTGTTTTGGCTCCAATTTCTAACAGAATGACAGCTAAGCTCAAAAGAAAACTAACTA

ACTAACATTCTTTTTATTCCTTCTCACATATACATTCCAAAACCTATTTTTATTCGTATCAATACCCCCC

CTAAAAGAATGACCTTGTCCTCAAGGGGAAAAGTTGGAAATAGTTCCTGCAATTTAGCTGCACTCTCCCA

AGTACTTTCATAAGTAGGTAGATTCTTCCAAAGGACCAAAACTTCCACTAATCCTTGTTGATCTTCACGC

CAGTTTATTACTTGGAGTTCATACTCTTCATTCAACATAGGAGGGAGAGGTTGGGGCTTCTGATGGGGCT

GTAAGGCTTTTTTCAAAAGAGAGACATGGAAAACTGGGTGAATTTTAGAATGAGAAGGTAGTAGGAGTTT

TTAGGCTACTGGACCGACTTTAGATAGCACCTGACAAGGACCATAGAACCTTGCAGCCAGTTTAGCATAG

GGTCTTGAGGCTAAGGATTTGATTTTGTAAGGTTGTAATTTCAAATAGAC

>Ca_linc_0424

AACAACATTTCAGCATGCCTCTGATGAACATGATTTTTGCGTGCTTCTGATGAACATGATTTCTACATTC

CTCTGATGAACAATTATTTTGAAGAAATATGAAGTCTGAAGCCATTCTTCAAGCCAACCTCTGAAGCTCA

TATTCTGCTGACTATAAAGACTGGAACACATTCCAAAATGTTGCATCTGATGTTGCTATGACTCTAATAA

TGAAACAAAGGTGAAGTTGCTCAAGCCACTAATTCTAAGTCACACAAATTTGCAACTGGCTCTGACTATA

ATCAGTCAAACAACACATCATCTGACTCCA

>Ca_linc_0425

CAGCAACAATAAAATTTACAAGGCAATTAAATTAATACTACAACTATTTTTCTATTTCCAATTCAACCAT

TTTTACTATAGATTCATAAACACGTATTAACTAACAAGATCAAAATACATTAATCACTCATCCACAAAAT

TAACGTATAACATGAATTGCAAGAGGGAAGCCCTTATCTTAGGTATTTTTCTTCCAAATAACACATCATA

AACCCCACAATTCGAAGAACAAGAGAAAGGTCCAAGCTCTTGAAATGAATAATTTGTATTAAATTTCCTC

CTTCGATGAATAATATCAAAAGTGAGTGTTGAACTAAAATTACCTTGAGATGATACTAAGAGGAAGACAA

GAAATTAAAATTCAACTTGATGGTGGTGGTCGTGCATGTGGGCGTGAGGTTGCTAAAGGAAGAATCATGT

TTTTCTTTTCTTTTCTTTCCTTTGTTTTGTAGAGAAAAACAAGTGTAGGTGTGGAAAAATGAATAAGAAG

TCGCTTATCTATAGACCACCCTTTTAATTTTTTCCGTTAACTACACTTATCTTTTGTAGCAAAACAAAAT

CATCATTTTCGTCATGCTAAAGAGAGGGACGAAATTTCTATACCACAGAAATAGGATATTTTTGTTCTCT

CTATTACACAAAAGAAAAACCACAAAACCCA

>Ca_linc_0426

CTTAGATATAAGAAAAAATTTACAAAACAAACAAAAGACTCCTAGACACACCTAACGAGACGTCTCTAGA

GTTCAAATGCTTCCTCCATTTGAGAAATGAGAATATCCATCTTGTGCTCGACGTCTTGTAGAGCAAAAAG

AATGCCACTCAGCACTGAGGTCACCTTAGATTGTGAGATTTCTTTCTTCACCAGAGAATGTGACTTTCTC

TTCCTTCCAAAAGATGAACCTGCATCCATGGAAGAGAGTTTGTGAGGCGCAGAGGGAAGGATAAGCACAA

CAAAAGAAATGAGGACAAAGAGAGGATGGAAATGACAAAAATGAG

>Ca_linc_0427

CTTCTGCAAACCTACGAATCCAAACCCGCGAATCCAAATCCATTTTTCATCCTTCAACATTCGTTTTTCT

TCAAACCCAACCACATTTAACCTTCATCGTGAAAACATTTTCATCCTTCACATTTAACCTTTATCCTTCA

GTGAAAACGTGAATGAATCGCAGTTTTTAGCCTTCATCGCCGGCGAGTTGGTCCGGCGCAGCTTTCAGTC

AATTTCTTTCACTATCGAGAAGTGTTTGTGGCTTTC

>Ca_linc_0428

TTGTGAGATGGTGGGCATGGTACTGCCATGGTGGATTGGCGTGGCGGATTTTCGCACACCACAATGCCTG

CCATGGCGGGGCCATAGACCGCAATGGTGTGTTTATATGGAAGATTTTTGGTCTTCTGCCATCTGACAAC

ATTGATAGCAACTCTCTCTATCCCTAATCTTTGGTCTTTTAAGTTTTTTATTTGTGTCTCAAAACTTAGG

TTGATTTATGAAACCAAG

>Ca_linc_0429

GATTCATTGTTGTGCTTCTCTCATCATCCACGCTCCAGAATCATTGCGTCTAAGTGTGAGGGAGTGTGTT

GGAAAATTAGTCTTCATTGCGACAACCCTGCAGTCTGTTGTTGAGATAATTCATTTTCCTTCATAGCAGT

GAACACTTTTAGTGTGCTGGTTGAGTTGGCGGTATTTTTTTTTGAGATTGTTGAGTAGGTAGATTTAGTC

CCCATATATCGCCAATAGATATGGTCTTTCAAGTGCTTACAAGGTTTAGACAGTCCTCACCTTTACTGGT

TTTTTTTTTTGGGATGAGTAATGATGTGTGTCCGAATTCTAAGAATAATCGTAATTAGGATACTTGCAAC

ATTGGACATCACCTTTGGGGATAATCAACGTGTTGCCCAACTTGACCCTCGGTCTC

>Ca_linc_0430

AAGACATCTAACATAATAATATTAACATCTGTAAATTAACTTTAAGCTTTAAATACAACATTAGTTAGTT

TTAATACGGGTAAATGGAATATTATCTGTGAAGATAGCTTGTTTTCTTTGGTAAGGGGGAGTGGGATACC

AAGTAAGGTACTAAGGGTGGAGCTAGCTTCTTGTTTAAATATGCATATATATGTAACTCTATCCTCGAGG

GAAGCCTATCAAATTTACAAGTACACCCACCCTTATGATAATGAGATAATTTTATTTTATTTTTGAATTT

TTTAGTTGCTTTTTGAGAGTAATATATAAACACCTTTTATTATTACAATAAGCAATGTATACTTTAC

>Ca_linc_0431

CTTAAATTAAAAAAAGACTAAATATTAACAATTTTAAAAATTGTTAATATTGCAGAAAAATAAATTAAGA

GACCAAAATTATTTAATTAAGAAATAGTTGTTTCTGTAAGCAATCTTGCATGCTATTAAGGAAACTTGTT

AAGCTCACTCTTTTTGGACTCTCTTAGGACTACTATTGAAGACACTATTATACAATATTTAATTTTTCTC

AAATACTAGCTACGTAATATAATAAAATATTATTTTTACTTTATACCACAATAAATATTCACTCTCATCT

TTATCTTTTTTCTTCTTCTCTCTCTATTTTTCTCTCAGTTCTCGTTATACTTTAGGCAATCTCATGGATT

AAAAATAATTATATTTTTGTACAATTTTTTTTCAATAAGAGATCTTCTGGAACAATAAAACTCATGCATG

GCTCAAGATGAGAAAAAAATCATTTGCTCTTAGATAGAATAACAACAAGACGAGAAAAAAATAAATTTAA

TTGCAAATTTGTTGTCAATAATACTTAATAACGAAATTAGAGATAAATTTGACTTTTTTTCTGTTTCTAA

AATTTAGTCGATATTTCAAAATTTTCTTATATTGATTGTATGTATTTTAAATGGCAGTTCTTTATGTATG

TGCTTAGGGTTTCATGGTCGGTAAGTCAGATTGGCTTTCTTATATGGGTTGAGTATCTTTGATGCTCTCT

CTTTTATGATTATATATATATGTGCGCGCTCTCACTCCTCCCTCGATCCCTTTCTTAACATACTTTATGA

ATAATTTTATTTTAATTAGAATAAAAATTGAAATATGCGTTAAAACACTAAAATATGTTTCTTACAATAA

TTTCTTGTTTGGTCATTTTTAATAAAAAAAAAATAATCC

>Ca_linc_0432

CATATATCAATCATTCAAGAGAATAATTATTACATCTTCAAGATAGCATCTTCAAATACAACAATCAATG

ATCACACTATGCTATAAATCAGAAGCAAGATCATTTGAAAAGGAAGCAAAAATACAAAGACAGAATGGAG

AACGTTCTTGACAGAAGTCTTGAAAACGGAGAACTTCTCCACAGTTCTAAGATTTCAAGAACAAATTATC

TCATTTGTTGAAGAAGATGTTGCCCCTGTATTTCGCAATCATGTGATTTGTAGTCTTCCAGGAATATAAA

GGATTCATCTCAGCAAAGAATCAATTCATAATACAAAGTAGAAAACCTATGAATTATGATGAATCAAGCA

AGTTCAAGCAAGACTGATCATTGTCTAACTTGAATGTCAAGAACGAAGAACGTTCTTGAAACATATGTTT

CAATTGCA

>Ca_linc_0433

AAAAAATAGGGGTAATCCAGCTATTCTGCAACCGCTATCCCAGTTTTCGGGGTTGGCCGTGACACGCTGC

TACCTGGGATAACTACCTAGATTCAACACATATCTAAATAAAATAGAACCCGCTATACATTTAAAAATAA

AAACAGAGAAATAATGATAAACTGACAGTCATGGGAGAAGAAAAAAAAAACAGAAACAGAAAAACAATAT

CACAACATGAAACGATGAGAGATGTTAAAAGCTTTTGTTGCAACCAATGCAAAGCTCAATGTGTGTTTGT

TTTCAAATTCTAGAAGCACTCAAACCTTATCTGCTCCAAAGCAACAAATCGTTTTTTGGCTTGCTTCTCC

ATTTCGACGCCAACAGATTCCCAAGTTATGTAACAGCCACACTTAAGATTTAAGGTGTGTCACGTGTCCG

ACACCGACACATTTAATTAAATTTATTTTGTTAAATGATTATTATTGGTGTTCGAGTGTCTATGTCAGTG

TTTCATAACTTATAAGCATACCCTTGGTTGAATTTATTCTAGATTCTACCACAATTAAGAAATTCATACA

ACAACAATAACAAAAAGTTTAGTTTTAAATTGTGGTCAGCAACTACAATTAAAGTAATATTCGCAACATT

TGCTGCTGCAAAA

>Ca_linc_0434

TAATAATAAATTGTTTAATATATGTTGGGATATCAATAAAAATTTGGATACTAGCCAAAGATGAGGTTAC

CTATGCTTATTGACAACCAATTTGGAGTCCATATTGAAATTGACATTCTCTAGTTGTAGCTTATGGACCC

ACTGCAAAGTCGTCAAAAGACCAACAACTTATCCTATTTTAACAGTAGTAGAAAACCATTATGTTTTTGG

CAAGTGCAAAATTCCCATCTTCATCATGAATACAAAGGCCAACTCTTGTCCTATTCAAATGAGATGAAAA

CGCATCATCAATATTATATTTCACTGTCACTGAATAGCCTCATTTGAGACTTGTATCGTCGCTGATCTGA

TGCATATATATTTGTTGTACTTGCTGCCATTCAAACATGTTATTGTGTGAGAATATAGTTTGCGAGTCTT

CCGTTTGTCGGTTCAAAAGTATATTATTGTGTTGCTTCCACTCACCCAGAGCATGGTAGCAAAGCTAGCA

ACAACATTTGTATTTAGTTGGTGCATCATTGTGAAAAATATAGCAG

>Ca_linc_0435

TGTAAAAGAGTGAATATGAACTGTAGATTTGTGTTTGAATAGATTGAATCAGACAATAAAGTAATATGCT

TTATGCTTCAAAGACATCAATCTGCTTCTACATGAAGACATCAACTACCTTATTTGAAAGGTTGGAGTTG

GTCCGAGTTTATAGCCCCAACATCATAAGATTAAAAGTTGCTCAATAATTTGAGAGGTTAGAGGTGATAT

GAGCTTATAGCTTGCTCACAGAAGGTTGGAGCATATCAAACAGTAATGATTATGTGAAAGATATGTACCA

GGAACACATTATAATGAAGAGGATTGATTTCATATGTGGAAAATAGACAAGAACGTGCAGAGTCA

>Ca_linc_0436

AAGAAGAGGTGATCGCGAGACTCCACCTTTCTAAAGCATGGTACAGAAGCTAAATTATTACCAGTCGCAA

GGGCTCCATGTTTTACTAGTTCCTCCCTGATAGGCAGTCGCAACAAAATAATCTCCAAAAAAAAAAAGAG

AGAAGTTTTGATGGTGCCTTAGTCCTCCATACTTCTTCATTCAGAGCATGTTTGATGTTTTCTGCCAAAG

GTTCTTGTTCTTGTGCATTTAACAAAGCTGTTGTATAATAGCTTTTCACAGTAAAGCCTTCAGTTTTATT

CAGGCAGCAAAACCACTTATCCTTAGTATCTTTAATCGGACTAATTTCTGCCAAATTTTTTAATAACTCC

TCCAACTGAGTATTGCACAATACATCTAAG

>Ca_linc_0437

CCCAACCAAACCAGATAAAAAAATTAAAAGGCAAATCTAAAGCATATAGTCATTTGCAATTGCATGTATC

AGTATATAGTCATTTACATATATCATTACAAAGCCAATAGTCATGGCATGTATCGACAAACAAGGTGCAG

AAGATTCAGGTGACCAAAGTAGAAAATTAAAATATGTGATAGGATCAAGAGAAATTGAATGAAACATTTT

ATAAAAATCGTTACTGCTAAAAGGTATAAATGAAGTAGTGCAAACAACATCCATTATCTACAAACAAAAG

AAAGAACAAAGATAATCATTGTGAGAGTGTGAGTTGTCTGGAAGAAGAAGATAATTGAACCATACTTTCA

ACATTGTGAACACGAATTGGAAAACGGAATTGAGGTGGACGACGACACAAACTGGAACAGAGGCAACCAC

TCTTGGCGATGGATGAGAACATGAAGTGCTATAGAGGCAACGGGAACAAACACAACCAGAGATGACGACG

ATGTTGGGTTGTGACAGCGCGATGGAGAGAAAGTGCAGAACAATCATGAAAGTGAAAGAGGGTTCTTGTT

TTGGTGACAAGAAAAAGAGGTTTTCACAAACTCAAGTACTGTAATATTAAAGCTTTTCAATGAGTGAAAG

CATATCGTGCATCTCGAACTTAGAGAGAAATTTGGAGATTTGATGCGTGAGGATTTCTGAATGTGGG

>Ca_linc_0438

TGGAGAAGCATTATCGTCAAGCAAACGTCGTTGTCATTTCTATCTCTTCCCTTTCCGTCGAGAACTATGA

CAATCGTTTCTTTTTCTTTTTCTCTCTCCGTGAAGAAGTGTTGCATATTTCGTTTTTTTCTCTTTTCCTT

CGTCTTCTTCCCTTTGCTTTTGTTTTAACCTAAACCCTAAGTCGATGTTCTCTTTTCCTTCTCTTTGCTG

AACACAAGAAGCCAACAATTTTGAATCAAACTTGGTGTTTCTCCCAAAGTAGTTTGTTCCTTTCTCAACT

CTTTCAATTTTAAATTTATATATT

>Ca_linc_0439

GAGTATGAAAGAGAGAGAAAAAAGGAGAAAAGAAAAAAAGAAAAAAAGAGGAGATAAGAAGAAAAATGAA

GAACTGAGAGGGAATAAAAGGAAGTATTTTGTTTTAAAAGAAAAAAAAAGGATAGAAGAGAAAATCACAC

ACATGGAGTTTGGGTGTGAGAGAGTATAGAGGCGACAAAGGGCGTCGTTTACAGTGCGGCCACGAGCTCA

GTAACAGTGCGATGGCGGTGTACGTCAGAGTTGCTCCTAAAAAGTGAGGTAGTTGTGGTTGGTTTGAAGT

TTTCTAGCGGTGTTGTCTCTGACGGAGGTTGTGGTGATGGTTTAATAAAGGATTTGCAAAAGATGATGAA

AATAGTGGTGTGTGTGGTTGAAGCTCTCCCTAGCTATATGTGTTTTTATTTTATATTATGTTTCTTTATT

TTCTCTCAAAATCTCTTCCTTCTACTCTTTTATTTTCTATTCTTTGTATATGTTTCTTTTTTTCTCGTTT

GGTCTGATTTTCTTATTTATACTCAAAAATTAGGTTAAA

>Ca_linc_0440

ATAAAAAAATATTCACATTTATTTACAAATTTAACACCGTCACGTTTTCTTAACTAGTTCGTTTACCTAG

TCAACCGACCTAAACATACCTCCGCCTCACCTATTAAGTCTGGTGACACATAAACAACTTAACTCATTGT

ATTAAAAACTCAATTTTCTACACCATGTCTTATGCAATTAGAGCATCTCCAACAGTAAATTCAACATGAG

TTAATTAAATAGATCCACTATGCCACATCATTCATTCATTTATTACACGAATGGTGAACTCAATATAGAT

TCATTAATAAATTATCACGAAACAA

>Ca_linc_0441

AAAAAGTTAACGTTAATAAATTTATTATTATTAAATTATAAAAGTATCGTTCTTTATTGAGGAACCATAA

TAACAGTTTGATACAACTTTAACAGTACTTGCAAAAAACTAAATTTACATAATTACACATTGATGAACGA

ACATATTTGACAACCGTTACAGACGATAACCATCACCATGCTACTATTAACAAAATAGATTCATAAGAAA

ATGGAGAAACTGTTGAATAATGGTTTGATAAAAAAGACTTAATATTAATCCTTCCTCATGCATATACTCA

ATCATAATAATAGTAATGATAAATAATATAAAGTTCAATTGTAAGAGATACTTGAAAGTTATAAATCATT

CATAAAAATTAAGGTTGTATATGGGTTGGTCCAATCCAATTCAGTTCTGACTCAAAAATCTGTTTGGATC

AGGTAAATTAATGTAAATGGACTGTTGCTAAATTTTG

>Ca_linc_0442

GAAATATTTTTACTAAATAAAATAATAACTTTTAAGGCTTTTTATTAAATAAAAATATTATTATTTTTAC

AAAGCACAAACCAATTGCTTTAATAATTTACCTTTTGGGACCGACCCTTTTTCTCTAGAACCAACTCTAT

TATTGTTGGTGAGATTTGAGAATCAACTGTGCCAACCAAAAATAAATTAATATAATATTTGTCAAAGATG

AATCTGCCAAAGAGAAATATAGAAAAACAAAAATAATATATGAATATAGAGAAGAAATGGTGAAGGATGC

AAATTCCCATGACACATTAGTAATTTTTTCAAAAAGTGTAAATTTTAGTTTTAACTTTTATTAATTAAAG

ATATTAATGAAAAATGTTGTCAGTCTAGTCAAAAAGACATTGATATATAGATACTATACTTTGAATCCGC

A

>Ca_linc_0443

ATTTTAACATGACATTATTTTTTTATTATCTTTTTCTCTTCACCACTCATTCTCAGTCGCACTGGTGCGG

AGATTACAATTTTCCAACTCTCTGGTGTCACTTTTTCAGCATTATAATTGGACCCATACATGTTTTACTT

TTCTTTTTTTGGACTCACATAACGTTGTATTAGTACCACGTTTCTAGTGCGTGTTTATTCATTTGGGGAG

ATTAACAAGGCGTGTAAAACATTTGTTTTGGATAT

>Ca_linc_0444

TTTGTCCTTGAGTGAATAAGGTAACTAACATCCTTACTCTAATACAATCATCAGGAATACCAACAATCTT

GAAGTTGTTAATAACCTTCACAATCTACTTTAGATGCAAATGGGGGTCTTCGGTGGCATTTCCCTCAAAT

CGTCCACTTGCTTGTAGAATATAGACAGAATGAAGACATATTGAGATTATGTCATTGGTTTAAACTCAAA

TTGGGCTACTTCAATATCATGTCTCACTATACTTGTGTCAAGGGTTTGAGGATCAAATATAACACACTCC

TTGATTCCTCTAGACTTGTCATT

>Ca_linc_0445

CGAAAGATAATAAAATAAAAAATGTAAAAAGAAAAGAAAATATATGTTGTATTCTGCATACTTTGTGGAA

AAAACGTTTATTTTGGAACAAGAGTTGATGGAAAATCTGCATCTATGTTGCTCGCTTTCGCATTGAGGTT

GAACGAAGGAACATATCGATTACAAATGCTTGTATCTCAAAGTTTAATTAGTGCTACACGAGAAGATTAG

CATGGCCCCTGCGCAAGAATGACACGCATAAATCGAGAAATTGTCCAAATTTTTTTTACTTTTTTTCCCT

TCATTTCGATATCTTCTTCTCGCTTCTTCTCACTTTCTATGCTTTTGAAAATATTTTTATTGGTGGCTTC

CTCTTCTTCTGATGCATTTCATGTTTCTGTTCTAACTTTACGGAGCTACATTTCATCGAACTTGACAACC

CTTTTCATTTCATTATCACTTTTTGTCTGACTTGTTATTCATCTTCTTACTCTTGCTATTTTTCACAATA

GATAAGATTGTTTACTTAGCTCTACACTTTTCATGTTTATTTTTCTAAGTTTAGCTTTGCTTTTCTTCAC

TTTCATGCTCTTAAATTCTCGGTCCCTTATACAATTTTATTTGTACGCCAAATATTTCAAATGCAATGAG

TTGTTTTCTACATTTCAATTTTACTTGTTAAACCCTAAAATTTTGGGGGTGGGAAGTTCATCAAGCTAAA

ATAAGTAAGGGAAAAGATCTTGAATGTAATGTGTTTGATAAAATGTCATTGAAAAATTGTCTTAACTTGA

GTTGCTGCTGTTTACATTTGGTTTTGTCTCTCTTTCAGTACCCATTCAAATTTTAACTAAACACTTAAAG

AGTTGTTATTAGAAATTTGGAAAAAAATAAATAAATGCTAGTAGGGTCAAAAGAAAAACTTCATCTTGCC

AATACATTTTCAATATTGAGTGAGACAAGGAAGACTGTTTTATTTGGTTGGTTTAAATCTTCTACTATCA

ATATTCATTTATTCAGAATCGTTTCCAGAAAACTGTTAGGAGAAAATTTCTCTAGATTTGGAGCAGCTAA

GCTCTGGTTTAGGAGTTCTTGGAACTCTTATTTAGTTTCTTTTCCTGCATTTCCTAGAATTTCATCATTG

TAATAGCTTGGCTCATCAAATTCCAGCATTAATAAACACATTTCTTTCATTGAAATTATGTTTTCTTGCC

TTACTTTCTGGTTTCTATCAGTTATTATTTGCTGTCTTTTTCATTTTTAATTAAAAATGATACAGTTGAA

AGCATACTGTGTATCTTTTTTATTCCTAAGGTTTATAAGTACATTTTGGTCTAAGTCTCATCTGGCTAAT

GTCTTGTTTTGGTTTTTCAGAAATAATTGATGCTGTCTAACTTAGTTGGTATGTAATATGCGGGTTTAAG

TCTTAGGCTAAATGTAGGTAGTTAAACAGCCATTACTGCCTTGTATATTTTCATTTTTCTTGTTGGTTAT

CTTGTTACTCTATTTTGAAACTCAAGAGGCTGAAGCTCACTGTATTGTTTTCATATCTTTGAAATGTATT

TAAGGATTTTTACTTAACATAGTTCTGTGCAATTAAAGGATTTCGGGTAGTGTTTATTGTTTTTCAGGTT

TATACAGAGTTGGTGATGGAAGATACTGATCAAGCTGTGTTGGTGATCCAATTTTTACAATATATAATAG

TTACTTTGAACATCATGAAGGTTGTGCTCAGAATTTTCATCTCTAGTTCATGACATACCATTGTACCAAG

AAAAACAAACCAGGCTGCATTCAATTCATGGCATCAATGTATCACTAGACCTTTGTTTATCTCCTTGTTT

TTGTGCTACCGCATGCACTTTTGCATTCTCTTTAGGTAACCTTAATTTGCAAAACAAGTAAGGTAGAACT

AGCTGGAGAGTATATATGCAATCCGGCATGGTTTGCAAGCTTTTAAGGTAGATAGGACAGAAGGAGAATT

AGTGTATATTTGCCATGCAAGCTTGATAAGTAAGCATTCAGATTTTTTGTATATTATATCATTTTTAATT

AAAAATGTTACATTTGAAAGCATACGATGTTACTCTGGAG

>Ca_linc_0446

TGTATCTGAGGTTTTTCTCACTTCTCCCCCTTATATTCTTCTTCTTCTTCTTCTTCTTTTCTTGAGTAGC

CTCCGTGCTATATTTGAGTGTCAGTCAATCGACTGGCATACTGAGGGTGTAATTCTAGAATGGTTTTCTA

CAGTGCAGTAAAACTCCGATACAGTGAAAGAAGTTGCTCATGTTCTTGCAAATGAAGACACATCTTATCT

TTGTTTCTATATCTTAATAGAAATACCACATTGTATTGAACACATTATTATTAATGACATGTGATAAGCG

CCT

>Ca_linc_0447

GTAAAAAATCAGAAATTTTGTTCAAAGTTTGATTGAAGGATTGTGTGTTTGTTAATCTGAAGTTATGGTG

TATTTATACTCCATAAACATCTCTTCAGATTCGTGGCCATTGATCCAAGAGGTTTGATGAACAAATACAA

TGATCTAGAGCCATTTCTAATCTGAAAAATTGCATTGCTTTCAGTTGTATTCGAATACAGGATGTATGTA

TTCGAATACACCTCTTTGTAACGTTCAATTTTATTCAAAATTTGCACCTTGTATTTGAATACATAGTCAT

GTAGTCGAATATAGATTTGTTATTTTTGCCACTGACTTGCTGTTTGTATTCGACTACAGCAGGTCGTAGT

CGAATACAGATGCGAAGCTTTTGCTACTCACTTGCTATTTGTATTCGACTACAGCAGGTCGTAGTCGAAT

ACAGATGCGAAGCTTTTGTTACTG

>Ca_linc_0448

AGGGGGAATAGGAAGCAAGAGAAGCATGCTGAGTTGGCAGAGTGAGATGTTAGTTAGCAGTGGTGGTTTG

TCAATTCTGATGTATAGGGGAGTGGAGTGGAGAGGGAGGAAGAAAAGTTAATTTCTGTTTGTTGAAGTTG

TGGGATCTATTACAATATATTAAAACAGAACTCCAACATCATACGTTGACATGTGATTGGATCGAATGCC

ATGAAATCGAATCTGTATCACTTCAACAAAAATCTGATGTGTCAATCTTCTTTTACCACCCTTCATTTTC

CACCGATTCTATAC

>Ca_linc_0449

TTGTGAATAATAAGATATGCAATTTGGTTATTGATAGAAAATATTGGATCAACATGGCTAGTACAATTAT

GGTTGAGAAATTGGGACTTCTGTTTCTTGAGTATCCGCAATCATACTCACTTGAATGTCTTGAGGATTCA

TAATTTAAAGATATGGGTGAAGTAATGGTAATAAAAAAAGTATGTGTGTTTCAAGTAGTATAGCCTAATA

TCCCAAAATGTTTGACATTTATCTAAAGCCAATTACAACCCGAACGTCCTTCAAAAGTGTATGTGTTAGT

TGCAATTGTGAATGCTAACTAGAATTTTTCAAACTTATGGTAATGTGATGCATGTTCCAGGATTTGAGTG

ATGAAT

>Ca_linc_0450

TCTTGGATTTAATGAAATGTTTATGTTTTTATTTCCTTGATTAAGTAGATATTTTGTGTAGTTTTGCGTT

GTTTATTTGTTTTAGTGTAGTGCTGAATTTTGGTGAAAAATCAACATGACCTATGTGGAGTATTTCAGCC

ATATCTGGAGTAGTAGATGTTCAATTGGAGTCAAATTAAGTGCAAATGAAAGCCAAGATTCATAGATACA

ACTTTTATGAAGACACGAAAACCAAATTCGAACTCCAAAGAGGAGAAAAATGCATTAAACACCATACAAA

AGATGTTGTGCGCTTGAAGCGCGCATCTGAACACATTAAAACATGTTTTTATCACTTTTTAGGCATCTTT

TTTACTATTGGGAAGTTTTGAGACTTGTGCCCTAGTAGAGAAACTCAAAGACAATTGTTTTTTACACAAT

TAGAGCAGTTTTATTAAGAATCAATCATGAATCCTTCTTGTAATTCTTCTTTAATCCCTATCTTTTGTAT

CTCTATCATGAGTAACTAAACTATATTTGTTAGGGATGAGTGAAACAAGATTTGAGACTTTTATGATGAC

ATGGAAACCAAATTCGAACTCCAAAGAGGAGAAAAATGCATTAAACACCATACAAAAGATG

>Ca_linc_0451

AAACTATAAGAGTTTCTTTATAGTTTGTTTAGATTGCATTGAATCTTATCAAAGTTAGATAGGTGTTGTT

GTTGCTTTCTGGGTGAAAAGTAATAGAGATTAAAACATATCAAGGTTGATCTAAGCTAGGTGCTGTAAAA

CCAAGAAGGTTCTTTGTTTTAAACATAGTGGAAAATCTCACGGTTGTGAGGACTGGACGTAACCCGAGTT

GGGTGAACCAGGATATATCGTTGTGTGTATTTCTTCCCTTATCTATTTACTTTTAGTTTGATTGATTATC

AAGTTAATTGCATAAACTGAATTATTGATTTTAACTAACCAAGAACGTTCTCCGTTTTCTGTTTTCACAA

TCAAGATCAATCTTGAGTTATTTTCTAAAGTTTTTAACATGAATTTTTAAAAAAGTGCATTTACAATTCA

AACTCCCCTTTGTTGTAAATTGAAATTGCTACTTCAGTTCAATCATAAAAAGCTTTTATGTTATCTTAGA

TCTTATATTTTATATTAGAAGATGAGGTGGCCATAGGTGAGACGTTGCATCTAGCAATTGGTGGTGGACT

AATTGTATGTCTATAAAAATCACATGTGTGTTCTTGAATCGTATTTATAGTAGCATTCAACCTTTTCTTA

TTTTTAATGGATGTAAAATTGTGAGATCCCCTTGGTGTGGTGTTGGTTGTTTGTGATAGAAAGCCACAGT

CTTGTATTTGTTTTAGGGGTTGCCTATTGTCCTATTTTAATTAACTGCATGAATCTTAAATATGTGAGGT

TATAATACATTATGGTTTTTTATTGTATTTTCAAAACTTTTTCTCTGAAATGTTAATAATTCACGTGCAA

ATGGAAAAGACACATATGTTAATATGGTGTCCACACACAAACTAAATCATAGATCATATTTTAATATTTT

AT

>Ca_linc_0452

CAAAAACTAATGTTTGCGATAGTTTAAACCGTCACCAAAAGTACATAGAGGCAGTTTGTAACCGCTGCAA

TACAATCCTAGAAGAAAAAAACTAGGTGCATTTAAAGACTTTATTGGAGGCGGTTAGAACTCTGGCAAAC

ATTGGCAGCAGTTGGAACGACAATTTTTTTAACCGTCGCAAAACAATCTTGTGATGTATGAATTTGTGAC

AGTTGGAGAATTATCACAAACATCAAATTAAAACCGCTGCAAAATATTTTTTTCTTGTAGTGAATTCAGA

AACTCTATCATACCATATCTTTCTAGTTAAAATTTTTATTCTAAATACAAGCTAAGTTAAAAACTA

>Ca_linc_0453

ATATATTGTCAACCAACTCTTTAAATTAGGAACAATCATGAACACCACATACCCGTCACATATGGTCAAT

CACAATTCCCGAACTACTCACCAGTATGAATATAACAATTATAGTTTAAGACATCTGAAAGTACTATTGT

CCTCAAGGATTTAGTCTCTGTTACTTAATTTTAAATGGTGTTTTAGTATATTATTTTATAGTTGTTTCTA

TAATTAGTCTCGACCACAATTAATGTTGAATAAC

>Ca_linc_0454

CTTCTTATGTCAAGTTCTACACAATTTATTTTATTTAAATCTAGGGACCTTTGTTTATGTGGTTTTTTTA

CTAGGATTGAAACAAATGCAGATTTGTAAGTGAGATGTTTTTTAAATTATTGGTGGGCCTTGTAAGGGGA

AAACAATAGCATATGGATACTGTATCGGAAGTAAAAAACAAAACAAAGAAAAATGCTTCATGCATGGTAT

TGATAAAACAAAATAATAGCTGATGGATATTGTATTTTTTGTGTTTCTTAATATTAATTTTTATTGATTT

AAGGTTACTCTTTGTTCAACTTCATTGAATTATTACATGGTTGACCAACTTTTAATTGGACCAAACAAGG

CTCATCCATGTCAATTTAAGATTGTAAGTATCTTTGGTTTCCATAAGCTAAACTAATAGAAATTATTGGC

AATCACAATGGATACTAGAAAAATCAACCTTTTAAATTATGGTTGCATTGGTTTAGATTTCAGTTTTGTA

TGGGGCATTGTGGTATTAACATGGCTTTATTCATGATGGGTGAGCTTTTTAATGATAAATATTGCTGTCG

TTTTAAATTCCAACTTATTGGCATAAAATGAGTTGCTATAACTGTGCCTTCAATTATGATTATTTGAGAT

TCACACTAATCATGTTTTGTTCGGTTTTACAGTATATAAATTGATTATATGCTGTGTGTGAATTTACCTG

TTTTTTTTCTTCTCATCTCGAGTTTCTACTTAATTAATTTGTGATTTTGTTTGGACTCTACTCATTCCCG

CTTGTTTCCCCTAGAACCTGGCCAAGTTTCTTTTATCTTCAACATTATGCATCTCTCAGCAAATATGTTA

TTCTCAACTTTTTTCTCCATTAGTTAGTGCACTATCTTTGATGATTCGACAAATACAATGATTTTGACTA

AATTATTGAATATGTACATGAGACATATATAGATATGAGTTTAAACATTGCTCATAAACCACGTTCATGA

TGAAAAAAACACTAAGACTCTAAGGTGTCTATGTGGTGTATGAGTGTCCATACATCACTCTAAGATCAAC

CTAAGGGTAAGACTAT

>Ca_linc_0455

AAAAAATCAATCATTTTAAAATCAAGAGCATCCCAATTTCTTGGATACATCAAACAAATGTTCAAATGGG

CTTACTTGCTCTACATTGTCCTCTTCATTATCAAGTGAATTATTCAAATTAAAATCATCAACATCATCAC

AAATTCACAATGAACAATATAAACATCATCATAATCATTTTCAATGGACACGTTATCAAGAAATTTGAAC

ATCAACATTATGATTTTCAACTAAAATTAGTGGTTCTTTTATAAAAACTTTATCAAGAGCTGATGTTTGA

GATTGAGTTAAACCTTCAAATCTTTTCTTTTTTCTTACACTTCTCATATCAAAAATCACACTTTTTAATT

TTAAGAGGCATAGTAAAAAAAACTTGAAATTTCTCACAATTGTGTCAGCTAATGATCAACTCTACAACGT

TTACTAGCAAACAATCTAATAGATTACGAGCAAACAAACGATCTTTTAGTACCATTGTAAATCACAAAAT

GAGTAATGAGTTGTTAAAATCTAAAACCCTAACATTAGATTAAACTCTACTAGATAATGAGTTCATTCAA

GATT

>Ca_linc_0456

CTTTTTTCCTATCTCCACAAACTTTTCGTCACAAAGTAAAGAAAGAAGAAAACTCTAGCCTCCTTCCCTT

GCTTTTCTCATGCCATTTCCAATTTTCTTCCAACAGATGGTATCATCTTAAGGTAAATTTCTTCCATAGT

TTCTTTTGATTTCTTTCACTAAGGAAAGAAAACTAGTACAAGTGCTCATTAGTAAAACTAGAACCTTTCT

CTTATTCTATGATTGGTGAGTCTTAGAACGTGATGCTAGGAAGAAAAACAGCTAAAGTAAAGGCTTCTCC

CAATGTAAAGTTAGTAATTTAATGAATTTAAATTAGTGGTTTCTTAGATATTGATATGTCTTGATGTCTT

AAAAGAAAAGTGGTG

>Ca_linc_0457

TATGTATGTTATGATGTATTTATTTAATGTTTTGATTTCTATGTATTTTAATTACAAAGTTTTGATTTTC

ATGAACAATAGTCTTGAGGCAATTGATGAAATTTTTTTATTTTTAATTATGAGAAAGTTGATTACTATGA

TGTGGTCCGGTGTAAAGTTCGATTTTTATGCTTAAATGACTAATAATAAGCGGAATTTTATAGTAGAAGC

AATAAAGTTTGTACTTTTATGTGTTCTTGGTGATTTAAGTTTTAATGTTTATCAATACCAAGTAGCTAAT

CAAATTTTAAGGTTTATAAAATGAAAGTTGTGATTTTGGGATATTTATTCATGTGTATTTATATGTTCTA

TATTTTGGATATTGAAATTACTGATTCTTGACTTTGAGCATTCATGAGAATATAAGTTTGATGTCATATG

TGATTAAAATTTAATATTGAATAATTTATTTTTGTGTTTGACTAAGTGATTTGTGTTTTGAATTTTTTTT

TTCTTTATTGTTATCTAAGTGGCTGATGATTTGTGTTTTGAACAATTTGTCTTTTGGTTGCCTAAGTGGT

TGAAATTGTGCTTTGTACATTTTTTGTCATGTGGCTGGTGATTTGTATTTTGAGTATTTTATCATTTTGA

ATGCCTAAGTGAGTGGTGATTTGTGGTTTAAACTTTGGAGTGGCCTAAGTGGCCGGTGATTTCTGTTTTG

AACATTTTGTCTCGTGGTTGCCTAAGTGGCTGATTTGATTTGTGTTTTGAACATTTTCCTCTTTGTGGTT

GCCTAAGTGATTGGTGACTTGTGTTTGAACATTTTATCTTTGTGGTTGCTTAAGTGGCAGGTGGTTTATG

TTTTGAACATTTTATCTTTTTGATAGCCTAAGTGGCTGGTGATATATACCTTGAGCATCATTATCATGAC

ATGACATATACATCAGTATGGGTTACTTTTGTGTGGACGACTTCGTGATTGGTTATATATAGATCATATA

GTTTTAAGAGCATGCATAACATGCATATATTTTTATTTTTACTTTAATCTAAATATGAATTGCTACGTGA

TTTAATCTAGTACATATTAATCCTTAGTATAAATTTAATCTCAACACATTTTTGTGAATTATTCTAAAAC

CGATTTATTTAAATCTTTTATTTCAAAAATATTTTCTAATTATAATTATGACTCTTAAATTCTTATTTGG

TTTAATTTTTGTCGTGGGATGTGTTGATTCCTTACACCAACATTTTAGATACTAATGATCATGAAGACAT

GCACGATTGAATTATGTTTGTTTAATGCGCGCGTGTGGTACAAGGTTGTTTTTATTTAAAAATAACGTTT

TTGTATTAATAAAACCAACGCAATTCATTTTTCAATATTATTTACTTTTTATAGTTAACTTTAATTAGAA

ATGCAAAGGTGGATTTTTCTTGTGTTAGGAAATAATTACATCATCATTAAATTTAGTAAACCTTATTTAC

ATTTGGAAAGAAAGACTCAACAAATTAGATTAGTAGGTGAAAGAGCTCTTAGAAATGGAAAACTATGAGA

TTTTAGAAAAATTGAATTAATACCTAAAATCTACAAATAACTTTCTTTGCTTCATATTCATAAAGCACAT

ATGCATATTCAGATTGCCCTCTTAGAATAGATTTGGCATTTTAAAAAGCCATTGGTGGAATAGTGTTGGA

TGTACATGAAGTTGAAGACATTGCAGGTTGAAGAATTTTGTGGATTGAAGAACTTAGGTTTTATAAGGTT

TGTTTAGAAAAATAGCAATAAGAAGAATGGGGAAAATGAAGATTTTATGAAACTTTATAGTTGGAATATC

CTCATTTATTTATGGTGATGTTTAAGTGAACAATCTAAGATGACATATTGCAAAAACATGGGTTTCTTAG

GAAATTGTGCGCAATTACTTTTTTTTACATGTCGTCTTCTCTTGGGGAGTGGGTAAGGTATAATCGTGTA

TCATCATTGTGTTAGGTTGAAAAGCGTTGCAATTGCGAAGTGTCATAACAGCTATATACAATGGTTGATT

CGAAGACTCTAATTTTGTGTTGCTAAAACGTAAAAACTGTCAATTTCGAAGTACCTTCAATTATCAACAT

TTTTAGGGGCATCTATTATACACAAAATTTTAATGTAGGATTAAAAGTCAATAAGTGGGCACTTTGAAGA

TAGAAAATATGATAGCTCTAACGATAATTTTCTAGATAAATGTTGAGGTTAATGATGGAAAGAGTAAGAT

TTTAAGAAATAGGAAAAGCACTCGAAGAAAGATTAGTAAAGACATGAGTTCGAACATAGAAACTTGTAGG

AGAAGTTTGGAGTTACTTGAAGAGGTTTCTAAAGGAGGAAATATTAAGAACACATACCTTACATGCAAGA

ATAAAGCAGTTACCGCATTTACCTATATATATAGGAGTTGCGTCAAATAAAAAAGGCATTCATAGTGTCA

TTAAAGCATAAATGGATTAATATCCGCTCTCAACAGAATTCTCCA

>Ca_linc_0458

TGTTGTGTAGAAAAAGAAGAACTGTGATATTTAGCAAATTCGCAAGTTTCACAATGAAATCACGATGGAT

CCTTCTTTGAAAATAATATAGGAAATCAATGTTTCAAATACGCAAAACTAGGATGCCCTAGCACTAAATG

CCTTATCATAATATCATCATTATTAGAAGAAACAAAAGTATATTCAAAGCAGGTACTTATTTGAGGTTGG

TATTTGAAGTCGAGTTCATTGTCAAGATAATAGAGTGCTCCACTCTCCTTAACATTGTCAATTATCTGCC

TCGTATTCAAATCCTTAAAAGTGCAATGAGAACGAAATAAGTTAGTTTGACAATTTATATCCTTTGTTAA

TTTAGTAATGGATATTAGACTACATGATAAATTAGGAAGATGTAAGATATCTTTTAAGGTCAACAAATGT

GATAGAATAACGGATCCTTTCTCTACAATAGCTGAAAGAGAGCCATCTAGAATTTTAATTTTGTAGTTAC

ATGCTCAAGTACTATAAGAAGAAAACATACTTGACTCCCCAGTCATATGATTAGTAGCATCAGAATCGAT

AATCCAAGTATGACTTGGATTGACACTGAGTAGAGCAATATTTGAAAAATTACCTCTATGAGCTATAGAG

CAAGAAGGAGTTTGAGATTTAACCTTTAGGGCATTAGAAGTTTCCTCATGACCAAAAAAATTGGAAAAAT

CATTGTTGGAGAAATCACTCCAGCCGCTA

>Ca_linc_0459

GCATAATTGTATTGTTGGATACATGGTAAAACACCCAATGCATAACTTTCTATAATACAATCCTTGGCAC

TTCAAGTTCATGGTGAACTTTCTATACCCAAGGTCAGCACATAACTTTTTGCCTAGAGAGAGAACTACGA

TCTATGTTACTCACTTTCTTGCATAAATAAGTTTTCCAAATGACTTGGTGGAGTAACTCTTTTCCTAACC

AACATTTCTA

>Ca_linc_0460

GGATGTCAACATTCATTACAACGAAAACACAGACCTTTCACACGCTTATCTTGCAACTCGACATACGACA

AGTGCTTCCCCGGCGATGACGTTCTCCCAGATGACGAGCTGGAACGTCAATCTTGCTTGCTCGCGAGGAG

GGTGCACATAAGAAATTTTAGAGTGTTTCTCGAATGTGTGCACCTGTGAGCCGATGTCACGGCGAATGTT

GC

>Ca_linc_0461

TGTAGATGTCCAATTGGAGTCAAAATAAATGCAAATGAAAGCTAAGATTCATAGCTACAACTTTCATTAA

GACACTAGAACCAACTTCGAACTCAACAGATGAGCAGAATGCAGTAAATGTTATACAATAGATGTTGTGC

GCCCGAAGCGCAAATGATGTGTTTGGGGAGCATTTCTACATTCTGATTATGTCCAAGCATGCCTGGAGTG

CAAATTTTGCGATTGAGACACGTCACGTACTGCATAATGCATTCAAACATGTTTTTCTCACTTTCTAGGG

ATCTTATTGGCTATTGGGAAGTTTGGAGACTTGTGCTCTAACAGAGAAACTCATAGATAGTTTATTCTAA

CACCATTGAAGGAGTTTTATCAAGAATTGTTTATGCATCTTTGTTATAATTCTTCTTTAATCTCTAACTT

TTATATATCTTCCGTGAGTAACTAAACCCTATTTGTTAGGGATGAGTG

>Ca_linc_0462

GAAACACACAAATGAGTAGTGCTTCTTGAAGGTTTCGACGAGAGGAATCCAAAAATGATGTCAGTTTTTC

AAGATGATGAACGTGTTGGCCGGATTTTGGCAACACAGCCGCGGCGAGGGTGAAAACCCATTTTTTTAAG

TACAACGATTGGCAATTTCAAGCATAGGTAATGGGAGAGGGTGTTTTGTGGAGTCCAAATATGGTCTCAG

TTTTCATTGGGGATAGTCGTGGTGGCCGGAATCTTGATCAAAAGGTGGAGGTGTGATTTTTGACAGAGAG

GAATATGGCGTTTAAGTTTGCGATTCAAGTTGGAAATGAAAGAGAAGATCACAACGATCACAAATGTGGT

CTCGGTTTTACACGAAACTTGGTATAATGGTTATAATTGAAGAGAGAAGGTCAAGAAGATATAAGAATGG

AGGCGGAGGCTAGTCTACGAAGAAGATGAGGATGCG

>Ca_linc_0463

TATACAAACTATTAAAACCCAAAATCCTTAAATTCTCTCTCACAAACTTTTACACACACAAATAATGGAG

GGTCTAATTCCTTTGGTTTACAAGACTATAATGCAATACAAGAGTGGAAAAGAAGGTGCTATAGGATCAT

GGATTTGTGAGTCACCTTCATATTCATACATAAGACTTCCTACCGGTGATTCAGGTCATTTTCAAATTCA

AAACTCTACTTCATTATCTACCAATCACGTTTCTTCTTCTTCGTCATCTTCTTCTTCCGCAACACAAATC

ATTGTATCTTCTGGTGTTCAATCTTCACATCAATGTGTTACACATCGTCGAATTGCGGCATGAATAACAT

AAACACAAAAAAGGGTCATTGCTATATTAGATGGACTTAGTTCAAGTTTTCTATATATAGTTCTTGCTTT

CAAATGTTGGGAAGCTTTTGGTTTTTATATTTTGAACTTTGAAGTTGTGAGCGTGTGGAAAAAAAAAGGA

AAAGTTCATCATGCTCTTGGTTAGTTTGTAAATTAATTAATTGTGATTATGTGTTGATGATGCTTAAGAA

AATGTTCCATTGTGTTTTATGGCCAAAAGGGTCACTTGATGTATGAGTTTTTAGGGTAATTGATATGTAC

GCGTTTCTTTGGCCAAAGAGGTCGTGAAGATAATTGGTTTGTAGAAAAAAAGGAATTAGTCTACAATTGT

CAATTGTTTACTTTATGTTCTTAATGGAAAGATAGATATCAAATTAGTATAAACATTAAACCATTTATCT

TTTTCACCAATATTTACATGATTGATCGAGTGGTAAGAAACTCACATCTTTTGACAATATTTAGAACTTT

G

>Ca_linc_0464

AGGATGTAGAGAGATCCAAAAATTTACCATCTACCTTCATCATAAACAAACACAAATCTATTTTAACAAC

ACAACCAACAAAAATCAAATAAAAAAAAAACTGAAGATGAGAGAAGAGAATCACAATTAATTTCTTCATC

TTCGACGTTCGCGGTTCACTACAACCTCCTCTTTACTGCAACTTTTATTGTACCGGGAAACCTTTATTGT

ACCGGGAAAGACATGTACTTTATCCCTCTCTTCTTTTTTGCTTTGCTTTTCAAACTTTATCTTATTGAAA

G

>Ca_linc_0465

CGGATCCATTTTGCCGACTTCCCTTGCCTACATTGTTCCATCGACCAGAGGATGTTCACTTTGGAGACCT

GATGCGGTTATGAGTACGACCGGGAATGGAAGGAACTCTGTCCTCTGGATTTTCAAGGGCCGCCAGGGGC

GCACCGAACACCACGCGACGTGCGGTCCTCTTTCAGCCGCTGGACCATACTTCGGCTGAGCCGTTTCTAG

GGTGGGCAGGCTGTTAAACATAAAAGATAACTCTTTC

>Ca_linc_0466

AGGTGGGTTGTTACACACTCCTTAGCGGATTTCGACTTCCATGACCACCGTCCTGCTGTCTTAATCAACC

AACTCCCTTTGTGGGGTCTAGGTTAGCGCGCAGTTGGGCACCGTAACCCAGCTTCCGGTTCATCCAGCAT

CGCCAGTTCTGCTTACAAAAAATTGCCCACTTGGAGCTCTCGATTCTATGGCATGGCTCAACAAAGCAAC

CACACCGTCCTACCTATTTAAAGTTTGAGAATAGGTCGAGGGCATTGCGCCCCAGATGCCTCTAATCATT

GGCTTTACCCGATCGAACTCACCCTCAGGCTCCAGCTATCTTGAGGGAAACTTCGGAGGGAACCAGCTAC

TAGACGGTTCGATTAGTCTTTCGCCCATATACCAAAG

>Ca_linc_0467

TGGTTCTAGTTTGCCTCTGGTTCTGGCTGCCTTTGGTTTTGGTTCCTGGTTCCCTCTTGTTCCCTGTTGC

CTCTAGTTCTGGTTCCATCTGGTTCCATGTTGCCTCTGGTTCTGATTGCCTCTGATTCTGATTGTCTCAT

GTTCCTAGTTCTCCCTGGTTCTGATTCCTTGTTGCCTCTGATTCCCTCTAGTTCTGGTTTCTGGTT

>Ca_linc_0468

GCAGCTGAAGGATTGGGAGTATAGAGCAGTATAATTAAGAAATTTTGAATGGCATTGATTTGGAGTCTAG

AGCAATCAGGTGTCACATTTACAATTCACATATGACACAATAGTAATTGGAGAGTAGAATTGGTGAATAT

GCAAGCATTAAAATCTATATTACATTTGTTTGAATTGACACCGAGTTTGAAGGTATAATTTTGTAAAAGT

AGTTCTATGTGGATAAATGTGTCTAGAACTTAGTTGAAAGAATCATCATTGTTACTAAAGTGTAAAGTTG

GAGAAATTCCTTTTATTTATTTATGTCTTTTAACTGGGGGAAATCCTTGAACTAAGA

>Ca_linc_0469

CAACAATCTCATCTTTAAACAAATAATAAAAAAACATCATTTTTAAACAGCAAGCAAACAACAAATGAAG

AACTATATTAAAGAGATAGAGAACAAGACAAACGCGATAGAGGCGGATCTGTGTATGGTAGCGAAGAAGA

AAGTGGCGGCGGTGCACATCTAATGGTTGACGATGATTACGGGTGGTAACTAAAAAAATGATATGATCTG

TTTCTAATTTCCTATTTTTCTCTTTTAATCTTACAATATATGGTTTTGAGGATGAAGTTGGGTTGTTGTT

TTTTCTCTACTCAAATTCATTCTTGTTCCTGAATATATTGTTTGCTTAGTTTTTTAATCAAGAAAAAATC

TCTTTTTTTTTT

>Ca_linc_0470

CAAAATCTGAAATAGGAAAATATTTCCTTTGATCATAATATTGTTAATTTTGCCTTTGGTACTATTTCTG

GAAGATGTCAAAGTTCAGAAAAAGACTTGAACAAGTTCTAGAAAAGTTCCAGTCGGCTACCTACCAAATT

GTATCGAATGGAAATATTGGCTAAGTATTTTCTTAATTGGTATAAATGGTTTGCAAAAGGTTCATAATTG

ACAACAACAACAAAAAATTTGAAATCCACAGTAATTGT

>Ca_linc_0471

TTGGACAATAACGTCGATCATATCAAAAATCGACCTATGGAGGTAAGTTCCTTTCACACAAGCTCAATTA

CCAAGACATCGATCCAACCAATCACGGGAATTCAACCTCAAAATATAACTACATTCTATGGACTGGATCG

TGTCAACCTCAAAATATACCGGATGAGTGATGATCAATTGCAGAGATGAGTACAAAACCAAGTTCAATGC

CTTGGGTACTTTGTTGCACCGAAGGAACAGAACCAGACCCTGCAGCAAGGGTGGATTGCACTGTAGAGCC

ATTAGGGTTTTCCTGGTCAACAAAAATTACTCTTTAACAAATCCATATCAATTCAATTAAATTTGCAAGA

ACAACACATTCATTCCACCACATGCTTTCAAATTTAGGAACACTCTCGTTTTTCATTTTCTACTCTTTAA

GCCACCATAAAACCATAAAATAAAACCCTACACACCAGTGTTT

>Ca_linc_0472

TCTGCTACCCGAAAGAAACCCAAAAATGGATACTTGAAGATTAAATGTCTGCTTTAGGAATAACACATGT

GCTTTCATCTTGGCACATTCCTCTTGAACTCAAGTCCATCTCTCTACGGTGAAAGGAGAAGCTCGTGTTA

TTATATTATCTCTCCAATTGGCCCATAGCTAGCTTGGGTTTGCATAATGTCCAATTTGAGTCAGATTGTA

AAATTGTAATAGATGCACTTTTATTATATCATCGTACAGTAGTCTCTAATCTTGGACCTGTTTTGAATAA

ATGTATTTGTTTCTTACAAGCATACCCCAACTACAATGTAGCTTTTTTCCGTAGGGAAGCTAACAAAGTG

GCTCATAATCTTGCTAAAGCTTCCATATTACACACTAGCCCTCATAATGTG

>Ca_linc_0473

CGTACTACTGTGTCCTTGTATTCTAAGGAGACATGAACCTAGATCTGTTTCCTTGTATTCTAAGGATACA

GGAACCTAGATCTGGTGGATGAATCATGACCGTGCGATTAGAGCGTGATTAACGGTTCTGTGATGGAAGC

TTCAGTGAGCATTAGTGAGCATTAGTGAGCATCAGTGAGCATTGTAGTTAGTAGTAGAGGATTGTTCTCT

GACAAATTTCAATCTTTTT

>Ca_linc_0474

CCTTAGTGTTGTAGGCGCCCTGTATATACTATTGCCAAAGAAGTTGCTTCATCAGTGCGATATTGCTTCC

ATCCTAGTGCAATGTCTAGCATTGGAAATCCATTTTTCATCTTTAACTACAAAAATAAATAAATTAACAA

AACAGAAGAAAATAATTACGAAAGAAAAATTAAAAAAAAAAATAAAATAGAAGAAAATAATTACCGGAGC

CCAGTGATTTCCATTAACAAAACCAATACAACAAATACGATCATTTTTTGTCGATCCTGAATGTGAACCC

CTCATAAGAAAGAAAATAATTGAAAATGTATGACCAAACGTACCATTTATCCATGGGTTGAAGACCTAAG

CTGGGATATCTTCAACGAACCACTTACTTCTTTAATGAGTCCTGTAAACAGTCTATCATATGGAGTTACA

TGAGAAGCAATCTTCTGATCCAAACTATTGCGGACCAAATACCAACATTCTTCAATGTGTCCAAGCAATA

ATGGAATAATGCGAAAACCACAATTTTCATCTGCTGCCACATCAGATATGTCATCAATGTATGACTGAAT

AACATCAGGAAATTGTCTCCATAACAAAAGTTTTGAAGATTGAGATGACTGAGAGGGTTATTTCTTTGAT

TGAGATACTTGTCACTTCCTTGATGACTGAGAGGGCTGGAAACCAAG

>Ca_linc_0475

CCGACTTCTTACCCTTACTCTTCTTCACTCGTTCTTTGGTTTTGACTTTTTCAAGTGGTGGACACATTGA

ACTTGTACTTCGAAAAGCAAGTTCACGCACTTTACCCTTCAACACTCGTTTCCATATAACATCAAGTGAT

CAAAAGCGTTTCCATAAGGCATTCATCTCAGTAGACATATCCAACTCTGATCCATCTTCTGTGTCTTGGG

TGAGTTCACACTCCATGCTTAATTTTCTCCATTGAATATGCACAGAATCTAGAGGAATTGGTTCACCCAT

CAATTTATAGTGTCCCAATTCACAAGCACAAGGTAAACCGTGTGTCCTTCTGAGAGAGCACCCACATACA

AATTTGTCAGTACCCATCCACTCAACTTTCTCGTATTTTTCAGCAATACGTCTTTGAGCTTCTCTTGATA

CAAATGCATTCAATCTTTGATAAAATAGATTTATGTGCTCATGCTCTTCATCATAAAAACTTTTTTGAAA

TGAGACCCTGATTTTTTCTCTAAAAACATATCAATAGTTACATAATTTGAATCATAGACAAACTACTTAA

AATCTTACACTTTATTAATTAAACTAACATATACTCCACAAGTAAATTTATAACATAATTAATAAAATTA

AATATATTTTTAATCCATCTATTTTTTAGAAATTAAATTTTAAC

>Ca_linc_0476

CCTTTTGGTGCGCTCGTTCACTAAGAACTCGTCTCTTCATTTCGCTTATGCGTTTCAGTCTCGTCATCAT

TTCTTAGTCTCTAACCTGTTCATTTCGCTTTTGCGTTTCTTCTTCTCTGATTAATTGAGTCGTTATTCCT

CTGTCTCCGATCAGTATCGTCTTTTCGTTGCCGATTGATCTCGTCGTCTTTTCGTCTTCGATCGATCTTC

ATTATCTCATATCCTCTCTCTCTCTCTCTCTTCAAAGGTGACCATATTTTCGATCTTCTATTTCAATTTC

TAACTCTAATAGTTGAATTTCTATTACTGGATTTCATTTTCAATCTCTCCTCTTATTATCATCTCAGTTT

CCGCTTCCATTTGGAATCTCAGTTTCTATGCAATTATCTTGAATGAGTTCCATTTGGTATGAGCAAGCTT

ATACTTATCTTAATTATAATTCTATTTAAATGCCTGCATAAATTGGATTACGAAAATGCAATTGATATTT

TTCAATTTATTGAAATTGCAATTATCTATTACCATGTATTTGATTCAGAATTATTGGAATAAATTTAATT

TAGGTTAGGTCAATTGATATGTGTATTTTCATATAATGGTGAATGTCTGTTAGGGTGTTGGTGGTTATAT

ATCTAACATATAACATGTTTGAAGTACTTAAAGTTGGTTCCTCAATTTTGAATTTGAAGCAGCAATTGAA

TAATTGCTTTTGTTTTTCTTCTTAATCAATTGCTTTTGTTTTTTGATTTGTGAATCCTTTTTAAATCAAG

CCACAATGGATGTGTTCGCTTTGGTCTTCAATTCCCGATCCTCTTTGAGAATTTTTAGAAGAGGGATGAA

TCTACATAAGCCTTTTATTTGTTCGTCGTTCAGTGATTCGATTTTCAATCTTACTCATTATTGATTTATT

TAGCTATTATAATTAATCGGTGGGATGTGTCAATTGCATTACAAAGTACATTTATTGATTATTTTGATCA

TTTTGTTGACTGTGTATATATGGAACAAAGGATGTAGAGTGTGCACAAAAAATTTGTTACACCATTGATG

TTCTCTAGTGTGTATAAGAGGTAAGAAAGGGGGGATAGAACACTGTAACGTGGTACAACACCCAGAGAGA

GTGAGAGAGATTTGAATATTGGTTGGAAATCAAAGATAAATGCTATCATTAGCCAGAAGTATGTCATATT

ATGCCTGCATTGCTATCTTCCATTGTGAATCAACTAAAGCTTGTTTAGAACTTAGAAGTAGTAGGTTCAG

CTTTTTCTAGGAAAATGATGGAATTTGTTTGAAACTCCAGACTTGGATCTGGTTTGCGTAGGACGAGAAG

GAACACGAGATAATGATGACTTTAAGGGTGATAACTTGGTATCTCTAGGTGAAGATGATTTTCGTGTTAA

TCTCTATATTACTTTATCTAAACTTTGTTAAATGTAATTTATGATAACTTTATGTGTTGATTTGGATAGG

GATACTCTTTTTATTATCATGATTATTTGGTTCGGTAAGAGCCAACTATTATAAATTGATTATT

>Ca_linc_0477

CTCCAGGTCTAAGAATTATCAAGTCAGCTTCAAGAACAATCAATCTCCACTGATTAAAAGAAGATATTGC

TTCTGAAATTAACACTAATATTATCAGTACTATCCAAGGAACAAACATAATCCATTCCAGTCAAATAAAC

GATCCGAATCACAAAAGAAAAAAGATCATGAATCAAGCAAGATTAAACAAGACTGATCTTTCTCCAGATT

GACTGCCAAGAACGTTGAACGATCTTGAGATATGTAAGACATTAGAGCACCCTCAAGAACGATAAGGCAA

ACGTCCAGAATGATCAAGATTAAATCTCCAGATCAATTATCAATCTCCAGAATGATCGTTGAATCTCCAT

AATGATCAAACATTCTCCAGAATGTTCAACAATCGTTCTGCAACAGATTACC

>Ca_linc_0478

ATTAAATGCCCTAAAAGCCTATAAAAAGGAAGACCAAAGCTCAAGAAAAATGTAGAAGTTCAATGCTAAC

AAGTCTCATTAAATCATTCACATACTTGAAATTCTCACTTTCTCAAGAAGAATCAATTTTGATTATATTT

CAATATATTTTTTTTTTTATATTTGCACTGAATCTTTTATAAAGAATCTAATCTCAAACAAGAGTTCAAA

CATATCAGATTCTATCAAAACAATCATATCATCATTGTGTAAAACTCAAACTACAAGAGTTTAGTATAGT

TTGAGTAGATTGTAAGAAATCCTTTTAAGGTTAAAAGGTAAAGTGTAAAGTCCTATCTAGAGACTAATAG

AAGATTGTTTCGAAAGCCTTTCTGGGTGGAAAAGACTTGTTGTGTAACAGATCAAGGTTGATCTGGAGAA

GTGGTGTAAACCAAGATTGTTCTTGGTTTTGAACACTTAGTGAAAATCTCACAGTTGTGAGGACTGGACG

TAGCCCGAGTTGAGTGAACCAGAATATATCATTGTGTGGTATCTCTT

>Ca_linc_0479

ATTGATCTGGAGATTTAGTCTTGATCATTCTAGATGTTTTCCTGATCGTTCTTGAGGGTGTTCTGATGTC

TTACATATCTCAAGACCGTTCAACGTTCTTGGTAGTCAATCTGGAGATAGATCAGTCTTGTTTGATCTTG

CCTGATTCATGATCTTTTTTCTTTTGTGATTCGGATCGTTTATTTGACTGGAATGGATTATGTTTGTTCC

TTGGATAGTACTGGTAATATTAGTGTTAATTTCA

>Ca_linc_0480

NNGAAGAGACGCCAACGCTTTCTTTTCCGATTTGGACCTCTGATTTCTTTTGATGCATTACATATCATAA

ACAAACTCTTTCCAATTTCTTTTTTCTTCTCTCTATTATTGATTGTTCTTGTGTTAATTTGTTTGAGTTT

CTGTGCAAGAGTCTCTTCTATTTTGTTGGGTTGTGTTCGCTTTTTGTCAAAGTTATGTTGTGTGGGATGA

TGAGAAATGGATTATGGGTTTAGCGAATCTCGCTCTCTTTATTGGGTTATCTATGAAACGGATGCTTTGT

GTTGTGTGTTTGAGAAAGAATCGGGGCGTGGGAGGAGTTTACGGTAATGATGATTGGAGAGCACGCACAC

ACATACATAAAAACAAAAATGG

>Ca_linc_0481

TTTTCTTATTCTTCAGTTCTACTACAACCATAAATATGAACTCAAGTATGGCATCCTTGTTGTCCTCTTC

AAAGATTGGAGGATAGAGGTCTACAACAGGAACTAGAGAATCTCTAGTCCCGCTTATCTAACTAATGTGC

TTCTTTCCTTTTCCTTCAACATGATGTTTAATGCGATCCTTTACATTGACAATCCTCATTTTCATTACAT

TATTTATATTAAATAATGGTGTATACTAAAAGTGTAGGAGCTCATCCCTTTTTGAACCACCAACCTCTTC

TAGAGTTTTGATTGGTGGAGTTTTTTTTTTCTTCAAAAAAGCATATATCATCCTTGCATAAGGAACAATC

TTTATTTTCCTCGTTCTCGAGTTGGTTTCACAACTCTTCATGTGTTCTAGAATGAGATTGGGTATATTCG

TCTTCTTCTATTGAATAATCTTCCACATTGCGCTCTTTGCTCACACGGTCTATAGAGCCACCCTTTGACA

CAAAGCATACAATTGACCAAACACTACATTTAGGTTTCAAATGAACAAACTTTCCATTCACTATATAATC

TTCAAATAAATATTTTTCGATTTTTTTCAAACCCATTGTTCGTTCATATCCACTCCAGAGTTCAATTCCT

TTATCCAAACATCTTGTTACAGCGACAATTGAGGGAAAAATGATTTTCATATGTGTTCCCAATATTGTGC

TTGTTACTTCATCACCTTAATAG

>Ca_linc_0482

CAGTTTTAAATATTTTGTAATAAAAATAGTTCTTGCATAGTCATCAACAGCAATAGTCCTATTAAGAAAC

TTCATATTAATTGGTTTAATTTTGACAAACTCAATGAAAACGTCAAAATTATTACACACATGATAAGTTG

CTCCATAATCTATAATTCAAACCACATCTTTGTGATAATTATGGATAGCTTTTAAAGAAGATTTACATGT

GGACTTGTTAGTTGGGGTTAAACCAGCCTGGGAGAAGGAGTTTATGTGATTGGGTGCATGATTAGTCCCC

AAACTAGCATGTTGTATCAGATTCATAATTCCTTTGATTTGATCTAGAGTGAATGGACCTTGTTGGTTGC

CACAATCATCATCTTCCTTGTTATTGGTGTTTTCTTGTTCACCATACACATTATTGATAGAAAAGCCTTT

CTTGAAATTAGGTGGAAACCCATGCTTTTTGTAACAAAGTCCATAGTATGACCAAGCTTACCACAGTGAG

TGCAAATCTTGGAATTTCTCTTTCCTTTCCAAGAACCTTTCTTTGTTTCAGCAACATTAACAAAAAAATT

GAGATTATGTGTTTTCATTTGATCCTCTATTTTGTCTTTCATGTTGGAGAACTAAAGAAAGAACTCTATG

GAGGTTTGTTAGGGGCTCTAAAAGCAAAACTTGAGTTTTAACCATATAAAAACTTTCATTTGAACCAAGA

TTGGCATTGTTATTGTTGTTATTTGATCTTGCCATGGTTGAAAATGATGAAGGTCCTCGATGATACCATA

TTATGAAACAAGATGAAACTACTTTACTAAATAATTTCACTTATATTGAAAAATAAAATCAATCAAATTA

CAATGTAGCATCTCAATCTCTTTATATACAATAGGAGAAAAGATATAGAATGATGTGTAACTACCAAAGC

TGAAATAACAGACATAACAAACTAATAACAACCTAGGACAAATATCATATATGCTATTAGAAGCATGATC

ACAGTTAGCTTTACGAATAAGTAACTATCAAGCTAGATGGGAGATTAGTTGGCTAGCTAGCAAGGTGATA

GACATATACTCGTGAACTCATGAGCAAGCTAG

>Ca_linc_0483

TTTTTTTTGATAAAATAGAGCTGGTGTCATTTGGTTCAAGCAGTTTCTCTAATTTTGGAATTAAGGTGGC

ATCAACAAGTTTGTGTTATGTTTGTTGCTTGATTTTTGTTTCTTGGAAAGTACCAATGGTTCAATATGAC

TTCTCTTATAAAATGGCATTCCCTTGATTATAAAGGGATACAAAGCCTAATCATTCCTATTAAGAAACTA

TATCATGTATGCTACTCAATCAATATAATTCAAACCACAAGTAGTGTATATTTTTGAATTATTTATTTTA

ATTTAGACATAGTCTGGTTTTGTTTGTTTCTGAGCATTGAAATGAATCGGATGGATATAGATAATATGAA

ATATAATGTCTTGATATTATTGGTCCTCTTACACTCCAAAGAAAGCGAGCAAGGATCGCTGATAAGAAGA

AGAGAATTGCTAAGGCAAAATCAGAGGCAGCTGAGTACCAGAAGCTACTTGCCTCTAGGTTGAAGGAGCA

GAGGGAGCGTCGTAGTGAGAGTGTGGCTAAGAGGAGATCAAAGTTATCCAGTGCTTCTAAACCAGCTGCC

ACTGCCTAGGCTGCTGTCTGATGTAGAGATTTGGTAGTTTAATCATCACAGTTTTTGGTAACGTATGATA

TTTTTTGATGATGGACTCATGATAAGCGCGAATGGATTTTGGTATTTTTGATACATTGAGTTATTGGAGT

ATTCTCATTTGTAGTGTCACTTGTTGAATTATTTTTAACTTTTTTAAAATATATGTATCCATTTCATGTG

TTACCTCGTTACCTGTAGGTATATCCTGCTTTTTATATCATATTTTATGGATTGAGAGTTTATGCATTGA

TAGTAATAGCTTGGTGACTTGTTTAATAAGATATATTAATTTTTTTTCCCTTGAAAATAGC

>Ca_linc_0484

CCCACAACAGCCTCAAACGTGGATAAGATAGAAAGATAAATATATTCCTTGTGTTATGCAGCAGCCAGCA

GCAACATTAAAAAAAACAAAAGCGTGGTCGTTGCATTTTCCTCCCTTTCTCGTGCTTCCATTTCAAACCC

CCCATAACGAACAACCACGAAATGCACCTCCCCATTATTACTATCTCTCTCCTCTCTTTCTAATTTCCGA

GCTACCATACGAAGATGTCCGTTGAAGCAACCAGAAGGGTTACCGTTTTATGTTCCCATCTCAACACGAC

CCGTTTAACTTCGGATCCACGCTN

>Ca_linc_0485

CCACCAACACAATAACCAATTTCGCCAGCATCTATATATTATATATTATAATATAATTAAGAAAGAAATA

GAGATCCGTACATACAAACATGTAGAAAAATAGTGTTTCTCGCTATACATTTCCACCCCCAAAAAGAAGA

AAAAAGAAAATTAATATGATGATGACGATAATTAAGAAGAGTAACAAAGTTCTCTTAATGAAAACAAGCA

ATATTAATTAATGATTGAAGATCTAAAATAAGAAATATGAAGATGACCATATATATAAACCGTTACATCT

CCTTTTCATCTTCAATATTAGC

>Ca_linc_0486

GCCAACTATTTCTTTTCCATCACCAATACAACTTACAACAAAAATATTATACAAACCATTTAAGTTCCTA

AAATAAGTTAATTCAGATTTTTTTTTTTCAGATAAGAAATTAAAGTTGGGTCATTTTTCCTTAGTGAGCT

TCTTCAAAGCTCGAGCTGTACGTGGGATCCCAAGCCCTCCGACAATTCCATGCAGTGCAACTCATGGTGA

TTGGCCGACCTTTTGAGCATAGCCCGCCATTTTTCCTATGGATACATGAGATCTTTTGACAAAGTCAATT

TTCTCCCATCAGTTTTTCCCTATTTTTTTGTTCGCTTTTAAAGTTGTTGAGTAATATTCTTCGTTTTTCC

TTCCGCTTTTGGGATAGGCTTTCTTTTCTAAGTGTGCAATTTAGCTTCGTTCTTCTATTGCTATTTATAG

GTTTGAGCATGAATGTCATCATCCCTTTTATCAATTGTGGCAGCCATGTTCCTCTTATTTGGTTCAACCA

AGTTATCAACTCCCATATCTGGGATTTTGTATTTAGTGACTACCTTTTCTGTCTTTGGAATTTGAGTCAC

TTCTTTTGTGACCACCTTCTTAGTTGTCGTGTCCTCCCCACTACCAACCCTAATCTTCTGACCTTCATAA

ACAGACAATCAGC

>Ca_linc_0487

TGTTTTTGCACTTTTTATCCAAGCATGCATTTACCTTCAAAAAACTTTATAGGGTTTCTATAGTATAATT

TAAATATTAGTAGTAAAGGTTGATTTAGACTCTTATTATTTGGAATAACCATGTTTTGAAGGTTATTTAT

ATTTGGGTACTGGTTTGGTTAATTCTATTTCTGATTGAGGATACAAACAGTGTCCTGTATGCTATACTAA

TTCAAGCACAAGTAGTAAACTTTCACATTCCTAAAGATAAAAGAATTATAGAAACCAATCTGTTTTGCTT

TTGCAGGATATGAACAGAGGAGAGGAGATTGCAGGTTTCTCTATCACATGT

>Ca_linc_0488

TACATACCCTCCTCTACAATATAATTGCATTTTGATCTCTGAAATTGACAATGATGATTTTAAACTCATT

TTTGAAATTGAAAATCATCAATAACTTAGATTATGTGATAGAGGATCGAATGCTGCGGCAAGGAACCGAT

TATGTATTTATTATGGTTTGTGATTATTCAGGTGTTTGGAAGAGAGTCTCTGGCTCTGCGCTCTGCACTA

AATAGGAAAACTGCTGTTGAGGGATCAAACTTCAAAATTGGTACTCTAGGTTGGAATACTCTGTGTTTTC

CTCTCTACTTCCATGATCTCAATGTTCCTTTATTCTGTCTATAATAGCTTTTGATTAGTAATCTATGAAG

AAACAAGTAGTATAGTTTTTCAATGCTATTTTTCCTTGCCTTGTTCTTGCTCTCAATGCAGACTACATCA

GTAAGGGAAGAACACAACAAATAAGGATATGATTCTTGCAGGCTGCATCTTTTGGTGAATTTTCTCTTTG

AAAATTCACTAACCAAACCTGCTGACTCATCTATGAACAATGTGATGTGCATTTATTTTTTACTATCTAG

TTTATGCCTTGCTTGTAGATTCTAAAGTCATTTTAGCTACACATTCACGGGTTGTAGATGATGAAGTTAC

TAAGAATAATAAAATATTCTTGAAGAGTGTGGAAAGTTAAAGATTCATGAACCATCTTTATTACTTACAC

TCCAAGAAAGCCAACAAATTTCGACACAATGCTATAAGTTAAAACACCATTCAAAAGTAGAATGTGGTCC

ACAGCCTATGAAGAATAACTATCTAGTAACATGTACATAACAGTTGGTTAAAGGGCATGTCCACT

>Ca_linc_0489

AGACAGTGTTGTTTCTCGATTCTGACATGCTAGGGAGAGCCAACATGGGTCTGTATGCATATAGTGTCAG

GCCAAAGCTTTCGCTTAACGACAACGGATTAGAGATAAAAGCTCTTGCTCACAAGCACATGTTGACAATA

AAGATATACAAGACAAGATGTTTGCACCGTCGTCGCTGCCACTATCACCACCAACACCATTTCAGGGACC

ACCACCACATATTCTGGGGTTTCTGACGGGCTCCATTGATATATGTTTGCTACCATATTTTGAGGACCAT

GTGGCACCCACATATGAGTTGAAAATATAAGT

>Ca_linc_0490

CAAATTTCAAAAGGCACAAGTAGCAAGAAGCAGATTTCACACATCAGAAATTACTCACAAACAAAACACT

AACCACACATCAAGCATCAAGATGGTCTCAACCAAAACTCAATCACATATAACACAACAAAATTTCACTC

TTCAATAATATACCGCTTTAAACAGGACAACAAACATAAAAGAATGAGAAAATATCTTCTTCCGATGCGC

GGTGGTGTGCGGATGTACTAGGTTCTCTCGTCCGGTTGTTGTTACAATGGACTTTAAGGTTATGGGTTGA

TGGGAAAGAATATGATGATAGATCTTTCTTCTGTTTGTTATTCTTTGTGGTAATACTTTATTTCTATTGT

TTTTTTTTTTAGAAATAGAAAAAGCAAGAAATGAAAATCTTCCAATTTTTATTTTGTGATTTCTTTTCTG

ATCAATCCCCCTCTTATTGAATTTTTTGTTTCAATATATAGGCAAAAACGAAGAATGATTGTGTTCACTG

TATAAACTTAAGTTCTTGATCAAACAAACTCATACAATTAAGGAAAAAGGGGGTTGGTCTTTTTGGTTGA

GTATATCTATTGAGTTCAATTCTAACCATTGAACATAAAACTGAAACTTAGAATTTTTCTAAGTGTTTTT

GGAATTCAATTTTAGATGAAATAGAATGCATTAGATCCAGAAGTGTGAACACTAGATGAATGGTTTCTTG

ACTAGAAACAAGAACATCATAATCTTGTTTTCTTGATTAAAAGCAAGAGAAAAACGTTTACTGGACCAGA

TGCAGGAACACCATATACCTGATTATTGGACTAGAGGCAATAGAAATCAGAGGCGCGATTGCTGGACCAA

AGGCACAATTATTAGAACAAAAGGCAAGAGCAATTAGAGCATGATTTATTGATCAGATGCAGGAACACCA

GAGACATGATTTTTTGACCACATGCAATGACCAAAAGCAATATTAATTGACCAGAGGCAGAATTACTATA

CCAGAAGCAATAACCAAAGCCAGGTTTACTAGACCAGAGGCAACAACAAGAAACAAAGGTAAATGACCAA

AGGCAGACTATCAGCGACACTGACTATGTTAGAGTTAAATTTAACCAGAGGCGTTGACTTTTTCCAATGT

AGACTTTTTTGCCATATGTGTTTATGTTTTCCAAAAGTTGACTTTTTATTGGATGTTTACTTTTTCCTAG

ACGTTGACTTTAACCATAACATTGACTTTTTTGGAAGTTGGCTTTTGTCATAATATTGACTTTCACCAGA

GACATCTTATCAGAGGCAAGAAGTTCAAATGAAATCTCTAGAGACAAATTATCAGAGGCAACTGGTCAGA

GGTAGTTTTGACCAAAAGCTTGATTGCTTCACCAAAGGCAAACTATCCCAAAGTCTGTTTTGCTTCATCA

AAGGCAGAATTTCCCAAAGATTCTTCACCAGAGGCAGCCTTTCCCAAAGTATGTTTTACTGCATCAAAGG

TAAACTTCAACAAATGCACTGAGAGCATAATTGAATCAGAATCAGATGTAGTCTTGGCAAACCTTCTTGT

ACCACCAAATGCAGAGGTAGTCTAATGTTTTGTATCATCAGATACAGTGTTTCAGATGCGCTTTTTCATC

AGTTTTGAATGCAAAATTTTCATGACTTTTTGACTAAATCATCAGAGGATATATCCTAATATCCTACAAC

GTTGCTTGTGGTCTAGGAACTGCACACTTAAATAAAATATTAGTGCCTAAAATTATTCCTACTAACTAAT

TTTGTTATCATCAAAACTAGTAGGATCTTTTGTACTTGAAACCAATAGTGTTCTCACACCAGTGGCTATT

GATTCGTGGTCGCTCATAAGTCAAATTTGAGTAATAAAAATGTAACTTAGGGAAACAACTTGAGTCGTAT

CGCAAAGATTTCTG

>Ca_linc_0491

GGATGAGGCGTAACAATCTTCAAGTATTCCCCAAAGCTCAAATTAATTGCATAGAAAGAAGGTTTTGATT

TTATAGTCATAGTTGAATTATGTAAACATGGGAGTGGTTAACACGTGTTTGCCAACTCCATGATTATGTG

TTGATTATTTCATTAACTGTGTACATACAGGGTGATTTAGCTTCTATCTTGCATTGCATTATTTCTCTCC

TAAGCTCCCCTCTTAGAAGGTTTTCTTGTGTGTTGTGTTTCAAC

>Ca_linc_0492

AATTTCTAAAGGGTAACCAATATCAGCATGACAAGAACACACAATGCAGCCACAATTCCAGCATGCCACC

AGCAGACAGCCCTCCTGAATGATTTTCAAAGTCTACAACAAAGTTGAAATGCTTATAGAAAATGACAAAG

CACGGCAGTGGAAAGAAACATGGAAAACAAATGATAAACAAAAACTTACTTGGTGTTACTGTGATAGTAG

ATATAAGAGGTCCATATACA

>Ca_linc_0493

GGGAAACTCGAGCAATACTCATGCTGAATTGATATATTTGTTTGTGGGCATGGTTTGAGTCATGCTAGGA

GGATGAATTTCTCACATGTAGTATGCAACTCAAACTGCATGGCTACATTCAATCACATTTTTTGGACCTT

TAATTATCTGGATCTTTTGCCGCTAAAATAATCTATTTGAAGATATGCTGAAGTTTGATTGGAATGTCCA

ATTTGTACATACATTACGACAGGATCAGGTGCGGAGCATCTCAGAGATAGGGGGCCGCAACCACCCCAAG

AAAAAAAAAAATAATTTATATGTAAAATGACTAAAATGCCCATATTAAAACTAAACAATTACAACAATAC

CTAAACCTAATTCAATTTTCTCTCTCTTCCATCCAGCCTCTCTCCTTCTCTGTTCTCTCCCTCTTGCTTA

CAAGTTACAGCCTTCCTCTCTCCCTCTTCCATAATTAGATTCACGAATCAATCACAACAAACGCACGGCC

GCCTCTGCAACTCTGCCAAATCACGAATCGCTCTTCTGCTCGATGCAGGCATGTGGTGGCCGCCTCAGCC

TAACTGCCTCCGTGGCCGTTCTTCGCTCTCGGTCTCGCCTCTTTGTTGGTCGCAACTCTCCAGATCGCGT

TGCTGCTTTGTTCAAAAG

>Ca_linc_0494

GATGAATAACACAAGAAATGGACATTGATTCATACTAGTTCAAACAAGTGGTACCAACACTCCGATTAGG

GCCTGATACTGAATCCACATATTTCACTAAAAGTTGTATCAAGAAGTGCAAGATTGAGAAAAACATGAGA

GTGAGAAGTTTCAATGGTTGAAGAGGAAAAAAACCTTCCATGGAGAGGACGAATGTTGCTCCACTCCATT

AGGGTCATCTGCTTGAGAAGGTTGCTGTAATGCAGAAAGTGACATCTTTACCGAGAGTTGAAGTAATTCC

CCCAGCCCCATTTTATCTTTTTAGTACCGATGGTCTTGTAGTGCATCCTTCATCGAAAGAGATTCACCTA

ACCAACAATCCCAAAATATGATGGTGATTATGATAATTGGTCGATGCTAATGAAAAATCTACTTTATTCC

AAAGAATACTGGAGTGTTATTGGTGAAGGGGTTTCAGAGATCTCATAGACAACAACACCAACACATATCA

AGATTATAGAAGAACCAAATTTGAAAGACTTGAATACAGAAAACTATCTTTTCCAGACAATTAATAGAGG

AATTATTCAGACTATTCTCAATAAAGACATGACAAAGG

>Ca_linc_0495

GTCTAAATCTTGGTGAAAATAAAACCCAAGTAGGATGGAGGATTAGTGACTTACGAGGTTTCCTTGAACT

TTCCTCTGTCCCTATCGTTATTACGGAATATACTTATGTACTATAGTAGTGCTTGGTGTTGAAACAGCAA

CCATATATGTGAATGTGATATTGTGATACGTGGATATTGTATTCAAATGTTCCACGCCTACACAATGATT

GTTTAATTTCAAATAATATCCATGCCTACACCATGTTGATTGTTTAATTTCACATAACATGCGTG

>Ca_linc_0496

TTTTACCTTAGTATGCTTGTCAAAAGACCAAGATTAGAGAGACTAATTTATCCCCCTTGGCTTCATCATT

CTCAACTTTATCTTCATCGGAAACTAGCTAATTATAGGGAAGCTGGATTTTAGGCTGAATGTTTTTGATG

TTGCTTTCTCTTACATCCTAAATATGGCATGAATGATATTCTTACTATCTCCAATCTGTGAACTTTCTGC

GTTTTATGTCTTCCGTGCGTAGCATACTCAACCCCCATTCCTTCACAATCAAACCATGGCAGGATTTAAA

TGTGATTTGTGCTCTTGTTTTCACAAAATGGCAGTGTTCCCTAGAGATATAAATTGGGAGCCATCAACCT

TGTTTAGTTCCAAATTAAGTGGCATAGCATATCAAAGCGTTCTTCAGTCTCAAAAGAAAGATAAAAAGGA

CATGACAGTGGCAAGGAGAACAACTGATGTTGAGAATCAGACACATGAGAAATTTCAAAA

>Ca_linc_0497

NNCAGTAAGACCCCACCTTGTTCAATATCTGATCCATTTCCCTCTTCTTCTATTCTCTCAATTCGCTACT

CTTCTCTCTTCACAATAAACAATTTCCCAAATATTCTTACAATTGTATAAAATAAATTTCAATGCTTTCA

CTAGTCTTTTTTTTAAAAAAAAAAGAAATTAAAATGGACTAGTCAGCTTTAATTGTAGTTC

>Ca_linc_0498

CTTTTTAGATCCTACATCACTCAAATAGCATGATCCAAGTTTAACATAAACAAAAAGGTGTTCATTGTTA

TTCGACGAGACACTCTTCACTTCCAAACTGATGATTTCCACGTGGATTATTATAAACCTAACATCACTAT

TATACTTACTCTTTTCCAAATCTCCACAAAGAAACAATAAAATAAAAAATCAGCATTAAGACCCTCACGA

AGCAAAATACAGTTGAGCTCATTCATCATAAAAAATAATTTCAATGTTAGAAACTGAACAGAATTCCAAA

TCCAATGATCAAATT

>Ca_linc_0499

AAAAGACTCATTTGTTGGGCGCACTTTGAAGGAACTACTTACATAGTGACATAAACTTTTCATGGGTTAA

CTACTTGAAAAATCTTATTAAAAGTTGTCATTCAACTTTTTACATTAAATTTTAATTTATTCATTTAATA

ATTTTTGATCATTTTCTAGTACTTATAGTATCCTAGAGATCATGCCATACCATGATGATATTACCAATTA

AAAGGAAAACTTTGATCAAGACAAATGAAGAGAAGGAATTTTGACATTTTATATTGATATATGCTATTGG

AAGTTGAAAAGGTAATTAAAAAAAAATGAAA

>Ca_linc_0500

TCAATTCAACTAGTAAAGGTTTCATTTCAACTTCATTTATGTAATTAATGCAGCTATTTCCTCATACTCA

GTACATGATTTTAATGATATGCATAAATAGTCTTGGCTCTTAAACTAAAACAATCATATGTGTTAATTAT

CAAAATGTTCTTCCAAGCATGCAATTAATCATAAATAATTTCATAACTAAGCTTCATACTACTTAGAAAA

ATAATTGTACATAGAATTTCTAAATCAATTTCAACTTATTCATTCACATCATAAGTCATACGGTATGCAC

TAAATAAAAATTTTATTTTATGAAATAAAAAC

>Ca_linc_0501

GAAACATCAAACAAAGAACTAGAAAGCTCAAACTCAAACAGAGACACAACCAAGAAAGCAACTAATCAGA

TAAAAAAACTCTATAAATACATCTTTTTTTTTTACAAAGAAGGCAGAGATTTTGGAGGGGAAGAAACAGA

TCTAAAGAAGAGAGTTGAGGTTTAAAAAAAAAGAGAAGAAGGGAAAAGATAAATTTTTTTAAAGAGAGAT

TCTGAAACAAA

>Ca_linc_0502

CTTTCCTTATCTTTCTTCAATAGCATACCATGTAGTGCTATCATTCAACCTCCACAAACCAATATTGAAA

ATTCATATTCTTACAACAATCCTTGAGTTATGAAAATCAGGGAGCAATTCTTGCAACTCCTCTCTTAGAT

GATTTTAATTACTATCATTGAGTTATGTTATGCGGGGAGCTCTTTTGTCGAAGAATAAACTTAAACTTGT

TGATGAAACAATCTCCATTTCCCGGACATCCGATACCAATTTTGAAGCTTAGCAATGATGCAACGCCATA

GCCATATCTTGGATCACCCGCTCACTATCTCCCTCAATTTCTTATAACATAATTTACACCGAAAATGCAA

TTGTCTTTGGAATAAACTGGAAGACCGTTTCACCGGTAATCACATTTTGCATTTCAAAATCTCCTTCGCA

AAATCCATATCATTTTGTCAAAGGGAATGTTCTACCACTGCATATTACACTGATTTAAAAGGTTTATAGG

TCAATTTGGAGCAATTGCGTCCAAACCCTAGTTGTATATGCACCCTTTCTTGTGCTTGTTCGGTCAATAT

GTCAAAAAAAACTAAAAATGCTTCCAAATTCTGAACATATGATATTTTTCCCCAAGTGTCTTAAAACTCA

TTCTCCAACAAGAAAGTCAATTTGCTGCTTCAAATATCATCTTTGTTTATTCTTGACTTCTATTTCAATA

TCATCTTTGTTCACAAACTTTTTGCATCTCTACATTGTAATCTTACTTTACAAAAAAGCACATTGTTCTA

TTCAAGAATCTCATCGATCTAGAATGATTCGTTATGCTAAATTCATGTTGGACTTTATCGTTTAGCAAAT

TATATTCCTAGTATTTATAATTCTAAACAAGGCTATAATTATGCCATTCATCCTGTAAATGAGATTAATG

AAGTTTCTACTAATGTTTTTTTTACTTTATATGATATATGTGTACTTAATACTATCAATAATTGTACCTT

TA

>Ca_linc_0503

TTTTCCTTCCAACAATAGGATTGATTAATTTTTTCAACACCATGTAAAATCCTGATCCAAATCTACGACC

ACAACTATGACTAAGACGAGGCTCTTGCATGACATGAGTCACAAGAGGTATTGGAGCTATGGTAATGAGT

CGAGAATGTGACCTTTGCAACCTTGGTTCTGATAGGCTTCGCCCCATCATAGCTTTATTTAGCTGCGATG

CGTGTGCCTCAAACGAGAGCCTCTCAAACTCATCCAATAAAGTGCTCTTTTCATCTCCGAATCTTCTTCC

AATCATAGTAATTTCC

>Ca_linc_0504

CCTCATTTCAATTTTTTTATCCTTCCTGTTTTGTATTTTTCTGCTAAATTCCTGCTCCATACTATGTTCT

AAGTTCCATGACATTTTCTCAAGATTATTGTATATATCTTGTCGGATACCCTAACATTCATGTAATATGT

ATGTTGGATGGTTTGTATGATTAGTTTATCTGTAAGATATATATGATTGTCTTAAGTAAATCTCTATGGA

ACAAAAAATAATATCCATAGATTTAAACAAGTGTGATTCTTGAATTTCGTTGCTTCATATTTGAATGCTT

TCTTAATGAAATATCGGAAGTGTGCTAATGTATTTGAAAAACCATGTCATTTCATT

>Ca_linc_0505

CCAGAGTCTTCCTAATAACAATAGAGAACATCAATCAACAATAACAACCTTATAACAACCTTCCACTAAA

GTGTGTATGCCAAAATAACTCTTAGAGAAGATAAAACTACTCAAATTGTATATTAAAATAACAAACTTAG

TGGTAGACATAACATACTAAATTTGTAGATCAAACGAAGCAAGTTTGCTACACACATGTTACCACCACCG

GAACCAAACCCTTATTTTTCTTCTCTGGCAGTCGTTCTCTTTGTTCTTCTACATTCAGATGTAACATCCG

AGCCTAGGAAGGAAGAGGGAAGGCAAGGGTCCCAGGAGCTATCAGTATTAGCTGCTGACTGGTGACGAGA

GGATCATATAGTTTGAGAGTTGGATCGAGTTGGAAAGCGTGGTTATGTCGGAATTGAATTGAATGTGCCC

TATCCCGCGAAGGAGAGGAACCCGTTAAATCCCTTTTATTTCCTTCACATGGGCCTAGCCCAATAATACC

TTTTTTT

>Ca_linc_0506

CCAGAATGATCAAAACAGTAACTTCAGATTGATCATCAAATCTCCAAAAAGATCAAAATGACAAATCCAG

ATTGATAATCAATCTCCGTTTCCTGCATAACTCCAGCAGCAGTTTGTTTGCCTCTGCAAATGGCCTATGA

TCTTCCTCCAAGCTTCTTCAGGCTACATTCAAGACTCAAGATCGAAACTGTACTATCCAAGATTAAGGGA

GAAGCTTCAAAGGCTTTTTAACTAGAAGACGAAACTGATTCAAAAGCTGTAACAAAAT

>Ca_linc_0507

TTTTAGATAGCATTTGGTAATGGATATAGCAATGCTATATTCTACTTGTTTCCCACACAATAGTGCTTTC

TTATCAAACACTTGATCAATTTTAACAACGGCTTAGGCTAACATGTAGAAGAGACAGCTACGTGAGACTG

TGCAGCTATATGGAGCTGATGTTGTAATTAATATAAAAGGTTAATGGGTCGGTGTCACAGGTCATGACTG

CAGCGGTGGAGCCAGCTTTAGATACAGAGGATAAGGCTAGCTGCTGATTTTAGTTCTGAGTTAGCAATGA

ATCCATACCTGGTGCTGTGTTTTCTGTCGTGTGTTGCTCAGGTGGTTAGGCCAAATCTGGTCTCAGATTG

TATGGCCTGGAAAACTTCGCTGCATTGTGATGTCTTAAGCAACTGGAAGTTAATAATGTGATTGTAATTT

GTAGGGAATAGTTATGGGGTGTTTTTGTTTGTGGTTGGAGGGGTGTTATTAAGGTGTTTGGCTGCTGCTA

AGGATGCTGGTATGCGACATTCATGCTAATAATACATTTAAACCCTTAACAGCTAGAAATTATCAAATTC

AGAAATTGGGTTCAGAATATGCAGCCAATTACTACTTAGCATTACACCCACAACCACAAAGGTAATTGTA

ATTCCACTTGCTCATAAGTCTGTAACAAATTAGGCTGAAGTCATATTAATATTATCTTCAGCTTTTAAGC

AGTTGTCCTGTAAAAAAGAGGCTTTTAATTAGTTGTATGTATCTTTTTAAACACTGCGTATATACTCACA

TAATCAAATGAGAATTGCTCAAAGCATAGGCCCATTTCACAAGTACTGCCATTTTTGTTTTGTGCTTCAC

AAGACAAAAATCCATAATGATGAAAACCCTTTCCAAAATCCAGGATTTGACATCCAATTCAAATAAAAAA

CAGAGAAAATATCTGATCCTCAATCTACTTCCATGGCAATTACTAACTATCCAATTCAAATAAAAACTGA

GAAAATATCTGATCCTCAATCTACTTCCATGGCAATTGCTAACTATCAAAGTTAATTATAACCTATTAAA

CATTAATACAAATTAATCATTTTGGCAATTAAGATTTGAGGCATGCCATTTCATTAAACAAAACATTCCC

TTTGGTCTCAGCAACAAACGAACAGCAGCACAATCAAAAATCCAAACAGCAAACTCTACTACTCCTTCAA

AACCAACTAGCTAGATAACACAAGAAATATAACTGTCATAAGAGTTAAGATCGAGCCTACAACCAATTAA

AATGTGCACTAAGAAAAAAGTTAATTGTTGAATCCCCCAAATAGTGGTCACAACTCATACAGTATTTATC

TGCAAAATCACGTCAGGTTACAGTTGCAAAGGGACTAGAGTCATTAAAGTTCAAGATATCAAATTATCTA

TCCATGCTTTAAGACAGCAACAACTAGCTTTCACATTAAGGTAACATCTATCATTTTTGGGGCATAAAGA

CATTCAATCCCTTACAGAAATCATGCAACAAAGTATATCCAACTTGCAGTCGAATTTGGAGTAATTAAGT

TCATGTATACCACCCCATTTTTAGATAATGAAAGAAAAAAATACATTTCATTTCACTTATCCATTCACCA

>Ca_linc_0508

TTCAAATACTTTGCAAAGTAAATTCCAAATTTACCATAATTAACGAAAAAGAAGTTTTTCAAAAATTGAT

GCAAAACAGAGTTCGAACATTGCGGGACAAAAACGTTCAAGAAGGCTAGGGTTATGGGTCGTGTACTCCA

CAGCCTTTTTGAAAAAAGATTTCATCTCGTTTATTTTTCTTAAGCAGAAGATTGAATGTTCTTGAACAAT

CAAGAATGAGAAAATGGATATTCCAATTATAAACCAGACGGGACAGAATTTGTTGAAATGAAATTCCAAA

ATCGTGTAACAACGTAAAAAGTGTATTTCAAAACCTTGGTACCAAATAGGGTTTTCAGAGACAGAACATG

TTTCATCAATCAAAGCAGAAGATTGAAATCAA

>Ca_linc_0509

ATGACAATCAACAAAACCAGAATCACGATCGAGCAACAGCGAGAAACAACCCAGAAGCACAATCTCAAAA

CCGAAAAATTGTGAAAGACAATCAGGAATCACGAAGAAGAAACCAAGAGTCGAACAAGCAATTGCGACAG

CTATGGTTTAGAGTTCGCAACGTAATTACAAAACAAGCAAGGACCAAGAACAGAAGGAACGATTTCAAGT

GCGATCCAATGAGGTGTCACTGAATAAAGCCGAGATTAACTAAGAACTCACATGTTTAATTGAACTTGTT

>Ca_linc_0510

NNGCTCTTCATCGTTTTCTTTCCTCTTTCTTCAAATCCTCTTTCTTTCTCTTTCTCTCACTCGCTTTATT

GCTACTTGTTGTGTTGTGTGCTTTTTCTGTTTTTTTTGTTTTTATTATTTTTTGTTTGATGATAACTTCT

TTTTGAAAATAAAAATAAATAAAAAAATATATGTACTCTAATTCAATCAACCGCCTACTGAGGAAGTTAG

TTTAGGTTGATCTGCCAACAATGGAAATTTTTTGGATCTGG

>Ca_linc_0511

GTCTTGTTATTGTTCTTTATGTTGTTTTGCTTCTCTTGATCTTTTGGGCTTGAGCATGGCTCGTTAATGT

TTTGACTTGATCTTGTTATTGTCCTTGATGTTGTTTTGTCATCTTTAAAATGAACTATCCAAAATGATCT

TGGATTACCAGACTGTTGCTTTTTTGAACTATAATGTTATTTTATATACCATAATGATCTTGTGTTGTTG

TATATGATTGTCTATCTTGGAATAACACACTTAAGAGCACAAGTTAAATAGAAACACAATTAGAATCATA

ATTAGAAATTAATTAAATTGTTTGTTTATCTTCAAAACATTAATTTGAAGTTTTGCCTCAACACACCTTT

CTGCAGAAAGATTTGCATGCTTGAAAGCTATAAGACTGATAT

>Ca_linc_0512

AGGGAGTATAATTTTTTTGCTTATATATGAGACTAGACGGAGTACATAACAAACATAGTTATGCAGGGGA

GTTGGGTTGACCAGAAAAAGGAGTATGAGTTTATAACCTTGCATACATCTACAAGAAAAGTGCTACTTTG

GGAAGGAAGAAAAAAACTCAAAATTTCACAGCTCACAACCATGTGCAGGATCATTGGATTGCATGTGCAG

AAAATGATTGATAACAATAAATCCTAAAAAATGATCGACTTATTCCCTTTTGTGTAAATAGGCATGGAGA

TGAAGGTGGGAGGTAGTATTGCACAATTTCTAATCCCAAAATTAAAAATAAGTTCAATTAATTTGTCCCT

AAGGTGTCACTTGTGTAAGAG

>Ca_linc_0513

CCTACTTTCATTTACATATATAGTATAAAGCATACATAATATTTTTCTAATGAATGTGCACATTTTCACA

ACATAAAATTTATTTACAATTAGAATTCGATTGTATGATATGAAATAAAATTTTGATTTTACCAAATGAA

GTGTGTTACAGCAATTACTATGCATTTAATATAGAAGTATGAAGTGGACAAAATAAGATATATGGGAAGA

TGAAATTCTACTACTATATATATGATGTCCCAAATGTGAATATTCCAAATCAACTATGCCTATTTTTTAT

TATATCTATAATC

>Ca_linc_0514

CTCTATCAATTACGAAGAAAATTGAAATCTAAATTCACACTCTATCAATATAAGATGAGAATGATGAGAA

TCAAAACTTTATTATTTCACATATTCAATCTGCTTACATATACATTGTCTTTAATAGTTTTTAATAAACT

AAACTTTATTCTAACTAACTTGATTAACTAAGTGTCTCTAACTGACTAATTTTGAATTACCTCATATTTC

TCAAGAGCACTCAACTATCATTTTTTATTATTTGTTTGGTAAGTTATGCCTTATCCCCTTTGGAGATAAG

TATTTCTTTTTCAAGCATTCTCTCTTATTTAATTATATTATTTTTTAAATATATTTTTGATATTAGACTT

AACTTAT

>Ca_linc_0515

ATTAATTGATCTAAGCTTCATTAAGTTGCTAACCTTCCGTTAATCAACATACATCAAATTTAGTATAAGT

AATGCACTCAAAGGTAGACAACTCATCAACCTGCTTCAACAAACCCATTTTCGATTCCTACAATCAATGA

TGCTTCTTTGTATTGAAGAAATATCATATTTGGTCTCCATCTTGTGGATGTGTAGATTCATTTCGAAGTT

CGCTTGGAATGCACTACTCCTAGTTCGAGTCCAAAACAAGTAAACAAAAAGGAGCATAGATATCTAAGGA

TTTTATTTAAATAAAACATAAATGTATTTTATATTTTATAAATGATATTAATTTCTATACGGTGATGGTG

TAAATTTTTTT

>Ca_linc_0516

NNGAATGAGAAAAGCGTGGGGGTTTGGTGGTGGTGGTAGGAGGTGAAGCAGTGACAGTAGCAGAAGTGGA

AGCCAGTGAGGATGCCATCAATCAAGTAAACGAAACAACAGAAAAGGAAAGAAGTAAAGTTTGGATGTTG

GATCGATTAAATCAAAGGTTTAGTATAGTAAATGTTGTGTTTCATGACACTGGGGCACCTAAACTCCACA

AACCTCACACATATCCTATGTATCTAATTCTTC

>Ca_linc_0517

TGACATTATAACAAACGCACAGAGTATTAAATAACATAACAAAATATCATGTGTTCTGAAATTACAAAAT

AACAAATAGTATCGAATAACAACTTTAACAAAGTGATCTAATACATAATTGTCAACCAATTACTACCAAA

AGTAACCATAACTATGACATTAAGGTTCGTGAAGCGACGCGTCGTCCAAATTATGATCGTAATGAGGATG

CGAAGGAGGATGACTCGCAATACATGATCCACCAGCTGATTGACGGTGCAAAGCGTCAATTTTATCGCCA

AAAAATTGCTTTAATGCATCAAAATCATTCTCTAACTTCTTTGTACGCTCCCTAGCCTCTTCGGCGACCT

TGGTAGCCACTTGAGCTTGGGCCAATGCATCCTGAGCAATTTGTTCAGCTCTAATTCTAGCTGCCTTCTC

TGCTTCTAAAGACATCCCAGTCATAGAATTAGAAGGATTCTGGGAATGTTGTGTAAGCGATACACACCCC

TGTCGGACGTTAATGGACAAGTCTCCCGTACCATAAACTCGACCACGAGTGCAACCCCCTGCAGATTCAG

TCCATAACTTAAGTATTGTCCCTCCATCTACCTCTTTACCACTTTCTTCACTTGTTTCGCCAATTT

>Ca_linc_0518

TGGGAGTTGGACTAGGTTTGTGACTTGTACTATGTAGGGTTAGACTCCTCTCTATATATGTATATAAATA

GCGATAATTGTGCGGTGAGTTTGGTGCGACGGCGTCAAGATAGCAGTGAGGTGGGAATGGCAGATTGGAG

CAGTTAGAAGTTGTGGGAATGGGTTGTTTGGTTAGAAACACTTTCAAATTCATAAGCTTAATCCGCTTTG

CTTTGTGTGTTTCTTACCAATTCCGCCCATTCCTATTATTTCTAATTT

>Ca_linc_0519

CTCAGAGGTCTCTTCTAAGGAAAGGAAGCACCCCTTTCGATCTAAAGCTATTCCCTCTTCAATTTCAAGC

TTTCATCTTCTTAGATGTGGTGTTTAATATTGTCACGCATAATTTTTATTTCTTATTTTCTTTCTCAAAT

ATTCATCTTAAATTAATTTTCCATGAGAGTAAGAGGCATTGATGATGCTGTTGACAGAAGATAGAGAGCA

CTGATGATGAAATGCATGAAAAACAATGCATTTCATTTATTTGTGCTTTCTATACTTCTGTCATCACCCT

TCTCATATCTTAATCATTAAAACATGTAAAAATAATTAGTGTTATATTTTTTAAACTTTTTTAAAACGAT

GAAAGAGAAATTATCATTAATTAAAGTAAGGTACTATTTTACAACCATCTTCAAATATATTCGAACTATG

TTCCTTAATAAATATTACAATATTTATCATTAATCTAATAATTCATTAACA

>Ca_linc_0520

CTCCGTGCTATATTTGAGTAGCCTCCGTGCTATGTCGACTGGCATACAGAGGGTGTAATTCTAGAACGGT

TTTCTACAGTGCAGTAGAACTCCGATACAGTGAATCTGGGCTGTTGGTTGATACTCTGCCTTGCACAATT

TTGGGCAGTGCTTCGAAACGTCTTAAAGAGAGCGACCTAGTCCGCGACTCATCCCAGTAATATTTTCGGT

GGCATAAATTGTTTTCAAAGGTTTTCGAGTTTCAAAAACAACAGAAGTTTTCACTGACAGTTTCCGTGGT

GCCGTGTGTTGTAAATATATGGATATTGTATTAGTAGTTTTGTTATTTTGTTATTGGATTTGCATGTGAA

ATGGGTATTTG

>Ca_linc_0521

TCCAACATTCCCCTTACTTAAAAGCTCAATTGTTAGAAAGGAGAAAAAGAAAGAAAGAAAAAACAAGAGA

ATGAAAGAAAGAAAGAAAAAACAAGAGAATGAAAGAAAGAAAGAAAAAGGGAGGGCATAATATAAAAGAA

GAGGGAAAATGAAGAAGAAAGAAAAGAAAGAAAAAAAAAAAAAAAAAGATAAAAATCTCTCCATTGCATG

TTCTACCTCCAAGTTTACCCACACAGTTCATTTACAATATATGCTTCTTACAAGAACATTGAATACAAAT

GAGGAGAGAAAGATAGAAAGAAAGAGGATTAAGTTTCACTCTTGAAGAAACAAAATAAAGATTGAGGATG

GAAGTC

>Ca_linc_0522

CAAATATTTTAATGCAAAATGTGTTAGATCTCTTTTTTATTTGTTATGATGAATAAGTTAAACATATTAA

AGATATCTCGGACTGTAAAGAGTTAACATAAGATTATACTGGACCTTTTAGTCCTAAGATATTTTGTTGT

TGTGAAGTGCTGCTCTCTATAAACTATTGTATTGCTCCATGTTATGTGATCTCAGTGACTGTAATATCTT

TTTTTTTTTGTTGACAAGGAGTGATGTAATATCTGATCTTGTATGATTTTGGTTTATGTTCAAATTATAT

GTACG

>Ca_linc_0523

GTCCGTGGTTTTTTTTTTACCTCTTACCTGAGGGGGTTTTTCTACGTTAAATCTTGTGTGTTCATCTCTT

GTTATTATTTTACTTCTCTTTGTTCTCAATTGATTAGATCATGTGCTTCCGCATACCGCTGATACCCTAA

CAATCTCCAACTTATGACAAACTCACCGGAGGCGTCGTGTTCCTCACCACAATATTCTTTCTCTCTTGGG

TCTCCATTTTCATTATAAAATAAAATAAAAAACTAAAACTGTCGACTTTTAAAATAAAATGATTAATTTT

GTGAATCAGTAAAAATAGAAGAGAGTAAAATTGCAATTAAGCCTAAATTAAATTCAACTTTAAAAGTATA

CAGACTTATTCATACATACAACCACATACAGTGCATACCAAAATTAATCTTCTCCAATTGTACCGTATGG

AATCTAATTTAGACCAAGACTATGAATAAATTATTCAAAATTTGTTTCAACGTATTCACAAACATAAAC

>Ca_linc_0524

ATTACTAAATCGTATCTCAGAAATGTGACTATATTGACTATATTGATAAAGCTGCCAAAAAGTAGGATTA

GATGCAATCTCTAAAGCTGCCAAAAAGTAGGATTAGTATTTTATTTTTCTTCCAAAATTGAGATATATTG

ATAAAAATTCCGAATAGTGGATCTCAATTCACTAATCTCATATACGTCTCTTCACGACTGGCCGTGCACT

TAAAATCAAGATTCGTTTATTCAAACAAATGGAAAGAAAGTTGTAGTCATCGAGTTTATACTAGAAAACA

AAACATCGTACTGAAAAACAAAGAATCGGTAGGACAAGCACACACAAAAACAAACCGAAG

>Ca_linc_0525

AAAAAATAAAGAAGACAAATTCTGAAAATAGTGTTTAGAGTTGGATAACGAAACAAACAAATAACAGAAG

ACGCTTCAGATCTAGCAAAGAAGAAGTGTGAAGAGTAAAACAACAATTGGAAAAGTGTAGATCGCAAAAA

GACGAGGTACAACTTAGAAGGATGGTACTGAACAGCGGCGTGGTGATGAAGGTGGCACACGTGTCAGCTA

GGTTTTGTCAATACATAGCGTGCAATCCTGAAAGGTTAAGTAGCGACGCCGTTTTGGGACTCATCTTCTG

TCTTCCTTTCCAACGTTTCTTCCTCTCTCTTTCCTCCTATCTCGGCAATCGTCATCACTCCGATTGAAAT

CAAAATCAAAAATCCAAAAACCTTACGGTGAATAAATTCGTATTGTATATATTATTATAGCACTATATCT

TAGGTTTTTGTTTCTTTTTTTATTTTTGTCGTTTAGTTTGTTTGTCAAACAATCTCCGTGTTTATGTATC

TACATATTTAACTGGATTTTATTTGCTAAATTGGAAGTGGTGATGATTCTTAATCCTGATGAAAGCGAAC

TCAAAATGTTTTTTTTTTTAAAATAAAAAGAATATTGAGAGCAATGATTGAATCAGGGAATATATAATAA

TACAAAAATGTGGGTGAAATTCATCGAGTCAGAAACGGAGGGCAAATGTAAAGGGTTTGATAGCCTTGTT

TTCAATTTTCGGTTCGGCTCTTTATTGTTGTGTTCCCAGTCTTTTTCTTTTGAGTTCCTTTGGAGTATAT

TTGGTTCAATGAATGGTAGAGCAGGTGAGAAATT

>Ca_linc_0526

GGCTATTTTGCTCATTTAAGCCTTCAGCTCGAGTTCTGGGTTTGACTATTGATTTGAATAGTCTTGATAC

TCAAAAACACAAGTTAAATAACAACATAATCAAAATCATGATTAGTGATTAATTAATTATTTGTTTATCC

TCAATACACTTGATTTGAGATTTTGCCTCAACAACTGCCTATGATAAGCAACATGCTCTAAAGATGCAAC

AAGGTGACAATGTTCATGCCACTAATTCCAAGTCATGCAGTTCCGCTTATGACTACTTGAACCAAAAAAC

ACAACATCTAACTCCAATGATCAAGTTATGTTATGATGAAGAAAGTCAATGACTTTGATGGAAAGTTAAC

ACTCTGATGGTGACTGAGCTGTTTCATGATTCTGAAGAACCAATAACATATTCTGAAAAGTTCCCGCTGC

TCTTAAACATACGTTTAACACAAGAATCAAGGTAAATTATTTTCAAGCACTATTTGGACATTCTTGCTCA

AAATCACTATAGAAATTTCAGTTAAAGATTCATTTTGAAGTTATTCAAACTTCAAACAACATATTCCCAT

>Ca_linc_0527

CGAAAGAGATAGAGATGTAGGTATAAAGCTTCTTGATTGAATTTTCAATTAATTTGTCGTCCTCTCAAGT

TTTGCAGTGTATTTCTTCTGAATTTGATATCTTTATTCACTTGTTCATCTATCACTTTTTCCATTTTCAA

TCGACTATCCTTTGGATTATTGATCCATCAATTCTTTTCTTCTATTGCGCAAACATTATCCATCTCCAAA

ACCATGTTTG

>Ca_linc_0528

GATGACCCTAAATTTAATAATAAGATGGATTTTTGACCTAAAAATGTGACTTCAATATGAATTTTTATTC

ATACGAAGTTGCAATACTATGTTTGATGATCTTAAAGTTAATCATGAGATGAGTTCTTGACCCAAAGATG

TGGCTTGCGATATGAATTTTTATTCATACGAAATGAAGATAGTATACTTGGATGACCCTAAATTTAATTA

CTAGATGAATTCTTGAACTACAAAATGTGGCTTGTAATATGAATTTTATATTCATATGAAATTGGGATAT

TATGTTTGGATGACCCTAAATTAATCACGAGATGAATTTTTTTATCCAAAAGTGTGGCTTGTAATATGAA

TTTTATATTCATATGAAATTGGGATAGTATGCTTGAATGATCCTAAATTTAATTACGAGATGGATTCTTG

ACCCAATAATGTGGCTTGCAATATGATTTCATATTCGTAAGAAATTGAGATGTATGCTTTGACGACTCTA

CATTTAATCACAAGATAGATAGTTGACCAAAAACGAGCTTGTAATATGAATTTTTTTATTCACATGAATT

AGGACTAATATGTTTGGATGACCCTAAATTTAATTACGAGATGAGTTCTTG

>Ca_linc_0529

GCATAATTGAGTATACAGCCACATCAAGATCCATTTATGGTGAAGGCAAATGGAGTAGTACTTGCCTCTG

CAGAATAAGCAAGTGTACGGGCATAATAATGATTGTATGGCTAATCTAGTGGATTGGAAGGTTCACAAGA

GGAAGAATTTGTGTTGGAGGAACCAAACATTTGCAACCAAAAGACTTGACTTATAGGGTGAAAGTGTTGT

AGTGTCTCAATGATCTGAAGGTTTTAGGCTGAAAGTTGAGAGAACTGACTTGTAGATTCAGGTGTTGTAA

TTGAGGGTTGCATTAGACCCGGTTAGTCCTACGAATCCGACCTGACCCATAAGCAACGGGTTGGGTCTGA

CCCGGTCCAAAATAAAACAAGAACAAAAAAAAAAAGACAAGACAGAGACAAGATAAAAGAAGTACCAGGT

GCGGGTTACCCATCGCGTCCCAATTGCACTAACTTTACTTAGGGTTTGAAAAAATTAGGGTTAATTTTTT

TTTTCTCTTCCCTGCATCATTTCTTCTCCTGCAACTACGTATTCACTTATGAACGTTGTAGAACCTTAGA

ATTCGAAGAAGGTGAGCATGTTTTCCTACGTGTTACACCCACTACTATGATTGGTAGAGTCGTCAACTCA

AAAAAGCTTATGCCAAAAATATAAGGATGTTAATTTCAAAAAATATGTTTTATATATTGAAAGTGTGTTG

TGGTTAATAATACTATTAGTCCTTACTCATTGTAAACTCTTTTAGTAAAATATACTCTAAGAAGGTTAGG

AGTAAGAAATTATTTGTAAAGTAGTTAAAGTTTAAGGG

>Ca_linc_0530

GGCCACGATATGATGTCATGTGATTTGTTGGTTCATCTTATCCTTATGGGGATTATCGCGTCGTGCTGAT

ATGTAAGAGATTGCACTCTATAATGTGCTGATCTGTGAGCGATTGCAGTCTAGAAAACAGTGTAAGACTT

GTGATAGGCGACACTTTAGTAAATCGTGCGAGAATGTGATGGGTGGCACTTGGAAATTAGTACAAGGCAT

GTGAAAGACGGTACAATTATGATTTACGCCCCTTCGAGGAGGGTTTTGGTTGGAATTCTGGAATCATGTA

TTTTGGCACATACGCATTGCAATAGGGTGTTTGAAACGCGAGTCATGTTTGATATACTGTGATGATTGTA

TAAACATACTCAATCGTTGTTTGTGATTGTGTCGTAATTGGTAAGTGTGATGATTGATTTTGTGTTGTAA

GTGTTGAATGATTTCAAAACGTTTTAAAAACTAAAAATCTGCATTTTTCGCAGTTGTATTCGAATACGAC

AACACACATTTTGCCACTGACTTGCACTATATTCGAATACGAGATTTCTGTAAATGCTATGTGATATTTG

TTAATTTCGGTTGGTGACCTTTACAATTATTGTGGAAATCTGGGATTTGCTCTCATATGAGGATCAAGAT

CATCCTACCGGTTGTTAGACATACCAGACTGATGCGGTGTTAGGAGAATTTTGCGGGGCGCGTGGAGATC

ACTTGGGTCATATAGTTT

>Ca_linc_0531

CTTGAATAAGACATAGCAGCTGCACGCAGACTGTAAACTGTGCACCAGGTAAGTTCCCAGGAAAGAGAGG

TGGGTCACAACAGACACGCTTAAATAGCAAGTCTTTCCTTAGCCAGAACCTCCCTAAACAGCACTTTCAC

AGTATCACCTTTCCCCTAACAATACCAAGGATAATTCTTTTCTGATGTGGAAGATCAGACAAAGTTGCAA

CCACAGAGCATACTAAGAAAAATTATCCTACCGAACCTCTGAACCTGGCTTCTGATACCAGTTGTTGGGA

AAAATAGCATAAGTTCAAAAGAATTAAGACACAATCTCAACATAAGAATATAACGTGGAAACTCCAAAAC

CGGAGAAAAAACCACGACCGCTGCCTAAACCAACTCCAAAACCAGAGAAAAAACCACGACCGCTGCCTAA

ACCGGCAACCAGAGAATTAACACTATGTGAAAATTGTTACAACACATAAACTTCTCTCACTCACACCAGA

CACCCTAGTACAACCACATTCTTACAAAACAAATATTTAAACTAAGTCAGATACAAGCTTAAAGAGCTAT

TGCTGACTAGTGCATTTGAAAACAAATAACCAAAACCCAATATATAGCTTTGGCCTTCTCCTTATTCTCC

CACACT

>Ca_linc_0532

ACCAATATGTGTTTGAACATCAGATTTTTATTCAACAAATCAACCAACACACGAGTTTTCCATCAAACAA

TCACTATGAAGTTGCTATTAGGGTTCTCAAATATCTCAAAACTTCTCCAACACTTGGAAAAAAAAATTTC

AGCAAAGTCTCGTATTCGAATTAAAAAAATTTGTGCTTATGATACTCATAAATTTGTTAGGGGGTTATGC

ATCTTTCTTGGGGATTCTTTGGTTTCCTAAAAATAAAAATAAAAACAATGGATTTTGCATATTTTTTTGT

GATTATTTGGTTTCATGGAAATTCAAATAGCAAGCAACAATCTCTCTAAACCCCTGTGAAGTTGAATAAG

AGCAATGGAAACAACTGATTCATATTTTTACTAAAGCATTAGATCTCCTATCTTATCATTCTTTTCCAAT

CGCGATTTACTCAATATTTGTACTTCAACTTATGGAAAGCTTTTGGACCAAAC

>Ca_linc_0533

TGAGTCTCGAAAGGCCGCGTGACATTTAATTTTAAACTTAATTTCCCTGTTTTTATTATAGAAAAAAAAT

TGAATTAAAACTCGATTAATTATAATATTGTATTGAGCTTGGATTGGACCGATCAGCAGTGGCCTCTAAT

CCGACTTAGTGACTTTGTGGTGTGTTTAATTAACTTATTATATATAATTAACATTTAACTATCATCTAAC

ACTAAACCAAATCACACACACACAAACTCCTATGTCAAGAGAAAGTGCTTCAACCCATCAACATGCATGG

TAATGCTCCTAAATGCAATTGTCAACAAAACTGCATTATTTTCATCTCAAATACAGCTAAGAATCCCAAC

AGACGATTCTTTGGATGTCCCTATTTTAAGGTAACAAAAATTTAAGGTGTGATGTTGTCTTCGGTTCGAA

CACTTATAAAAAGATTTGCATTTAC

>Ca_linc_0534

ACACTAATCAATAAAAGTTGAATCAAGATTCTCATACCTTCCTTTGCTTGCAAAAAACCAAAATCATCTT

CACTTGTTTCATTGGATAAAAATAGAATGACTTGATTTCAGCAAATAATAATGAATTGAAACTCACTTCT

GTATTTGTTTCAGCAAAATGGGAAAAATATCTCAAAATGAATATTTGTGTGATGGTGTTCATTTCATGAA

AATGATTAACTTAAGTCATTGTTAAAAAGAGAAACATATATGTGCAAAAAATTATTTCATTCCTCTTATA

ATAAATGTCACTTTAACAAAACATAATTTATCTCAAAATAAAGATTATTATCAATTTTCAATACAATTTT

TTTAAAATATCTTTCATCTCCTGAAAATGATTAACTTGGAGATGAAGGAAGATGTTTGAATGACTAGCTT

GCTATTAGCATACATAGAGATTGGTGGTGGTGGTGGATCAAGGGGAAGAGATCCATATACAAATATATTT

TTTTTCCAAGAGAATCGACTAAAAAATATCATCAAAAGATCATTTTATTTTATCATCAAAAGATTATTTT

ATTTTTTTCGCTTTTGCAAATATGGATAAAACATTATAAAAACTCGGCTAAAATCTTATTTACAAAACAT

AAAAGATATCAATATATATTGATGTTCTAATTAACTATTTTTTTATGAAGCATAAATTCAAAGTATTGTT

GTTGTTCATTTAGAAATTTACATAATTATGAAATTACATTCCCATCTTCCTCTTTCAAAACCCCTCAAGA

ACAAGTAAATAATTAATGCAAATAAACACATTAATATCAAAGTATATATATTCTAAAAAATCCTATGGCT

AGTTAGCTAGAACATAAACAAACAGCGATCTACTTAGATCTAATATATAAATATCTATACCATATTTTTA

TTTTATACGGGAAAAAAAATTACCTGGATATATATATCATGTGAAGGAGAAAATAAATTGATTATTTTCC

TTTGGCTAGGCTTGTTGATGATACAAACTAGCTAAGAAAGGTAGGTGTGAGGAATGAAGCCTGGTCCGAG

ATCAATCTCAACTCATCTCATGCATGGAATTAGATAATATATATTATATACACATGTATATGAAAATAAA

TAAATAATTAATCTTTTATAAAGTAATGAAATGAGGGAAGAAGAAACAAATGATATGATGTGATGATCTT

GGATCAAACCTCATTCCAAACACCCCCTTTCCATCACAAATCTCTTTCTCTTACTCTTTGAGGACAAGCA

AATATTTTTATTTTTTTGTAAAAAGAAAGAAAATGATTTCGAATCAAACTTCATTCCAAACACCTCTCTT

TCCTTCTTCAATATAAAAGGAAGGAACAATATATGATGAAAGTCTAAGCATATGTTATATATTTATAAGG

TTTATGAGTGACATGCACTCTCTCTCTCTCTCTGCAGGTTCCATCTTATGTGTGTGTCTCTTCTCTTCTC

TTCTCTATTGAATTAATCTCTTTCTCTAGGTTCGAGAGAACCAAGAAAGGCTTGCAAGCACTCAAGTTTC

C

>Ca_linc_0535

GGATGGAAGTTAGTCATAAAATGGAGCAGTTCATTTGGTTCAATTAATCGAACCATGCACAGCTCTAACC

ACTTTCATGTCAAAAAGTGAAAGTAAAAGTAAAAGTAAAGTAAATTGAAATAATCAAAATGACTAAACCT

GGAAACAAGTTTTTGACATTCATCCAAAGCAATGTGCAATGTAAGTGAAAATCCGGTTTCTTCAAAATCT

GAACAAGATTGAACAATAATGTTCAAAATCAAAATTTTGAAACTGGGACCACAATCTTGTTGGCTGAATG

ACTGTTGTGAAAGGCATGTGCCTCATATTGATGTATATGTGTTAATGTTGTTGGTTGAATGATTG

>Ca_linc_0536

CTTGAACTAATATTAACAAGATGTCACATATCAGTTAATCCTAATTTATCTATAAAAATATTATTTTAAC

ATTGAGACCACCAAATTGTCACAACTCATGTGTCGTAAATATATAATATTAATAAGATGTCACATCTCAA

TCAATATCATTTTTCAAAAATAAAATTTATTTGATATGTGTAAGCGTTAAAATTTGATATTAGATTTCAG

GTAATAAAAAAAATATTACAAAGAGTAGTTAAAACAATTACATGGGCCTAGAGCGCGATCTACTAGGTAA

GTAAATTAGAATTATGATCAAACATTTTGATTTGATAAAAGAAAAAAAACTAATATAATAATAGAATTAG

AAAATTGAAAGAAGACTTTAGGTCTTCTATTGGTGGTTTAGGATGCTGCATTCAACATTTATGGCGACGA

ATCTCGGAAAATGTTTGGTGGCGAAAGAGGATGAATGTGGTGGTGTGCAGGTAACTAGGATATCCGCGAC

AAACGTTCGCATTGAGGACGTCAAAGGTGGGGGTCCCCAAATTCTTCACAGAGCGGCTAGTCGAAGATTG

AGGTTTCAACGTAAGTTTTCTTCTCTTCATCATCAATGGTATTTTTCTTTCCCCTCACAAGTACTCTATT

TCTATTTACATGATCTACTTTGACGAGGTAGTATAAAGTTGTTTGAGGTTGAAATAGTTGTTATAATGTA

TGGAGGAGTGTGGAAAGGAAGAAGGTAAGCTTGTTATTTCCCTTATACCTATTTATCTGTTTTTTATCTC

ACTTTTTTGTGTTGATTTTTGTTCTCAATTTATAAGTTAAAATGATGAATCTTTGAAAAAGTATAATTGT

GAC

>Ca_linc_0537

TTTCTATGTCAGTCTCAATTTCAAACACAGAATAAAATACTAACTTTTCTGCTAATCATTTTCCTTTTCT

AATTTTTTAAGCTCGGAAACCAGGAAAGGAATCTGTTCCAAATTGTTAATTTTTCTATGCCTAATTTCCT

AGTACTTTGTTACTAAAGATTTTGAAATTTCCTAAAAAACTACAATATCTAGTAGCTTGTTGAATAACGG

TTCCACTTGTGCACTTGTTGAATTTTTTTGTTTGAGAGGGATTCAACTCTTTTTAAACAATAATGCACAT

AAATA

>Ca_linc_0538

GTTAAATTCGTCTCAATGTTTAGAGTAAAAAATCCTCAATAAATTATTACACGAATTTTTGGAGGTTTTA

TTCTTTAAATCAAGAACATAGTACATGCATGCATCCCTCACTCTTTCACGCCATTTCTTGAAATAAACCA

TAGCTCCATATAACAAAGAAATTTGAGGAAGCTTGAAATATCACACTCTCAAGTCTCTGTTAAGCCTCAC

ATCCAAACCATGTTGCAAACCACTCTTCTCAAGACCAACATACAAAGGACATGATGATGGTCAGTTTCTG

TTCATCATAATGGAACGTCCTCCATTGACGCAACAATAAATGAGAGATAATTGCTCTTGCTTAATCTAAC

AATTTTTCCTTTAAAATAAAATAAAAAGTAATTTGATTATTGTGTATTGAAA

>Ca_linc_0539

AGATTTACATGAATAGTTTAGAGTTAAATTTCCTTTTCCTTTTCCTTTTCCTTTTGGCTAATGATTTTCC

AACTCTATAATATGTATCACAATGCACATCTGTTAAGCTGGAACCCAGTGGACCAGCACAACCCATTCAG

TGTTGGCATGCAACAAATGCCATGTTGCTTCACTTGCTTGATGAAGAAGTCTACAAAGGACCACGGAGAT

GCCAACTTTATTTTTTTCTTTTCAATTAGTAGCAAAGAGTCCGTGTGAAGCTAGTACCTTATAATGATTT

ACTGTTTCATAGCCAAATATATTCATAACGAGGGGACCTTTCAAAAAAAATTACAACCACTAAGTGAAGG

TCTTCCTCTTCCAATATTACTTGTTGGTCTGGTCCCCATTT

>Ca_linc_0540

TATGAATATGAACAAACTTACAATAATCTTAATTTAACAATCAATCAATTCGAAGCTCCAAAGTTTGATA

TTAAAAGCATTATGCTATCTGAATAAACTAATTAAGTTAGCTTCATCTTCTTTTCATGGATCTCTTCTTA

ATTCTATATGAATATGAACAAACTTGCAATAATCTTAATTTAACAATCAATACATGCATACTATATTCTC

CAAAGCAAATAGTAATGAGTCATTGACATGAACGAAGCTTAACATCAGTCGTGTAAAAACATTACCTGAA

AGGGTACAATCCAAGACTTATTAATTATAAAGCCTACCTCACATGT

>Ca_linc_0541

AAGACATTTTGGTACGATGGAATCTAATACGCACCATATCTTAGTCTTATTTATAAGGACAATACTCAAT

TTGATCGTGATGAAGATTTGAATGATGTGATACAAGGATCCAGTAAAATGCCTACGATGCATAATAAGCC

AAAAGATTGGATACTGAGAAGAATTATATTCTCGATGTTCTGATACAAACATTCCAAGGCATAGATGAAA

AAAAAGTTGTGCATTTTAATATAGAGTTCAAAAGTTTTAAATAAAAGGGACATAGATGCAAAATAATCCC

TTCTTCTGAGACTGGACCAATCGATCACTCAAATCACTTTGAGAGTCATACTTTTACCCCCATTGCCAAT

TTGCCATTAATAGACAATGGTGCTGAAATTCTTCCTTAGTATGTATGTATTTATGTACAAGTTAATAATA

TATTATTTGTCCTTATATTACAATTATGCTCTGCATGATTTTACCCTTCCTTGATTTGATTTATTTATGG

CTCTGTTTT

>Ca_linc_0542

TTATAGGATGTAAACAGCTCACAAGTCACAAATGAGAAAGCAAGTGGATTAATTAATGACCCACTATTAG

AAAACTAAGTCTAGTTAGGTAGAATAGTTCATTGTCAATTTTGACCTTGCTTGAAGTATTTGTCCAAAAT

GTGTGCAATGTTAATTTGTTTTGAATATTACTTCTTCCATCCTATGAATTTCGTACAAATAGTCAAATAC

ACGTTTTGCTTCCTCAATATTTTGTTATAGATACGATATCATGAGTATTTATCGGACGACCTTATCCAAA

TACTATGTGTGACGTGTCTTGATCTTTTGTCACTTGCATGCCAAGTGTCCAATGGATTAGTTTGGAATAA

TAAAGGGTGTAACTTTTATTGGTCCTTTTCATTCAGCACTAACTCACACTTTGGGAACTGGGATTGGAAG

AGAAAAGTTACCTTTTTATGTAAGTGGGAGAGAAACATGAGTATGAAATAGGGAATGGCACGTCACCTCA

AAATATGAAATATCATATCATAACATATAATAGTAAGAAGATTGACGTCAAATATTCTAGTAGATAAATC

ACAACTATTTATATATACTACCACTTAATATTATGATTGAACTCATATTTTGATTAATTTTGACAATTAT

AATTTAT

>Ca_linc_0543

ATCACATTGCTAAATTCCAATTTGAATACCTTATCATTACATTTTCGATCTTTCTTTAAAATGCCACGAA

ATGTGATTGATGAAATCTTCTCACTTCAAAGGAAAGTTTTATGGAGAAATAATCGTGAAAATAAGGGTTT

GTTGGGGCATTGTATACCTTTACATACAAAATATGGTAAACTAGGTGGAAATAATTTGGAGGCTTTTAAT

GTTGCATTATTGCACAAGTCGGCATTGGCAGTTTTTTGGTTGAAAACAAATTTATGTGAAACAAGTTATT

GACTTTTAAATATGACAATCTGCCACATAGGGTCATGTGTCATAATGAAGTGGGACGTTTCTCAAAATTA

TCACCATAGTCTTGTGTTAATCAATCAAAATGCTTCTTTCATGTGGTTTAGCCCTCATCTTCATAAGGAA

TGTTATCCTCTAATATGCATTTTACCACATAAGAAATTATGAGACATGACCCTATGTTATTAATGTAAAT

ATCACACTTATAATTATTCTATTTAGGAAGTCTATTATGGAGTAGTCTCTATGTGAGACGAGATACATTT

AACATAATCAAATTATTCTTAAACATTTGTACTAAAAGAAAATAAGTCATTGTTGCTTCCTTCCATCAAC

ACCTTGTATGCATCCTTAGATGATATACGCATCATCTTGAACATTACTCCAAGCCCAAACATCTTCCATA

TGTTATCCTTAAAAAAACGTTAATCAACATAGAGTTATACTCTACCACTAATCTTATTTTTGTTGGAACA

AATCCCTCCTCTGATGTCACCTCTATCCCACATCTAAACATGTCAGTCTACGAGATATTTTTATAAGATT

TCGAACTAAACAACCTAATAAACCGATTACTTAAGACTTCCCCTTTCAGCAGCAATGTAACAAAAAAGAA

GATAAATCTAATTTAATAAATTTCATTCAACTAGCAATACAATAACTTTTTAATTAGTTTAGAAATTTTA

GTTTATATTTTAATTTTGATGGGA

>Ca_linc_0544

CTCGTGTATGATTATGTGTGTTCTCTTTTGTTGATTTAGAGCATGACAAAGATATAGCGTTGAAATGATC

TCTTGTAATTTTGAACATTGTTCTGTTGTTGGTTCCACGCGTTTGGTTTCTCTTAACAGTTCCTTCTAAC

TCCTGACATAATATGATTATATACGATTATGATGACATCATTCTTAAAAACATTGGTGAATGATTTTTTT

TTTTGGATTATGTGGCATTACTAGCAATGTTAAAGCTGAATTGTTAGCTATATTGTTCATTGTCTTTATT

GCAAATCTGATTCTAAACTTGCTCTCGTTCACATTGATGAAGGGAATCCAAACAACCGCCTTTATGTTGT

TCGGTGAATGTAAATCTTATCGAAAGTTATAAAGTTCATGCTTACTCTTAGCTTTCATTATACCTTCAAA

TATGGCAATTTTTTTATTGATTGGCTCGCTAAGATAGGCTATTTTTTCCAATGAAATTGAAGGTCTCGTG

TATTTAACATTTATGTAATTGTAGTGCTGATGAATTGATTTATTTGGGGTTTCATCTCAATCTTTTACTT

GTTGAAATCATGTCATTCTTCTTTTGGTCCAAATACATTTCGCTA

>Ca_linc_0545

ACTCCTTTGGCATTTACAATGATAAAGACTATGTCCTCAAAATATCCTTGTCCCTTTCTAAAAGATTAAC

AATAAAATCCCCCCTCATCCCTCTTCACAATTCCTTCATCTTTTCAATTTCCACAAAACTCTCTCACAAA

TAATAAATAACACACATTTATAGCCACAATCATTCACCATACCCACTATATTGATCTCAATGTTCTTCAT

TCCATTGTCAATGTGGTTTTTTCATGGTGTGGTTCAACATTGAAGATGATGATGGTAAAAGAGAGCACTA

GTACTAGTAGTGGATGCATGATTAGGTACTCTTTCTTGATTTTGGTTCTTCTGGCTTTGTCAATTCTACT

TTTACCTTTGGTGATGCCTCCTCTACCACCACCACCTTTGATGTTTCTCTTTGTTCCTGTTTTTATATTG

TTGCTTTTATTTTTCTTGGCATTTTCACCTTCTACCGTTCCAAATATGGCTTCTTTTGTATCTTGATCAC

TTGTAACAATTAACTAATTAAGCATATAGCTAGCATTTTCAAATCTATACTGTTATGCACTTGTAATTAA

GTTTGAGGAAGATTAATTTGTGTGGAATATGATTTTCTTAAGTATCATCTTGAAGAACTTAATTATGAGG

TAGTATTCAATTTTGTACTTGTTAAGGTTTTCC

>Ca_linc_0546

GCACTATTGACCATCATATAGAATTTTGATGAAAAACAATTTTGACTGTGAAAAATACAAGTTGTTGATG

TATTGATATAACATGGATTAAACATTGTGTTTAACAGCACACATTTATTAGAAGATATGATATATGGTGT

GCCATGAAATTATGTCGATGGTTGAAGAACTTCATAAAAAAGTCAATATGGAAATTGCTAATTGTTTAGT

CTTGTTTTTTACATGTAGAAACTCATTGAGAGGATTTGCATCAAGTGAAGTATACCCATTGAGAGGATTT

G

>Ca_linc_0547

GAGAGCTTCTGCCGAAAAGAAGACAGAGTTCGTTGATTGGATTTCATGAGTCTGCAATCTGAATTCGATC

TCGCCACGGTTACTTTCATCTTTTTTCTTCTGAAATTTGAATTGAATTTTTTATATTATCTAGTTATTGA

AAGAGAACCAAGAAGCGGCATAAACGATTATTTTCGATCAATTGTTCCCAATTTTTTATATCAATTATTT

TTTTTTAAATATCACTGAGTCATAAACAATGATTTTCGAATTCAAGTTTGGTTGTCACTGGTTGCTTCGA

TTCTAAAGAGAGATGTCGAAACAAAAGAAGGAAATATCAAACTTTTTTATTGGACTTTCAATGAATCTGC

AGTGCTATAAAATTTTCCACTTTCAATCCTTTCTCCTTTGAATTTCTTTATAAATTCATTTTGGTTTGCG

GCGGTAACCTGTCGTTCTTGATTCAAGAACGTTTAATTTAATATCCAGATTATTCAAATCCAAGAATTTT

GATTTAATTGTTGCTGAAAAAATATGACCATATGTGGATTCAAATTTCTAGAAATATAAGCTTCTTGATT

GGAC

>Ca_linc_0548

CACATTTCATGTGGTTGTATTTCCACCTCTTATAGCATACAGCACACCAAGCAAAATATGATAATTATTA

TCAATGAAAAATGAAGTATCTTATTAATGAAAAAAAAATCATCAATCTTTTGGTTGAGATTTAACATTTA

CACATCCGACTATTATTGATTTATTATTACAATAGGTATGCCATTTATCTAGGAATAAAAAAATTAAAAA

CTGAATTAAAGTACACACTCTACTAAAAAAAAATACATAGATCCAAATACAGATCTTCTACGAGTGCTTC

ATAACGTGATGAAAAACAATATTTGTTTCTAACAAGAACATGCCACCACTATCTAATTAAGAATGAAAAG

CAAGTGCAAACATCTGAGAAGTAGGTAAGTTTGTATAAAAACCAAGAGGTATTATTGGACTAATTAGTTG

ATCCCAACACTGTGCCAGTGATGTAGTAGACATTGGTCTATTCATACAAAATGAACGAATGTTGCAACAC

AATATGAATATTTTCCCACGTAGAAGCACAACGTGACTGTCTATAAAGAAAGTGATAGTAGAGAGAAACC

ATAGAAGGAACAAAAGATAGTAATATATGCAACCGATTGAGGTTGCCATTGGATTTTGGAACTGAAGGAT

TAAAATCTGTCGATACTAAGCAAGAAAGCCTTTCTTTATATATGCAAAGATTGAACACACTACAAATTC

>Ca_linc_0549

TCATGAGTGCTAATCATTCTACATCACCGTATCATATGTCACGTTATTTTAAACATGTTGCATTATTAAG

CTAAACAATGAAAATGAAATACCAAAACTGTAAAACTTCAAAACCGGGGACTAATTTTGTGAAATTGAAA

AAATAAGGAATTAAATACACGTGCTAAAATTCAACTGGAATCATAAAATGTTACATATATTTACACGCAT

CTCAATCGTCTTATGAAAACTGAACGATCTAAATTTTAAGTTAGATATCTTTGATTTAAAAAACTTGGAT

CATCTTATTTTGATCGACCATCTAAAATATCCATGATTAGGTGCAATTATAGTATACTTAATACCACTCT

GTCGTCTCTTTTTCTTTTGCACAGTAAGACCTTGCT

>Ca_linc_0550

AGATATAAAGGATCCAATAACAATTATATCGGCCTTTTGTAAAGCAAAATTCTTGAATCCTACTTTTATT

TAGAATTCTAGTTCTTATGTACATTTCTTTCAACCTTGTCTCTCTAAACCTTCAACATTACAATTAAAAT

AGTTCTAAATTGCAGTCTCAATTGCAGATGCGGCCATAACTAAGAAATATTTAAAACCTCTCTAATCGCA

TCGCGACCGCAAATTACAACACAATATCAAAGATTTGTATCGTAATTGCAGACACAATTTAAAATCATAC

GAAGCATACAAGAGGTAATAATAACCAAGGAAATATAAGAATCTAGATAGTGTATACTATATAAGCAAAA

GGCTAAGAAAACAAACAAGAAGGGAAAACCACAAAGAATTCTTCCTGTCTGAGATCTCTAAGAGAACTCC

AATCTCACTTGTTCTGAGTAGGATTTGGTATGCTTGGTGGTGCTATCCTTAAACTAGGTTCCACAACATT

CCTGTTCTGTTTCTTGCTTCCACTTATTTTACCAAACCTTGTTTCAAAAGAACTAGAAGAAGATAAAGTT

AGCATCTTCCTTGACACTGGCAAACTTGTCTTTCTTGAAAAGCTTTGTTCATAATGGAGACAACAACTAG

AGAAGAGAAGAAAGTGAATCAGAATCAAGGCCAAGACAACAGCAAAAACTCTGCAGCTAGTGATCTTCAT

AATAAATTGAAAAAGGTTTATTGTGGTTAAGAAGAAAACATGCTAAGATGAAAG

>Ca_linc_0551

CAAAAATTGTAAAATAATTTTGTGGGTTTAAAGTGTTACTGATTAGTGTGATTTATAACGAATAACTAAA

GTTATATATATAGTTTTAGTCTTCATGTTCTTTTACAATTTTAAGTAATATGAGACTATTTCATTATGTA

ATATTTTCTTGACAACTAACATTTTTCTCTCATTTAACATATATACTAACTATTATAAGATTTGTCTATA

AAAAAAAACCTCAGCCTTTATTGAAATGATACATCTTGTGTTATCATTATCTATACAGATAGTCATAGTT

CAAAAGAATTTATTATATTCCATAAAAAAAAATATAGTTTACTCAAAGTTAAACCTCTTCAAGAATTTCC

ACTGAAAGCTAAATCACTCCAATAACTTCTACTTAAGATAAATTACTTCATGAATTTTCACTTTAAGGTA

AACAATTTTAATAATTTATTCTACATGTCAAAAACAAAACTAAAAACTTATGGTGCAACTTTCGTGTTGG

CTTTATCAAGAAGTTCTTTAGTAATCCACATAATCTCTGGGCACACCTTGCAAAAATGTCAGCCAATGTT

CATGTGGTAATCGACACCTTGTATAATCATGATTGTATCAACGCAATAATGACTATAATTTTTCATTAGC

CAAATATGATTCTTCCATAAAATTTCTCTATGTCAAACTTTACAAAGGAACTTGACATTTTATAATTGTG

CACCACAGATTTTCAAAAAAGAGAGGTGAGTCACAAATGACCATTTAAAAATCAACGCTTTCTTAGTCAA

AACTTTTTCAAATAATATTTTTTCAATTTCGTCATTCTTTTTTAACGTGGAAGATCAAAAAATATTACAA

CCACGTAACATACGAA

>Ca_linc_0552

AAACACAATAATAATCAATTGTATGTTTCTCTTTTAAATAATTGATTACGACAAATATACAACCAGTATA

TGAACTATATAGTAAATTATTACTATTGTATTATACACATAATTATTGAAATTATGTTTTACCATTTGAA

AACGTCCTCCTATGAATGTGAATATGTGTATCGGTAAATTTAAACGTCCTCATATGAAAGTGAAATATAT

ATAATAGCATCATTTTTGTGATTTAAATTGTTTACCTCTTATCAAGAAAAACTTAAAAGTTCCCCATAAG

TTAAAAATAAGGTTAAAGTTTATAGAGAGTGATAACATTCACTTTACAAATCCATTTTATAAGGATTAAT

TGTGATCAGTATAAAAATTTTACAAATTCAAACTCAGGTAAACTACAGTAAAATCGCTATAATAAAAGTA

GAATAAAATATCATAAAATGAAAAACTATATGTTCTATATATATATTGACATCTCCCATTTTTTGGTCTT

CTAGATGATTCTTTATGTCTTGGTGGGTGGTTTATTAGAGGAAAAGAGAAAAAATTATTTATGCCTATTA

TTTTGTTATTGTTGTGTAGAGATAGAGACAAATTTTTTGAAATTTGAATTAAGG

>Ca_linc_0553

CACAATGTTAGAAAGGATCCCCCACTGTGTTGTACTGATCTACTCTAGTGATCTACAAGACTGCTATAGC

AGCTCTCTACATATACTAATCTCTGACCTACCTACCTATACTTCTTCATCAATCATCATGCGTCTGCTAT

TCCCTTTATAGCTTTCTTAGCTGTTTTCTTTGCATGTTTCTTTTTTCAATTTTTCATTCCTTCTTTATTA

TAGTATCTACCTCTTTTTTATTTACATCTGTCCCCATTCTTTTCTTCTATATATTCCCTTCCTTCCACTT

CTTTCTAGTATACTATTATCTACCTTCTTTCTTTCTTCTTCTTCTTCTAGGTCAATATTCCCCTTACTCT

CAACATATTTTGTTCACAAAGGAATAAGTGAAGCTGCCAGCATGATCTAAACTTAGGTTAGAGAGGTTTT

CTTTTCTTTTCTTTAAAAGAAGAACAAGATGAAGATGAAAGACTAACCCTAACTTGGATTAGGTCATGCT

GTGACAGCCTCACTCATTCCTATTTTGTGGACCCAATTCATTCCTAACGATTTTTATCTCTTCAACTGAA

AGACACAACAAACAAACTTTAATGAAAAAGGGTGACTCATAATTATTTGATATCAATGAAATTACTGTTA

CAAGTATCTTTGTTATTCCTTTTTCCGAGTTTCTGTTCTGGCAGGCGTGCGATTTATTACTAACTTTTCA

CCTTTTTTTTGTTTCACTCTCTCTCCTTTTTCTTTCTCTCTTGTTATTTCATGCCAACCTGTTTTCACTG

TTTCTTGTTGACTATGCTCAATACTATCCCTGTATCGTTCTTCTCTCTTTTTATCTTGTTATAAGCTTCT

TTATGTTATTTTAGGTTTTCAGATACTCAGGTTCATTGTATAAATGCTTGTTTTGTTTATCTTTATCATG

TTTTTAATTCTGCATTTGTGCATTTTAATATATTTGTTTTTCATTTTTCAGGTGCAAGTGTCATCATTTA

TCCGCATATTAATTAATATAAAAGCTCATCTAGGAAAGGAAGGAGCTGTACTATCTTAATCAGCGTAATT

TATAAGCACAAACTCACACATTTTTATGTATATTTTTACATATTTTTAATTTGTTATATTATATTCATGA

TATTTTTGCCTCATCTTAGTTTTTGACGGTTGGTTGCAGTGTATAAAAAGCAGGTTATAGGTTATAGTTG

ATATTAAATGTTAGAATTTGTATTGTAAAATTTGTATAGCAGGTTATAAGTTATAGTTGATATCAAGTGT

TATTTTAACAATTATCATCTCACTGTAGAACAAATTTTTTGGAATAAAAAGAATGGTTAGTTTGAGTTTT

AGTCTTGTGTTTTCATTTTTAGATTTTTCTATTATTGTATAAGATTTATGAG

>Ca_linc_0554

CATATATAGATTTATTGATTCAAAAAAACGTACGCATGACTTTGTCTTATAGAAAGATCCTTTCAAGAGT

TTTGGTCCTTGTACTAATCATAATCAACATTTTTATTACTTCTCAATCAGGAGTAGAATGCAGGCCATTA

TTATTGCATTATTATCAATGGTCGTGGGATCATGGCCTTCTTTTGCAATCGCTTCCAAATGGTCCTGCGC

CTGGCACAAAGGGAGATACCACTCATCCCTAAGCTACATATACACCTTCAATTCAAATTTATTAAATATG

GACCATACTATATGATTGCTCAGGAAGCTGCTTGCAACCAAATCAATCTTACTTTTTTATGATATATGCG

ATTTATGAAGTAGTTAATAACTCTCGTGGTCGAAGCTCCACGTGTCCGTGGATTTTATATATCATGCTTT

AATTATTTTATTTTTTTATAGATTGCATGTCTCACATGCAATAAACAAGGATATGGTATTCCTTATAGAT

TCTTGTATTCACATTCAATTTCAATCATCCT

>Ca_linc_0555

CTGATTAGGGTTTCAATCTATTTCGATTGAGATTAGCAAATTTTGTTCATCCTATTTACGTTCTCTTCTT

TTATTTGGCAAATGATGAAGAAATTCTTATCCTACACACCGAATTTATCGTGTAGACTACAACTCCGCTT

ATTATCTGATGCCGTATTCGTTTTCTCTATTTTTTATTTAGGTTAAAACTTATGTTAATTTATTAGATTT

TCTTCTGATTTAGATAAAATGATTTTGTTTAAATTCCGAGATGTGTATTCTGCTATGATGATTTGCATAA

TACGTCTGCAAATACGAGATTGCTCGATGAGTTTGAAATTGTATGCTCTCGCTGAGCAGAATGCACATCT

TTTGTTTTGGTTACTTACTGTATCTTTTGTGAAAATGGTTTAAATTATTAACAAATCCAAG

>Ca_linc_0556

ATTTTATAAGCAGGATATACAAAAGAATCAAAACCTAGCTTCCTTTGAGAAAGCAGAGAAGGATTCACAG

AATGTTACCCTTAGTATAACCGGACAGTTCAAATGAACTTGTTTGCAAAAAGCTTTTGGCAGGTGAAAAT

CAGCTCGTGTATATCATTATGTAATACAGATTTTGATTATATGCCACTAAAAATAACTTCAAGCTAAAAG

CTCTGTCTTGAGCATTTTTACAAACTATAAGTCATTCCGAACACACTGAAAGGTCACTTAACCATGTAAC

CAGGAAGCTTTATTAAAACCATAAGTTGTTGAAAAAGGGAAAATAATACAGAATTGACAAGCTTTTCATT

TAATGCTAAACTTTGACTTCAGTTTCAGATTTCTCT

>Ca_linc_0557

TGCTTTCGAAGAATACTGGTTTCAATGTTAAATAAATAGTTTTGTATATGATATTTTCTTTTAACAAATA

CATTCCTGTTTATGTTCATCTTTAAAAAAAATTGTGGCATATTCGCTTTACACTGAAATGATGGAGAAAG

CTTCAAAGGAAGTAGTATGGCTTTCTCCATCTGAAAACTTTCCATTTACCATAAAATTAACAATCAAAAC

ATGGCGCAGATATCCAAGTTCACTCAACTTTCTTCATAATCTAACCCAATTTTCTGTCCTGTTAAGATCA

TTCATTGCCCCCATAGGTGTGAGGATTCAAAATTAGGATATGTACACTTGAACATGGAAGTAAGATAATT

TGCACCCCTCAC

>Ca_linc_0558

GTAGGGCATAAGTTTGCCTCATTTAGAGGGAGAATGAGAGGTGGAGAAAATTCAAAAAAGAGATAAAAGA

AAGTTTATTGGGAGGGGATTGGACCCTACAACAAGTACTCAGACATAATGTTTTCCAGCTAAGCTATATA

TAATGAGGTTGCTCTTTTCAGCTCTTTCTTTTTGCCAAATATGTCTCAATCAACCAACCAACCAGGTAGG

TCAAAGTTCGTTCATTCATTCATTGAATATTTTCTTCTTCTTTTTTTAGAATAAGCACAATGAACGTGAT

ATTAAAATCTCTCACCAATTTGCCATTCACGTGAAGTTCCTACATAAATCTCACAAAATCAAGTTTAATA

TCTCCCTAGAATTCTTTCAAAAAAGCAAAATTCACCTCATTTAGCCTAAAACTTTTACAACCACCACAAT

CTCAAACTATCTCCCTAATTTCATCCTAAAAAAAAGTCTTACAAGCAACAAAGACTCATCTTCGCTTAAC

ATAGAAAACTACAAACAAACATCAACTGAAACAAACCTCTGCATTTAAAAATGATTCTCAAAGTGAGGAA

ATATCACCTCTTGAATATGTTTCACCCCTCCATTGTGCCTCCCTCAACTTCTAGACTCCTTATAGAATTA

TGACGATGCTTACTAGATACCACCCCATGAAAGTATCTAATATTACCATTCTCTTCGTTCAACCACCGAA

CTCTAGACTTTTGCCACATAATGCTACAATTCAATTTAGATGACTAACTCAATGGTCGTTAAGAACCTCA

TCTCCTCAATATCAGAGTCAATCAACATATTCATCTCTCTTTTCAAATCAATTGCATTTAGACCATCTTT

GGAAGTCTTAAATTTTTCAATAACATTAGAACTGTGAATCTTATGTCAACCCCTCAAAGACATCTTAATT

CCCTTTATTTTTTCTTTCGGCACAAAACCACCCCAACCATCAAAGGACATCGAACTCTAAATTTATTTGA

CAAAATCACGATAATCTCCATAATTCGCGC

>Ca_linc_0559

TTTTTACTGAAATTTTAGTTTATGATGGAATTTGTTGTTGGTGAATCACACAGGAATGCTGCCAATTCAT

ATTTAGATAACCCTGCCAGGTGAGATAACACGTGGTTGTAATTTTATAGTTTTAGCATTACGCAATGAAA

AGCTATAGGGTTGTTGCATTTCATAAGTTTATGTGGTAGTAGTGATATTATTAAAAAAAATTACTATTAT

CAATATGTCAATTCAGTTAATAAATCTTTTTTTTTGAACAAATGGGTCTTCCTTCCCTACTATAGAGAAC

ATGTTTGTACATTGTAAATGGGAAGATATCTTGTTTCCCTTTATATATTCTTTGTTCCATTTCTTCTCAG

TAATTTATTCTTGTGTAAAATAACCGCTTTAGGAAACAAGAGATTAGAAGATGATTTGAGAGTGAAGTTG

AGGAATGATGGGGACAATCCTTTGTTAGAAAAGTTTGACACAGCTGTTGAAGAGGGGGATGGCTATAAAA

GAGAATAGTGTGCATATATTAG

>Ca_linc_0560

ACCTTCCTTTTCTCATTTAGAGATTCACAGAACACAGTATAATGTCTTTTTGTTACTTAGGTCAGAGCTA

TTTTTTGTAAAGTACTTTGTTTTCTATCCTTTTCCATGTTTTGATACGGAAGAAGGAAAGTCTAGTTATC

TAATTGACTCTGAGGACTGGAATTTAGTGAGGCCAGTTTTGTAGTTGAAGCTATTCCAAGGAAGTAACCA

GAAGAATCTACTTTGCTTTATAGATATTTTAATGTTGAAATTTCTTTACAGCAATCCCAAATTATATAGC

TAGTCTATGTGCTCTGATTTAATAATTAATCTAAGGCATATTATATCAACAAATGATCCCTCTGAAAAAT

CGACCATATAGATATAAAATTACATATATTGATGTCTTACTCAGATAACATGTTTTTATCTGCAACATGT

AGTCATCTGGATTTATCAAAATATTCACTTTTTAAATGTTGCGTTGCGTGCACTCAAGCTCAACAAGGAC

AATATTTTCGAGTTCAGCACGTTTGCATGGCTGCTTCCTTCATTCTGCTAAATCTTTTGGCTTCTGCGAT

TAGAGGTAGAAACAACATATCAATTACCAAGTTATTGTTTCGTGTCAGTTACACCTTACAAATGTAGTAG

AGGCTTCTAAATTGCAATAGTATATATTTAACTCTTGTATATGTCTATTATAT

>Ca_linc_0561

ATAATTGACAGTTTGACACCAATTTTTACTTTAAGATAAAAATTCTTAGTTCGACCACGGACATGGGCAA

ATAGATCAACAGTTCTATTTTACCAAAAATAATTTACCTTCAGCTTCAACATGACTTCCCTCTTCAGACA

TAGAATGGTTATTTTGGTTGAGTTCATTATAGCCATCAATTGAGTGCCCTTCCTCTTGTGATTGTCCACA

ATTTTGAGTAGTATTCAACTTCCTTTTGTGCTTCCCCATGGTACTTTCTACATATGCCAAAGCCAAATTA

GTTTTTAATTGTTGTAAAAAACAAATAACCGATGACAATACTAATCTACCTCAAATGAAACACATAAAAT

GGTAGGGAGCAAAAATAACTACACACCTTTGTAACTTCAATAAATTAGAAGTCAAATAAATTGATTGATA

AATAATTTGCTCTATATATTAGAAGAAGTCTCCCTCACTCTCATCCTCTGTATCATCACCACCCTCATCA

TTTTGGAGAATGCTCTCCATGGATATTGTAACTTCCATGATATCATGATTATCATCAACTTCTGGTCTAA

TCAAGTCATTGTC

>Ca_linc_0562

AGGAATATGTATGACATCTTTACTAAATCAAATCAACATTCATCCTTTGCTGGTTCATGTTATTTTTGTT

GCAGAAAGGGACGTTTTGTTTCTAATTGTAAACTAAAAAAACTAGCCAACAGAGGATGGAAACAGATTTG

GATAGCAAAGACACAAATGTTTGAAACTAACCTCCTAGGACCCAATTTAAATTGGGCACCTAAAATCAAA

TTTTGATTGTGTGTTGCAGGTGTGCTTGACATCCAATAGGCATTCTTGGTACCTAGATAGCGGATGCTTA

AAGCACATGACACGAGACAAGTCTAAATTCTTGTCTCTAACATTAAAGGAAG

>Ca_linc_0563

GTCTTTTGAAGAATTCTCAAATCTGTCAGGACTGCCCTTCCATGGAAAATATATTGATGGCAGTTCTGAG

GCTGAAGCTGCAGATGAAGGCTGGGAAGCTTCTTTTGATAGACACACCGCTGTGAAGGACCTGATGGTCG

ACAGCTTTGTCGAAACTATTTCGCTGCCGATTGGTCAAATGAAGGTCCATCATCGGATGCTCATGTACAC

ATTAACCCGGATGCTGGTGCCCCGATCCGGAAACCATGTCGTCGTCCAGAAGCTGGACATACTGCTGTTG

TGGGGACTGTCCAAAGAGCTCAAGATGAGCTGGACATGGATAGTGCTGAAGCACATGATGGAAGCATCAC

AAAGCAAGCTGGTGCTGCCCTATCCCCAGTTAATCACCAAGATTTTAAGACATTTCAAAGTGTCACTTGT

CGATG

>Ca_linc_0564

TCTCCTTCCCCCTTTCCACTATCGCTCTCCTCTCCTTCCCCCTCTCCATTATCGCTCTCCTCTCCCTCCT

CTTCTCCACTATCGCATTCCTCTCCATCTCCCTCTCCGCTGCTCTCCCAATGTTTCGTTTTTCCCTTTTT

TCATAGTTGCCCTAATTTCCTTTGATTTTCTTATTGTGTTAATGAAATAATTTTTTTATTTTCTCTGATT

TTATGATTGTTTTCTATTATTGATGTTGATGTTGAGTTGAACTTATGGTTGTTGATGTTGAATTGATTTC

CTCTGAATTTATCATCCCTTTGGATTTATTATTCCTCTGATTTTATGATTGTTTTATGTTGTTGATGTTG

GGTTGATTTCTTTTTTTTCATATTTGC

>Ca_linc_0565

ATTCAATGTTTTTCTCTTTGCTCAAATTCTCAAACATCTTACTAGATTCACAGAAGATCCAAACACCCAT

CACCAGACCGACCCGGTGACAAATACCAACCAAACGACTGAGCCATCGGCGGCGACTCCGGCGGAGAAGA

AACGAACGTCAACATTGAAACGCAAGCAATTAGTACCGTCCTGCATGGCTGAGTACAATTCCCCTTTCAG

TAAAAATGTCTTTTCACTTGGAAGTTAAAAAGAAAGGGTCGTAAAAGAGGAGTATGGGTGTACACCCATA

ATTTTTCAAACAAAATAGGTAAATTGCTATTAATATGAGAATCTTTGTG

>Ca_linc_0566

ATCCAACTATTGTGATAATTCTAAACAAAATTTATATCATGTTCACAAATCAGAACTAAATATTAATTAA

AACATCATCCAAAAAAAAAATACTAAGTATAACCAAGTACATAATACAACCAATTTAACAAAATAAAAAC

AAATACTACTCAATATGATCACTACCAATGTCCTCATCCCCGCTATCTTAATCAGTTTCATTGTTGGGTT

GTGGTGATGAACTTGGACGAGAAGAAGATCCAACAAATCCTAAATGTTGCATCAGAAAGGTTAAACTTTT

GTTGGTCTCGACCATCTCTTTT

>Ca_linc_0567

CCACAAACCAACACTACTCTTCATCAATATTGCACTATACTAAAACAACACCTATACTCTAAATACCAAA

TTTCTAGCTACATGAAACTACCTATAGAAACAGAGTGGTGTGTACCAAATAATGTTGTGGACATTATGTG

CATGGATAACCAAATGTGAGGCACTGATAACAAGATCAGTGATATAAAAAATTATGAGATCTCG

>Ca_linc_0568

GAAAAACAAAAGGCCATTACAGAGCAATATATCATACAATGATGTCTAACCTACTTATAGCATGTGTGGA

TAATAGTTTGCAAAGCATGGTTTGATAGGAGAGTAAAGCAACGTTTTCTTCATTATGCTCAAGCCAATCT

TTAAGACTTAAAAATCACGTTTAAATGCAAACCAAAGTGATTTTGCCAAACATGTCAATTAAATATGAAA

GAAACTCAGTTTACACACCACAAACTGAGGTTTGGGAGGTGCAAAACGCAATCCAAACACGAACTTATAC

GTTACAGCATGAAACAAATAAAGCTAAAAGCCATGGTTATTAGAAAATATATGCCTCCACAAAGAGGTTC

ACGTTCAACGATTGGCTACGTAGAATAACCTAGCGAAACTCATTGAATAGCAATGAACTAACAAAGCATG

ACTCTTCT

>Ca_linc_0569

NNGAAAGAGAATATTGAAAAAGAAAGATAGGTAAGAAGGTTGTATAGTGTTGCTTTAGAAAGTGGCTTTT

TATTTATGGGGATGGTTTGAGACGAGGACGGTCTAAGGAGGTCCTAATATTTAGTGTTGCATGTGAACTA

AGTAATAAAGGTTGCTCTAATTAATTGGCTCGATAAACTTTGTGGACAGTAAAATGATCTGGACCGTAGA

TTTTATTTGGAATAAAAATGTGTAGATGGAGGTATCTAGAAGATTGACCATCTGGTCGCGTGCATTGAGA

ATCAGAGGGATTTCAGGGTTTAGGCATGGCATGAATGAATATTGAAAGTGATAATTTGTATTGCTAATGT

GATTGATTTTGATTTGGGGAAAAATAAGTGCCTGATTAAGTATGAGATTCTGAAGATCTGTCTTTAATGA

CACTTGACAAGTACCAGCTGACATGACAGCATAGCAAGAATAGTACTAAGGATTAACTTTTTAAGCTTTT

TTTATATTAAAAATTTGTACGAATAGGAATATCCGTTTGGATTTGTAATTTGTAGATATTTGTTATTTAA

TTCGATCTCTTTCAAAGAGCTTCATCGAAGAAAGAAAAACAAATCTGATCAATTGCACTATCCTACTGTA

TT

>Ca_linc_0570

GTTTTGCCACTGTTTACAAAATGCGTCCACACTCAGCAGAGTCCTTAGCAAACAACAACGTGAAACCTCA

AATTCCACCATTATCGTTGGTAAGTATCTCACTCAAACTCCAAAACCAATTCTATAGTAACAGATGAGAT

AAGTTTTGCACGTTTTGAAATTAAAATCTTAGATACCATGAATTTTATTGGACACCTGGCAAAATAAACT

CAAATTAATTAAATATTAAATAAGGACATGATTTCAGATTATTAGTTACTGTACTCACTTAAACAATAAG

TTATTAGAGTATTCATCCTCTATACAGACTTATTTTTATTTTGGATTAATAAATTTTAGTATTAGATCAA

ATGACACTTTTTAATAAACTTATGTACAATACAAAAATATATTGGATCGTTAAATGATATTTA

>Ca_linc_0571

CAATATATTTTCAAATTTTATTATCTTTTCCTTTTCGTTTTGAATTTAAATTTGTGATTCTTCGTTATTT

CTCAACTTGGTTCTATAATATTTGAATATTCAAGATTGAAAATGCTTTGTGATTTATGATTCGATTATTT

GAAAGTTTAATTGTTCATTAGCCATTATCTTATTTAGTGATATATTTCACTATAATTTCATTCTTTTTAG

CCAACTTTCTTTATGTGCTTCTACCATATGGTAGTAAAATAATTTTCTTTGTTCTTTTATTCTATGGTAT

TAGACATTTTTTATTCTCTTTGTGCTTTCATCCTATGATAATTATGTTTTGTTAGTCAAATTTGACATTT

GAAGATCCAAATATTGAAATTCTCGATTCTATAATTGAGTGTTGTGGAAACACCAAAAAATTTCAAGTTA

GTCTAAAGTAGGTCGAATACTTCAACGAGAGTCATGCATCTCGATCACTACCACTTTCTACATGAATTTT

ATTGTTTAGCCGATGCGATTATCGTTTGTATTATTTTTCTTGTAATTTTCTTTCATTAGTTTCATTGTAT

TTTTTATTTTTCTTGTATCATATTATTCTTCACAAGTTGGAAATGGTGTTACATACTTTCAACTTATGGG

AGTAATTAAAATATATTTATAGATATGTAGAATTTGTTAGGTTAGTTACATTAAGAACACAC

>Ca_linc_0572

TTCAAAACCGTCTTCTAATACAATTTTTTAATTTTCCGTAATAGAGAAATTTTGTATAATGAGAAAATTT

GACCCCCGAAACTCTCCCCTTTCAAAGTCATCTCTTCCTCTCACTTTGACCTTCCATTTTTAAAGCCCGC

TTCTCCTCTTCCCTCCTAAACTCATAAACAAAGTCTTAGTAACACAAAACACTCACTCACCTACGCATAT

AAACCAACCTGCACAACACAACAAGGAAAACCAGTCTTACTATAGTTAATAGCATGAACAAATCATCGAT

AAAAGATTACTTTGTCACAAATAAGGGTGAAAATAAACACTTGACTTCACTTCCATAAAACTCACTCCAA

T

>Ca_linc_0573

CAAGAATTTTGTATTGTTAACAAAAAATTTATTGTATGATACTAATATCACACTTTTATCTTGTATGATA

TTAATAATTATATAATATTAATATTTTATTGTTAATAAAAACCAAATCTTCCACATCTTATTACTTACGT

TTTAATTCTTCCACCTCGGCCAAATCAACTTTCTACTGTCAAATATTTCATTTTTAGTGCCTTTAAATTT

TTT

>Ca_linc_0574

CGTCTCTCTTTCTTGAACATATGTTTTCTCTCTAGCTTATAAGTAATTTATTTGTATCATGATTTATTTC

TCTCTAGGTAGGATTGGTGAATCAAACCAAAGTAATGCTTTGAGAAATCAGTTAATCATACAATATTTCA

AGAGTTTAATTTACATGCATGATATATCAAAATTTTATACTATATCAAAACTATTTACGTATGTGATACT

ATTTGGTTGGAACCCTTCACTGTACATAACTAAAATTAATTTAGGACTAACAGTACAATTTAGTCAATTA

TAGTTTATTATAATATACAATCAATTTAATATTTTTAATATTCGAAAAATATTGGATCATCATATTTAAA

TATTTTTCAACTTAACACAGCTTTACCGAATGAATTTCATATCTGATCATCTTAACAAAAAAAATCTTTA

ATTATGTACAAAATATAGATTCAATTCATTAAAATTAAGAGTCGGAAGTATTAAGTTTATATAAAATAAA

TTCTGAAATTTTCTCAGACAAATGCCTCGATCTGTCCTCATAATTGCTTAGAATGGAAATTACATCGGAC

TCGTCATCGTCATGATCATCATGGAGAATACCACATTTCATAGTAATTCACAAGAGTCATTTTTTCAGAG

TTGTTCACAAACAGTAAACTAAAATTTG

>Ca_linc_0575

AAAAAGTGTTGGATTTTAAATTTCAAAAACTATTTAAAATTCACAATTTATGCCACCGAAGATATTACTG

GGTTGAGTCGCGGACTAGGTCGCTCTCTTTAACCGTAGAAAACCGTTCTAGAATTATACCTCAATATGGC

AGTTAAAGTCACTCTCAATATGAGAGTTAAAGTCACTATCAATATGGTACTATGACCATTCATAACTACG

GTATAATAACCGTTCAAAAAGACAAAAAAAACGAATGGACTACAGCATAGGACTACGGCATAATAACCGC

TCTAAAAACAAAGTGATTACGGCATAGCAACCGCTCTAAAAACAAAAGAACTACGGCATAACAACTGCTC

AAAAAACCGAAAGAACAGCATAACAACTGCTCAAAAAACCGAAGGGACTACGACATAATAACTACTTTAA

AACCGAAGGGACTACGGCATAATAACCGCTCTAAAACCGAAGGGACTGCGGCATAATAACCGCTTTAAAA

CCGAAGGGACTACAACATAATAACCGCTC

>Ca_linc_0576

ATGGTTTTTAATGCAGTGCTCTAACTTGATAGGTAGTGTGCAATTACAATGTAAAACAACTTAAAGTGAA

TGTTCATCAGCAACTTAATCCAATCATAATCTGATAGGACACGACGGTCCATTGGGTTTGAGGACCCAAT

TCACACCTTCTAGAATCTGTTGGAAAGGGAGGGAATATTCAGTCTGTTAGAATCCTCCCTGATTTTTAGG

GTATTTCTGTTATAAGAGTGAGGAAAAGGTAGTTAAAAAATCATTCAGAAAAAAAATATTGTTTGTCAGT

TACGAGAGTCTCTCTCTGGATTAAGAGCAGATTATGCTCTGGTAGGAGTTCTTGGAACTGTGGTTTTCTT

TCTTTTTCTTGCTGTTCTCACCATTGTAGAGCTTGATGCTCATAAC

>Ca_linc_0577

CAAACATCTGAATGCATGTTTCTAGGAAACTTGGATATGTAACATTTTTATGGTATTGATTTGACTAAAT

AGTCCTACATAAATGTACAAATAAGTTCCGCCTCCAAAGATGCCAAAATCTGTATAAATTGGCAAGTCAA

AAACTGGAATCCCAACCTCAGTGACTTTCAAATATCCTCTGTGGTACTCTAGACTGAACTTACAGAGAAG

AAATCAAGATAATTTGGACTCATTTCTGAAAGCCTATATTCTTTTTAATTGGCCAGTCAGTCATAACCTG

CAAGTACAACGCTAAGTTGTTCCAATAAGACACTCCCTTAACTTCAAATATTAGTAATACTTACTTGTGA

GATTTATCGATCACAAGGCTGTCTTCTCTACCGGGATTAACACCTCCTTTGTCATTTGTCGATGAGGCAT

GACCACCATCGAGTACCAAATTTGATTGTTCAAGTCCACCAGTTGTTGCTTCACACTAGCATAACCTAGG

AAGTCGTACCCTTATAGAGGTGGTAGTTGGCCAAGATCCGCACATTTCAGATATCCTCACGAGAGATTAG

GAGTCTTATGGTCTGTGTGCTACTTGCTTATTGGATAGGAGTCAAGGTATAGAATTTGTTACGAACTATT

TAAATCATGTTATAAGTGGACTTCCAAAAGGCGTATCTTTATGCTGATAAGTTAGTTAGGTTGAAGTATG

TTTCCAGATAAGGGTTTATCTCCAAGCAACTCACTTAATAGTAGAGTGAGCTCCAAGTCAATAATGGGTG

CAGATTAGCCTACACTGGTTGGAGCTTAGCTTTTAAGGCACATTGAATCTTGGGCCTACTCAACAAGGAC

ATTGTTTGGTGGATTCGTCCAAGTCAGTAATCTTGGAAAGCATAGCAGCCTAAAGCTATGCTTAGATCAG

TGTCATAACACCATTCAATGAAAAGTTATTTTTCACATGTCACCATGCATCCAAAGTCCAAACATAGTAT

AAATTGATCCTTAGAAGAAAAGTTTGGACAACAAAATCTGAAGTGATTGTGAGTTATCAGCTGTCACTCT

CAAACAGTTGTGTTTGTACAATGTCTTTCCCCCAATTGAAGGTTGCCGCCACTTGCCGACAACAGACAGA

ATTGCAAAGAATATATAAGTGAAAGTACAAGCTCAATGGCAGCCCCCCAAAATGGC

>Ca_linc_0578

GAAATAAACAAATACAATGATAATGATATATTGGGGGATAAATAAAGTTGAACAAGTAGTCCTAATTAGT

AAGGTCCGAAACAAGTTTAATCTTCTGGCGATGAATGAATTCACGAGCTTTATTGTTAATTTGAGGATCA

GCTACACCCCATCTTTCTTTGTCGTAATCACTAATATGCCACACTTTACTCCCACCTTCAAATCTATTAT

TATTATTATTATCACATTTCCTAATCCCTCCTTTCCCC

>Ca_linc_0579

ATGGGAATTTTTATAATCTTCAAGATGATATTGAAAGGAATACTGATCGTTTGGTGCATCTCTCACCGTA

GAAATTGTGCCGTAATGCAGTCTGGTAATTGTCATTCGACACAATAGGGTTTGTAGTACTTAGTGCAATT

TTGGTCTCCATATTGTTTTAGTGCAATTTTTGTCCTATTTTTAAAAATTCAAACATTCAGTCCAATCAGC

AATTTGATTCCAAATTTAGTTGATGTGGCAATATATTAAAGCCATATGGAATCCATGTCATATTTAACTT

GCATATTTAATTAGTTAATATTTTTAATAAAATGTAATGTATTTTATTCAGTTTTTTATTTTTAAAAATT

CAACTTGTCAACATAAATTAAGAACATAAAATCAAAATGAGACAAGATGAAAGAACCCTAGGCGTCTTGT

CATTCAACACTTTCTCCCATCACAGTCACAAGAGTTGGATTGAGTTGGTCGTCCTCCTTCTTTCTCTCAT

GTGCGTTCAAACCCCTGCACTAGATGAGTGATAATGATGATGGTAAGAGTCTGTTTCGATTGTTTTAGGT

TTTGGTATTATTTGTTTCGGATACAATTGCATTTGCACAAAGGAACAAAAACCCCAAAGAAACCTAGGGC

CTCTTTTGTGTCGCTCAGTTTATATTCCCTACTTTATTTTTTCGTGGAACGAAAGGCTAGTTATTGCTAC

ATATTGTCGGTGTTGATGGAGGTGTGAAAGAAGACACCAAAACACTGGATCTATTTATTACCTTCTTCTT

CATTACTTAATCTGTTCTTTTGTCTTTAGATTTACAGATTTGGAAAAAAACAAATGCTTCTCACCCCCAC

TGGATCCAAAATCCCCTATTCGTCTTCTGTGATCATTCTTGTTTCTCATTTTTTTCTTTCACTTTTTGAC

AAAAGTGATTGAATCTAAGAATGAAGGCATTCATATACCATTGAACTCTACACTTTATTCATTATCTTTT

GAACTATATGTCTTCTGTGGAACCAATGATATTATTTTTCAAGAAACTTTCTTATTTCTTCTTCGGTTCT

CTTTTTA

>Ca_linc_0580

GTTGTAGTTGACTGGTTCTAGTCAGGGAAAATAACCAATCTCCTCGTTGATCACCAGAGAAATGTGCTTC

AACATGAGGGTCATCAATATGATCAAAAGCTTACTCACATGCCTATATGATTTTCTTTGACAATGGGAGC

AAGATAAAACAGATAATGTTGGTTGCCTCATGGAGCGCATTAATGTCTGATTTGGCTAATTTGCATCATT

CCAGAGCCAAAGGGGAAAGGGAGCCTCTAGATCTAATTTACTCTTGGTTCAAAATAGTTACAGAATAGTT

GACAGAATTATAGCCACTATTTTCCATTGTTAATACCTAATTGCGAATGCTACAAATAATTAATATAAAG

ATTTATGGAAGAATTCTTTAAGATT

>Ca_linc_0581

CTTTAATTTGTGAAATATTATAAAATAGAATTGAAGTAAGATGTCATATAAAATAAAATAGATTTTTAAA

TAGAATAGCAAGCCATACTAGACTTTTATCGATGTCAAAACCAAATCTAAAAAATAAGTTTACGCCATAA

ACTTGACTTAGATGTTAAATTTTTTTAATGGACCAAACTCAAACCGTGTAAAATCTAACTTATTCTCACC

CTAATAAAAACACACAGTAGAACATTTAACCATAATGGACTACAACCCTACAATATCCCTCCACCTATCT

CTATATGTATTAACTTTTTAAATATTAATATTATTTATATTTTATTTCGGAAGTAGTATATCAAAAACCA

AAGACCCAATACACCCTGGCAAAATTGTCTTCTCCCGTAAACCCTAGAGTAAGAACTTGTAAATCAGTCT

TCTAACAAACCTCCGTACATCCTATGTCATAAGCAAAACGTAGCACGATGAGGAACGAAACCGACAGTTG

CATCTTCACAGGAACATATGAGCTCTTTTCCGTAAATTTGTGCACATGTATTTCTATCTTCATAGCAATA

GGAGTTTCTATCCCACCAAATATTGTATAGCATTATAGCTGCAACTTATAGGACATGTAAATGACCTTTT

GACAAAGCTCTTTCTATCTACATAACAAGTTTGATTTGAATGTCTTTAAATTTATCTCCGAATAATGGAA

TCATATTTGTTTATCCCAAACACACTACTTGACAGTTTGATTTATAGTTTCCACTTTTTCCTCACACCTG

CGAAAAAGAATAGAATAATCGATGCTTTTTTTTTAAGGTTATCTCAAACATTCACGGCTAGGTTTGTTAT

TTTCCAAAAGAGATACAGGTACTCCTAGAGTAATATAATCCTTTCTCGTTTTTTAAAAAAAAAATAGTAA

TAATACATATTTTCTTGTTAACAATAGTATGATAAAAAAAAAGTGTTGGAAACATCAATCTAACACTATT

TTTATCATTCGTTATTATTTTTTTGAATGGAAATACTTTAAGTCTCTCTACTTAATGAATGATTCATTCT

TAAAATTGTGAAACCTACCTCAATTTAAC

>Ca_linc_0582

CTCAAATTTATTTCATATGAATAAAAAATTCATATTGAAAGCCACATTATTAATCAAGAATCTAACTCAC

TTTTGGGTCATCCAAACATAGCAATAGACTTGTCTAAGGGTACATATTGCAACTTTTGAATTTTTATCAA

CCTTTTCGGTCAAGAATCATCAAGATTATACATTCTTGGATATAATACGTTAATGTTGCAAACCAAAACC

TACCCCAAAACATTTGTGTCAAACACAGTTTTGGAGACAGAGAATGTTTGCTCAATAGAAGAAGAGAATC

AATAGATCAGAAACCAAAGGATAATGGGTTGAAAATGAAATAAAGAGAGATTTACATATCTTCTATATCT

GGCTTTCGAATGAAGCAAATCATGGATAACAGAAACATAAATCCAAATAACAAAGTCTTGATTTCGTTGT

CATCATTTTACGGCACCAATTCATATAAGAATTCTAGGGTTCGATGACCAAGATTTATAGAGATCGTGCG

TTCTTGGTCATCTCTCTCTTTCTTGTGACTATGCGATCATCTTTTTCACGGCAGCAATTCAAATAAAGAT

TTCTAGGGTTTGAATAATCAAAGAAATTGAGCGTCATTGATTGAATTTCAAGAACGGGATGTTACCGTTA

CCGTCAACGTCGCAACGAATAATAAACAAATAAAGTGTCTCAAAAACATCAACGGCAAACACCGCTAGAA

TCGATTAATGCAGAAGATTGAAAATTGAGAAGGAGAAAAAGAGATATCAAATTCAAGCGATTAGGGTTGA

GAGGACACACCTCTTTATCAGTCTTTCTATTATTTCCCACTGATGAAGCAACCGCAGACTTGAAACCCAA

GGAATGATATGTTTAATTGGTACTGCTCG

>Ca_linc_0583

GTAGGCTTAGTGGGCCTATAGACCCACATTTTCAAAAAACAAAATTGACAACTCTAATCAAAAGTGACAT

AACTCATTCAAGCCTTTTAGAAACCTTTTCCTTACAATGAAGATGGGAATATTATTACTGCTCTAATCAG

AATTTGCATGAAGATATATTCTATCAAGATTAATATTGCTCTAATCAGGATTTTCTCTTATGACCTCTAC

AATATATAAACAAACACTATTTAGAGAGTTCACTTTGTATATACAATGTGAAGGTCACAAGAGACTTAAG

AATTTGTATCTTCGATTTGACATGGATTGATATGAGAGCAATATTAACCGCACTATTAATCTTTATAAAC

TATTATTCTAACCATGTCGTTGAACCTTCCCTCTATCGTTTTGGGAGGTTTCTAGAAGGCTTGAATGACT

GCGTTGAATCTTCCCTCTATTCCTTGTGGGGAGGTTTCTAGAAGGTGACTGATGTGACCTTGATAGTTTC

TTTACAAACTTTTATGTAACAATAAGATACCTAACTTTGTCAACAAAGGCAATGATTCAGTCAAATTGAT

TGACTACTATTTTTGCAACAATCGAAATTGCTGTCTCAAGAATATAGAATGAAATATGTTATTAAAACTA

AAAATTTAATTATATTCTTTGTGTGATTTTATTTCTTTTATAATTAAAGTAAGAGTTTTACTTTTTACTT

CAAAATTGTCATTAAACGTGGTACTATATCAATTAAAGTTGTATTAACGATCACTGTTAATCATATATAT

CAGTCCAGCATCCGTCAACGACCAATTCGTCATTGTCGTGATTGATGAACTACAAAACATAAGGGAGAAA

AACAACATTCTAGAACAAAATAAAGTTAGTTAGAACTCTCATTTGGGAAAGATTTCTAGAAGGCTTGCTT

AAATGATTGATGTCATTTTTGATGTTATTGTGTACTGAAACGTAC

>Ca_linc_0584

TGAATAAAAAAGAGTCAATTTTGGAGCAATACCTCACTTTGAGCATGAATAAATCCTATTTCATTTTCTA

TTAATCAAAAAATTATTCTCTCCTTATTTTTACTTGTCCACCATAATCTAGTTACAAGTTGCTCAATATT

GTCACATAATATTTTTGGGTGCCTAAACCAACTCATTAAGCAAATGGAGATGTTCTGAACAACAACTTTA

AATAGAATACCTCCTACATGTACAAGTAAGAACTTTTTCCTTTTCATCCTTTTAACTTCTCCCAAACCCT

ATCTATTACATATTTAAATATTTCTCTCATGATTTAACAACAAATGTGGGAAGACCAATTTATTCTGTTA

TTCTCTCACAGATTATTGAGAGTTTGAATTTGCTATGATTTATGAGTTGGCATCATTTTGTTTGATAAAA

ATTAGAAAATTACTTTTTCCTCAAGATTGACATTTAAAAATAAAATACCTCATTTACAAAAAGGAATGTA

AGAAATCTACAAAGTTGTTCTAACTATTTTTATTCTATGCAATTATCTCTTTGTCTTAATAATGAATATA

AGG

>Ca_linc_0585

CGTCCTTACAAATAAAAAATATTTCTATTTTTATTGATATAAAAGATAAATTAATTTTTGAAGTGAATTA

TCTAAAGTGACTAAGCCCTTCAACTCCATTGTCCCAATCAAGATCAGGATTATGCCAATTTGAAATAAGT

TTTTAAATTCTTTTAAAAAACAACAAGTCCTATAGAGTTGTGGCATGTGGAGGACGATATATAGTGTCTC

ATACTCTACACGCAATTCATTTGAATGATTGCCGTGGTGTACATACTACATAGTACATACACCTTTTGGA

TGCACCGTATAATAGGTCCACTATCAATCATTTTCAAGTGGATGCCAGGTGTATCACAAGTTTGACAAAT

ACTATTTAGTTTGGATTATATATATATTTAAAAAAAAATTAACTATTATATCTCACGCTTAAATTTATAT

ACTAAAATTTCCTATTGTTTACCTTCAAGAGGTCG

>Ca_linc_0586

TTCAAAAATTAGAAGGGTACACATAAGTTGACTTAACAGCAGGGCTAAAATTGTTGACATAAAATATTGT

AAGGACTAATAGTGGAAGAAATCTTTTTGAAGGACTAAAAACATATTTAACCCTAAATAATATAATCCTC

ATTATTTCATAGCAACTCCAATTGTCCTCTTCGTGGCAAGATGATGTAGCACATAATGGGAGTGAAAAAA

TGTCATTATCACACTTTAAGTCTTGGGTAAGCTTTTAAATGGAAATAAATTTATTGGTTAGTTTA

>Ca_linc_0587

CAATTACATATTGACAACTTTATCAAATAAAAAACAAAAAAGACAAGTTCTAAATATATTTAATCAGTTT

AAAGGCAAAAGTTATTGTGTTGCAGCTGTTTTAAGTCTGATTATGGTTGTGTTGCTGCTGTATAACCTAA

AAAAGTTTTCATTCTCCAAAGGCAAAGCTCTTGTTCTAATTTGGCAATGCACAACTGGTACTGTCTTGTG

ACCATATAAGTTCAATATACACATCAACATGACTAAGATGACACAAACTGAATATGTACCTTTTTACTTT

TGCTTCACTAAAACGACAATTTCTTTTGTTTAGATACATTGAAAATCAATTCAATTTAGTAATAATAGAA

AATCATATGACTTTATTAGTGTTAACAGAAAAGTAGAGTGTGTGGCAAATTACTAGTAAACCCCTTTGAT

ATAACAAGATACAGAAAAGTGGAGTGTATGGAAAATTACTAGTATATTCTGCATTGTGCCATGAGGGTTT

AACTGCATACTAAGATAAAATAAGCAAACAATTGATCCATCATCAGAGAGGAAAATTCATCTCATCCACA

ACATTAGTTGTGTAGGAAGGTGTCCTAAGATTTATTCCAATATCATCACACGATGTAAAGACATGCATAG

AGAAGTTATAAGGGAGACCAACAATAAAAGTCATTTCATATATGCTATCATACTATTTCAACATAAGAAA

ATAATAAAACATTTAATTTAATTCAACTCAAACAATTTAACCGAAAACCATATATTTTCATTAACAGAGT

CAAACAATAAACAACAACAAAAAATGTCAATTGATCAAAAAACTGAACAAAAGTGCACAAGATGTAGAAT

CAGGAGCATATAGATATATTTAGCATCAAAAAATCAGATCAAGTGTTGGATTATATATTTTGCGTCATCT

TTATCTACAATAGTAAAACACTAAAGGCAAATAATGGATCTGCTTTCTTCAAATTAAAGTCCAAACATAT

ATCTATATTTTCAAATAAAGAACCAGAGCTAAATACATTAGGATAACAGAAAATCATTCAAACATGAAAA

ATTTTGTTCCCCGACGTCCAAATGAATCATCAATTTGGTGCATCGGATAGCGAGGGGAACACAATTCTAG

GCTTCAATTGAACATTGCTAAATACATTAAAGTAGGTGAAAGCATGAATGATATTAACATATGAAGCCCT

TTAAAACATTATAATCAAAACTTAACCAAAAAAATCAACAAAAACCAGGATTTTTAAACATAAAAACAAT

TAGGAAAAAATGATTAAAAGATTAGTAGAATCACTAGATCACTCAGTCCCTTAGATGTTCAATAATAACT

GCGATCATATCCGCGAATCATTACAGGTCTGTAGACTCATCACTGTTAAAACTAGCAAAACCCCATTTAC

TCAATTGACTCAAAAACGAAAACCCATCAATAAAAAAATAAACTTTCAAGAGAAATTTATGATTTAGTTG

CTCTCGCGTTCTCCCAAAACCATAAAAAAAAGGGTTGTGGAAATGAATTGAAAGAACAACACATATTACT

ATTGCTTCAAGGGAACGAGTCAAGTGGGAATTGAAAATATCCTCATCGACCAGATAGTGAAGCCTACAAC

TTTACAAATTGCAAAATTAAACAGATGGTAAGAGCAAAATCCAAGAAAATTGGAAAACCCTAATTTCATA

AAGGGGCTAATAAATTGTCAAAAAATAAAATAAAAATAAAGGGGTTAAGAAGAAGAGAGGAGA

>Ca_linc_0588

GTAAAGTATCATGTATAGAGAGAGAGAGAATCATCCAGCAAGTATCATTTGAAGTGTTTAATACAAAACT

TTAAAAAGGGTATTTTTATTTTTCTAAATGACGATAATTAACAACCAATACTATCAATCAATCATTATTG

CACAAGCCAAAACCAGAAAACATGAAAGTGAAGCTACTCTTCTTAAAAACAGGACAATGAAACAAAAGAA

CCTATACATGAAAGTACTCTACTCATCCACATTTTGAGGTGTGCTAAAAGCTTCATCTTTATGGCTCATG

GAAATACAAGGGTCAGAAACGGTTAAGGAAAGGCAAACAATGATTGGAGTGAGGAAGAGAACAAAGCCAA

AGGGTACCATCCAAAGTTGCCTATTTGTTGGATGGTATTTGTGTAGCCACCATCCAAGCACACAACCTCC

AATTATGCTTACAACAAAGGATGTCAATAAAATTACAAAGTATGGAGGCTTTGTAATCCCATTTTCATCA

TTTGATCCCATCCAATTGCAGCTCTGCTCTCTCACAAAGAAATGATGGAAATGAAAACTAATTACAAACA

CTCATTTTGCTGAAAATAAGGGGCATGCAAAAATTGCCATATAGGAGGAGTAAGAGCACAACTAAACTTG

CCTACGTCTCTTTTAAAATGGGCGTG

>Ca_linc_0589

NNGAGATGGACCCTTGATCCAAAATGTGGCTTGCAATATGAATTTTTTACCCGTATGAAAAGAAATTGAG

ATAGTATTTTTGGATGACTTGAAATTTAATGAGAAGATAGAATCTTGACCTTAAATGTGACCTTCAATAT

GATTTTTTTATTAATATGAAATTGAGATAGTATTTTATGAGATAGATTCTTAACCATAAATGTGGTTTTC

AATATGAATTTTTTATTCATATGAAATGAAATTGAAAATTTGAGATAGTATGATTGGATGACCCAGAGTT

AAATTACGAGATGAATTCTTGATCCAAAATGTGGCTTGCAATTTAAATTTTTTATTCATANN

>Ca_linc_0590

AAACTATTTAACATTTAATGCGTAAGAGTGAGAAGGGGTTGTGGGGGGTTTTTTCTGGTATCCAAATCCT

CCCAAAACTTCAAACTTTTGAAGGAAGCCTCCATGAAAATATATAATAAATCATAATCACCAAGGAACAA

GATGTGGATGATTCAAATCATCAAAGGGTAAGCACAATATATTTTAATTATAAACATAAAACGTGGAAGA

AAAAAAAAATCGAAATCGCCATCAATTTTTTGTGACCCCACCCCAGATTGTTTTCTGAATCTTGCTGTTA

TTGATCCGAAACGGTAATCATTGGAAAGATACAAATGTTGGAGGAATGATTTATGTTTGTTGTTTGGAAG

CAAGGTTTGAACTTTGAGTTTGAGGTCTTGATTTTTTTTTTTTAAAAAAAGAAAAATTTATTCTAAGAAA

AAAATGAATG

>Ca_linc_0591

ATAATAATCAAATCATTTGGAGTGGAAATCAGAAACAAAATGATGAAGTGGACTTGGAGCTCAGACTTGG

CCTTGATCCATGATTAATTCATCATACTATATATTTCATCTTTTAATTAAGGTTTGATATGATGCACAGC

TAGAAGTGCATGAAATATTGTCAGATATTAACACTGAGGTACATTAATTAATTCCATTATTGTAGTTTAC

TAATTAATATCTTCATATATTTTGTAAGAACTAAGAAGAAAAAGTACTATAGTAGG

>Ca_linc_0592

CAGCATTTGACGGGCTAACAAAATCACCTAAACATATCAGTTTAGCAACAAGTTAGGGAGGGCAAAGATA

TTTATGTAACTTAGTAAGCAGCGACATTTATGGTTGAATATGCTTCATTGAATCACTAGTTACGGAGCTG

ATTAACTCTAATGTGAACCAAGTCAAATAAGCTGCCCTATCAGCTTTTATATAATAAAATTATCTTACCT

ATTGCGTTGCGTTGTGTTGCGCCGTCGATGGAGAAGTGTTGCGTTGCACCATCACCAGAGAGAGGCAGGA

AGCAGCACCGTCACCGGAGAGGGGGGCACGAAGAAGCGCCGTCACTGGAGAGAGGTAGGAAGCAGCGCCG

TCACCGGGGAGAGGCAGGAAGCAGTGTCATTCAATCTGGAAACT

>Ca_linc_0593

CAGCTTACAACTTAAAGGTTTTGTTTCTCCCATTCTTTTGCTACCACTTCATCATTTTCACTTTTTCTTC

TCAATGTTACCTAGACATTTGGAAACAACAAACAATCTAACACAAGAATCACTAAAAAAAATTCAAACAC

AAGTTCGTTCCATTTCCAATTGTAGGAGACTATGGGAGAAAAGGCTTGCATAAATTAAAAGAAAAAATGT

TGTTGCAATGCGGAATATAGGACAACATAAATATGTTTGTTGTTGATATTGAAAAAGAGTGACTGGTAGA

GAAAGTTGTAAGGGAGACCAGCGTTTTTTATGTGAGAGAGATATATGCGCCATGTGAACTATTTCTCAAA

GAATGAGAGAGTGGATATTGAGCTCGAACTTTTCTTCTATTTCTATTTTTTTTTTTCATTTGATAAACCC

AA

>Ca_linc_0594

GGGAAAAAGCACAGAGCCAAACCAGAAAAGGAAACAACAAAGCCATTCAACACATCGAACTCGAAATATC

AATAGAAAAAAAAAAAAAAAAAGAGTACAGAGATAGACAGTGGAAAATGGCGGAAGGAAGCTCCAGGACC

GATATCCAGAGGATACTCGCCGCCATTAAATCCTCCGAGGTTGTTGAGGACCGTGTTCAATTGTTTACTG

ATCTTGGAAATTTAAATTTGATCGAGGATGTATCTGACTCTGATTATGCTTCCGTCATGAATTGCCTCGT

AGTATC

>Ca_linc_0595

ACATACTCTACTAACTTTCCCTCACAAGTCCATATACATACATTAGGTGCTGTGTTACTACTAGCTAGCG

AGGTGCAATGGGTAAGGAAAAGGCTCAGGTGAATATTGTTGTGGTTGGTCATGTGGATTCTGAGAAGCCA

AATACTGAAGAACCCATAAACATGCCAATTGTTGTGGAGATCCAAGCTGATGAGATGATATAATTAAGAG

GTCGTTAATGACCTCAATGTGTTAAAAGGGAACCAGAAAATTAGTAATTAACAATGCTGTAAAAATAAAT

CTGGTACCCCTACTCAAACTTATGGTTATGTTTATTTTGCTTTCACTGAAATAAAATGCATGCCTCTTAA

TTTACCTCTTCTGCTATTTAAGAATTACTGAAATAGTTATCCT

>Ca_linc_0596

TGTGCACACTTGATATACTGCGGGATTCACAGATAGAAAAAGAGTTGAAACTCGGCGAATCGGTAAAAGA

AAAAGAGGTGCGAACTTGACGAATCGGAAAAAAGAAAAAGTTCGGAGCTTCTTGATTGGATTTCGATTAT

TCTGAATTCGTTCTAGTTCAGTCGCTGATTGATTTCGATTGTTCTTATGAAACCGGTTAAAGAAAAAGAG

AGGTGTGTCTTCTCTACTCTTCATTTTCAATCTTCTGCACTTTGAAACCAAATATTTTTAACGGTAACAT

CTCGTTCTTGAAATCAACCAAGAACGTTCAATCTCGTCGAATGATGATTATTCAAACCCTAGAAATATGG

TTTTAATTGATGCTGGAAAAAGATGACTAGTCACAAACTTTGATATTCGGATTCTAGTTTCTTCAATTCT

AGGGTTTGAATTATCAACATTCATGGTTTGCTTCGATTCGAAATAAAAAGCTTCTTGATTGGACCAACCT

TCAATTAATCTGTAGTCCTTTAAAGTATGAATTTCCTTTCTCTTATCACTGCTTCATTCTCCAAAACCGT

GTTTGGGTTTTGGGGTAATTTTTAGTTTGCAACATTAACAGATTATACTTGAGTATCAAAGAATGTATAA

TCTCCCTTGATTATTCATGCCTAAGAAGGCTGATAAATATTGAAAAGCTGCAATGTGTATCATAATTTAA

TTTAATCGCGAGATGGATTCTTGACCCAAAATGTGGCTTGCGATATCAAATTTTTATTCAATCATAGTAA

GTTTGGATGACCCGGATTTTAATCATGAGATGGATTCTTGACCCAAAGTGTGGCTTGCAATATGAATTAT

TTGTTCATATGAAATTGGGATAATATGATTGGATAATCCTAAATTTAATCACAAGATGGATTATTGATCC

AAAATATGACTTGCAA

>Ca_linc_0597

NTCATTGAGCAGCGTTAGGGTTTGCAGAGTTGTTCTTTCCCTCCATCTCCCATCGTAGTGTCATTATTTT

CTGCTAAAACACCTCACACTTTTGCAATTCACCTCGTAGATTCGTTTTTGCAACTCACCAAATTTACCGT

ACCGTGCTGGGCCTCGTTGCGAACCTTTTTCATTCTAACTAAATTGGAACTTATGGGTCACGAAGAAGAG

AATAATAGTAGCAGTGTGAAGAAAATAATTTCACCATGCGATGTTGAGGCTTTAAAAAAGTGTTTGGAAG

AGAACAAGGGTGACTATGTTAAATGCCAGTCACAAATTGAGGCTTTTAAATCATCATGCTCACTCAAGAA

ACCAAATCATTCTCCTGAAGGGAAATAAAAATTTATGAAATTTGAGCCTCTTTAAAGGTATTATAAAGTT

GTATTAATTTGTAACAAAATGAAATGTTCTCGAGTTGTAGCTTTTTAAGGATAATAAATTTTACGAAAGA

GTTTCGATGATACTAGTGATTGTTAACGGTTAAGAATGAGAGTCAACACTATGCTGAAGAGTAGTGATGT

TTTTGCCACAATTTTGTATGGAGTTTCCCGCTGATTTACTCTGTCTAGGTTTGTGTCCGTGCAATGAAAT

TATGATATTTCCTGTGTTTATCTAATTGTTTAATTGAGTATTAACGTGGTTGAACTGTTTTTGCTCCTCT

TTTAGTTTCTTTGTACATTATCTTTTTCCTGGCTTATCTTAATCTAGTTATGTCTGCAGTCTGCATTTTG

AGGATCCTAGGC

>Ca_linc_0598

AGAGTGAGTCGGTCCACAATGAAAATAAGTTATTCGTGAAAATTGATAAGGAACTCGATTCTCTTCTTTC

TTTTTTCTTCCTCTCCACTAAAATTGTGTTTCACGACTTTATTTCTTCTTCTTTTCTCTCGCCGTTGATC

ATCGACCTGAAAACGCTGTCGTTCGCTCCCACGTTTCGATCTTCTCATTGTTTCTCTCTTTCTCACGCGC

CTCAATCTCTGCCCAAGGAAAATAAAAGTGGACACAGCGACCTTGGAAATTACTCAGTCAAGATCTCATA

AACGTTTGTCTCTCCAATATATTGGACTGTAAAGGAACCTAGTACTGTTTTGCCAATCATGGACAGGCGC

AGGAGCACCACCCGACCAGTAACCTTATCTTCAGGTTCTGCTACTGCAACCACCACCATAACAATTGAAA

GCTCAGATAGATC

>Ca_linc_0599

AGAAAAAGAAGATGATGTACCACATGACTCTGATAAACATCCCCATGATTGTTGCAAGAATCACGAAGAA

GCTAGCCCGAGCAGTTAGGCTATGTTTGGCCGAGGGTAGATTTGATGTATTATCAGTAGTATAAGAGAAA

GAGAAGAAATAGCAACAATAAAGCGTGTTCTCTCTTTTCTCTTGTTTGGTTGAATAGAAAATGAAAAGAG

AGAAATGTTTTTTTATTTTTAGGACAATAATAC

>Ca_linc_0600

CGGAACCGTAACATATCAGAAAAAATGAAGAGAGAAAAAAAAGAGAGAAAAGATGAAAAAGAAGAGAGAA

AAAAAAAAAACAAGAGAGAACACACTAACTCCATTGCATGTCCTTCTCAAGCTCCCTCATTTCATCATCT

CATCGTAGAAACACCCTCACACCTATATTAGAGTAAAAGAGGACCAAGAGGTAGAGTGAAATGAGAAATT

ATGAGAAAGAAGAAAAGGAAAGTTAAGGTTTCAATATAGGAGAAAAAGTTTAGAATTTTCTGCATGTTAA

GAAGGAAGATGGAAACGTGAAGAGCAGTTAAAGTTTCACTTGTAGAAGAAGGAATTAGAATCAAACTTTG

GAGGTCTCCAATCTATTCTTAAGGTAAACATTGAAAGTCTGAGGAGTATCCAGATTTGTTCACCAGTCCT

TCAATGTCATGAGTCAGAACCGGATCACGTCTTGAGTCTAGATTAGAGTCATAGTTTAATTAAGTCTCAC

TTAAAAGTTGTTTTAAAT

>Ca_linc_0601

CTCTCATATGTTGTCGGTACTATGGTGGGTCCAAGTAGTGGTCAAATAATATATGGGCTTGTTCAGTGTA

CACTTGATTTGTCTGGCCACAGTATGAAGGATGTTTGATTCAATCTATTATTGAAGTGTCAAGATGTTGT

AGCAATAATATAGGTGCTAGAATTATTAGATCAAGTTGTAATTTGAAATTTGAAACTTCGTACCAATTCT

ATCAACCTCCAGCACCACCGGCTTCACATCTCTGATGATCATCCACTACACTTTGTTGGAAGATAAAGGA

AGCACATCTAGAAGACAAGAAATGTAATTGCTATAGTCCTGCTGATGCTGCTTTTGTTATTGTGCATATT

TTTATCTTGAGTCTATTTGAGAATAAGGAAAACCAAGGAAACTTTGAAAGTATTTGTAATAAGCACAGTT

GCTTCATTAGGAGAATCTTGTTGAATATGCTGCAACGACTTCATTAGTTGAGTATATTATACATAGTGCT

CGAT

>Ca_linc_0602

GTCAAATCCTACAGCCTGGCAACAGAGGTTCAACGCAAGTTGGCATAAAGCAAATGGGTTTGAAGACATG

GCACGTAAAAAAAAATTTCTGGTTGGATCACTTTTCTTTAAAAGGTATTCTTTGGGTTCGTCTGAATGGG

GCTTGCCAACACATTCCGACTAAACCTTCTCCACTACTGTTATGTGGGAAGTGGCATTCTTTCCTTCTCA

TGGTCGCTCTCTGGGCTAAGATTTAGTGAACCTTTTGGGGGCTTGGCTTGGTCTTCTCTTATTCAAATGG

AAAAAATCGAGTTTTGTGCTATCAAGAATAAGGAATAGCCTTTATTTTAGCTACATCAGTGGCAAGGATT

GCCTCTGAAGCTGAGCATCATAATAGAAAACAAAAAACCCCTTTTAAACTTTATTAACACAGTAGAATGC

TCTCAACCAAAAAAACAAGACATCCACACAAATAAAGCAAAACTCCATCTTTCAAATAAAACAACTCTAG

TTTCATATTTTAACTTTCAATCTGATAATATAAAGTTTGAAAAAGCTGTAAATATGAAGATAATGAGAAG

ATACATGCCTTTTCAGATACCGGTGAAGACAAAGACGGTGGTGTGCGGTGGAAGTTAGAAAGACGGTGAT

GCCTTCTTTTTCTTCTTCTCTTTCTTTTTATGTGTTTTTATCAGTATTAATCTCCGATTTTCTTTCATTT

CTTTGAAATTTGATAAGGTGTATTTATAGAGTTTTTTTTATCTGTTTAGTTGCTTTCTTTGCCTGCCTTG

GTCCCTCTCTATCAATTTCTTGTTTGATGTTTCATTTCCTTTGCTCATCATCTGCGTTTTTTTCTACACC

TCTGATTCATTCATGGTTTTGACTTATTTGTTTTGATCTGTTCTGTTGCTGAGCTTTAATTTGTCCCTTA

GTTTATTTTTTAAACTCAAAATTTTGTCTTTTTGTTCAGCACAATGTGTACCAAGACTGATTTTAAAAAA

TTATATTTTTTTATGTTTATCAAAATAGTTACAGCAAAGGTCAATAACACTGCATCTGAAACAGAAAGGC

ACCAACAAAACAGAGCCAAACACTGCATCAGAAACATAGAGGCAACAACAGCAACAAAACAGAGCCAAGG

CTTAATTTGAAAAAGTTTGACCTAATTTTTATTGTAATTTAATTTGGCTTTATTGTTTGTTGCAATTTGA

AATTGTAATTTGATTTAAATTTAGAATTGTAA

>Ca_linc_0603

NCCTTTTTATGAAAACAGATTTCATATTGCAATGATTCAGTCTTTCAGGTTTTGTATAACCTGTTAAATT

TTGATTTACAAAATGGAAGGTTTTCTGGTTTTTTAAGCAGAAGATTGAAGGTCTTGAATATATCAAGAAC

CATAAAGTGAGTAGCCCAATATCGTTCCTAATAGGTATCAAAAAAACCAGACATACAAAGTTGTTTGAAA

ATTCTAAAATGGTGCAAAACTCAATTTAAAAATCATTTTGAAATATTTGGGCCAAAGAGTGTCTTGGGAC

AGAACACATTTGCTCAGTTAAGGTTCTTTATTTTTTCATGCAGAAGATTGAATGTTCTTCGACGAACAAA

AATGAGAAAAATAGTTTTCCAATAGGCATCGATGCAAATATGATGGAACAAAAATTCCAATTTTACAGTA

GCAACATTCATGCAGGTTAGAGCTACGGACCATGAATTTCACCGCTTTTATGGAAAAAAATCCCACCTTT

CAGTGATTCACGGTCTTTAAGGGTTTGTATACCCTAATAAATTTTGATTAAAAAATGAAAGGGTCTTTTT

TTTTCTCAAGCAAAAGATTGAATATTCTTGAATAATCAAGAACGGGAATGAATATTCCAAAATCATGTAT

AACTCCTTGATGGTAAAAAAAAAAATTCTTTCAAAATCTTGGTACCAAATTGGATTTTGGGACATAACAT

GTTTCCTCAATTAAGGTTATTTTCGCAGAAGATTGAATGTTTTGGGACAAAAAATGTTTCCTCAATCAAG

GTTCTTTTCTCAAGCAGAAGATCGAATGATCGAATGTTCTTAGACTAATGAAGAACGAGAAGAATAACGA

GAAAAAGAACCTACCAAGCAGAAGATTGAATGTTTTGGGACGGAACACGTTATAAGCAGAAGATTGAATT

TACTTGAATAATCAAGAAGGAGAAAAGAAACACGACAACGAAACCAAACGAAGTGGCAACATAG

>Ca_linc_0604

CAGGGCACCAAGTTTGGGATACCAAATTGTCAGGACCAGAGAGTATTGTCTGAGCTTGTTGTGAGATAAT

GAACTAACAGTGGAGGTAAATTATGTGATACATTTAAAAAGTCCTTCTCTACACATTCCAATAGTAAGGC

TATTGTCAAATAATGAGGGATATCTATGAAGCACCGACACTGGCACATTGACATCGATTATATTTTTGAA

AAAATGAAAAAGTGATTGAACTCAACAATATATGTCGATGTTGTGTCGGTGTCTGACACTGACGTGTGTC

CGATACTAGACACGTCATTAATTTGAAGTGTCGGTGCTACCTACTAAATACTATCTTATTACTTAAATTT

ATGGGTTTCTTTGTTTTTCGAGAAAAAGACAATTCCAGGCTATCATTAGTGTTGAATGAACTGTGATTTG

GAAGAAAAATCTAGAGAAAAGGATTGGAGAGGATGTATTGCATTGGTTTGGATTACAAGAAATATGGCCT

TGTCAAAAATATTGCTGAAGTAGCATCCAGTTCTTTTTCCAGTCTAGTTGGTTCAAAATGGAATTCCTCC

TAGGTGCATGTTTCAAAAGTTCTGTACTTGTAAGTCATTTTGAATTTTGAGTTCAATGTATGTTAATCCA

AACAAACAATTTAACATTCTTTTCTCTATGCTTGTAAGTAATTTAACCTTCAAG

>Ca_linc_0605

CTTTCCTTGCATGGCTGATTATGTAACGCATTGGATGAAGTCAAGAGGCCAACGCACTATGCGGACTCAA

ACATTGCAATTCCCCTTTCAGCGAGATGTCTTGCAACTTAGAAGAAAGACGATTGATATGTAAAGCCATG

CAAATGACGTGCAAAGGCGAGTTGTGCACAAATCGGGAATTGTAAATCATTTTGCATTCATCAAGGCTAT

TTGCAATGGGAATGTCGTCCAAAGGCAAATAATCTTTTAACTTGCTGCTATTACAAATTTTGATGATAAG

CAACCTGCTGATGATGTGCCAGTCTTGTAGAAATTGAATTGCACGATTGCAAACCTGCTCACTTTGTTAA

TTTACATGATTCCATAACTTGTTGTTATGACATTTACAAATTATCCAAATAATCGACGCCAAGAGGGCAA

CATGTTGCTATTAATTTCTATTAATGTTGTTTAGAATATTTCTTTTGATTGGTGGTTTCTTTCTTGC

>Ca_linc_0606

TGGGTAAGATGGAGCAAAAAGCTTTTTGAAGGAGTTTGCTATTGTTTCCACTAATGATGATTTACATCCT

ACCATATATTAACATCAACTTCTATTGTTGTGGCATTTGATCAAAATTGACAAAAGAAAATCTATATGTC

ATGTTGGAGTTTCACTGCTATGAGAACATAACAAAGTTGAAGCAAATTTCTCACTACTCTTGGGGTATTT

TACATTTTTACCAATTGATGTTATTCTGTTATGCAACTATCATTGGTTCTTTCTAATATGAACGTTGGTC

TTATTATGTTCTACAACTTTTTTTTCTTTCTTGTAATTTGTATTATGTGTGTGTAGAAGAAGAAATATTC

ATGTTGCTGAAATCACCCAGTAAATATTCATGATGTAGCAATATTGTCTTCCTTTGATTAATGTCGAATT

TTGATTCAGTTAATCATAATGTAATTTATACTTGAGAATGTAATTTGTATTGGTTGTGTTATCATGTGAC

TGTGACTTATGTTGCTGATGAC

>Ca_linc_0607

TGGTAGAGTAGAATTTAATTTATTAATCCAATTCACCGTATATTTTACACGTCTCTAATTCAATTTAACC

ATGTTGCTAAAGCTACTAGGACTCTCATGTGTAAAATTCAATGCATTGTGATGAAATTCTGGTAAAAACT

TTCGTATAGATTGACATCGTCACTTTCCTTTGTAATAACGTTGTTTACTCTTTGCTATTATTTTTGCTTC

TATTTTTTGAAGTTTCACACTTCTTGTGGTTTTATCATATTATTAGGTTGGCAATTAATGAATTTTTACT

GCGTTGGTTGCAGAAAAAGTCAAGTTTTCTCAAGATGTGGATGCACATGTTTGTGATTGTGTCGAGGTAA

ATTCATCATAAACAAAAGTGTTGATTCTTTCATATTGTATTTTGCTCTCATGTATTTTAATTTGACTATG

TATTGAGAACATGTTGGCAATTATGGCACCATTATTGATTTAGACAGGTGAACAATTTGAACATTATATG

ACAATCTACATAGTTCATAATGTATATAGTTTAGATCCAAAAGAGATGTAGTGAAATATGTATATAAATT

AGTGTAATTTTACTTGATGTTGGCTAATTGAAAATTCAATTTGGTCACCTAATCTAGAACTGAACGTGCA

AACAAGTTTTTGGTAACTTGAAAATTCTCCATTTGTTTATCTCTATGATTTTCATATTTATTCCTCTGGT

AGAATGTTCTTTAATTGCAACTCTTGGCAACTCTCATTCTTAATGCATTTGTTCTTGGTAACTTGTGAAT

CCCTCTTCTGCCCTGTACAACAGCTCTGTCCCTGCTACACAGAGGCTCTGCCATGGGCCTTTTGCATATG

TTTTTCCTGCTTTAGTCAGACTATATATATACACACACTTTACAATGCTTATATTTTTCATGTTCTTTTG

CACATTGTTAACTTAAAATAGTGTTGTATTTTTATCTTGTCATGATTAAATGTTTTATGTGACCCTAATA

TAATTGTAATAGACAATATATATGTTTTATGTTGTTTCTGGATTTTATTTTCACTA

>Ca_linc_0608

CTAAACTTCAATTTGTATGAAATCGAACTCAATCTCATTTTCTTCACTCAAATCCATACTAAAATATCAT

TTTTTGACTTATCAATTTTTAATCATCAAAGCATGGAAGATGACGGTGATCTTATCCGATTTCGACATGG

TAGGGAGACGCAATATGGGTATGTACATAGAGAGCTTCAGACCAAAGCTCATGCTAAACGACAATGGGTT

AAAGGAACGAATTCCTCTTATATGCGTCAACAATGGTATAAATATATCTCAACTAGAGGTTCCACCTTCT

ACTATAGATTGGTTTTAGAATCCAATTAAAGCTTCTAGTTTAGCTAATCTTGGACAAAAATGACGAGTTA

ATAGACATCGACATTTTGTTCGCATTTGTTGAGAGGTATCGCACATAGACTAGCGCATTCCATCTTTCTA

TTGGTGTGATGCAATGACTCTGAACGATGTGTCATGTCTTCTGCACTTGCTCATCACTGGACACCTCTTG

GCACGTACACTACTGGTGAAAGATTAAAGGATTGAGTGCTTAATGATCTCGAATTCAACTTAGATGATGC

GCCGACAGAGGTTCAAATGACCAAATAAGCTCATGTCAAATTATGAACCATTAAAGCACATTTTGTGAGT

CATCTGAACAAATCAGTTCAGTTTTCTGTTGATGACGAGGTGAGAGAATATCATAGACTTTATGACATCA

AATGTTATCATCTCTACTTGGTGGGAACTGCCATATTCAATGACAAAAGTGAAATGTACGTCGATATTAT

GTATGTGCAATACTTCAAGACTTGGCTATTGTCCGAGACTACGCATATGATGGTGTTGCTCTTGCTTTCT

TGTACATTCAACTAAGAGGTGTTAGAAAGCCGAATATCAAACAATTAGTTGGTTGTTTGTCCTTT

>Ca_linc_0609

GCTTTTTCTTGGCCAAACAAAAGCACAAGTGTTAGTGTTTGCAAGAAAAAAAACTTCATTTTTCTACTAA

ATCAAAGTTTCATTTTAAGAAGTGTTAGAAAGAAAAGTTGCTTTGATCTACAAGGTGGTGCTTGTGAGCT

AATTTATTTGAGATGATACTCGAATTCGTTCTACCGACTATTGCCCTGACGAAGGAAATGTCATTAGACT

TCCCTGACTGACACTCGTCGACTGCTTAGGCATTCAATCCCATCTCAAGTAGGGCTACATATATGGGTCG

AGTCAGAGCTCTCGCTTTCGGTAGTGAGCTGTTTGGATTTTCGTGCGCCGCTGAAGCCACTTATCTCGTG

GGTGGACCTGGCTTTTGAGGAGACTGGTCCTTCTATGGCTAATGAGGAAGATGAAGTTACTTCGAGGAGA

GATACTACGTCATGAGGAATTTGTGCAGTGTGTGGAGATTACCCTTGTTACTGTAGTTATTAGTGGATGA

TCAGATAATAGGGTCTTAGTGTTGTTCTTTTGGTGGTTGTACTTATTTGGTTCTGGGATAACTATATCTA

TGACATAACTTGTCAGTTGACTTTTTGGTGGGTCCATGTTCCATTTTTTTGACGTTTTGGGGGATGACAT

TCTTGGAGCAGTACTTACTTATGTGCAAGTGTATGCCGAGAATACCCCAGGGGTACGAGCGATGTCTAAT

AGGTCTCATAGCCCAGATGTATTTTCTATTTGTATATTTTGGATTATTGTCCAATAAACTATCTTAGGTT

GTTAGTATTATATATGATGTAATTATTTATAACTCTTTTCGTTTTGTG

>Ca_linc_0610

AAGCAATTGGTGTGGGCAAGGGCATGGGAAAGCGAGTGTCAAAGGCTCAAGTTGTTGCTACGTGGGAACA

AATGAATAAAGGAGTATCTAATAGAGCTATCATCAGTTTTACAAGCAAGCCTAAATCTACTATAATACTC

CAAAGAATGATACCTTGTTTCTTATTATTAACAGAGAAGTCCATAGGATCCATGGTAAGGATGAAACCAC

TGGTGCCATGGTGATCAAGCTATCAACATTTGGTGTGACATGCTAGGAAAAGTGGAAGAACACTAGGGCA

TTTGAATTTGAGGTACCCAACTTAGTGGTAAATTTCTGGTA

>Ca_linc_0611

CTCACACCTGCACCCACCCGACATAAATGCAATTACTTAAATATCATTATATATCTTTAAATAGTAAAAA

AATCCTAATTTTCCTTCTTTTTTCTTCGTGTGTTGTTGCCGTCGTCCACCTCCTCCACCCTCATGTTATC

TGTTCCAGTCGTCCTCCCTTCTCTCCGTCCACCGTCTTCGTCATCCTTCGTGTGTTGTCTCTGTTCTCTT

ATTCTTTTTGCCGCTTTAATTTTAAGCTTTTGAATCTGTATTCAGTACTGGCGGGCATATCTTAAGTCCT

CGTCGAAGTCGACTTCATTGGACTACTTTAGAGGTCTTGATATGTGCTAGAAGTTGGCTATGTAGTGCTC

A

>Ca_linc_0612

TTTTGTAGGCAAAAGCAATCCAGAATGAAGACAAAATCCACCAAGCAGAACTGTGTTGTGCACTTGTATA

TTTCTAATCCTGAAACAATCCCCAATTGTTTACACGAAGAAGCATGATGACAACATCAAACAAACAAATG

ATACCAAACCCATCATATTCAATAATTCACAAAAATCAAATACTTAAAATCACAGTTGAGAGCAACAAAT

CAAGGCTGAACACTATGAAGGATGTTACTGTTATAATACCCTACGCTGCTGAAAAACAGGTTAACAATAC

AATGTGTATGACTGGTGTTAAAACCATAGACAGAAAAATAAAAGTAATATAGAATAAATTAGATTAATCC

AAAACCATTTGCCATTCTTAGCCTTCCAAATCAAGAATCTTAAAACACTCTTACACTTGAGCACAAGTAA

TTCTTAGCCTTTTGAGTGATAGTTGATAAGTAAAAAGTGATAACTAATAAGTGATAAGTGATAATTGATA

GTGGATAAGTGATAAGTATTATCAAGTAATAACTGATAAAGAGGCAAGACAATAAACACACACAAACTGA

TGATACCAAACCCGTTATATTCAATAATTCACAAAAAATCAGACTTCAAAATCGTGAAATGGAAGAGAAA

TATGTCAAAGAACCAAGAATTCAAACTCCAGGAAAGTGTCGCCATAATTGTGCATGAAGCTAGATTTATC

ATGTAATGTTGCTCTCAAACACTTAACTGCACAAAAACAAGTGCACAATTTAATTCATATGTGCAAACAT

TCAAGAAAGAAAAAAGACAAACAAACTCGTCAAAGTAAAATAATCCTAAGCTGTTCATGTTATTGTCTGA

TAAAATCCCTTCAAAATTCCAACTTCAATTGTGATGATTGTAGACTGCAGTGCTCAATATATAAAAGTGA

CAATTTAACCAAACTCCAAAGGCAGGTCTCATGTACCACTGTAACCAAGATGATATTTAATTAGGAGAGA

AGAGATCATTCATAGATCAACACCATCAAAAACTTCCGATGAAAAGTAAAAGAGTTGCAAATTTATCCAA

ACCAAGAGGCAGGAATAAAGAGCCCAGCCATGACCGTCCAAAATCCCCCATCCTGCCATAGGAAATATAT

ATAACTCTACAGAAAAACACAACGACCACAACATTCAGTTCTCACTGTCAAGAAGCCAAATCTTAAAAAT

ATTCATAAATAACAAAAACAAATAAAAAGAGTGATTTTATTCCCATGGTTGGAGACCAAGATTGTTATTC

TTCAAAAGCTACCACATCCCACCAACATTCATACCCTGTAACACCCATGAAGAAATCACAAAAGGGAAGC

AAACGACAAAAACTAATCAATCAGAATTGAAAGATTGAAAAACAAGAAGTCCATCCATAAATCAAATAGA

GATGAATAAATTGAACAAAAAGAAACGTTGTCTTGATTGCATTTAAACCTAAAGCAGAATGGGTCTCCAC

CATAGCTTCCATGTGTGACCAAGAGTTCATAGATTACCTCTCCTAGAACACCAAAGGAAAAATCACAAAG

TCCATAAAACTAGACACTTAGAACCGACAAAGAGTATAAACGAAAAGAACTCATGATATTCCGTGTAGAG

ATCAGCAAATCAAACTCAAGGCGGTGGCGGTGGAGCTGGCTCAACACTGTGCCGTTATTATTTTGATGCA

CCACGTGCTGCAGAAAAACAGGGTAACAATCAACGTTAATGTCTTGTGTTGTGACCATAGCCAGAAAAAA

TAAGTGATACAGAAGACTGATGACGAAACCCTAACAATAAGCCGTGTTTAATCCTTTGAATCAAGAATTG

TAAAACTTCTATGATCCGATCTTGAATAGGCAACACCTTGGAACAGAAAACTGATAAAGAAACCCTAACA

ATTAAGCAATAATTTCGAAAGAATAATATCAAAACAATCGAAAGAAGAAGTGTCGATTTCTTAGAGTCTT

CGTCATATCATAATCAAACCCCCAATTAAGATGTATCAGATGACAAAAATAAGAATCTAAAAATCTGAAT

TCCATATAGAAAGGAAACAATCGGGTTACAAGTATCAAGAACAAGACCAGCAAAGTTCGGAATCATGTAA

TCGATATCAACAATCACCAGAATGCAAGAATAGAGAATGAAGAACAAACGAGAGAAAGATTATAAGAAAC

CAAAACACCGCGACCAGCTACATTGAGAATGAGGG

>Ca_linc_0613

TAAAATTTGGATGGTGTTCTTGAGACATAATGGCCCTTTCAAAATTTTTGATGGTATAATTGGAAGTTAT

TGACCAGAAATGAAATCAATAATCATTATGAATTTTGTACTTTTAGTCTTAAATGTGGATAATGTTCTTG

AGACTTGATGCCCCTAAGAGGATTCACAAAAAAGAAAATAATACTCATTTTGATGAAGAGAAAAAGTAGT

GAATCAGACTTCCCCTAATGCAAAACAAATCCTAAAGAGAAAAATATTAAGATATAAAACTTAAGAACCC

CTTTGAAAAATTGAGATAATATAATTGACAGCTAAATGTAATTCATAAATGAATTTTGTAGCTAAATGTA

ATTCATAAATGAATTTTGTAATTCATGAACGTTGTTGGTGATCTTGAGACTTGATGAACCTTGCTGCTAT

AACAATATGGGTCTTTTATCTTGAGACTTGATGAACCTTGCTGCTATAACAATATGAATCTTTTATCTTT

TTGTGAAATAAGGTGGTATATGTGACAATTATTGGCCTTAAACGCATTTACTAATGAAGTTTTATTCATC

ATCATAGCATGAACTATGGATAATGTTCTTGAAATGCCCTTGTTGCAGTGACAATATAAATCTTTATTGT

TAATGTGAAATTGGATGGTATTCACTGGGCCTTGATTAGTCATGGATTCTGGTTGCAAATATATTTGAGA

TGGGAAGCTATAACATCGATAATCCTGAATTCAATTACAAGATAAATTGTATAAAATTGGGACGGTATGC

TTGACGGGACATGTCCCTAAATGCGATCACTATATGGATTTTGTATTCATAATGGGAAGTATACAATGTT

CTTGAGAATTGATGGACCTTTTTGCGATAACTATATGAAATGAATCTTTATCTTTGATGAAAAATAGTAT

GGGGTGCCTAGTTCTTTGATGAGTCGGTGATTCTAATTGCAAGTATTATATTTGAGATGGTATGGTTGAG

CATTGATGACCCTAATTCGATTGCTCTATGAATTATATATCGGAATGAAATATTTAAGCCTTGTGAACGC

TAAATATATGAAAATCGAAGACCAAGAACAATATCATATTTTCTTTGAGACAACTCAAAAATTGAAAATC

TATATGGAATGCTTGAATTGATTTTATCATAGATAGAATATTTAACACTGGATCCATATTGAAAGTTGTC

ACCATCACTTCTCGTGTCTGTCGCAATCTGTGTTCAAAATTGTCTATACTATGTCCATTGATATAGCAGT

AACTATGAGGTGAGAAGGTAGAGCAATAATTGTGGGATATTGAGCTTTATATAATTCTGCAAATGAAGAT

GGGTTGTTGCCAATTGATACTAAGTTTTTTCTCCTAGCTTGATGATGCAGATTTAAGGGTGTGCCTAATG

GTTTTAGGAGATTGCTGTTTGATCATAACCAATTGCGAACTTTAGA

>Ca_linc_0614

AAACATTCATTATTTATAGCTACTACAAACTAAAGCAATTTTGCAGCTCCTGGTATTAGTCCCCACATCG

AACACTTTCACAAAATTCTCCTTCAATTTTGTAACACCCCTCTTTACCTTAAATAATTCATAGTATTCAA

TTAATGTTTTTACACGAGTATAAAATAATAATTTGTGAAAAGCCCCTGTCCTCCACTTACACACACAAAA

GACACTTACTCTCTTACACTTTAGGGAGAAAAGAAAACAAAAACGCTTTTTCACAAAAACAAAGAAAAGA

AGCATAGAAGGAAAGAAAATGGGCACATATGAGGGAAAGGAGAAGAAGAAAGGAAAAAGCTTGTCTAAAT

TAAAGGGAAGATGAGAAAGATTAGGAGTAAAATACTTCTTGTTAGAGGCTAAGGAAGAAAAGGCACAAAA

TCACCATCTTCTTCATCTCCATTTTTCTGAAAATAATCTCAAAGATGCCTAGCACTGTACAAAGACGCAT

GACGTCTTGCGAGTCACATATCTAGCGCCTAGCGCACTGGCTCTAGGCGCCTAACGCTATCTAGGATGCT

TCCAAGGGGCCCTTGGTGCAAGAGGGGCTGTCATAATTGATGTTTTTGACCTCATTTGATGTATACTTCT

ATTTCTATTCTTTTTTCGTGTACTATTAATGTAATTACATTCATG

>Ca_linc_0615

ATGAAATACAGAAATTTATTTTGCTGGTCCTTGCATTGCATTGCATTCGGTTTCTTCTTTTCATTGAGCA

GCAAAGCAGATAGATATGACCTCGCCGACGCATTATCGAAATTCATAACTATTAATTCCTTTTTCTGATA

ATAATCAATCAATTAGGTATTCGTATCAAAACATATTCAAATCCCGTAATTCACATAAACACAAAAATAA

CAACTTGTAGTATCAGAAACAAGAAGTTGGAGTCGCACCACTTAACTCAACTGCCCGGAAAACGGAGCCG

GAGTTGCTCGTCGGCTGCCAGAAACGGTCATGGCTGCTAAGAAGACAATTGCAATTTGTCAGTCAGGTGG

CAAATTTGAAACTGAAAAAGATGGTACTTTGTCATATAAAGGCGGTGATGCTCAT

>Ca_linc_0616

TTACTTGAAGAATTTTTTAAGTTTAAATGTCTTTTACTTTTATTTTTTGTTTTTGATTTGTTTTTGGACA

TTGTGTAAATGAAGTTTTTTATTGGGATTAGAGGCACACATGTGGGGAATATAGTATTCACAAATGCTAA

TGGTGATGTGTACAAAAAAGATAAAATGAAAAGGAGTATGAGTTAATATGGTAGAGTTCAA

>Ca_linc_0617

GTGTAATGCTAAGTAGTAATTGGCTACATATTCTGAACCCAATTTCTGAATTTGATAATTTCTAGCTGTT

AAGGGTTTAAATGTATTATTAGCATGAATATCGCATACAAGCATCCTTAGCAGCAGCCAAACACCTTAAT

AACACCCCTCCAACCACAAACAAAAACACCCCATAGCAATTCCCTACAAATTACAATCACATTATTAACT

TCCAGTTGCTTAAGACATCACAATGCAGCGAAATTTTCCAGGCCATACAGTCTGAGACCAGATTTGGCCT

AACCACCTGAGCAACACACGACAGAAAACACAGCACCAGGTACGGATTCATTGCTAACTCAGAACCAAAA

TCAGCAGCAAGCCTTATCCTCTGTATCCAAAGCTGGCTCCACCGCTGCAGTCATGACCTGTGACACCGAC

CCATTAACCTTTTATATTAATTACAACATCAGCTCCATATAGCTGCACAGTCTCACGTAGCTGTCTCTTC

TGCATGTTAGCCTAAGCCGTTGTTAAAATTGATCAAGTGTTTGATAAGAAAGCACTGTTGTGTGGGAAAC

AAGTAGAATATAGCATTGCTATATCCATTACCAAATGCTATCTAAAACAGAAGATATAGCATTGCTATTA

TGCTATATACCCTGTGAAAAGTTGTTTTGTTCCGTATGTGATTGATCAGGTACCAGCAGGTGGCGAGCTC

ATTTTTTTGCTGGTTTAGTCAGTCTATTATCATGACAGTTATAACAAAACTTTGATCTGTGTTGGTGGGG

TTATTAAGAATTGGTGGATGGAGGTGTCTTTAGTCTACTTGTTATATTTTTTAATATATAGCCACTAGTA

GAAAACACAACATTGGATGTTGGATGTTTTTGAATATATAGTTTCTATTTTTAAAGCCTTGATCATGAAG

AGGACATATTATAGTATTAGCACCCTTTTATGATTTTTTTTGCATGAAATTAATCAACTGTTAAGTAATT

GGTATTTGGATATTTGCAGAAGATGCTGTAAGGTATTGTTGGCAATATTATAGAAGTAGAATTAGATGAG

ACCAGGTGAAGAAAGAGAAGGATAACTCTAGCCTTCTATCTTCTAACAACTCAGCTGATTAGATGCAAGT

CTTGAATTTAGTGACTGAATACAAGTGAGCTCTTATAAGTTATAAGTAGGTACATACAACAATTGCAATT

GGAGAATTGATCATGTAAGTGTTACAGCCTCTCGATTATGCAATATATTAACCTGTTGTGATGACAGTTC

TTTTGGACAATTATTCCTATGTAGCATAAATGTTTCTTGCATATGTACATTCTGGTATTCATTTCGCGAT

TTCATACGATAACTTGTTGTGATGACAGTTCTTTTTAGATACTTTTTATAAATATCATATGATAATTATG

GATAGTTTGCTCAAAATACC

>Ca_linc_0618

CTCAAATGCCTTATATCTGGGGTCACAGAAACATGTCCTAAAAGCTTATTAGCATTTTTAAGTTAAACAA

ATGATGTTGTAATATTTGAGAGTATTCTGTTGTGAGCTATATCCAAAGTGTTTTATCAGCGGCAGCAAGT

CATAGACGACCAAGGTGTTCTTCAAACTGAAGCAACTAATAATGAAAAGCAATGTTCATATACAGACCAA

GCACAAGGATCTCATCATATTCCTCCTCTGGATCTATCAAAGGTTCCTTGGTTAGATGGTCTACTTGATA

GTCAGGATGACTATGACCTGATTGCAAAAATTGATCAATTGTTGGTAGAAACTCAAAACATCAAAGGGAT

AGATGACATTGAAAATAATGACATCGAAGACTCGAAAATATGGTGGGACAGTGATTCGCAAAATATATTG

AATCCACAACAACTCTTGAAAGGATTTCCCTTGTTGGATGATTTCTTTCAAAGTCAGACTCTGAAAAGGG

ACGGAGAACACAACGAACAACCGAATCTATTTGTCTCACATCATCTCTTTCCTATGTTCTCAATAATCTT

TCCATTACATTAATTTCATTCTAATTGTCACACACCAAATTTTCTCTATTTGTGATGCATTTCTGTTTTT

CTTTTTAATAGGAATTCAGCTCACAGGATAGCTATATTGCTTGGGGTGGTACCAAGACAATCAACTAATA

CTTTCTTCACCTTTTGCAACTTTATTTTTTGATAAATTGCAAATTTTGAAATCAGGGGTAAATTCAATTG

TTACAACTTTTGTTGTAGTGGTTTGATACTTTGATCAACCAAGATGTAATCAATGCTTTCATTTTGTATG

ATGAAAGTTATAGACTTTTAGTTACTATGTTTCATTCAGGTTCTCAAATATTGATATGTGGACTTGACTT

GTCCACATAAAAAGACACATCAGACATGTTGCTTGCTTTGAAAGTAACAGATACATTGTGCATAT

>Ca_linc_0619

CAATTACCCAAATATATGAATGAAAAGTTGAAAATTTTAGGTTAGAAGTTCTGTTAGGGTTTGCTCACCC

TCACTATCGCCGCATCAACCTTTCGCCGCTTAGTCACACTTTCGTCTCTCGTTTCTCGTCTCTCAGCTTC

CCTCTCACTCTCCTTTGTTCCTAATGTAGTAATAACATGCTTAAAGTTGACAAGTGGTTTTCCACTCTTG

AAATGGAACCTCGTCTTGGAAAGTAAATCAATTTTGAGTTTAAAATGGAAAGTAAATCAATTTTGCATGT

GGAGTTGGAAAGGTAAGCTAGAAAGCAGAATCTTTGCCAGAGAATATTGGCGATGTATTGCAAACAAAGC

CTGCTGGTTTGAAGAAGAGTAAGTAGTTTTACCAAGCATAATCTTTTTTTAACATATTTCAATTCCACCG

CGTTATGTTTTTCTTCTAATTTGTATTAGGTGTACATAAGCTACTAAAATCACTCGGTGAATCCCCATGA

TGCAGCAATATTCTTTTGATTAATTTCATTTTAATTAGGTTGATTATGATGTAATTTATGTAATTTTTAC

TTGAGAATGTAATTTGAGACTGTAATTTGTATTGGTTGTGTTGTCTTGTGAATGTGACATGATGTTGCCC

ATAAATTTGTGATTTAGTATTTGTCTTAAACTTTTTGGCCAATGGCAAATGCTATGTCCTTG

>Ca_linc_0620

CGTCTTTCGCGATGGACTCTCAGATGCTTTAATTTGACTCTTTTTTTTTTCTTCACGAATTTTAGAACAC

CTTAGGTGAATCATAATGCATGCAATCCTACGATCAAAGCAGAGACAACAGCTTGACGGTAAAAATACCA

ATGACCTTGTAAATCTATGAGATGGTAAAATCTCTGATTTTTTTGGTATTTATCATTGACCTTTTTTTCT

TTCTATATGATTTATTTTATTTATCTATGTTCAACAAGTGTTGTTATTTATTTGTCAAAGGAAAATAGTA

GGTTTATATTAGTCGCATTAATTTTTACTCCTTGAATGTAGCATGTTGTCTTCTATGATGCCCAATGATG

TAAAAGTAGACTATTGATCGGCTTCATGGTTTTGTTCATAACTGGATTTGCACCATTCTTTCTCTCTCTC

TCTCTCTATGTGTGTGTGTGTGTGTGTGTATGTATGTATATAGCTTTGGCTCTCTCTTTCTTTCTGTCTA

TCTATGGTGCAAGAAGTTTGTGAATGAAGCCATTGAAAATGCCGTAAATGATTTTTATTAGCTATGAGAT

CTGAGGGCTTCGTGTTATTTATTTATTTATTTCATGTCTATATCTTGATATTCATTGCTATTGCAAAAGG

GGTTTTTGTTATGCTTAAGAAATACATACTGCTTAACAAAAATATGGTGTCATTGCATGAGGTTTTTTTG

CTATGCTTTAGAAATCCATACTGTAAAATAATAGTATGATGTCGCAGAAGCTCAAAATATCTAGTTTTAT

TGGCCCAGAGATAGACCTGAAGTATAATAACTTGTGTTGGTTATCTGCAATTTTTCTTTTACCAGCAGCC

TCTGTCCGAGTTTTTCTCCAAGTTTTATTTTGCCATTTGCTTATTTTTACTTGTCATAGAGTTACTTTCA

ATGCTGACTCCAGAATGAATGTGTGAAAGTTCTCTTTTAAAGCTGACTTATCATGTTAATGTTAATGTAA

GTAATGACTACATAACGAGTAGTTTGTAATTTTATAATCTTTATTAATTATTAGTATTTGATTTTCCAAT

GTATTACCCTTCTATTTCCTTTATCTCAGTGCTGTCTAATAGCAGCTAGAGTAACTCTACAGCACTATAG

CATATCGGGCTTTAAAATGGTGATCAGCTATCTTAAACTTTTGAGCAATTCTTCAAGTTGTATATTACTA

CTTCTAATTTCTTGTGGATTTTCTTTGTAATTGGTGACTGCATATTTTGCTTTTACTCTATTCCATATTG

CCTCTTCTGGATATGCCCAATGATGACTGCTTGTTGTGATATTGTACTCTGAATATCATTCACAGTCTTT

CCATCCATTCTGCCTTTCTTCACACTGCCATCACTAAGTTTCTTCTTATTCTTCTTCTTCATCATTGCTG

TCACTATCACCTTCATCATCATCATCATCATCATTATTATTATTATTATTATTATTAATTAATTCTTAGT

GACAGTGGGTTGAGATGGCGGAGGTTGCGGGCATTTGTGATGTGTGCACTGCCTTCATGGATGCTGAGAG

GCTTTTATGGATGTTGAAAAAGTACAAACATATCAATATCTGAGGGCTGAGATTTGCTGCAATACAATCA

AAGACAATCAATTTTGCTTGTTCAATTGCCTTGGGATAAATTTGCATATAATTCCTTTTGGATATGCCCG

ATGACGAATTTTCACTACTAGATATACTCCACTAAATAATCAATCAAAGCTTTTCCATCCATTGTGCCTT

TCTCCCATCTGTCATCACTAACTATGCTGCTGTTGTTGTTGTTGTACTACTACTATTTCTTCTTCTTCTT

CTTCAACATCATTGTTGTCATTTACTACTACTGACAGTGGGTGGAGATGATAGAGGTTGTGTGCTTGTTA

ATTATGTGCACTACCATGATGGATGGCGGGAGGCTTTTATGCATGTTGAAAAAGTACTAATCTACTGTTA

TCTGAGGGCACAGTTGCTATCTTGTATTTGAATAACTGTCAGTGATTTCTTTGTGATATTTCTTTGATAC

GATAATTTCATGAATCATGACAATCATCATTCCTGTTTTTTTGTGCTAGTATTGTATTGTCTTGTTAATT

CTAGTTTTGCATGTATTTGCCGCCCATGTTGGAGAGAATGATTGTGATAAGTTTATGTAAACTCCAAATT

TTCAAGTTTTGTTTAAGTGTGTTTATAATTAGAGCCACAGGTAGTAATGTTTGAAGTTGGACAGTGTATA

TTATTTAAGTTTCCCCTTGTTTTCTCTTTTGGTGTCTTTTTTTTTTTTATTACCGCATAACATTGAATTT

CTTCTGTTTCTAAACTTTTACCTTTCCTCTCCTAATCTCCTTTTCTACATATGGCAATAATTCTCCATGA

CTTTTGCAAATAAATTCAAGACTGCAATTGAGTGCTTCAATTTTAGAATCCTTCTAAAGGTCAAAAGGTC

AAGAATGCTGTCTTGTTGGGGAACTAAATATAGGGAATAGGTGGTTATTGGTGGTTGTGTAGGAGACAAT

GTTAGATGGGTCCCACCATTAAGACCTGTATTTGAAATGTAACTAACCAACTGGCTATTGAAATGTATAT

GGGATAGTCAACAAAGTCTTACTCTTGCATGGAGTGACTATTGAAATGTATATGGTAGAATATTTTGTAT

GTATTTTTACTATTGGGCAATGTTAAAATTTATCCTATATTGTACTTTAAAAAGGTAAGTAAATATATGG

TTAAGCA

>Ca_linc_0621

CTAAACTTATTCATTCCCATTCTCACTCTCACTACACTATCCCTTATACTTTGGCCATTTACATTTTCTT

ACTCGTCCATTATCAATCTTAATTATTTTTCATACCACCCAAAAAATTAACATGGCTTGTAAGATTGTGT

CTTTGACGGTTCCGGTAGCGGCGGCGAATGATGTCACCACCGTCATAGACGGCGGTGGCATAGGATGGAA

GGGTAGTAATGTAAATATGATTAAAAGTTGATTATGAAAGAATCAACACAAAAAAGGCTTTGCAATTGCA

CTCTCCCTAGTTTCTAGCCCATCAGAATTGGGATAGTGTCAACATTGCATTGCGGGGAATTGAGGAGATG

CATACGAGGTTGCCGGTTGGTTTTGGTGGTTTTTGGTTAAATGGGGATTAAGGAAATTTTGGGGGTTAAT

TTGGTATTTAATGCTTCTGGCATGCATCACTTACACCCATTAATGTAATATGTTTTTTTTTATTGGTGAA

AATTAATGTAATATGCTTTGTTATGAATTTTGTTCTATATAAAGGAA

>Ca_linc_0622

CAAGACGACAAACAATATCACAGAACCCGAATTCACTGCATCGATCGTTGAAACACGACAACTAAAACCT

TAACCAGATACAGCCATACAGCGATGATCCTGATGTGGCTGCTAGTGAGGTTCTAGAAATGTCAGCTGGA

AAAAATCACTGCTATGATAATGCCAGCATTCCTATTGTGACTGAAGCATAATGTTCTGAAAATGCTGCTA

ATTATGCTGTGAAGGGTGCCTTATACAATTCCAAACCTGCCTTTAACTAGAAAATTATGCTTAATGTAAA

TCAAAATCACTATTCTGGTGTGAAAGAAACGGGTTGTGCCTATGGGAATCCTTAGTAAATTTGCAGTAGA

ATTTTGAGTGGCTTATGTACCTTTCATAAGCATCATTTTGGTATACCAACACTAATTAGTGAGTTGGAGA

ATATAATACAGTGATATCATTAGTGCCTTTTACGTAGTGATTGTAACTATAAACTATAAACTATACCATG

TTGACAATTAAGTAATGCAGATACGGTAGGTCTCCCCCCTTTAATTATTTGATCTGGCATATTTGCATTA

GCAGACAGCAATTATCATGTTGACCAGAGAAGGACAATTATTTGTCATCAGCAGTAAATTATTGAGAA

>Ca_linc_0623

AGTAGAATTATTTTTTCAAACTTAACTTTTACTTCATTCCAAAAAAATTCCAACTTAAAAGCTATACTTG

GTTGGTTTTCAAAGTAGAAATAGTAACCTATGAAACTATGAGGGACCCTTTTGTCTTTTAGGTGGAAAAT

CAATATATGAAGAACTTTAACTTTAAATTTTCTTCCTTCTTACCTCTTACTGTATATATGTTGATGATCA

ATTTGCTCCTATCCACAACTTTCAGATCACTTCAAGCTCTTGAGATAAAAAGGTACAATTTTCTTTGCTC

TATGTATCTTATAACCATCTTTCATTTTGTGTGTAATAATTTGTATATAATTAATTACATTTAGTATTTG

TGTAGTGACATAATGTATAGATAGATATATATACTTTTGTGTGCCTGGCTCCCTGTATGCCATTTGTAGA

GTTCATCAAAATAGTGATGGCTTTAATGAATGGCGTGCGAGGAGCCATGCATGCTGTGTATATTTTCTAT

ATCCTGTACAAGATCTTGTTCTTAATTTTTGCATGTTATCTTAATTATGTTTCAATCTTTTCTTGTTTGT

ATTGTTTGGTTGTTTAATTTTCATTCACCTTTGGGAAGGGATGCAGGGGAAGCAGGGGTGTAGTAATTGA

TCTTTGGATTGGATCTTCTTTCTTTAGGTGTCTAGATGGATAATTAGTACTAATCTGTTTTTAATTAATA

ATGCAATGTTCAAGTGAATTTATGTATATAGAGCTTCATTGTTCAAGATGCCTTTGACTTGGTTAGATTA

GATAGTAGATAGGTAGGTTTTTATTAGGTATAGATAACAAATAATTTTTTAAATTGTGAATGTTGCTTTC

ATATTTTTGAACAAATATTTTAAATAAAATTAAAATAACAATGATTTTTGTTTGTGAAAATATGTTAGTA

TTTAGTATAGTTAGTATCACATAAGAATGATATGACAATTTTTGTTTTCATGTTTTGTTATATTACTTTC

TGACTTTATTTGATCACTTTAGAGGTTAATAATTGACTTATTAATTTGTCCATGTGTAAAGATCATTATC

ACTCTCAAGTGGACGTAGTCACCAATCGTTTTCGTAGTTCTGTTGTCATTTTTGTTGTAATTGAATAGTT

AATTGTTTATGTATAAACTCATCACTAAAAAATTGTTTATGTGTAAATATTAAAACACATTCATTACAGC

ACAGTAATGAGAAGCTTCTCAATTCAAATAATTAATTAATGATACACATTCTTTCTTATAATTATTAAGC

TTATTTGCTTCAGAAAATATGTATTGTTTTCACTATCTGTCTAAAGATGAAATTGCAAAAAAGAAAAAGT

ATTTTTGATTGATGGAGACCAAGGAAGTTGAAGTGGTGGTCCAATAGGGCCATAGTCCAAAGATGCAGTG

CAAGAAATTGAGAGAATCCCTGACAAGGGCCAACAAATTGAAAGCAGGAGTTCAGAGATTCCATGACAAA

AACAACTTGAAGCCTTTGTTTCGATATTTTATCGAATAATTATCAAAGTTCTAGCCCAATAAATAGGCTA

ATGATTGGTGTCATTATAAATAATGAGTTTCTTTTTCCACCATAGATACACTTTGGAAGTACATATTTGT

AAAAAAAAAAATGTTTTTTGCCCTCTAGATTTGTTCAAACATAATTTAGTTGCCCTTTGAATTTGTACTA

CGAGGCAGACATATATGATTTTTTTAAAAACATGAGGCCCAAATATCTAATTTTGTGTATGTCATTAC

>Ca_linc_0624

CTTAGACACAATACTATAGTGGAAATTTCCGCGAGTCCAACCAACCAACCATACCAAGTGATATTTATAT

TTTGCACTTATAAAAAGACAACCCCCACGAGCATCATAGCATCACCAACCTCAGTTTCAGCCACACACAC

TTTCCTTAAAATAACTCATTAACACTTTTGTTAATTAGTTCCATTCTTTTCCATGGCAAAGGGAATCAAT

TTCTTGGCATCATTTGTTGAGTTTGCACCTGCACCTTTGATTCAACTCAAAAAGATTTCACATGCTCCAC

TGTTAGAAACTATTGCGGAAGAAAAAGAGTCTGAAGATCAATAAGCTAACTACCAGTTATGTTGCTTTGT

GGTACAGAATATACAGTTCCCACCATCTACTGATCACTGTTTTTGTTGTCAATTTTGTGGGATAAATTTT

TGTTTGTTGATTTATCTATTTGTATATTAATTAATATTAGTTGTGTAATTTAATTTTGGTTATTTTGTTG

AAATTGTAAGTTAGTCGAAGAATCAACTAATAATTCTCAACTAATTAATGTTTCTCGTTTACTTTTAATG

ATTACATTAAAAAATGAACTCTCTCATTTGATTGTCACATCTTTAACATCTCTGACGAGTGTTAAATACC

AAGTTAAATTTTTTGTTGGTTACCAAATTAATGAAAACATAAAGCACACATAAGGATAAATGATAAATTG

TAAGAAAAAAAAAATGATAAATTTGATTGATTATTTCTGATTCTTAAATGTACAAATAAGTGTACACAAC

ACCCCGTATTTGGTTGATCTAGCAGATAGTGTGTATTTTTGTCTGTTACGAAGTGTACGTAACAACCTAC

ATATGGTCTTGATCTCCCTTCATTTCATGGCATGACAATGCCCCTCATCCACATATGCATTTCCACTCAT

ATTCAACCTCCGTGAAAATAACAGTTTCAGTTAGTTGTGGCTAAAAATATGGAACACAAGTCTTATAGTT

GAATCATCAATTCTCATGTGCTTTTGACAACATCTAAAAATCTCAGGACCCTCTTAGGGATTCTTGGACT

CGAGAAAAATTATCCTAACTCGTATTCGACTACCCCTAGTTTGCATGACCTTCATTCAGTTACTTGAGCA

TCGACTAAACCCCATGTGACTATTGATTGTTCTCGATTAACAACTTATATTATTTTTTAGCGTAAAAAAT

GCCTCTACATTGTATCTTCTATTTGAATTGATGTGAGAGGTGATTCAAATAATAATTATTGAATGAATAG

TGTCATTATTGCGAAGTACAATAGCCGTTGTACACTCTTCTTGGTTTAAGAATAGATGAAAACAAGTATG

ATTTTATCTTTTGCAGATCTTGTCTTGAAAGATAAGATAGTTGTTTACGACTTAGTTAATCATCGCATAG

GATGGACTAATTATAATTGTAAATTTACCACTTGAATTTTGTATTTCTTGCAAGTAGTATATGGTCCTTC

ATTATGTCTTAAAATTGAAAGTTATTTTTAGGTTCTATGCCCGTAAACGTCTCTGTGATCTTGAACAAGG

ATAAGTACAACAATCCAAGAGCAAGACGGTCAAGTGCCAGCAACTCATAGATAGGGATTTTCTCCATGTT

GTTACAAGTAACCATTGTGGCACTTTTAATGCACATAGTATAATCTGTATAGTCACCACATTTGTAACTG

TAATTGTAAACATGAGTACTCTGAAGCAGTTTTTGATTATTATGCAAGCATATCAAATTATAGTGGAATC

TATAGTATGATGTATATATGCTAGTCTTGTGATTGAACACTGATGAGAATGAAGACAACTAAAAACTAAT

AAAAAACCTCATTCCAATACACAGGATCTTAATGATATCACTGCCTACTTTAATTTTTTCTTTGGCTGAA

AATGGTTATTGATGTTGTTGTTATACTATTCACACATGGGGTATACTTCTCTTATCCAAAGAAATGCGG

>Ca_linc_0625

CGAAGATATTGCACATATAACTCCTATCAACACAATTGAGGAAACTGATGCATGGTGTGCTTGGAGCGAA

AACTTAGCAACAAAAACATGGAATAGGAATATAATGAGTTTAGCATGCAATCAAGTAACAAGAGGAAGAT

AGATCCTAAATAATAGTCTGAAAATGAGGATGAAGCCCTTCTAGATATATCGATTGAAGCTATGAACATT

GGTCAAAGAGGTGATAATGGACAATTCAAAGCAAACACCTTAACAACAGTCGAGGCAAAATTGAAGGAAA

TTGTTTTCTAGATGTGGAATTAAGAGTCATCCAAATGCAAAATATTTATTCGTGTTTTTTGGAAAGATCG

TGTTGCTCTTAAAGGAGGTGCCCATGCTGATAATATCGAAGAGATAGATAAAGAAAAGACATAAAAAATT

AATGACGAGGATCTTGAAGTAGAAAGTACTCAATCACCTTCTCTTTGACAATCACAATCTGGGAGAAAGA

ATGACCAAGTGGAAATTAATTCCATCAAGGAAAAGAAATGGAGGAGCACATATTATTACTAATGATATTT

TCAAGTTAGAAAAAACTTTTGGCAATTGGCTTGAATAAAGTTTAGAATGATTCGGTGAAGATGCAAATCG

TCT

>Ca_linc_0626

TGGCGTCAGTGTCTGCTGCTGCTTCGATAGTGAAGAATGAATTGAGGAGTCATGAAGTCGCCATAGCCGA

ACTCAACGCCCTTCCTTCTTCCAGAGTCAGTCTTCTCATTTTCACTTTCACGCTATTCTCCCAATTCAAT

ATATATACATAGGACTGTCTATCAGAGAAATGGGAACTTGTTTTTTCGCACATCTATCCAAACAGCAACA

AGCATGGAGCAGAAACAACTTGATTCAGCCAAAGCCAAGCTAAAGAATCTGAATTCTTCGTCGAAATCAT

ATGCTAGAACTTGGGTGGTTCAGTAGTAATGCGGCATTTTTCTGTGAGATGAGGGCGATAAGGACATGTT

ATATGTTATCACTTGGAGACTTCATTTTCTAAATAGCTGTTAGGTCCTAGATACTGTAAATTACATAAAA

TGCATACATACAATAAATATGTTAAGGATATTTTGAATTTTTTTAGTTATTTTCACTGAAACACTGAAAT

GTGTTGTAGGCGTGACTTTTAATACAATTGATAAAACTGTATTCATTCATCTATTAAAACTTGCACATTT

CATAATGTATGTAAAATGCACGGGCTTTAATTTTATTGTCCCTTCTTGATTCACGTTTAATAACATATTT

TGAAATGAAACCAA

>Ca_linc_0627

CAAAACCTTCAACTACAAAATTGTTTCACTACTTGAAAGGAATGCAAAGAGCTAAGGATCAATAGTTGAG

ATCGTGACCCTCTACTATTATTGTGGATTGGGCTTACCCCCCAAGTTCTTAGAGTTTTGAGTCGTGCGAG

AATTACAACGACGTCGAAGACATGACAAAACAATGATTTTGGATTCGCAATTGGAAGCAGTGAGGCTTGA

TGCCACCCGTAGACCATCAGTTTCTAATCATCCTCAGTTAGAGTTCAAGATCTGCTTAGGTTTTAATTAA

GAGTTTTATTTATTCTCCCTTCCTTATTGCAATGTTGTCGTTGCCATGGAATTTGTGTATCTGAAATAAT

TGTGATTTGTTTTGGGTCAATGTGAATTGTTCGGTTGTAAGTTATTTGTGTCGAAACCATTTTATATAAT

GCAACAAAAATTCTAATCGTCGTTTTTGGAAAAGAATTTTACAATTAAATATTTTACTCTGATTTAAATA

AAATACGATTATGAATATTTTAGCCTAA

>Ca_linc_0628

CTCATCCGGCACCCGTGTTTGCTCATTTTTCTGGCTTTTTACCGGATCTTAGCATCCATTAGTGTGAAAT

TGAGGAAAATACACTACCAGATCCTAGCTTCCGGTAATGTATTTAGGGGCGAGTGCGCAAACACGGGGGG

CAAATGAGCAACCTTCTGAGCTTTGTAATTCTCTTGAGGAGTGTGTGATCTCCATATTACCTTTTTTCTA

TCTAGTGAAAACTTGTTGCTTATTGTGGTTGTAGCCCACACTAGATTAAAATGTTGTTGTGTTCTTGTGC

TTTCTTATTCTTCTTGTTTGATTTGATTTTCAGCATCTTAATTCATTTCTGCAATTGTGTTTCTCGGGTT

ATTCTTTTGAATTACATAATGGTAAGCTCTTTCAGGGCACCAATCCATGTACTCATACTTCAGACATTAA

TCAAGCTAAATTCCACTTGGGAAAAACTATCAAAACATAAATATATGTATATATTTCTTTTTCCAAAACT

TATCATTATGCGAACAAGATGATGATAAGTTTTGTGGAAAAAGAATTATTAAGAACAGAAGCAGAACAAA

GAAAGGGGATTTTAGGGTTCTGCTGCTTGATGATTAGATTGGGAATTAGAATTCCATGACAACTATTAAG

TTCATGCTTTTGTAGAGTATAGCATGAGCTATTAGATCCTCTACCGACTACAGCTAGAGTGATATGGTGG

CTTGGTGGTTTAGAGGAGAGACATATGAGTTAGAGTGATAAGAGGTCATGGATTTTAGTCTCCCACCAAC

AAAATTACAGCTAACAACTAACATTTGTCATTCAAAAAAATAAAAATCTTCTATCACCGGTTGCAGAGCC

GTTGAATTAAATTGTAATGAGTTTTGAAATTCAGATTTAACGGCTCTGCAGTCCGATGGAGCTCCAGAGG

ATCTATCTTGGTTACATTCTTACAGGTGAGGTTTTATCCACATGAGTCTCATTGTGAATTTTTACTTTTC

TGATTAAAACATTATTTAAGTTAAATATTTCTGTTAAAAAAAACTTAAATACAATTTACACATAGTTATT

TTGCAATCTAGTTATGGCATGGCTTCATCATGAACAGATTTATCTTCTTTTGGTGTCTGCATTTGTTGGC

TGCTGTATTTTGTAGCTTAATTAAGCTTTTGTTGGTTGTTTGTCAAGAGCAACCTCTAAAACAGCAACAT

GGAAAAGTGAGACCAATTGCTGCTCATGGCTTGATGTCAGCTACAACACCATATTTGGTCAATTATTACT

AAACTTTGAATCCAATGTCAATTACAATTTTTCTATCCTATCAACATTGAAATTGTCTTCTGTTGGTGTG

ATGGAATTTCCAAAATTATTAAGAAAATTTCCCAGTTTGAGATATTTTGATATTACAATAAAAAACTTAA

TGGAAGAATCCCCAATTGGTTACTTGAAATAGATTATTACAATTTTTTGGCTTCTTTCGAAACTTGTTCA

TGTCAATAGACCAATTCTCAAGGAATCATTCTGTCAGTTTGCAATACAAGTTCACTTGAAATGTTCAACT

TGGCACACAAAATTGGAAGGTATCATTCCACAATGTCTAGCAAATTTATCATCCCTTCAAGTTTTGGATC

TATAAATGAACAAGTTTTACGGCACTTTCCCAACAACAAAATAGAAGACCACTTTCCTAACTAGCTTCAA

ACTCTGCAAAATTTGAAAGTATTTGCTTTGAAATGAAATATGGACCTGAAGAACATTCTCCACCTTCATC

CAACTTTTGTAGTGAAGAGAAATTTGGATTTGGATGAAAACTGGTGGCTATTGGATATGGATGTGGAGTG

TTGTTTGGACGAAAGTTAAGCAGATCAAATTATCAATCAGGGCAACTTCTGCTACAACTCATGTGTCATT

CTGCTGGGTGACTGGCTATCTGTTATTCAATTGAGATCCTGGGATTGTGAGCATTGCAAGGAAAACTAAG

AATAAACTAAAATAACACTAACTACTTTTCTTGTGGTTCTTGAAATCACTTTTAAATTTATTGTCTATAT

TGTAGCTAAATACATAGAATTTAT

>Ca_linc_0629

GAAAAGAGTAACAAACTTTTGGCTATATTCACTATATAAGGAAAAGCACCCAACAACATAGGAAATAAAT

TATTTATGAAGAATCTAGATTCTCTCCACTTGACAAATTTTAAAATGTCGTTTCAATGTGAATTATGTAG

TGTGCAGTCCAAATTGGAGCTGTATTTGGAGCTATAACTCCTAGGGTTGGACAAATTTGAGATAATTAGC

CCAACCCAACCGACCTGCAAAAGAAAAATGGTGATCGAGAGGGTGGGGCGGATGACGTGGACATTTGTGA

TTGGGTCTGTACGAGCGGGTTCATGTGAGTTAATTTGGACTGGCGGTGTCCGAACGGGGTTCACCCAATA

TATATAGCAAATAGGTGAGTTATTTTAAATAAGAGGAAACTCAATTAACCGAGTCCAAGACCCAAGACCA

ACATAGAAATCCTAAGTATAAAAAGCAAGGTTTCAGAAAAACTGAAACCTAGTTTTGTGGATCCGCCTCT

AGCTCTAGTGCTCAAGTATCATCTCTTACCTAAGTTTTGTTTCTTCGTTCTTAATCTTTCTCTCTTTCTC

GTTTTCATCTATAAAACTCAAAACTTAGCCTCATTTGCATCTTGTCTCTCCACTTTTCTCTATTACGTTT

TTAGTTTCAGTAAAGTTTTTCATTCTTAAACTTATACTAACAATGAGTTGTTTGTATTTTTATTTTTATT

CTAAACCGGACCATGCTTGAAGTGAAGGATTGCAACCATAAGAAAATGATTTGAAAGCTTAGTTATCTCA

AAATCGAACCCTAGTGTCCTTTGAATCTTGTTCTTCCCCTTTCTATTTTACTCTTTTATTTTTTTATTTT

TTTATTTCATTGTTAGAGTTTTCAATTTTGAACTTATACTAATTTGTTCGTGTTTTTTCTTTTTATTTTA

AATAAATCGGACAACACTTGAAATTGAAGGTTTGGAGATTAATATAGCAACACCATTTTAAAGGAACAAG

GTGATGTGCTAGAATTGAATGGAACAACCTGTTGCAACATGGATCAAGCTTCTTGAACTATATTTTGTAT

TTACATTTTAAAGTTCATGCGATGATGTTATTTGATACTAGATAATTGATACATTAAGTGTATTATATGC

TATGTATAAATGTTTTATGCTATATTTAAGTGTCTTAAATATTATCTTTAAGTTATTGGTACATGCTCAT

CAGTTACTCCTTTATTCCAAAATCTGTGTACATGTAAAATAATGAAAATTGAATAAACACATTTTCCTTT

TTATCTCTTTGTAAGTGTCTTATGTAATATTTATGTATCTTGATTTGTATGCTAAACTTAGTTTAAG

>Ca_linc_0630

CAAGAACATGAGTTCAGAAACGTAAACCCTTGAGAAGTCAAATGTGAATCGAACCCTAATCCTAAATCAT
[truncated: 1,057,478 more chars]
